# Catalytic Asymmetric Spirocyclizing Diels–Alder Reactions of Enones: Stereoselective Total and Formal Syntheses of α-Chamigrene, β-Chamigrene, Laurencenone C, Colletoic Acid, and Omphalic Acid

**DOI:** 10.1021/jacs.2c01971

**Published:** 2022-04-07

**Authors:** Santanu Ghosh, Johannes Eike Erchinger, Rajat Maji, Benjamin List

**Affiliations:** Max-Planck-Institut für Kohlenforschung, 45470 Mülheim an der Ruhr, Germany

## Abstract

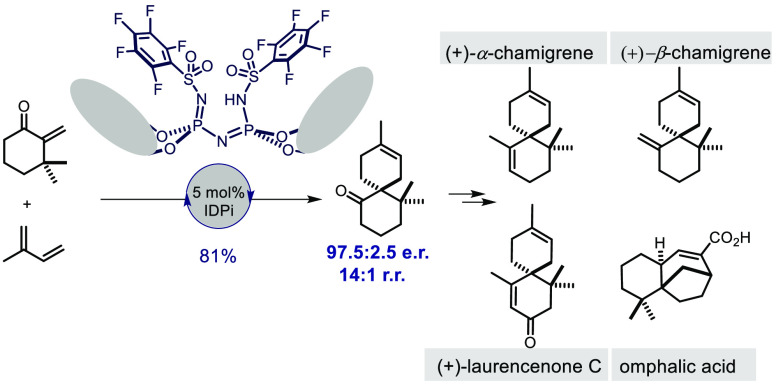

We disclose a general catalytic enantioselective Diels–Alder reaction of *exo*-enones with dienes to give spirocyclanes. The obtained products feature highly congested quaternary stereogenic spirocenters and are used in concise total and formal syntheses of several sesquiterpenes, including of α-chamigrene, β-chamigrene, laurencenone C, colletoic acid, and omphalic acid. The stereo- and regioselectivities of our spirocyclizing cycloaddition are effectively controlled by strongly acidic and confined imidodiphosphorimidate catalysts. Computational studies shed light on the origin of reactivity and selectivity.

The catalytic enantioselective, step-economic, and atom-economic construction of spirocyclanes is an interesting problem for chemical synthesis due to their unique structural characteristics and biological activities.^1,2^ Particularly, spiro[4.5]decane and spiro[5.5]undecane skeletons have attracted considerable attention, as they represent the core structure of several biologically active sesquiterpenoids, such as acoranes, spirovetivanes, omphalene, and chamigrenes (Figure 1).^3−5^ However, the presence of a spiro-fused quaternary stereogenic center, which is often sterically encumbered by neighboring quaternary and tertiary centers, imposes a significant challenge toward the straightforward catalytic and enantioselective construction of such spirocyclanes. Previous approaches typically relied on either a stepwise formation of the quaternary stereocenter followed by an intramolecular spirocyclization, or diastereoselective transformations of enantiopure precursors.^5^ Direct, catalytic enantioselective cycloadditions would provide a more strategic and potentially powerful solution to this problem. However, catalytic enantioselective spirocyclizing Diels–Alder reactions of *exo*-enones with dienes, to the best of our knowledge, are unprecedented. Here we describe a highly stereoselective spirocyclizing Diels–Alder reaction that is catalyzed by strongly acidic and confined imidodiphosphorimidate (IDPi) catalysts. We show that the products of our reactions are useful precursors of several spirocyclic natural products.

**Figure 1:**
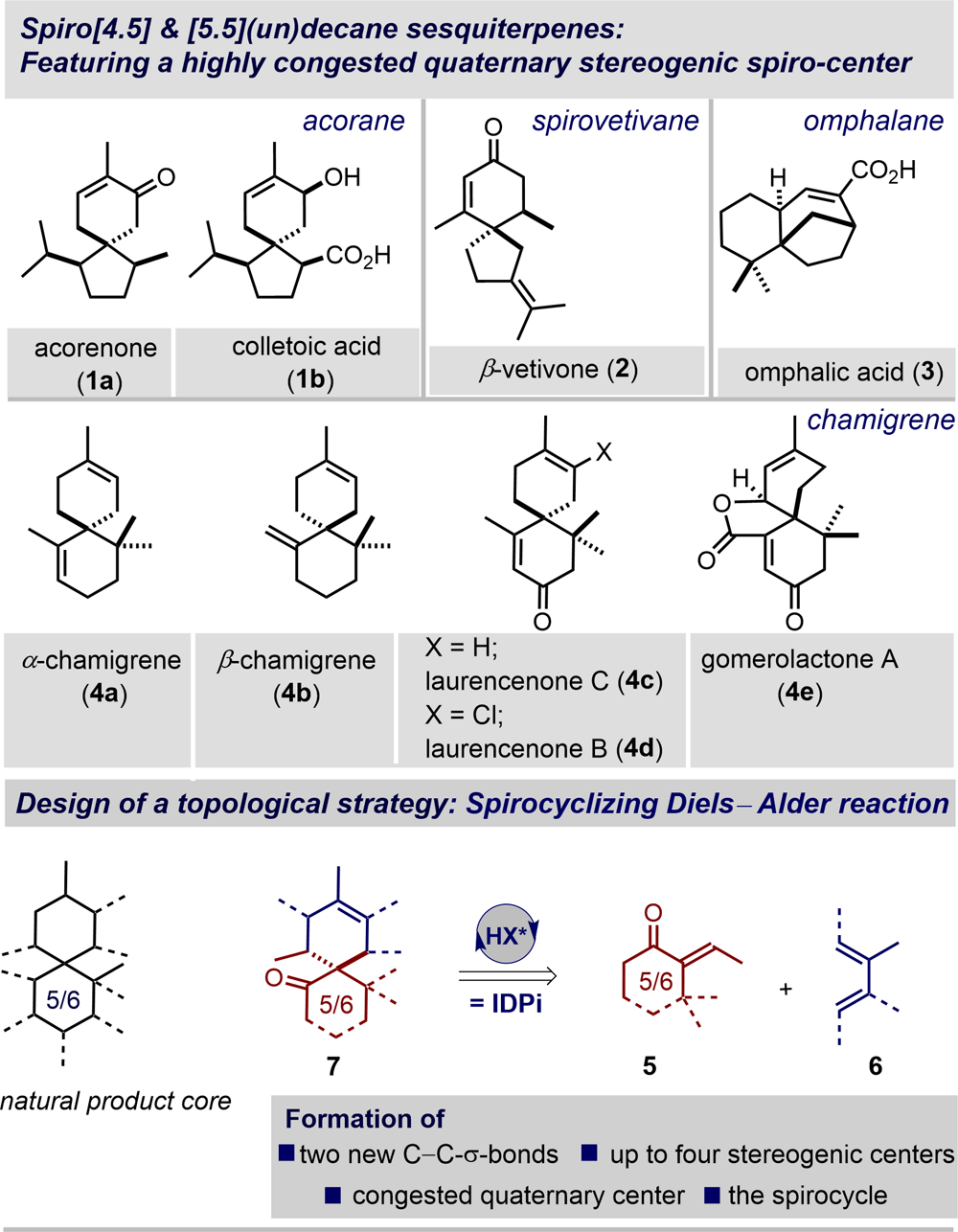
Spiro[4.5]decane and [5.5]undecane sesquiterpenes; design here.

Given the unique high reactivity and stereoselectivity observed in confined acid Diels–Alder reactions,^6^ we envisioned that enantioselective cycloadditions of *exo*-enones **5** with various acyclic dienes **6** would constitute an atom-economic topological approach to spirocyclanones **7**, potential precursors toward the acorane (**1**), spirovetivane (**2**), omphalane (**3**), and chamigrene (**4**) natural product families (Figure 1). If realized, this disconnection would strategically enable, in a single step, the simultaneous formation of two new C–C σ-bonds, up to four contiguous stereogenic centers, including the critical spiroquaternary center, and of the spirocarbocycle itself. However, despite the remarkable progress made in advancing asymmetric Diels–Alder reactions of α,β-unsaturated ketones,^7^ the use of α-alkylidene exocyclic ketones has not previously been reported. Given the proven ability of confined acid catalysts to activate small and challenging substrates, we hypothesized that these acids may also be suitable to protonate such *exo*-enones, which could then engage in the desired asymmetric cycloaddition within the chiral pocket provided by the catalyst (Figure 1).^8^

We commenced our study by exploring various chiral Brønsted acid catalysts in the reaction of (*E*)-ethylidene cyclohexanone **5a** with myrcene (**6a**) as challenging models of dienophile and diene, respectively (Table 1). As anticipated, most of the conventional unconfined and/or moderately acidic chiral Brønsted acid catalysts such as chiral phosphoric acid (CPA) **8**, disulfonimide (DSI) **9**, and imidodiphosphate (IDP) **10** were essentially inactive (Table 1, entries 1–3). In contrast, the significantly more acidic IDPi **11a** gave 67% conversion and furnished the desired product **7a** in 44:56 e.r., >20:1 r.r. (*para*:*meta*-addition) (Table 1, entry 4). We next set out to fine-tune the 3,3′-substituents of the IDPi catalyst backbone. Gratifyingly, a beneficial effect on the reactivity was observed using catalyst **11b** with Ar = 4-SF_5_-C_6_H_4_, which also showed promising selectivity (Table 1, entry 5). Additional efforts focused on the inner core (R), where introducing a pentafluorobenzene (C_6_F_5_) substituent in catalyst **11g** led to further selectivity improvement (Table 1, entry 10). Finally, lowering the reaction temperature from −20 to −60 °C, increased the e.r. to 96:4 (Table 1, entries 10–12). Hence, using the conditions applied in Table 1, entry 12, we proceeded to further explore the reaction scope.

**Table 1:**
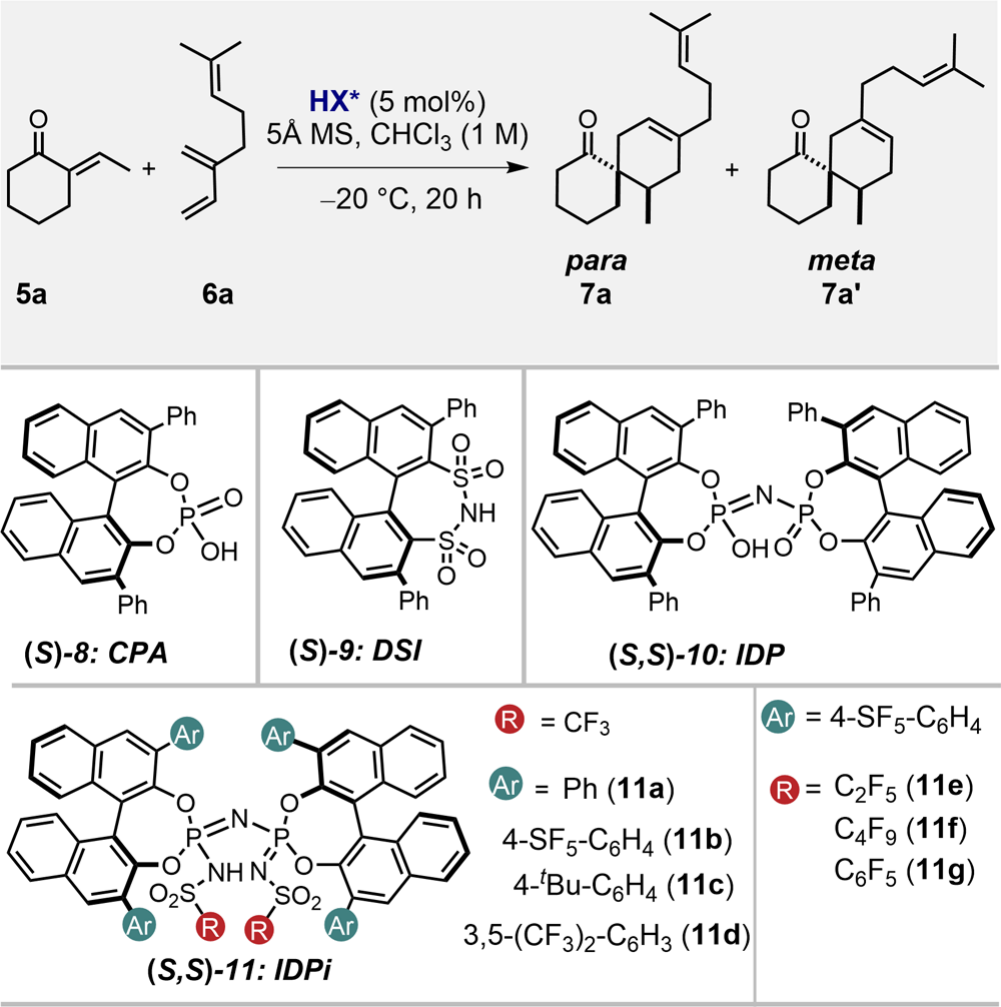
Reaction Development^a^^,^^b^

entry	HX*	temp. (°C)	conv. (%)	r.r.	e.r. (**7a**)^c^
1	**8**	–20	trace	–	–
2	**9**	–20	trace	–	–
3	**10**	–20	trace	–	–
4	**11a**	–20	67	>20:1	44:56
5	**11b**	–20	full	>20:1	64:36
6	**11c**	–20	9	–	–
7	**11d**	–20	full	>20:1	51:49
8	**11e**	–20	full	>20:1	69:31
9	**11f**	–20	full	>20:1	71:29
10	**11g**	–20	full	>20:1	93:7
11^d^	**11g**	–40	full	>20:1	95:5
12^e^	**11g**	–60	full	>20:1	96:4

a Performed with substrate **5a** (0.025 mmol) and molecular sieves (MS). Conversions (conv.), and regioisomeric ratios (r.r.) were determined by ^1^H NMR analysis with anisole as the internal standard.

b *Para*-regioisomer formed as the major product, confirmed by NMR analysis (see the SI).

c Enantiomeric ratio (e.r.) measured by GC or HPLC (see SI).

d 48 h.

e 72 h.

First, we investigated various exocyclic enone dienophiles with myrcene as the diene (Table 2A). To our delight, different (*E*)-alkylidene substituent (e.g., methylene **5d**, ethylidene **5e**, propylidene **5b**, **5f**, and pentylidene **5g**) of the 5- and 6-membered cyclic ketones were well tolerated and delivered the desired products **7a**, **7b**, **7d**, **7e**, **7f**, and **7g** in up to 94% yield, 99:1 e.r., and >20:1 r.r. α-Benzylidene-substituted enone **5c** was also examined and proved to be less reactive (22% conv., 12 d at −50 °C). However, upon introducing acidifying 6,6′-*i*C_3_F_7_ substituents, catalyst **11h** gave product **7c** in reasonable yield, good enantioselectivity, and excellent regioselectivity. Gratifyingly, our efforts to use tetrasubstituted enones such as cyclopentanone **5h** were rewarded when we found that catalyst **11i** gave the corresponding cycloadduct **7h** with two vicinal quaternary centers in moderate yield and with excellent enantioselectivity and regioselectivity. Interestingly, sterically congested 2,2-dimethyl-substituted enones **5i**,**j** also produced adducts **7i**,**j** in good yields and enantioselectivities, albeit in diminished regioselectivity of up to 5:1. This observation may suggest that a sterically demanding quaternary center in the α-position to the ketone unfavorably interacts with the active site of the catalyst. More importantly within the context of our sesquiterpene disconnection, however, enone **5k** with a quaternary center at the α-position to the olefin was well tolerated. The corresponding product **7k** was obtained in good yield and excellent regio- and enantioselectivities (82%, >20:1 r.r., 98:2 e.r.). Remarkably, substrates **5l**–**n**, featuring both an *exo*- and an *endo*-enone functionality, readily furnished spirocycloaddition products **7l′**–**n′** in excellent yield, enantioselectivity, and regioselectivity (up to 91%, >99.5:0.5 e.r. and 18:1 r.r.). Strikingly, in these cases, the *meta*-regioisomer **7′** was obtained as the major product. We also explored a 1-tetralone-derived enone, **5o**, which again provided the *meta*-regioisomer **7o**′ in 87% yield, 18:1 r.r., and 93:7 e.r.

**Table 2:**
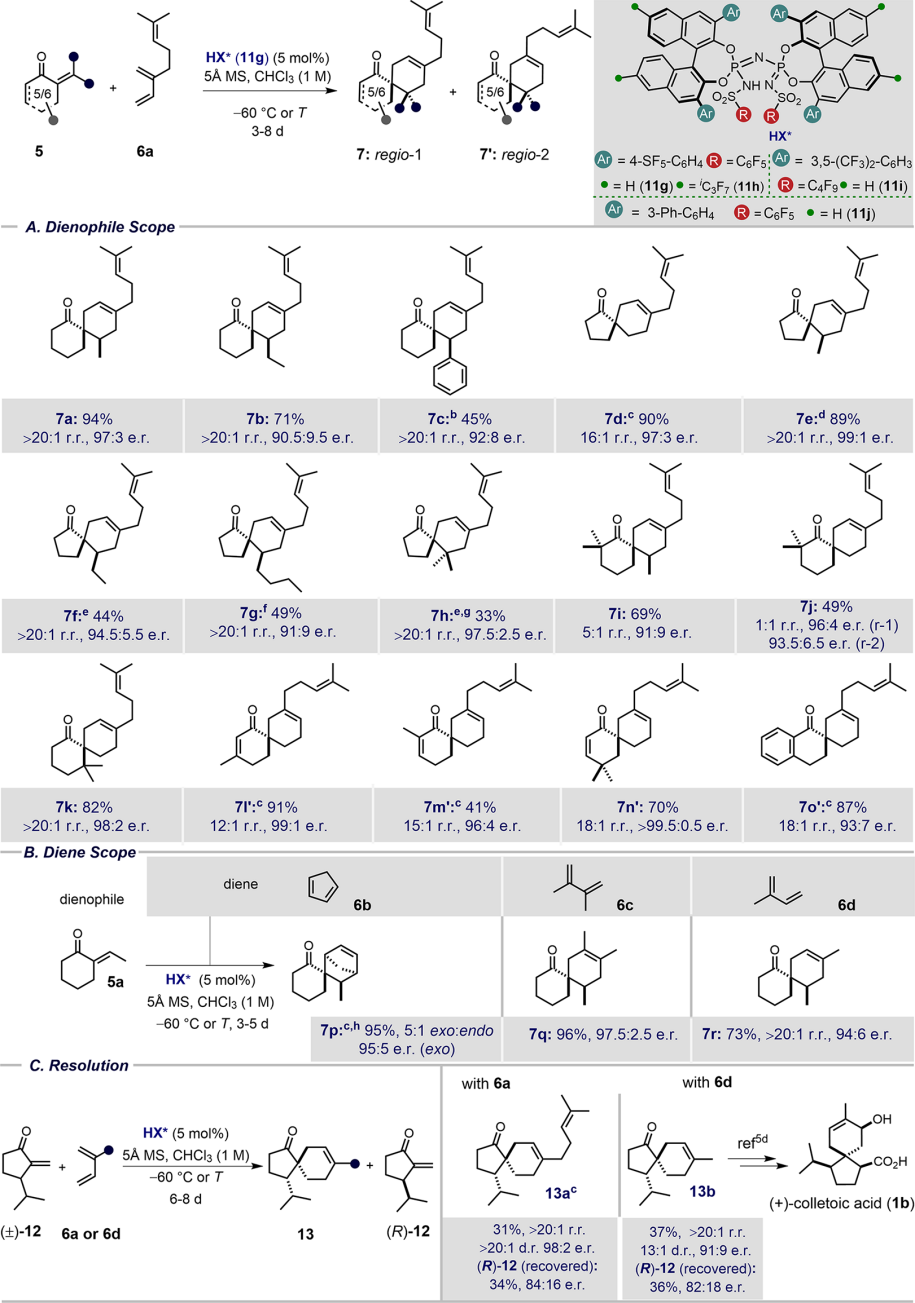
Substrate Scope^a^

a Reactions on 0.2 mmol scale (see the SI for details). The relative configurations of **7a**–**k**, **7p**–**7r** were assigned by analogy in comparison with compound **14** (*vide infra*). The absolute stereochemistry of **7l′** was determined by NMR spectroscopy of Mosher ester derivatives (see the SI), and the configurations of **7m′–o′** were assigned by analogy.

b With catalyst **11h**.

c At −80 °C.

d At −50 °C.

e At −45 °C.

f At −20 °C.

g With catalyst **11i**.

h With catalyst **11j**.

Second, we explored other diene classes with dienophile **5a** (Table 2B). We chose dienes with a broad spectrum of Diels–Alder reactivity, including cyclopentadiene (**6b**, *k*_rel_ = 1350), 2,3-dimethylbutadiene (**6c**, *k*_rel_ = 4.9), and isoprene (**6d**, *k*_rel_ = 2.3).^9^ To our delight, all of these dienes furnished the corresponding cycloadducts. In the case of diene **6b**, catalyst **11j** effectively produced adduct **7p** in moderate diastereoselectivity (5:1*exo*:*endo*), with excellent yield (95%), and enantioselectivity (95:5 e.r.). Both dienes **6c** and **6d** proceeded with a very high degree of stereocontrol and yields of up to 96%, >20:1 r.r., and 97.5:2.5 e.r. under the optimized conditions with catalyst **11g**.

We also became curious to explore the possibility of a kinetic resolution of racemic enone **12** using our optimized catalyst **11g** (Table 2C). Indeed, reacting *rac*-**12** with diene **6a** gave the corresponding spiroadduct **13a** in excellent enantio- and regioselectivities, while (*R*)-**12** was recovered in 34% yield with 84:16 e.r. Furthermore, the use of diene **6d**, produced cycloadduct (−)-**13b** in 37% yield, 13:1 d.r., >20:1 r.r. and 91:9 e.r. The remaining enone (*R*)-**12** was recovered in 36% yield with 82:18 e.r. Notably, cycloadduct (−)-**13b** has previously been used in a total synthesis of (+)-colletoic acid (**1b**).^5d^ With our kinetic resolution, we therefore have accomplished a formal synthesis of (+)-colletoic acid **1b**.

We next explored our newly established methodology in an approach toward several chamigrene sesquiterpenes. The cycloaddition between enone **5k** and isoprene **6d** was conducted on a 4.4 mmol scale (Figure 2A).^10^ Product **14** was obtained in good yield (81%) and excellent enantio- and regioselectivity (97.5:2.5 e.r., 14:1 r.r.; and >99.5:0.5 e.r., >99:1 r.r. after preparative HPLC purification) (Figure 2A). Importantly, catalyst **11g** has been recovered in 95% yield and full catalytic activity (see SI). Spiroadduct *rac*-**14** has previously been used in the total synthesis of omphalic acid (**3**).^11a^ Hence our method offers an enantioselective synthesis of omphalic acid (**3**), which still has to be accomplished. Enantiopure ketone **14** was also transformed into (+)-β-chamigrene (**4b**) in 94% yield using a Wittig methylenation (Figure 2A). Notably, our synthesis represents the first catalytic enantioselective route to (+)-β-chamigrene. Subsequently, we focused on synthesizing (+)-α-chamigrene (*ent*-**4a**), employing a two-step sequence. Accordingly, upon treatment with KHMDS and PhNTf_2_, enone **14** was converted into enol triflate **15** in high yield (Figure 2A). This compound was then subjected to an iron cross-coupling reaction with methylmagnesium iodide, leading to the formation of (+)-α-chamigrene (*ent*-**4a**) in three steps and 28% overall yield starting from enone **5k**. Furthermore, we were keen on synthesizing (+)-laurencenone C (*ent*-**4c**), the enantiomer of the natural product, using the same precursor **14**.^11b^ Thus, ketone **14** was converted into the corresponding silyl enol ether, which was directly oxidized by Pd(OAc)_2_ to give enone **16** in good yield (Figure 2A). Treatment of compound **16** with MeLi resulted in a 4:1 mixture of the corresponding 1,2- and 1,4-addition products **17** and **18**, respectively. Finally, oxidative transposition of the tertiary allylic alcohol **17** with pyridinium chlorochromate (PCC) completed the total synthesis of (+)-laurencenone C (*ent*-**4c**) in five steps and 23% overall yield starting from enone **5k** (Figure 2A).

**Figure 2:**
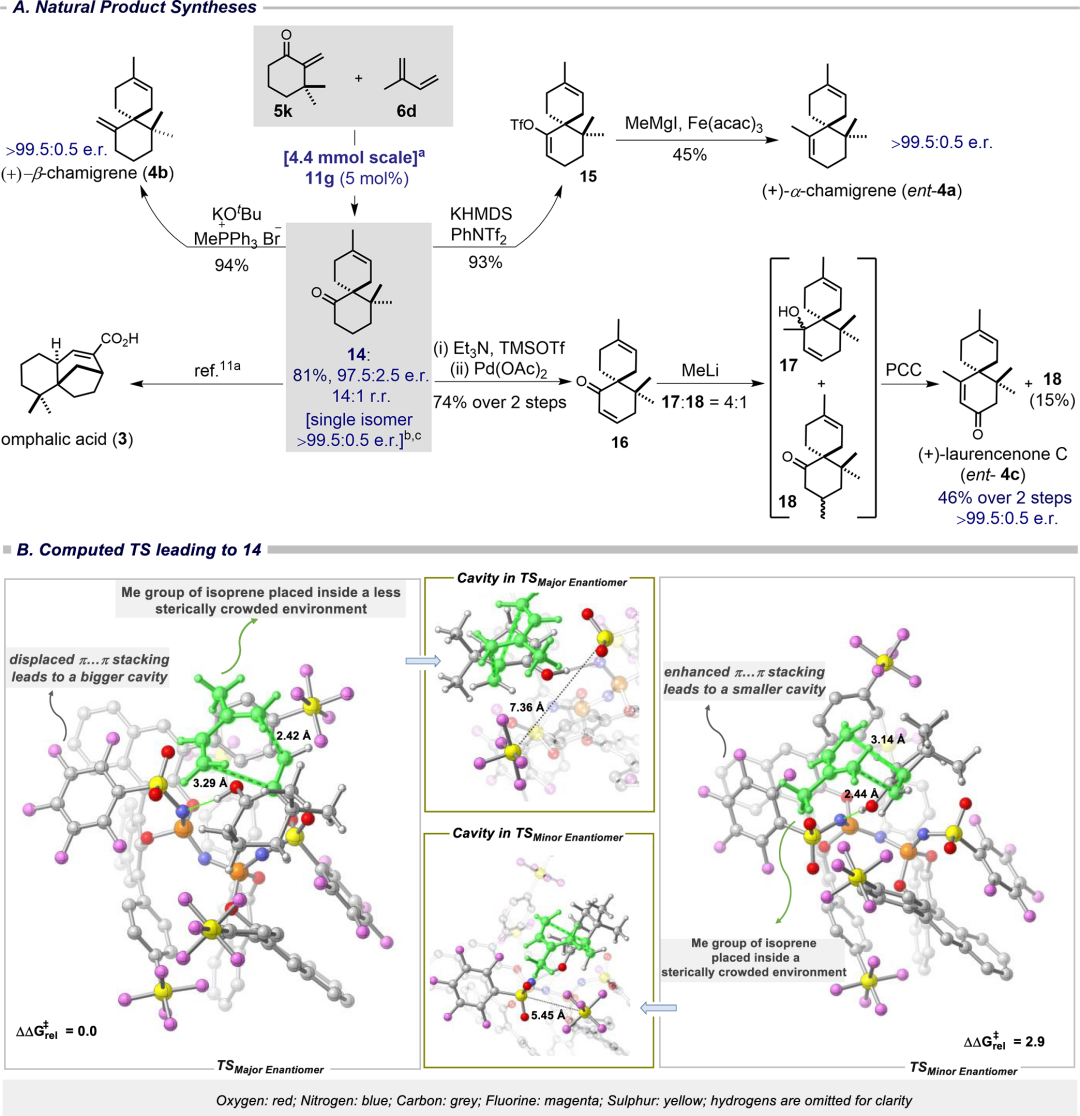
(A) Synthesis of (+)-α-camigrene (*ent*-**4a**), (+)-β-chamigrene (**4b**), and (+)-laurencenone C (*ent*-**4c**), and formal synthesis of omphalic acid **3** (see SI). ^a^5 Å MS, CHCl_3_:pentane (1:5) (1 M), – 60 °C, 7 d (see SI). ^b^Preparative HPLC was used (see SI). ^c^The absolute configuration of **14** was assigned from the natural products **4** in comparison of their specific rotation.^6^ (B) Computed structures (B3LYP-D3(BJ)/def2-TZVP + CPCM(chloroform)//PBE-D3/def2-SVP level of theory) and the relative free energy of the enantiomeric transition states leading to **14**. Key reactive atoms are highlighted in green. Energies in kcal mol^–1^.

Toward a molecular-level understanding of our reaction, we resorted to computation. DFT analysis has been performed on the IDPi-**11g**-catalyzed formation of compound **14** (Figure 2A). Computed free energy differences at the B3LYP-D3(BJ)/def2-TZVP+CPCM(chloroform)//PBE D3/def2-SVP level of theory between the key enantiomeric transition states (TS) is in line with that of the experiment (experimental and calculated e.r. values are 97.5:2.5 and 99:1, respectively). Furthermore, the computed r.r. value of 17:1 (ΔΔ*G*^⧧^ =1.2 kcal mol^–1^) is in excellent agreement (see SI) with the experimentally determined r.r. value of 14:1 (ΔΔ*G*^⧧^ =1.14 kcal mol^–1^). Our computational studies suggest that the reaction proceeds via an asynchronous concerted TS arrangement where the bond formation between the β-methylene carbon of enone **5k** with the C1 of **6d** proceeds earlier than the α-carbon of **5k** with the C4 carbon of **6d** (Figure 2B. Both the diene and dienophile engage in several non-covalent interactions within the confined catalyst’s counteranion cavity (CH··π, CH··O, CH··F; see AIM analysis in SI). The stereoselectivity for this reaction is primarily controlled by steric factors, leading to a favorable approach of **5d** to the catalyst bound dienophile from the less congested face. Notably, in TS_major_, the counteranion adopts a displaced stacking arrangement that significantly widens the cavity (*d*_av_ in TS_major_ and TS_minor_ are 7.36 and 5.45 Å, respectively). Such conformational change for substrate binding is reminiscent of enzyme catalysis and helps to stabilize the incoming isoprene by preferentially placing its methyl group in a relatively open quadrant. Additional study using distortion interaction (DI) analysis^12^ also suggests that the TS leading to the major isomer is more stable due to the less substrate distortion, supporting the steric-guided selectivity model (see SI).

In summary, we have developed an efficient Brønsted acid-catalyzed enantioselective intermolecular Diels–Alder reaction of diversely substituted *exo*-enones with various challenging dienes that enable the rapid construction of enantiopure spirocarbocyclic scaffolds of bioactive sesquiterpene natural products. The high acidity and confined chiral microenvironment of the IDPi catalyst controls the regio- and stereochemical outcome of the process and leads to good to excellent yield, enantio- and regioselectivities. A challenging tetrasubstituted enone also provided the corresponding cycloaddition product in excellent regio- and enantioselectivity. DFT analysis has been undertaken to rationalize the stereochemical outcome. The applicability of our strategy is demonstrated with step-economic constructions of (+)-β-chamigrene **4b**, as well as of (+)-α-chamigrene (*ent*-**4a**), (+)-laurencenone C (*ent*-**4c**), and the enantiopure precursors for the synthesis of (+)-colletoic acid (**1b**) and omphalic acid (**3**).

## Supporting Information Available

Experimental details and analytical data for all new compounds, NMR spectra, GC and HPLC traces, computational studies, optimized structures, and Cartesian coordinates (PDF)
