# Supplementary material for: Catalytic Asymmetric Spirocyclizing Diels–Alder Reactions of Enones: Stereoselective Total and Formal Syntheses of α-Chamigrene, β-Chamigrene, Laurencenone C, Colletoic Acid, and Omphalic Acid
Source: J Am Chem Soc. 2022 Apr 7;144(15):6703–8. doi: 10.1021/jacs.2c01971 (PMC9026245; doi:10.1021/jacs.2c01971)
Supplement: Supplementary file 1 — ja2c01971_si_001.pdf [file ja2c01971_si_001.pdf]

Catalytic Asymmetric Spirocyclizing Diels–Alder Reactions of Enones: Stereoselective Total and Formal Syntheses of *α*-Chamigrene, *β*-Chamigrene, Laurencenone C, Colletoic Acid, and Omphalic Acid

Santanu Ghosh, Johannes Eike Erchinger, Rajat Maji, Benjamin List*

Max-Planck-Institut für Kohlenforschung, Kaiser-Wilhelm-Platz 1, 45470 Mülheim an der Ruhr, Germany.

*E-mail: list@kofo.mpg.de

Table of Contents

General Information	S2
Synthesis of Exocyclic Enones	S3-S6
Synthesis of Catalysts	S6-S9
Reaction Optimization	S9-S10
General Procedure for the Catalytic Enantioselective Diels–Alder Reaction	S10
Characterization of the Spiro Carbocycle **7**	S11-S19
Resolution of (±)-**12**	S19-S21
Natural Product Synthesis	S21-S27
Absolute Configuration Determination	S27-S30
Computational Studies	S31-S123
References	S123-S124
Copies of NMR Spectra	S125-S184
HPLC and GC Traces	S185-S229

General Information

Unless otherwise stated, oven-dried (80 °C) or flame-dried glassware were used to conduct the experiments. All reactions were performed in anhydrous solvents under Ar atmosphere, applying standard Schlenk techniques. Dry argon was purchased from Air Liquide with >99.5% purity. Thin layer chromatography (TLC) on silica gel pre-coated glass plates (SIL G-25 UV254, 0.25 mm, (Macherey-Nagel) or plastic sheets (0.2 mm, MachereyNagel) were used to monitor the progress of the reactions and visualized by UV light at 254 nm, and/or anisaldehyde stain, and/or parmanganate stain. Preparative thin-layer chromatography was performed using SIL G-25 UV254 with 0.25 mm SiO_2 layer coated glass plates. Column chromatography was performed using Merck silica gel (60 Å, 230−400 mesh, particle size 0.040–0.063 mm). Technical grade solvents were used to elute the compounds during column chromatography. Elution was accelerated using compressed air. All isolated yields were reported, unless otherwise specified.

Chemicals were purchased from various commercial sources (Abcr, Acros Organics, Alfa Aesar, Fluorochem, Sigma-Aldrich, TCI) as reagent grade and used without further purification unless otherwise stated. Et_3N was distilled over $LiAlH_4$ and stored under argon prior to use. Previously reported compounds were synthesized from commercially available starting materials according to literature procedures.

Solvents ($CHCl_3$, CH_2Cl_2, Et_2O, DMSO, THF, toluene) were distilled using appropriate drying agent in the technical department of the Max-Planck-Institut für Kohlenforschung and store under Ar atmosphere, and used for the reaction.

Nuclear Magnetic Resonance Spectroscopy (NMR) spectra of ^{1}H, ^{13}C, ^{19}F, ^{31}P nucleus were recorded on a Bruker AV-600, AV-500 spectrometer in $CDCl_3$ or CD_2Cl_2. The resonance multiplicity is described as s (singlet), d (doublet), t (triplet), q (quadruplet), p (pentet), hept (heptet), m (multiplet), and br. (broad). Chemical shift δ in ppm and coupling constant *J* in Hz. The reported data were processed with Bruker TOPSPIN or MestReNova suits of programs. All spectra were recorded at 298 K unless otherwise noted. The residual deuterated solvent signal relative to tetramethylsilane (TMS) was used as the internal reference in ^{1}H NMR spectra ($CDCl_3$ δ 7.26, CD_2Cl_2 δ 5.32). ^{13}C NMR spectra reported in ppm from tetramethylsilane (TMS) with the solvent resonance as the internal standard ($CDCl_3$ δ 77.16, CD_2Cl_2 δ 53.8). ^{19}F, ^{31}P NMR spectra are reported relative to CCl_3F (δ (^{19}F) = 0 ppm), H_3PO_4 (δ (^{31}P) = 0 ppm) respectively.

High resolution mass spectrometry (HRMS) was performed on a Finnigan MAT 95 (EI) or Bruker APEX III FTMS (7T magnet, ESI). Electron impact (EI), mass spectrometry (MS) was performed on a Finnigan MAT 8200 (70 eV) or MAT 8400 (70 eV) spectrometer. The ionization method and mode of detection employed is indicated in the individual entry.

Enantiomeric ratio (e.r.) were determined by chiral either Gas Chromatography (GC) or High-Performance Liquid Chromatography (HPLC) analysis. All of the GC analyses were conducted in the GC department of the Max-Planck-Institut für Kohlenforschung using HP 6890 and 5890 series instruments (split-mode capillary injection system, flame ionization detector (FID), hydrogen carrier gas). The conditions employed are indicated in the individual experiments. HPLC analyses were performed on Shimadzu LC-20AB liquid chromatograph (reversed phase, SIL-20ACHT auto sampler, CTO-20AC column oven, SPD-M20A diode array detector) using Daicel columns with chiral stationary phases. The column employed and respective solvent mixture (HPLC-grade) are indicated for each experiment.

Specific rotations $[\alpha]_D^{25}$ were recorded by an Autopol IV polarimeter (Rudolph Research Analytical) at 25 °C with a sodium lamp (sodium D line, λ = 589 nm). Measurements were performed in an acid resistant 1 mL cell (50 mm length) with concentrations (*c*, g/100 mL) reported in $CHCl_3$.

Abbreviations

e.r. = enantiomeric ratio, d.r. = diastereomeric ratio, r.r. = regioisomeric ratio, r.t. = room temperature, TLC = thin layer chromatography, THF = tetrahydrofuran, MTBE = methyl tert-butyl ether, DEE = diethyl ether TFA = trifluoroacetic acid.

Synthesis of Exocyclic Enones

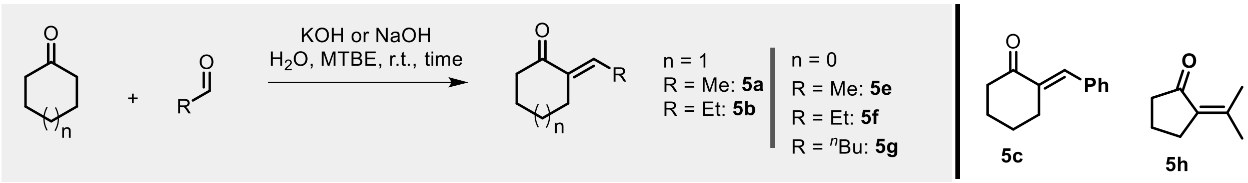

[**5a-b**, **5e** were prepared using literature reported procedure.[1] Compound **5c** was purchased from the commercial source and recrystallized using 5/95 CH_2Cl_2/pentane mixture. For the characterization of compound **5g** see ref.[5] Compound **5h** was prepared according to the literature report.[6]]

(*E*)-2-ethylidenecyclohexan-1-one (5a)

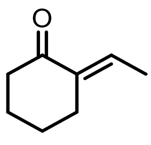

5a

The title compound **5a** was prepared according to the literature procedure[1], in 100 mmol scale, the desired product was obtained as colorless oil (1.6 g, 12%). **^{1}H NMR** (501 MHz, $CDCl_3$) δ 6.72 (m, 1H), 2.50–2.46 (m, 2H), 2.41 (t, *J* = 6.4 Hz, 2H), 1.87–1.82 (m, 2H), 1.76–1.72 (m, 5H); **^{13}C NMR** (126 MHz, $CDCl_3$) δ 201.1, 137.3, 134.4, 40.2, 26.5, 23.7, 23.5, 13.6; **HRMS** (ESI-pos) (m/z) calculated for $C_8H_{12}ONa$ [M + Na]$^+$: 147.078034, found: 147.078060.

(*E*)-2-propylidenecyclohexan-1-one (5b)

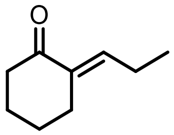

5b

The title compound **5b** was prepared following the literature procedure,[1] in 86 mmol scale. The product was obtained as colorless oil (1.22 g, 10%). **^{1}H NMR** (501 MHz, $CDCl_3$) δ 6.62–6.58 (m, 1H), 2.49–2.46 (m, 2H), 2.43–2.40 (m, 2H), 2.14–2.08 (m, 2H), 1.87–1.79 (m, 2H), 1.78–1.73 (m, 2H), 1.04 (td, *J* = 7.5, 1.3 Hz, 3H); **^{13}C NMR** (126 MHz, $CDCl_3$) δ 201.4, 141.2, 135.8, 40.3, 26.7, 23.7, 23.5, 21.2, 13.1; **HRMS** (GC-EI) (m/z) calculated for $C_9H_{14}O$ [M]$^+$: 138.103915, found: 138.104150. ^{1}H spectral data matches that reported in the literature.[2]

(*E*)-2-ethylidenecyclopentan-1-one (5e)

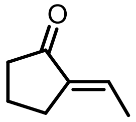

5e

The title compound **5e** was prepared according to the literature protocol,[1] in 100 mmol scales. The product was obtained as colorless oil (1.2 g, 11%). **^{1}H NMR** (501 MHz, $CDCl_3$) δ 6.64–6.58 (m, 1H), 2.61–2.56 (m, 2H), 2.33 (t, *J* = 7.9 Hz, 2H), 1.93 (p, *J* = 7.6 Hz, 2H), 1.80 (dt, *J* = 7.2, 1.9 Hz, 3H); **^{13}C NMR** (126 MHz, $CDCl_3$) δ 207.1, 138.5, 131.1, 38.8, 26.7, 19.9, 15.3; **HRMS** (GC-EI) (m/z) calculated for $C_7H_{10}O$ [M]$^+$: 110.072615, found: 110.072800. ^{1}H NMR spectral data matches that reported in the literature.[3]

Procedure for the synthesis of compound (5f) and (5g)

A mixture of aldehyde (69.3 mmol, 1.0 equiv.) and cyclopentanone (2 mL) was added dropwise to a vigorously stirred biphasic mixture of cyclopentanone (5 mL) and aqueous NaOH (40 mL) solution over a period of 1 h at 0°C. After complete addition, the mixture was stirred at room temperature for 2.5 h. Then the reaction mixture was diluted with Et_2O and extracted the organic layer. The organic layer was dried over anhydrous $MgSO_4$, filtered, and solvents were removed in *vacuo* to give the crude oil. The excess cyclic ketone was remove from the crude mixture *via* fractional distillation. Then the flash column chromatography was performed using 1-2% Et_2O in pentane mixture as eluent, and afforded the desired enone.

(*E*)-2-propylidenecyclopentan-1-one (5f)

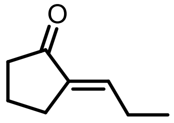

5f

Prepare in 69.3 mmol scale. The desired product was obtained as colorless oil (1.7 g, 19%). **^{1}H NMR** (501 MHz, $CDCl_3$) δ 6.52 (tt, *J* = 7.4, 2.7 Hz, 1H), 2.60-2.56 (m, 2H), 2.33 (t, *J* = 7.9 Hz, 2H), 2.15 (pt, *J* = 7.6, 1.6 Hz, 2H), 1.97–1.91 (m, 2H), 1.05 (t, *J* = 7.6 Hz, 3H); **^{13}C NMR** (126 MHz, $CDCl_3$) δ 207.8, 137.8, 136.7, 38.8, 26.7, 23.2, 19.9, 13.0; **HRMS** (GC-EI) (m/z) calculated for $C_8H_{12}O$ [M]$^+$: 124.088265, found: 124.088420. ^{1}H NMR spectral data matches that reported in the literature.[4]

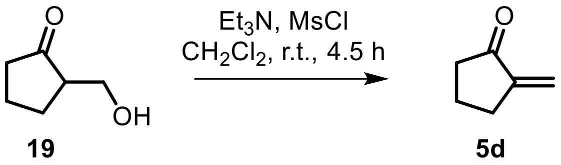

[Compound **19** was prepared following literature procedure.[7a]]

2-methylenecyclopentan-1-one (**5d**)
A solution of 2-hydroxymethylcyclopentanone **19** (1.5 g, 13.1 mmol, 1.0 equiv.) in CH_2Cl_2 (20 mL) under argon atmosphere was treated with Et_3N (7.32 mL, 52.6 mmol, 4.0 equiv.) at r.t. Afterwards MsCl (1.22 mL, 15.8 mmol, 1.2 equiv.) was added slowly to the solution over 20 minutes (process is exothermic, reaction temperature was control by using ice bath) and the mixture was stirred for additional 4 h at r.t. Then the reaction was treated with 10% HCl (10 mL), the phases were separated, and the aqueous phase was extracted further with CH_2Cl_2 (3 × 20 mL). The combined organic phases were further treated with aqueous saturated $NaHCO_3$ solution (20 mL). The organic layer was separated wash with brine water and dried over anhydrous Na_2SO_4, and the solvent was removed in rotary evaporator using low temperature (20 °C) water bath, under reduced pressure. The crude mixture was purified by flash column chromatography over silica gel, using 4-7 % (Et_2O/ n-pentane) as eluent and yielded the desired product as yellow oil (0.3 g, 24% yield). **^{1}H NMR** (501 MHz, CD_2Cl_2) δ 5.89 (td, *J* = 2.7, 1.3 Hz, 1H), 5.29 (td, *J* = 2.4, 1.3 Hz, 1H), 2.67 (tt, *J* = 7.3, 2.5 Hz, 2H), 2.30 (t, *J* = 7.8 Hz, 2H), 1.95–1.89 (m, 2H); **^{13}C NMR** (126 MHz, CD_2Cl_2) δ 207.1, 145.5, 116.3, 38.6, 30.1, 20.2; **HRMS** (GC-EI) (m/z) calculated for C_6H_8O $[M]^+$: 96.056965, found: 96.057090.

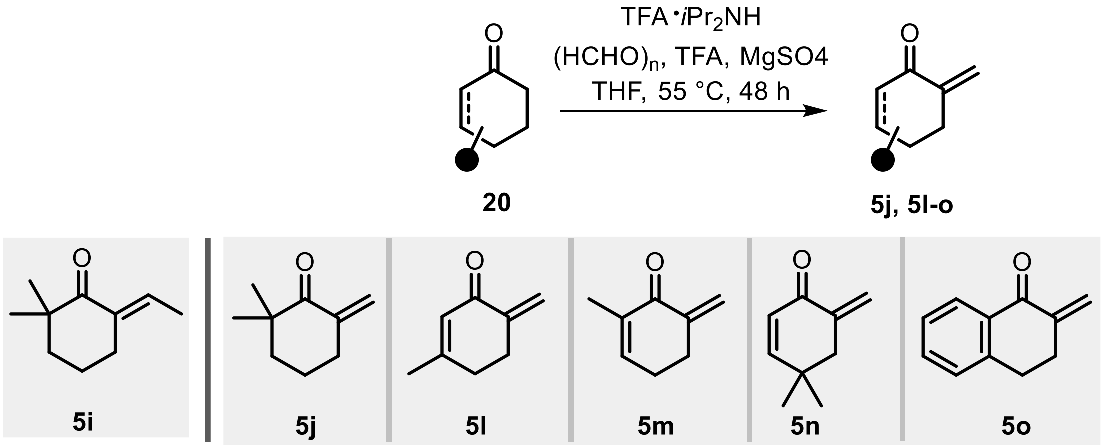

[Enone **5i** was prepared following literature reported protocol.[8a] Compound **5j**, **5l-o** were prepared according to literature reported procedure[8b]]

4,4-dimethyl-6-methylenecyclohex-2-en-1-one (5n)

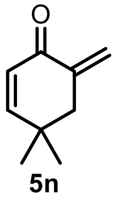

The title compound was prepared according to literature reported procedure[8b] using 4,4-dimethylcyclohex-2-en-1-one (1.06 mL, 8.05 mmol, 1.0 equiv.), TFA•^{i}Pr$_2$NH (3.47 g, 16.10 mmol, 2.0 equiv.), anhydrous $MgSO_4$ (0.969 g, 8.05 mmol, 1.0 equiv.), TFA (1.23 mL, 16.10 mmol, 2.0 equiv.), and $(HCHO)_n$ (1.93 g, 64.42 mmol, 8.0 equiv.). The crude product was purified by flash column chromatography using 2-3% Et_2O in pentane as eluent, afforded the desired product as colorless oil (0.626 g, 57%). **^{1}H NMR** (501 MHz, $CDCl_3$) δ 6.76 (d, *J* = 10.1 Hz, 1H), 6.03–6.02 (m, 1H), 5.97 (d, *J* = 10.0 Hz, 1H), 5.29 (m, 1H), 2.58 (br m, 2H), 1.15 (s, 6H); **^{13}C NMR** (126 MHz, $CDCl_3$) δ 188.7, 160.3, 141.4, 127.6, 121.4, 45.7, 35.0, 28.3; **HRMS** (GC-EI) (m/z) calculated for $C_9H_{12}O$ $[M]^+$: 136.088265, found: 136.088570. Spectral data matches that reported in literature.[8c]

2-methylene-3,4-dihydronaphthalen-1(2H)-one (5o)

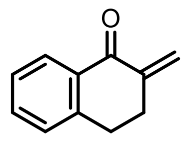

5o

The title compound was prepared according to the literature reported procedure[9b] using 3,4-dihydronaphthalen-1(2H)-one (0.91 mL, 6.84 mmol, 1.0 equiv.), TFA•^{i}Pr$_2$NH (2.95 g, 13.68 mmol, 2.0 equiv.), anhydrous $MgSO_4$ (0.823 g, 6.84 mmol, 1.0 equiv.), TFA (1.05 mL, 13.68 mmol, 2.0 equiv.), and $(HCHO)_n$ (1.64 g, 54.72 mmol, 8.0 equiv.) at 55°C for 40 h. The crude material was purified by a flash chromatography on silica gel using 4% ether in as eluent, afforded the desired exocyclic enone as yellow solid (0.84 g, 78%). **^{1}H NMR** (501 MHz, $CDCl_3$) δ 8.12 (dd, *J* = 7.9, 1.4 Hz, 1H), 7.49 (td, *J* = 7.5, 1.5 Hz, 1H), 7.37–7.32 (m, 1H), 7.26 (d, *J* = 8.0 Hz, 1H), 6.24–6.23 (m, 1H), 5.46–5.45 (m, 1H), 3.02–3.01 (m, 2H), 2.89–2.85 (m, 2H); **^{13}C NMR** (126 MHz, $CDCl_3$) δ 187.7, 144.3, 143.5, 133.5, 133.3, 128.6, 128.3, 127.2, 121.9, 31.9, 29.8; **HRMS** (GC-EI) (m/z) calculated for $C_{11}H_{10}O$ $[M]^+$: 158.072615, found: 158.072880. Spectral data matches that reported in literature.[7b]

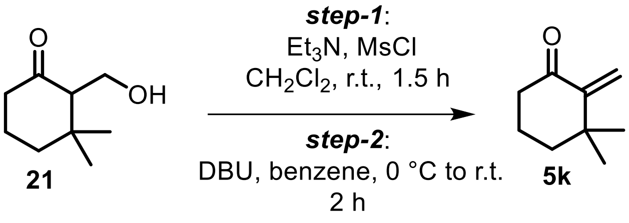

[Compound **21** was prepared according to literature reported procedure[9a]]

Synthesis of 3,3-dimethyl-2-methylenecyclohexan-1-one (**5k**)
Step-1
A flame-dried schlenk flask with a magnetic stirring bar was cooled under argon and charged with **21** (3.35 g, 21.44 mmol, 1 equiv.) in CH_2Cl_2 (60 mL). Then the solution was treated with Et_3N (11.96 mL, 85.77 mmol, 4.0 equiv.) at r.t. Afterwards MsCl (1.99 mL, 25.73 mmol, 1.2 equiv.) was added slowly to the solution over 20 minutes (process was exothermic, reaction temperature was control by using ice bath) and the mixture was stirred for additional 1 h. Then the reaction was treated with 10% HCl (30 mL), and diluted with CH_2Cl_2 (60 mL). The organic phase was separated and further washed with saturated $NaHCO_3$ (aq.) solution (50 mL). Then the organic layer was separated wash with brine water and dried over anhydrous Na_2SO_4, and the solvent was removed in rotary evaporator under reduced pressure. The crude material was directly used for the next step without further purification.
Step-2
To the solution of crude mesylate in dry benzene (40 mL) was added 1,8-diazabicyclo[5.4.0]undec-7-ene (DBU) (4.81 mL, 32.16 mmol, 1.5 equiv.) at 0 °C. Then the reaction mixture was slowly warmed to r.t. and was stirred for 2 h. Then the mixture was diluted with ice cold ether (100 mL) and treated with ice cooled 10% HCl (aq) solution (30 mL). The organic layer was separated out and washed with ice-cold $NaHCO_3$ (aq.) solution (100 mL), then with brine (50 mL). The combined organic layers were dried over anhydrous Na_2SO_4 and concentrated in vacuo using low temperature (20 °C) water bath. The crude compound was purified by a short plug silica gel column chromatography using (–80°C cold solution of 5-8% Et_2O in pentane mixture as eluent). The title compound was obtained as yellow oil (1.63 g, 55% yield over 2 steps). [N.B.: The enone is unstable at r.t. Hence it was synthesized and immediately used for the reaction]. **^{1}H NMR** (501 MHz, $CDCl_3$) δ 5.66 (d, *J* = 1.3 Hz, 1H), 5.16 (d, *J* = 1.3 Hz, 1H), 2.43 (t, *J* = 6.8 Hz, 2H), 1.94–1.88 (m, 2H), 1.67– 1.64 (m, 2H), 1.13 (s, 6H); **^{13}C NMR** (126 MHz, $CDCl_3$) δ 204.4, 155.9, 116.3, 40.9, 38.6, 38.0, 28.5, 19.9. Spectral data matches that reported in the literature.[9b]

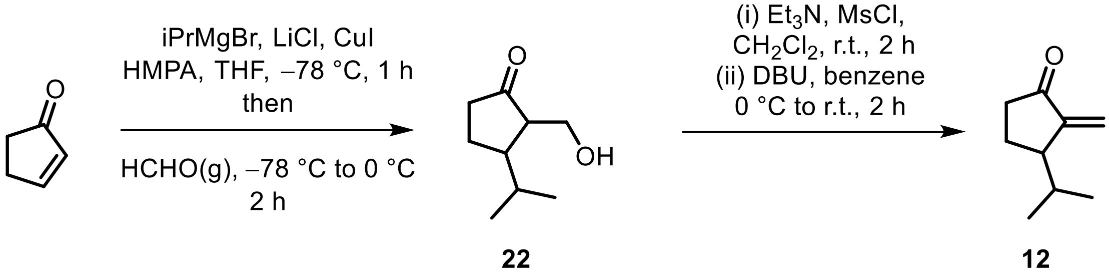

Synthesis of 3-isopropyl-2-methylenecyclopentan-1-one (**12**)
Step1: (**Synthesis of alcohol 22**)

A flame dried 250 mL 2-neck flask under argon was charged with CuI (231 mg, 1.22 mmol, 0.05 equiv.) and LiCl (103 mg, 2.44 mmol, 0.1 equiv.) in THF (50 mL) and vigorously stirred the mixture until a light-yellow homogenous solution resulted. Then HMPA (8.47 mL, 48.72 mmol, 2.0 equiv.) and cyclopentenone (2.04 mL, 24.36 mmol, 1.0 equiv.) were added sequentially at r.t. Afterwards the reaction vessel was cooled to –78 °C and isopropylmagnesium bromide (3 M in THF) (9.74 mL, 29.23 mmol, 1.2 equiv.) was slowly added into the CuI/LiCl/HMPA/enone solution over a period of 20 minutes and the strring was continued for 1 h (TLC showed full consumption of enone). Then the gaseous formaldehyde [generated by heating paraformaldehyde (2 g) to 150 °C] was bubbled into the solution by using a gas inlet. After 1 h the reaction mixture was slowly warmed to 0 °C and stirred for another 1 h. After that the reaction mixture was treated with saturated aqueous NH_4Cl (100 mL) solution. The resulting mixture was stirred at room temperature for 30 minutes and then diluted with EtOAc (100 mL). The organic layer was filter over celite and re-extracted with brine solution (100 mL). Then the collected organic layer was dried over anhydrous Na_2SO_4 before concentrating under reduced pressure. The crude product was purified on silica gel using 20-50% EtOAc in hexanes to afford the desired alcohol as a yellow oil (1.63 g, 42 % yield). (The isolated alcohol ca. 90% pure; it contained some unknown impurities).

Step 2:
The corresponding enone **12** was prepared using similar procedure as described for the synthesis of enone **5k** from **21**, starting with alcohol **22** in (1.2 g, 7.68 mmol, 1.0 equiv.) scale. The crude product was purified by flash column chromatography using 5-10 % Et_2O/pentane mixture as eluent, afforded the desired enone as colorless oil (0.39 g, 37%) . **^{1}H NMR** (501 MHz, $CDCl_3$) δ 6.09 (dd, *J* = 2.8, 1.1 Hz, 1H), 5.24 (dd, *J* = 2.5, 1.1 Hz, 1H), 2.71–2.66 (m, 1H), 2.40–2.34 (m, 1H), 2.30–2.23 (m, 1H), 2.01–1.93 (m, 2H), 1.74–1.66 (m, 1H), 1.00 (d, *J* = 6.8 Hz, 3H), 0.87 (d, *J* = 6.8 Hz, 3H); **^{13}C NMR** (126 MHz, $CDCl_3$) δ 208.0, 147.8, 117.8, 47.3, 37.2, 30.1, 21.4, 20.8, 17.8. Spectral data matches that reported in the literature[9c]

Synthesis of Catalysts

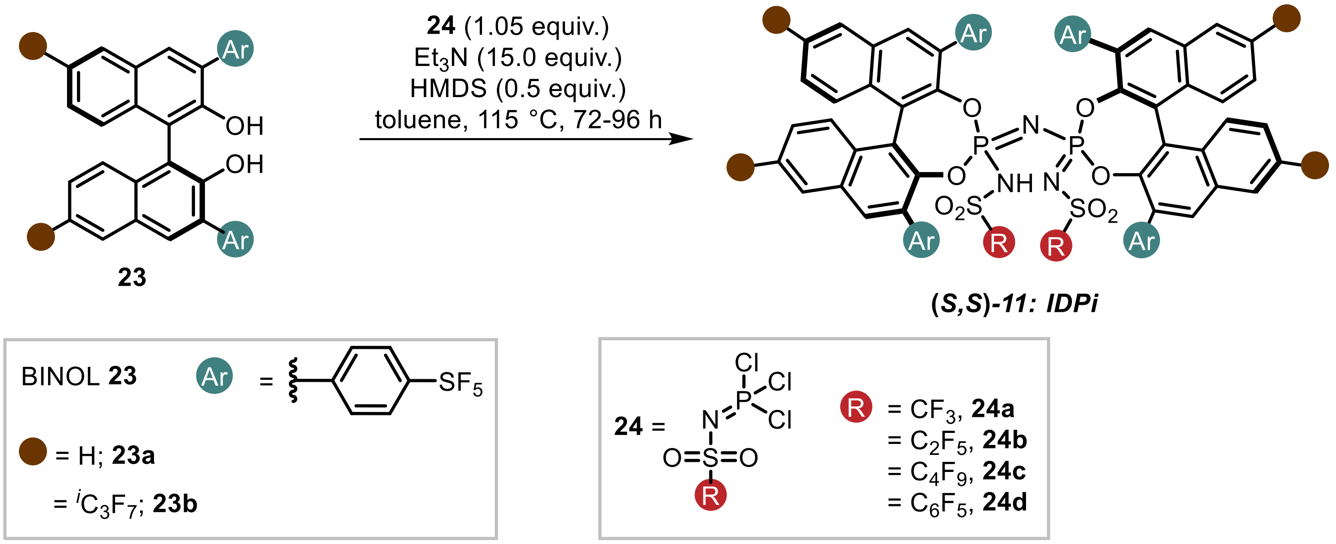

BINOL **23a** was synthesized according to the reported procedure[10a]
Phosphorimidoyl trichlorides **24a-d** were prepared according to the literature procedure[11]
Using known literature protocol Catalyst **8**,[10b] **9**,[10c] **10**,[10d] **11a**,[11c] **11c**,[11d] (**11d**, **11i**),[11a] **11j**[25] were synthesized.

(*S*)-3,3'-bis(4-(pentafluoro-l6-sulfanyl)phenyl)-6,6'-bis(perfluoropropan-2-yl)-[1,1'-binaphthalene]-2,2'-diol (23b)

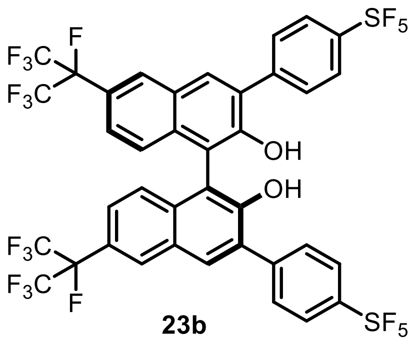

23b

The title BINOL **23b** was prepared according to literature reported procedure [10a] using corresponding Boronic ester (0.5 g, 0.52 mmol, 1.0 equiv.).[11e] The crude material was purified by flash column chromatography using 2-10% Et_2O in hexane as eluents and afforded the desired diol as colorless amorphous solid (0.475 g, 89%). **^{1}H NMR** (501 MHz, $CDCl_3$) δ 8.23 (d, *J* = 42.4 Hz, 4H), 7.91–7.83 (m, 8H), 7.56 (d, *J* = 9.0 Hz, 2H), 7.33 (d, *J* = 9.1 Hz, 2H), 5.44 (s, 2H); **^{13}C NMR** (126 MHz, $CDCl_3$) δ 153.8 (m), 151.9, 140.1, 134.1, 133.4, 130.5, 130.0, 128.7, 127.6 (d, *J* = 11.8 Hz), 126.4 (m), 125.1, 124.4 (d, *J* = 9.5 Hz), 123.6, 123.5, 122.0 (d, *J* = 28.6 Hz), 119.6 (d, *J* = 28.1 Hz), 111.6; **^{19}F NMR** (471 MHz, $CDCl_3$) δ 84.13 (p, *J* = 150.1Hz, 2F), 62.95 (d, *J* = 150.2 Hz, 8F), –75.38 (d, *J* = 14.3 Hz, 12F), –181.93 (s, 2F); **HRMS** (ESI-neg) (m/z) calculated for $C_{38}H_{17}O_2S_2F_{24}$ $[M-H]^-$: 1025.029234, found: 1025.029770; $[\alpha]_D^{25}$ = –49.490 (*c* 0.392, $CHCl_3$).

Imidodiphosphorimidate *(IDPi)* (11b)

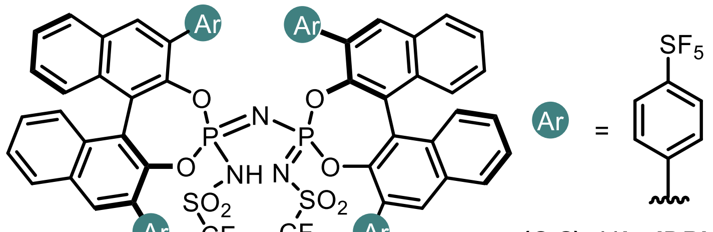

(S,S)-11b: IDPi

Brønsted acid catalyst **11b** was prepared according to known literature procedure[11b] using BINOL **23a** (100 mg, 0.145 mmol). The crude mixture was purified by column chromatography using 70-80% Et_2O in hexane mixture as eluent, afforded the brownish solid, which was subjected to acidification. The solid was dissolved in 10 mL of CH_2Cl_2 and 6.0 M HCl (aq) 10 mL was added to the solution, the resulting mixture was vigorously stirred at r.t. for 30 minutes. Afterwards the organic layer was collected and again washed with 6.0 M HCl (aq) (2 x 10 mL), then the organic layer was separated, concentrated, and dried under reduced pressure to afford the desired acid catalyst as a brownish amorphous solid (76 mg, 60% yield). **^{1}H NMR** (501 MHz, $CDCl_3$) δ 8.18 (d, *J* = 8.2 Hz, 2H), 8.08 (s, 2H), 8.05 (d, *J* = 8.3 Hz, 2H), 7.87 (ddd, *J* = 8.2, 6.5, 1.4 Hz, 2H), 7.74–7.68 (m, 8H), 7.62 (ddd, *J* = 8.2, 6.6, 1.4 Hz, 2H), 7.51 (s, 2H), 7.44–7.36 (m, 8H), 7.22–7.19 (m, 4H), 6.60 (d, *J* = 8.4 Hz, 4H); **^{13}C NMR** (151 MHz, $CDCl_3$) δ 153.5–152.8 (m), 143.3 (t, *J* = 5.1 Hz), 143.0 (t, *J* = 5.6 Hz), 139.5, 139.1, 132.7, 132.6, 132.1, 132.05, 132.0, 131.9, 131.5 (t, *J* = 1.9 Hz), 131.1, 130.3, 129.7, 129.2, 128.9, 128.3, 127.73, 127.68, 127.3, 127.0, 126.9, 126.0 (t, *J* = 4.3 Hz), 125.3 (t, *J* = 4.0 Hz), 123.9 (m), 122.2 (m). (other signals not detected or observed); **^{19}F NMR** (471 MHz, $CDCl_3$) δ 85.81–82.83 (m, 4F), 63.22 (d, *J* = 149.8 Hz, 8F), 62.72 (d, *J* = 149.5 Hz, 8F), –78.62 (s, 6F); **^{31}P NMR** (203 MHz, $CDCl_3$) δ –12.42; **HRMS** (ESI-neg) (m/z) calculated for $C_{66}H_{36}N_3O_8P_2S_6F_{26}$ $[M-H]^-$: 1745.989246, found: 1745.991270; $[\alpha]_D^{25}$ = +276.751 (*c* 0.179, $CHCl_3$).

Imidodiphosphorimidate *(IDPi)* (11e)

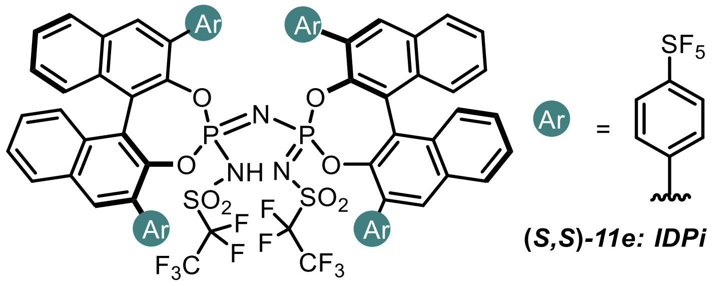

(S,S)-11e: IDPi

Brønsted acid catalyst **11e** was prepared according to known literature procedure[11b] using BINOL **23a** (734 mg, 1.063 mmol). The crude mixture was purified by column chromatography using 70-80% Et_2O in hexane mixture as eluent, afforded the brownish solid, which was subjected to acidification. The solid was dissolved in 30 mL of CH_2Cl_2 and 6.0 M HCl (aq) 30 mL was added to the solution, the resulting mixture was vigorously stirred at r.t. for 30 minutes. Afterwards the organic layer was collected and again washed with 6.0 M HCl (aq) (2 x 20 mL), then the organic layer was separated, concentrated, and dried under reduced pressure to afford the desired acid catalyst as a brownish amorphous solid in (220 mg, 33% yield). **^{1}H NMR** (501 MHz, $CDCl_3$) δ 8.15 (d, *J* = 8.2 Hz, 2H), 8.10 (s, 2H), 8.06 (d, *J* = 8.3 Hz, 2H), 7.89 (ddd, *J* = 8.2, 6.3, 1.6 Hz, 2H), 7.75–7.67 (m, 8H), 7.62 (ddd, *J* = 8.2, 6.7, 1.2 Hz, 2H), 7.41 (ddd, *J* = 8.1, 6.7, 1.3 Hz, 2H), 7.37–7.28 (m, 12H), 6.63 (d, *J* = 8.5 Hz, 4H); **^{13}C NMR** (151 MHz, $CDCl_3$) δ 153.2 (m), 143.5 (t, *J* = 5.1 Hz), 143.2 (t, *J* = 5.4 Hz), 139.7, 139.0, 132.7, 132.6, 132.09, 132.07, 131.9, 131.7, 131.5 (m), 131.0, 130.4, 129.8, 129.2, 129.0, 128.2, 127.6, 127.5, 127.2, 127.0, 126.0 (m), 125.2 (br), 123.9 (m), 122.1 (other

signals not detected or observed); ^{19}**F NMR** (471 MHz, $CDCl_3$) **δ** 84.41 (m, 4F), 63.22 (d, *J* = 149.9 Hz, 8F), 62.79 (d, *J* = 149.0 Hz, 8F), –79.14 (s, 6F), –116.54 (m, 4F); ^{31}**P NMR** (203 MHz, $CDCl_3$) δ –13.79; **HRMS** (ESI-neg) (m/z) calculated for $C_{68}H_{36}N_3O_8P_2S_6F_{30}$ [M-H]$^-$: 1845.982860, found: 1845.984350; **[α]$_D$^{25}** = +255.849 (*c* 0.265, $CHCl_3$).

Imidodiphosphorimidate *(IDPi)* (11f)

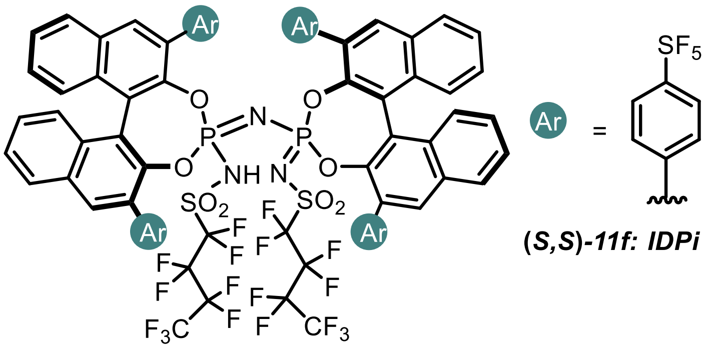

***(S,S)*-11f: IDPi**

Brønsted acid catalyst **11f** was prepared according to known literature procedure[11b] using BINOL **23a** (400 mg, 0.579 mmol). The crude mixture was purified by column chromatography using 70-80% Et_2O in hexane mixture as eluent, afforded the brownish solid, which was subjected to acidification. The solid was dissolved in 20 mL of CH_2Cl_2 and 6.0 M HCl (aq) 20 mL was added to the solution, the resulting mixture was vigorously stirred at r.t. for 30 minutes. Afterwards the organic layer was collected and again washed with 6.0 M HCl (aq) (2 x 20 mL), then the organic layer was separated, concentrated, and dried under reduced pressure to afford the desired acid catalyst as a brownish amorphous solid in (292 mg, 49% yield). ^{1}**H NMR** (501 MHz, $CDCl_3$) δ 8.14 (d, *J* = 8.3 Hz, 2H), 8.11 (s, 2H), 8.06 (d, *J* = 8.2 Hz, 2H), 7.89 (ddd, *J* = 8.2, 5.6, 2.4 Hz, 2H), 7.75–7.70 (m, 4H), 7.69–7.66 (m, 4H), 7.62 (ddd, *J* = 8.1, 6.7, 1.2 Hz, 2H), 7.41 (ddd, *J* = 8.2, 6.8, 1.3 Hz, 2H), 7.35 (dd, *J* = 8.7, 3.0 Hz, 6H), 7.31–7.28 (m, 6H), 6.65 (d, *J* = 8.4 Hz, 4H); ^{13}**C NMR** (151 MHz, $CDCl_3$) δ 153.2 (m), 143.5 (m), 143.1 (m), 139.3, 139.0, 132.7, 132.6, 132.1, 132.0, 131.7, 131.5, 131.0, 130.2, 129.8, 129.1, 129.0, 128.3, 127.6, 127.5, 127.2, 127.0, 126.9, 126.1, 125.2, 123.9, 122.1 (other signals not detected or observed); ^{19}**F NMR** (471 MHz, $CDCl_3$) δ 84.0 (m, 4F), 63.1 (d, *J* = 150.2 Hz, 8F), 62.5 (d, *J* = 149.4 Hz, 8F), –80.8 (t, *J* = 9.9 Hz, 6F), –111.85 (br, 4F), –121.1 (br, 4F), –126.13 (m, 4F); ^{31}**P NMR** (203 MHz, $CDCl_3$) δ –13.27; **HRMS** (ESI-neg) (m/z) calculated for $C_{72}H_{36}N_3O_8P_2S_6F_{38}$ [M-H]$^-$: 2045.970088, found: 2045.970210; **[α]$_D$^{25}** = +205.455 (*c* 0.22, $CHCl_3$).

Imidodiphosphorimidate *(IDPi)* (11g)

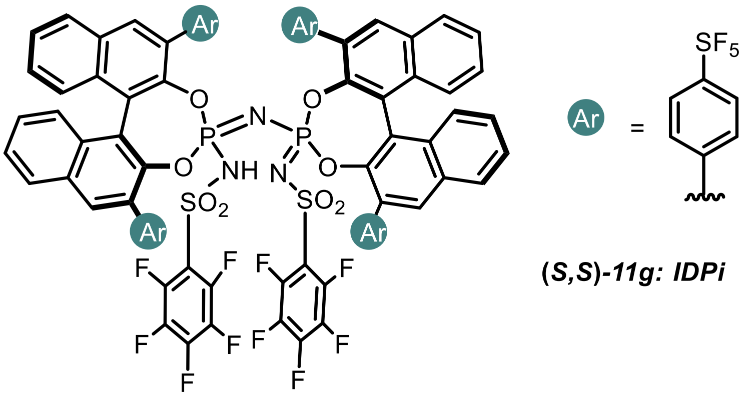

***(S,S)*-11g: IDPi**

Brønsted acid catalyst **11g** was prepared according to known literature procedure[11b] using BINOL **23a** (0.801 g, 1.16 mmol). The crude mixture was purified by column chromatography using 15-20% EtOAc in hexane as eluent, afforded the brownish solid, which was subjected to acidification. The solid was dissolved in 50 mL of CH_2Cl_2 and 6.0 M HCl (aq) 50 mL was added to the solution, the resulting mixture was vigorously stirred at r.t. for 30 minutes. Afterwards the organic layer was collected and again washed with 6.0 M HCl (aq) (2 x 30 mL), then the organic layer was separated, concentrated, and dried under reduced pressure to afford the desired acid catalyst as an off-white amorphous solid in (0.632 g, 56% yield). ^{1}**H NMR** (600 MHz, $CDCl_3$) δ 8.22 (d, *J* = 8.0 Hz, 2H), 8.06 (s, 2H), 8.02 (d, *J* = 8.3 Hz, 2H), 7.95 (s, 2H), 7.79 (ddd, *J* = 8.2, 6.8, 1.1 Hz, 2H), 7.72 (d, *J* = 8.5 Hz, 4H), 7.64–7.60 (m, 4H), 7.49 (d, *J* = 8.5 Hz, 4H), 7.43–7.40 (m, 4H), 7.33 (d, *J* = 8.5 Hz, 2H), 6.85 (s, 8H); ^{13}**C NMR** (151 MHz, $CDCl_3$) δ 153.4 (p, *J* = 17.3 Hz), 153.0 (p, *J* = 16.9 Hz), 143.9 (d, *J* = 263.0 Hz), 143.5 (d, *J* = 253.0 Hz), 143.1 (t, *J* = 5.1 Hz), 142.8 (t, *J* = 5.1 Hz), 139.1, 138.4, 137.2 (dt, *J* = 253.1, 12.2 Hz), 132.39, 132.38, 132.1, 132.0, 131.74, 131.69, 131.6, 131.5, 129.8, 129.7, 129.0, 128.8, 128.2, 127.9, 127.8, 127.5, 127.3, 126.5, 126.47, 125.3, 123.8, 122.4, 116.7 (m); ^{19}**F NMR** (565 MHz, $CDCl_3$) δ 84.4 (h, *J* = 149.6 Hz, 4F), 63.1 (d, *J* = 150.0 Hz, 8 F), 62.6 (d, *J* = 149.8 Hz, 8F), –135.6 (d, *J* = 22.4 Hz, 4F), –144.89 (s, 2 F), –159.64 (t, *J* = 20.5 Hz, 4F); ^{31}**P NMR** (203 MHz, $CDCl_3$) δ –5.44; **HRMS** (ESI-neg) (m/z) calculated for $C_{76}H_{35}F_{30}N_3O_8P_2S_6$ [M-H]$^-$: 1941.982860, found: 1941.984740; **[α]$_D$^{25}** = +153.160 (*c* 0.269, $CHCl_3$).

Imidodiphosphorimidate *(IDPi)* (11h)

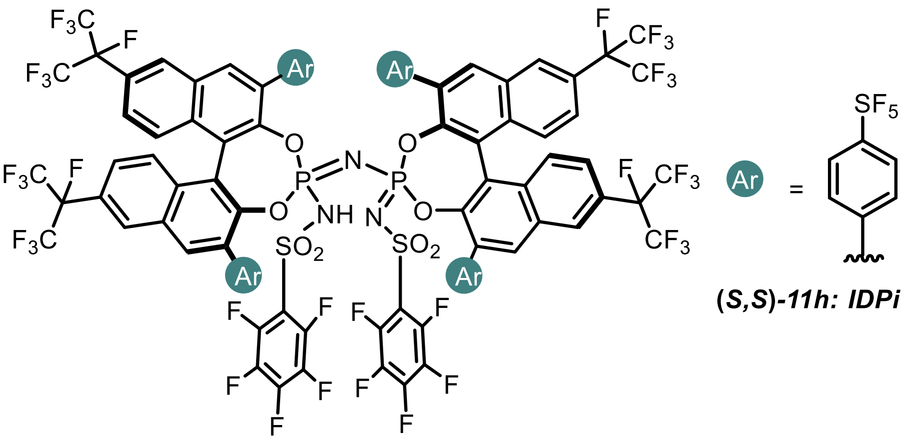

Brønsted acid catalyst **11h** was prepared according to known literature procedure[11b] using BINOL **23b** (0.19 g, 0.186 mmol). The crude mixture was purified by column chromatography using 15-18% EtOAc in hexane as eluent, afforded the brownish solid, which was subjected to acidification. The solid was dissolved in 15 mL of CH_2Cl_2 and 6.0 M HCl (aq) 15 mL was added to the solution, the resulting mixture was vigorously stirred at r.t. for 30 minutes. Afterwards the organic layer was collected and again washed with 6.0 M HCl (aq) (2 x 10 mL), then the organic layer was separated, concentrated, and dried under reduced pressure to afford the desired acid catalyst as an off-white amorphous solid in (0.127 g, 52% yield). **^{1}H NMR** (501 MHz, $CDCl_3$) δ 8.62 (br, 2H), 8.35 (br, 2H), 8.18 (s, 2H), 8.12 (s, 2H), 7.84 (d, *J* = 9.2 Hz, 2H), 7.74 (d, *J* = 8.6 Hz, 4H), 7.64 (d, *J* = 9.0 Hz, 2H), 7.49–7.44 (m, 8H), 6.89 (d, *J* = 8.4 Hz, 4H), 6.76–6.74 (m, 4H); **^{13}C NMR** (126 MHz, $CDCl_3$) δ 153.7 (m), 153.4(m), 144.9 (m), 144.2 (m), 143.0 (m), 138.1, 137.9, 136.1, 133.9, 133.4, 133.2, 133.0, 132.2, 132.1, 131.3, 130.8, 129.9, 129.57, 128.0, 127.8, 127.7, 127.6, 127.5, 127.1, 126.8, 126.7, 126.5, 126.4, 126.2, 125.2, 124.2, 123.5, 121.9, 121.7, 119.7, 119.5, 117.2 (other signals not detected or observed); **^{19}F NMR** (471 MHz, $CDCl_3$) δ 83.97 (m, 4F), 62.92 (d, *J* = 149.7 Hz, 8F), 62.51 (d, *J* = 149.9 Hz, 8F), –74.83 – –75.60 (m, 24F), –135.58 (S, 4F), –145.04 (s, 2F), –159.24 (s, 4F), –181.34 (m, 2F), –181.91 (m, 2F); **^{31}P NMR** (203 MHz, $CDCl_3$) δ –4.49; **HRMS** (ESI-neg) (m/z) calculated for $C_{88}H_{32}N_3O_8P_2S_6F_{58}$ $[M-H]^-$: 2613.906859, found: 2613.907200; **$[\alpha]_D^{25}$** = +127.692 (*c* 0.13, $CHCl_3$).

Reaction Optimization: Table 1

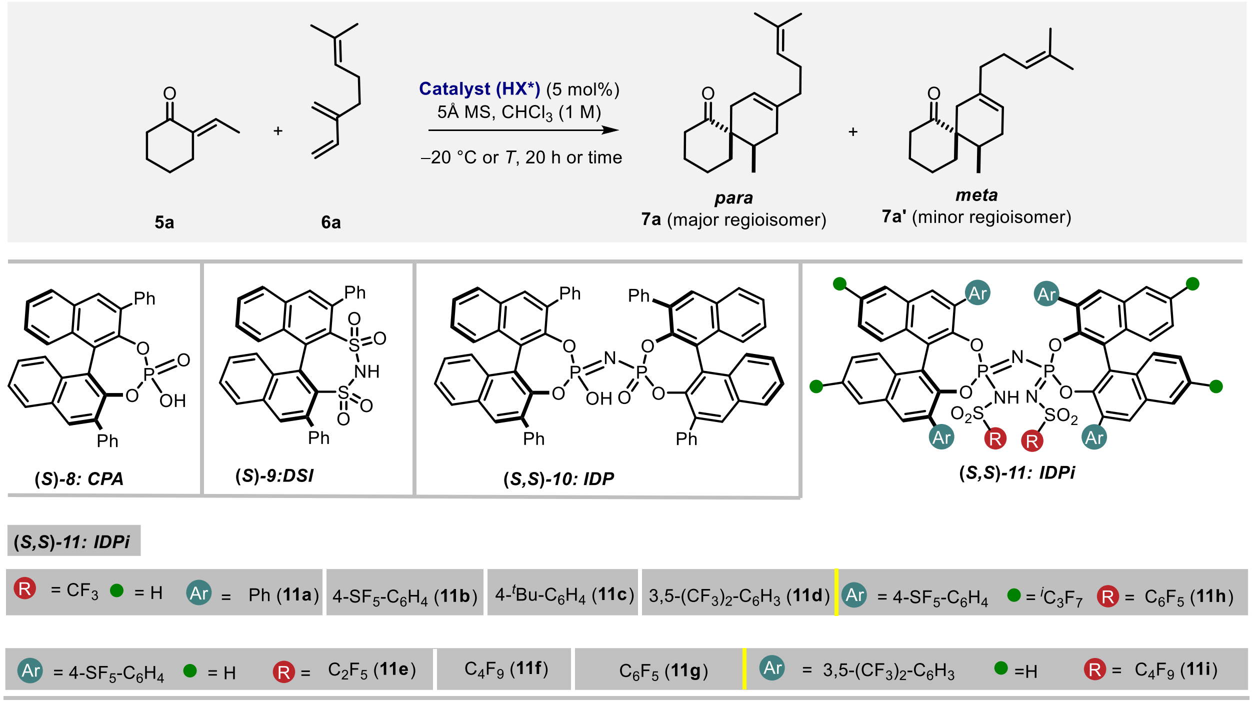

entry	*catalyst*	*temp.(°C)/ solvent*	*conv.(%)*	*r.r.*	*e.r.*[c]
1	**8**	–20 / $CHCl_3$	trace	–	–
2	**9**	–20 / $CHCl_3$	trace	–	–
3	**10**	–20 / $CHCl_3$	trace	–	–
4	**11a**	–20 / $CHCl_3$	67	>20:1	44:56
5	**11b**	–20 / $CHCl_3$	full	>20:1	64:36
6	**11c**	–20 / $CHCl_3$	9	–	–
7	**11d**	–20 / $CHCl_3$	full	>20:1	51:49
8	**11e**	–20 / $CHCl_3$	full	>20:1	69:31
9	**11f**	–20 / $CHCl_3$	full	>20:1	71:29
10	**11g**	–20 / **$CHCl_3$**	full	>20:1	93:7
11	**11g**	–20 / CH_2Cl_2	full	>20:1	89.5:10.5
12	**11g**	–20 / toluene	full	>20:1	89:11
13	**11g**	–20 / MeCy	full	>20:1	88.5:11.5
14	**11g**	–20 / MTBE	29	nd	86.5:13.5
15[d]	**11g**	–40 / $CHCl_3$	full	>20:1	95:5
16[e]	**11g**	–60 / $CHCl_3$	full	>20:1	96:4
17[e]	**11h**	–60 / $CHCl_3$	full	>20:1	95:5
18[e]	**11i**	–60 / $CHCl_3$	full	>20:1	58:42
19[e,f]	**11g**	–60 / $CHCl_3$	83	>20:1	95.5:4.5
20[e,g]	**11g**	–60 / $CHCl_3$	49	>20:1	95.5:4.5

[a]Performed with substrate **5a** (0.025 mmol) and diene **6a** (0.10 mmol, 4.0 equiv.), Conversions (conv.), and regioisomeric ratios (r.r.) were determined by ^{1}H NMR analysis with anisole or 1,4-dioxane as the internal standard. [b]*para*-regioisomer formed as the major product, confirmed by NMR analysis (see below) [c]Enantiomeric ratio (e.r.) for the major regioisomers, measured by GC or HPLC (see SI). [d]48 h. [e]72 h. [f]With diene **6a** (0.05 mmol., 2 equiv.), [g]With diene **6a** (0.025 mmol., 1.0 equiv.)

Synthesis of Racemates

Two methods were used for the synthesis of racemic spirocarbocycle **7** for the reference sample to determine the enantiomeric excess either by HPLC or GC analysis.
Method A: To a solution of enone (1 equiv., 0.5 mmol) in toluene (0.25 M) under argon was added anhy. $AlCl_3$ (5 mol%) at 0 °C. After wards diene (2.5 equiv. 1.25 mmol) was added and the reaction mixture was stirred for 2-3 h. Upon completion reaction mixture was diluted with Et_2O (10 mL) and 10% HCl (aq) (2 mL) was added and stir for 10 min. The organic layer was separated and washed with sat. $NaHCO_3$ (aq) (10 mL). Then the organic layer was separated, concentrated under reduce pressure, and purified by flash column chromatography using Et_2O-pentane mixture as eluent to afford the desired cycloaddition product.
Method B: A 2 mL flame-dried GC vial was charged with racemic catalyst (±)-**11d** (5-10 mol%) in $CHCl_3$ (1 M).To that solution was added enone (1.0 equiv., 0.5 mmol) and followed by diene (2.0 equiv. 1.07 mmol) and then the solution was cooled down to –20 °C and stirred for 1-2 d. Afterwards, the crude product was purified by flash column chromatography using Et_2O-pentane mixture as eluent, afforded the desired racemic spirocarbocycle.

General Procedure for the Catalytic Enantioselective Diels–Alder reaction of Exocyclic Enones (5)

A 2 mL flame-dried GC vial was charged with magnetic stir bar, catalyst (**11g** or mention in the separate entry) (5 mol%, 0.05 equiv., 0.01 mmol), 5Å MS (25 mg) and $CHCl_3$ (1 M, 200 μL). Afterwards the enone **5** (1 equiv., 0.2 mmol) was added to the reaction vial and was cooled to specified temperature (–40 °C, –60 °C or –80 °C). After 10 minutes at that temperature, diene (4 equiv., 0.8 mmol) was added through the wall of the reaction vial and stirred at the specified temperature for the specific reaction time. After specified time the reaction mixture was treated with Et_3N (15 μL), and strring was continued for an additional 10 minutes. Then the reaction mixture was warmed up to r.t. Purification was performed by column chromatography on silica gel using Et_2O/hexane or Et_2O/pentane mixture as eluents.

Characterization of the spiro carbocycle (7)

(6*R*,11*R*)-11-methyl-9-(4-methylpent-3-en-1-yl)spiro[5.5]undec-8-en-1-one (7a)

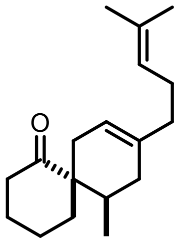

7a

The title compound was prepared according to the representative procedure at –60 °C for 3 d. The product was purified by column chromatography using 2–3% Et_2O in pentane as eluents and was obtained as colorless oil (94%, >20:1 r.r.). **^{1}H NMR** (501 MHz, $CDCl_3$ spectra contained >20:1 r.r.) δ 5.29–5.26 (m, 1H), 5.08–5.04 (m, 1H), 2.49–2.43 (m, 1H), 2.38–2.27 (m, 2H), 2.08–1.96 (m, 4H), 1.94–1.87 (m, 3H), 1.85–1.74 (m, 3H), 1.69–1.61 (m, 6H), 1.59–1.55 (m, 4H), 0.88 (d, *J* = 6.7 Hz, 3H); **^{13}C NMR** (126 MHz, $CDCl_3$) δ 215.9, 136.0, 131.5, 124.5, 117.9, 50.7, 39.1, 37.5, 33.7, 32.5, 31.2, 30.6, 26.9, 26.6, 25.9, 20.6, 17.9, 15.6; **HRMS** (GC-EI) (m/z) calculated for $C_{18}H_{28}O$ $[M]^+$: 260.213465, found: 260.213600; The enantiomeric ratio was determined by GC on a 25,0 m Hydrodey-gamma-TBDAc-CD chiral column, 0.60 bar H_2 gas pressure, t_R (minor) = 282.0 min., t_R (major) = 294.2 min., e.r. = 3:97; **$[\alpha]_D^{25}$** = –39.669 (*c* 0.303, $CHCl_3$).

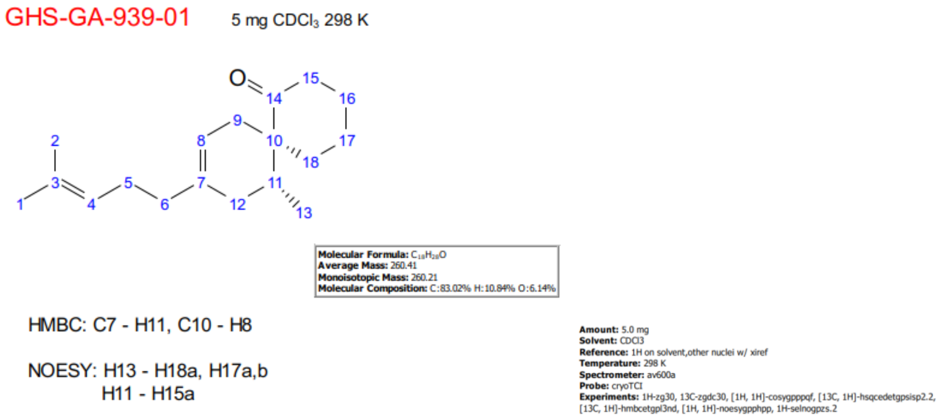

Atom	δ (ppm)	J	COSY	HSQC	HMBC	NOESY
1 C	25.69			1	2, 4	
H3	1.67	1.30(?)	2, 4, 5	1	2, 3, 4, 6	4
2 C	17.69			2	1, 4	
H3	1.59		1, 4, 5	2	1, 3, 4	5
3 C	131.31				1, 2, 5	
4 C	124.30			4	1, 2, 5, 6	
H	5.06	7.00(?), 2.90(?), 1.40(?)	1, 2, 5	4	1, 2, 5, 6	1
5 C	26.38			5	4, 6	
H2	2.04		1, 2, 4, 6	5	3, 4, 6, 7	2, 8
6 C	37.32			6	1, 4, 5, 8, 12a, 12b	
H2	1.92		5	6	4, 5, 7, 8, 12	8
7 C	135.83				5, 6, 9a, 9b, 11, 12a, 12b	
8 C	117.71			8	6, 9a, 9b, 12a, 12b	
H	5.27		9a, 9b, 12a, 12b	8	6, 9, 10, 12	5, 6, 9a, 9b
9 C	32.35			9a, 9b	8, 11, 18a, 18b	
Ha	2.06		8, 9b	9	7, 8, 10, 18	8, 11
Hb	1.99		8, 9a	9	7, 8, 10, 11, 14, 18	8, 13, 15b
10 C	50.57				8, 9a, 9b, 11, 12a, 13, 15a, 17a, 17b, 18a, 18b	
11 C	31.02			11	9b, 12a, 12b, 13, 18a, 18b	
H	2.34	13.70(?), 6.80(?), 5.30(?)	12a, 12b, 13	11	7, 9, 10, 12, 13, 18	9a, 12a, 12b, 13, 15a, 17a
12 C	33.51			12a, 12b	6, 8, 11, 13	
Ha	1.89		8, 11, 12b	12	6, 7, 8, 10, 11, 13	11, 13, 15a
Hb	1.62		8, 11, 12a	12	6, 7, 8, 11, 13	11, 13
13 C	15.43			13	11, 12a, 12b	
H3	0.88	6.80(?)	11	13	10, 11, 12	9b, 11, 12a, 12b, 17a, 17b, 18a
14 C	215.74				9b, 15a, 15b, 16a, 16b, 18a, 18b	
15 C	38.93			15a, 15b	16a, 16b, 17a, 17b	
Ha	2.46	14.70(?), 7.20(?), 5.40(?), 1.20(?)	15b, 16a, 16b	15	10, 14, 16, 17	11, 12a, 15b, 16a, 16b, 17a
Hb	2.30	14.60(?), 8.60(?), 5.60(?)	15a, 16a, 16b	15	14, 16, 17	9b, 15a, 16a, 16b
16 C	26.77			16a, 16b	15a, 15b, 17a, 17b, 18a, 18b	
Ha	1.82		15a, 15b, 16b, 17a, 17b	16	14, 15, 17, 18	15a, 15b
Hb	1.79		15a, 15b, 16a, 17a, 17b	16	14, 15, 17, 18	15a, 15b
17 C	20.47			17a, 17b	15a, 15b, 16a, 16b, 18a, 18b	
Ha	1.76		16a, 16b, 17b, 18a, 18b	17	10, 15, 16, 18	11, 13, 15a
Hb	1.66		16a, 16b, 17a, 18a, 18b	17	10, 15, 16, 18	13
18 C	30.43			18a, 18b	9a, 9b, 11, 16a, 16b, 17a, 17b	
Ha	1.66		17a, 17b, 18b	18	9, 10, 11, 14, 16, 17	13
Hb	1.57		17a, 17b, 18a	18	9, 10, 11, 14, 16, 17	

Table 2. Peak table for **7a**, COSY, HSQC, HMBC, NOESY signals for the assignment of relative stereochemistry.

(6*R*,11*R*)-11-ethyl-9-(4-methylpent-3-en-1-yl)spiro[5.5]undec-8-en-1-one (7b)

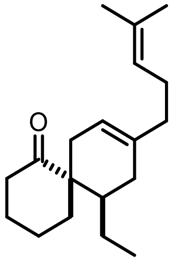

7b

The title compound was prepared according to the representative procedure at –60 °C for 5 d. The product was purified by column chromatography using 2-3% Et_2O in pentane as eluents and was obtained as colorless oil (71%, >20:1 r.r.). **^{1}H NMR** (501 MHz, $CDCl_3$, spectra contained >20:1 r.r.) δ 5.28–5.26 (m, 1H), 5.09–5.05 (m, 1H), 2.49–2.43 (dddd, *J* = 14.8, 6.7, 5.2, 1.2 Hz, 1H), 2.29 (ddd, *J* = 14.8, 8.9, 5.7 Hz, 1H), 2.08–1.91 (m, 8H), 1.86–1.72 (m, 3H), 1.70–1.67 (m, 4H), 1.66–1.60 (m, 3H), 1.60 (s, 3H), 1.48–1.41 (m, 1H), 1.06–0.97 (m, 1H), 0.94 (t, *J* = 7.1 Hz, 3H); **^{13}C NMR** (126 MHz, $CDCl_3$) δ 216.1, 136.0, 131.5, 124.5, 118.0, 51.4, 39.2, 38.6, 37.6, 33.0, 30.4, 29.8, 26.7, 26.6, 25.9, 22.8, 20.7, 17.9, 12.8; **HRMS** (GC-EI) (m/z) calculated for $C_{19}H_{30}O$ $[M]^+$: 274.229115, found: 274.229270. The enantiomeric ratio was determined by HPLC on a chiral stationary phase; HPLC column: IA-3R, solvent system: CH_3CN/H_2O = 70/30, flow rate: 1 mL/min, temp.: 25 °C, 220 nm, t_R (minor) = 9.0 min., t_R (major) = 11.9 min., e.r. = 9.5:90.5; **$[\alpha]_D^{25}$** = –49.286 (*c* 0.28, $CHCl_3$).

(6*R*,11*S*)-9-(4-methylpent-3-en-1-yl)-11-phenylspiro[5.5]undec-8-en-1-one (**7c**)

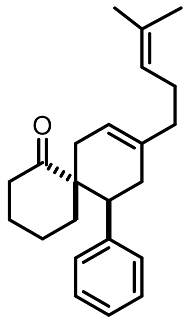

7c

The title compound was prepared according to the representative procedure at –60 °C for 8 d using **catalyst 11h** (10 mol%). The product was purified by column chromatography using 2-4% Et_2O in pentane as eluents and was obtained as colorless oil (45%, >20:1 r.r.). **^{1}H NMR** (501 MHz, $CDCl_3$, spectra contained >20:1 r.r.) δ 7.26 (br, 2 H), 7.25 (br, 2H), 7.22–7.18 (m, 1H), 5.48 (m, 1H), 5.11–5.07 (m, 1H), 3.41–3.39 (m, 1H), 2.49–2.39 (m, 3H), 2.30–2.25 (m, 1H), 2.19–2.14 (m, 1H), 2.12–2.05 (m, 2H), 2.04–1.93 (m, 3H), 1.87–1.80 (m, 1H), 1.77–1.71 (m, 2H), 1.68 (d, *J* = 1.5 Hz, 3H), 1.63–1.52 (m, 5H), 1.43–1.38 (m, 1H); **^{13}C NMR** (126 MHz, $CDCl_3$) δ 215.6, 143.3, 137.7, 131.6, 129.4, 128.2, 126.6, 124.4, 118.7, 51.5, 43.7, 38.8, 37.5, 35.4, 32.8, 31.6, 27.5, 26.5, 25.9, 20.8, 17.9; **HRMS** (GC-EI) (m/z) calculated for $C_{23}H_{30}O$ $[M]^+$: 322.229115, found: 322.228840; The enantiomeric ratio was determined by HPLC on a chiral stationary phase; HPLC column: AD-3R, solvent system: CH_3CN/H_2O = 60/40, flow rate: 1 mL/min, temp.: 25 °C, 220 nm, t_R (minor) = 21.8 min., t_R (major) = 25.8 min., e.r. = 8:92; **$[\alpha]_D^{25}$** = –44.749 (*c* 0.22, $CHCl_3$).
Reaction in the presence of catalyst **11g,** gave 22% conv., in 12 d at –50 °C, 88.5:11.5 e.r

(*S*)-8-(4-methylpent-3-en-1-yl)spiro[4.5]dec-7-en-1-one (**7d**)

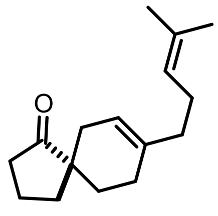

7d

The title compound was prepared according to the representative procedure at –80 °C for 3 d. The product was purified by column chromatography using 2-4% Et_2O in pentane as eluents and was obtained as colorless oil (90%, 16:1 r.r.). **^{1}H NMR** (501 MHz, $CDCl_3$, spectra contained 16:1 r.r.) δ 5.37–5.35 (m, 1H), 5.10–5.07 (m, 1H), 2.31–2.28 (m, 2H), 2.15 (dp, *J* = 17.1, 2.6 Hz, 1H), 2.08 (q, *J* = 7.4 Hz, 2H), 2.01–1.95 (m, 4H), 1.94 – 1.83 (m, 2H), 1.83–1.74 (m, 3H), 1.68 (d, *J* = 1.5 Hz, 3H), 1.67–1.62 (m, 1H), 1.60 (d, *J* = 1.4 Hz, 3H), 1.46-1.41 (m, 1H); **^{13}C NMR** (126 MHz, $CDCl_3$) δ 224.0, 136.9, 131.6, 124.3, 118.6, 47.8, 38.0, 37.6, 34.0, 32.5, 28.6, 26.6, 25.9, 25.3, 19.0, 17.8; **HRMS** (ESIpos) (m/z) calculated for $C_{16}H_{24}ONa$ $[M+Na]^+$: 255.171934, found: 255.171720. The enantiomeric ratio was determined by HPLC on a chiral stationary phase; HPLC column: AD-3R, solvent system: CH_3CN/H_2O = 60/40, flow rate: 1 mL/min, temp.: 25 °C, 220 nm, t_R (minor) = 16.5 min., t_R (major) = 17.9 min., e.r. = 3:97; **$[\alpha]_D^{25}$** = –32.558 (*c* 0.258, $CHCl_3$).

(5*R*,10*R*)-10-methyl-8-(4-methylpent-3-en-1-yl)spiro[4.5]dec-7-en-1-one (**7e**)

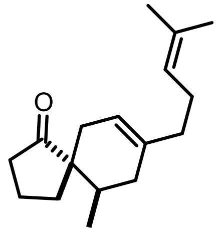

7e

The title compound was prepared according to the representative procedure at –50 °C for 5.5 d. The product was purified by column chromatography using 2-4% Et_2O in pentane as eluents and was obtained as colorless oil (89%, >20:1 r.r.). **^{1}H NMR** (501 MHz, $CDCl_3$, spectra contained >20:1 r.r.) δ 5.30–5.29 (m, 1H), 5.08–5.04 (m, 1H), 2.39– 2.33 (m, 1H), 2.15–1.85 (m, 10H), 1.84–1.75 (m, 2H), 1.72–1.62 (m, 5H), 1.60 (br, 3H), 0.76 (d, *J* = 6.6 Hz, 3H); **^{13}C NMR** (126 MHz, $CDCl_3$) δ 225.4, 137.9, 131.7, 124.2, 118.1, 52.0, 40.1, 37.4, 34.7, 34.6, 32.7, 27.3, 26.4, 25.9, 19.1, 17.9, 16.4; **HRMS** (GC-EI) (m/z) calculated for $C_{17}H_{26}O$ $[M]^+$: 246.197815, found: 246.197970; The enantiomeric ratio was determined by HPLC on a chiral stationary phase; HPLC column: AD-3R, solvent system: CH_3CN/H_2O = 60/40, flow rate: 1.2 mL/min., temp.: 25 °C, 220 nm, t_R (minor) = 14.7 min., t_R (major) = 18.2 min., e.r. = 1:99; **$[\alpha]_D^{25}$** = –76.761 (*c* 0.284, $CHCl_3$).

(5*R*,10*R*)-10-ethyl-8-(4-methylpent-3-en-1-yl)spiro[4.5]dec-7-en-1-one (**7f**)

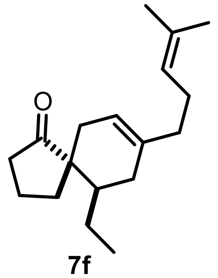

7f

The title compound was prepared according to the representative procedure at –45 °C for 5 d. The product was purified by column chromatography using 2-4% Et_2O in pentane as eluents and was obtained as colorless oil (44%, > 20:1 r.r.). **^{1}H NMR** (501 MHz, $CDCl_3$, spectra contained > 20:1 r.r.) δ 5.31–5.29 (m, 1H), 5.10–5.06 (m, 1H), 2.38–2.32 (m, 1H), 2.18–1.70 (m, 13H), 1.69 (d, *J* = 1.4 Hz, 3H), 1.61 (d, *J* = 1.3 Hz, 3H), 1.57–1.51 (m, 1H), 1.26–1.17 (m, 1H), 1.09–1.00 (m, 1H), 0.88 (t, *J* = 7.4 Hz, 3H); **^{13}C NMR** (126 MHz, $CDCl_3$) δ 225.4, 137.7, 131.6, 124.3, 118.1, 52.5, 40.3, 40.1, 37.5, 35.0, 31.5, 28.4, 26.6, 25.9, 24.5, 19.2, 17.9, 12.2; **HRMS** (GC-EI) (m/z) calculated for $C_{18}H_{28}O$ $[M]^+$: 260.213465, found: 260.213510; The enantiomeric ratio was determined by HPLC on a chiral stationary phase; HPLC column: IA-3R, solvent system: CH_3CN/H_2O = 70/30, flow rate: 1.0 mL/min., temp.: 25 °C, 220 nm, t_R (minor) = 7.5 min., t_R (major) = 9.4 min., e.r. = 5.5:94.5; **$[\alpha]_D^{25}$** = –85.246 (*c* 0.183, $CHCl_3$).

(5*R*,10*R*)-10-butyl-8-(4-methylpent-3-en-1-yl)spiro[4.5]dec-7-en-1-one (**7g**)

The title compound was prepared according to the representative procedure in 0.2 mmol scale at –20 °C for 4 d. The product was purified by column chromatography using 2-4% Et_2O in pentane as eluents and was obtained as colorless

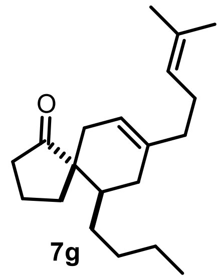

7g

oil (49%, > 20:1 r.r.). **^{1}H NMR** (501 MHz, $CDCl_3$, spectra contained > 20:1 r.r.) δ 5.31–5.29 (m, 1H), 5.08 (tp, J = 6.7, 1.5 Hz, 1H), 2.39–2.33 (m, 1H), 2.16–1.70 (m, 13H), 1.68 (d, J = 1.4 Hz, 3H), 1.61 (d, J = 1.2 Hz, 3H), 1.59–1.56 (m, 1H), 1.37–1.25 (m, 2H), 1.24–1.13 (m, 2H), 1.12–1.01 (m, 2H), 0.86 (t, J = 7.0 Hz, 3H); **^{13}C NMR** (126 MHz, $CDCl_3$) δ 225.7, 137.7, 131.7, 124.3, 118.0, 52.4, 40.4, 38.0, 37.4, 34.8, 32.0, 31.4, 29.7, 28.2, 26.5, 25.9, 23.0, 19.2, 17.9, 14.3; **HRMS** (GC-EI) (m/z) calculated for $C_{20}H_{32}O$ $[M]^+$: 288.244765, found: 288.244900; The enantiomeric ratio was determined by HPLC on a chiral stationary phase; HPLC column: AD-3R, solvent system: CH_3CN/H_2O = 70/30, flow rate: 1.0 mL/min., temp.: 25 °C, 220 nm, t_R (minor) = 8.9 min., t_R (major) = 12.4 min., e.r. = 9:91; **$[\alpha]_D^{25}$** = –79.322 (c 0.295, $CHCl_3$).

(*R*)-10,10-dimethyl-8-(4-methylpent-3-en-1-yl)spiro[4.5]dec-7-en-1-one (7h)

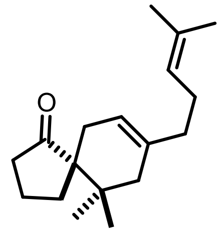

7h

The title compound was prepared according to the representative procedure in 0.2 mmol scale and in the presence of catalyst **11i** (10 mol%), in $CHCl_3$/pentane (1:5) mixture (1 M) as solvent, at –45 °C for 7 d. The product was purified by column chromatography using 2-4% Et_2O in pentane as eluents and was obtained as colorless oil (33%, > 20:1 r.r.). **^{1}H NMR** (501 MHz, $CDCl_3$, spectra contained > 20:1 r.r.) δ 5.28–5.26 (m, 1H), 5.10–5.08 (m, 1H), 2.34–2.28 (m, 1H), 2.21–2.12 (m, 2H), 2.11–2.02 (m, 3H), 1.96 (t, J = 7.7 Hz, 2H), 1.93–1.70 (m, 6H), 1.68 (d, J = 1.3 Hz, 3H), 1.61 (brs, 3H), 1.02 (s, 3H), 0.85 (s, 3H); **^{13}C NMR** (126 MHz, $CDCl_3$) δ 222.9, 137.9, 131.5, 124.5, 117.3, 52.4, 41.9, 40.7, 37.7, 34.0, 32.2, 32.2, 26.6, 25.9, 25.2, 23.6, 18.8, 17.9; **HRMS** (GC-EI) (m/z) calculated for $C_{18}H_{28}O$ $[M]^+$: 260.213465, found: 260.213650; The enantiomeric ratio was determined by HPLC on a chiral stationary phase; HPLC column: AD-3R, solvent system: CH_3CN/H_2O = 60/40, flow rate: 1.0 mL/min., temp.: 25 °C, 220 nm, t_R (minor) = 16.5 min., t_R (major) = 26.4 min., e.r. = 97.5:2.5; **$[\alpha]_D^{25}$** = –94.444 (c 0.144, $CHCl_3$).
Reaction in the presence of catalyst **11g** provided trace of conversion at –50 °C, in $CHCl_3$.

(6*R*,11*R*)-2,2,11-trimethyl-9-(4-methylpent-3-en-1-yl)spiro[5.5]undec-8-en-1-one (7i) and (6*R*,11*R*)-2,2,11-trimethyl-8-(4-methylpent-3-en-1-yl)spiro[5.5]undec-8-en-1-one (7i')

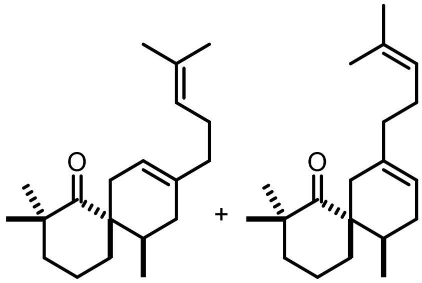

7i **7i'**

The title compounds were prepared according to the representative procedure in 0.2 mmol scale at –60 °C for 8 d. The product was purified by column chromatography using 1-1.5% Et_2O in pentane as eluents and was obtained as colorless oil (69%, 5:1 r.r.). **^{1}H NMR** (600 MHz, $CDCl_3$, spectra contained 5:1 r.r.) δ 5.37 (m, 1H for minor regioisomer), 5.22–5.21 (m, 1H for major regioisomer), 5.09–5.05 (m, 1H for major regioisomer +1H for minor regioisomer), 2.36–2.30 (m, 1H for major regioisomer), 2.29–2.26 (m, 1H for minor regioisomer), 2.26–2.21 (m, 1H for major regioisomer), 2.11–2.03 (m, 2H for major regioisomer + 3H for minor regioisomer), 1.98–1.91 (m, 3H for major regioisomer + 3H for minor regioisomer), 1.86–1.75 (m, 2H for major regioisomer + 2H for minor regioisomer), 1.70–1.61 (m, 6H for major regioisomer + 6H for minor regioisomer), 1.61–1.59 (m, 3H for major regioisomer + 3H for minor regioisomer), 1.59–1.57 (m, 1H for major regioisomer + 1H for minor regioisomer), 1.56–1.49 (m, 2H for major regioisomer + 2H for minor regioisomer), 1.12 (s, 3H for minor regioisomer), 1.10 (s, 3H for major regioisomer), 1.08 (s, 3H for minor regioisomer), 1.07 (s, 3H for major regioisomer), 0.73 (d, J = 6.6 Hz, 3H for major regioisomer) 0.72 (d, J = 6.6 Hz, 3H for minor regioisomer); **^{13}C NMR** for major regioisomer (151 MHz, $CDCl_3$) δ 222.3, 137.4, 131.6, 124.4, 118.0, , 50.4, 44.6, 39.0, 37.4, 37.1, 33.6, 33.3, 27.8, 27.3, 26.5, 25.9, 25.5, 17.9, 17.5, 17.0; **^{13}C NMR** for minor regioisomer (151 MHz, $CDCl_3$) δ 222.2, 135.0, 131.6, 124.3, 120.5, 50.8, 44.6, 40.0, 39.0, 37.7, 33.0, 30.6, 27.9, 26.7, 25.9, 25.8, 18.0, 16.7 (other peaks are not identified); **HRMS** (ESI pos.) (m/z) calculated for $C_{20}H_{33}O$ $[M+H]^+$: 289.252590, found: 289.252520; The enantiomeric ratio was determined by HPLC on a chiral stationary phase; HPLC column: AD-3R, solvent system: CH_3CN/H_2O = 70/30, flow rate: 1.0 mL/min., temp.: 25 °C, 220 nm, t_R (major for the major regioisomer) = 11.9 min., t_R (minor for the major regioisomer) = 16.2 min., e.r. = 91:9.

User Report
GHS-GB-209-01

The sample is a ~ 5:1 mixture of the following regioisomers:

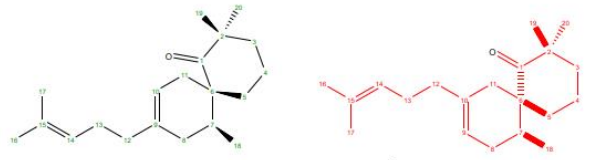

major

minor

Remarks:

The 2 different regioisomers could be desigushed with the the help of the 2D NOESY and HMBC data.
For the major regisomer an NOE of H10 with H11'/H11" and H12 is observed. Additionally H9 has a HMBC cross peak to C6.
The minor isomer shows an NOE to H8'/H8" and H12.

Major differences in the structure are found at C11,C10,C9 and C9, which supports the presence of different regioisimers rather than diastereomers.

Futhermore, the chemical shifts of the second ring are very similar, is can be assumed, that both compound have the same relative stereochemistry at C6 and C7.

The relative sterochemistry was determinded from the 2D NOESY. Relevant NOE cross peaks are shown in the Chem3D model on the left.

All extractable assignment are concluded in the assignment tables on the next page.

Chem3D models from different angels
with important NOEs for the major regiosomer

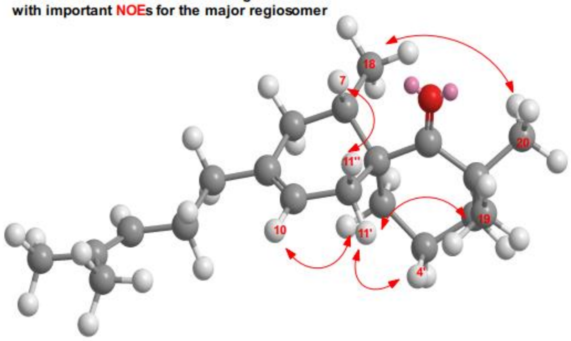

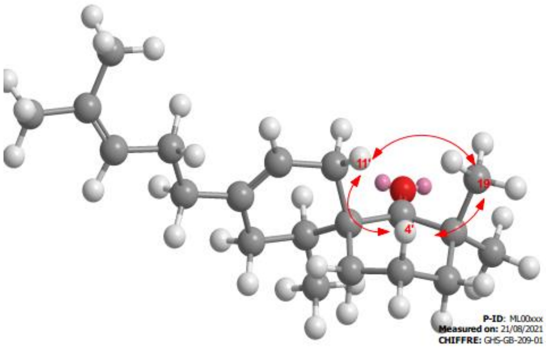

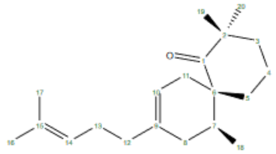

Atom	δ (ppm)	J	COSY	HSQC	HMBC	NOESY
1 C	222.257				7, 19, 20	
2 C	44.620				19, 20	
3 C	38.973			3', 3''	5', 19, 20	
H'	1.691		3'', 4', 4''	3		19, 20
H''	1.575		3'	3	19, 20	20
4 C	17.464			4', 4''	5'	
H'	1.793		3', 4'', 5	4		11', 19
H''	1.623		3', 4', 5	4		
5 C	25.514			5	7, 11'	
H'	1.516, 1.661		4', 4'', 5'	5	3, 4, 7, 11	18
H''	1.516, 1.661		4', 4'', 5'	5	3, 4, 7, 11	18
6 C	50.356				7, 8'', 10, 11', 18	
7 C	33.321			7	5', 8', 11', 18	
H	2.330	6.60(18)	8', 8'', 18	7	1, 5, 6, 8, 11, 18	8'', 11'', 18
8 C	33.615			8', 8''	7, 10, 12, 18	
H'	1.563		7	8		18
H''	1.826		7	8	6, 7, 9, 10, 12	7, 11'', 18
9 C	137.433				8'', 11', 12, 13	
10 C	117.963			10	8'', 11', 12	
H	5.213		11', 11''	10	6, 8, 11, 12	11', 11'', 12, 13

Atom	δ (ppm)	J	COSY	HSQC	HMBC	NOESY
11 C	37.110			11', 11''	5', 7, 10	
H'	2.231		10, 11''	11	5, 6, 7, 9, 10	4', 10, 19
H''	1.951		10, 11'	11		7, 8', 10
12 C	37.375			12	8'', 10, 13, 14	
H2	1.946		13	12	8, 9, 10, 13, 14	10, 14
13 C	26.536			13	12, 14	
H2	2.063		12, 14	13	9, 12, 14, 15	10, 14
14 C	124.411			14	12, 13, 16, 17	
H	5.068		13, 16, 17	14	12, 13, 16, 17	12, 13, 16
15 C	131.568				13, 16, 17	
16 C	25.861			16	14, 17	
H3	1.677		14, 17	16	14, 15, 17	14
17 C	17.851			17	14, 16	
H3	1.599		14, 16	17	14, 15, 16	
18 C	16.993			18	7	
H3	0.735	6.60(7)	7	18	6, 7, 8	5', 7, 8', 8'', 20
19 C	27.757			19	3'', 20	
H3	1.097			19	1, 2, 3, 20	3', 4', 11'
20 C	27.304			20	3'', 19	
H3	1.075			20	1, 2, 3, 19	3', 3'', 18

Atom	δ (ppm)	HSQC	HMBC	NOESY
1 C	222.164			
2 C	44.610			
3 C	38.947	3		
H2		3		
4 C				
H2				
5 C	25.786		7	
H'	1.631			
H''	1.546			
6 C	50.811		7, 18	
7 C	33.032	7	9, 18	
H	2.269	7	5, 6, 8, 11, 18	
8 C	30.649	8', 8''	7, 18	
H'	1.596	8		9
H''	1.959	8		9
9 C	120.451	9		
H	5.369	9	7, 11, 12	8', 8'', 12, 13
10 C	135.010			
11 C	39.968	11', 11''	7, 9	
H'	2.086	11		
H''	1.934	11		

Atom	δ (ppm)	HSQC	HMBC	NOESY
12 C	37.707	12	9	
H2	1.929	12		9
13 C	26.726			
H2	2.039			9
14 C	124.341			
H	5.072			
15 C	131.630			
16 C	25.845			
H3				
17 C	17.892	17		
H3		17		
18 C	16.741	18	7	
H3	0.724	18	6, 7, 8	
19 C	27.933	19		
H3	1.118	19		
20 C	27.297	20		
H3	1.083	20		

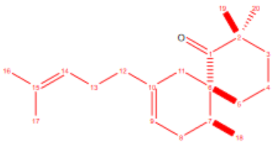

Table 3. Peak table for the adduct **7i** and **7i'**, COSY, HSQC, HMBC, NOESY signals for the assignment of relative stereochemistry.

(*S*)-2,2-dimethyl-9-(4-methylpent-3-en-1-yl)spiro[5.5]undec-8-en-1-one (**7j**) **and** (***S***)**-2,2-dimethyl-8-(4-methylpent-3-en-1-yl)spiro[5.5]undec-8-en-1-one** (**7j'**)

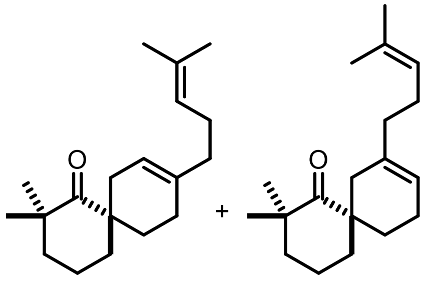

7j 7j'

The title compounds were prepared according to the representative procedure in 0.2 mmol scale at –60 °C for 5 d. The product was purified by column chromatography using 1-1.5% Et_2O in pentane as eluents and was obtained as colorless oil (49%, 1:1 r.r.). **^{1}H NMR** (501 MHz, $CDCl_3$, spectra contained 1:1 r.r.) δ 5.36–5.34 (m, 1H, for regioisome-1), 5.29–5.27 (m, 1H, for regioisome-2), 5.10–5.06 (m, 2H signal merge for both the regioisomers), 2.22–2.17 (m, 2H for regioisomer-1), 2.09–2.04 (m, 4H signal merge for both the regioisomers), 2.03–1.71 (m, 18H signal merge for both the regioisomers), 1.70–1.66 (brm, 10H signal merge for both the regioisomers), 1.60 (brs, 6H signal merge for both the regioisomers), 1.57–1.46 (m, 4H signal merge for both the regioisomers), 1.13 (s, 3H regioisomer-2), 1.12 (s, 3H regioisomer-2),1.11 (s, 3H regioisomer-1), 1.11 (s, 3H, regioisomer-1); **^{13}C NMR** (126 MHz, $CDCl_3$, spectra contains r.r. = 1:1) δ 220.5, 220.3, 136.3, 135.7, 131.6, 131.5, 124.5, 124.4, 119.1, 118.5, 47.1, 46.3, 44.87, 44.89, 40.2, 40.1, 38.0, 37.51, 37.50, 34.4, 34.1, 33.6, 30.9, 30.5, 27.8, 27.7, 27.6, 27.4, 26.7, 26.6, 25.86, 25.84, 25.0, 22.1, 17.87, 17.86, 17.8 (one peak was not detected); **HRMS** (GC-EI) (m/z) calculated for $C_{19}H_{30}O$ $[M]^+$: 274.229115, found: 274.229390; The enantiomeric ratio was determined by HPLC on a chiral stationary phase; HPLC column: Chiralcel OD-3, solvent system: n-heptane/2-PrOH = 99.8/0.2, flow rate: 1.0 mL/min., temp.: 25 °C, 220 nm, t_R (minor for regioisomer-2) = 5.1 min., t_R (major for regioisomer-2) = 5.4 min., e.r. = 6.5:93.5; t_R (minor for regioisomer-1) = 12.6 min., t_R (major for regioisomer-1) = 12.9 min., e.r. = 4:96.

(*S*)-5,5-dimethyl-9-(4-methylpent-3-en-1-yl)spiro[5.5]undec-8-en-1-one (**7k**)

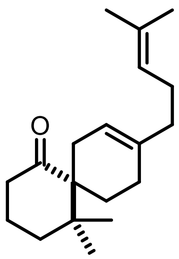

7k

The title compounds were prepared according to the representative procedure in 0.2 mmol scale at –60 °C for 5 d. The product was purified by column chromatography using 2-4% Et_2O in pentane as eluents and was obtained as colorless oil (82%, >20:1 r.r.). **^{1}H NMR** (501 MHz, $CDCl_3$, spectra contained >20:1 r.r.) δ 5.40–5.38 (m, 1H), 5.06 (tp, *J* = 7.0, 1.5 Hz, 1H), 2.62 (td, *J* = 12.9, 6.8 Hz, 1H), 2.35–2.30 (m, 1H), 2.24–2.15 (m, 2H), 2.04–1.98 (m, 3H), 1.94–1.83 (m, 5H), 1.82–1.76 (m, 1H), 1.71–1.65 (br, 5H), 1.58 (d, *J* = 1.3 Hz, 3H), 1.35–1.30 (m, 1H), 0.96 (s, 3H), 0.81 (s, 3H); **^{13}C NMR** (126 MHz, $CDCl_3$, spectra contains r.r. >20:1) δ 214.8, 135.6, 131.4, 124.6, 120.2, 55.2, 41.0, 37.4, 37.2, 35.7, 27.3, 26.6, 26.5, 26.2, 25.9, 25.0, 23.3, 23.2, 17.8; **HRMS** (GC-EI) (m/z) calculated for $C_{19}H_{30}O$ $[M]^+$: 274.229115, found: 274.229110; The enantiomeric ratio was determined by HPLC on a chiral stationary phase; HPLC column: Amycoat R, solvent system: CH_3CN/H_2O = 70/30, flow rate: 1.0 mL/min., temp.: 25 °C, 220 nm, t_R (major) = 11.9 min., t_R (minor) = 15.5 min., e.r. = 98:2; **$[\alpha]_D^{25}$** = +62.500 (*c* 0.192, $CHCl_3$).

(*R*)-3-methyl-8-(4-methylpent-3-en-1-yl)spiro[5.5]undeca-2,8-dien-1-one (**7l'**)

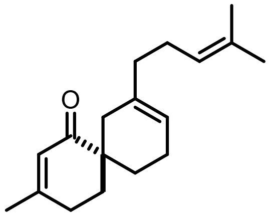

7l'

The title compound was prepared according to the representative procedure in 0.2 mmol scale at –80 °C for 6 d. The product was purified by column chromatography using 6-8% Et_2O in pentane as eluents and was obtained as colorless oil (91%, >12:1 r.r.). **^{1}H NMR** (501 MHz, $CDCl_3$, spectra contained >12:1 r.r.) δ 5.81 (q, *J* = 1.5 Hz, 1H), 5.36 (br, 1H), 5.11–5.07 (m, 1H), 2.45 (dq, *J* = 17.3, 2.8 Hz, 1H), 2.35–2.29 (m, 1H), 2.24–2.18 (m, 1H), 2.11–2.02 (m, 4H), 1.98–1.95 (m, 2H), 1.93 (brs, 3H), 1.90–1.85 (m, 1H), 1.83–1.75 (m, 1H), 1.71–1.65 (m, 4H), 1.60–1.53 (m, 5H); **^{13}C NMR** (126 MHz, $CDCl_3$ spectra contained rr>12:1, represented signals for the major regioisomer) δ 204.3, 160.2, 135.8, 131.6, 125.7, 124.4, 118.9, 42.5, 38.1, 34.5, 29.8, 28.3, 27.6, 26.7, 25.9, 24.1, 22.6, 17.9; **HRMS** (GC-EI) (m/z) calculated for $C_{18}H_{26}O$ $[M]^+$: 258.197815, found: 258.198010; The enantiomeric ratio was determined by HPLC on a chiral stationary phase; HPLC column: AD-3R, solvent system: CH_3CN/H_2O = 40/60, flow rate: 1.0 mL/min., temp.: 25 °C, 220 nm, t_R (minor for the major regioisomer) = 13.7 min., t_R (major for the major regioisomer) = 16.8 min., e.r. = 1:99; **$[\alpha]_D^{25}$** = +19.26 (*c* 0.322, $CHCl_3$).

NMR data support the following regioisomeric structures

For major regioisomer

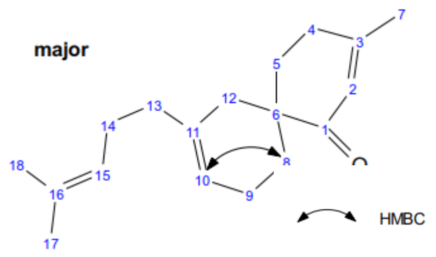

Atom	δ (ppm)	Min..Max (ppm)	J	COSY	HSQC	HMBC	NOESY
1 C	204.12	204.12..204.13				4', 4", 5', 5", 8', 8", 12', 12"	
2 C	125.55	125.54..125.55			2	4', 4", 7	
H	5.81	5.81..5.82		4', 4", 7	2	4, 6, 7	
3 C	160.11	160.10..160.11				4', 4", 5', 5", 7	
4 C	28.13	28.12..28.13			4', 4"	2, 7	
H'	2.32	2.29..2.35	19.00, 8.50, 5.10	2, 4", 5', 5", 7	4	1, 2, 3, 5, 6, 7	8"
H"	2.21	2.18..2.24	18.90, 5.40	2, 4', 5', 5", 7	4	1, 2, 3, 5, 6, 7	12"
5 C	29.62	29.62..29.63			5', 5"	4', 4", 8', 8", 12', 12"	
H'	1.87	1.85..1.90	13.80, 5.40	4', 4", 5"	5	1, 3, 6, 8, 12	
H"	1.78	1.75..1.81	13.70, 8.40, 5.20	4', 4", 5'	5	1, 3, 6, 8, 12	
6 C	42.31	42.30..42.31				2, 4', 4", 5', 5", 8', 8", 12', 12"	
7 C	23.99	23.99..23.99			7	2, 4', 4"	
H3	1.93	1.93..1.93		2, 4', 4"	7	2, 3, 4	
8 C	27.41	27.41..27.42			8', 8"	5', 5", 10, 12"	
H'	1.67	1.67..1.67	12.90, 10.50, 6.10	8", 9', 9"	8	1, 5, 6, 9, 10, 12	
H"	1.55	1.55..1.55		8', 9', 9"	8	1, 5, 6, 9, 10, 12	4'
9 C	22.39	22.39..22.39			9', 9"	8', 8", 10	
H'	2.07	2.06..2.07		8', 8", 9", 10	9	11	10
H"	2.04	2.03..2.04		8', 8", 9'	9	11	10

Atom	δ (ppm)	Min..Max (ppm)	J	COSY	HSQC	HMBC	NOESY
10 C	118.70	118.70..118.71			10	8', 8", 12', 12", 13	
H	5.36	5.33..5.39		9', 12', 12", 13	10	8, 9, 12, 13	9', 9", 13, 14
11 C	135.66	135.65..135.66				9', 9", 12', 12", 13, 14	
12 C	34.28	34.27..34.28			12', 12"	5', 5", 8', 8", 10, 13	
H'	2.45	2.42..2.49	17.20, 3.50, 2.50, 1.10	10, 12"	12	1, 5, 6, 10, 11	13, 14
H"	1.62	1.59..1.65	17.20	10, 12'	12	1, 5, 6, 8, 10, 11, 13	4"
13 C	37.94	37.93..37.94			13	10, 12", 14, 15	
H2	1.97	1.97..1.97		10, 14	13	10, 11, 12, 14, 15	10, 12'
14 C	26.54	26.53..26.54			14	13, 15	
H2	2.07	2.07..2.07		13, 15	14	11, 13, 15, 16	10, 12'
15 C	124.23	124.22..124.23			15	13, 14, 17, 18	
H	5.09	5.05..5.12		14, 17, 18	15	13, 14, 17, 18	17
16 C	131.40	131.39..131.40				14, 17, 18	
17 C	25.69	25.69..25.70			17	15	
H3	1.68	1.67..1.68		15	17	15, 16, 18	15
18 C	17.73	17.72..17.73			18	15, 17	
H3	1.60	1.59..1.60		15	18	15, 16	

For minor regioisomers

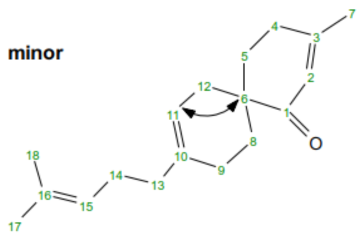

Atom	δ (ppm)	Min..Max (ppm)	COSY	HSQC	HMBC	NOESY
1 C	204.24	204.24..204.24				

Atom	δ (ppm)	Min..Max (ppm)	COSY	HSQC	HMBC	NOESY

2 C	125.58	125.58..125.59		2	4', 4", 7	
H	5.80	5.80..5.81		2	4, 6, 7	
3 C	160.01	160.00..160.01			4', 4", 5', 5", 7	
4 C	28.11	28.11..28.12		4', 4"	2, 7	
H'	2.31	2.28..2.34	4", 5', 5"	4	2, 3, 5, 6, 7	
H"	2.23	2.20..2.26	4', 5', 5"	4	2, 3, 5, 6, 7	
5 C	29.58	29.58..29.58		5', 5"	4', 4", 8', 8", 12', 12"	
H'	1.85	1.85..1.85	4', 4", 5"	5	3, 6, 8, 12	
H"	1.78	1.78..1.78	4', 4", 5'	5	3, 6, 8, 12	
6 C	41.75	41.75..41.76			2, 4', 4", 5', 5", 11	
7 C	23.98	23.97..23.98		7	2, 4', 4"	
H3	1.93	1.92..1.93		7	2, 3, 4	
8 C	27.98	27.98..27.98		8', 8"	5', 5", 12"	
H'	1.77	1.77..1.77	8", 9	8	5, 9, 10, 12	
H"	1.58	1.58..1.58	8', 9	8	5, 9, 10, 12	
9 C	25.30	25.29..25.30		9	8', 8", 11, 13	
H2	1.97	1.97..1.97	8', 8"	9	10, 11	
10 C	135.70	135.70..135.71			8', 8", 9, 12', 12", 14	
11 C	118.63	118.62..118.63		11	9, 12', 12", 13	
H	5.36	5.36..5.36	12', 12"	11	6, 9, 12, 13	12', 12", 13, 14
12 C	31.28	31.27..31.28		12', 12"	5', 5", 8', 8", 11	
H'	2.45	2.45..2.45	11, 12"	12	5, 10, 11	11
H"	1.75	1.75..1.75	11, 12'	12	5, 8, 10, 11	11
13 C	37.37	37.37..37.38		13	11, 15	
H2	1.97	1.97..1.97	14	13	9, 11, 14, 15	11
14 C	26.41	26.41..26.42		14	13, 15	
H2	2.07	2.07..2.07	13, 15	14	10, 15, 16	11
15 C	124.29	124.29..124.30		15	13, 14, 17, 18	
H	5.09	5.09..5.09	14	15	13, 14, 17, 18	
16 C	131.38	131.38..131.39			14, 17	
17 C	25.71	25.70..25.71		17	15, 18	
H3	1.68	1.67..1.68		17	15, 16, 18	
18 C	17.68	17.68..17.69			15, 17	
H3	1.60	1.59..1.60			15, 17	

Table 4. Peak table for the adduct **7l' and 7l**, COSY, HSQC, HMBC, NOESY signals for the assignment of relative stereochemistry. The absolute configuration of **7l'** was determined by converting to the corresponding Mosher's ester derivatives see *vide-infra*.

(*R*)-2-methyl-8-(4-methylpent-3-en-1-yl)spiro[5.5]undeca-2,8-dien-1-one (7m')

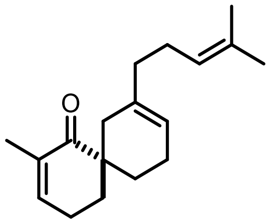

7m'

The title compound was prepared according to the representative procedure in 0.2 mmol scale at –80 °C for 7 d. The product was purified by column chromatography using 6-8% Et_2O in pentane as eluents and was obtained as colorless oil (41%, >15:1 r.r.). **^{1}H NMR** (600 MHz, $CDCl_3$, spectra contained >15:1 r.r.) δ 6.64–6.62 (m, 1H), 5.37–5.35 (m, 1H), 5.11–5.07 (m, 1H), 2.47–2.43 (m, 1H), 2.38–2.31 (m, 1H), 2.28–2.22 (m, 1H), 2.10–2.02 (m, 4H), 1.99–1.95 (m, 2H), 1.89–1.85 (m, 1H), 1.83–1.79 (m, 1H), 1.78 (m, 3H), 1.70–1.62 (m, 5H), 1.60 (brs, 3H), 1.59–1.55 (m, 1H); **^{13}C NMR** (151 MHz, $CDCl_3$, spectra contains r.r. >15:1) δ 204.7, 143.5, 135.9, 134.4, 131.6, 124.4, 118.9, 43.5, 38.1, 34.7, 30.3, 27.7, 26.7, 25.9, 22.9, 22.8, 17.9, 16.8; **HRMS** (GC-EI) (m/z) calculated for $C_{18}H_{26}O$ $[M]^+$: 258.197815, found: 258.197670; The enantiomeric ratio was determined by HPLC on a chiral stationary phase; HPLC column: OJ-3R, solvent system: CH_3CN/H_2O = 45/55, flow rate: 1.0 mL/min., temp.: 25 °C, 220 nm, t_R (major for the major regioisomer) = 34.0 min., t_R (minor for the major regioisomer) = 39.6 min., e.r. = 96:4; **$[\alpha]_D^{25}$** = +25.850 (*c* 0.147, $CHCl_3$).

NMR data supoorts the following structure:

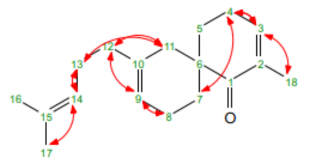

User Report
GHS-GB-130-01

Atom	δ (ppm)	J	COSY	HSQC	HMBC	NOESY
1 C	204.663				3, 5', 5", 7', 7", 11', 11", 18	
2 C	134.354				4", 18	
3 C	143.545			3	4", 5', 5", 18	
H	6.633		4', 4", 18	3	1, 4, 5, 18	4', 4", 18
4 C	22.889			4', 4"	3, 5', 5"	
H'	2.349		3	4		3, 7'
H"	2.252		3	4	2, 3, 5, 6	3, 11"
5 C	30.308			5', 5"	3, 4", 7", 11'	
H'	1.869	13.50(5")		5	1, 3, 4, 6, 7, 11, 12	
H"	1.795	13.50(5'), 8.24(4a), 5.41(4b)		5	1, 3, 4, 6, 7, 11	
6 C	43.493				4", 5', 5", 7', 7", 11'	
7 C	27.673			7', 7"	5', 5", 9, 11"	
H'	1.580			7	1, 6, 9, 11	4'
H"	1.673			7	1, 5, 6, 8, 9, 11	
8 C	22.757			8	7", 9	
H2	2.048		9	8	10	9
9 C	118.867			9	7', 7", 11', 11", 12	
H	5.355		8, 11', 11"	9	7, 8, 11, 12	8, 12
10 C	135.876				8, 11', 11", 12, 13	
11 C	34.678			11', 11"	5', 5", 7', 7", 9, 12	
H'	2.452		9	11	1, 5, 6, 9, 10	11", 12, 13
H"	1.645		9	11	1, 7, 9, 10, 12	4", 11'
12 C	38.083			12	5', 9, 11", 13, 14	
H2	1.970	7.60(13)		12	9, 10, 11, 13, 14	9, 11'
13 C	26.694			13	12, 14	
H2	2.074	7.60(12)	14	13	10, 12, 14, 15	11', 14
14 C	124.399			14	12, 13, 16, 17	
H	5.089		13, 16, 17	14	12, 13, 16, 17	13, 17
15 C	131.555				13, 16, 17	
16 C	17.880			16	14, 17	
H3	1.596		14	16	14, 15, 17	
17 C	25.853			17	14, 16	
H3	1.675		14	17	14, 15, 16	14
18 C	16.758			18	3	
H3	1.776		3	18	1, 2, 3	3

Remarks:

There are multiple complex multiplets in the 1H NMR. I regions with multiple overlaps the ^{1}H NMR shifts in the assignment table was extraxted from the 2D HSQC.

The shown regioisomer was confirmed by NOE data and HMBC data. H11 and H9 have both an NOE to H12. Additionally, H9 has a HMBC cross peaks to 4 different aliphatic non-quaternary carbons. This fits better to the shown regioisomer.

P-ID: ML00xxx
Measured on: 13/08/2021
CHIFFRE: GHS-GB-130-01
ELNA #: 6748

Table 5. Peak table for the adduct **7m'**, COSY, HSQC, HMBC, NOESY signals for the assignment of relative stereochemistry.

(*R*)-4,4-dimethyl-8-(4-methylpent-3-en-1-yl)spiro[5.5]undeca-2,8-dien-1-one (**7n'**)

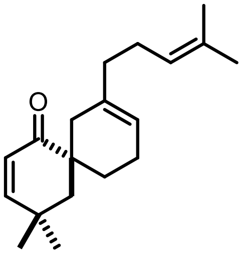

7n'

The title compound was prepared according to the representative procedure in 0.2 mmol scale at –60 °C for 5 d. The product was purified by column chromatography using 2-4% Et_2O in pentane as eluents and was obtained as colorless oil (70%, 18:1 r.r.). **^{1}H NMR** (600 MHz, $CDCl_3$, spectra contained 18:1 r.r.) δ 6.56 (dd, *J* = 10.1, 1.2 Hz, 1H), 5.84 (d, *J* = 10.1 Hz, 1H), 5.38 (m, 1H), 5.11–5.07 (m, 1H), 2.57–2.53 (m, 1H), 2.10–2.03 (m, 4H), 1.98–1.94 (m, 2H), 1.88 (dd, *J* = 14.5, 1.3 Hz, 1H), 1.73–1.65 (m, 6H), 1.64–1.61 (m, 1H), 1.60 (br, 3H), 1.21 (s, 3H), 1.14 (s, 3H); **^{13}C NMR** (151 MHz, $CDCl_3$, spectra contains r.r. = 18:1) δ 204.6, 157.3, 135.7, 131.6, 125.6, 124.4, 118.8, 43.5, 42.5, 38.2, 37.3, 33.2, 31.9, 30.5, 29.7, 26.6, 25.9, 22.0, 17.9; **HRMS** (GC-EI) (m/z) calculated for $C_{19}H_{28}O$ $[M]^+$: 272.213465, found: 272.213550; The enantiomeric ratio was determined by GC; on a chiral column: 24.0 m Cycloextrin-H 0.2510.125df:G/632, temp.: 220/120, 300 min. iso. 6/min. 180, 3 min. iso / 350; Gas: 0.50 bar H_2 gas; t_R (minor) = 203.6 min., t_R (major) = 252.4 min., e.r. >99.5:0.5; **$[\alpha]_D^{25}$** = +35.573 (*c* 0.253, $CHCl_3$).

(*R*)-3-(4-methylpent-3-en-1-yl)-3',4'-dihydro-1'H-spiro[cyclohexane-1,2'-naphthalen]-3-en-1'-one (**7o'**)

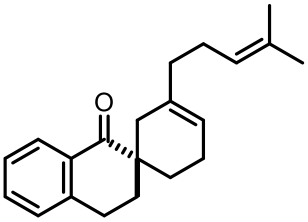

7o'

The title compound was prepared according to the representative procedure in 0.2 mmol at –80 °C for 5 d. The product was purified by column chromatography using 2-3% Et_2O in pentane as eluents and was obtained as colorless oil (87%, r.r. > 18:1). **^{1}H NMR** (600 MHz, $CDCl_3$, spectra contained >18:1 r.r.) δ 8.04 (dd, *J* = 7.9, 1.5 Hz, 1H), 7.46 (td, *J* = 7.5, 1.5 Hz, 1H), 7.32–7.29 (m, 1H), 7.23–7.21 (m, 1H), 5.43–5.41 (m, 1H), 5.12–5.09 (m, 1H), 3.02 (ddd, *J* = 17.2, 9.6, 4.9 Hz, 1H), 2.90 (dt, *J* = 17.3, 5.4 Hz, 1H), 2.63–2.51 (m, 1H), 2.15–2.04 (m, 5H), 2.02–1.99 (m, 2H), 1.95 (ddd, *J* = 14.0, 9.6, 4.9 Hz, 1H), 1.78–1.72 (m, 2H), 1.71–1.66 (m, 4H), 1.61 (d, *J* = 1.4 Hz, 3H); **^{13}C NMR** (151 MHz, $CDCl_3$, spectra contains r.r. = 15:1) δ 202.8, 143.4, 135.8, 133.1, 132.0, 131.6, 128.8, 128.2, 126.7, 124.4, 119.0, 43.9, 38.1, 34.8, 30.3, 27.5, 26.7, 25.9, 25.4, 22.6, 17.9; **HRMS** (GC-EI) (m/z) calculated for $C_{21}H_{26}O$ $[M]^+$: 294.197815, found: 294.197540; The enantiomeric ratio was determined by HPLC on a chiral stationary phase; HPLC column: AD-3R, solvent system: CH_3CN/H_2O = 70/30, flow rate: 1.0 mL/min., temp.: 25 °C, 220 nm, t_R (major for the major regioisomer) = 13.0 min., t_R (minor for the major regioisomer) = 17.1 min., e.r. = 93:7; **$[\alpha]_D^{25}$** = +51.118 (*c* 0.313, $CHCl_3$).

(1*R*,2*S*,3*R*,4*S*)-3-methylspiro[bicyclo[2.2.1]heptane-2,1'-cyclohexan]-5-en-2'-one (**7p**)

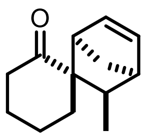

7p

The title compound was prepared according to the representative procedure in 0.2 mmol scale and in the presence of catalyst **11j** (Ar = 3-Ph-C_6H_4 and R = C_6F_5) at –80 °C for 5 d. The product was purified by column chromatography using 2-4% Et_2O in pentane as eluents and was obtained as colorless oil (95% yield, *exo*:*endo*.= 5:1). **^{1}H NMR** (501 MHz, $CDCl_3$, for *exo* isomer) δ 6.24 (dd, *J* = 5.8, 2.9 Hz, 1H), 6.19 (dd, *J* = 5.8, 3.2 Hz, 1H), 3.08 (br, 1H), 3.03 (qd, *J* = 7.4, 3.5 Hz, 1H), 2.69–2.61 (m, 2H), 2.42–2.37 (m, 1H), 2.07–2.02 (m, 1H), 1.72–1.66 (m, 3H), 1.46 (dq, *J* = 14.0, 3.1 Hz, 1H), 1.33–1.22 (m, 3H), 0.66 (d, *J* = 7.4 Hz, 3H); **^{1}H NMR** (501 MHz, $CDCl_3$, for *endo* isomer) δ 6.21 (dd, *J* = 5.6, 3.0 Hz, 1H), 5.78 (dd, *J* = 5.7, 2.8 Hz, 1H), 3.15 (br, 1H), 2.50–2.44 (m, 2H), 2.43–2.37 (m, 1H), 2.30–2.26 (m, 1H), 2.12–2.08 (m, 1H), 1.92 (tt, *J* = 13.5, 3.8 Hz, 1H), 1.85–1.77 (m, 2H), 1.65–1.57 (m, 2H), 1.37 (dq, *J* = 9.0, 1.9 Hz, 1H), 1.33–1.22 (m, 1H), 0.92 (d, *J* = 7.4 Hz, 3H); **^{13}C NMR** (126 MHz, $CDCl_3$, for *exo* isomer) δ 214.0, 137.9, 134.3, 59.7, 50.7, 48.6, 46.0, 40.1, 35.2, 33.3, 27.8, 22.8, 14.6; **^{13}C NMR** (126 MHz, $CDCl_3$, for *endo* isomer) δ 213.2, 139.7, 131.3, 58.5, 50.2, 49.0, 44.6, 41.0, 35.0, 34.7, 28.7, 23.2, 16.4; **HRMS** (ESI pos) (m/z) calculated for $C_{13}H_{18}NaO$ $[M+Na]^+$: 213.124984, found: 213.125060; The enantiomeric ratio was determined by HPLC on a chiral stationary phase; HPLC column: AD-3R, solvent system: CH_3CN/H_2O = 40/60, flow rate: 1.0 mL/min., temp.: 25 °C, 220 nm, t_R (minor for *exo* isomer) = 28.2 min., t_R (major for *exo* isomer) = 30.7 min., e.r. (*exo*) = 5:95.
Reaction in the presence of catalyst **11g**: Reaction temp: –60 °C, time : 5 d, Conv. : full *exo*:*endo*.= 1:2, e.r. (*exo*) = 53:47; e.r. (*endo*) = 59:41.

(6*R*,11*R*)-8,9,11-trimethylspiro[5.5]undec-8-en-1-one (**7q**)

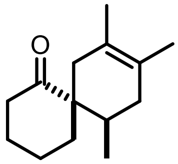

7q

The title compound was prepared according to the representative procedure in 0.2 mmol scale at –60 °C for 3 d. The product was purified by column chromatography using 2-3% Et_2O in pentane as eluents and was obtained as colorless oil (95% yield). **^{1}H NMR** (501 MHz, $CDCl_3$) δ 2.48–2.42 (m, 1H), 2.34–2.27 (m, 2H), 1.98 (m, 1H), 1.92– 1.87 (m, 2H), 1.84–1.73 (m, 3H), 1.69–1.62 (m, 3H), 1.61 (brs, 3H), 1.58–1.53 (m, 4H), 0.86 (d, *J* = 6.7 Hz, 3H); **^{13}C NMR** (126 MHz, $CDCl_3$) δ 215.9, 123.7, 122.6, 51.5, 39.1, 38.6, 37.2, 31.4, 30.7, 27.0, 20.6, 19.0, 18.9, 15.5; **HRMS** (GC-EI) (m/z) calculated for $C_{14}H_{22}O$ $[M]^+$: 206.166515, found: 206.166580; The enantiomeric ratio was determined by chiral GC; Column: 25.0 m Hydrodex-gamma-TBDAc-CD .,0.25/ df G/624; Temperature: 220/120, 35 min. iso 8/min 250,3 min. iso/350; Gas: 0.60 bar H_2(g); t_R (minor) = 25.0 min., t_R (major) = 28.4 min.; e.r. = 2.5:97.5; **$[\alpha]_D^{25}$** = –37.97 (*c* 0.295, $CHCl_3$).

(6*R*,11*R*)-9,11-dimethylspiro[5.5]undec-8-en-1-one (**7r**)

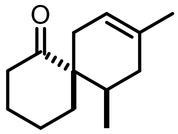

7r

The title compound was prepared according to the representative procedure in 0.2 mmol scale at –60 °C for 5 d. The product was purified by column chromatography using 2-3% Et_2O in pentane as eluents and was obtained as colorless oil (73% yield, >20:1 r.r). **^{1}H NMR** (501 MHz, $CDCl_3$, spectra contained >20:1 r.r) δ 5.28–5.25 (m, 1H), 2.49–2.43 (m, 1H), 2.39–2.27 (m, 2H), 2.08–2.02 (m, 1H), 1.99–1.94 (m, 1H), 1.94–1.88 (m, 1H), 1.86–1.73 (m, 3H), 1.70–1.52 (m, 7H), 0.89 (d, *J* = 6.8 Hz, 3H); **^{13}C NMR** (126 MHz, $CDCl_3$) δ 215.9, 132.3, 118.1, 50.6, 39.1, 35.5, 32.4, 31.2, 30.9, 27.0, 23.5, 20.6, 15.6; **HRMS** (GC-EI) (m/z) calculated for $C_{13}H_{20}O$ $[M]^+$: 192.150865, found: 192.150810; The enantiomeric ratio was determined by chiral GC; Column: 25.0 m Hydrodex-gamma-TBDAc-CD 0.25/ df G/624; Temperature: 220/120, 30 min. iso. 8/min. 240, 3 min. iso/350; Gas: 0.50 bar H_2(g); t_R (minor) = 23.4 min., t_R (major) = 25.1 min.; e.r. = 6:94; **$[\alpha]_D^{25}$** = –44.280 (*c* 0.271, $CHCl_3$).

Resolution of (±)-12

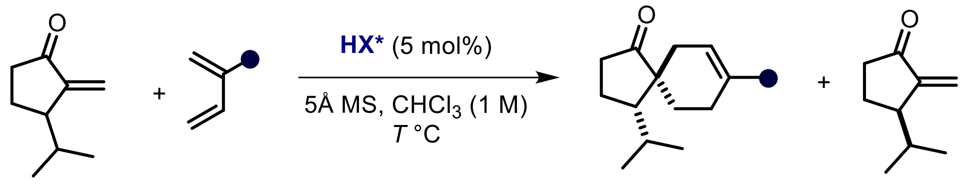

(±)-12 6a or 6d 13a-b (+)-12

● = 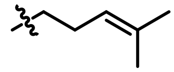

● = Me

13a:
[Reaction condition –80 °C , 8 d]
For 13a:
31% yield, 98:2 e.r., >20:1 r.r., >20:1 d.r.
For recovered 12:
34% yield, 84:16 e.r.

13b:
[Reaction condition: –60 °C , 6 d]
For 13b:
37% yield, 91:9 e.r., >20:1 r.r. 13:1 d.r.
For recovered 12:
36% yield, 82:18 e.r.

(4*S*,5*S*)-4-isopropyl-8-(4-methylpent-3-en-1-yl)spiro[4.5]dec-7-en-1-one (**13a**)

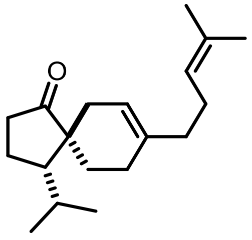

13a

The title compound was prepared according to the representative procedure in 0.3 mmol scale in the presence of catalyst **11g** at –80 °C for 8 d. Then the reaction mixture was treated with 30 µL Et3N and the reaction vial was stirred at –80 °C for additional 10 min. Afterwards the crude reaction mixture was filtered through short pad silica gel to separate the catalyst from the mixture of starting material **12** and product **13a**, using 10% Et_2O in pentane mixture as eluent. Then the mixture of **13a** and **12** was separated by reverse phase preparative TLC plate using 50/50 mixture of CH_3CN/H_2O as eluent. The silica gel was extracted using 20% (V/V) Et_2O in pentane (20 mL) mixture and concentrated under reduce pressure. The corresponding Diels–Alder adduct **13a** was obtained as colorless oil (31% yield, >20:1 r.r., >20:1 d.r.) and remaining (+)-**12** was recovered in (34% yield) [N.B.: Starting material is low boiling colorless liquid, hence during the evaporation of the solvent low temp. water bath was used]. ^{1}**H NMR** (501 MHz, $CDCl_3$ spectra contained 18:1 r.r.) δ 5.34 (br, 1H), 5.12–5.08 (m, 1H), 2.32–2.19 (m, 2H), 2.12–2.06 (m, 3H), 2.04–1.81 (m, 7H), 1.79–1.70 (m, 3H), 1.68 (brs, 3H), 1.60 (s, 3H), 1.59–1.55 (m, 1H), 0.97 (d, J = 6.7 Hz, 3H), 0.82 (d, J = 6.7 Hz, 3H); ^{13}**C NMR** (126 MHz, $CDCl_3$ spectra contained 18:1 r.r.) δ 223.2, 137.8, 131.5, 124.5, 118.1, 50.4, 50.3, 37.7, 35.9, 33.1, 28.3, 26.5, 25.87, 25.4, 23.1, 22.8, 20.6, 19.8, 17.9; **HRMS** (ESI pos+neg) (m/z) calculated for $C_{19}H_{30}NaO$ $[M+Na]^+$: 297.218884, found: 297.219270; The enantiomeric ratio was determined by chiral GC; Column: 25.0 m Hydrodex-gamma-TBDAc-CD .0.25/ df G/624; Temperature: 220/150, 80 min. iso, 6/min. 240, 3 min. iso/350; Gas: 0.50 bar H_2(g); t_R (major) = 74.5 min., t_R (minor) = 77.3 min.; e.r. = 98:2; $[\alpha]_D^{25}$ = –50.242 (c 0.207, $CHCl_3$). The absolute configuration of **13a** was assigned by analogy in comparison to **13b** (*vide-infra*).

Characterization of the recovered (+)-**12**

(*R*)-3-isopropyl-2-methylenecyclopentan-1-one (+)-**12** [9c]

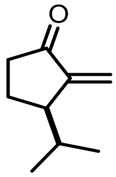

(+)-**12**

^{1}**H NMR** (501 MHz, $CDCl_3$) δ 6.09 (d, J = 2.8 Hz, 1H), 5.24 (dd, J = 2.5, 1.1 Hz, 1H), 2.71–2.66 (m, 1H), 2.40–2.34 (m, 1H), 2.30–2.23 (m, 1H), 2.01–1.93 (m, 2H), 1.70 (dq, J = 12.8, 8.1 Hz, 1H), 1.00 (d, J = 6.8 Hz, 3H), 0.87 (d, J = 6.7 Hz, 3H); ^{13}**C NMR** (126 MHz, $CDCl_3$) δ 208.0, 147.8, 117.8, 47.3, 37.2, 30.1, 21.4, 20.8, 17.8; The enantiomeric ratio was determined by HPLC on a chiral stationary phase; HPLC column: AD-3R, solvent system: CH_3CN/H_2O = 50/50, flow rate: 1.0 mL/min., temp.: 25 °C, 220 nm, t_R (minor) = 5.7 min., t_R (major) = 6.1 min., e.r. = 16:84, $[\alpha]_D^{25}$ = +27.59 (c 0.058, $CHCl_3$).
Absolute configuration of the recovered enone **12** was determined in comparison of specific rotation data to the literature known value.[9c] Literature report for **(*S*)-3-isopropyl-2-methylenecyclopentan-1-one** (-)-**12** is $[\alpha]_D^{20}$ = –36.2 (c 0.64, $CHCl_3$) for single enantiomer.

(4*S*, 5*S*)-4-isopropyl-8-methylspiro[4.5]dec-7-en-1-one (**13b**)[9c]

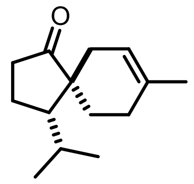

13b

The title compound was prepared according to the representative procedure in 0.3 mmol scale and in the presence of catalyst **11g** at –60 °C for 6 d. Then the reaction mixture was treated with 30 μL Et3N and the reaction vial was stirred at –60 °C for additional 10 min. Afterwards the crude reaction mixture was filtered through short pad silica gel to separate the catalyst from the mixture of starting material **12** and product **13b**, using 10% Et_2O in pentane mixture as eluent. Then the mixture of **13b** and **12** was separated by reverse phase preparative TLC plate using 55/45 mixture of CH_3CN/H_2O as eluent. The silica gel was extracted using 20% (V/V) Et_2O in pentane (20 mL) mixture and concentrated under reduce pressure. The corresponding Diels–Alder adduct **13b** was obtained as colorless oil (37% yield, >20:1 r.r., 13:1 d.r. along with some unidentified isomeric products ca. 3%) and the starting material was recovered in (36% yield) [N.B.: Starting material is low boiling colorless liquid, hence during the evaporation of the solvent low temp. water bath was used] **^{1}H NMR** (501 MHz, $CDCl_3$, spectra contained 13:1 r.r.) δ 5.34–5.32 (m, 1H), 2.32–2.18 (m, 2H),2.13–2.07 (m, 1H), 2.04–1.97 (m, 2H), 1.93–1.80 (m, 3H), 1.77–1.70 (m, 3H), 1.68 (brs, 3H), 1.63–1.53 (m, 1H), 0.98 (d, J = 6.8 Hz, 3H), 0.83 (d, J = 6.7 Hz, 3H); **^{13}C NMR** (126 MHz, $CDCl_3$, spectra contained 13:1 r.r.) δ 223.2, 134.1, 118.5, 50.4, 50.1, 35.9, 33.1, 28.3, 27.1, 23.7, 22.9, 22.8, 20.8, 19.9; **HRMS** (ESI pos.) (m/z) calculated for $C_{14}H_{23}O$ $[M+H]^+$: 207.174340, found: 207.174700; The enantiomeric ratio was determined by chiral GC; Column: 30.0 m BGB-176/BGB-15 0,25/0,25df G/618; Temperature: 220/120 70 min. iso, 6/min. 220, 3/350; Gas: 0.60 bar H_2(g); t_R (minor) = 59.2 min., t_R (major) = 61.3 min.; e.r. = 9:91; **$[\alpha]_D^{25}$** = –52.85 (c 0.167, $CHCl_3$). The absolute configuration of the compound **13b** was determined in comparison of specific rotation data to the literature known value.[9c] Literature report for (**4*S*, 5*S*)-4-isopropyl-8-methylspiro[4.5]dec-7-en-1-one** (**13b**) is **$[\alpha]_D^{20}$** = –99.8 (c 0.45, $CHCl_3$) for single enantiomer.

Characterization data of recovered starting material (*R*)-3-isopropyl-2-methylenecyclopentan-1-one 12 has well agreement to the literature report[9c]: The enantiomeric ratio was determined by HPLC on a chiral stationary phase; HPLC column: AD-3R, solvent system: CH_3CN/H_2O = 50/50, flow rate: 1.0 mL/min., temp.: 25 °C, 220 nm, t_R (minor) = 5.7 min., t_R (major) = 6.1 min., e.r. = 18:82; **$[\alpha]_D^{25}$** = **+23.93** (c 0.118, $CHCl_3$).

Natural Product Synthesis

Synthesis of Spirocarbocycle (14)

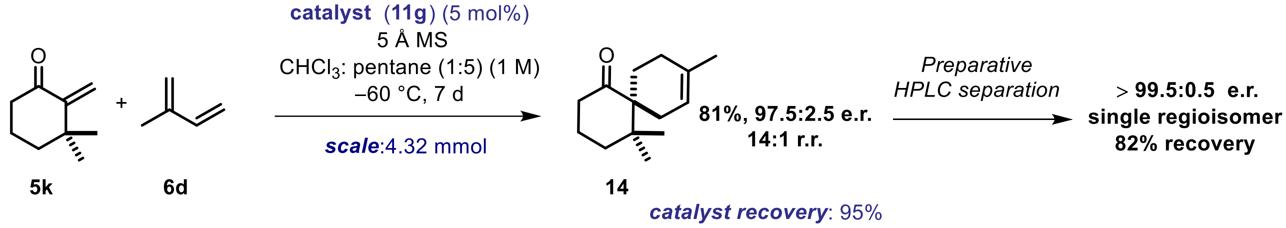

[**N.B.**: Reaction with our optimized solvent ($CHCl_3$, 1 M) also provided **14** in similar stereo- as well as regioselectivities (97:3 e.r.; 14.1 r.r.), but with lower yield 44% (due to the decomposition of the enone). After modification of the reaction solvent system to $CHCl_3$/pentane mixture, the yield of the transformation was improved to 81%.]

A flame-dried schlenk flask with a magnetic stirring plate, cooled under the argon, was charged with catalyst (420 mg, 5 mol%, 0.216 mmol), 5Å MS (520 mg) in (1:5) $CHCl_3$:pentane mixture (1 M, 4.32 mL). Then the flask was cooled down to –60 °C using acetone dry ice bath, and freshly prepared enone **5k** (597 mg, 4.32 mmol, 1.0 equiv.) was slowly transferred to the reaction flask [enone **5k** is unstable even at –20°C, hence it was instantaneously prepared and used for the reaction]. Afterwards isoprene **6d** (1.73 mL, 17.28 mmol, 4.0 equiv.) was added through the wall of the flask, the reaction vessel was placed into a cryostat maintaining temperature –60 °C, and stirring was continued for 7 d. After that, the reaction mixture was treated with Et_3N (0.3 mL) and stirred for additional 10 min. at –60 °C. Then the reaction mixture was warmed up to r.t. and Purification was performed by column chromatography on silica gel using 2-4% Et_2O in pentane mixture as eluents, afforded the desired adduct (**14**) as color less solid (722 mg, 81% yield, 14:1 r.r., 97:3 e.r.) [**N.B.**: The minor regioisomer as well as enantiomer was separated by using preparative chiral HPLC and the major regioisomer was recovered in 82% yield, > 99.5:0.5 e.r.]. The catalyst was recovered from the silica gel column using 15-20% EtOAc in hexane as eluent, and obtained as off-white solid, which was subjected to acidification. The solid was dissolved in 30 mL CH_2Cl_2 and 6.0 M HCl (aq) 30 mL was added and vigorously stirred at r.t. for 30 minutes.

Afterwards the organic layer was collected and again washed with 6.0 M HCl (aq) (2 x 30 mL). Then the organic layer separated, concentrated, and dried under reduced pressure to afford the desired acid catalyst as an off-white solid (397 mg, 95% recovery of the catalyst **11g**).

(*S*)-5,5,9-trimethylspiro[5.5]undec-8-en-1-one (14)

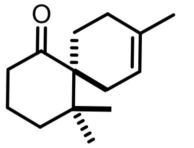

14

^{1}H NMR (501 MHz, $CDCl_3$) δ 5.39–5.37 (m, 1H), 2.63 (td, *J* = 12.9, 6.8 Hz, 1H), 2.34–2.27 (m, 1H), 2.23–2.18 (m, 2H), 2.01 (td, *J* = 13.4, 4.7 Hz, 1H), 1.93–1.74 (m, 4H), 1.73–1.67 (m, 2H), 1.58 (br, 3H), 1.35– 1.31 (m, 1H), 0.96 (s, 3H), 0.81 (s, 3H); **^{13}C NMR** (126 MHz, $CDCl_3$) δ 215.1, 131.9, 120.6, 55.0, 41.0, 37.2, 35.7, 30.5, 27.9, 27.2, 26.5, 25.0, 23.4, 23.2; **HRMS** (GC-EI) (m/z) calculated for $C_{14}H_{22}O$ $[M]^+$: 206.166515, found: 206.166790; The enantiomeric ratio was determined by chiral GC; Column: 30.0 m G-TA 0.25/df; G/448; Temperature: 220/110 62 min. iso, 8 °C/ min.; Gas: 0.60 bar H_2(g); t_R (major) = 52.1 min., t_R (minor) = 55.9 min.; e.r. = 97.5:2.5; Preparative HPLC condition: the minor regioisomer and enantiomer were separated by preparative HPLC on a chiral stationary phase; HPLC column: 150 mm Chiralcel OJ-3R, 4.6 mm, solvent system: CH_3CN/H_2O = 70/30, flow rate: 1.0 mL/min., temp.: 25 °C, 220 nm, t_R (minor region isomer) = 3.5 min., t_R (major region isomer) = 5.2 min. Collected solvent (water and acetonitrile mixture) was concentrated under reduce pressure using low temperature water bath. Then the concentrated solution was extracted with Et_2O and concentrated, afforded the desired adduct as a single regioisomer with >99.5:0.5 e.r.; **$[\alpha]_D^{25}$** = +82.235 (*c* 0.448, $CHCl_3$). The absolute configuration of **14** was assigned by analogy in comparison to **4a**, **4b** and **4c** (*vide-infra*).

Synthesis and Characterization of (+)-*β*-chamigrene (4b)

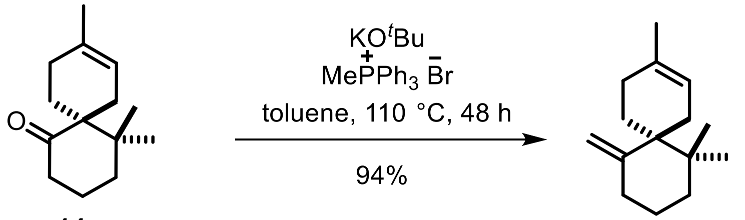

14
> **99.5:0.5 e.r.**
single regioisomer

>**99.5:0.5 e.r.**
(+)–*β*-chamigrene (**4b**)

A flame-dried schlenk flask under argon, charged with KO^{t}Bu (24 mg, 0.218 mmol, 1.5 equiv.) in dry toluene (1 mL). Into that solution was added methyltriphenylphosphonium bromide (78 mg, 0.218 mmol, 1.5 equiv.) and placed the reaction schlenk-flask over a preheated oil bath maintaining temperature 100 °C, and stirred for 30 mintutes. Then the mixture was cooled to rt and the enantiopure spiro-ketone **14** (30 mg, 0.145 mmol, 1.0 equiv.) was added and the reaction mixture stirred at 110 °C for 48h. Afterwards the reaction vessel was cooled down to r.t. and diluted with Et_2O (5 mL), treated with water (2 mL), the layers were separated, the aq. layer was extracted with Et_2O (3 x 5 mL), the combined organic layers were dried over anhydrous Na_2SO_4 and concentrated in vacuo. Purification by column chromatography on silica gel using pentane as eluent afforded title compound **4b** as colorless oil (28 mg, 94% yield). **^{1}H NMR** (501 MHz, $CDCl_3$) δ 5.31–5.29 (m, 1H), 4.87 (t, *J* = 2.0 Hz, 1H), 4.51 (d, *J* = 2.0 Hz, 1H), 2.27–2.20 (m, 1H), 2.13–2.06 (m, 2H), 1.99–1.91 (m, 2H), 1.80–1.72 (m, 2H), 1.69– 1.62 (m, 1H), 1.57 (brs, 3H), 1.56–1.44 (m, 3H), 1.15 (m, 1H), 0.87 (s, 3H), 0.82 (s, 3H); **^{13}C NMR** (126 MHz, $CDCl_3$) δ 149.4, 133.0, 120.3, 110.7, 45.0, 37.5, 37.3, 32.5, 29.3, 28.2, 26.2, 25.3, 24.1, 23.5, 23.3; **HRMS** (GC-EI) (m/z) calculated for $C_{15}H_{24}$ $[M]^+$: 204.187250, found: 204.186850; The enantiomeric ratio was determined by chiral GC; Column: 30.0 m G-TA 0.25/df; G/448; Temperature: 220/60 230 min. iso, 6 °C/ min. 3 min. iso/350; Gas: 0.60 bar H_2(g); t_R (minor) = 194.6 min.; t_R (major) = 205.1 min.; e.r. > 99.05:0.5; **$[\alpha]_D^{25}$** = +85.217 (*c* 0.115, $CHCl_3$).

Comparison of synthetic and Natural ^{1}H NMR, and Specific rotation data for (+)- *β*-chamigrene (4b)

**Our Report**	**Natural for (+)-*β*-chamigrene** [12]
**^{1}H NMR** (501 MHz, $CDCl_3$) (δ in ppm)	**^{1}H NMR** (90 MHz, CCl_4)
5.31–5.29 (m, 1H)	5.22 (m, 1H)
4.87 (t, *J* = 2.0 Hz, 1H)	4.82 (d, *J* = 2.0 Hz, 1H),

4.51 (d, J = 2.0 Hz, 1H)	4.47 (d, J = 2.0 Hz, 1H),
2.27–2.20 (m, 1H); 1.99–1.91 (m, 2H), 1.80–1.72 (m, 2H), 1.69– 1.62 (m, 1H)	--
1.57 (brs, 3H),	1.55 (brs, 3H)
1.56–1.44 (m, 3H); 1.15 (m, 1H),	--
0.87 (s, 3H), 0.82 (s, 3H)	0.87 (s, 3H), 0.82 (s, 3H)
**[α]$_D$**^{25} = +85.217 (c 0.115, $CHCl_3$)	**[α]$_D$** = +66 (c 1.0, $CHCl_3$)

Synthesis and Characterization of (+)-*α*-chamigrene (***ent*-4a**)

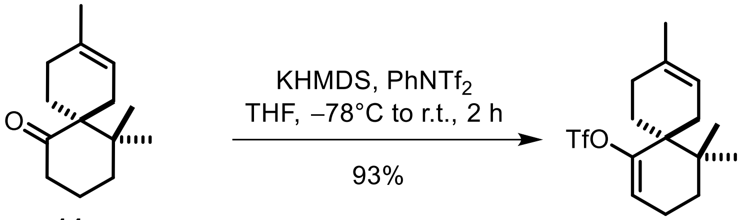

14
> **99.5:0.5 e.r.**
single regioisomer

15

Synthesis of (*S*)-5,5,9-trimethylspiro[5.5]undeca-1,8-dien-1-yl trifluoromethanesulfonate (**15**)
In a flame-dried schlenk flask under argon, enantiopure spiro-ketone (**14**) (50 mg, 0.24 mmol, 1.0 equiv.) and $PhNTf_2$ (95 mg, 0.27 mmol, 1.1 equiv.) were dissolved in THF (0.4 mL). Then the flask was cooled to –78 °C, and a solution of KHMDS (0.5 M in toluene) (0.73 mL, 0.36 mmol, 1.5 equiv.) was added drop wise. Afterwards the reaction mixture was slowly warm up to r.t. over 2 h with constant stirring. Then the reaction mixture was treated with brine (5 mL), and diluted with Et_2O (5 mL). The organic layer was separated, dried over anhydrous Na_2SO_4, and concentrated under reduce pressure. Purification of the crude material by flash column chromatography using 1% Et_2O in pentane as eluent yielded the desired product as a colorless oil (76 mg, 93% yield). **^{1}H NMR** (501 MHz, $CDCl_3$) δ 5.74 (t, J = 3.9 Hz, 1H), 5.36 (br, 1H), 2.25–2.12 (m, 3H), 2.06–1.90 (m, 3H), 1.78–1.68 (m, 3H), 1.64 (br, 3H), 1.26 (ddd, J = 13.4, 6.2, 2.8 Hz, 1H), 0.95 (s, 3H), 0.94 (s, 3H); **^{13}C NMR** (126 MHz, $CDCl_3$) δ 155.7, 133.9, 120.6, 119.6, 116.0, 42.5, 37.8, 31.9, 30.2, 28.3, 27.8, 24.7, 23.3, 23.0, 21.5; **^{19}F NMR** (471 MHz, $CDCl_3$) δ –75.16; **HRMS** (ESIpos) (m/z) calculated for $C_{15}H_{21}O_3S_1F_3Na$ $[M+Na]^+$: 361.105572, found: 361.105980; **[α]$_D$**^{25} = +48.718 (c 0.156, $CHCl_3$).

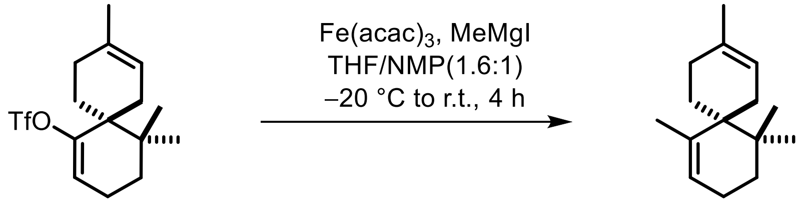

15

α-chamigrene
(**4a***)*
45%; e**.r. > 99.5:0.5**

Synthesis of *α*-chamigrene (***ent*-4a**)
A flame-dried schlenk flask under argon, charged with triflate **15** (52 mg, 0.154 mmol, 1.0 equiv.) in a mixture THF (2.5 mL) and NMP (1.5 mL). Then $Fe(acac)_3$ (17 mg, 0.048 mmol, 0.3 equiv.) was added to the solution and resulted into a red color solution which was then cooled to –15 °C, and methylmagnesium iodide (3 M in Et_2O) (0.153 mL mg, 0.461 mmol, 3.0 equiv.) was added dropwise. The resulting yellow suspension was then slowly warm up to r.t. and stirred for 2 h (TLC analysis showed incomplete conversion of the starting material). Again the reaction flask was cooled to 0 °C and another portion of methylmagnesium iodide (0.153 mL mg, 0.461 mmol, 3.0 equiv.) was added and

stirring was continued for further 2 h. Afterwards the solution was treated with H_2O (10 mL) and diluted with Et_2O (20 mL). The organic layer was separated and the aqueous layer was washed with Et_2O (3 X 10 mL). The combined organic layer dried over anhydrous Na_2SO_4, and concentrated under reduce pressure. The crude material was purified by flash chromatography using pentane as eluent and afforded the desired α-chamigrene with some unknown impurity as colorless oil (20 mg). The unknown impurity was removed by preparative $AgNO_3$ coated silica gel TLC plate, using 1% Et_2O in pentane mixture as eluent and afforded the *α*-chamigrene as colorless oil (a minor unidentified impurity 2% by area was present) (14 mg, 45%). **^{1}H NMR** (501 MHz, $CDCl_3$) δ 5.46–5.45 (m, 1H), 5.34–5.32 (m, 1H), 2.12 (m, 1H), 2.00–1.91 (m, 4H), 1.85 (d, *J* = 18.3 Hz, 1H), 1.75–1.63 (m, 9H), 1.19 (ddd, *J* = 13.5, 6.1, 4.1 Hz, 1H), 0.89 (s, 3H), 0.83 (s, 3H); **^{13}C NMR** (126 MHz, $CDCl_3$) δ 140.5, 134.0, 123.0, 122.6, 40.7, 36.1, 33.0, 30.9, 29.9, 29.2, 29.1, 25.3, 23.6, 23.5, 23.2; **HRMS** (GC-EI) (m/z) calculated for $C_{15}H_{24}$ $[M]^+$: 204.187250, found: 204.187140; The enantiomeric ratio was determined by chiral GC; Column: 30.0 m G-TA 0.25/df; G/448; Temperature: 220/60 300 min. iso, 6 °C/ min. 180, 3 min. iso/350; Gas: 0.60 bar H_2(g); t_R (minor) = 237.7 min.; t_R (major) = 245.3 min.,; e.r. > 99.05:0.5; **$[\alpha]_D^{25}$** = +45.176 (*c* 0.213, $CHCl_3$).

Comparison of synthetic and published ^{1}H NMR, ^{13}C NMR, and Specific rotation data for *α*-chamigrene (4a)

**Our Report**	**Stoltz Report**[13b]	**Natural** [13a]
**^{1}H NMR** (501 MHz, $CDCl_3$) (δ in ppm)	**^{1}H NMR** (500 MHz, $CDCl_3$) (δ in ppm)	**^{1} H NMR** (300 MHz, **CCl4**) (δ in ppm)
5.46–5.45 (m, 1H)	5.44–5.48 (m, 1H),	5.3 (unresolved m, 2H)
5.34–5.32 (m, 1H)	5.31–5.35 (br m, 1H)	
2.12 (m, 1H)	2.12 (dddd, *J* = 18.1, 5.1, 2.4, 2.4 Hz, 1H),	
2.00–1.91 (m, 4H),<br>1.85 (d, *J* = 18.3 Hz, 1H)	1.80–2.01 (brm, 5H),	
1.75–1.63 (m, 9H)	1.60–1.79 (m, 9H)	
1.19 (ddd, *J* = 13.5, 6.1, 4.1 Hz, 1H)	1.19 (ddd, *J* = 13.7, 6.1, 4.39 Hz, 1H)	1.63 (br s, 6H)
0.89 (s, 3H)<br>0.83 (s, 3H)	0.89 (s, 3H)<br>0.83 (s, 3H)	0.89 (s, 3H)<br>0.84 (s, 3H)
**^{13}C NMR** (126 MHz, $CDCl_3$) (δ in ppm)	**^{13}C NMR** (126 MHz, $CDCl_3$) (δ in ppm)	**Not reported**
140.5	140.3	
134.0	133.8	
123.0	122.8	
122.6	122.5	
40.7	40.5	
36.1	35.9	
33.0	32.8	
30.9	30.7	
29.9	29.1	
29.2	28.9	
29.1	25.1	
25.3	23.47	
23.6	23.46	
23.5 (br)	23.3 (br)	
23.2	23.0	

**[α]$_D^{25}$** = +45.176 (*c* 0.213, $CHCl_3$).	**[α]$_D^{26}$** = –64.60° (c 0.21, $CHCl_3$)	**[α]$_D$** = –14.5° (c 0.21, $CHCl_3$)

Synthesis of (+)-laurencenone C (*ent*-4c)

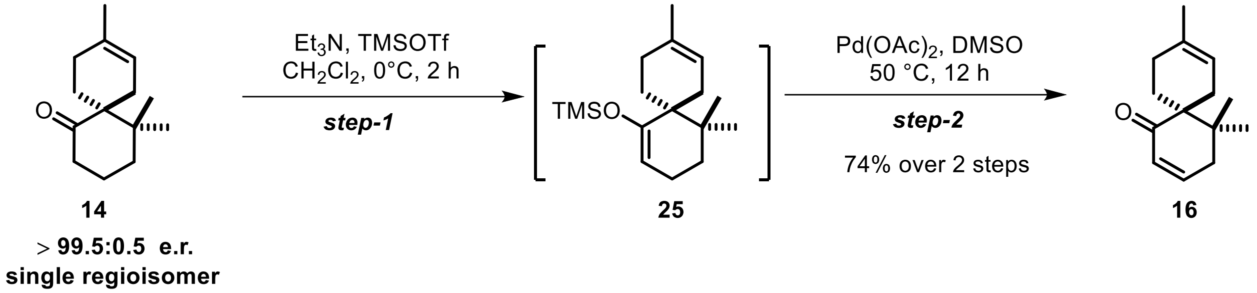

Synthesis of (*S*)-5,5,9-trimethylspiro[5.5]undeca-2,8-dien-1-one (16)

Step1

To a solution of enantiopure ketone **14** (100 mg, 0.48 mmol, 1.0 equiv.) in dry CH_2Cl_2 (3 mL) under argon at 0 °C was added Et_3N (0.202 mL , 1.45 mmol, 3 equiv.). Afterwards TMSOTf (0.132 mL , 0.727 mmol, 1.5 equiv.) was added slowly to the solution and the resulting mixture was stirred for 2 h at 0 °C. Then the reaction mixture was treated with saturated aqueous $NaHCO_3$ (5 mL) and the aqueous phase was extracted with CH_2Cl_2 (3 x10 mL). The combined organic layer were washed with brine (10 mL), dried over anhydrous Na_2SO_4 and concentrated under reduce pressure. The crude was directly used for the next step without further purification.

Step 2

The crude TMS enol-ether **24** was dissolved in DMSO (3 mL). Then $Pd(OAc)_2$ (163 mg, 0.727 mmol, 1.5 equiv.) was added to the solution. Afterwards the reaction vessel was placed over a 50 °C oil bath and stirring was continued for 12 h. Then the reaction vessel was cooled down to r.t. and diluted with Et_2O (10 mL), H_2O (15 mL) was added and vigorously stirred the mixture. The organic layer was separated and the aqueous layer was washed with Et_2O (2 X 15 mL). The combined organic layer was dried over anhydrous Na_2SO_4 and concentrated under reduce pressure. The crude material was purified by column chromatography on silica gel using 1.5%–5%) Et_2O/pentane mixture as eluent to afford the desired enone **16** as colorless oil (73 mg, 74% over 2 steps). **^{1}H NMR** (501 MHz, $CDCl_3$) δ 6.63 (ddd, *J* = 10.2, 5.1, 2.7 Hz, 1H), 5.91 (ddd, *J* = 10.1, 3.0, 1.3 Hz, 1H), 5.43 (brs, 1H), 2.50–2.47 (m, 1H), 2.22 (dd, *J* = 17.5, 5.0 Hz, 1H), 2.01 (dd, *J* = 33.0, 18.8 Hz, 2H), 1.88–1.83 (m, 2H), 1.72 (m, 2H), 1.65–1.59 (m, 3H), 1.02 (s, 3H), 0.91 (s, 3H); **^{13}C NMR** (151 MHz, $CDCl_3$) δ 204.4, 144.8, 132.2 (br), 127.8, 120.4 (br), 51.3, 39.7, 39.2, 28.0, 25.8 (br), 24.4, 23.6, 23.5; **HRMS** (GC-EI) (m/z) calculated for $C_{14}H_{20}O$ $[M]^+$: 204.150865, found: 204.151130; **[α]$_D^{25}$** = +161.468 (*c* 0.164, $CHCl_3$).

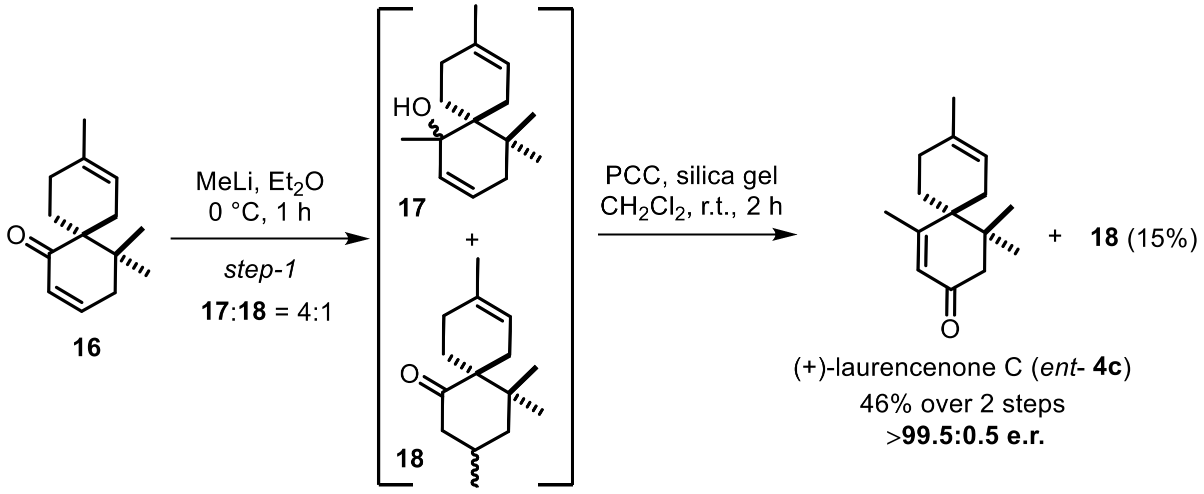

Synthesis of laurencenone C (*ent*-4c)

Step1:

In a flame-dried schlenk flask under argon, enone **16** (60 mg, 0.293 mmol, 1.0 equiv.) was dissolved in Et_2O (0.25 mL) and cooled down to 0 °C. To this solution was added MeLi (1.6 M in Et_2O) (0.367 mL, 0.587 mmol, 2.0 equiv.) and stirring was continued for 1 h. Afterwards the reaction mixture was treated with saturated NH_4Cl (aq.) solution (5 mL) and diluted with Et_2O (10 mL). The organic layer was separated and dried over anhydrous Na_2SO_4, concentrated under reduce pressure. The crude material (contained 1,2-addition product **17** and 1,4-addition product **18** in 4:1 ratio) was directly used for the next step without further purification.

Step2:

To a solution of PCC (139 mg, 0.646 mmol, 2.2 equiv.) in CH_2Cl_2 (1 mL) under argon atmosphere was added silica gel (60 mg). Then the crude allylic alcohol mixture (**17** and **18**) (obtained from step 1) was dissolved in CH_2Cl_2 (2 mL) and slowly added to the reaction mixture at r.t.. The resulting blackish solution was allowed to stir for 2 h at r.t. (TLC analysis indicated the complete consumption of the allylic alcohol). Afterwards the crude reaction mixture was directly poured on the silica gel column and eluted with 8-20% Et_2O in pentane mixture, afforded the desired product laurencenone C (***ent*-4c**) as yellow oil (30 mg, 46% yield). and 1,4-addition **18** product also separated from the mixture and obtained as colorless oil (11 mg, 15%). **^{1}H NMR** (501 MHz, $CDCl_3$) δ 5.86 (br s, 1H), 5.50 (d, *J* = 3.9 Hz, 1H), 2.62 (d, *J* = 18.1 Hz, 1H), 2.27–2.21 (m, 1H), 2.07–1.97 (m, 6H), 1.92–1.84 (m, 2H), 1.79–1.76 (m, 1H), 1.68 (br, 3H), 1.03 (s, 3H), 0.95 (s, 3H); **^{13}C NMR** (126 MHz, $CDCl_3$) δ 198.9, 170.6, 134.3, 127.2, 121.8, 49.1, 43.6, 40.6, 30.8 (br), 28.4, 28.2, 25.0, 24.4, 24.1 (br), 23.5; **HRMS** (GC-EI) (m/z) calculated for $C_{15}H_{22}O$ $[M]^+$: 218.166515, found: 218.166280; The enantiomeric ratio was determined by chiral GC; Column: 25.0 m Hydrodex-gamma-TBDAc-CD .,0.25/ df G/624; Temperature: 220/150, 40 min. iso 6/min 240, 3 min. iso/350; Gas: 0.60 bar H_2(g); t_R (minor) = 29.0 min., t_R (major) = 32.2 min.; > 99.05:0.5 e.r.; $[\alpha]_D^{25}$ = +82.131 (*c* 0.45, $CHCl_3$).

Comparison of synthetic and published ^{1}H NMR, ^{13}C NMR, and Specific rotation data for *α*-laurencenone (4a)

**Our Report for**<br>(+) **laurencenone C**	**Stoltz Report**[13b]<br>(-) **laurencenone C**	**Natural (isolation)**[14]<br>(-) **laurencenone C**
**^{1}H NMR** (501 MHz, $CDCl_3$) (δ in ppm)	**^{1}H NMR** (500 MHz, $CDCl_3$) (δ in ppm)	**^{1}H NMR** (400 MHz, $CHCl_3$) (δ in ppm)
5.86 (br s, 1H)	5.87 (br s, 1H)	5.87 (s, 1H)
5.50 (d, *J* = 3.9 Hz, 1H)	5.47–5.53 (br m, 1H)	5.50 (m, 1H)
2.62 (d, *J* = 18.1 Hz, 1H)	2.48–2.76 (br m, 1H)	1.98 (s, 3H)
2.27–2.21 (m, 1H)	2.19–2.28 (br m, 1H)	1.68 (s, 3H)
2.07–1.97 (m, 6H)	1.82–2.14 (br m, 5H)	1.03 (s, 3H)
1.92–1.84 (m, 2H)	1.97 (d, *J* = 1.5 Hz, 3H)	0.95 (s, 3H)
1.79–1.76 (m, 1H)	1.71–1.82 (br m, 1H)	
1.68 (br, 3H)	1.68 (br s, 3H)	
1.03 (s, 3H)	1.03 (s, 3H)	
0.95 (s, 3H)	0.95(s, 3H)	
**^{13}C NMR** (126 MHz, $CDCl_3$) (δ in ppm)	**^{13}C NMR** (126 MHz, $CDCl_3$) (δ in ppm)	**^{13}C NMR** (100 MHz, $CDCl_3$) (δ in ppm)
198.9	198.6	198.5
170.6	170.4	170.3
134.3	134.1	131.4
127.2	127.0	127.0
121.8	121.6	121.6
49.1	48.9	49.0
43.6	43.4	43.4
40.6	40.4	40.4
30.8 (br)	30.6 (br)	30.6
28.4	28.2	28.2
28.2	27.9	28.0

25.0	24.8	24.8
24.4	24.2	24.2
24.1 (br)	23.9 (br)	23.4
23.5	23.3	23.3
**[α]D**^{25} = +82.131 (*c* 0.451, $CHCl_3$)	**[α]D**^{26} = –87.98 (*c* 1.0, $CHCl_3$)	**[α]D** = –43 (*c* 1, $CHCl_3$)

Absolute Stereochemistry Assignment

Absolute configuration of the spirocarbocyle **14** was assigned after converting to the (+)-***α*-chamigrene** (***ent*-4a**), (+)-***β*-chamigrene** (**4b**), and (+)-**laurencenone C** (***ent*-4c**). The relative configuration of **7a-k** were assigned by analogy.

The relative stereochemistry for the regioisomers **7l′** and **7l** were assigned by COSY, HMBC, HSQC, NOESY NMR analysis (see page **S15-17**). The absolute configuration of **7l′** was determined by converting to the corresponding Mosher's ester derivatives **27a** and **27b** as described below and the relative configuration of **7m′-o′** were assigned by analogy.

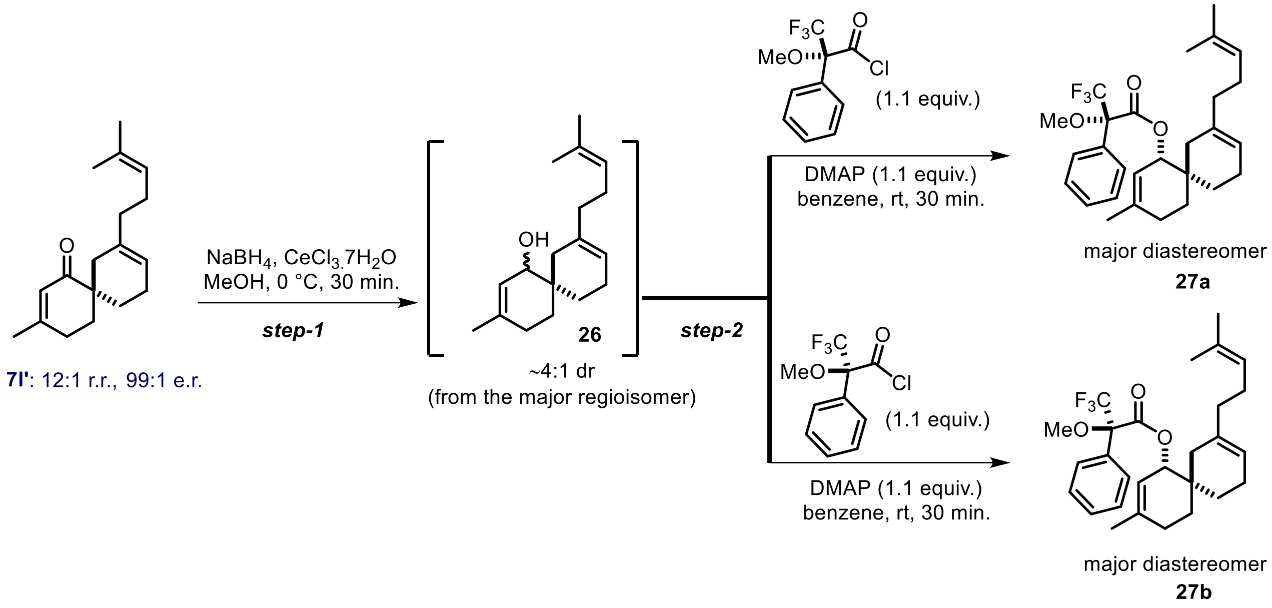

Procedure for the synthesis of compound 26
(***step-1***)
A round bottom flask, with a magnetic stirring bar was charged with **7l′** (12:1 r.r., 99:1 e.r.) (1.0 equiv., 0.077 mmol, 20 mg) in MeOH (2.0 mL) at 0 °C. Then $CeCl_3.7H_2O$ (1.2 equiv., 0.093 mmol, 35 mg) was added. Afterwards $NaBH_4$ (1.5 equiv., 0.116 mmol, 4.4 mg) was added to the solution and stirring was continued for 30 minutes. Then the reaction mixture was diluted with CH_2Cl_2 (10 mL), and treated with H_2O (5 mL). The organic layer was collected, dried over anhydrous Na_2SO_4 and concentrated under reduced pressure, afforded the crude alcohol **26** as mixture of regio- and diastereoisomers. The crude material was directly used for the ***step 2***.

General procedure for the synthesis of ester 27 (***step-2***)
A flame dried Schlenk flask, under argon was charged with crude **26** (obtained from the ***step-1***) (1.0 equiv., 0.038 mmol, 10 mg) in benzene (0.1 mL) at 0 °C. Afterwards DMAP (1.0 equiv., 0.038 mmol, 5 mg) and (*S*)-3,3,3-trifluoro-2-methoxy-2-phenylpropanoyl chloride or (*R*)-3,3,3-trifluoro-2-methoxy-2-phenylpropanoyl chloride (2.0 equiv., 0.076 mmol, 20 mg) were added to the solution and stirred for 20 minutes. Then the reaction mixture was filtered through a short anhydrous MgSO4 pad. The crude mixture was collected and purified by flash column chromatography using 1-2% Et_2O in hexane as eluent and afforded the desired product **27** as inseparable mixture of region- and diastereomers. (Unoptimized reaction condition was used)

(1*S*,6*R*)-3-methyl-8-(4-methylpent-3-en-1-yl)spiro[5.5]undeca-2,8-dien-1-yl (*S*)-3,3,3-trifluoro-2-methoxy-2-phenylpropanoate (27a):

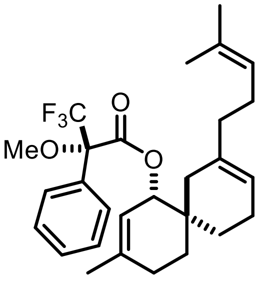

27a

The desired product was synthesized using general reaction procedure mentioned above, in the presence of (*R*)-3,3,3-trifluoro-2-methoxy-2-phenylpropanoyl chloride. Compound was obtained as inseparable mixture of regio- and diastereomers. The configuration of the major diastereomer obtained from major regioisomer was determined by HSQC, HMBC, COSY, NOESY spectra to confirm the absolute configuration of the spiro Diels–Alder product **7l′**. **^{1}H NMR** (600 MHz, $CDCl_3$,) δ 7.53–7.50 (m, 2H), 7.40–7.38 (m, 3H), 5.57-5.55 (m, 1H), 5.40-5.39 (m, 1H), 5.11 (d, *J* = 4.7 Hz, 1H), 5.07 (ddq, *J* = 8.4, 5.6, 1.4 Hz, 1H), 3.50 (m, 3H), 2.08 – 2.01 (m, 3H), 1.99 – 1.89 (m, 5H), 1.89 – 1.83 (m, 1H), 1.74 – 1.68 (m, 7H), 1.60 (br, 2H), 1.57 (br, 1H), 1.53 (m, 2H), 1.40-1.36 (m, 1H), 1.33-1.29 (m, 1H); **^{13}C NMR** (151 MHz, $CDCl_3$) δ 166.5, 141.7, 135.1, 132.4, 131.6, 129.6, 128.4, 127.9, 124.3, 123.6, 120.5, 117.7, 84.9 (q), 76.1, 55.4, 37.9, 35.6, 33.9, 29.1, 28.7, 27.4, 26.7, 25.9, 23.4, 22.1, 17.9; **^{19}F NMR** (565 MHz, $CDCl_3$) δ –71.54; **HRMS** (ESIpos) (m/z) calculated for $C_{28}H_{35}O_3F_3Na$ $[M + Na]^+$: 499.243049, found: 499.243240.

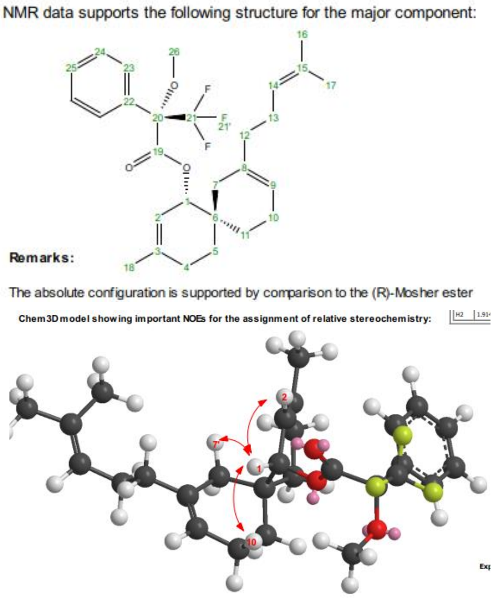

Atom	δ (ppm)	COSY	HSQC	HMBC	NOESY
1 C	76.129		1	5', 5", 7', 7", 11"	
H	5.112	2, 18	1	2, 3, 5, 6, 7	2, 5', 7', 10, 11', 23, 26
2 C	117.718		2	1, 4, 5", 18	
H	5.561	1, 4, 18	2	4, 6, 18	1, 18, 23, 26
3 C	141.706			1, 4, 5', 5", 18	
4 C	27.396		4	2, 5', 5", 18	
H2	1.960	2, 5', 5"	4	2, 3, 5	18
5 C	29.052		5', 5"	1, 4, 7', 11', 11"	
H'	1.308	4, 5"	5	1, 3, 4, 6, 11	1, 5"
H"	1.709	4, 5'	5	1, 2, 3, 4, 6, 7	5'
6 C	33.862			1, 2, 5', 5", 7', 7", 10, 11', 11"	
7 C	35.602		7', 7"	1, 5", 11', 11", 12	
H'	1.865	7", 9	7	1, 5, 6, 8, 9, 11	1, 7"
H"	1.707	7', 9	7	1, 6, 8, 9, 12	7'
8 C	135.083			7', 7", 12, 13	
9 C	120.494		9	7', 7", 10, 11', 11", 12	
H	5.396	7', 7", 10, 12	9		10, 12, 13
10 C	22.081		10	11', 11"	
H2	1.951	9, 11', 11"	10	6, 9, 11	1, 9, 23
11 C	28.665		11', 11"	5', 7', 10	
H'	1.532	10, 11"	11	5, 6, 7, 9, 10	1, 11", 23
H"	1.378	10, 11'	11	1, 5, 6, 7, 9, 10	11'
12 C	37.904		12	7", 13	
H2	1.914	9, 13	12	7, 8, 9, 13, 14	9, 14

Atom	δ (ppm)	COSY	HSQC	HMBC	NOESY
13 C	26.671		13	12, 14	
H2	2.037	12, 14	13	8, 12, 14	9, 14, 17
14 C	124.317		14	12, 13, 16, 17	
H	5.071	13, 16, 17	14	13, 16, 17	12, 13, 16
15 C	131.641			16, 17	
16 C	25.859		16	14, 17	
H3	1.683	14	16	14, 15, 17	14, 17
17 C	17.887		17	14, 16	
H3	1.598	14	17	14, 15, 16	13, 16
18 C	23.376		18	2	
H3	1.692	1, 2	18	2, 3, 4	2, 4
19 C	166.538				
20 C	84.929			23, 26	
21 C	123.572				
21' F	-71.544				
22 C	132.441			24	
23 C	127.860		23	23, 25	
H	7.520	24	23	20, 23, 25	1, 2, 10, 11', 26
24 C	128.397		24	24	
H	7.390	23	24	22, 24	
25 C	129.565		25	23	
H	7.380		25	23	
26 C	55.418		26		
H3	3.504		26	20	1, 2, 23

Table 6. Peak table for **27a**, COSY, HSQC, HMBC, NOESY signals for the assignment of relative stereochemistry.

(1*S*,6*R*)-3-methyl-8-(4-methylpent-3-en-1-yl)spiro[5.5]undeca-2,8-dien-1-yl (*R*)-3,3,3-trifluoro-2-methoxy-2-phenylpropanoate (27b)

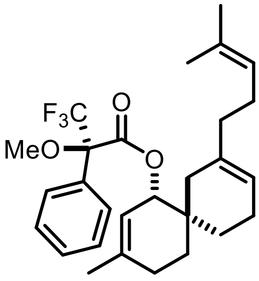

27b

The desired product was formed using general reaction procedure mentioned above, in the presence of (*S*)-3,3,3-trifluoro-2-methoxy-2-phenylpropanoyl chloride. Compound was obtained as inseparable mixture of regio- and diastereomers. The configuration of the major diastereomer obtained from major regioisomer was determined by HSQC, HMBC, COSY, NOESY spectra to confirm the absolute configuration of the spiro Diels-Alder product **7l′**.**^{1}H NMR** (600 MHz, $CDCl_3$) δ 7.56–7.54 (m, 2H), 7.39–7.35 (m, 3H), 5.63 (m, 1H), 5.38 (br, 1H), 5.12 (d, *J* = 4.9 Hz, 1H), 5.06 (m, 1H), 3.58–3.55 (m, 3H), 2.06–1.98 (m, 4H), 1.93–1.79 (m, 5H), 1.74 (br, 3H), 1.69-1.67 (br, 4H), 1.60 (m, 3H), 1.44–1.39 (m, 1H), 1.34–1.23 (m, 3H); **^{13}C NMR** (151 MHz, $CDCl_3$) δ 166.4, 142.0, 134.9, 133.1, 131.6, 129.5, 128.4, 127.4, 124.3, 120.6, 118.2, 117.8, 84.6 (q), 75.4, 55.6, 37.9, 35.4, 33.8, 29.4, 28.8, 27.5, 26.7, 25.9, 23.4, 22.0, 17.9; **^{19}F NMR** (565 MHz, $CDCl_3$) δ -71.32 (spectra contained excess Mosher's acid chloride); **HRMS** (ESIpos) (m/z) calculated for $C_{28}H_{35}O_3F_3Na$ $[M + Na]^+$: 499.243049, found: 499.243240.

NMR data supports the following structure for the major component:

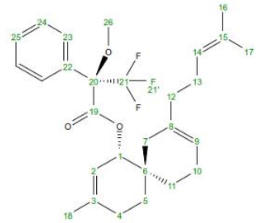

Remarks:

The absolute configuration is supported by comparison to the (*S*)-Mosher ester

The sample contains various other side components, which were not further analysed. [1]H signals of regions with overlaps were assigned from the cross peak in the HSQC. The signals of H10' & H10" and H4' & H4" could not be destinguished although they would be exspected to be diastereotopic. The strong NOE from 1 to H7' and H10 is nicely seen in the 1D NOESY.

Atom	δ (ppm)	COSY	HSQC	HMBC	NOESY	Atom	δ (ppm)	COSY	HSQC	HMBC	NOESY
1 C	75.430		1	2, 5', 5", 7', 7", 11"		13 C	26.659		13	12, 14	
H	5.124	2, 18	1	2, 3, 5, 6, 7, 11, 19	2, 5', 7', 10, 11'	H2	2.024	12, 14, 16, 17	13	8, 12, 14, 15	9, 14, 17
2 C	117.795		2	1, 4, 5", 18		14 C	124.335		14	12, 13, 16, 17	
H	5.625	1, 4, 18	2	1, 4, 6, 18	1, 18, 23	H	5.063	13, 16, 17	14	12, 13, 16, 17	12, 13, 16
3 C	142.009			1, 4, 5', 5", 18		15 C	131.617			13, 16, 17	
4 C	27.448		4	2, 5', 5", 18		16 C	25.855		16	14, 17	
H2	2.007	2, 5', 5"	4	2, 3, 5	18	H3	1.683	13, 14	16	14, 15, 17	14, 17
5 C	29.402		5', 5"	1, 4, 7', 11', 11"		17 C	17.880		17	14, 16	
H'	1.314	4, 5"	5	1, 3, 4, 6, 11	1, 5"	H3	1.597	13, 14	17	14, 15, 16	13, 16
H"	1.734	4, 5'	5	1, 2, 3, 4, 6, 7	5'	18 C	23.402		18	2	
6 C	33.842			1, 2, 5', 5", 7', 7", 10, 11', 11"		H3	1.742	1, 2	18	2, 3, 4	2, 4
7 C	35.423		7', 7"	1, 5", 9, 11', 11", 12		19 C	166.396			1	
H'	1.827	7", 9	7	1, 5, 6, 8, 9, 11, 12	1, 7"	20 C	84.286			23, 26	
H"	1.701	7', 9	7	1, 6, 8, 9, 12	7'	21 C	123.650				
8 C	134.933			7', 7", 10, 12, 13		21' F	-71.323				
9 C	120.632		9	7', 7", 10, 11', 11", 12		22 C	133.092			24	
H	5.377	7', 7", 10, 12	9	7, 10, 11, 12	10, 12, 13	23 C	127.442		23	23, 25	
10 C	21.979		10	9, 11', 11"		H	7.547	24	23	20, 23, 25	2, 26
H2	1.856	9, 11', 11"	10	6, 8, 9	1, 9	24 C	128.357		24	24	
11 C	28.759		11', 11"	1, 5', 7', 9		H	7.363	23	24	22, 24	
H'	1.411	10, 11"	11	5, 6, 7, 9, 10	1, 11"	25 C	129.534		25	23	
H"	1.259	10, 11'	11	1, 5, 6, 7, 9, 10	11'	H	7.354		25	23	
12 C	37.888		12	7', 7", 9, 13, 14		26 C	55.595		26		
H2	1.901	9, 13	12	7, 8, 9, 13, 14	9, 14	H3	3.567		26	20	23

Chem3D model showing important NOEs for the assignment of relative stereochemistry:

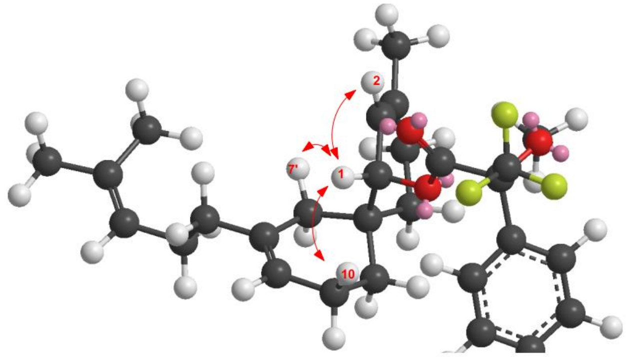

Table 7. Peak table for **27b**, COSY, HSQC, HMBC, NOESY signals for the assignment of relative stereochemistry.

**27a** (*S*)-Ester		**27b** (*R*)-Ester		
Atom	δ (ppm)	Atom	δ (ppm)	$\Delta\delta^{SR}$
1 C	76.129	1 C	75.43	
H	5.112	H	5.124	-0.01
2 C	117.718	2 C	117.795	
H	5.561	H	5.625	-0.06
3 C	141.706	3 C	142.009	
4 C	27.396	4 C	27.448	
H2	1.96	H2	2.007	-0.05
5 C	29.052	5 C	29.402	
H'	1.308	H'	1.314	-0.01
H"	1.709	H"	1.734	-0.02
6 C	33.862	6 C	33.842	
7 C	35.602	7 C	35.423	
H'	1.865	H'	1.827	0.04
H"	1.707	H"	1.701	0.01
8 C	135.083	8 C	134.933	
9 C	120.494	9 C	120.632	
H	5.396	H	5.377	0.02
10 C	22.081	10 C	21.979	
H2	1.951	H2	1.856	0.10
11 C	28.665	11 C	28.759	
H'	1.532	H'	1.411	0.12
H"	1.378	H"	1.259	0.12
12 C	37.904	12 C	37.888	
H2	1.914	H2	1.901	0.01
13 C	26.671	13 C	26.659	
H2	2.037	H2	2.024	0.01
14 C	124.317	14 C	124.335	
H	5.071	H	5.063	0.01
15 C	131.641	15 C	131.617	
16 C	25.859	16 C	25.855	
H3	1.683	H3	1.683	0.00
17 C	17.887	17 C	17.88	
H3	1.598	H3	1.597	0.00
18 C	23.376	18 C	23.402	
H3	1.692	H3	1.742	-0.05
19 C	166.538	19 C	166.396	
20 C	84.929	20 C	84.286	
21 C	123.572	21 C	123.65	
21' F	-71.544	21' F	-71.323	
22 C	132.441	22 C	133.092	
23 C	127.86	23 C	127.442	
H	7.52	H	7.547	-0.03
24 C	128.397	24 C	128.357	
H	7.39	H	7.363	0.03
25 C	129.565	25 C	129.534	
H	7.38	H	7.354	0.03
26 C	55.418	26 C	55.595	
H3	3.504	H3	3.567	-0.06

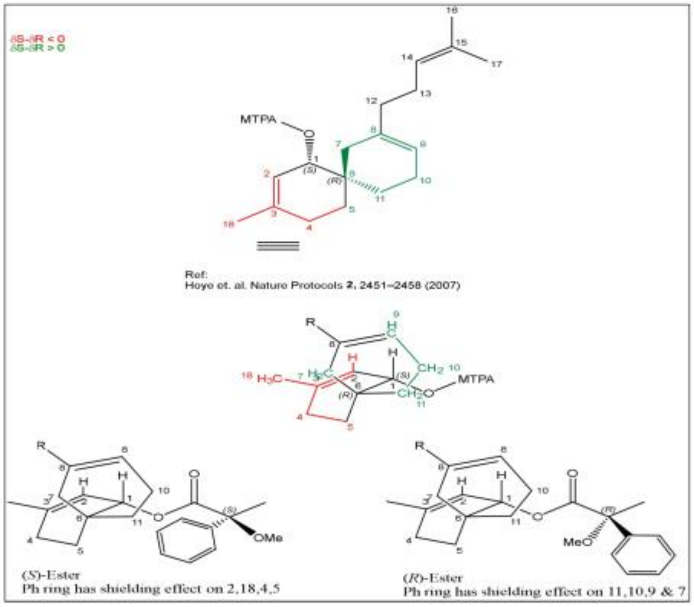

The Mosher analysis following the Hoye et. al. Nature protocol procedure suggests that the configuration at C1 is *(S)*.

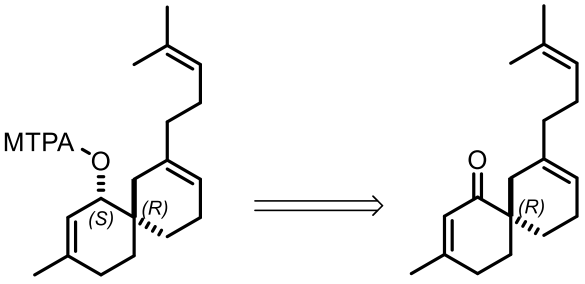

27 **7l'**

Table 8: Confirmation of Absolute Configuration of **7l′**

Computational Studies

1. Method

All calculations presented in this paper were carried out with a development version of the ORCA suite of programs base on version 4.2.[15] Molecular geometries were optimized in the gas-phase using the PBE functional[16] in conjunction with the D3 version of Grimme's dispersion correction with Becke-Johnson damping function,[17] using the resolution of identity approximation. The def2-SVP basis set was used for all atoms with matching auxiliary basis.[18] Analytic frequency calculations were performed to verify the nature of all stationary points (minima and transition states) and to calculate free energies and enthalpies at 213 K by using the rigid-rotor harmonic oscillator (RRHO) approximation. Solvation effect has been accounted by using CPCM (Chloroform) solvation model,[19] as implemented in ORCA. An exhaustive manual conformational search has been performed on possible catalyst substrate orientations. Transition state structures were verified by the presence of a single imaginary vibrational frequency. Single-point energies (gas phase) are calculated at B3LYP-D3(BJ)/def2-TZVP[20] and M06-2X/def2-TZVP[21] level respectively. All crucial non-covalent contacts between the substrate and the catalyst has been identified by AIM analysis.[22] Distortion-interaction study has been performed to qualitatively understand the reason behind stereoinduction.[23] Molecular structures were generated using CYLview program.[24]

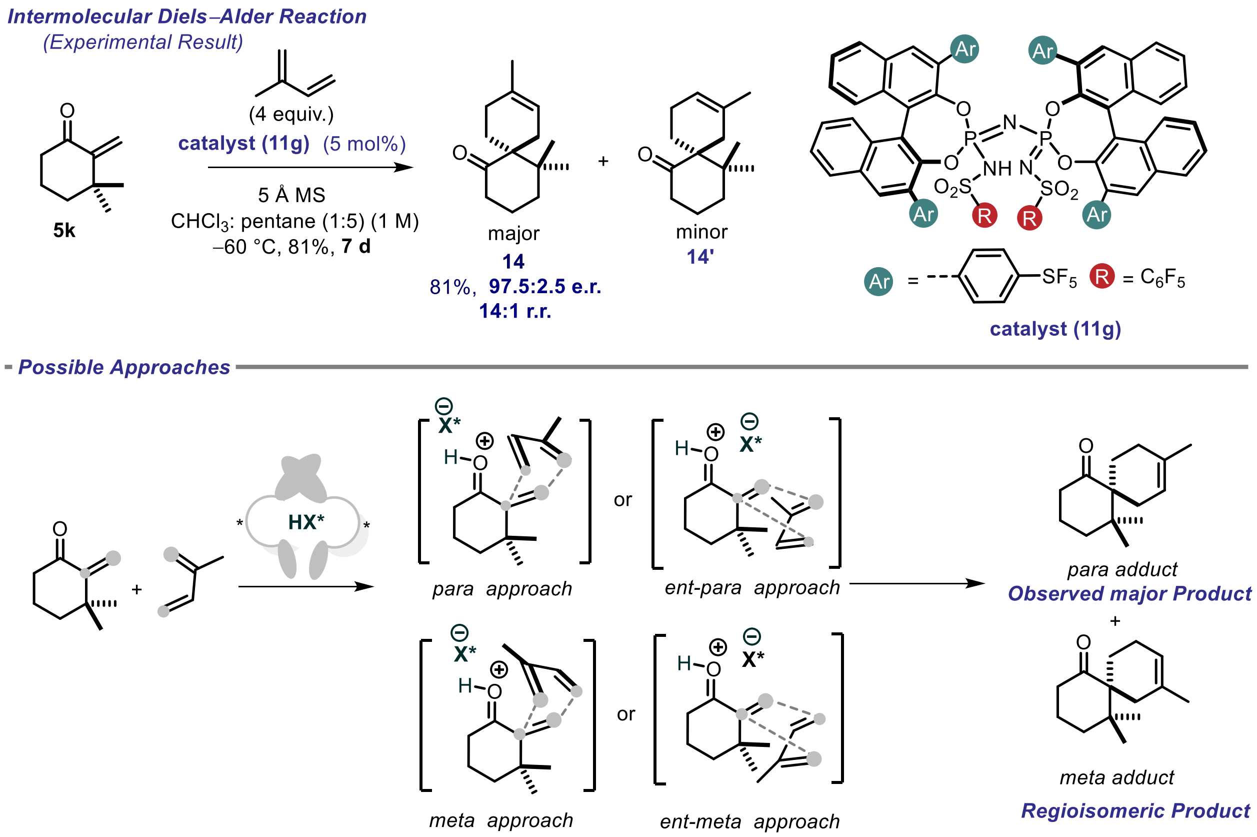

Figure S1. Experimental results and summary of the overall computational approaches undertaken for this work

2. Results and Discussion

Based on our mechanistic hypothesis and previous reports,[25] we anticipated the activation of the substrate by the catalyst is likely to happen *via* the carbonyl protonation within the stereoselectivity-determining cycloaddition step. Particular emphasis has been given to understand the preferred site of protonation of the catalyst (protonation via N atom of IDPi vs O atom of IDPi). Other than computing enantioselectivity, we have also conducted TS search leading to the regioisomers to understand the observed high regioselectivity (14:1 r.r.).

Our computational studies identified that these reactions follow a highly asynchronous concerted TS geometry where the bond formation towards the exo-methylene carbon proceeds earlier than the carbon at the tertiary centre. Computed TS structures shows the catalyst present as a chiral counterion after transferring the proton to the carbonyl of the enone.

Notably, protonation prefers from the N centre of the IDPi catalyst in all cases (See Fig **S4**). Gibbs Free energy difference at the *B3LYP-D3(BJ)/def2-TZVP + CPCM(chloroform)//PBE-D3/def2-SVP level of theory* has been found to be 2.95 kcal/mol (e.r. 99:1), providing a good agreement with the experiment (97.5:2.5 e.r.). Furthermore, the Gibbs Free energy difference of 1.2 kcal/mol at this level between the two major regio-isomeric TS [TS-A1 and TS-C1: (**Table S3**)] provides great agreement with the experimentally observed high regioselectivity (14:1 r.r.). Similar qualitative agreement was also observed by using *M06-2X/def2-TZVP + CPCM (chloroform)//PBE-D3/def2-SVP level of theory* (**Table S4**).

Several non-covalent contacts (e.g. CH..N, CH..O, CH..F) have been identified within the stereo determining TS structures by the AIM analysis (Fig **S6**). In order to understand the reason behind the stereoinduction, Distortion-Interaction (DI) analysis has been performed. DI analysis reveals that TS leading to the minor isomer is significantly more distorted than its counterparts (**Table S5**). The origin of distortion predominantly stems from the difficulty in substrate binding within the catalyst cavity ($\Delta\Delta$ $E^{\neq}$= 1.12 kcal/mol) (**Table S5**). A similar high reorganization energy is also evident in the major regioisomeric TS ($\Delta\Delta$ $E^{\neq}$= 4.36 kcal/mol) (**Table S7**).

In order to further probe the origin of stereoinduction, we zoomed into the active site of the catalyst for both stereodeterming TS structures (Fig **S7**). We observed that for the major stereoisomer (**TS-A1**), one of the perflourinated phenyl core has been distorted to create a bigger cavity (7.36 Å) which can easily accommodate the bulky methyl group of the isoprene. Notably, such displaced stacking arrangement is preferred compared to TS with retained stacking [e.g. **TS-A4** contains stacking and 5.87 kcal higher in energy compared to **TS-A1**, **Fig S5**], due to minimization of steric interaction of the overall TS arrangement (**Table S6**). In contrast, π–π interaction in TS_{minor} (**TS-B1**) is almost intact leading to a much smaller catalyst cavity (5.45 Å). With such small cavity, Methyl group of the incoming isoprene moiety is now placed in a more sterically congested environment, destabilizing the overall TS structure. Taken together, this highlights the power the confined small molecular catalysts to perform enzyme like action emulating an induced fit type mechanism.

Finally, the catalytic cycle of this reaction has also computed by using *B3LYP-D3(BJ)/def2-TZVP + CPCM(chloroform)//PBE-D3/def2-SVP level of theory*. Consistent with the experimental condition, computed activation barrier for this reaction is low and the reaction is exothermic in nature (**Table S8** and **Fig S8**).

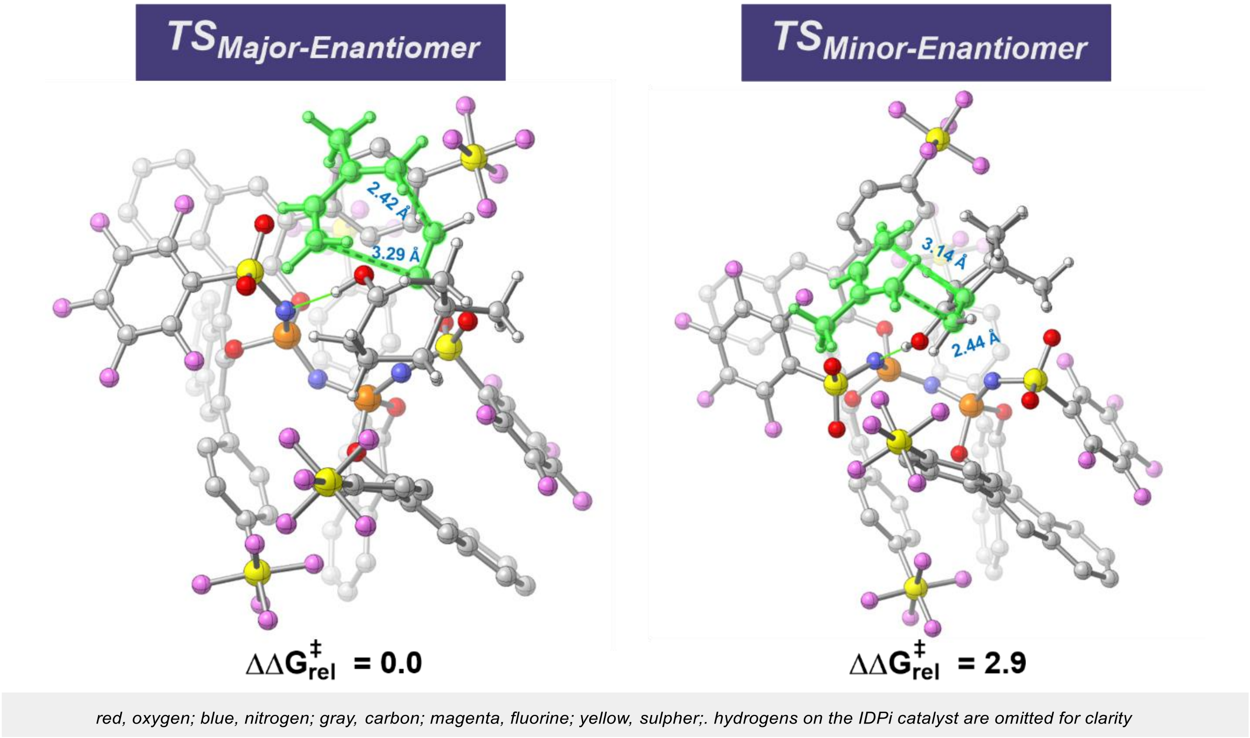

Figure S2. Stereodetermining TSs computed at the *B3LYP-D3(BJ)/def2-TZVP+CPCM(chloroform)//PBE-D3/def2-SVP level of theory. Relative free energy difference has shown in kcal/mol.* Predicted stereoselectivity (99:1 e.r.) is in good agreement with the experimentally observed high selectivity (97.5:2.5 e.r.).

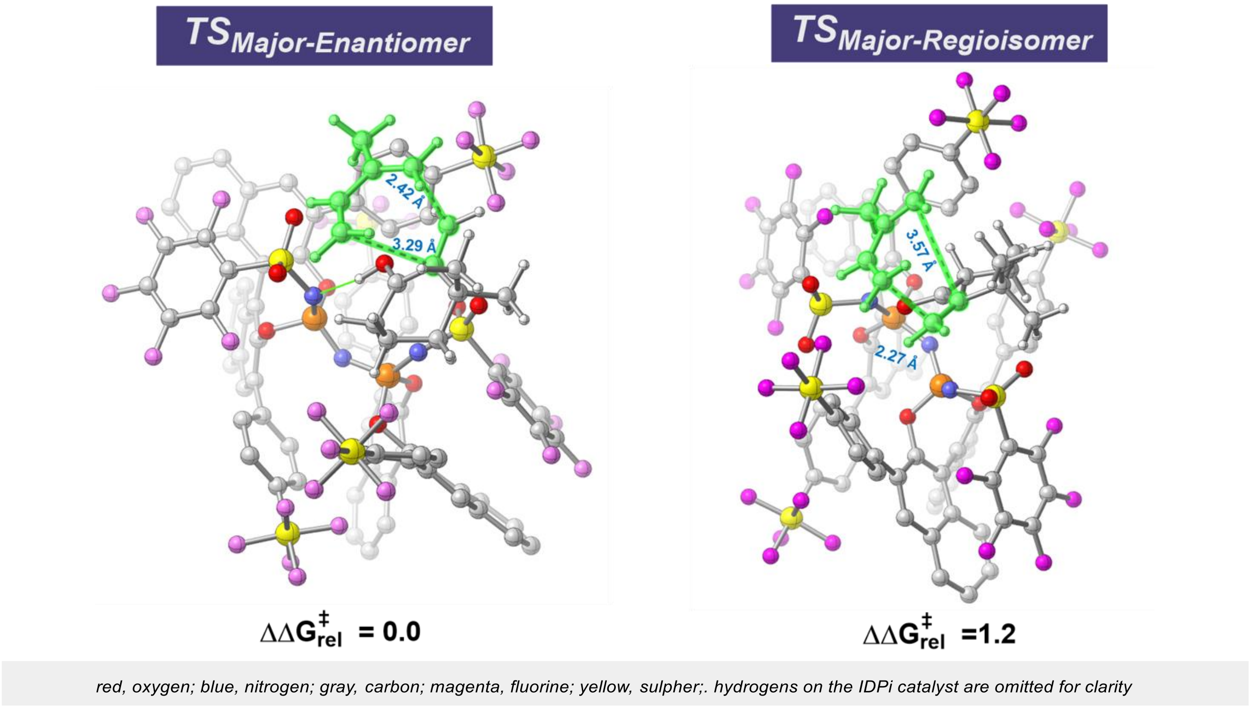

Figure S3. Comparison between the two major regio-isomeric TS at the *B3LYP-D3(BJ)/def2-TZVP + CPCM(chloroform)//PBE-D3/def2-SVP level of theory. Relative free energy difference has shown in kcal/mol.* Predicted regio-selectivity (17:1 r.r.) is in excellent agreement with the experimentally observed high regioselectivity (14:1 r.r.).

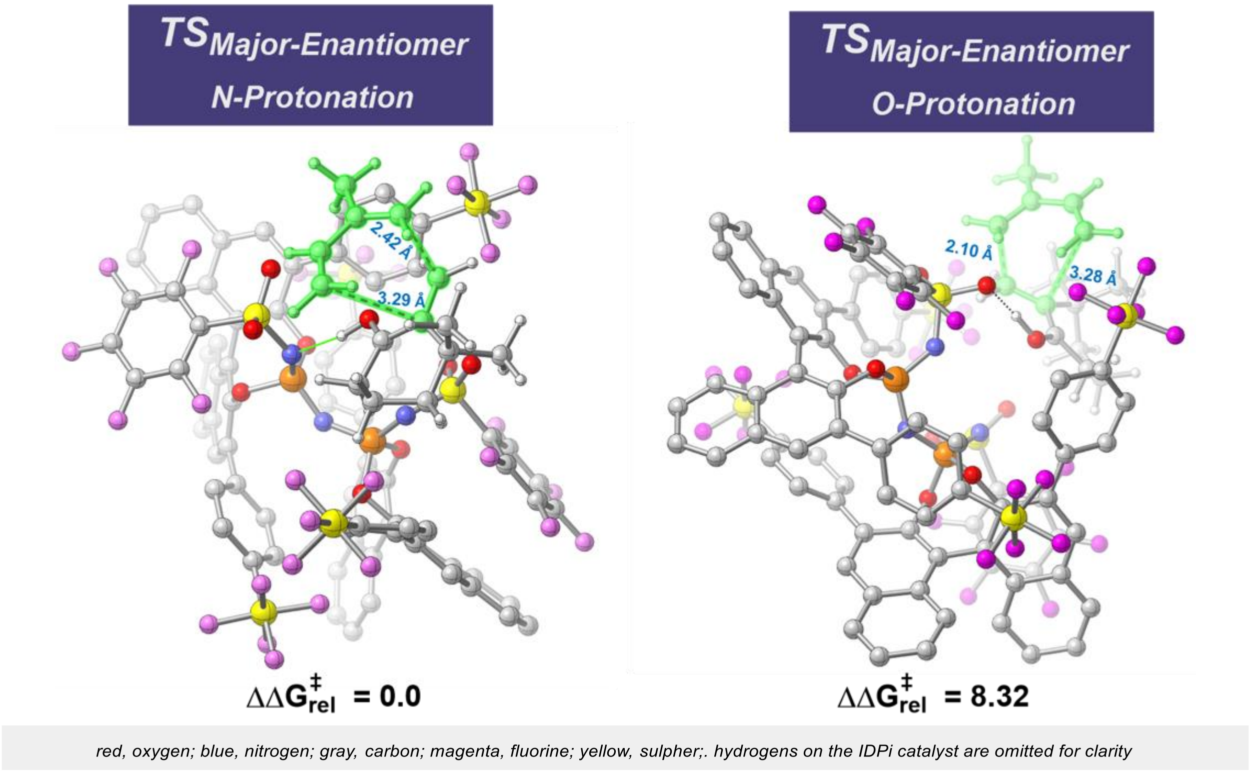

Figure S4. Understanding the preferred protonation site: Comparison of the N *vs* O protonation in TS leading to the Major isomer. Computed at the *B3LYP-D3(BJ)/def2-TZVP + CPCM(chloroform)//PBE-D3/def2-SVP level of theory. Relative free-energy difference has shown in kcal/mol.*

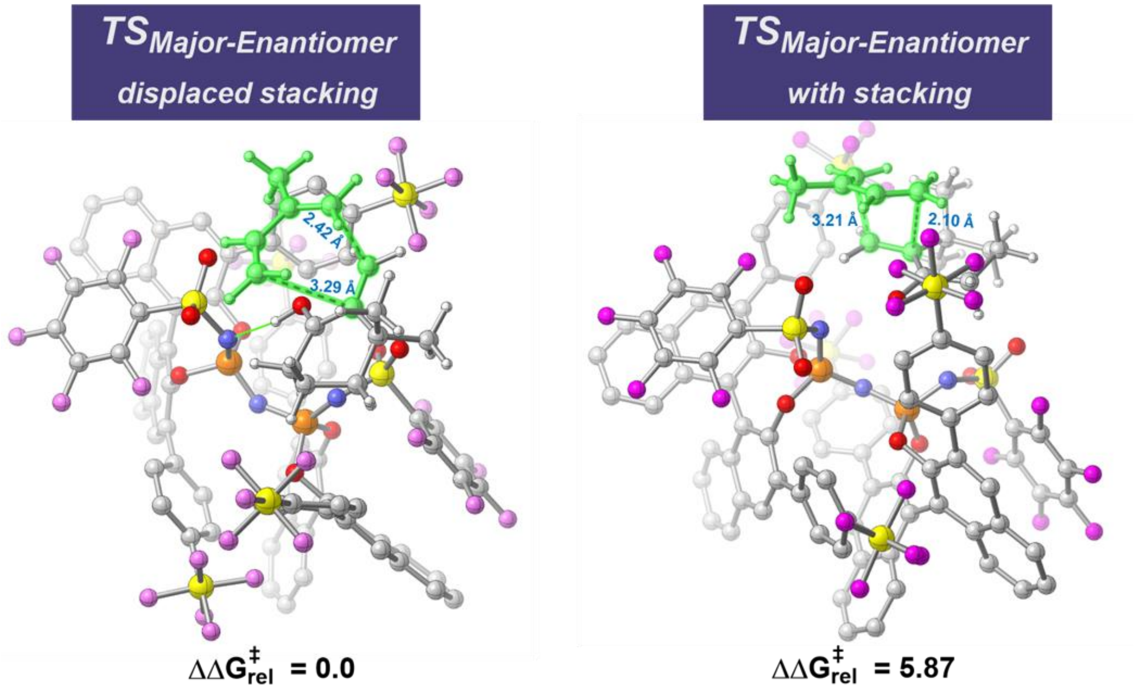

red, oxygen; blue, nitrogen; gray, carbon; magenta, fluorine; yellow, sulpher;. hydrogens on the IDPi catalyst are omitted for clarity

Figure S5. TS with displaced stacking/no stacking (**TS-A1**) and with stacking (**TS-A4**) leading to major stereoisomers at the *B3LYP-D3(BJ)/def2-TZVP + CPCM(chloroform)//PBE-D3/def2-SVP level of theory. Relative free-energy difference has shown in kcal/mol.*

Key non-covalent contacts identified by the AIM analysis for the TS $_{major}$. Electron density values at the bond critical point ($\rho_{\beta\chi\pi}$ X 10^{-2}) has provided within parenthesis.

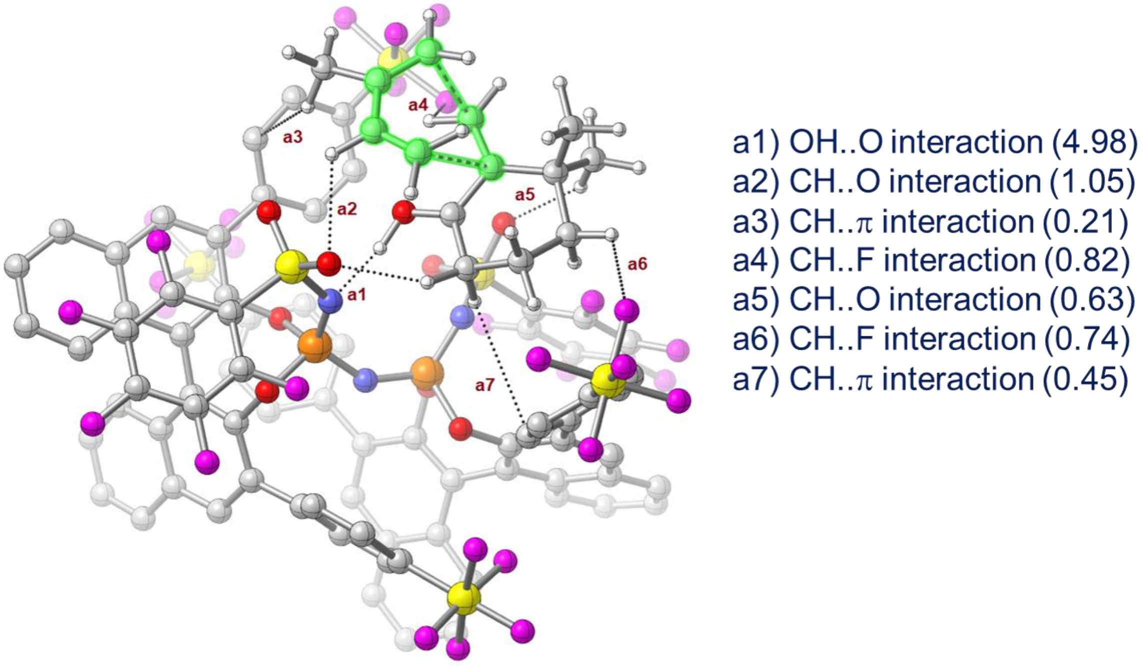

red, oxygen; blue, nitrogen; gray, carbon; magenta, fluorine; yellow, sulpher;. hydrogens on the IDPi catalyst are omitted for clarity

Figure S6. AIM analysis summary of the major stereo-determining Transition State

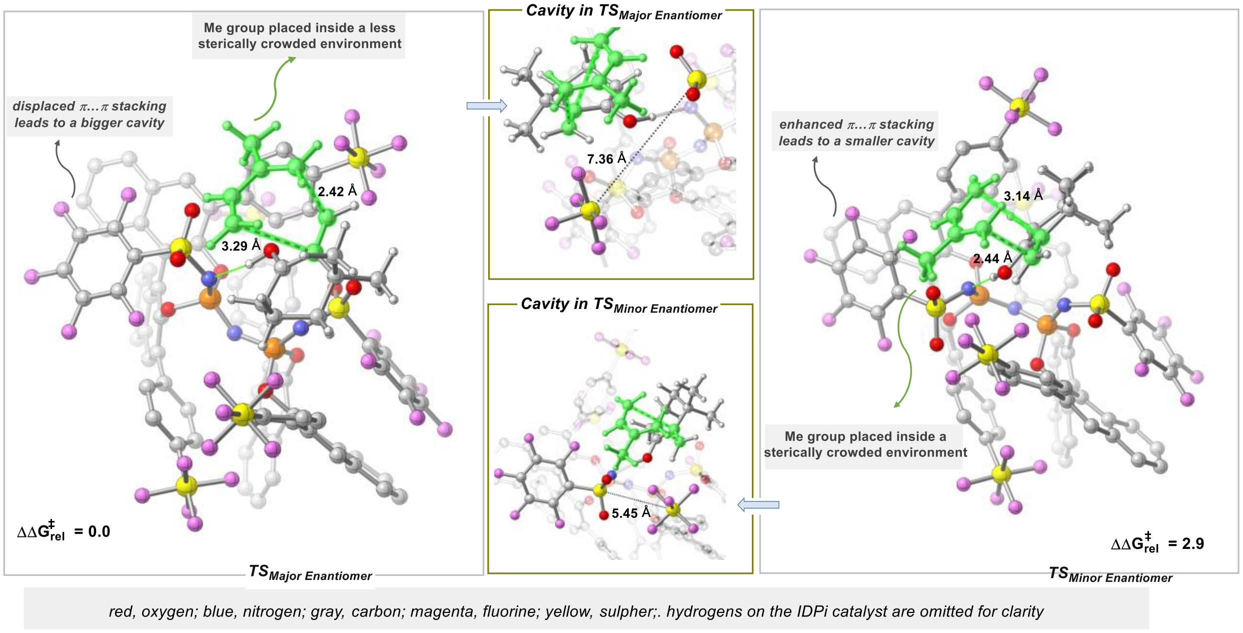

Figure S7. Understanding the reason behind the enantioinduction

Table S1. TS Major cycloaddition energetics at the *B3LYP-D3(BJ)/def2-TZVP- + CPCM(chloroform)//PBE-D3/def2-SVP level of theory*

TS No	PBE RRHO corrections	*B3LYP-D3/def2-TZVP* single point ($CHCl_3$ solv.)	*Imaginary Freq.*	ΔG(TS) Final	Relative TS Energy (kcal/mol)
Protonation via nitrogen atom of the IDPi Catalyst					
TS-A1	0.14230298	-10372.96499	-94.50	-10372.8227	0.0
TS-A2	0.14244350	-10372.96462	-98.26	-10372.8222	0.3
TS-A3	0.14205151	-10372.96262	-171.19	-10372.8206	1.35
TS-A4	0.14209322	-10372.95540	-189.50	-10372.8134	5.87
TS-A5	0.14410080	-10372.95249	-215.51	-10372.8083	9.05
Protonation via oxygen atom of the IDPi Catalyst					
TS-A6	0.141798	-10372.95123	-166.15	-10372.8095	8.32

Based on the optimized structures, predicted selectivity (*e.r.*) at the *B3LYP-D3/def2-TZVP-) + CPCM(chloroform)//PBE-D3/def2-SVP level of theory* at 213 K (*e.r.*) 99:1, provides a good agreement to the experimentally observed enantioselectivity (*e.r.*) 97.5:2.5.

Table S2. TS Minor cycloaddition energetics at the *B3LYP-D3(BJ)/def2-TZVP + CPCM(chloroform)//PBE-D3/def2-SVP level of theory*

TS No	PBE RRHO corrections	*B3LYP-D3/def2-TZVP* single point ($CHCl_3$ solv.)	*Imaginary Freq.*	Δ G(TS) Final	Relative TS Energy (kcal/mol)
Protonation via nitrogen atom of the IDPi Catalyst					
TS-B1	0.142989	-10372.960968	-97.97	-10372.8180	2.9
TS-B2	0.142280	-10372.96003	-154.14	-10372.8176	3.1
TS-B3	0.143122	-10372.95868	-160.23	-10372.8155	4.45
TS-B4	0.14223	-10372.95540	-154.06	-10372.8132	5.95
TS-B5	0.14254495	-10372.94962	-151.74	-10372.8071	9.75
Protonation via oxygen atom of the IDPi Catalyst					
TS-B6	0.14414010	-10372.95412	-165.17	-10372.81002	7.95

Table S3. TS energetics of some regio-isomeric cycloaddition TS structures at the *B3LYP-D3(BJ)/def2-TZVP+ CPCM(chloroform)//PBE-D3/def2-SVP level of theory*

TS No	PBE RRHO corrections	*B3LYP-D3/def2-TZVP* single point ($CHCl_3$ solv.)	*Imaginary Freq.*	Δ G(TS) Final	Relative TS Energy (kcal/mol)
TS-C1	0.13988827	-10372.96063280	-165.14	-10372.8208	1.2
TS-C2	0.14122974	-10372.9607793	-163.1	-10372.8195	1.95
TS-C3	0.14127733	-10372.9555690	-116.29	-10372.81436	5.29
TS-C4	0.14129309	-10372.9546743	104.48	-10372.8133	5.9
TS-C5	0.14129382	-10372.9536772	-107.58	-10372.8123	6.57
TS-C6	0.14257275	-10372.946219	-214.29	-10372.8037	11.97
TS-D1	0.14252530	-10372.948237	-124.51	-10372.8057	10.65
TS-D2	0.14383911	-10372.948869	-130.21	-10372.805	11.16
TS-D3	0.14304784	-10372.944894	-173.17	-10372.8017	13.2
TS-D4	0.14168287	-10372.941294	-110.19	-10372.7996	14.45
TS-D5	0.14301509	-10372.9338073	-187.98	-10372.7908	20.07
TS-D6	0.14515224	-10372.93379	-188.47	-10372.7886	21.42

The high free energy difference (1.2 kcal/mol) between the two major regio-isomeric TS (TS-A1 and TS-C1) also consistent with the experimentally observed high regioselectivity (14:1).

Table S4. Difference of Energetics of stereo determining TS structures at the *M06-2X/def2-TZVP + CPCM (chloroform)//PBE-D3/def2-SVP level of theory*.

TS No	PBE RRHO corrections	*M06-2X/def2-TZVP* single point ($CHCl_3$ solv.)	*Imaginary Freq.*	ΔG(TS) Final	Relative TS Energy (kcal/mol)
Protonation via nitrogen atom of the IDPi Catalyst					
TS-A1	0.14230298	-10373.521699	-94.50	-10373.3793	0.0
TS-B1	0.142989	-10373.51716	-97.97	-10373.3742	3.16

TS-C1	0.13988827	-10373.5151650	-165.14	-10373.3752	2.57

Based on the optimized structures, predicted selectivity (*e.r.*) at the *M06-2X/def2-TZVP- + CPCM (chloroform)//PBE-D3/def2-SVP level of theory* at 213 K (*e.r.*) 99:1, provides a good agreement to the observed enantioselectivity (*e.r.*) 97.5:2.5. Additionally, the free energy difference (2.57 kcal/mol) between the two major regio isomeric TS (TS-A1 and TS-C1) also consistent with the experimentally observed high regio selectivity (14:1).

Table S5. Distortion-Interaction analysis on the optimized TS structures to identify reason behind stereoinduction

TS No	*B3LYP-D3(BJ)/def2-TZVP* single point (gas phase)	Relative Energy (kcal/mol) ΔΔE
TS-A1	-10372.931711244	1.88
TS-B1	-10372.928750531854	
Substrates Only		
Subst_TS-A1	-622.147459741858	1.12
Subst_TS-B1	-622.145695798825	
Catalyst Only		
Cat_TS-A1	-9750.615849100692	0.43
Cat_TS-B1	-9750.615170260860	
Total Distortion	-10373.51848	1.6

Table S6. Distortion-Interaction Analysis to understand the conformational change in major isomer

TS No	*B3LYP-D3(BJ)/def2-TZVP* single point (gas phase)	Relative Energy (kcal/mol) ΔΔE
TS-A1	-10372.931711244	1.75
TS-A4	-10372.9289256	
Substrates Only		
Subst_TS-A1	-622.147459741858	3.82
Subst_TS-A4	-622.139694228335	
Catalyst Only		
Cat_TS-A1	-9750.615849100692	- 2.1
Cat_TS-A4	-9750.619398212968	
Total Distortion	-10373.51848	1.7

Table S7. Distortion-Interaction Analysis to understand the regioisomeric preference.

TS No	*B3LYP-D3(BJ)/def2-TZVP* single point (gas phase)	Relative Energy (kcal/mol) ΔΔE
TS-A1	-10372.931711244	2.63
TS-C1	-10372.927574375	
Substrates Only		
Subst_TS-A1	-622.147459741858	4.36
Subst_TS-C1	-622.140496663962	
Catalyst Only		
Cat_TS-A1	-9750.615849100692	-0.31

Cat_TS-C1	-9750.616344948097	
Total Distortion	-10373.51848	4.06

Table S8. Reaction profile at the *B3LYP-D3(BJ)/def2-TZVP + CPCM(chloroform)//PBE-D3/def2-SVP level of theory*

	ΔG(TS) Final ($CHCl_3$ solv)	Relative Free Energy (ΔΔG) (kcal/mol)
Diene+Dienophile+catalyst	-10372.8164	0.0
Dienophile complex+ Diene	-10372.8341	-11.1
Pre Rxn complex	-10372.8308	-9.05
Cycloaddition TS	-10372.8227	-3.95
Product complex	-10372.8499	-21.02
Pdt + Catalyst	-10372.8438	-17.25

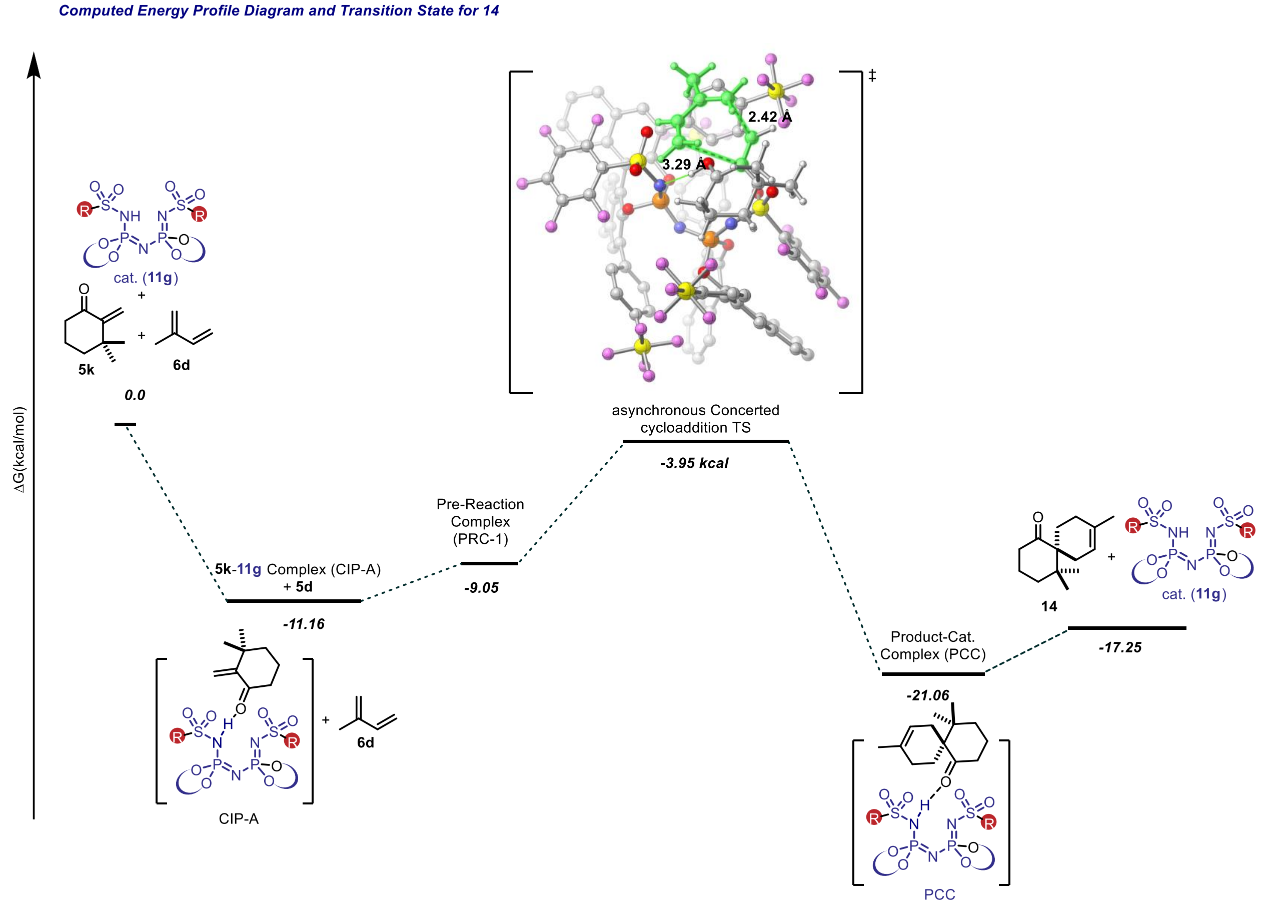

Figure S8. Computed energy profile diagram of the enantioselective cycloaddition reaction at *B3LYP-D3(BJ)/def2-TZVP + CPCM(chloroform)//PBE-D3/def2-SVP level of theory*

4. <u>Optimized Cartesian Coordinates PBE-D3/def2-SVP</u>

Major ISOMER:

TS-A1: Imaginary frequency = - 94.50

15	0.320344000	-1.179090000	-1.626699000
8	1.951526000	-0.887171000	-1.551453000
8	0.195086000	-2.655190000	-0.882249000
6	2.823731000	-1.909263000	-1.914353000
6	3.595384000	-1.774557000	-3.115298000
6	4.454313000	-2.823915000	-3.442285000
6	4.534067000	-4.016380000	-2.678567000
6	5.357902000	-5.106619000	-3.086323000
6	5.377811000	-6.287556000	-2.362259000
6	4.560429000	-6.425952000	-1.209787000
6	3.769279000	-5.372861000	-0.772380000
6	3.748072000	-4.134679000	-1.478587000
6	2.943536000	-3.014854000	-1.065590000
6	2.248551000	-3.040498000	0.249255000
6	2.982740000	-3.197700000	1.475887000
6	4.408510000	-3.192787000	1.529564000
6	5.074283000	-3.319757000	2.740683000
6	4.348665000	-3.470525000	3.952480000
6	2.962858000	-3.461802000	3.935103000
6	2.246128000	-3.306538000	2.710718000
6	0.826894000	-3.223571000	2.688611000
6	0.106875000	-2.988983000	1.516468000
6	0.863106000	-2.890499000	0.305545000
6	3.604763000	-0.561241000	-3.968091000
1	5.104245000	-2.727512000	-4.323650000
1	5.956412000	-4.999526000	-4.003646000
1	6.002906000	-7.129455000	-2.694359000
1	4.548117000	-7.379393000	-0.661065000
1	3.134486000	-5.494921000	0.115848000
1	4.978237000	-3.056093000	0.600108000
1	6.173957000	-3.294772000	2.761639000
1	4.889311000	-3.579580000	4.904427000
1	2.390211000	-3.557611000	4.870707000
1	0.282499000	-3.346507000	3.636817000
6	-1.370096000	-2.849094000	1.552658000
6	3.550733000	0.736382000	-3.415252000
6	3.786820000	1.866714000	-4.205553000
6	4.050155000	1.697413000	-5.570276000
6	4.047314000	0.429899000	-6.166196000
6	3.819845000	-0.689166000	-5.360396000
6	-1.971753000	-2.233162000	2.672113000
6	-3.359913000	-2.131732000	2.788316000
6	-4.160561000	-2.633449000	1.758285000
6	-3.603222000	-3.239568000	0.626035000
6	-2.209617000	-3.347104000	0.528903000
1	3.375227000	0.865814000	-2.342919000
1	3.797361000	-1.685941000	-5.822703000
1	-1.346025000	-1.796567000	3.463128000
1	-1.786571000	-3.820038000	-0.365452000
7	-0.285499000	-0.032126000	-0.719495000
15	-1.465274000	1.011992000	-0.650963000
8	-1.155299000	2.067756000	0.578702000
8	-2.775726000	0.200572000	-0.033127000
6	-1.243311000	1.616379000	1.891880000
6	-0.026065000	1.339130000	2.585941000
6	-0.120912000	0.945601000	3.917180000
6	-1.373449000	0.782320000	4.568037000

6	-1.440188000	0.351311000	5.927202000
6	-2.659239000	0.108383000	6.538564000
6	-3.860580000	0.273550000	5.800097000
6	-3.829916000	0.704758000	4.481379000
6	-2.593833000	0.998763000	3.829493000
6	-2.508724000	1.446648000	2.458498000
6	-3.731029000	1.631391000	1.630303000
6	-4.806916000	2.473735000	2.075602000
6	-4.684243000	3.373692000	3.174760000
6	-5.760033000	4.149561000	3.580060000
6	-7.009054000	4.058519000	2.910441000
6	-7.151160000	3.210327000	1.824429000
6	-6.057463000	2.416091000	1.368354000
6	-6.168476000	1.596309000	0.218160000
6	-5.095964000	0.887690000	-0.325983000
6	-3.870495000	0.932499000	0.420833000
6	1.299377000	1.391645000	1.919574000
1	0.802189000	0.747866000	4.483189000
1	-0.497376000	0.204134000	6.476612000
1	-2.698588000	-0.227182000	7.585594000
1	-4.828471000	0.045154000	6.270801000
1	-4.768191000	0.790388000	3.920708000
1	-3.719568000	3.455657000	3.694738000
1	-5.640712000	4.850163000	4.419831000
1	-7.856322000	4.674912000	3.245806000
1	-8.107253000	3.149250000	1.282151000
1	-7.153589000	1.535284000	-0.263411000
6	-5.287217000	0.128115000	-1.591712000
6	1.755223000	2.521102000	1.208467000
6	3.034756000	2.540307000	0.639274000
6	3.854774000	1.412612000	0.779013000
6	3.425710000	0.269772000	1.460763000
6	2.148102000	0.270296000	2.027281000
6	-6.356560000	0.484101000	-2.449890000
6	-6.662253000	-0.248614000	-3.598318000
6	-5.855829000	-1.342797000	-3.933451000
6	-4.761455000	-1.704796000	-3.145401000
6	-4.492403000	-0.975573000	-1.979669000
1	1.119066000	3.408937000	1.109747000
1	1.792184000	-0.633172000	2.542172000
1	-6.963327000	1.372731000	-2.231739000
1	-3.648562000	-1.304932000	-1.366142000
7	-0.155729000	-1.366256000	-3.144162000
7	-1.756750000	1.867746000	-1.981499000
16	-1.005701000	-2.646844000	-3.683906000
8	-2.038684000	-3.108158000	-2.722616000
8	-1.419179000	-2.370168000	-5.072439000
16	-2.586176000	3.261653000	-2.295428000
8	-1.815105000	3.978445000	-3.337133000
8	-4.013325000	2.973396000	-2.552778000
1	4.218786000	0.314954000	-7.243465000
1	3.787108000	2.864417000	-3.749879000
1	4.068601000	-0.613770000	1.552271000
1	3.385583000	3.429959000	0.101852000
1	-7.510878000	0.041510000	-4.228930000
1	-4.098792000	-2.538347000	-3.408986000
1	-4.237653000	-3.629702000	-0.179730000

1	-3.804203000	-1.655267000	3.668938000
1	-2.094361000	1.071528000	-3.470730000
16	-5.973767000	-2.474272000	1.909463000
16	-6.236445000	-2.306088000	-5.435813000
16	4.377929000	3.164098000	-6.607897000
16	5.531336000	1.419557000	0.054449000
9	3.139961000	4.008451000	-5.925587000
9	3.358072000	2.635637000	-7.791845000
9	4.669932000	4.473979000	-7.538936000
9	5.411558000	3.792038000	-5.502037000
9	5.630325000	2.422729000	-7.366021000
9	5.241271000	0.013706000	-0.765110000
9	5.035443000	2.289380000	-1.257664000
9	5.925211000	2.814407000	0.818218000
9	6.155211000	0.555377000	1.302518000
9	7.024774000	1.418576000	-0.605791000
9	-6.240174000	-3.721295000	-4.616379000
9	-7.855990000	-2.157629000	-5.204397000
9	-6.582982000	-3.150054000	-6.794524000
9	-6.261243000	-0.935333000	-6.377926000
9	-4.647693000	-2.517362000	-5.799565000
9	-6.211226000	-4.044990000	1.509321000
9	-6.140747000	-2.046094000	0.332518000
9	-5.941969000	-2.880523000	3.501781000
9	-5.861198000	-0.877314000	2.325729000
9	-7.596186000	-2.322015000	2.051645000
6	-4.616190000	2.504777000	-6.476995000
6	-3.556576000	3.363556000	-5.964830000
6	-2.370377000	3.631893000	-6.565279000
6	-4.493505000	1.789933000	-7.646848000
1	-2.134682000	3.292753000	-7.584079000
1	-3.682206000	1.990735000	-8.358432000
1	-5.322711000	1.157568000	-7.996864000
1	-1.618698000	4.242327000	-6.043409000
6	-0.792278000	0.157789000	-7.704527000
6	0.608629000	0.484784000	-7.128370000
6	0.607122000	1.715989000	-6.229832000
6	-0.216982000	1.418746000	-4.985487000
1	0.963479000	-0.382943000	-6.533058000
1	1.321508000	0.607921000	-7.969437000
1	1.630206000	1.995877000	-5.936312000
1	0.195277000	2.596008000	-6.766290000
1	0.291148000	0.650736000	-4.354089000
1	-0.372426000	2.301672000	-4.330450000
6	-1.878650000	0.343246000	-6.620940000
6	-3.187999000	-0.061282000	-6.818064000
1	-3.491417000	-0.587387000	-7.733109000
1	-3.875395000	-0.128952000	-5.970560000
6	-1.547483000	0.838357000	-5.315419000
8	-2.435342000	0.715567000	-4.364179000
6	-1.060535000	1.042642000	-8.939186000
1	-0.332459000	0.799370000	-9.739375000
1	-2.074462000	0.871305000	-9.353614000
1	-0.955093000	2.121724000	-8.713209000
6	-0.793104000	-1.321371000	-8.153352000
1	-0.697671000	-1.993993000	-7.280344000
1	-1.722653000	-1.587393000	-8.696049000

1	0.056558000	-1.500736000	-8.844198000
6	0.249373000	-3.973617000	-3.783652000
6	1.279093000	-3.842540000	-4.733968000
6	0.228285000	-5.125892000	-2.975846000
6	2.251767000	-4.836874000	-4.898716000
6	1.204748000	-6.127441000	-3.130456000
6	2.194268000	-6.000989000	-4.116212000
9	-0.684417000	-5.324477000	-2.024378000
9	1.186406000	-7.208396000	-2.346975000
9	3.095833000	-6.966192000	-4.285815000
9	3.244016000	-4.672925000	-5.782698000
9	1.381749000	-2.745393000	-5.493515000
6	-2.482877000	4.303336000	-0.816085000
6	-1.209789000	4.701481000	-0.367945000
6	-3.624326000	4.796173000	-0.156408000
6	-1.066257000	5.509871000	0.765476000
6	-3.486463000	5.642039000	0.959941000
6	-2.210028000	5.987144000	1.429332000
9	0.152030000	5.819760000	1.223783000
9	-2.081782000	6.767319000	2.499820000
9	-4.568429000	6.132032000	1.565675000
9	-4.861347000	4.495399000	-0.536634000
9	-0.103940000	4.260480000	-0.969532000
6	-5.819743000	2.354944000	-5.592096000
1	-5.506253000	2.060519000	-4.569878000
1	-6.338181000	3.332124000	-5.486295000
1	-6.535135000	1.610137000	-5.983808000
1	-3.718117000	3.767848000	-4.953547000

TS-A2: Imaginary frequency =   - 98.26

15	0.229347000	-1.034729000	-1.704750000
8	1.861922000	-0.747069000	-1.645867000
8	0.113081000	-2.530410000	-0.998921000
6	2.726833000	-1.759908000	-2.049761000
6	3.480084000	-1.593307000	-3.258323000
6	4.332628000	-2.634072000	-3.626840000
6	4.422649000	-3.846872000	-2.897038000
6	5.239230000	-4.926100000	-3.346542000
6	5.269138000	-6.126143000	-2.654990000
6	4.469073000	-6.295297000	-1.494491000
6	3.685390000	-5.254113000	-1.016993000
6	3.654731000	-3.997207000	-1.688964000
6	2.857898000	-2.888432000	-1.233420000
6	2.182730000	-2.949005000	0.090585000
6	2.934968000	-3.140749000	1.301284000
6	4.361399000	-3.139414000	1.333633000
6	5.045035000	-3.300252000	2.530692000
6	4.337392000	-3.482876000	3.748732000
6	2.951498000	-3.471505000	3.752464000
6	2.216776000	-3.281838000	2.543686000
6	0.797556000	-3.195566000	2.545350000
6	0.060526000	-2.927923000	1.391026000
6	0.798580000	-2.798439000	0.171870000
6	3.477606000	-0.357294000	-4.077940000
1	4.969034000	-2.514240000	-4.515164000
1	5.823992000	-4.794501000	-4.269528000
1	5.888494000	-6.959005000	-3.019078000
1	4.464255000	-7.263200000	-0.971577000

1	3.063911000	-5.399854000	-0.122940000
1	4.917364000	-2.978199000	0.399793000
1	6.144944000	-3.277407000	2.535772000
1	4.892055000	-3.618606000	4.689117000
1	2.392773000	-3.591767000	4.693647000
1	0.267217000	-3.343028000	3.498015000
6	-1.415390000	-2.786187000	1.453264000
6	3.433669000	0.924957000	-3.489437000
6	3.658650000	2.076117000	-4.252465000
6	3.900684000	1.943608000	-5.625167000
6	3.887280000	0.692662000	-6.255015000
6	3.670930000	-0.447629000	-5.476382000
6	-1.998721000	-2.198794000	2.597464000
6	-3.384766000	-2.098270000	2.737439000
6	-4.201982000	-2.571595000	1.706987000
6	-3.663103000	-3.148391000	0.550726000
6	-2.271361000	-3.255649000	0.429659000
1	3.274979000	1.025514000	-2.411409000
1	3.640139000	-1.431499000	-5.965117000
1	-1.360149000	-1.783980000	3.389940000
1	-1.862910000	-3.705489000	-0.483171000
7	-0.361099000	0.087334000	-0.757282000
15	-1.536425000	1.132138000	-0.641759000
8	-1.204743000	2.153284000	0.611283000
8	-2.839357000	0.307113000	-0.026179000
6	-1.273915000	1.666659000	1.912956000
6	-0.046941000	1.368081000	2.580579000
6	-0.122553000	0.938753000	3.901938000
6	-1.365453000	0.760506000	4.567148000
6	-1.412578000	0.292509000	5.914851000
6	-2.622764000	0.035438000	6.537910000
6	-3.834790000	0.223503000	5.822774000
6	-3.823120000	0.690714000	4.516070000
6	-2.596391000	0.999720000	3.853538000
6	-2.531020000	1.484482000	2.494007000
6	-3.765368000	1.694284000	1.689904000
6	-4.832047000	2.526676000	2.174281000
6	-4.690192000	3.396318000	3.295317000
6	-5.757634000	4.163291000	3.737921000
6	-7.017032000	4.093056000	3.085463000
6	-7.177969000	3.274862000	1.979249000
6	-6.093413000	2.490986000	1.485026000
6	-6.224108000	1.703112000	0.314761000
6	-5.161856000	1.007296000	-0.264902000
6	-3.925114000	1.028860000	0.464168000
6	1.268398000	1.436270000	1.895932000
1	0.808527000	0.723822000	4.448264000
1	-0.461881000	0.128302000	6.445528000
1	-2.646991000	-0.328716000	7.575806000
1	-4.795980000	-0.015599000	6.301817000
1	-4.769630000	0.794021000	3.972474000
1	-3.717460000	3.462234000	3.802419000
1	-5.623630000	4.840717000	4.594329000
1	-7.857477000	4.701944000	3.450404000
1	-8.142418000	3.230533000	1.450299000
1	-7.216635000	1.657343000	-0.153089000
6	-5.374385000	0.283197000	-1.547955000

6	1.715637000	2.583463000	1.208134000
6	2.986310000	2.615673000	0.620027000
6	3.806108000	1.483319000	0.717095000
6	3.385329000	0.323637000	1.375152000
6	2.116578000	0.311269000	1.961107000
6	-6.455453000	0.665673000	-2.379637000
6	-6.781225000	-0.034596000	-3.542683000
6	-5.983612000	-1.121497000	-3.920001000
6	-4.878324000	-1.507885000	-3.159180000
6	-4.589153000	-0.811622000	-1.978177000
1	1.079911000	3.474670000	1.142748000
1	1.766904000	-0.604854000	2.457582000
1	-7.055901000	1.549702000	-2.127927000
1	-3.737090000	-1.160044000	-1.386918000
7	-0.268844000	-1.179956000	-3.219726000
7	-1.846043000	2.024438000	-1.943790000
16	-1.131845000	-2.442946000	-3.779994000
8	-2.151668000	-2.926240000	-2.815462000
8	-1.565512000	-2.128360000	-5.154211000
16	-2.675860000	3.428387000	-2.207043000
8	-1.918721000	4.171090000	-3.240800000
8	-4.107737000	3.151140000	-2.449869000
1	4.041902000	0.606802000	-7.337535000
1	3.666942000	3.061146000	-3.770056000
1	4.027676000	-0.563097000	1.433584000
1	3.330563000	3.518713000	0.101009000
1	-7.638586000	0.274975000	-4.151882000
1	-4.222309000	-2.335601000	-3.455702000
1	-4.310440000	-3.516096000	-0.255319000
1	-3.814725000	-1.644509000	3.636953000
1	-2.208190000	1.269064000	-3.448083000
16	-6.012363000	-2.414107000	1.890075000
16	-6.391079000	-2.042947000	-5.441486000
16	4.214039000	3.437544000	-6.627795000
16	5.470903000	1.506460000	-0.033791000
9	2.986380000	4.263286000	-5.905237000
9	3.176987000	2.940947000	-7.810614000
9	4.492976000	4.771824000	-7.527604000
9	5.263806000	4.035738000	-5.520675000
9	5.455068000	2.716699000	-7.423415000
9	5.163415000	0.125234000	-0.888130000
9	4.956811000	2.414515000	-1.312595000
9	5.881204000	2.877918000	0.762973000
9	6.112193000	0.605417000	1.178905000
9	6.953542000	1.520458000	-0.717810000
9	-6.385155000	-3.479763000	-4.660565000
9	-8.006239000	-1.897350000	-5.179756000
9	-6.761707000	-2.849243000	-6.816658000
9	-6.427824000	-0.647248000	-6.345842000
9	-4.808950000	-2.247698000	-5.836592000
9	-6.258589000	-3.973254000	1.451737000
9	-6.202568000	-1.943588000	0.327901000
9	-5.957024000	-2.862907000	3.470299000
9	-5.890811000	-0.829036000	2.347240000
9	-7.632178000	-2.263661000	2.060989000
6	-4.771743000	2.792467000	-6.374397000
6	-3.702360000	3.634951000	-5.855992000

6	-2.525364000	3.917842000	-6.467896000
6	-4.667819000	2.107299000	-7.563937000
1	-2.306682000	3.607065000	-7.499472000
1	-3.866834000	2.325237000	-8.282128000
1	-5.504666000	1.487741000	-7.918641000
1	-1.764081000	4.512230000	-5.941447000
6	-0.980627000	0.466766000	-7.726481000
6	0.430885000	0.774707000	-7.165723000
6	0.448356000	1.981513000	-6.234745000
6	-0.355398000	1.653344000	-4.984852000
1	0.793082000	-0.109451000	-6.599765000
1	1.129853000	0.918408000	-8.015182000
1	1.477095000	2.250817000	-5.951412000
1	0.030202000	2.876755000	-6.740294000
1	0.161682000	0.868724000	-4.381923000
1	-0.498305000	2.519516000	-4.305049000
6	-2.047945000	0.627012000	-6.620180000
6	-3.362750000	0.234029000	-6.806429000
1	-3.683067000	-0.267491000	-7.729481000
1	-4.035616000	0.145201000	-5.949222000
6	-1.692786000	1.084753000	-5.307634000
8	-2.564785000	0.938091000	-4.344982000
6	-1.266508000	1.384279000	-8.932940000
1	-0.550808000	1.161875000	-9.750182000
1	-2.286695000	1.224773000	-9.336588000
1	-1.156464000	2.456801000	-8.679717000
6	-0.993845000	-1.000168000	-8.213652000
1	-0.884983000	-1.695691000	-7.360377000
1	-1.933683000	-1.249545000	-8.746459000
1	-0.157123000	-1.163587000	-8.923984000
6	0.117556000	-3.769933000	-3.933516000
6	1.134360000	-3.615670000	-4.894199000
6	0.105106000	-4.943095000	-3.156205000
6	2.102806000	-4.606845000	-5.098245000
6	1.077712000	-5.941549000	-3.349991000
6	2.053992000	-5.790814000	-4.345490000
9	-0.795271000	-5.165334000	-2.198231000
9	1.068069000	-7.042599000	-2.594859000
9	2.951471000	-6.752527000	-4.552362000
9	3.082803000	-4.421551000	-5.991606000
9	1.228570000	-2.499252000	-5.626221000
6	-2.546444000	4.429427000	-0.701870000
6	-1.265330000	4.812085000	-0.263199000
6	-3.675969000	4.907104000	-0.011239000
6	-1.101699000	5.589372000	0.889093000
6	-3.518168000	5.722141000	1.125233000
6	-2.233534000	6.051386000	1.583505000
9	0.124512000	5.883855000	1.336299000
9	-2.086244000	6.802242000	2.672355000
9	-4.589073000	6.198244000	1.760948000
9	-4.919683000	4.619834000	-0.379888000
9	-0.170326000	4.384551000	-0.893681000
6	-5.962652000	2.622551000	-5.476166000
1	-5.635036000	2.302028000	-4.466293000
1	-6.477520000	3.597651000	-5.338465000
1	-6.685187000	1.889026000	-5.875962000
1	-3.846929000	4.011748000	-4.831616000

TS-A3: Imaginary frequency = - 171.32

15	0.374619000	-1.373373000	-1.336388000
8	1.982462000	-1.156837000	-1.018087000
8	0.194283000	-2.963350000	-0.931709000
6	2.902360000	-2.057351000	-1.529401000
6	3.776709000	-1.609464000	-2.572489000
6	4.725983000	-2.526464000	-3.025220000
6	4.782786000	-3.864341000	-2.553717000
6	5.712941000	-4.801013000	-3.095837000
6	5.722063000	-6.119396000	-2.672112000
6	4.782589000	-6.559089000	-1.701834000
6	3.877147000	-5.669853000	-1.141776000
6	3.867300000	-4.297510000	-1.528910000
6	2.965759000	-3.334366000	-0.957618000
6	2.149217000	-3.648200000	0.250378000
6	2.760910000	-4.085111000	1.481447000
6	4.173189000	-4.205740000	1.648054000
6	4.719220000	-4.592036000	2.864012000
6	3.882900000	-4.889862000	3.971921000
6	2.508426000	-4.761721000	3.850893000
6	1.914829000	-4.338500000	2.623905000
6	0.514554000	-4.104223000	2.533039000
6	-0.081142000	-3.618688000	1.371768000
6	0.770799000	-3.424700000	0.239840000
6	3.711681000	-0.241307000	-3.151433000
1	5.462513000	-2.215372000	-3.779753000
1	6.411177000	-4.457179000	-3.874017000
1	6.436056000	-6.834860000	-3.105987000
1	4.764512000	-7.616837000	-1.399955000
1	3.146259000	-6.020916000	-0.400221000
1	4.836089000	-3.949206000	0.812985000
1	5.812349000	-4.654725000	2.970690000
1	4.328200000	-5.204107000	4.927633000
1	1.849237000	-4.965137000	4.709111000
1	-0.110872000	-4.298273000	3.417844000
6	-1.517651000	-3.244045000	1.337086000
6	3.275094000	0.889951000	-2.420078000
6	3.253034000	2.165466000	-2.994108000
6	3.701014000	2.326164000	-4.309645000
6	4.150329000	1.237104000	-5.065804000
6	4.131737000	-0.036111000	-4.487542000
6	-2.074537000	-2.562061000	2.441292000
6	-3.418970000	-2.177599000	2.459211000
6	-4.215879000	-2.474859000	1.348018000
6	-3.697916000	-3.143256000	0.231384000
6	-2.350787000	-3.525629000	0.231034000
1	2.947751000	0.785131000	-1.381470000
1	4.421755000	-0.898694000	-5.103978000
1	-1.438323000	-2.288673000	3.295273000
1	-1.952261000	-4.034838000	-0.654856000
7	-0.328364000	-0.446764000	-0.258370000
15	-1.093594000	0.934283000	-0.376694000
8	-0.775965000	1.872626000	0.946481000
8	-2.669831000	0.522408000	-0.094841000
6	-1.175051000	1.392201000	2.188653000
6	-0.179384000	0.796505000	3.024838000
6	-0.594020000	0.317737000	4.263824000

6	-1.954793000	0.365747000	4.673809000
6	-2.362986000	-0.199988000	5.918753000
6	-3.700743000	-0.242556000	6.277709000
6	-4.683337000	0.282250000	5.397668000
6	-4.317421000	0.862675000	4.191586000
6	-2.948718000	0.936071000	3.795595000
6	-2.525066000	1.484738000	2.528876000
6	-3.513992000	2.027527000	1.554217000
6	-4.422713000	3.079983000	1.916247000
6	-4.275592000	3.853408000	3.103473000
6	-5.175321000	4.864873000	3.407651000
6	-6.271634000	5.137870000	2.547919000
6	-6.435277000	4.407297000	1.381365000
6	-5.513881000	3.378473000	1.026285000
6	-5.643101000	2.657216000	-0.189159000
6	-4.704696000	1.720996000	-0.623440000
6	-3.620259000	1.459228000	0.278351000
6	1.223246000	0.625393000	2.569930000
1	0.148575000	-0.126605000	4.944223000
1	-1.591098000	-0.621181000	6.581520000
1	-4.004159000	-0.694204000	7.233958000
1	-5.748464000	0.216895000	5.664806000
1	-5.093333000	1.233771000	3.512329000
1	-3.426042000	3.651048000	3.770984000
1	-5.033538000	5.464760000	4.318995000
1	-6.982348000	5.937789000	2.803750000
1	-7.272137000	4.622262000	0.698977000
1	-6.527922000	2.863824000	-0.807051000
6	-4.878757000	1.033384000	-1.933346000
6	1.950972000	1.666764000	1.954530000
6	3.269839000	1.476384000	1.526811000
6	3.870199000	0.226616000	1.727603000
6	3.185112000	-0.824224000	2.345468000
6	1.863555000	-0.618374000	2.754912000
6	-5.707289000	1.622778000	-2.920723000
6	-6.028632000	0.962354000	-4.109437000
6	-5.471894000	-0.301216000	-4.351152000
6	-4.583208000	-0.887405000	-3.445689000
6	-4.305263000	-0.223404000	-2.244138000
1	1.492610000	2.651281000	1.815148000
1	1.309888000	-1.459155000	3.196756000
1	-6.113876000	2.630893000	-2.764008000
1	-3.640617000	-0.726427000	-1.536436000
7	0.044254000	-1.234810000	-2.901426000
7	-0.751031000	1.830386000	-1.668967000
16	-0.805885000	-2.357817000	-3.729899000
8	-1.969703000	-2.885902000	-2.978751000
8	-1.036790000	-1.845162000	-5.097047000
16	-1.296298000	3.339547000	-2.056420000
8	-0.379221000	3.841819000	-3.102811000
8	-2.751306000	3.365933000	-2.314031000
1	4.487626000	1.373548000	-6.100687000
1	2.879893000	3.019723000	-2.416800000
1	3.664673000	-1.798056000	2.497867000
1	3.815811000	2.301096000	1.051642000
1	-6.699204000	1.429914000	-4.841298000
1	-4.092712000	-1.848657000	-3.648539000

1	-4.327059000	-3.366677000	-0.639076000
1	-3.830012000	-1.645602000	3.324974000
1	-0.887271000	1.144629000	-3.347783000
16	-5.974436000	-1.973519000	1.359079000
16	-5.930061000	-1.207328000	-5.867281000
16	3.654127000	3.986838000	-5.064056000
16	5.587243000	-0.046816000	1.165540000
9	2.143253000	4.275639000	-4.484460000
9	2.994597000	3.404635000	-6.466760000
9	3.603580000	5.471981000	-5.749245000
9	4.302240000	4.686267000	-3.732553000
9	5.152140000	3.807807000	-5.708969000
9	5.170442000	-1.427660000	0.361360000
9	5.347234000	0.815626000	-0.214575000
9	6.115828000	1.307683000	1.921197000
9	5.952234000	-0.939508000	2.494717000
9	7.121711000	-0.296819000	0.659799000
9	-6.044673000	-2.630890000	-5.069458000
9	-7.529125000	-0.932666000	-5.621811000
9	-6.339400000	-2.008160000	-7.234140000
9	-5.858737000	0.166359000	-6.796866000
9	-4.358559000	-1.530989000	-6.227751000
9	-6.474283000	-3.499266000	1.034389000
9	-5.967239000	-1.630528000	-0.247813000
9	-6.118826000	-2.262868000	2.970729000
9	-5.588030000	-0.400851000	1.683768000
9	-7.546354000	-1.519390000	1.375463000
6	-1.573633000	4.363174000	-7.180596000
6	-2.978021000	4.048663000	-6.965711000
6	-3.423034000	3.428411000	-5.814218000
6	-0.562691000	4.153084000	-6.297061000
1	-2.806634000	3.368384000	-4.905220000
1	-0.726898000	3.824307000	-5.259122000
1	0.479195000	4.379420000	-6.568382000
1	-4.503072000	3.277219000	-5.666688000
6	-1.147951000	0.437336000	-7.824445000
6	0.302355000	-0.074701000	-7.645462000
6	1.155718000	0.852831000	-6.786744000
6	0.612915000	0.890751000	-5.358166000
1	0.271042000	-1.072822000	-7.162085000
1	0.753337000	-0.212778000	-8.650138000
1	2.206375000	0.508103000	-6.766756000
1	1.172547000	1.877889000	-7.210735000
1	0.904273000	-0.014542000	-4.781880000
1	1.008285000	1.748678000	-4.772970000
6	-1.695938000	0.934394000	-6.475838000
6	-3.041414000	1.251544000	-6.298119000
1	-3.471657000	1.178855000	-5.292523000
1	-3.747434000	1.178376000	-7.134185000
6	-0.872049000	0.969085000	-5.313481000
8	-1.479004000	1.117629000	-4.163931000
6	-1.194644000	1.532360000	-8.906815000
1	-0.943485000	1.095976000	-9.895268000
1	-2.207104000	1.977369000	-8.985885000
1	-0.481989000	2.350749000	-8.694325000
6	-2.017010000	-0.758679000	-8.279039000
1	-2.056157000	-1.532006000	-7.488088000

1	-3.057703000	-0.457155000	-8.511060000
1	-1.583589000	-1.206834000	-9.197063000
6	0.411976000	-3.709473000	-3.914622000
6	1.567214000	-3.461788000	-4.680890000
6	0.263738000	-4.974524000	-3.318249000
6	2.545796000	-4.446471000	-4.868259000
6	1.247505000	-5.965830000	-3.489575000
6	2.370022000	-5.714848000	-4.292507000
9	-0.780229000	-5.288816000	-2.551090000
9	1.113300000	-7.154607000	-2.896499000
9	3.281127000	-6.667196000	-4.479551000
9	3.642536000	-4.179926000	-5.585839000
9	1.791430000	-2.262282000	-5.234300000
6	-0.977999000	4.340928000	-0.575592000
6	0.337266000	4.410381000	-0.082085000
6	-1.997445000	5.030408000	0.107090000
6	0.631956000	5.108560000	1.094503000
6	-1.706878000	5.742567000	1.286255000
6	-0.394482000	5.779236000	1.781705000
9	1.876954000	5.111421000	1.584867000
9	-0.121558000	6.445390000	2.900999000
9	-2.674773000	6.395935000	1.930453000
9	-3.261068000	5.033773000	-0.303596000
9	1.333637000	3.739944000	-0.671202000
6	-3.921915000	4.333526000	-8.102241000
1	-3.934734000	5.420208000	-8.333417000
1	-3.590651000	3.824827000	-9.033547000
1	-4.956683000	4.013537000	-7.877404000
1	-1.322249000	4.765478000	-8.177711000

TS-A4: Imaginary frequency = - 189.50

15	0.070024000	-1.128324000	-1.902624000
8	1.705646000	-0.878853000	-1.962597000
8	-0.095760000	-2.435566000	-0.889964000
6	2.583009000	-1.948995000	-2.055884000
6	3.403975000	-2.066225000	-3.225090000
6	4.278873000	-3.149090000	-3.290883000
6	4.333609000	-4.147109000	-2.283501000
6	5.174599000	-5.290980000	-2.416452000
6	5.172607000	-6.286599000	-1.453012000
6	4.316566000	-6.179181000	-0.325720000
6	3.507773000	-5.064344000	-0.154269000
6	3.504500000	-4.009938000	-1.114222000
6	2.678783000	-2.836174000	-0.980821000
6	1.956562000	-2.597449000	0.298935000
6	2.688841000	-2.486483000	1.531447000
6	4.108561000	-2.362660000	1.581788000
6	4.762578000	-2.186210000	2.793478000
6	4.032176000	-2.145608000	4.011777000
6	2.651916000	-2.273149000	3.995469000
6	1.947484000	-2.433286000	2.764505000
6	0.532889000	-2.533219000	2.730834000
6	-0.202608000	-2.563683000	1.544255000
6	0.562948000	-2.511859000	0.328598000
6	3.441794000	-1.060091000	-4.314844000
1	4.957094000	-3.236028000	-4.152082000
1	5.806081000	-5.378311000	-3.313530000
1	5.812116000	-7.173455000	-1.572585000

1	4.288579000	-6.989968000	0.417295000
1	2.844448000	-4.997815000	0.718752000
1	4.681728000	-2.380437000	0.644446000
1	5.856690000	-2.070965000	2.809867000
1	4.564798000	-2.008169000	4.964549000
1	2.071854000	-2.236876000	4.930827000
1	0.006487000	-2.584032000	3.693693000
6	-1.685218000	-2.676198000	1.588203000
6	3.495086000	0.322156000	-4.039155000
6	3.751917000	1.249179000	-5.057164000
6	3.917772000	0.787678000	-6.368372000
6	3.818341000	-0.573619000	-6.686346000
6	3.585361000	-1.488194000	-5.654862000
6	-2.378826000	-2.300715000	2.761389000
6	-3.769283000	-2.376283000	2.848969000
6	-4.494156000	-2.832648000	1.743968000
6	-3.845251000	-3.271660000	0.586272000
6	-2.448252000	-3.195155000	0.517154000
1	3.388964000	0.683122000	-3.010499000
1	3.507957000	-2.558359000	-5.891720000
1	-1.836531000	-1.905853000	3.628401000
1	-1.972449000	-3.535681000	-0.406347000
7	-0.441519000	0.250718000	-1.362595000
15	-1.663913000	1.152319000	-0.910145000
8	-1.240143000	2.099595000	0.373803000
8	-2.730999000	0.122534000	-0.173738000
6	-1.082711000	1.592179000	1.646539000
6	0.252625000	1.484323000	2.150204000
6	0.403303000	1.015196000	3.451327000
6	-0.709436000	0.642424000	4.251671000
6	-0.515355000	0.125286000	5.568387000
6	-1.589659000	-0.287625000	6.340151000
6	-2.906400000	-0.196172000	5.815842000
6	-3.128238000	0.306915000	4.542015000
6	-2.047573000	0.748845000	3.720444000
6	-2.225143000	1.242204000	2.375037000
6	-3.570022000	1.325647000	1.733290000
6	-4.661937000	2.022403000	2.363401000
6	-4.494364000	2.836669000	3.523525000
6	-5.581043000	3.451457000	4.126976000
6	-6.888677000	3.289697000	3.596389000
6	-7.076971000	2.553284000	2.439275000
6	-5.976331000	1.926300000	1.781616000
6	-6.147216000	1.261103000	0.540232000
6	-5.079240000	0.701540000	-0.158312000
6	-3.801462000	0.721848000	0.487165000
6	1.433067000	1.813050000	1.312240000
1	1.411885000	0.930462000	3.882850000
1	0.513370000	0.057493000	5.955727000
1	-1.426681000	-0.687212000	7.352198000
1	-3.760650000	-0.536095000	6.420597000
1	-4.149635000	0.341236000	4.144584000
1	-3.485401000	2.987941000	3.930326000
1	-5.424729000	4.086069000	5.011709000
1	-7.743206000	3.778517000	4.086872000
1	-8.077459000	2.458850000	1.991073000
1	-7.159276000	1.198985000	0.114882000

6	-5.283617000	0.145625000	-1.521095000
6	1.447325000	2.906381000	0.415522000
6	2.583328000	3.188233000	-0.351284000
6	3.712185000	2.370831000	-0.219541000
6	3.732227000	1.275330000	0.652087000
6	2.590101000	1.008304000	1.412484000
6	-6.201374000	0.779149000	-2.390950000
6	-6.447340000	0.288381000	-3.677396000
6	-5.760043000	-0.855589000	-4.100397000
6	-4.837951000	-1.507386000	-3.276122000
6	-4.607750000	-1.001345000	-1.992909000
1	0.567744000	3.549803000	0.293034000
1	2.593293000	0.134464000	2.077707000
1	-6.705559000	1.700956000	-2.071229000
1	-3.883730000	-1.520928000	-1.357529000
7	-0.494181000	-1.671205000	-3.328992000
7	-2.379577000	2.112899000	-1.967330000
16	-1.222674000	-3.128097000	-3.565899000
8	-2.283627000	-3.424319000	-2.577760000
8	-1.587147000	-3.178651000	-4.996504000
16	-2.246853000	3.739990000	-1.928439000
8	-0.861257000	4.200436000	-1.679840000
8	-2.966341000	4.278002000	-3.103892000
1	3.939969000	-0.919989000	-7.720237000
1	3.852435000	2.311989000	-4.807728000
1	4.614770000	0.629135000	0.736310000
1	2.572670000	4.038032000	-1.045028000
1	-7.153746000	0.801254000	-4.341875000
1	-4.283441000	-2.391726000	-3.612725000
1	-4.409080000	-3.663637000	-0.269477000
1	-4.278608000	-2.060540000	3.765745000
1	-1.440120000	-0.609107000	-4.308768000
16	-6.315416000	-2.797694000	1.818276000
16	-6.037922000	-1.493615000	-5.785858000
16	4.274698000	1.982742000	-7.702271000
16	5.196704000	2.735654000	-1.214108000
9	3.236364000	3.097149000	-7.075935000
9	3.047385000	1.378014000	-8.638334000
9	4.577270000	3.043269000	-8.904088000
9	5.503655000	2.655803000	-6.859707000
9	5.324705000	0.955330000	-8.431774000
9	5.359114000	1.152112000	-1.659476000
9	4.287886000	3.020140000	-2.569841000
9	5.137930000	4.330390000	-0.850617000
9	6.203819000	2.474543000	0.052925000
9	6.516110000	3.049740000	-2.124389000
9	-6.212419000	-3.032057000	-5.258061000
9	-7.658211000	-1.263497000	-5.690018000
9	-6.272553000	-2.049076000	-7.309309000
9	-5.879827000	0.021216000	-6.458762000
9	-4.428366000	-1.752773000	-6.021431000
9	-6.440294000	-4.297785000	1.169651000
9	-6.429953000	-2.139138000	0.316275000
9	-6.330497000	-3.445171000	3.328617000
9	-6.315794000	-1.283339000	2.476277000
9	-7.948245000	-2.754200000	1.890214000
6	-2.065114000	-1.135764000	-7.877014000

6	-0.637403000	-1.378721000	-7.952793000
6	0.266775000	-0.328500000	-8.018424000
6	-2.672690000	0.068288000	-8.054586000
1	-0.047040000	0.663953000	-8.357119000
1	-2.127543000	0.951106000	-8.418625000
1	-3.755812000	0.171746000	-7.896290000
1	1.342934000	-0.538341000	-8.098494000
6	-0.544214000	2.898023000	-6.176062000
6	-1.811943000	3.779091000	-6.004969000
6	-3.124125000	3.056836000	-6.295213000
6	-3.269460000	1.896297000	-5.317619000
1	-1.867368000	4.147996000	-4.963216000
1	-1.700131000	4.675413000	-6.648737000
1	-3.976299000	3.752820000	-6.165907000
1	-3.167150000	2.694618000	-7.344606000
1	-3.397142000	2.294525000	-4.283589000
1	-4.152807000	1.254205000	-5.499594000
6	-0.801786000	1.414402000	-5.807495000
6	0.223191000	0.467092000	-5.930202000
1	1.251064000	0.810814000	-6.108467000
1	0.147551000	-0.525252000	-5.464200000
6	-2.068032000	1.017969000	-5.263351000
8	-2.265200000	-0.094891000	-4.606447000
6	-0.028089000	3.048585000	-7.622036000
1	0.222231000	4.111658000	-7.810179000
1	0.891063000	2.467983000	-7.820298000
1	-0.800176000	2.759022000	-8.365144000
6	0.527939000	3.431336000	-5.194634000
1	0.189423000	3.340096000	-4.143113000
1	1.491488000	2.899572000	-5.305234000
1	0.715580000	4.506200000	-5.394862000
6	0.094072000	-4.369771000	-3.330234000
6	1.146442000	-4.404528000	-4.263039000
6	0.056910000	-5.360949000	-2.330725000
6	2.113828000	-5.416410000	-4.240452000
6	1.036603000	-6.370188000	-2.285931000
6	2.040907000	-6.422531000	-3.263935000
9	-0.872704000	-5.387252000	-1.377866000
9	1.005193000	-7.290229000	-1.321065000
9	2.941734000	-7.400811000	-3.243660000
9	3.113581000	-5.415506000	-5.129605000
9	1.263936000	-3.452972000	-5.196336000
6	-3.274745000	4.281632000	-0.515658000
6	-2.744580000	4.865243000	0.649451000
6	-4.674820000	4.225030000	-0.657971000
6	-3.596203000	5.398349000	1.633515000
6	-5.530561000	4.771661000	0.308429000
6	-4.986329000	5.386080000	1.446398000
9	-3.085423000	5.922811000	2.751631000
9	-5.785502000	5.924525000	2.366572000
9	-6.855886000	4.702994000	0.148808000
9	-5.240467000	3.642969000	-1.718469000
9	-1.433722000	4.932063000	0.889490000
6	-0.175589000	-2.800643000	-7.840889000
1	-0.582394000	-3.265788000	-6.920765000
1	-0.568330000	-3.386355000	-8.699964000
1	0.926925000	-2.883451000	-7.836368000

1	-2.690216000	-1.992260000	-7.578079000

TS-A5: Imaginary frequency = - 215.51

15	0.390201000	-1.441959000	-1.256245000
8	2.010011000	-1.202167000	-1.021990000
8	0.253018000	-3.028246000	-0.830046000
6	2.937973000	-2.126015000	-1.469854000
6	3.806306000	-1.742248000	-2.544378000
6	4.801119000	-2.655767000	-2.896289000
6	4.891789000	-3.949687000	-2.321313000
6	5.861192000	-4.894282000	-2.773712000
6	5.890858000	-6.181084000	-2.262923000
6	4.933061000	-6.580449000	-1.293048000
6	3.990758000	-5.680220000	-0.817832000
6	3.960348000	-4.335894000	-1.292909000
6	3.026631000	-3.356908000	-0.804650000
6	2.202789000	-3.615958000	0.411125000
6	2.805703000	-3.982533000	1.669882000
6	4.217295000	-4.067555000	1.862529000
6	4.752037000	-4.392507000	3.101305000
6	3.905141000	-4.659377000	4.209028000
6	2.530690000	-4.562858000	4.062657000
6	1.948098000	-4.204625000	2.809857000
6	0.544950000	-4.006479000	2.689027000
6	-0.045340000	-3.586780000	1.499712000
6	0.819849000	-3.426110000	0.372169000
6	3.673167000	-0.460053000	-3.286628000
1	5.539682000	-2.381923000	-3.662861000
1	6.571968000	-4.583954000	-3.554724000
1	6.635067000	-6.904345000	-2.627652000
1	4.930337000	-7.616616000	-0.923341000
1	3.246878000	-6.002208000	-0.076203000
1	4.888400000	-3.834517000	1.027346000
1	5.844464000	-4.430912000	3.225607000
1	4.341765000	-4.924693000	5.183397000
1	1.862072000	-4.742570000	4.918867000
1	-0.088858000	-4.176235000	3.572754000
6	-1.490392000	-3.251759000	1.432899000
6	3.142877000	0.726677000	-2.724392000
6	3.034637000	1.904670000	-3.473536000
6	3.496237000	1.909382000	-4.794495000
6	4.032184000	0.761011000	-5.390300000
6	4.096974000	-0.413463000	-4.637390000
6	-2.083524000	-2.554628000	2.508663000
6	-3.438133000	-2.207618000	2.493398000
6	-4.205922000	-2.551397000	1.375375000
6	-3.652311000	-3.236013000	0.286666000
6	-2.297633000	-3.587980000	0.323306000
1	2.806626000	0.745622000	-1.683458000
1	4.449607000	-1.330664000	-5.128622000
1	-1.469238000	-2.238920000	3.364186000
1	-1.873575000	-4.115588000	-0.538747000
7	-0.276807000	-0.522636000	-0.152740000
15	-1.058858000	0.851647000	-0.326492000
8	-0.810540000	1.825465000	0.987125000
8	-2.637200000	0.418015000	-0.083329000
6	-1.206717000	1.356947000	2.231422000
6	-0.195109000	0.822611000	3.090574000

6	-0.600884000	0.362705000	4.338970000
6	-1.966681000	0.375739000	4.736980000
6	-2.362914000	-0.161024000	5.998583000
6	-3.699977000	-0.234504000	6.354872000
6	-4.694714000	0.226779000	5.453410000
6	-4.342073000	0.775898000	4.228659000
6	-2.974742000	0.882775000	3.835258000
6	-2.562754000	1.408193000	2.554168000
6	-3.557546000	1.896969000	1.555938000
6	-4.509709000	2.919596000	1.895104000
6	-4.400507000	3.717781000	3.070154000
6	-5.344326000	4.693424000	3.357056000
6	-6.449851000	4.904723000	2.491692000
6	-6.575367000	4.152579000	1.334204000
6	-5.606713000	3.162109000	0.995674000
6	-5.693538000	2.427058000	-0.215126000
6	-4.707989000	1.534988000	-0.636153000
6	-3.623988000	1.318880000	0.280274000
6	1.210317000	0.694334000	2.630905000
1	0.150813000	-0.034715000	5.038206000
1	-1.580677000	-0.533251000	6.678359000
1	-3.992735000	-0.661677000	7.325593000
1	-5.758510000	0.137324000	5.719111000
1	-5.126683000	1.097738000	3.534486000
1	-3.544448000	3.564909000	3.742233000
1	-5.231213000	5.312301000	4.259733000
1	-7.196990000	5.674880000	2.734935000
1	-7.417693000	4.321196000	0.645439000
1	-6.579956000	2.591201000	-0.843592000
6	-4.819308000	0.862159000	-1.960903000
6	1.873854000	1.735046000	1.944775000
6	3.177246000	1.574298000	1.463806000
6	3.832558000	0.357676000	1.692198000
6	3.221672000	-0.683875000	2.397643000
6	1.910393000	-0.510180000	2.854086000
6	-5.628881000	1.444627000	-2.967954000
6	-5.832753000	0.830788000	-4.206835000
6	-5.178774000	-0.379565000	-4.475947000
6	-4.341140000	-0.974670000	-3.529257000
6	-4.181867000	-0.360594000	-2.280459000
1	1.369579000	2.692631000	1.779649000
1	1.409674000	-1.349678000	3.357424000
1	-6.103885000	2.419315000	-2.792358000
1	-3.548084000	-0.869899000	-1.549153000
7	-0.026015000	-1.346297000	-2.821079000
7	-0.637378000	1.689882000	-1.616648000
16	-0.789492000	-2.583539000	-3.606645000
8	-1.939946000	-3.120400000	-2.843369000
8	-1.014433000	-2.149141000	-4.998980000
16	-1.238932000	3.106566000	-2.139482000
8	-0.366705000	3.577187000	-3.241101000
8	-2.702445000	3.076465000	-2.357398000
1	4.365808000	0.769936000	-6.435135000
1	2.577061000	2.795536000	-3.025199000
1	3.747853000	-1.628975000	2.576277000
1	3.664177000	2.394774000	0.921982000
1	-6.475806000	1.302621000	-4.959855000

1	-3.803290000	-1.908736000	-3.735280000
1	-4.258176000	-3.496006000	-0.590224000
1	-3.879674000	-1.665392000	3.337620000
1	-0.668280000	0.234341000	-3.626137000
16	-5.973388000	-2.087323000	1.337961000
16	-5.398983000	-1.183411000	-6.100293000
16	3.328974000	3.426654000	-5.792078000
16	5.521148000	0.116117000	1.039888000
9	1.745457000	3.596722000	-5.362698000
9	2.860084000	2.574144000	-7.134521000
9	3.154370000	4.784415000	-6.696516000
9	3.772524000	4.390075000	-4.546700000
9	4.882281000	3.353907000	-6.305126000
9	5.148574000	-1.376236000	0.438256000
9	5.126536000	0.785683000	-0.411278000
9	6.011525000	1.581814000	1.585221000
9	6.032048000	-0.582989000	2.434849000
9	7.031042000	-0.103551000	0.451530000
9	-5.314414000	-2.685929000	-5.460645000
9	-7.031757000	-1.199335000	-5.942604000
9	-5.590265000	-1.887597000	-7.567162000
9	-5.513476000	0.273262000	-6.891333000
9	-3.775930000	-1.202075000	-6.378154000
9	-6.429917000	-3.612964000	0.952029000
9	-5.914112000	-1.698161000	-0.257604000
9	-6.165609000	-2.427758000	2.934382000
9	-5.636068000	-0.516975000	1.722064000
9	-7.554671000	-1.667450000	1.310144000
6	-1.707783000	5.214263000	-6.901907000
6	-2.985422000	4.634969000	-6.524323000
6	-3.127552000	3.817541000	-5.402783000
6	-0.570768000	5.170830000	-6.157751000
1	-2.330053000	3.770163000	-4.650838000
1	-0.529686000	4.704698000	-5.159747000
1	0.367857000	5.598979000	-6.540014000
1	-4.142511000	3.662044000	-5.006267000
6	-1.352637000	1.203108000	-7.908850000
6	-0.626700000	-0.165810000	-8.006495000
6	0.666044000	-0.226602000	-7.190008000
6	0.500490000	0.383771000	-5.782140000
1	-1.326515000	-0.948305000	-7.644639000
1	-0.410814000	-0.386958000	-9.073076000
1	0.979649000	-1.282172000	-7.082901000
1	1.484294000	0.300139000	-7.720223000
1	0.862715000	-0.291183000	-4.985843000
1	1.107401000	1.309239000	-5.691832000
6	-1.752152000	1.372176000	-6.433919000
6	-3.008074000	1.851735000	-6.007675000
1	-3.309236000	1.567361000	-4.989460000
1	-3.825436000	1.913438000	-6.734842000
6	-0.879700000	0.836882000	-5.452805000
8	-1.280332000	0.785931000	-4.204842000
6	-0.427029000	2.335156000	-8.408252000
1	-0.055232000	2.098240000	-9.427244000
1	-0.974007000	3.294657000	-8.458618000
1	0.449175000	2.496829000	-7.753539000
6	-2.596956000	1.150049000	-8.812704000

1	-3.304363000	0.361778000	-8.489359000
1	-3.140304000	2.117006000	-8.826201000
1	-2.290816000	0.934041000	-9.855956000
6	0.480402000	-3.896030000	-3.686253000
6	1.628439000	-3.671245000	-4.468695000
6	0.360868000	-5.129151000	-3.017916000
6	2.632542000	-4.640200000	-4.594258000
6	1.369602000	-6.103372000	-3.126921000
6	2.488420000	-5.874132000	-3.941575000
9	-0.674754000	-5.426212000	-2.234201000
9	1.264136000	-7.255144000	-2.460318000
9	3.426178000	-6.809723000	-4.064310000
9	3.722827000	-4.390806000	-5.327579000
9	1.822019000	-2.505904000	-5.096742000
6	-0.927229000	4.222666000	-0.730469000
6	0.395389000	4.358326000	-0.271598000
6	-1.961086000	4.852428000	-0.013825000
6	0.686043000	5.071505000	0.897648000
6	-1.676838000	5.575684000	1.159896000
6	-0.354588000	5.683163000	1.617524000
9	1.942518000	5.137559000	1.354755000
9	-0.085704000	6.357861000	2.733740000
9	-2.662282000	6.163364000	1.841750000
9	-3.237334000	4.779599000	-0.382566000
9	1.410950000	3.737383000	-0.884154000
6	-4.142337000	4.867940000	-7.452121000
1	-4.301245000	5.953330000	-7.626478000
1	-3.932105000	4.425838000	-8.451942000
1	-5.083489000	4.429232000	-7.070977000
1	-1.678573000	5.725270000	-7.880889000

TS-A6: Imaginary frequency = - 166.15

15	0.395172084	-1.433208597	-1.234192703
8	1.981718177	-1.176025725	-0.841959190
8	0.231494817	-3.025898125	-0.803979112
6	2.915572512	-2.064115286	-1.355973041
6	3.761774698	-1.629913543	-2.428508160
6	4.720083412	-2.545882643	-2.867236359
6	4.804352177	-3.870971838	-2.366295195
6	5.738777245	-4.807660286	-2.901555003
6	5.763230047	-6.119255268	-2.458271272
6	4.841132426	-6.550797063	-1.467503630
6	3.933744101	-5.660931004	-0.912309831
6	3.904690195	-4.296141576	-1.325147380
6	2.997136215	-3.332629025	-0.767413461
6	2.171554344	-3.638900622	0.435595027
6	2.772392531	-4.044054021	1.681158900
6	4.183446992	-4.155625879	1.865673857
6	4.716314256	-4.525829598	3.092521585
6	3.868097633	-4.810756380	4.195185762
6	2.494930736	-4.687219946	4.057039594
6	1.914546309	-4.284009672	2.816860476
6	0.515340126	-4.058771227	2.702985771
6	-0.072598161	-3.604315678	1.523985235
6	0.789116760	-3.438277292	0.394063560
6	3.649822949	-0.291724667	-3.068382447
1	5.439939667	-2.246838941	-3.642183458
1	6.425160769	-4.470630514	-3.693166429

1	6.478802558	-6.835321071	-2.888686344
1	4.837764579	-7.603206032	-1.146964857
1	3.216287211	-6.005089510	-0.154566923
1	4.854524743	-3.912522019	1.032785408
1	5.808262366	-4.588441894	3.211678900
1	4.303421687	-5.112308937	5.159584142
1	1.826209880	-4.881185853	4.910127169
1	-0.118372842	-4.238183703	3.584806091
6	-1.512167979	-3.241956812	1.483560312
6	3.129340107	0.848520298	-2.409986306
6	3.046630171	2.089847430	-3.047874564
6	3.517972931	2.210469966	-4.358821195
6	4.049940695	1.113491067	-5.045743939
6	4.093003557	-0.126379593	-4.402180124
6	-2.076762443	-2.583565903	2.599104519
6	-3.430150526	-2.235526513	2.634865431
6	-4.228715960	-2.541325594	1.527901490
6	-3.702246893	-3.173332175	0.394286140
6	-2.345487849	-3.520953969	0.376327778
1	2.777114931	0.774974880	-1.377194689
1	4.449735849	-0.996163996	-4.970787985
1	-1.439632183	-2.303836628	3.450353368
1	-1.941115398	-4.007988709	-0.519371297
7	-0.398000997	-0.552591939	-0.174893790
15	-1.236799198	0.779158038	-0.306709975
8	-0.875688688	1.792151414	0.956409903
8	-2.775509941	0.328805911	0.098178200
6	-1.160981285	1.330645980	2.236724040
6	-0.088132229	0.797666350	3.018317999
6	-0.398927620	0.353882754	4.301255990
6	-1.726935209	0.366049727	4.807008228
6	-2.022121030	-0.158175923	6.101241809
6	-3.327884256	-0.233489935	6.558386536
6	-4.391135141	0.210641129	5.728789712
6	-4.135952261	0.746975473	4.474567006
6	-2.801854934	0.859487977	3.980652281
6	-2.486939897	1.378931957	2.671545379
6	-3.558967308	1.858069938	1.754800578
6	-4.479767472	2.885321843	2.155019121
6	-4.285380688	3.688681514	3.316937461
6	-5.211424166	4.658554923	3.671706404
6	-6.377183912	4.864985642	2.887044643
6	-6.582554766	4.113753296	1.741223335
6	-5.638543862	3.124059976	1.334801769
6	-5.806361394	2.389950376	0.132136258
6	-4.852941141	1.494541600	-0.354580057
6	-3.722905748	1.257946457	0.498949669
6	1.291642501	0.649581057	2.490242624
1	0.406181416	-0.033216116	4.944192774
1	-1.188339599	-0.519245452	6.723272629
1	-3.544154584	-0.650004788	7.553488241
1	-5.431066185	0.118527240	6.075840420
1	-4.972863144	1.055062458	3.837208779
1	-3.383773418	3.536345508	3.926896520
1	-5.037133676	5.278388296	4.563922819
1	-7.107261885	5.632982508	3.182916136
1	-7.471607698	4.280908554	1.113722821

1	-6.724456805	2.566664208	-0.445299120
6	-5.014499901	0.877580347	-1.699805642
6	1.951075854	1.666953490	1.764790656
6	3.268132084	1.495767877	1.319495631
6	3.931111767	0.295122748	1.605897557
6	3.307115773	-0.734933870	2.315298530
6	1.989078600	-0.551789314	2.743643947
6	-5.821462075	1.534813302	-2.659857703
6	-5.997446574	1.030731254	-3.949286783
6	-5.326015296	-0.145903326	-4.305890209
6	-4.505909109	-0.824440476	-3.401297846
6	-4.371754330	-0.316452019	-2.103283217
1	1.442939618	2.615746899	1.555030662
1	1.482435870	-1.378642773	3.261918998
1	-6.291604382	2.496438991	-2.415215336
1	-3.742145366	-0.879027382	-1.408846171
7	0.202932689	-1.301558767	-2.814714650
7	-1.034776319	1.620755091	-1.647868959
16	-0.721437888	-2.318042927	-3.680511844
8	-1.949821114	-2.752635728	-2.966568228
8	-0.861308320	-1.780312978	-5.047914066
16	-1.677078187	2.958973533	-2.233302759
8	-0.715239984	3.467527971	-3.275864371
8	-3.080994596	2.826427629	-2.675843888
1	4.406890181	1.213552529	-6.078230233
1	2.598978644	2.943368509	-2.523758623
1	3.830929405	-1.674700699	2.526916185
1	3.772146436	2.298605529	0.766458092
1	-6.627285833	1.562568446	-4.672942247
1	-3.944839087	-1.727160376	-3.678139723
1	-4.334351188	-3.399847720	-0.473529114
1	-3.847507973	-1.725937985	3.511452415
1	-0.242392269	2.275146684	-4.270084063
16	-6.001551018	-2.101701987	1.573904527
16	-5.510786814	-0.795941914	-5.996787468
16	3.391523374	3.817616837	-5.207935976
16	5.654217214	0.058363790	1.046030503
9	1.913342031	4.130010233	-4.554630626
9	2.654818560	3.133797936	-6.532902643
9	3.261132356	5.255654072	-5.989743704
9	4.104214148	4.616978106	-3.971378025
9	4.847675856	3.632747737	-5.940198914
9	5.328890472	-1.450814429	0.462055600
9	5.333624525	0.691993985	-0.437455913
9	6.098367910	1.542427375	1.583808176
9	6.096910889	-0.600831951	2.483051538
9	7.196194775	-0.157203109	0.546340244
9	-7.142722901	-0.637754474	-5.961871688
9	-5.402401408	0.731467633	-6.661205566
9	-5.660042157	-1.351131102	-7.532673614
9	-3.880391152	-0.983218052	-6.167859807
9	-5.639018356	-2.342037805	-5.484741813
9	-6.451349494	-3.599858541	1.086142092
9	-5.993042139	-1.593295976	0.010566858
9	-6.143455781	-2.563946723	3.144181141
9	-5.674775762	-0.558408712	2.067295622
9	-7.588141183	-1.703184197	1.621062754

6	-1.407216837	4.792819449	-7.479947267
6	-2.705977447	4.431477661	-6.949034222
6	-2.830497974	3.854440658	-5.688520246
6	-0.217085145	4.544226501	-6.867469033
1	-2.044737645	4.009120926	-4.936706660
1	-0.151086708	4.127369982	-5.853744865
1	0.740157531	4.792858839	-7.347274339
1	-3.835021317	3.679795561	-5.274374138
6	-1.275654782	0.961895742	-8.076536256
6	-0.813428521	-0.521354877	-8.057014071
6	0.653664707	-0.669865210	-7.650845535
6	1.057545061	0.253537602	-6.485873943
1	-1.459147817	-1.074250633	-7.345871614
1	-0.974641495	-0.963031559	-9.063452332
1	0.846604048	-1.717808635	-7.352255571
1	1.307579650	-0.463781486	-8.522247394
1	1.447129845	-0.312119723	-5.619231363
1	1.886675105	0.934766723	-6.773141775
6	-1.211830302	1.428157691	-6.606274732
6	-2.359180790	1.809728001	-5.881991654
1	-2.349449511	1.723638747	-4.784428894
1	-3.332147654	1.582112654	-6.331157965
6	-0.000441053	1.141454447	-5.912808372
8	0.318690795	1.557395557	-4.717035316
6	-0.345861343	1.796939482	-8.987656558
1	-0.232178231	1.296018112	-9.971402752
1	-0.768261008	2.803729866	-9.162848405
1	0.663201729	1.942729964	-8.556391460
6	-2.694851899	1.020386402	-8.659800061
1	-3.388690994	0.331711014	-8.146280487
1	-3.122073080	2.040983846	-8.614752129
1	-2.661105833	0.723680460	-9.727301566
6	0.380437069	-3.781582846	-3.810507719
6	1.592081286	-3.649585047	-4.514150299
6	0.118450897	-5.006378695	-3.170389255
6	2.514418751	-4.700689041	-4.599553447
6	1.047218613	-6.062366370	-3.229273065
6	2.235007473	-5.919449010	-3.961868740
9	-0.990936822	-5.226022959	-2.463254912
9	0.796774355	-7.211615166	-2.595959106
9	3.098057030	-6.931287173	-4.038778280
9	3.655258441	-4.540675760	-5.279365220
9	1.929563908	-2.498851083	-5.111374058
6	-1.633170271	4.164519151	-0.884926275
6	-0.416042329	4.375458494	-0.210383632
6	-2.796644168	4.796467696	-0.403791681
6	-0.367462510	5.125537552	0.970687459
6	-2.750292544	5.567327910	0.771492552
6	-1.540619317	5.721348532	1.466123886
9	0.782793491	5.253071081	1.639761981
9	-1.504361394	6.430292307	2.591353718
9	-3.854892698	6.161375753	1.221798745
9	-3.977634723	4.684511853	-1.005119242
9	0.712576582	3.794485643	-0.628963968
6	-3.901288879	4.603473399	-7.842812599
1	-4.161395552	5.681867671	-7.927800595
1	-3.697597507	4.251985711	-8.876006248

1	-4.788902306	4.069796253	-7.453402853
1	-1.397027259	5.239491651	-8.489595220

Minor ISOMER:

TS-B1: Imaginary frequency = - 97.97

15	0.156054610	-1.245127770	-1.605058235
8	1.791687658	-0.985217081	-1.731490668
8	0.084073460	-2.685661317	-0.793647084
6	2.661067854	-2.031857545	-2.008880773
6	3.402705997	-2.002424755	-3.235785696
6	4.271952432	-3.062094792	-3.490268640
6	4.393060814	-4.176317542	-2.621236157
6	5.222899855	-5.288309412	-2.950406457
6	5.279677780	-6.397852885	-2.122880342
6	4.492683138	-6.440547620	-0.942190939
6	3.697984860	-5.361589215	-0.581131554
6	3.640524995	-4.191542580	-1.394545851
6	2.829264415	-3.048170675	-1.060705209
6	2.183627700	-2.976370763	0.277825775
6	2.967569805	-3.045656795	1.482257123
6	4.393236354	-2.998865542	1.481746621
6	5.105982044	-3.043720564	2.671881740
6	4.429434715	-3.150679209	3.916345893
6	3.044364611	-3.184081682	3.950642177
6	2.279766911	-3.114500033	2.747400421
6	0.859106700	-3.082763836	2.774630122
6	0.086660977	-2.926460723	1.622881670
6	0.798459387	-2.854376645	0.381961204
6	3.379891278	-0.876603722	-4.202302634
1	4.893175707	-3.036167222	-4.397150893
1	5.796708188	-5.256827601	-3.888972205
1	5.909521795	-7.258186440	-2.392900801
1	4.506425907	-7.340411267	-0.309515922
1	3.087053525	-5.413226241	0.330127830
1	4.924550975	-2.898840546	0.525149763
1	6.204545210	-2.988181777	2.650369491
1	5.007239253	-3.193787297	4.851560616
1	2.508763873	-3.247991314	4.910553565
1	0.356012750	-3.182081021	3.747802968
6	-1.393278329	-2.848015122	1.709401181
6	3.456881203	0.465022968	-3.774061897
6	3.697178082	1.500584022	-4.686421364
6	3.814638676	1.192138101	-6.047210075
6	3.673484194	-0.121561116	-6.514241721
6	3.459089559	-1.146848670	-5.587811720
6	-1.990010700	-2.265082504	2.848869632
6	-3.378141976	-2.199688580	2.988779755
6	-4.186289978	-2.697344809	1.963176126
6	-3.635049739	-3.291570591	0.822885547
6	-2.241832111	-3.372555659	0.707613022
1	3.393131007	0.702045752	-2.707724290
1	3.351541513	-2.180199497	-5.945525503
1	-1.365142820	-1.820769631	3.635710301
1	-1.830136244	-3.842669450	-0.190819584
7	-0.323565844	-0.026140473	-0.738966253
15	-1.520119906	1.013502138	-0.583691785
8	-1.186364522	2.007140742	0.696189284

8	-2.782324092	0.153265807	0.078569435
6	-1.206521463	1.537604853	1.998286872
6	0.054261004	1.318240527	2.634202775
6	0.046786103	0.925032797	3.967676741
6	-1.164419023	0.716059620	4.681054858
6	-1.142265437	0.292539501	6.044042875
6	-2.316328354	0.011616589	6.723357667
6	-3.560501020	0.130295936	6.050461086
6	-3.615918868	0.551467509	4.729079922
6	-2.429708563	0.883934575	4.006668120
6	-2.436030009	1.326942650	2.629767210
6	-3.699747830	1.486663077	1.861806146
6	-4.795524219	2.264469169	2.379244736
6	-4.668396737	3.133199457	3.503763150
6	-5.764299213	3.824915884	3.998194388
6	-7.044498441	3.671917016	3.402393675
6	-7.189544384	2.878276309	2.277277616
6	-6.071970783	2.189386479	1.717887534
6	-6.183617876	1.466870841	0.503489739
6	-5.093300811	0.848501876	-0.106685924
6	-3.858516606	0.848899526	0.620095265
6	1.329605266	1.448569193	1.888432014
1	1.004245800	0.772837791	4.488999135
1	-0.166043931	0.184043102	6.541793359
1	-2.286760133	-0.316058895	7.773211214
1	-4.493481342	-0.124871495	6.574998432
1	-4.586563951	0.603171627	4.221841068
1	-3.682853610	3.262030994	3.971966948
1	-5.637264430	4.502852502	4.855165884
1	-7.909547373	4.211549894	3.815333169
1	-8.164938606	2.788958250	1.775126851
1	-7.168399340	1.414782796	0.017616053
6	-5.254007729	0.240975497	-1.451655760
6	1.636220835	2.581164090	1.103606495
6	2.855938455	2.667942893	0.422113273
6	3.764464069	1.605977549	0.524414466
6	3.488835129	0.467081719	1.287569254
6	2.269891643	0.401106888	1.968390176
6	-6.134472836	0.860927163	-2.367876142
6	-6.376481024	0.319902223	-3.632193106
6	-5.709470803	-0.856067579	-3.991586948
6	-4.803888620	-1.485204376	-3.130448812
6	-4.584896782	-0.933281858	-1.863174943
1	0.916999142	3.406424620	1.022948768
1	2.030415198	-0.501314859	2.547554622
1	-6.605849662	1.816772344	-2.104966427
1	-3.884482546	-1.442851657	-1.192731145
7	-0.506369370	-1.574837017	-3.056174073
7	-1.800099637	1.895926219	-1.858660098
16	-1.232846116	-3.016974960	-3.421564452
8	-2.237753905	-3.428190096	-2.416897556
8	-1.652761272	-2.926015489	-4.832725005
16	-2.656438335	3.202728508	-2.306956056
8	-1.741079259	4.127597854	-3.015655586
8	-3.902614853	2.799795240	-2.981467861
1	3.748772989	-0.347894229	-7.584927801
1	3.825298758	2.527235955	-4.321773606

1	4.201267593	-0.364336670	1.350856595
1	3.089903518	3.553978013	-0.180955012
1	-7.054911274	0.822620456	-4.332166171
1	-4.263331887	-2.393644137	-3.422885698
1	-4.270708365	-3.692495099	0.023408155
1	-3.816776202	-1.745482651	3.883322927
1	-1.187488610	-0.485522212	-4.108943668
16	-5.997474540	-2.531935367	2.120717830
16	-5.996194244	-1.569481876	-5.641773867
16	4.200402048	2.512834408	-7.249596711
16	5.350811256	1.691038444	-0.372820022
9	3.226553049	3.597944645	-6.487482621
9	2.941775550	2.065433857	-8.223043954
9	4.537216449	3.686033325	-8.333618151
9	5.470231583	3.036515146	-6.358973111
9	5.199189003	1.530392551	-8.103230014
9	5.107793405	0.200444605	-1.050885957
9	4.641796909	2.404196872	-1.687654411
9	5.695040423	3.173169847	0.231640698
9	6.174980519	0.988307032	0.858828005
9	6.760112533	1.756391117	-1.195094434
9	-6.085859631	-3.095797849	-5.056832163
9	-7.622995815	-1.419157047	-5.523046984
9	-6.226033847	-2.204807505	-7.142053681
9	-5.925693428	-0.088796532	-6.377080583
9	-4.377036196	-1.762341523	-5.891410517
9	-6.247091615	-4.082535999	1.653354094
9	-6.156045745	-2.035277512	0.562200039
9	-5.968476923	-3.006460135	3.694103331
9	-5.873892225	-0.956105538	2.605048007
9	-7.618340908	-2.374054066	2.266399717
6	-1.933726117	-1.370660151	-8.137404210
6	-0.574906685	-1.228225046	-7.623537771
6	0.227319354	-0.144044425	-7.775422030
6	-2.596679515	-0.320528334	-8.732828943
1	-0.046998961	0.707025037	-8.414454904
1	-2.067107883	0.577997260	-9.071597141
1	-3.630518854	-0.452975488	-9.086405654
1	1.220010589	-0.101307702	-7.306323932
6	-1.389904685	3.083024953	-6.933244282
6	0.096847763	3.290252837	-6.563496344
6	0.394927527	3.029191974	-5.089909987
6	0.038335009	1.598179953	-4.683164772
1	0.726211290	2.631321930	-7.196605834
1	0.385861675	4.328098984	-6.828830807
1	1.465576070	3.209832800	-4.882061534
1	-0.179243070	3.723489253	-4.448062577
1	0.849469941	0.881432085	-4.944459869
1	-0.094172799	1.517316381	-3.582177326
6	-1.888823031	1.725231128	-6.404665925
6	-3.067480005	1.144828451	-6.845034732
1	-3.725039559	1.674460386	-7.548025332
1	-3.496639439	0.289267685	-6.315034793
6	-1.211467750	1.070763153	-5.315309479
8	-1.739055216	-0.017505466	-4.843666695
6	-2.270204175	4.183781127	-6.293848996
1	-1.928724405	5.185881155	-6.627124728

1	-3.328013506	4.060500297	-6.603669196
1	-2.244149862	4.156014561	-5.189035880
6	-1.524093953	3.192031164	-8.464093877
1	-0.889991593	2.454196455	-8.995668136
1	-2.569886921	3.067981784	-8.809050634
1	-1.191516498	4.197896613	-8.790024814
6	0.102405284	-4.260355763	-3.399831672
6	1.074280269	-4.200617513	-4.415656954
6	0.131668192	-5.350737786	-2.510038538
6	2.032888994	-5.209845254	-4.570052024
6	1.099146926	-6.362555529	-2.648708199
6	2.024277217	-6.313297447	-3.702048874
9	-0.721460400	-5.473661903	-1.494207546
9	1.133249009	-7.379151342	-1.785672578
9	2.914836754	-7.289692864	-3.853823687
9	2.964610164	-5.116453143	-5.525453884
9	1.126256306	-3.161406594	-5.257408066
6	-3.076144084	4.105154974	-0.780187483
6	-2.022119945	4.624935168	-0.005869473
6	-4.398628190	4.400147850	-0.402700114
6	-2.270778132	5.312712387	1.188904175
6	-4.656611788	5.149869249	0.758597992
6	-3.595845009	5.587255294	1.565369023
9	-1.254854509	5.725281020	1.954144509
9	-3.845633423	6.248906011	2.694723167
9	-5.912100458	5.443564598	1.100952406
9	-5.455010488	3.992575297	-1.106656446
9	-0.748823028	4.452102998	-0.370180283
6	-2.592128961	-2.707511892	-7.945221819
1	-2.083898316	-3.458840275	-8.588909385
1	-2.485943026	-3.051760216	-6.898377706
1	-3.665032626	-2.687358829	-8.206990077
1	-0.216262656	-2.064044489	-7.001021261

TS-B2: Imaginary frequency = - 154.14

15	0.164332085	-1.267022045	-1.443066873
8	1.761288064	-0.887389972	-1.653026726
8	0.248226914	-2.722013593	-0.665284530
6	2.671472053	-1.870162766	-2.022986729
6	3.315059034	-1.750866139	-3.296682384
6	4.207292272	-2.755751686	-3.665040448
6	4.455142646	-3.889170765	-2.847251345
6	5.311200937	-4.944486768	-3.279531228
6	5.503821680	-6.068125707	-2.492187348
6	4.832983560	-6.183449998	-1.246378576
6	4.014491506	-5.161437847	-0.786372696
6	3.816190352	-3.979879328	-1.559624840
6	2.969059271	-2.900316093	-1.123780006
6	2.425457788	-2.886066726	0.261954598
6	3.290542565	-2.900778970	1.411296618
6	4.708650004	-2.794711228	1.309652036
6	5.501557922	-2.764401902	2.448040788
6	4.916111384	-2.855861066	3.738874162
6	3.539051050	-2.951655037	3.870493157
6	2.691446501	-2.958047716	2.722575872
6	1.274249390	-2.974203927	2.850958350
6	0.424937346	-2.886215400	1.749657951
6	1.048417868	-2.831190080	0.462624225

6	3.169523410	-0.566816336	-4.178719183
1	4.751236286	-2.665512534	-4.616590604
1	5.796976688	-4.857854296	-4.263268235
1	6.153787477	-6.883729371	-2.841766260
1	4.957846439	-7.093820786	-0.641448989
1	3.494527700	-5.265171622	0.175781738
1	5.167619694	-2.687459414	0.318342150
1	6.591100597	-2.651053133	2.348285243
1	5.556557421	-2.836219629	4.633239544
1	3.074709428	-3.004491045	4.867552298
1	0.838592751	-3.049696841	3.858981907
6	-1.050035968	-2.838797556	1.904271445
6	3.222318349	0.740621130	-3.651869610
6	3.330193385	1.852605501	-4.494106565
6	3.352616813	1.651736985	-5.880252453
6	3.228164840	0.372596241	-6.439108295
6	3.134891865	-0.729412126	-5.581542012
6	-1.631348167	-2.119225818	2.970769815
6	-3.020082361	-2.055579453	3.129593458
6	-3.836271390	-2.698697994	2.193461686
6	-3.296387949	-3.435194044	1.133558914
6	-1.905928667	-3.511571313	1.004905526
1	3.239059122	0.892609114	-2.567713276
1	3.039185676	-1.738638253	-6.007356400
1	-0.994508861	-1.562776248	3.673559582
1	-1.496347357	-4.090361016	0.171670135
7	-0.366310998	-0.131644514	-0.491183210
15	-1.557998004	0.919648277	-0.455492344
8	-1.278290432	1.987268417	0.769170643
8	-2.861151864	0.093795886	0.147022768
6	-1.393701592	1.622754627	2.095379045
6	-0.187175016	1.460421964	2.844578156
6	-0.314810734	1.177995409	4.201230671
6	-1.583718229	1.004835950	4.819050473
6	-1.681255238	0.672782346	6.203936180
6	-2.909358385	0.421086692	6.793397837
6	-4.090939579	0.483825994	6.008990909
6	-4.032655946	0.822369135	4.664455727
6	-2.787584131	1.113035083	4.029210683
6	-2.672897512	1.455453201	2.631172110
6	-3.864084980	1.588990593	1.745138074
6	-4.954373839	2.457970545	2.103833827
6	-4.883156573	3.383724011	3.187574343
6	-5.971791996	4.175306575	3.521165520
6	-7.184523727	4.083829679	2.787253637
6	-7.268866216	3.232860950	1.698592534
6	-6.158661865	2.421364838	1.316385349
6	-6.202624019	1.619977098	0.147239637
6	-5.107339823	0.879518947	-0.298603967
6	-3.949673635	0.867381819	0.543704707
6	1.139748904	1.504583971	2.183077183
1	0.591011154	1.078766736	4.818749674
1	-0.752995395	0.606833058	6.792588153
1	-2.969979052	0.158564527	7.860096399
1	-5.064402926	0.249318260	6.464815158
1	-4.953821972	0.832581593	4.070912278
1	-3.945183137	3.478249522	3.750857887

1	-5.888363015	4.892661930	4.350926139
1	-8.041801300	4.714504687	3.065046410
1	-8.186318331	3.185728821	1.092671368
1	-7.136497862	1.600426545	-0.432956076
6	-5.199932672	0.158316148	-1.596088077
6	1.475731065	2.462817681	1.201740184
6	2.720644168	2.427393098	0.562669687
6	3.637002390	1.423837040	0.906230897
6	3.342608214	0.470509261	1.888742801
6	2.095237041	0.522023544	2.517316256
6	-5.962085586	0.741891654	-2.636760660
6	-6.213391992	0.064209456	-3.832719276
6	-5.674310856	-1.217522786	-4.001828352
6	-4.855903115	-1.803664506	-3.028864148
6	-4.620965714	-1.110009943	-1.832881075
1	0.758867179	3.236439111	0.900599615
1	1.846373141	-0.253747815	3.254573696
1	-6.352299139	1.761524236	-2.515212249
1	-3.992556535	-1.589018747	-1.073344962
7	-0.566891012	-1.603846567	-2.863170882
7	-1.962712186	1.767145235	-1.752474112
16	-1.302404153	-3.054740776	-3.118803608
8	-2.167963963	-3.491074258	-2.002607089
8	-1.905393050	-3.005597500	-4.466701589
16	-1.543197314	3.336246504	-1.890684025
8	-0.178557506	3.635329449	-1.399821034
8	-1.891387117	3.787829421	-3.254681507
1	3.222580289	0.230799204	-7.526707205
1	3.419260712	2.857945032	-4.064113014
1	4.057882001	-0.314656429	2.155228086
1	2.950076416	3.174356113	-0.205673119
1	-6.821119105	0.530755396	-4.617849291
1	-4.396188704	-2.788305099	-3.179015952
1	-3.938001385	-3.944366631	0.403985064
1	-3.454694250	-1.494330708	3.964176842
1	-0.556575736	-0.276479453	-4.059114685
16	-5.651431017	-2.550469962	2.339981997
16	-6.090021648	-2.150124354	-5.518423465
16	3.591313408	3.087416576	-6.989226825
16	5.247987294	1.334342414	0.041036220
9	2.662790502	4.068582670	-6.047385397
9	2.248254326	2.694135697	-7.885426687
9	3.792653435	4.361908032	-7.989454838
9	4.926108788	3.572196166	-6.179629976
9	4.520942130	2.217659655	-8.024143480
9	4.843723843	-0.107075721	-0.669538429
9	4.674372044	2.137042331	-1.284174806
9	5.745591534	2.757164554	0.677682424
9	5.968126990	0.521044561	1.272410883
9	6.678972172	1.242175832	-0.747371830
9	-6.450587474	-3.505633658	-4.681587781
9	-7.658833182	-1.682498116	-5.464238292
9	-6.451160055	-2.966507977	-6.895042541
9	-5.751764866	-0.840948092	-6.494128085
9	-4.540671520	-2.683173187	-5.710737609
9	-5.861998427	-4.165151336	2.153847132
9	-5.832966026	-2.335824987	0.718412123

9	-5.602939230	-2.735952321	3.971872570
9	-5.572316902	-0.911783134	2.531333215
9	-7.276257961	-2.414311773	2.470842067
6	-0.920945026	-0.342796466	-8.876894097
6	-1.874284356	-1.285851896	-8.310245627
6	-1.720548341	-1.774736178	-7.028929317
6	0.113430181	0.228505464	-8.204281664
1	-0.756923402	-1.705003453	-6.507491325
1	0.372773780	-0.056769726	-7.173849738
1	0.755061614	0.986733092	-8.675596990
1	-2.409414142	-2.532546354	-6.637735796
6	-2.770878524	2.168855495	-6.645455195
6	-1.777196625	3.223121406	-7.185848105
6	-0.842498410	3.784229870	-6.119600354
6	-0.065498980	2.673367107	-5.417284642
1	-1.180226385	2.751393884	-7.996783072
1	-2.357142551	4.044236153	-7.656859405
1	-0.116385081	4.489416825	-6.570888212
1	-1.415298280	4.348086123	-5.360220887
1	0.820198139	2.384306644	-6.019817921
1	0.340004385	2.998935618	-4.438645562
6	-2.023678203	1.070608689	-5.857286357
6	-2.584236252	-0.194687636	-5.680743196
1	-3.602778009	-0.383898794	-6.034387811
1	-2.244146445	-0.883054747	-4.893962853
6	-0.799779092	1.392161114	-5.196711363
8	-0.138966862	0.584178861	-4.401488213
6	-3.786198203	2.803548072	-5.663798943
1	-4.330992702	3.632236277	-6.162874908
1	-4.529803155	2.043373518	-5.347761416
1	-3.301482332	3.190124034	-4.749092343
6	-3.555883531	1.611480524	-7.846289294
1	-2.879361418	1.205325294	-8.619991223
1	-4.284758517	0.829284805	-7.560923246
1	-4.130969039	2.438214992	-8.309782547
6	0.076936095	-4.238402972	-3.274907325
6	0.967010996	-4.070029948	-4.352218658
6	0.238817740	-5.359529948	-2.441618764
6	1.980574690	-5.000200963	-4.613396016
6	1.264322266	-6.291325647	-2.685539546
6	2.107570029	-6.134832788	-3.795681870
9	-0.537839354	-5.583037976	-1.381329398
9	1.425682245	-7.338131880	-1.874703843
9	3.048952034	-7.039594291	-4.050608227
9	2.830190845	-4.807381836	-5.628099371
9	0.891979910	-2.994620784	-5.148062651
6	-2.748242510	4.194858699	-0.816738753
6	-2.394011576	4.893119324	0.350915137
6	-4.099348541	4.193374356	-1.213480612
6	-3.367911914	5.587438963	1.091645263
6	-5.073504074	4.895505893	-0.491199142
6	-4.699580660	5.619713471	0.651472107
9	-3.028965045	6.226138232	2.215415750
9	-5.609726518	6.310479560	1.336749726
9	-6.351440765	4.869773296	-0.885027272
9	-4.505349208	3.507286822	-2.286437831
9	-1.145865164	4.919139878	0.821677838

6	-3.079662036	-1.663797277	-9.126753358
1	-3.499113632	-0.792960231	-9.670337964
1	-2.797294326	-2.413533706	-9.898356727
1	-3.875286242	-2.105344139	-8.497355193
1	-1.108227349	-0.022193508	-9.916672956

TS-B3: Imaginary frequency = - 160.23

15	0.194963427	-1.795243905	-1.372376518
8	1.732259209	-1.322716824	-0.974563534
8	0.187034121	-3.328999973	-0.790065273
6	2.797619422	-2.131362784	-1.359465475
6	3.670169781	-1.648385727	-2.389179112
6	4.773615290	-2.445542637	-2.697819604
6	4.973729669	-3.730213411	-2.133928889
6	6.059617876	-4.558962357	-2.546895108
6	6.194136243	-5.847278280	-2.057017487
6	5.228036766	-6.366374165	-1.154425286
6	4.177934244	-5.574612900	-0.713171744
6	4.040139407	-4.226631121	-1.158471512
6	2.994124586	-3.353469384	-0.694262611
6	2.168605029	-3.719057123	0.491335403
6	2.767923410	-4.092527531	1.753313107
6	4.178325260	-4.120151465	1.976133941
6	4.705263257	-4.482560012	3.207955714
6	3.853220620	-4.837236725	4.286193636
6	2.479441103	-4.788388183	4.116906900
6	1.904618818	-4.401842000	2.868702723
6	0.496349673	-4.261955855	2.732431045
6	-0.091022122	-3.857482599	1.538033267
6	0.776373684	-3.636620474	0.425060337
6	3.499927897	-0.352311371	-3.098434285
1	5.520598548	-2.075346111	-3.413716734
1	6.773905760	-4.160605997	-3.283192212
1	7.026628674	-6.482841638	-2.392808194
1	5.304559245	-7.408823124	-0.811838605
1	3.428551755	-5.990592886	-0.026097508
1	4.857492023	-3.820180106	1.169940073
1	5.796380095	-4.480986187	3.348966072
1	4.283739573	-5.131233508	5.254981482
1	1.802753502	-5.033967565	4.950054043
1	-0.143577901	-4.471614476	3.603091306
6	-1.540572493	-3.555492092	1.451079790
6	2.935366945	0.801060503	-2.506012371
6	2.957449982	2.040420111	-3.160685626
6	3.560709459	2.134553432	-4.418995793
6	4.061102612	1.000810299	-5.071824149
6	4.007082135	-0.231428669	-4.415719119
6	-2.124717049	-2.793523530	2.484443589
6	-3.466677091	-2.406552828	2.437667395
6	-4.235498387	-2.802286251	1.337186179
6	-3.695061795	-3.576305930	0.300942981
6	-2.345815775	-3.945801300	0.358747970
1	2.494703610	0.749928617	-1.504621614
1	4.367298235	-1.125873559	-4.942610450
1	-1.506014934	-2.448075910	3.324031481
1	-1.920847310	-4.520887820	-0.474911417
7	-0.794685121	-0.873565819	-0.550988719
15	-1.250724803	0.643090752	-0.741237217

8	-0.700586261	1.556065288	0.539110164
8	-2.888163914	0.594125451	-0.457469491
6	-1.047366564	1.159793303	1.820658299
6	-0.052012680	0.560799757	2.656693142
6	-0.465837951	0.114713891	3.910560259
6	-1.819624887	0.205844560	4.340067551
6	-2.246591106	-0.350931003	5.582400877
6	-3.581188706	-0.313403933	5.957883586
6	-4.543508085	0.291261711	5.105458539
6	-4.158031245	0.867032734	3.902982650
6	-2.793910373	0.844816396	3.489927860
6	-2.360592664	1.375177569	2.226242877
6	-3.281852436	2.138493839	1.344483606
6	-3.873264091	3.363249580	1.814610069
6	-3.481486071	3.988082747	3.035381082
6	-4.051331892	5.189284416	3.433019695
6	-5.054822273	5.811036241	2.643851058
6	-5.451987735	5.231831627	1.449819863
6	-4.859855389	4.017142895	0.995071123
6	-5.222508826	3.430898883	-0.245957299
6	-4.605807087	2.285313990	-0.744950339
6	-3.575262055	1.689431585	0.052931555
6	1.358123109	0.430414748	2.217452022
1	0.274695067	-0.327705482	4.594380387
1	-1.496290315	-0.829397914	6.231095257
1	-3.899392619	-0.760971497	6.911243449
1	-5.605236844	0.291057301	5.393179065
1	-4.910382103	1.307301929	3.234994072
1	-2.703516412	3.518059751	3.652685930
1	-3.719528540	5.665431158	4.367788870
1	-5.504261534	6.758605383	2.975867953
1	-6.211488072	5.715177505	0.816735503
1	-6.035787369	3.891120688	-0.826262760
6	-5.145622549	1.677222400	-1.985902225
6	2.006734563	1.462368832	1.501202719
6	3.337278171	1.334286448	1.088417847
6	4.035101411	0.165930360	1.418580826
6	3.438614057	-0.856739643	2.160666804
6	2.099422094	-0.724983580	2.542215652
6	-5.526395294	2.491391756	-3.074673183
6	-6.262854608	1.970151997	-4.141741769
6	-6.621312024	0.614861259	-4.114376095
6	-6.202284277	-0.236116227	-3.084154920
6	-5.449919078	0.302159520	-2.032099636
1	1.472223866	2.387543513	1.261235870
1	1.617885535	-1.556579286	3.077002119
1	-5.228461584	3.547779617	-3.089321280
1	-5.153086034	-0.351214034	-1.205354786
7	0.014344519	-1.943751674	-2.979694050
7	-0.707653894	1.370119846	-2.048926008
16	-0.421058523	-3.361282388	-3.716144979
8	-1.495067197	-4.080880521	-3.007931589
8	-0.616069057	-3.028660371	-5.146053146
16	-1.186992976	2.720796579	-2.813589008
8	-0.102280570	3.099462852	-3.744935098
8	-2.565888519	2.606593718	-3.334670803
1	4.493683472	1.076549274	-6.077068135

1	2.512945898	2.919374935	-2.680706541
1	4.000250975	-1.758476398	2.428540994
1	3.819082596	2.141250958	0.522047172
1	-6.564743807	2.616420731	-4.974985232
1	-6.482335202	-1.296710020	-3.066900118
1	-4.303949726	-3.880230525	-0.558453009
1	-3.890175102	-1.792067069	3.240135165
1	-0.876907320	-0.862235168	-3.771141687
16	-5.982191323	-2.267781731	1.249707961
16	-7.706751073	-0.031263187	-5.430879534
16	3.762305504	3.771618541	-5.207464284
16	5.760161019	-0.041018550	0.855210982
9	2.189427090	4.167334100	-4.984070376
9	3.405576204	3.180927495	-6.705381380
9	3.967564526	5.234128858	-5.912497418
9	4.142884756	4.479448632	-3.776716769
9	5.356093567	3.487131781	-5.488600509
9	5.482224750	-1.593044025	0.358334717
9	5.399117427	0.497073163	-0.655807180
9	6.159711414	1.482628425	1.306306158
9	6.238817393	-0.607792005	2.320904672
9	7.302280835	-0.233311200	0.348058834
9	-8.777572694	-0.637801416	-4.354648825
9	-8.567321889	1.359319341	-5.518178506
9	-8.657210737	-0.616316405	-6.629834089
9	-6.695198547	0.529429452	-6.619946942
9	-6.928904108	-1.494762904	-5.461853528
9	-6.525924048	-3.811144938	1.271171773
9	-5.986343979	-2.301768202	-0.404607239
9	-6.131377969	-2.183668349	2.881416590
9	-5.542917634	-0.671310435	1.212070452
9	-7.536930040	-1.771644375	1.156427617
6	-2.606329917	-1.823153721	-8.563078093
6	-3.840824381	-1.730284662	-7.793474408
6	-3.840132653	-1.705992349	-6.412218604
6	-1.355381867	-1.970128548	-8.054495471
1	-2.945742749	-1.981546690	-5.833348395
1	-1.160875426	-2.140332994	-6.984946761
1	-0.484088784	-1.987381575	-8.727554795
1	-4.798952217	-1.727032878	-5.879595782
6	-1.717164701	1.785665956	-7.347132848
6	-0.258962002	1.482017076	-7.763018684
6	0.706566041	1.449712949	-6.579016293
6	0.292479058	0.430654979	-5.515919108
1	-0.245278794	0.505612971	-8.294473432
1	0.071466383	2.249129025	-8.494394005
1	1.732843978	1.222621530	-6.924436993
1	0.760744524	2.449305101	-6.108365050
1	0.651377847	-0.597040661	-5.756243958
1	0.743252143	0.667917107	-4.529367812
6	-2.152867829	0.826237592	-6.232979425
6	-3.484487861	0.503282420	-5.975882866
1	-3.763852327	0.177778201	-4.965523683
1	-4.296111881	0.906118675	-6.594574637
6	-1.183955529	0.297163535	-5.328506161
8	-1.624784938	-0.410930275	-4.328144416
6	-1.824007720	3.220467933	-6.771062051

1	-1.498555207	3.962858728	-7.529476632
1	-2.873134967	3.442331287	-6.489392951
1	-1.212682046	3.351929110	-5.858777395
6	-2.620979080	1.684895854	-8.586844572
1	-2.600919801	0.666892185	-9.020419127
1	-3.673353293	1.949576018	-8.360612478
1	-2.266778782	2.394672383	-9.361370176
6	1.079583872	-4.384497127	-3.683049353
6	2.173421637	-3.983927411	-4.474786194
6	1.140093352	-5.623888175	-3.018335900
6	3.288523281	-4.815119748	-4.642069000
6	2.255283618	-6.464112474	-3.187357781
6	3.310565297	-6.077349611	-4.027135030
9	0.176744853	-6.047564171	-2.203035441
9	2.317184910	-7.633075017	-2.545389045
9	4.349923683	-6.889462129	-4.204117949
9	4.332498710	-4.404163328	-5.369143893
9	2.189906592	-2.793157531	-5.080967674
6	-1.221785509	4.010089803	-1.518011872
6	-0.120650881	4.146124989	-0.655283889
6	-2.304401303	4.894524782	-1.356820128
6	-0.156592754	5.016697805	0.441531650
6	-2.333194109	5.806130066	-0.286668906
6	-1.271196372	5.851471982	0.629485524
9	0.867940296	5.060570381	1.300717281
9	-1.314273906	6.688619336	1.663229601
9	-3.360156087	6.648030749	-0.153398744
9	-3.344840798	4.915461955	-2.191595350
9	0.982716865	3.405065310	-0.822618280
6	-5.113723323	-1.558892350	-8.576311585
1	-5.086523218	-0.610698000	-9.157655508
1	-5.225251648	-2.375187136	-9.321080835
1	-6.007725767	-1.538260087	-7.930021173
1	-2.718810893	-1.731267730	-9.658255715

TS-B4: Imaginary frequency =   - 154.06

15	0.195623879	-1.319491889	-1.412256536
8	1.793247524	-0.940770934	-1.618626333
8	0.277111520	-2.769141327	-0.624212674
6	2.705035308	-1.925692072	-1.978804704
6	3.353240611	-1.814441969	-3.250871048
6	4.247177883	-2.821367328	-3.609391382
6	4.492488006	-3.949297906	-2.783311343
6	5.350630682	-5.006992153	-3.205539252
6	5.540895494	-6.125358355	-2.410167777
6	4.865491746	-6.232892017	-1.166124664
6	4.044803140	-5.208315530	-0.715837843
6	3.848814134	-4.031911545	-1.497495434
6	2.999647772	-2.949888949	-1.071826584
6	2.451062814	-2.926695702	0.311830351
6	3.312059367	-2.933549448	1.464330689
6	4.730508251	-2.827869868	1.367080308
6	5.519335935	-2.789794224	2.508070172
6	4.929302194	-2.872708901	3.797386499
6	3.551796547	-2.967865219	3.924705771
6	2.708315690	-2.982124247	2.773826660
6	1.290685742	-2.997689292	2.897263596
6	0.445270882	-2.917267522	1.792390822

6	1.073293140	-2.870695747	0.507222870
6	3.210516593	-0.636200028	-4.141099267
1	4.794599727	-2.737120751	-4.559495028
1	5.840009471	-4.926532336	-4.188011721
1	6.192546342	-6.942897762	-2.752002740
1	4.988535273	-7.139237195	-0.554811946
1	3.521305102	-5.306053324	0.245029008
1	5.193016812	-2.727186987	0.376724098
1	6.609209731	-2.676921117	2.411449173
1	5.566541537	-2.846952228	4.693884003
1	3.083885126	-3.014100823	4.920422898
1	0.851456567	-3.066481970	3.904217446
6	-1.030232502	-2.869292115	1.941651866
6	3.260152721	0.674701783	-3.622579934
6	3.370749444	1.781251976	-4.471587591
6	3.399072983	1.571352481	-5.856290335
6	3.277882746	0.288523232	-6.407289381
6	3.181821129	-0.807913255	-5.542968358
6	-1.615299380	-2.142837294	3.001407035
6	-3.004568577	-2.078722775	3.155238993
6	-3.817451412	-2.728350005	2.220732388
6	-3.273822050	-3.471620137	1.167488959
6	-1.882912855	-3.548332588	1.043936717
1	3.272151976	0.833846717	-2.539390527
1	3.088592155	-1.819916416	-5.962656075
1	-0.980911497	-1.581375492	3.702446423
1	-1.470361718	-4.132490055	0.215929085
7	-0.339064123	-0.177826860	-0.470254605
15	-1.531418868	0.872994166	-0.444399888
8	-1.255588809	1.948566382	0.774185170
8	-2.835619619	0.050181679	0.159984705
6	-1.375187624	1.593021048	2.102457248
6	-0.170977259	1.436584389	2.856633037
6	-0.302722123	1.163455065	4.214795963
6	-1.573469919	0.993666117	4.829752720
6	-1.675202567	0.671062065	6.216569097
6	-2.905003380	0.422466552	6.803799401
6	-4.084098801	0.478907025	6.015184364
6	-4.021763975	0.808340450	4.668569636
6	-2.774866902	1.095606225	4.035336721
6	-2.655978696	1.428614466	2.635353998
6	-3.844516635	1.555401267	1.744802030
6	-4.936501876	2.425976920	2.094318513
6	-4.869320276	3.358921968	3.172132618
6	-5.959582527	4.151828916	3.497145814
6	-7.169957565	4.054597333	2.760087044
6	-7.250284307	3.196420697	1.676791494
6	-6.138305438	2.383251567	1.303390552
6	-6.178006455	1.574183578	0.139391390
6	-5.080784322	0.831647721	-0.298162635
6	-3.925875131	0.825743683	0.547974272
6	1.157988460	1.477031834	2.199007348
1	0.601228544	1.069033518	4.835807634
1	-0.748789720	0.609865936	6.808634721
1	-2.968857301	0.167210829	7.872071766
1	-5.058845706	0.246771963	6.469480137
1	-4.941033621	0.813826529	4.072029409

1	-3.933166847	3.457938987	3.737667064
1	-5.879259692	4.874715655	4.322399831
1	-8.028561966	4.686434292	3.031055976
1	-8.165813695	3.144611818	1.068349852
1	-7.110012958	1.550210246	-0.443632549
6	-5.168412600	0.102340626	-1.591450771
6	1.496553727	2.429168006	1.212637518
6	2.743461110	2.390297938	0.577703780
6	3.659159081	1.389312431	0.930356340
6	3.362116869	0.442006583	1.917881155
6	2.112816061	0.496982849	2.542355591
6	-5.927671696	0.678790237	-2.638198768
6	-6.173763290	-0.006165996	-3.831093339
6	-5.632326862	-1.288126802	-3.990833337
6	-4.816793823	-1.867554393	-3.011488032
6	-4.587090208	-1.166629375	-1.818730697
1	0.780240043	3.200518023	0.904436454
1	1.861895071	-0.274199737	3.283737102
1	-6.319607179	1.698616616	-2.524041337
1	-3.960847659	-1.640392908	-1.054148066
7	-0.529748511	-1.666512262	-2.832919449
7	-1.933508186	1.711986200	-1.747716586
16	-1.263474887	-3.119617322	-3.081103611
8	-2.133274197	-3.548285595	-1.965216896
8	-1.861164367	-3.080548426	-4.431671423
16	-1.515158895	3.280567231	-1.894974575
8	-0.152071160	3.584122143	-1.402490532
8	-1.860247405	3.723156976	-3.262710736
1	3.276885792	0.139634413	-7.493950773
1	3.457265827	2.789425919	-4.047761861
1	4.076861256	-0.341214290	2.191398228
1	2.975066159	3.132556503	-0.194515769
1	-6.779261192	0.455006095	-4.621094637
1	-4.355310632	-2.852488802	-3.154174327
1	-3.912849955	-3.985907811	0.439233671
1	-3.442113408	-1.512150839	3.984659813
1	-0.515892588	-0.345878144	-4.036782383
16	-5.633128455	-2.579641980	2.360218057
16	-6.041019856	-2.229712819	-5.503762264
16	3.641195300	2.999840995	-6.973723767
16	5.272781828	1.295135101	0.070544735
9	2.709384316	3.987056728	-6.041456077
9	2.301194670	2.600348734	-7.871839836
9	3.845627098	4.267806268	-7.981592385
9	4.973164051	3.490139251	-6.162837135
9	4.574480712	2.123618748	-7.999888467
9	4.871047202	-0.150610787	-0.632605734
9	4.702876748	2.089684184	-1.261165497
9	5.768038286	2.721908119	0.700148241
9	5.989492858	0.489444319	1.308893955
9	6.706136595	1.198715649	-0.713026127
9	-6.401206737	-3.581687283	-4.661094324
9	-7.611095775	-1.765531906	-5.457128940
9	-6.395741296	-3.053982239	-6.877340619
9	-5.702626831	-0.924665772	-6.484905787
9	-4.489864276	-2.760441602	-5.688272025
9	-5.842830542	-4.195353593	2.182169109

9	-5.809060669	-2.373852897	0.736879277
9	-5.590179148	-2.756220679	3.993246773
9	-5.554996645	-0.939918678	2.542953442
9	-7.258415663	-2.443078949	2.484727237
6	-0.858119004	-0.443129649	-8.855879284
6	-1.810763245	-1.384993356	-8.286133451
6	-1.660266140	-1.864664886	-7.000917429
6	0.171871918	0.136115549	-8.183330272
1	-0.698693241	-1.788607177	-6.476509361
1	0.428042322	-0.141217545	-7.149874046
1	0.812860968	0.893334990	-8.657173327
1	-2.348391164	-2.621696254	-6.607011459
6	-2.721812505	2.079479326	-6.648041830
6	-1.727520272	3.131997502	-7.190563328
6	-0.798930542	3.701669296	-6.123528092
6	-0.023582234	2.596956193	-5.409710505
1	-1.125856871	2.656130097	-7.995611835
1	-2.306652960	3.949045324	-7.669583358
1	-0.071719136	4.404951077	-6.576034547
1	-1.376392812	4.269775329	-5.370840914
1	0.866535790	2.306469764	-6.004932130
1	0.375246770	2.929880480	-4.430806948
6	-1.976236340	0.987492537	-5.849856033
6	-2.534825740	-0.278042972	-5.668671274
1	-3.550258563	-0.472903577	-6.027952451
1	-2.198022842	-0.960468819	-4.875334224
6	-0.756342796	1.315553799	-5.185120403
8	-0.097944133	0.513502974	-4.381906812
6	-3.742310212	2.718433986	-5.674513504
1	-4.286098073	3.543440893	-6.180730453
1	-4.486112378	1.958935388	-5.357331080
1	-3.262176965	3.111072392	-4.759957415
6	-3.500832857	1.513416719	-7.848698679
1	-2.820503825	1.102738812	-8.616681829
1	-4.230292530	0.732513577	-7.561317985
1	-4.074638023	2.336487573	-8.320224440
6	0.116879971	-4.303893652	-3.223150148
6	1.011051337	-4.143343561	-4.298266627
6	0.275603914	-5.418905226	-2.381094256
6	2.025475283	-5.075510936	-4.548907517
6	1.301963682	-6.352471587	-2.614352638
6	2.149324863	-6.204152715	-3.722467523
9	-0.505029090	-5.634692977	-1.322128013
9	1.460287035	-7.393291698	-1.795250214
9	3.091549297	-7.110823475	-3.967291346
9	2.878803885	-4.890266411	-5.561876256
9	0.939116792	-3.073656573	-5.102087748
6	-2.723729534	4.145184731	-0.829860135
6	-2.373211645	4.850789700	0.334497453
6	-4.073641615	4.140888755	-1.230694563
6	-3.349497415	5.549562083	1.067854791
6	-5.050132082	4.847323063	-0.515787078
6	-4.679822655	5.578797681	0.623425225
9	-3.014098148	6.195377077	2.188625605
9	-5.592172795	6.273704731	1.301551909
9	-6.326845761	4.818841170	-0.913336940
9	-4.476281331	3.448011709	-2.300552618

9	-1.126501203	4.880085685	0.808874377
6	-3.012384934	-1.771553785	-9.104141695
1	-3.432436378	-0.905332837	-9.654599781
1	-2.725468944	-2.525385495	-9.870054027
1	-3.808889371	-2.211296338	-8.474568324
1	-1.042480606	-0.130397882	-9.898565012

TS-B5: Imaginary frequency = - 151.74

15	0.078845221	-1.817607514	-1.374526248
8	1.639650132	-1.380929781	-1.019534250
8	0.051089128	-3.349250490	-0.781006134
6	2.692490575	-2.215108985	-1.378564614
6	3.596168536	-1.760120608	-2.394942104
6	4.688406795	-2.583423403	-2.674372059
6	4.852938549	-3.865343741	-2.093935619
6	5.927111720	-4.721941900	-2.480127548
6	6.029351511	-6.004860595	-1.969087949
6	5.042384231	-6.490221446	-1.069998492
6	4.002498512	-5.671317575	-0.655478334
6	3.896296915	-4.328627076	-1.125259303
6	2.861365660	-3.428651284	-0.689350203
6	2.029315726	-3.752580188	0.503137243
6	2.627089139	-4.107476401	1.771861118
6	4.037542953	-4.158857805	1.991096380
6	4.561845129	-4.503602621	3.229098755
6	3.707298628	-4.815927845	4.318351054
6	2.334282268	-4.741351267	4.152821430
6	1.763095134	-4.371251890	2.897960005
6	0.358476438	-4.199872222	2.764599811
6	-0.225502233	-3.809664670	1.563770716
6	0.638943297	-3.640821431	0.439557267
6	3.474332598	-0.463527864	-3.112386428
1	5.457168163	-2.235541361	-3.378047795
1	6.658825210	-4.349184736	-3.212711334
1	6.852927613	-6.662146010	-2.284497109
1	5.094106883	-7.528287614	-0.709914527
1	3.236217202	-6.061387782	0.028192037
1	4.720200478	-3.891184626	1.176797031
1	5.653360964	-4.520729752	3.365960966
1	4.135558834	-5.096806721	5.292025421
1	1.655607551	-4.952519660	4.993752904
1	-0.281939596	-4.371381421	3.643184451
6	-1.667827113	-3.473241133	1.487874126
6	2.906973092	0.698498889	-2.541752245
6	2.970302770	1.935212190	-3.195961528
6	3.622788159	2.018007302	-4.429998639
6	4.135398242	0.877634030	-5.062432123
6	4.034181793	-0.352008539	-4.408796154
6	-2.213481824	-2.667463664	2.509173397
6	-3.551054279	-2.264869658	2.486173943
6	-4.355561600	-2.690219686	1.422927915
6	-3.851061244	-3.498830271	0.394698394
6	-2.504119273	-3.881356614	0.426164503
1	2.428254309	0.655228644	-1.557973645
1	4.397254124	-1.253050696	-4.922308617
1	-1.567382177	-2.302876171	3.319615409
1	-2.106088732	-4.485420180	-0.400624701
7	-0.846627579	-0.879228913	-0.495130866

15	-1.289513196	0.631521941	-0.736301577
8	-0.718053717	1.591909595	0.498531205
8	-2.921978929	0.612781490	-0.426398235
6	-1.055530612	1.241738973	1.797391881
6	-0.061438861	0.642477262	2.634461986
6	-0.465544459	0.237363433	3.905200284
6	-1.811046733	0.365372481	4.350637194
6	-2.230768479	-0.153792784	5.611572391
6	-3.559945041	-0.087387773	6.001662258
6	-4.523605087	0.508796365	5.144955286
6	-4.144226255	1.049743669	3.924463039
6	-2.785149874	0.999606857	3.496851736
6	-2.359565773	1.491038227	2.214237031
6	-3.290718053	2.225686333	1.318960510
6	-3.886649886	3.462395652	1.750251711
6	-3.479632132	4.138122211	2.938215265
6	-4.056299983	5.347390448	3.300348298
6	-5.081355649	5.926801604	2.506776515
6	-5.493444522	5.297715999	1.343302350
6	-4.896569165	4.072595925	0.924511089
6	-5.276449981	3.434333764	-0.285882023
6	-4.655022743	2.277558893	-0.749241002
6	-3.603657475	1.724677805	0.052178701
6	1.338389842	0.464842050	2.179277086
1	0.275831373	-0.203741059	4.588923346
1	-1.479658938	-0.627819536	6.262549377
1	-3.873142087	-0.506620106	6.969450394
1	-5.582049342	0.529689349	5.443828015
1	-4.899002909	1.482457656	3.254336997
1	-2.685229656	3.699783636	3.558184823
1	-3.713281751	5.862843856	4.209886602
1	-5.535732708	6.881489964	2.810521578
1	-6.270270053	5.748075058	0.706778788
1	-6.103150552	3.864526903	-0.870443319
6	-5.171267923	1.614063551	-1.969550866
6	2.007387578	1.464574101	1.437061400
6	3.331534846	1.294207105	1.018592026
6	4.001632274	0.115400896	1.368336430
6	3.382726144	-0.878920741	2.130381252
6	2.050178321	-0.705034706	2.517781690
6	-5.558920875	2.374320072	-3.093604780
6	-6.187893885	1.773493537	-4.187448238
6	-6.440428010	0.394638384	-4.144208076
6	-6.042617388	-0.394552371	-3.058648373
6	-5.395193452	0.223187520	-1.982802306
1	1.495171352	2.398583208	1.183121715
1	1.550160602	-1.513771577	3.070293908
1	-5.332551892	3.448382507	-3.124688634
1	-5.104844705	-0.384335910	-1.120336427
7	-0.180125293	-1.962564726	-2.969034180
7	-0.755529795	1.286807331	-2.089601449
16	-0.560268423	-3.398220638	-3.672044103
8	-1.673115767	-4.098232942	-3.004925253
8	-0.675251854	-3.111090280	-5.123808676
16	-1.245903631	2.627842542	-2.859146259
8	-0.216138285	2.966046615	-3.866361779
8	-2.661182602	2.517309733	-3.277436839

1	4.601477690	0.945062232	-6.053123040
1	2.520159905	2.821129700	-2.734907272
1	3.921935663	-1.790466291	2.410843702
1	3.830093790	2.077238004	0.433483686
1	-6.479780049	2.372865524	-5.058558315
1	-6.250879685	-1.471026166	-3.026944282
1	-4.488223146	-3.823446054	-0.436266105
1	-3.944867023	-1.618595145	3.278784277
1	-0.237900575	-0.719807476	-4.262828617
16	-6.102124773	-2.149958546	1.377468433
16	-7.325559371	-0.382661511	-5.534382153
16	3.826256516	3.647153458	-5.232666950
16	5.721634957	-0.141976773	0.810277307
9	2.238235285	4.021006527	-5.049629772
9	3.507073307	3.029107297	-6.733437355
9	4.020157480	5.101266597	-5.955817476
9	4.163710699	4.381912032	-3.805905000
9	5.427549780	3.383070497	-5.479577117
9	5.406875502	-1.694747305	0.339282724
9	5.375863131	0.380120784	-0.709545153
9	6.157243076	1.378962782	1.236986008
9	6.185275459	-0.695277274	2.285969917
9	7.259448524	-0.379681176	0.308853710
9	-8.439889788	-1.062266165	-4.550129339
9	-8.297774448	0.916836882	-5.754425686
9	-8.090117046	-1.085424837	-6.801891296
9	-6.251616857	0.242344343	-6.631248726
9	-6.414174263	-1.763631435	-5.437013807
9	-6.650481499	-3.689692770	1.461467202
9	-6.162474204	-2.230593325	-0.272895571
9	-6.196121278	-2.019487979	3.010511168
9	-5.657485004	-0.557457474	1.280839139
9	-7.657031868	-1.649236435	1.323232383
6	-2.051340118	-1.694043910	-8.430869238
6	-3.271476926	-1.848395166	-7.652000626
6	-3.245112508	-1.946896036	-6.272964061
6	-0.783365359	-1.617913055	-7.941432490
1	-2.333057730	-2.290544335	-5.767192424
1	-0.539570590	-1.819930975	-6.889036141
1	0.060438730	-1.443162831	-8.627190282
1	-4.193521746	-2.053015474	-5.728524032
6	-2.120873565	1.865852248	-7.104096212
6	-0.980736420	2.218789265	-8.086163537
6	0.316254164	2.571187128	-7.371130954
6	0.796732777	1.380367748	-6.554322129
1	-0.810468245	1.351258943	-8.761683347
1	-1.320947024	3.058530135	-8.727190835
1	1.108452041	2.869464384	-8.085082498
1	0.168488625	3.435779286	-6.694183908
1	1.299905107	0.625700142	-7.200852543
1	1.553690748	1.667490309	-5.804822026
6	-1.665829620	0.786951884	-6.083457793
6	-2.604958887	0.083162131	-5.330300409
1	-2.299532246	-0.477903763	-4.434859071
1	-3.661481561	0.379062657	-5.358957830
6	-0.266085096	0.630574765	-5.809692579
8	0.281540388	-0.147510631	-4.915883199

6	-2.539774119	3.131807634	-6.319679695
1	-2.837995980	3.926582875	-7.034802608
1	-3.395483718	2.917177750	-5.651631622
1	-1.727652993	3.512816935	-5.675177083
6	-3.329365050	1.413883542	-7.941383005
1	-3.057530546	0.597624113	-8.633747488
1	-4.185821054	1.083866346	-7.325388312
1	-3.677299028	2.268529680	-8.556183555
6	0.925363403	-4.441383381	-3.574338511
6	2.035065335	-4.081547213	-4.364528243
6	0.941913078	-5.677374686	-2.901762917
6	3.119789730	-4.953404568	-4.525669328
6	2.028972606	-6.555266307	-3.060361643
6	3.097395154	-6.211316225	-3.902212281
9	-0.039718303	-6.062359178	-2.088394770
9	2.052705285	-7.719319978	-2.406227123
9	4.110819860	-7.058434642	-4.067751697
9	4.176886367	-4.585698647	-5.257261078
9	2.090089399	-2.897173805	-4.977871416
6	-1.203639234	3.956740990	-1.603542012
6	-0.072861093	4.096111778	-0.781537569
6	-2.273336276	4.853934730	-1.420134200
6	-0.060348845	4.988823589	0.297791366
6	-2.256708895	5.782224879	-0.364088596
6	-1.161619867	5.834428487	0.512040605
9	0.993697749	5.037065243	1.120438421
9	-1.161911799	6.688750530	1.532425202
9	-3.272097285	6.633188989	-0.205193799
9	-3.342064641	4.871835173	-2.217863118
9	1.012110581	3.332170234	-0.966313572
6	-4.566042830	-1.845277234	-8.418563986
1	-4.669450341	-0.930938376	-9.040746437
1	-4.581311323	-2.704326193	-9.123595587
1	-5.446993857	-1.917073278	-7.759424999
1	-2.194780775	-1.564313752	-9.518725271

TS-B6: Imaginary frequency = - 165.17

15	0.342026883	-1.593032878	-1.398674396
8	1.946391995	-1.288910865	-1.127551771
8	0.213250291	-3.131603285	-0.800429587
6	2.892996889	-2.218349468	-1.528454932
6	3.760814896	-1.877379902	-2.616712690
6	4.725936703	-2.820480812	-2.969926358
6	4.814629365	-4.093103503	-2.347249575
6	5.762283398	-5.067096458	-2.782410529
6	5.805340609	-6.324240748	-2.203114937
6	4.883779690	-6.664324438	-1.177290885
6	3.961769907	-5.734497146	-0.719479960
6	3.916152390	-4.419703640	-1.269682788
6	2.993667195	-3.416391310	-0.810913848
6	2.195267915	-3.608716058	0.432432508
6	2.833720989	-3.878879052	1.696111449
6	4.251383040	-3.911303099	1.858557650
6	4.822255570	-4.138455181	3.102913818
6	4.006919035	-4.357852367	4.244620292
6	2.627005020	-4.310596012	4.125402689
6	2.006922104	-4.049153278	2.866722411
6	0.596933323	-3.893359991	2.762649958

6	-0.026989257	-3.555441751	1.563518794
6	0.809245329	-3.440291833	0.408660347
6	3.675022376	-0.582036697	-3.338272218
1	5.451574145	-2.580590113	-3.760342743
1	6.446722383	-4.803824567	-3.603112238
1	6.532529262	-7.070795480	-2.554969949
1	4.892760819	-7.677950202	-0.749778865
1	3.244941852	-6.010171997	0.066285507
1	4.895261309	-3.711015256	0.993221575
1	5.917612715	-4.134462252	3.204951844
1	4.472233492	-4.546672889	5.223535441
1	1.983433496	-4.453275865	5.007434604
1	-0.015231811	-4.028860181	3.667327023
6	-1.483173027	-3.269405815	1.512631866
6	3.257428787	0.617217661	-2.714479151
6	3.199141180	1.823083182	-3.420308251
6	3.580748803	1.839945128	-4.765178689
6	4.017533003	0.679678772	-5.417025575
6	4.050267149	-0.521289098	-4.701041295
6	-2.073865552	-2.543904011	2.570872343
6	-3.443260360	-2.262707158	2.588442607
6	-4.232514290	-2.707035384	1.522251330
6	-3.681551468	-3.416995743	0.447897310
6	-2.308634652	-3.694793436	0.446959258
1	2.969833598	0.617732849	-1.658836961
1	4.328633800	-1.443992346	-5.229616493
1	-1.447827224	-2.158771648	3.388409293
1	-1.886486193	-4.240979330	-0.405370954
7	-0.372469644	-0.575321366	-0.415112752
15	-1.288065596	0.700895071	-0.559620604
8	-0.997301279	1.743372495	0.693714409
8	-2.804127412	0.162051232	-0.178333533
6	-1.334736257	1.342917663	1.980049894
6	-0.284012140	0.899017061	2.842192131
6	-0.637693175	0.518151654	4.133129009
6	-1.988620943	0.520380594	4.576692539
6	-2.329234752	0.064937888	5.885554439
6	-3.652216031	-0.020155030	6.288682850
6	-4.687567199	0.346840575	5.389292863
6	-4.389282237	0.815024281	4.117504639
6	-3.037929249	0.931039655	3.674406193
6	-2.680377295	1.373110106	2.347322653
6	-3.724987345	1.747953210	1.352928357
6	-4.689538461	2.773623398	1.637927144
6	-4.556153640	3.677404409	2.730947180
6	-5.506084275	4.663197570	2.956325529
6	-6.642205994	4.776911006	2.112136521
6	-6.793474537	3.917227269	1.035445082
6	-5.818677447	2.913843369	0.757487170
6	-5.929670490	2.068243493	-0.376100685
6	-4.939956968	1.159186441	-0.751233899
6	-3.819982353	1.054203490	0.139199589
6	1.117739421	0.778943348	2.370715656
1	0.148547681	0.193431564	4.831830580
1	-1.515871121	-0.235128151	6.564436201
1	-3.902662215	-0.384341259	7.296208701
1	-5.739499898	0.246907566	5.695356158

1	-5.203869571	1.063385434	3.427553609
1	-3.677125329	3.598322723	3.386121967
1	-5.374455911	5.365898902	3.792646740
1	-7.393675226	5.556490659	2.307242430
1	-7.660739782	4.008674731	0.363351599
1	-6.840751615	2.156644328	-0.983601943
6	-5.102171394	0.349159252	-1.990964972
6	1.763548716	1.802892540	1.645393100
6	3.088461234	1.665455870	1.216373599
6	3.776239891	0.483577884	1.522601602
6	3.170920640	-0.549555477	2.245024575
6	1.844029909	-0.395423842	2.658780124
6	-6.006103616	0.785694434	-2.990877760
6	-6.324676330	-0.003006466	-4.098931360
6	-5.695191366	-1.245057021	-4.246485554
6	-4.730967219	-1.680670995	-3.334113070
6	-4.449862160	-0.887987678	-2.213557449
1	1.231784365	2.732975298	1.419889406
1	1.355029559	-1.225315498	3.188436373
1	-6.475870357	1.775053110	-2.911527691
1	-3.720902886	-1.274640821	-1.495536343
7	-0.000830559	-1.659001121	-2.962079470
7	-1.120356994	1.563295075	-1.900783216
16	-0.812640473	-2.895494440	-3.633628613
8	-1.959608334	-3.371816955	-2.822915238
8	-1.076033470	-2.564374331	-5.055666509
16	-1.755275207	2.980928395	-2.299317921
8	-0.982153554	3.470671083	-3.499182966
8	-3.224655401	2.996716489	-2.434796738
1	4.297761971	0.701164518	-6.477360530
1	2.844106034	2.733136696	-2.921782301
1	3.716626005	-1.471178850	2.477259219
1	3.569319524	2.478578633	0.658379831
1	-7.052755285	0.351290781	-4.838788784
1	-4.184112149	-2.623551437	-3.467025451
1	-4.306288278	-3.756221659	-0.387789356
1	-3.881256600	-1.698566841	3.419893169
1	-0.242049833	2.397684672	-4.369376729
16	-6.024872141	-2.347420774	1.541945762
16	-6.127259245	-2.305889724	-5.666512889
16	3.446740396	3.391463907	-5.714169892
16	5.503168449	0.272578347	0.963207345
9	2.011927397	3.792723783	-5.012834022
9	2.623669156	2.630343609	-6.935051983
9	3.318817691	4.769792787	-6.585080441
9	4.242456489	4.248432943	-4.568962042
9	4.859342687	3.109590939	-6.500486428
9	5.147637781	-1.156769484	0.216545350
9	5.220290658	1.070399607	-0.447515832
9	5.966371500	1.679713436	1.663690598
9	5.919040952	-0.548213815	2.323663350
9	7.045726001	0.076860825	0.458642888
9	-5.992562440	-3.674672891	-4.780154178
9	-7.728090607	-2.223025894	-5.316490018
9	-6.516678418	-3.249602876	-6.945094433
9	-6.302609536	-1.006865385	-6.685818470
9	-4.551280244	-2.445652806	-6.130099001

9	-6.405875338	-3.922698243	1.302800123
9	-6.069267930	-2.087770855	-0.079455423
9	-6.119723361	-2.566046844	3.168300437
9	-5.762233598	-0.734245133	1.782233058
9	-7.627841912	-2.019674950	1.566641826
6	-3.654710317	0.708119715	-6.100290274
6	-4.015510345	2.081655258	-6.390775226
6	-3.350583897	3.143520203	-5.807436682
6	-2.711284033	0.295008910	-5.210695108
1	-2.815694493	3.022433427	-4.860443432
1	-2.224904606	0.981940826	-4.506339693
1	-2.463110861	-0.771827937	-5.108549809
1	-3.639521539	4.174697738	-6.064517300
6	-0.717247912	1.278667533	-8.374724907
6	-0.372043336	-0.228687833	-8.394277490
6	0.895277794	-0.556876441	-7.612567893
6	0.696095083	-0.173568859	-6.153684001
1	-1.220154201	-0.796527689	-7.953772285
1	-0.285753901	-0.553868469	-9.451984589
1	1.125639908	-1.638966348	-7.671343988
1	1.768863364	-0.019620303	-8.039309040
1	0.031518816	-0.921945966	-5.660603791
1	1.622544210	-0.206965008	-5.552653081
6	-0.683050804	1.851081518	-6.935838077
6	-1.189723244	3.123837335	-6.680801484
1	-0.907237288	3.674955839	-5.773395214
1	-1.536463728	3.739163844	-7.522074751
6	0.051614305	1.146474555	-5.919348438
8	0.258496703	1.574983611	-4.707529072
6	0.323473188	2.069504243	-9.205965210
1	0.362044958	1.677709852	-10.243560854
1	0.052235632	3.143333497	-9.256743991
1	1.337795090	2.014817651	-8.769481176
6	-2.093888012	1.447410072	-9.040793230
1	-2.853923534	0.815302741	-8.547088874
1	-2.449879583	2.496200136	-9.033004273
1	-2.028697483	1.128930079	-10.100814186
6	0.439315469	-4.227753560	-3.677624099
6	1.585302455	-4.039567691	-4.474300248
6	0.328764377	-5.418435223	-2.936958725
6	2.587841712	-5.015001460	-4.554759926
6	1.337715693	-6.397198968	-2.999962448
6	2.449645994	-6.212051779	-3.834674404
9	-0.702517117	-5.670177459	-2.130297738
9	1.237443276	-7.512376236	-2.272094363
9	3.384678691	-7.156240250	-3.917819954
9	3.673587435	-4.804657262	-5.307565629
9	1.775570631	-2.907615391	-5.162060970
6	-1.287209759	4.125343263	-0.975988168
6	0.077565659	4.251655949	-0.656695680
6	-2.239010084	4.804939027	-0.192664591
6	0.492085239	5.013830620	0.441685660
6	-1.827996032	5.567750147	0.916703449
6	-0.464642512	5.671956217	1.234219811
9	1.788264814	5.083479255	0.765397191
9	-0.077034926	6.387326555	2.286701707
9	-2.731595665	6.199929422	1.667024071

9	-3.542191114	4.748737307	-0.442017612
9	1.011172793	3.583455003	-1.340892018
6	-5.094019916	2.298403944	-7.416354574
1	-4.881560299	1.753059349	-8.359472236
1	-6.052152544	1.881844727	-7.037994920
1	-5.246588188	3.369290781	-7.648119273
1	-4.160272367	-0.062764260	-6.701109269

Regio-isomeric TS structures:

TS-C1: Imaginary frequency = - 165.14

15	0.056798000	-1.233419000	-1.847041000
8	1.702093000	-1.042783000	-1.871853000
8	-0.197856000	-2.544964000	-0.864085000
6	2.560525000	-2.133026000	-1.870837000
6	3.444930000	-2.327910000	-2.984725000
6	4.304976000	-3.424694000	-2.931657000
6	4.277362000	-4.370638000	-1.874935000
6	5.096734000	-5.537289000	-1.900434000
6	5.004977000	-6.488256000	-0.896916000
6	4.083364000	-6.307100000	0.167208000
6	3.294867000	-5.166873000	0.236270000
6	3.378726000	-4.160528000	-0.769773000
6	2.573741000	-2.965344000	-0.749132000
6	1.770374000	-2.658757000	0.467018000
6	2.420038000	-2.498347000	1.739430000
6	3.833730000	-2.369921000	1.876120000
6	4.410025000	-2.176060000	3.124015000
6	3.603924000	-2.127675000	4.293338000
6	2.227289000	-2.254488000	4.191564000
6	1.601343000	-2.422812000	2.920396000
6	0.192307000	-2.535622000	2.798122000
6	-0.464780000	-2.611239000	1.567462000
6	0.377533000	-2.588465000	0.403228000
6	3.534972000	-1.415290000	-4.151346000
1	5.031408000	-3.571709000	-3.743799000
1	5.781580000	-5.680964000	-2.750002000
1	5.626851000	-7.394740000	-0.935000000
1	3.987496000	-7.081128000	0.943111000
1	2.580331000	-5.043978000	1.061333000
1	4.464175000	-2.406648000	0.976598000
1	5.501058000	-2.061041000	3.208482000
1	4.075928000	-1.987594000	5.277367000
1	1.589336000	-2.213587000	5.087152000
1	-0.395310000	-2.571214000	3.725867000
6	-1.943326000	-2.769235000	1.527433000
6	3.490806000	-0.011987000	-4.014830000
6	3.735304000	0.825379000	-5.111044000
6	3.991947000	0.250936000	-6.359954000
6	4.017168000	-1.137118000	-6.539925000
6	3.793792000	-1.957948000	-5.432185000
6	-2.718462000	-2.344232000	2.630396000
6	-4.105413000	-2.496410000	2.663185000
6	-4.743495000	-3.096297000	1.571543000
6	-4.011942000	-3.553888000	0.470691000
6	-2.620765000	-3.387376000	0.453106000

1	3.306885000	0.441543000	-3.033908000
1	3.793367000	-3.046906000	-5.572661000
1	-2.239993000	-1.851333000	3.483791000
1	-2.079839000	-3.737650000	-0.430307000
7	-0.403796000	0.159985000	-1.276319000
15	-1.706122000	0.969515000	-0.847195000
8	-1.297490000	2.029403000	0.350071000
8	-2.635975000	-0.097365000	0.010827000
6	-0.958548000	1.559629000	1.604831000
6	0.428073000	1.550867000	1.956925000
6	0.752078000	1.137338000	3.245504000
6	-0.238740000	0.718507000	4.173102000
6	0.129670000	0.297650000	5.486471000
6	-0.819071000	-0.172605000	6.379831000
6	-2.181330000	-0.240783000	5.984556000
6	-2.573889000	0.180645000	4.721911000
6	-1.627238000	0.688371000	3.780376000
6	-1.985860000	1.130916000	2.453187000
6	-3.390532000	1.098330000	1.947357000
6	-4.466141000	1.729885000	2.666266000
6	-4.253500000	2.581126000	3.791646000
6	-5.323806000	3.132545000	4.479996000
6	-6.658898000	2.864768000	4.075556000
6	-6.897402000	2.087277000	2.955031000
6	-5.817498000	1.525708000	2.209418000
6	-6.049799000	0.837179000	0.991132000
6	-5.012809000	0.355816000	0.192575000
6	-3.689340000	0.456817000	0.734232000
6	1.486515000	1.938598000	0.990892000
1	1.804319000	1.138262000	3.566671000
1	1.192033000	0.348342000	5.769876000
1	-0.521962000	-0.495477000	7.388819000
1	-2.935435000	-0.630558000	6.684652000
1	-3.630539000	0.113078000	4.434278000
1	-3.225434000	2.812517000	4.101611000
1	-5.135396000	3.798231000	5.335238000
1	-7.498802000	3.304164000	4.633539000
1	-7.924581000	1.910992000	2.601939000
1	-7.088952000	0.701837000	0.659733000
6	-5.291768000	-0.138804000	-1.181583000
6	1.336480000	3.031262000	0.106340000
6	2.372394000	3.401738000	-0.757633000
6	3.568436000	2.674147000	-0.735991000
6	3.742475000	1.568368000	0.105865000
6	2.695550000	1.207389000	0.960975000
6	-6.347093000	0.455329000	-1.912217000
6	-6.608666000	0.109557000	-3.240493000
6	-5.808435000	-0.865711000	-3.845514000
6	-4.785290000	-1.513068000	-3.147533000
6	-4.534089000	-1.143536000	-1.822623000
1	0.405319000	3.609048000	0.080080000
1	2.816766000	0.321729000	1.601701000
1	-6.942020000	1.256831000	-1.456703000
1	-3.724573000	-1.657313000	-1.296963000
7	-0.499338000	-1.668774000	-3.309090000
7	-2.572841000	1.749044000	-1.928918000
16	-1.236985000	-3.107685000	-3.661660000

8	-2.319600000	-3.435215000	-2.707152000
8	-1.551773000	-3.081045000	-5.100880000
16	-2.647502000	3.366435000	-2.103161000
8	-1.300039000	3.993989000	-2.060801000
8	-3.538131000	3.651382000	-3.240910000
1	4.205949000	-1.575164000	-7.527701000
1	3.759956000	1.911961000	-4.971489000
1	4.675274000	0.990699000	0.095791000
1	2.239188000	4.257042000	-1.431596000
1	-7.406464000	0.614699000	-3.798344000
1	-4.168178000	-2.291840000	-3.610530000
1	-4.509461000	-4.028933000	-0.383820000
1	-4.681210000	-2.135686000	3.524376000
1	-1.073028000	-0.382714000	-4.238491000
16	-6.560192000	-3.271306000	1.593269000
16	-6.057954000	-1.259046000	-5.605850000
16	4.285815000	1.335072000	-7.797408000
16	4.940569000	3.193198000	-1.822096000
9	2.957170000	2.250197000	-7.449482000
9	3.354795000	0.381287000	-8.781160000
9	4.530029000	2.300869000	-9.090567000
9	5.219509000	2.358897000	-6.926643000
9	5.613031000	0.487694000	-8.248087000
9	5.309259000	1.633696000	-2.216033000
9	3.931967000	3.280900000	-3.138825000
9	4.672057000	4.779942000	-1.521885000
9	6.027720000	3.135225000	-0.599100000
9	6.154079000	3.649638000	-2.814220000
9	-6.159849000	-2.865641000	-5.318282000
9	-7.687738000	-1.124399000	-5.496663000
9	-6.259253000	-1.587270000	-7.201385000
9	-5.962534000	0.341714000	-6.033568000
9	-4.434025000	-1.410576000	-5.864831000
9	-6.495736000	-4.741541000	0.871046000
9	-6.711683000	-2.552300000	0.124107000
9	-6.528482000	-3.991715000	3.068812000
9	-6.762893000	-1.807347000	2.325097000
9	-8.188177000	-3.426099000	1.618188000
6	-2.042525000	-0.404553000	-7.520243000
6	-0.607476000	-0.262702000	-7.744110000
6	-0.092859000	0.746319000	-8.504082000
6	-2.999113000	0.528276000	-7.862790000
1	-0.741920000	1.441434000	-9.054394000
1	-2.758811000	1.357075000	-8.545373000
1	-4.061251000	0.277475000	-7.739504000
1	0.991827000	0.867289000	-8.634787000
6	-1.132431000	3.735883000	-6.227065000
6	0.399115000	3.686767000	-6.015911000
6	0.783284000	3.168248000	-4.630452000
6	0.328212000	1.718979000	-4.450472000
1	0.853460000	3.035854000	-6.792401000
1	0.811847000	4.704209000	-6.175971000
1	1.876525000	3.232757000	-4.471418000
1	0.311324000	3.785430000	-3.839714000
1	1.025369000	1.011241000	-4.954502000
1	0.326848000	1.423441000	-3.379098000
6	-1.757881000	2.394988000	-5.796828000

6	-3.055592000	2.009039000	-6.143069000
1	-3.707669000	2.702920000	-6.693265000
1	-3.557785000	1.228875000	-5.559760000
6	-1.032404000	1.458402000	-5.007099000
8	-1.590524000	0.286434000	-4.823546000
6	-1.734549000	4.896681000	-5.401447000
1	-1.332906000	5.860054000	-5.778352000
1	-2.838169000	4.916596000	-5.485773000
1	-1.503242000	4.813964000	-4.325546000
6	-1.420449000	4.026421000	-7.711934000
1	-0.988622000	3.249764000	-8.369784000
1	-2.506199000	4.105724000	-7.920962000
1	-0.963713000	4.996487000	-7.993662000
6	0.055201000	-4.379982000	-3.422321000
6	1.110499000	-4.450547000	-4.351217000
6	0.007190000	-5.347754000	-2.398536000
6	2.095661000	-5.443011000	-4.262858000
6	0.997214000	-6.342235000	-2.298371000
6	2.022788000	-6.412906000	-3.251676000
9	-0.940723000	-5.367890000	-1.463661000
9	0.951766000	-7.231365000	-1.305240000
9	2.944707000	-7.368839000	-3.177120000
9	3.115547000	-5.463813000	-5.130773000
9	1.229890000	-3.546317000	-5.324758000
6	-3.540634000	3.978662000	-0.622261000
6	-2.903883000	4.627351000	0.453673000
6	-4.941556000	3.842780000	-0.575819000
6	-3.646975000	5.108757000	1.546178000
6	-5.692432000	4.331469000	0.503509000
6	-5.044482000	4.989568000	1.558530000
9	-3.024989000	5.693309000	2.574804000
9	-5.746358000	5.478433000	2.579830000
9	-7.019389000	4.168408000	0.523775000
9	-5.613827000	3.234321000	-1.553121000
9	-1.583769000	4.814150000	0.509120000
6	0.305809000	-1.265756000	-7.091036000
1	0.260726000	-1.193597000	-5.986542000
1	-0.010579000	-2.301742000	-7.322834000
1	1.354214000	-1.126901000	-7.410937000
1	-2.363972000	-1.301977000	-6.967672000

TS-C2: Imaginary frequency = - 163.1

15	0.180041000	-1.268942000	-1.657966000
8	1.809268000	-0.986360000	-1.777275000
8	0.113976000	-2.713779000	-0.851914000
6	2.688440000	-2.027283000	-2.052929000
6	3.428229000	-1.995764000	-3.279654000
6	4.307294000	-3.047713000	-3.531020000
6	4.440385000	-4.156984000	-2.656870000
6	5.281415000	-5.262300000	-2.981163000
6	5.350786000	-6.366882000	-2.148472000
6	4.565521000	-6.412190000	-0.966366000
6	3.757983000	-5.341123000	-0.611197000
6	3.686940000	-4.175670000	-1.430905000
6	2.862158000	-3.040664000	-1.103129000
6	2.211641000	-2.968674000	0.232691000
6	2.994249000	-3.018764000	1.438331000

6	4.419069000	-2.955019000	1.439132000
6	5.130363000	-2.985494000	2.630528000
6	4.452973000	-3.093854000	3.874724000
6	3.068251000	-3.142716000	3.907250000
6	2.305144000	-3.087985000	2.702133000
6	0.884815000	-3.068012000	2.726160000
6	0.111898000	-2.926081000	1.572591000
6	0.824740000	-2.862587000	0.331503000
6	3.383612000	-0.875987000	-4.252406000
1	4.923909000	-3.020402000	-4.441114000
1	5.855668000	-5.228441000	-3.919305000
1	5.990551000	-7.221256000	-2.414181000
1	4.591405000	-7.307937000	-0.328173000
1	3.149549000	-5.393883000	0.301726000
1	4.949819000	-2.847823000	0.483103000
1	6.228184000	-2.916043000	2.610559000
1	5.029856000	-3.125451000	4.810989000
1	2.531845000	-3.206997000	4.866718000
1	0.381205000	-3.163354000	3.699395000
6	-1.368011000	-2.851407000	1.663466000
6	3.477695000	0.465646000	-3.829745000
6	3.694947000	1.498245000	-4.751093000
6	3.767266000	1.185947000	-6.114469000
6	3.602581000	-0.127678000	-6.573961000
6	3.418655000	-1.150541000	-5.638448000
6	-1.961925000	-2.267293000	2.804143000
6	-3.349460000	-2.201137000	2.948045000
6	-4.160630000	-2.699082000	1.924875000
6	-3.612609000	-3.295748000	0.784418000
6	-2.219847000	-3.376934000	0.665496000
1	3.446846000	0.704842000	-2.762516000
1	3.304303000	-2.185935000	-5.987634000
1	-1.335133000	-1.820347000	3.588041000
1	-1.810667000	-3.848501000	-0.232709000
7	-0.320632000	-0.071649000	-0.770869000
15	-1.541635000	0.920244000	-0.552320000
8	-1.172209000	1.935546000	0.694654000
8	-2.764208000	0.037838000	0.130924000
6	-1.172155000	1.491503000	2.003598000
6	0.094828000	1.293523000	2.633552000
6	0.089791000	0.919210000	3.973920000
6	-1.118356000	0.704634000	4.691658000
6	-1.092485000	0.297679000	6.059743000
6	-2.264255000	0.017444000	6.743586000
6	-3.511503000	0.123543000	6.073582000
6	-3.571971000	0.528705000	4.747486000
6	-2.387393000	0.853183000	4.019344000
6	-2.398109000	1.275496000	2.638789000
6	-3.662129000	1.436770000	1.867359000
6	-4.727509000	2.270422000	2.358115000
6	-4.579995000	3.137664000	3.481679000
6	-5.648272000	3.891277000	3.944199000
6	-6.914971000	3.818169000	3.305359000
6	-7.075894000	3.026066000	2.181434000
6	-5.989284000	2.257189000	1.666283000
6	-6.110784000	1.533594000	0.453503000
6	-5.043322000	0.846280000	-0.125463000

6	-3.832056000	0.781759000	0.637300000
6	1.363797000	1.419954000	1.876081000
1	1.047811000	0.786502000	4.499375000
1	-0.115432000	0.201703000	6.558463000
1	-2.230739000	-0.298101000	7.797038000
1	-4.441697000	-0.126541000	6.605452000
1	-4.543738000	0.577470000	4.240686000
1	-3.600696000	3.217676000	3.972394000
1	-5.507589000	4.564716000	4.802532000
1	-7.755319000	4.417599000	3.685297000
1	-8.038274000	2.996718000	1.648555000
1	-7.084980000	1.538988000	-0.055769000
6	-5.204460000	0.252692000	-1.478243000
6	1.612014000	2.478436000	0.973777000
6	2.827321000	2.558362000	0.283706000
6	3.792992000	1.564971000	0.492441000
6	3.572901000	0.494266000	1.365849000
6	2.357814000	0.434420000	2.053683000
6	-6.071372000	0.892040000	-2.396791000
6	-6.316353000	0.365396000	-3.667270000
6	-5.667392000	-0.819146000	-4.034182000
6	-4.776604000	-1.467656000	-3.172874000
6	-4.554173000	-0.929258000	-1.899750000
1	0.851812000	3.247461000	0.788623000
1	2.166190000	-0.421483000	2.715573000
1	-6.539903000	1.847630000	-2.127075000
1	-3.864347000	-1.457445000	-1.233095000
7	-0.469314000	-1.572830000	-3.119857000
7	-2.102798000	1.796620000	-1.765191000
16	-1.244559000	-2.991192000	-3.473614000
8	-2.270732000	-3.349834000	-2.468633000
8	-1.654550000	-2.910400000	-4.889019000
16	-1.784398000	3.386260000	-1.912642000
8	-0.378190000	3.730029000	-1.591806000
8	-2.336976000	3.824911000	-3.210041000
1	3.647019000	-0.358004000	-7.645666000
1	3.838158000	2.525069000	-4.392511000
1	4.323791000	-0.291731000	1.507446000
1	3.002540000	3.386191000	-0.413846000
1	-6.990777000	0.880586000	-4.362252000
1	-4.247277000	-2.381765000	-3.468113000
1	-4.250273000	-3.697684000	-0.012889000
1	-3.785158000	-1.744986000	3.843238000
1	-1.095386000	-0.398144000	-4.212918000
16	-5.971135000	-2.527720000	2.083230000
16	-5.975429000	-1.524191000	-5.686254000
16	4.140533000	2.501237000	-7.326698000
16	5.380109000	1.643820000	-0.406642000
9	3.215784000	3.604110000	-6.529489000
9	2.839559000	2.084491000	-8.261053000
9	4.464165000	3.670670000	-8.418947000
9	5.450519000	2.995121000	-6.479601000
9	5.087950000	1.500880000	-8.217570000
9	5.137581000	0.142978000	-1.065472000
9	4.676582000	2.339564000	-1.733455000
9	5.719743000	3.133584000	0.179201000
9	6.211726000	0.959366000	0.831240000

9	6.790392000	1.699781000	-1.230164000
9	-6.090820000	-3.048734000	-5.107309000
9	-7.597552000	-1.338739000	-5.553985000
9	-6.227666000	-2.138630000	-7.186577000
9	-5.881292000	-0.032804000	-6.414627000
9	-4.361226000	-1.746229000	-5.952255000
9	-6.225784000	-4.077716000	1.615931000
9	-6.128131000	-2.030688000	0.523791000
9	-5.943766000	-3.001199000	3.656680000
9	-5.844356000	-0.951641000	2.566249000
9	-7.591699000	-2.365498000	2.227984000
6	-2.165443000	-1.018467000	-7.923523000
6	-0.712779000	-1.043259000	-7.961627000
6	0.013457000	0.101236000	-8.117147000
6	-2.982677000	-0.000588000	-8.363062000
1	-0.468074000	1.067142000	-8.301167000
1	-2.597675000	0.784831000	-9.029565000
1	-4.071524000	-0.152338000	-8.340936000
1	1.112456000	0.096351000	-8.075813000
6	-1.387107000	3.328676000	-6.852311000
6	0.105739000	3.516432000	-6.497480000
6	0.413926000	3.216125000	-5.031315000
6	0.113139000	1.754237000	-4.693328000
1	0.724956000	2.863007000	-7.146404000
1	0.401745000	4.556513000	-6.744673000
1	1.476206000	3.428594000	-4.810268000
1	-0.184537000	3.866007000	-4.365479000
1	0.928589000	1.080952000	-5.042054000
1	0.048258000	1.599983000	-3.594274000
6	-1.892774000	1.981300000	-6.295633000
6	-3.135103000	1.456054000	-6.644720000
1	-3.829157000	2.053958000	-7.252924000
1	-3.579441000	0.664181000	-6.032969000
6	-1.156004000	1.252470000	-5.304355000
8	-1.643234000	0.105508000	-4.918042000
6	-2.233946000	4.466790000	-6.234457000
1	-1.873483000	5.447126000	-6.609003000
1	-3.298678000	4.362727000	-6.528435000
1	-2.194259000	4.461537000	-5.132195000
6	-1.552492000	3.427739000	-8.383890000
1	-0.932233000	2.699728000	-8.942003000
1	-2.607884000	3.301768000	-8.699073000
1	-1.234865000	4.436574000	-8.715392000
6	0.049269000	-4.280953000	-3.396221000
6	1.090733000	-4.230900000	-4.339064000
6	0.003488000	-5.378992000	-2.516667000
6	2.038405000	-5.256285000	-4.447084000
6	0.971712000	-6.397269000	-2.590246000
6	1.966820000	-6.356535000	-3.578592000
9	-0.922448000	-5.504111000	-1.566922000
9	0.936356000	-7.417098000	-1.731053000
9	2.855054000	-7.342307000	-3.670060000
9	3.012553000	-5.181453000	-5.360685000
9	1.214014000	-3.182142000	-5.157586000
6	-2.855310000	4.185681000	-0.665634000
6	-2.364150000	4.813970000	0.493361000
6	-4.242237000	4.218867000	-0.908420000

6	-3.240154000	5.467074000	1.379111000
6	-5.119906000	4.883369000	-0.040461000
6	-4.612702000	5.531444000	1.095962000
9	-2.768338000	6.037140000	2.491903000
9	-5.431934000	6.182915000	1.920203000
9	-6.433188000	4.892990000	-0.292347000
9	-4.774433000	3.607054000	-1.967949000
9	-1.070358000	4.815508000	0.819369000
6	-0.063202000	-2.385734000	-7.759055000
1	-0.444280000	-2.855750000	-6.832419000
1	-0.313590000	-3.062948000	-8.602955000
1	1.037960000	-2.304256000	-7.696725000
1	-2.645467000	-1.887618000	-7.445157000

TS-C3: Imaginary frequency = - 116.29

15	0.473884343	-1.365211397	-1.734442433
8	1.822589643	-0.805237134	-0.952801559
8	0.450689091	-2.926241978	-1.208304140
6	2.991383516	-1.539746538	-1.141848603
6	3.975142945	-1.029935422	-2.051524384
6	5.166366874	-1.749106197	-2.146921913
6	5.358322581	-2.997092681	-1.501791997
6	6.552562564	-3.753348822	-1.699447853
6	6.687310904	-5.022309519	-1.162406708
6	5.616514868	-5.597248051	-0.427162083
6	4.451846573	-4.879741691	-0.198767570
6	4.302623871	-3.549164828	-0.691823535
6	3.131417201	-2.750564617	-0.444404798
6	2.077149177	-3.189418098	0.517638930
6	2.375832002	-3.515415602	1.891557580
6	3.691998880	-3.457404615	2.440936167
6	3.925050260	-3.731221184	3.781267685
6	2.854746740	-4.081909809	4.646195725
6	1.563018556	-4.143758807	4.148282837
6	1.287349256	-3.857584991	2.776925064
6	-0.047967936	-3.866316171	2.290446732
6	-0.356031000	-3.546995347	0.971472478
6	0.739768601	-3.223319549	0.110091107
6	3.725132038	0.129351588	-2.948882370
1	5.983665919	-1.355632733	-2.767972191
1	7.355211465	-3.314728020	-2.311328812
1	7.607094393	-5.600851613	-1.333061221
1	5.703747946	-6.627058264	-0.050348760
1	3.622879979	-5.341684386	0.354537288
1	4.529079314	-3.171119569	1.792150853
1	4.950056925	-3.669889590	4.176872831
1	3.052910458	-4.299520599	5.706273183
1	0.720438689	-4.409252213	4.805954577
1	-0.857899059	-4.135182220	2.985114518
6	-1.763056974	-3.489924376	0.500607712
6	2.898300915	1.227700080	-2.617949387
6	2.585516026	2.215862872	-3.560273699
6	3.130874590	2.115619315	-4.845047593
6	4.002138475	1.079476030	-5.196752134
6	4.290603255	0.099686466	-4.245022050
6	-2.758485248	-2.985447254	1.367355281
6	-4.098290354	-2.912602123	0.974028356

6	-4.442373680	-3.352273527	-0.309846485
6	-3.484167349	-3.849295854	-1.201013920
6	-2.148056223	-3.917191544	-0.791190177
1	2.466649543	1.310882405	-1.615149663
1	4.915604199	-0.750162541	-4.547050317
1	-2.481705094	-2.610294556	2.362139000
1	-1.406809621	-4.299530889	-1.502479977
7	-0.763368484	-0.592083252	-1.091432316
15	-1.272347269	0.899687460	-0.974918403
8	-0.768436024	1.630339199	0.434686935
8	-2.907682991	0.722663516	-0.749743268
6	-1.027590195	0.976032659	1.636602362
6	0.053059661	0.397544645	2.385843090
6	-0.307278403	-0.348857377	3.509146902
6	-1.656669310	-0.561098144	3.893640520
6	-1.993638958	-1.429945540	4.974732295
6	-3.318228010	-1.657793894	5.315025995
6	-4.358340620	-1.013118857	4.592232728
6	-4.062717011	-0.144831928	3.550075715
6	-2.711256516	0.102423726	3.170564909
6	-2.355693467	0.936940839	2.055726240
6	-3.379724446	1.769063257	1.367961031
6	-4.077912593	2.785040703	2.114494756
6	-3.713835239	3.150038559	3.444891850
6	-4.393952777	4.155039501	4.117732039
6	-5.477168378	4.834708239	3.501521867
6	-5.849786057	4.508870153	2.207312635
6	-5.158718092	3.495179103	1.481951026
6	-5.509612886	3.164049925	0.146746006
6	-4.792214606	2.242205382	-0.608902892
6	-3.675283663	1.596138000	0.012319458
6	1.484666503	0.587076912	2.041067339
1	0.477744569	-0.802038648	4.130702536
1	-1.178182859	-1.928611058	5.521701837
1	-3.568221749	-2.340244215	6.140970379
1	-5.408151750	-1.207249529	4.858511664
1	-4.872370570	0.342137013	2.988739861
1	-2.872360350	2.637772182	3.931383781
1	-4.086042923	4.431131373	5.137408364
1	-6.010122589	5.626446289	4.048716840
1	-6.678552868	5.037344396	1.711482611
1	-6.379412399	3.658714629	-0.311188012
6	-5.293133949	1.917383319	-1.969749823
6	1.939177162	1.695886919	1.294729302
6	3.287334128	1.864570543	0.963472957
6	4.208950461	0.907695614	1.399846584
6	3.809281578	-0.174275410	2.190686794
6	2.457021326	-0.328083523	2.501251056
6	-5.551058443	2.945234640	-2.900962289
6	-6.205685536	2.674718767	-4.107889492
6	-6.622182912	1.361969981	-4.373861532
6	-6.362819961	0.317057480	-3.479128015
6	-5.678697268	0.601376289	-2.289277293
1	1.222755051	2.441904400	0.951366807
1	2.163176425	-1.203515205	3.092732982
1	-5.215187355	3.967401742	-2.681348989
1	-5.496076995	-0.210925632	-1.576275371

7	0.696438468	-1.380552480	-3.313652023
7	-0.801279793	1.890273050	-2.163735372
16	0.409748022	-2.656317238	-4.284937954
8	-0.847613441	-3.369714363	-3.936410327
8	0.606412669	-2.214104568	-5.677362677
16	-1.245537047	3.461333296	-2.422810404
8	-0.232792269	4.037927500	-3.326333684
8	-2.677643690	3.548811834	-2.782151851
1	4.422122118	1.011285122	-6.207639910
1	1.900104844	3.032387060	-3.297165686
1	4.539177761	-0.906909542	2.551370531
1	3.599733588	2.733514776	0.371598866
1	-6.399350937	3.481849020	-4.824481237
1	-6.699347804	-0.707123938	-3.677908578
1	-3.761427438	-4.181710955	-2.209082334
1	-4.851820902	-2.504579675	1.658913877
1	-0.633624597	1.263637191	-3.848572511
16	-6.189084081	-3.277093622	-0.829017625
16	-7.558887055	1.020521343	-5.909688923
16	2.652817429	3.327881934	-6.118562961
16	5.959480446	1.037147416	0.908451816
9	1.075110635	3.277274636	-5.669798624
9	2.357694230	2.140052991	-7.236218374
9	2.208186279	4.411372613	-7.280430110
9	2.903776302	4.599337189	-5.120009719
9	4.185989832	3.461101711	-6.685476940
9	6.053734826	-0.580266075	0.598175990
9	5.561407479	1.314093651	-0.662349244
9	5.988435999	2.652923141	1.184857700
9	6.474816510	0.762724383	2.443649633
9	7.528646172	1.143482795	0.464456733
9	-8.856545558	0.482632071	-5.068907489
9	-8.201429559	2.530602153	-5.940717396
9	-8.386392559	0.724165800	-7.287847281
9	-6.303627773	1.541543417	-6.851914497
9	-6.995085732	-0.521208417	-6.012669868
9	-6.127547589	-4.758935865	-1.520676492
9	-5.787612369	-2.589577719	-2.285563199
9	-6.713827630	-3.940164977	0.573396804
9	-6.381086752	-1.767784352	-0.175747616
9	-7.749470719	-3.183468877	-1.305300808
6	-1.208099542	3.164955043	-7.710162050
6	-2.215239552	3.475715260	-6.705710695
6	-3.276471771	2.646847039	-6.477276483
6	-1.277027562	2.183819613	-8.666609281
1	-3.484343988	1.772825386	-7.105183832
1	-2.223149155	1.685236877	-8.915001304
1	-0.450998189	2.067864441	-9.383680101
1	-3.983841491	2.849226774	-5.660088230
6	-2.802984466	-1.075469747	-7.057833219
6	-3.949662058	-1.123145529	-6.024057672
6	-3.449384551	-1.352962110	-4.604447194
6	-2.561376106	-0.194464688	-4.158920462
1	-4.517946125	-0.172099416	-6.069226689
1	-4.663736095	-1.916815907	-6.323624760
1	-4.289011770	-1.459303941	-3.893328291
1	-2.862913976	-2.290800317	-4.540383505

1	-3.161598407	0.684050402	-3.827298683
1	-1.953921201	-0.486923994	-3.279593239
6	-1.694856483	-0.107251201	-6.584041368
6	-0.651662580	0.274404777	-7.417206665
1	-0.541073580	-0.197757972	-8.403381864
1	0.231453591	0.779797571	-7.007027097
6	-1.615082291	0.294965953	-5.209248753
8	-0.631555210	1.073039432	-4.844441193
6	-2.188912791	-2.485259392	-7.228973383
1	-2.969647747	-3.190114541	-7.582437697
1	-1.369182094	-2.473375707	-7.974665760
1	-1.755310295	-2.874180930	-6.291065766
6	-3.404927980	-0.653919177	-8.413438806
1	-3.938579002	0.315936801	-8.356067771
1	-2.644110577	-0.597409472	-9.217370520
1	-4.153384554	-1.408825998	-8.727026342
1	-0.270127242	3.738635190	-7.637697767
6	-1.991866238	4.726578994	-5.896553262
1	-2.721977329	4.817116688	-5.072114437
1	-0.979495556	4.732421054	-5.449000971
1	-2.075265489	5.620630586	-6.550588313
6	1.777796205	-3.821087485	-3.931546315
6	3.052496404	-3.538164083	-4.458626846
6	1.616454226	-5.001997934	-3.181148635
6	4.142273943	-4.393529863	-4.240500697
6	2.704533485	-5.864151238	-2.956336078
6	3.960420477	-5.577735583	-3.511469710
9	0.452576849	-5.362532560	-2.637850893
9	2.544891578	-6.967047402	-2.218517534
9	4.978871686	-6.415327608	-3.323498639
9	5.348563498	-4.079456896	-4.725707385
9	3.277293035	-2.436096786	-5.174677182
6	-1.021817464	4.318768645	-0.833627701
6	0.274475458	4.399114547	-0.294250193
6	-2.079696653	4.923030414	-0.130664787
6	0.507416278	4.979470994	0.959001670
6	-1.860850533	5.500085272	1.133962664
6	-0.568153564	5.529630461	1.679060411
9	1.738043427	4.999532423	1.476387047
9	-0.358886069	6.078833337	2.872043223
9	-2.876493117	6.044916517	1.803654653
9	-3.320950023	4.972476415	-0.607009731
9	1.319567391	3.854652251	-0.929303654

TS-C4: Imaginary frequency =   - 104.48

15	0.212086801	-1.465075043	-1.669519800
8	1.843110829	-1.180461856	-1.592495807
8	0.062089022	-2.845687546	-0.758856109
6	2.742544484	-2.210377223	-1.817745095
6	3.570868967	-2.162185172	-2.988737024
6	4.497714704	-3.193064317	-3.147541083
6	4.576473885	-4.298721045	-2.262890541
6	5.471827843	-5.382223243	-2.507520283
6	5.491299288	-6.486990058	-1.672703357
6	4.598146579	-6.557773795	-0.571000879
6	3.733247576	-5.509146731	-0.292624669
6	3.713417490	-4.342268963	-1.112086793

6	2.848930230	-3.219650108	-0.854466975
6	2.106237731	-3.135058512	0.432914226
6	2.812819296	-3.184318247	1.687308066
6	4.236238656	-3.141051963	1.778956023
6	4.872143423	-3.159358995	3.012567452
6	4.119070798	-3.235640895	4.214250203
6	2.734746201	-3.265525184	4.160252575
6	2.048843935	-3.221898267	2.909288274
6	0.630121445	-3.189075222	2.847524806
6	-0.068705871	-3.053207223	1.647629179
6	0.715918582	-2.996914094	0.448888164
6	3.531878448	-1.064812305	-3.987663424
1	5.199250590	-3.159460826	-3.992869945
1	6.129600361	-5.331889207	-3.388398989
1	6.174297968	-7.324245505	-1.878481836
1	4.584109683	-7.456708403	0.063012366
1	3.039739569	-5.580595460	0.556241990
1	4.830971729	-3.073062698	0.857730986
1	5.970250063	-3.108904797	3.058210177
1	4.636444652	-3.258864043	5.184896590
1	2.137853434	-3.307202972	5.084642023
1	0.068863958	-3.274562643	3.789507779
6	-1.552925071	-2.991074442	1.648275992
6	3.220105604	0.269585944	-3.644210128
6	3.332677743	1.304263966	-4.579460884
6	3.749998221	1.002833149	-5.880525077
6	4.029958870	-0.312856792	-6.272209646
6	3.907356299	-1.333575837	-5.325757502
6	-2.216035183	-2.424331836	2.759649056
6	-3.609567560	-2.389795909	2.837657675
6	-4.359093837	-2.909226502	1.778799365
6	-3.742444524	-3.485713074	0.662946205
6	-2.343211865	-3.527360309	0.604432465
1	2.922847759	0.517140739	-2.620886835
1	4.081024019	-2.371306078	-5.642599475
1	-1.638440895	-1.972040952	3.576995863
1	-1.881263923	-3.980459694	-0.279202275
7	-0.342284541	-0.176714394	-0.939334913
15	-1.548691197	0.819006938	-0.784403315
8	-1.244898345	1.865737898	0.456781351
8	-2.808103313	-0.043811806	-0.146197360
6	-1.262775995	1.407360553	1.768605803
6	-0.004409166	1.187003891	2.410169871
6	-0.023364242	0.799503364	3.745679636
6	-1.239393366	0.596388787	4.451926544
6	-1.227530812	0.177187717	5.816329669
6	-2.407437023	-0.101537591	6.486676837
6	-3.646997599	0.017900600	5.805019206
6	-3.692374323	0.433518147	4.481596512
6	-2.499815343	0.758280291	3.767352795
6	-2.496578117	1.187726613	2.386832165
6	-3.759683147	1.303603517	1.607619795
6	-4.863348832	2.095474658	2.084808155
6	-4.736982205	3.025883985	3.156804725
6	-5.824363558	3.775149405	3.581530427
6	-7.094583471	3.614962066	2.967866392
6	-7.243163093	2.734852461	1.907662923

6	-6.134991011	1.980146452	1.421363027
6	-6.246647286	1.168710210	0.263394356
6	-5.150832634	0.533870630	-0.314854639
6	-3.911425790	0.614904826	0.395356341
6	1.281252282	1.295333104	1.677486577
1	0.930001506	0.641375457	4.272491965
1	-0.255340061	0.069625893	6.322055054
1	-2.386290131	-0.426599986	7.537557963
1	-4.583925714	-0.231291917	6.325452794
1	-4.658598758	0.488882527	3.965633266
1	-3.756816562	3.162896224	3.634309119
1	-5.698213927	4.504181800	4.395868580
1	-7.953318229	4.204272814	3.322571362
1	-8.215495539	2.623700340	1.403503498
1	-7.232365344	1.077863514	-0.215100968
6	-5.284272395	-0.109855813	-1.646182829
6	1.613048832	2.413415111	0.884915958
6	2.842961348	2.491717398	0.222891714
6	3.746876847	1.429155762	0.354231839
6	3.447828898	0.300994810	1.125011142
6	2.215948636	0.245026502	1.783667427
6	-6.176044983	0.463858982	-2.583439019
6	-6.381938746	-0.103416488	-3.842657032
6	-5.663480369	-1.256886749	-4.181552246
6	-4.742020936	-1.835754741	-3.303815122
6	-4.563068321	-1.258880827	-2.039737712
1	0.911379646	3.248335064	0.792266689
1	1.965206098	-0.649309920	2.370459645
1	-6.691066431	1.401690915	-2.336172227
1	-3.845757066	-1.731262196	-1.360282325
7	-0.273376146	-1.831402622	-3.148044798
7	-1.851702003	1.743290233	-2.081613064
16	-1.047691543	-3.207103626	-3.560147432
8	-2.145525979	-3.551809486	-2.622736417
8	-1.358461002	-3.153607148	-5.000874714
16	-2.854972218	3.067176744	-2.157582620
8	-2.540311535	3.755140659	-3.430392375
8	-4.260982582	2.749226777	-1.861402904
1	4.321847656	-0.544128668	-7.303892457
1	3.126795434	2.338080420	-4.276099765
1	4.155590755	-0.532215227	1.213639359
1	3.085108424	3.376564965	-0.378657756
1	-7.078296686	0.357639054	-4.553846980
1	-4.149515326	-2.718821781	-3.575245509
1	-4.333308385	-3.904307880	-0.161475792
1	-4.098133781	-1.947073612	3.711679473
1	-1.624820383	1.110590614	-3.702619699
16	-6.178426796	-2.799197592	1.867603874
16	-5.921259854	-2.005711827	-5.821857331
16	3.921174800	2.346403409	-7.102184376
16	5.358216261	1.508928592	-0.499449271
9	2.418126361	2.948454292	-6.737151965
9	3.245664788	1.431580554	-8.300529151
9	4.052958524	3.550022382	-8.198261216
9	4.585251263	3.360554330	-6.001238621
9	5.408679425	1.839727308	-7.556615092
9	5.139572729	0.015844924	-1.170076195

9	4.690661181	2.210408773	-1.835084913
9	5.679806160	2.995764416	0.109809565
9	6.143243691	0.817696612	0.765275854
9	6.794622515	1.572939887	-1.273454680
9	-6.007755558	-3.519291624	-5.211732469
9	-7.551337026	-1.860314863	-5.717723131
9	-6.138931626	-2.652561745	-7.313039727
9	-5.855253186	-0.523070902	-6.575000666
9	-4.302621344	-2.191816311	-6.061640790
9	-6.363553654	-4.356357349	1.391876009
9	-6.294857669	-2.307976628	0.303839505
9	-6.194173725	-3.274957740	3.440881235
9	-6.119017244	-1.221652082	2.356454559
9	-7.807855034	-2.690007451	1.953176411
6	-1.893290731	-0.725997043	-7.354446356
6	-0.537564770	-0.196975899	-7.315065512
6	-0.171901181	0.891169386	-8.054179788
6	-2.921975240	-0.216508920	-8.105951991
1	-0.854549779	1.355676839	-8.777826567
1	-2.742687082	0.503514263	-8.918523277
1	-3.915255738	-0.682858710	-8.064506346
1	0.845427615	1.302216253	-7.994814487
6	-2.167602458	3.777025148	-7.209778868
6	-0.704649581	4.247821455	-7.027348763
6	-0.226115824	4.208630562	-5.574754417
6	-0.320466736	2.793834518	-5.004460919
1	-0.036821509	3.611863598	-7.645253719
1	-0.615764038	5.278317714	-7.430380718
1	0.823334410	4.557088404	-5.512801067
1	-0.833848116	4.886199300	-4.942864558
1	0.499443268	2.159356059	-5.409387523
1	-0.205645965	2.775679461	-3.902920313
6	-2.405216048	2.439313843	-6.485609879
6	-3.514878012	1.636908919	-6.719832704
1	-4.262329404	1.914648126	-7.476129789
1	-3.820931727	0.889084791	-5.980122251
6	-1.581855399	2.079353515	-5.371158309
8	-1.930240130	1.026576344	-4.673276070
6	-3.136774102	4.800978391	-6.563784050
1	-2.975827670	5.810586060	-6.995898324
1	-4.188941091	4.508328394	-6.757011207
1	-3.011303195	4.850698779	-5.465910456
6	-2.484133271	3.699032737	-8.713323838
1	-1.810467678	2.999352960	-9.245428078
1	-3.530116851	3.387647502	-8.906077240
1	-2.352835087	4.698925013	-9.173851474
1	-2.109931633	-1.545689949	-6.648755343
6	0.428245202	-0.847687044	-6.368752128
1	1.425741104	-0.378572259	-6.419771539
1	0.059180676	-0.804828918	-5.323699241
1	0.528408850	-1.926637726	-6.592544000
6	0.231661689	-4.510572993	-3.381672315
6	1.336254789	-4.491497222	-4.254052719
6	0.142506706	-5.561402222	-2.449873736
6	2.311740074	-5.497289483	-4.225953751
6	1.127353825	-6.564947423	-2.399312200
6	2.192107526	-6.555063562	-3.311759905

9	-0.845509954	-5.654275964	-1.558597591
9	1.046304651	-7.539162049	-1.489501681
9	3.103567957	-7.525283653	-3.283968399
9	3.359728758	-5.443161422	-5.056879754
9	1.513148749	-3.493339488	-5.125057404
6	-2.260201010	4.177610586	-0.845695221
6	-0.907366345	4.561056147	-0.838747843
6	-3.109133427	4.665876503	0.165767181
6	-0.386188449	5.355656927	0.189449607
6	-2.589579976	5.455854470	1.208712832
6	-1.230886656	5.806697550	1.218312958
9	0.916527509	5.661229623	0.213549954
9	-0.739447341	6.554244370	2.203163915
9	-3.391989133	5.889789236	2.182688930
9	-4.408498313	4.396153665	0.204724303
9	-0.053045557	4.107818144	-1.763089499

TS-C5: Imaginary frequency = - 107.58

15	0.226434834	-1.469941578	-1.637405986
8	1.857542647	-1.186883811	-1.556704152
8	0.072772155	-2.849091277	-0.725116069
6	2.756420671	-2.218053291	-1.778394385
6	3.587501950	-2.172399086	-2.947534113
6	4.513572477	-3.204518073	-3.102754430
6	4.589100702	-4.308992402	-2.216345221
6	5.483868673	-5.393788928	-2.457353400
6	5.500273513	-6.497364078	-1.620897471
6	4.604525304	-6.565618142	-0.521143728
6	3.740078433	-5.515691474	-0.246281865
6	3.723350771	-4.349977224	-1.067468792
6	2.859441816	-3.226092838	-0.813459122
6	2.113724271	-3.138920806	0.472011092
6	2.817194371	-3.187148651	1.728193655
6	4.240428697	-3.145212542	1.823248016
6	4.873305283	-3.162462715	3.058430717
6	4.117225534	-3.236307863	4.258376189
6	2.733005985	-3.264838478	4.201047569
6	2.050196271	-3.222246059	2.948357839
6	0.631658189	-3.188072412	2.883087932
6	-0.064105196	-3.053187196	1.681300980
6	0.723508358	-2.999330963	0.484407280
6	3.552050120	-1.076353120	-3.948058567
1	5.217087048	-3.172877755	-3.946512770
1	6.143721579	-5.345416430	-3.336785337
1	6.182869047	-7.335626359	-1.823895291
1	4.588097859	-7.463613670	0.114141680
1	3.044544312	-5.585181808	0.601088846
1	4.837471020	-3.079105904	0.903380817
1	5.971348563	-3.113071684	3.106683909
1	4.632208328	-3.258718389	5.230312390
1	2.133823678	-3.304618262	5.124038290
1	0.068026560	-3.271674567	3.823822530
6	-1.548263588	-2.989606493	1.678157243
6	3.240900528	0.258861964	-3.607199306
6	3.356834162	1.292121587	-4.543614299
6	3.776933380	0.988431163	-5.843259803
6	4.056401726	-0.328099640	-6.232448086

6	3.930413938	-1.347373817	-5.284884609
6	-2.213663746	-2.420896850	2.787156965
6	-3.607360913	-2.384828843	2.861511540
6	-4.354692198	-2.904685000	1.801311051
6	-3.735756573	-3.483203279	0.687779757
6	-2.336421773	-3.526396690	0.632956657
1	2.941451782	0.508157010	-2.584938468
1	4.103689097	-2.385731787	-5.599879999
1	-1.637736313	-1.968223212	3.605468194
1	-1.872692423	-3.981087424	-0.248925598
7	-0.328447237	-0.179969617	-0.910531302
15	-1.534070542	0.817400416	-0.760143463
8	-1.232389943	1.865406134	0.480477212
8	-2.796156945	-0.043160297	-0.124133286
6	-1.254163167	1.408844884	1.792848789
6	0.002323261	1.188123966	2.437963572
6	-0.020431941	0.802454973	3.773935590
6	-1.238478361	0.601482390	4.477307907
6	-1.230523252	0.184043401	5.842279938
6	-2.412414383	-0.092630773	6.509973124
6	-3.650102749	0.027177695	5.824990457
6	-3.691670517	0.441091488	4.500908836
6	-2.496973332	0.763717770	3.789279302
6	-2.489781020	1.191310855	2.408192593
6	-3.750788520	1.307423159	1.625602091
6	-4.854921473	2.100905337	2.099044282
6	-4.730385596	3.032515945	3.170208491
6	-5.818156933	3.783236921	3.591354992
6	-7.087001488	3.623407868	2.974764435
6	-7.233725692	2.742151389	1.915256017
6	-6.125025258	1.985885084	1.432618256
6	-6.234557133	1.173103760	0.275398171
6	-5.137915895	0.536514817	-0.299337371
6	-3.900159667	0.617328989	0.413820794
6	1.289955129	1.294133470	1.708405474
1	0.931422655	0.644073965	4.303399019
1	-0.259730980	0.076155600	6.350616154
1	-2.394271979	-0.416381522	7.561314417
1	-4.588614283	-0.220399977	6.343338191
1	-4.656512662	0.496693905	3.982403688
1	-3.751279241	3.169235735	3.649963558
1	-5.693373534	4.513143441	4.405120622
1	-7.946094103	4.213890547	3.326642443
1	-8.204907323	2.631230043	1.408833725
1	-7.219184699	1.082521788	-0.205386867
6	-5.268829241	-0.108905039	-1.630115190
6	1.625023839	2.410885866	0.915347716
6	2.856667786	2.486958588	0.256286133
6	3.759017935	1.423506481	0.391147380
6	3.456755647	0.296627945	1.162545394
6	2.223166539	0.242878288	1.818183718
6	-6.157883598	0.464384038	-2.570218195
6	-6.361497287	-0.104443414	-3.829106291
6	-5.643447242	-1.259116372	-4.164753467
6	-4.724535334	-1.837654596	-3.284121767
6	-4.547868625	-1.259189319	-2.020437606
1	0.924580615	3.246526210	0.819999981

1	1.969922152	-0.650455399	2.405428655
1	-6.672523974	1.403075140	-2.325441383
1	-3.832560667	-1.731348748	-1.338723459
7	-0.255583028	-1.838007337	-3.116634803
7	-1.832420234	1.740277238	-2.059380421
16	-1.030737634	-3.213311015	-3.528361686
8	-2.131022140	-3.555368163	-2.592847767
8	-1.338350900	-3.161496328	-4.969818629
16	-2.834442801	3.064812016	-2.139914358
8	-2.515624090	3.751055934	-3.412597932
8	-4.241541023	2.748223268	-1.847421303
1	4.350527979	-0.561109239	-7.263105148
1	3.151340055	2.326579552	-4.242180687
1	4.163326856	-0.537291550	1.253954554
1	3.101345833	3.370793359	-0.345727028
1	-7.055795132	0.356306685	-4.542505119
1	-4.132329995	-2.721717330	-3.552950352
1	-4.324902768	-3.902176090	-0.137678453
1	-4.097733299	-1.940586600	3.733747540
1	-1.600802649	1.105057218	-3.679082696
16	-6.174126574	-2.792448942	1.885118469
16	-5.898494026	-2.010139865	-5.804490750
16	3.952715175	2.330140960	-7.066316097
16	5.372527606	1.500374555	-0.458688552
9	2.449593866	2.934631100	-6.705688072
9	3.278855436	1.414523998	-8.265032670
9	4.088645435	3.532080756	-8.163737222
9	4.615509353	3.344914888	-5.965191900
9	5.440619918	1.820920916	-7.516568644
9	5.153934886	0.006597842	-1.127805523
9	4.709000058	2.200726373	-1.796919630
9	5.694228432	2.987702684	0.149308860
9	6.153725132	0.810038251	0.808898925
9	6.810888436	1.561793311	-1.229269833
9	-5.987382557	-3.522754631	-5.192337779
9	-7.528652404	-1.863303932	-5.703893400
9	-6.113621363	-2.659030376	-7.295147825
9	-5.829708758	-0.528690496	-6.559751504
9	-4.279496012	-2.197900801	-6.040725736
9	-6.359778211	-4.350051527	1.411048848
9	-6.285811633	-2.303263934	0.320362430
9	-6.194624839	-3.266006447	3.459009122
9	-6.114221681	-1.214283032	2.371930745
9	-7.803651170	-2.681270828	1.966177110
6	-1.864978924	-0.736671066	-7.328783547
6	-0.508554890	-0.210195850	-7.287411467
6	-0.139527812	0.876338284	-8.027314193
6	-2.891697632	-0.225265743	-8.082275599
1	-0.819590689	1.340556841	-8.753556331
1	-2.709173245	0.492510571	-8.896113741
1	-3.885569364	-0.690581029	-8.042884696
1	0.878309499	1.285887002	-7.966224110
6	-2.135394137	3.764682867	-7.192367221
6	-0.672724118	4.235557716	-7.007820666
6	-0.197330277	4.199324632	-5.554108088
6	-0.293199524	2.785738398	-4.981016884
1	-0.003782216	3.598041451	-7.622904055

1	-0.582682210	5.265156761	-7.412889728
1	0.852055610	4.547714052	-5.490625890
1	-0.806293165	4.878379343	-4.925004313
1	0.527775908	2.150335011	-5.382401251
1	-0.181271465	2.770086562	-3.879143454
6	-2.374534710	2.428405946	-6.466139677
6	-3.484065484	1.625453283	-6.701147871
1	-4.230821469	1.903161633	-7.458157060
1	-3.792395355	0.880518232	-5.959433672
6	-1.553770234	2.070621276	-5.349387421
8	-1.903679138	1.018986194	-4.650290351
6	-3.105797409	4.789933639	-6.550264927
1	-2.943796373	5.798760671	-6.983815578
1	-4.157600375	4.497112307	-6.745221001
1	-2.982659390	4.841574988	-5.452218966
6	-2.448881623	3.683955881	-8.696405631
1	-1.773942493	2.983562719	-9.225958379
1	-3.494393748	3.371949137	-8.890727714
1	-2.316923474	4.683067150	-9.158439215
1	-2.084539771	-1.555156949	-6.622627398
6	0.454343953	-0.861469182	-6.338541083
1	1.452093533	-0.392590348	-6.386518681
1	0.082292500	-0.819162254	-5.294616614
1	0.555033098	-1.940300364	-6.562811790
6	0.246756392	-4.517941946	-3.345217446
6	1.353389331	-4.501179181	-4.215039196
6	0.154353048	-5.567387514	-2.412182644
6	2.327818041	-5.507888960	-4.183239653
6	1.138086955	-6.571833221	-2.357901792
6	2.204990630	-6.564278944	-3.267864153
9	-0.835848566	-5.658034550	-1.523099062
9	1.053910033	-7.544711914	-1.446947681
9	3.115408298	-7.535373337	-3.236596532
9	3.377784814	-5.455965063	-5.011809627
9	1.533313970	-3.504381768	-5.086991820
6	-2.242603800	4.176385379	-0.827629082
6	-0.889456971	4.558689658	-0.817153422
6	-3.094104639	4.666637861	0.180703836
6	-0.370646863	5.354097410	0.211620340
6	-2.576978158	5.457508880	1.224177120
6	-1.217997482	5.807103895	1.237426200
9	0.932259398	5.658584221	0.239231083
9	-0.728831927	6.555394501	2.222846069
9	-3.381900590	5.893481178	2.195167146
9	-4.393811954	4.398065316	0.216125932
9	-0.032771546	4.103586822	-1.738377185

TS-C6: Imaginary frequency = - 214.29

15	0.438386974	-1.662731167	-1.566648800
8	1.782219944	-1.101768347	-0.777730247
8	-0.023161381	-2.905832516	-0.579256005
6	2.822066262	-2.033296781	-0.811937567
6	3.828141699	-1.889528184	-1.817573110
6	4.796503349	-2.888590589	-1.875559362
6	4.771251740	-4.022788053	-1.019869603
6	5.746081182	-5.057047524	-1.141822161
6	5.686285124	-6.189520033	-0.345832822

6	4.636811011	-6.335836662	0.599169432
6	3.681657248	-5.341482609	0.753994444
6	3.730201944	-4.152581062	-0.030012910
6	2.761892367	-3.094838964	0.090906384
6	1.682558811	-3.120164153	1.118088382
6	1.999386723	-3.177390848	2.524404248
6	3.339529200	-3.112175711	3.014138808
6	3.606983252	-3.123283118	4.376062430
6	2.550615191	-3.217118897	5.320557448
6	1.237857131	-3.277335995	4.879373746
6	0.928058252	-3.240983543	3.487173575
6	-0.417987347	-3.259719408	3.033940772
6	-0.741768438	-3.167062912	1.684381411
6	0.334916915	-3.047303814	0.743814371
6	3.838339126	-0.752707118	-2.770759836
1	5.607219133	-2.807860037	-2.614570641
1	6.540131438	-4.942926518	-1.895664073
1	6.439905708	-6.983553289	-0.455746802
1	4.574488436	-7.250043551	1.208421166
1	2.865664346	-5.472572329	1.477445092
1	4.167269531	-3.037554361	2.295791909
1	4.648460828	-3.060539424	4.725483911
1	2.776765258	-3.239577778	6.397097115
1	0.406057510	-3.346800140	5.597051753
1	-1.223141476	-3.362066716	3.776761335
6	-2.158536981	-3.233291666	1.246248095
6	3.475223860	0.552994834	-2.376153233
6	3.551670332	1.630476964	-3.263157329
6	3.995972651	1.396233899	-4.568942210
6	4.337590747	0.110623479	-5.007603470
6	4.248024338	-0.956446091	-4.107245534
6	-3.157557895	-2.561095606	1.981089133
6	-4.509794417	-2.670768868	1.641380822
6	-4.866831478	-3.474122654	0.551136813
6	-3.900962143	-4.142779420	-0.213665960
6	-2.551978905	-4.017723523	0.139422431
1	3.138984299	0.740147657	-1.351429212
1	4.450974945	-1.976445621	-4.461226170
1	-2.874690961	-1.925567315	2.829916800
1	-1.796040551	-4.549357210	-0.451304455
7	-0.574828449	-0.442775522	-1.537798746
15	-1.397669709	0.733774761	-0.965214214
8	-0.825587905	1.617571968	0.310261001
8	-2.778267554	0.083322216	-0.355694238
6	-0.893315385	1.139507115	1.621714558
6	0.338684342	0.835932916	2.287912567
6	0.253415863	0.308418230	3.574357240
6	-0.989381119	0.079316778	4.219940990
6	-1.045045278	-0.508645973	5.518856152
6	-2.261024781	-0.763724344	6.132143014
6	-3.470664535	-0.435522170	5.463959498
6	-3.448567977	0.158466730	4.209899525
6	-2.213272467	0.442661584	3.552782123
6	-2.144573822	1.018363768	2.230995515
6	-3.388853593	1.434749242	1.528535326
6	-4.286343091	2.398949121	2.109157930
6	-3.957027917	3.156400313	3.270446123

6	-4.852682003	4.075960786	3.797100318
6	-6.122401987	4.276055538	3.193754496
6	-6.462995352	3.570698419	2.050636189
6	-5.555575855	2.635909125	1.469743637
6	-5.871228038	1.953578145	0.264813131
6	-4.965886460	1.107415718	-0.369515410
6	-3.717347231	0.887858515	0.288714628
6	1.675139592	1.116882618	1.706945951
1	1.178883784	0.075304587	4.121064263
1	-0.097570024	-0.766541199	6.015642547
1	-2.293506724	-1.224241824	7.130841971
1	-4.436299407	-0.655782147	5.943096078
1	-4.392911684	0.396832915	3.703011365
1	-2.973137426	3.017835020	3.739254477
1	-4.573419883	4.659821385	4.686969454
1	-6.826643443	5.002118124	3.626533871
1	-7.434933341	3.733807775	1.560083085
1	-6.859350655	2.120102790	-0.189233041
6	-5.295534459	0.496210279	-1.679403085
6	1.948168674	2.318944167	1.019631831
6	3.245113278	2.632897805	0.595854627
6	4.276939102	1.716666702	0.836236367
6	4.036810655	0.503226445	1.491371658
6	2.740400515	0.219776517	1.930457297
6	-6.026576780	1.243014088	-2.631907579
6	-6.456275026	0.667977406	-3.832846935
6	-6.134742295	-0.671154166	-4.089836020
6	-5.374386144	-1.428047811	-3.192193135
6	-4.956553720	-0.838238788	-1.994107584
1	1.136117855	3.032073605	0.837567285
1	2.551054034	-0.734804983	2.437084929
1	-6.247154717	2.302052211	-2.435049233
1	-4.381397025	-1.442661857	-1.282629500
7	0.703778529	-2.183081790	-3.033762229
7	-1.623321086	1.890050375	-2.123098306
16	1.126297536	-3.666302046	-3.579880922
8	2.309447321	-3.537064697	-4.453097797
8	1.107344870	-4.722321047	-2.546375682
16	-2.168085573	3.476303313	-1.907934916
8	-2.219624325	4.061428293	-3.255923420
8	-3.338542424	3.503532789	-1.021515565
1	4.653777990	-0.065051189	-6.042946996
1	3.281363093	2.639237984	-2.931333130
1	4.846335596	-0.218551431	1.656020037
1	3.453799895	3.580319035	0.085030797
1	-7.029583274	1.258589821	-4.558291673
1	-5.130850433	-2.476163296	-3.400809427
1	-4.186528296	-4.754163125	-1.078957769
1	-5.271914484	-2.136631618	2.222262898
1	-1.188249155	1.695677511	-3.210406089
16	-6.636221098	-3.670699247	0.140520450
16	-6.717045822	-1.447627602	-5.638310621
16	4.134135767	2.806853859	-5.719025814
16	5.968157870	2.099370545	0.262722956
9	2.814075801	3.555516776	-5.075338473
9	3.165705936	2.073657456	-6.841368948
9	4.248377854	4.071315325	-6.752677065

9	5.098854818	3.635362082	-4.686577885
9	5.455355189	2.174564955	-6.456744327
9	6.096869914	0.645977270	-0.492176165
9	5.418407962	2.777650575	-1.128188423
9	5.934915506	3.572545046	0.985010702
9	6.635379695	1.453240496	1.616984532
9	7.481188558	2.437713313	-0.244684913
9	-7.326985698	-2.735399974	-4.839031235
9	-8.166856604	-0.687301027	-5.547587795
9	-7.232364713	-2.125308684	-7.030245590
9	-6.125935814	-0.196365208	-6.549938958
9	-5.297261238	-2.256835889	-5.856492003
9	-6.513585984	-5.304584529	0.140143877
9	-6.334624811	-3.655361077	-1.478009725
9	-7.067770096	-3.703360188	1.724112380
9	-6.869819027	-2.040158173	0.116417583
9	-8.217855005	-3.840631228	-0.227193505
6	-1.199276585	-0.899629669	-5.368228406
6	-0.262317057	-0.464879053	-6.385819604
6	-0.703587746	0.035738582	-7.578632223
6	-2.573147688	-0.911391450	-5.507387792
1	-1.763925722	0.013406304	-7.863713722
1	-3.055304951	-0.890274303	-6.495644525
1	-3.191856382	-1.323653325	-4.699300315
1	0.005552120	0.427965508	-8.323622446
6	-3.028283733	2.850980775	-7.044988107
6	-1.909376909	3.443698255	-7.933101725
6	-0.839069416	4.182328765	-7.132225450
6	-0.144525745	3.243007114	-6.146226984
1	-1.434208463	2.615933668	-8.502903980
1	-2.372700085	4.118832172	-8.683196237
1	-0.087305385	4.635066962	-7.810014883
1	-1.301848196	5.026451307	-6.578736352
1	0.614506998	2.613326534	-6.656865059
1	0.427006488	3.795874282	-5.377045141
6	-2.429257576	2.069853751	-5.865088626
6	-3.190453399	1.170859511	-5.119618847
1	-4.240337394	0.995022351	-5.388194603
1	-2.932245902	0.976123817	-4.073667656
6	-1.075661106	2.308351166	-5.413703300
8	-0.594673151	1.699193787	-4.398890584
6	-3.883099633	3.990696796	-6.432403606
1	-4.299310121	4.641159899	-7.229964024
1	-4.728733621	3.564716068	-5.855083815
1	-3.298911043	4.613563414	-5.727955373
6	-3.942118669	1.980937385	-7.926448839
1	-3.384743059	1.143802746	-8.391732847
1	-4.795430602	1.552521827	-7.367751687
1	-4.362990415	2.595536429	-8.748088403
1	-0.758466153	-1.158238075	-4.392286544
6	1.196600611	-0.467847242	-6.020491711
1	1.356968934	0.189089482	-5.141495128
1	1.540829708	-1.477863227	-5.722479706
1	1.830080703	-0.103673242	-6.849394385
6	-0.322381238	-3.971276270	-4.660392434
6	-0.246810664	-4.045837212	-6.062778546
6	-1.587737900	-4.087436863	-4.058171253

6	-1.412135108	-4.218009398	-6.835588493
6	-2.749711320	-4.277850393	-4.813474609
6	-2.664967836	-4.337130600	-6.214523793
9	-1.731890169	-3.962025485	-2.731633744
9	-3.941632030	-4.375242076	-4.204557711
9	-3.763723781	-4.498231832	-6.946121120
9	-1.328296212	-4.263247763	-8.166512742
9	0.901418617	-3.922909068	-6.723645307
6	-0.791589284	4.288008349	-1.046824160
6	0.453083665	4.375247133	-1.701538285
6	-0.945761339	4.899218065	0.213495200
6	1.524964010	5.067182767	-1.121783798
6	0.128928380	5.595547972	0.799685753
6	1.360523887	5.685453999	0.129354778
9	2.717180623	5.098464800	-1.724550135
9	2.385505510	6.315488527	0.694085440
9	-0.018667549	6.167491126	1.994379122
9	-2.076344104	4.832652589	0.909046308
9	0.658299180	3.767813464	-2.868334287

TS-D1: Imaginary frequency = - 124.51

15	-0.318878546	-1.677417311	-1.839918344
8	1.165443664	-1.071849100	-1.415744578
8	-0.270389006	-3.134695464	-1.073147089
6	2.324969022	-1.824335901	-1.466158333
6	3.341191077	-1.391906115	-2.380494039
6	4.539129611	-2.104914661	-2.381119282
6	4.723919911	-3.277552227	-1.603113268
6	5.919033143	-4.050588442	-1.704614185
6	6.057051381	-5.238636916	-1.005919274
6	4.990665393	-5.711859766	-0.196450143
6	3.828322258	-4.967769882	-0.054154663
6	3.671610646	-3.718377368	-0.723774401
6	2.493006949	-2.900208331	-0.579124863
6	1.513600819	-3.163779248	0.514363449
6	1.935506083	-3.286099472	1.893404190
6	3.285701561	-3.106783865	2.323736334
6	3.639719253	-3.231454502	3.660075640
6	2.665887135	-3.557141468	4.639768014
6	1.341574903	-3.710699583	4.262620425
6	0.939619464	-3.552866147	2.902615693
6	-0.433234091	-3.622911620	2.544420766
6	-0.863378272	-3.451112965	1.232643632
6	0.145769599	-3.241303706	0.240929597
6	3.204089026	-0.195397304	-3.249815616
1	5.373134270	-1.754090164	-3.005180866
1	6.718549927	-3.695072324	-2.372041813
1	6.976074435	-5.834834549	-1.104463186
1	5.080178669	-6.682788171	0.312708672
1	3.003784884	-5.350901063	0.562352011
1	4.058063925	-2.856163508	1.585612686
1	4.687581028	-3.078998402	3.958897174
1	2.962285547	-3.675378018	5.692710205
1	0.567059620	-3.941314895	5.009455828
1	-1.172935734	-3.816782380	3.335198725
6	-2.308941595	-3.471730130	0.894533404
6	2.533612315	0.974222695	-2.828268336

6	2.604711738	2.155537035	-3.572312671
6	3.343187534	2.169196977	-4.757735038
6	3.949002297	1.008682607	-5.254662073
6	3.868363944	-0.165145926	-4.498656578
6	-3.227110695	-2.853944505	1.772677608
6	-4.603956654	-2.893182560	1.530439186
6	-5.064761168	-3.560266525	0.388624145
6	-4.181837276	-4.159121217	-0.518641882
6	-2.806430246	-4.112161545	-0.263511183
1	1.987337273	0.986755722	-1.879390769
1	4.330487536	-1.083345631	-4.888008904
1	-2.860606953	-2.308617031	2.653713725
1	-2.127078969	-4.584076386	-0.984820691
7	-1.343615114	-0.663997552	-1.248935619
15	-1.935526186	0.748881694	-0.869377219
8	-1.125937815	1.542833772	0.359447608
8	-3.319980606	0.275809341	-0.104972391
6	-1.007954506	0.978129038	1.618601488
6	0.308524943	0.624569208	2.064323469
6	0.417004927	-0.031790771	3.288270515
6	-0.721961590	-0.347049614	4.077257330
6	-0.596170162	-1.068542139	5.301424003
6	-1.714472583	-1.406259012	6.047610458
6	-3.006639158	-1.026632763	5.595961005
6	-3.159034780	-0.291740631	4.428427248
6	-2.027780117	0.079014553	3.641105606
6	-2.150485964	0.817095217	2.408867652
6	-3.456316059	1.404691862	1.998737113
6	-4.151803103	2.334478988	2.854375602
6	-3.584273309	2.855627907	4.053431609
6	-4.287045729	3.752532181	4.845890562
6	-5.593163057	4.170452453	4.480526879
6	-6.162789980	3.702728879	3.306639904
6	-5.459700344	2.796209089	2.459674080
6	-6.014782580	2.363787627	1.223621633
6	-5.317004704	1.538124220	0.351386210
6	-4.022514111	1.092033711	0.764047535
6	1.535057760	1.008271228	1.318134908
1	1.412445661	-0.302011423	3.671409494
1	0.411275501	-1.361417683	5.632352366
1	-1.605432386	-1.970103136	6.986283586
1	-3.895124434	-1.312933306	6.178507431
1	-4.162425016	0.000434567	4.089510860
1	-2.568858645	2.552203222	4.342130744
1	-3.822878173	4.148675342	5.761619190
1	-6.143215089	4.876495262	5.120465945
1	-7.165258001	4.037630402	2.998217020
1	-7.020679461	2.712909037	0.945244433
6	-5.849467470	1.185873151	-0.985890130
6	1.677643091	2.282169829	0.722418136
6	2.889463989	2.683899643	0.146341197
6	3.965124408	1.786236910	0.131700900
6	3.849912986	0.505300800	0.683117175
6	2.641594542	0.135367881	1.282160554
6	-6.452354619	2.183394680	-1.782191342
6	-6.914117354	1.898327028	-3.070481024
6	-6.767734817	0.594135099	-3.559207095

6	-6.201582010	-0.427399852	-2.789418347
6	-5.743924549	-0.121790719	-1.504323537
1	0.833479909	2.981412253	0.731835526
1	2.551957243	-0.864243180	1.722416812
1	-6.499374117	3.215782769	-1.410282300
1	-5.296412553	-0.917939130	-0.899703576
7	-0.504172153	-2.062009919	-3.403721825
7	-2.001647532	1.791889928	-2.079039452
16	-0.514005721	-3.613223751	-3.985825925
8	-1.603428856	-4.409786221	-3.387582123
8	-0.457687178	-3.506373746	-5.462585809
16	-2.571453284	3.320622263	-2.009235948
8	-2.555100529	3.862293861	-3.383460773
8	-3.805944897	3.457921026	-1.207264919
1	4.493156255	1.019786530	-6.207195134
1	2.126047732	3.068277621	-3.204116900
1	4.690396006	-0.199355169	0.650744625
1	2.994935927	3.686311785	-0.286485579
1	-7.350132642	2.689020953	-3.693171257
1	-6.123420372	-1.452547633	-3.169476277
1	-4.551536237	-4.667852234	-1.417619981
1	-5.302988995	-2.404046185	2.220412277
1	-1.828275073	-1.166260196	-4.094822630
16	-6.862628534	-3.666534846	0.084719684
16	-7.266847875	0.233214402	-5.270898405
16	3.575896301	3.756561124	-5.630424335
16	5.552543916	2.291105347	-0.619024313
9	2.012351731	4.209390622	-5.392844063
9	3.156179918	3.094057353	-7.087891108
9	3.790450038	5.172305413	-6.420287554
9	4.005326401	4.522999500	-4.249892567
9	5.150605517	3.420495262	-5.943605635
9	5.613390336	0.929131254	-1.544970449
9	4.809828964	3.093028592	-1.842078620
9	5.590505243	3.674267443	0.260022651
9	6.414919885	1.524460511	0.551281815
9	6.977727971	2.732938632	-1.283121420
9	-7.937087419	-1.205257437	-4.867963517
9	-8.718041549	0.969314610	-5.101320455
9	-7.673558858	-0.087250125	-6.829723283
9	-6.615859998	1.649473218	-5.817673270
9	-5.826986066	-0.535216636	-5.579037868
9	-6.760997548	-5.245151522	-0.345670181
9	-6.679930890	-3.230295141	-1.496237024
9	-7.162863980	-4.106771432	1.636049605
9	-7.092747262	-2.088611100	0.495250041
9	-8.468601050	-3.756874690	-0.194157274
6	0.191462021	1.979873493	-6.565184480
6	0.643341888	0.688484582	-7.064510618
6	0.402268725	-0.472053529	-6.385985684
6	-0.486604581	2.212026603	-5.394033094
1	-0.056152382	-0.484002009	-5.390243788
1	-0.580172467	1.438341502	-4.616797755
1	-0.710426640	3.236986881	-5.071498261
1	0.728090714	-1.448575654	-6.773516320
6	-3.365397788	0.533220714	-8.007336812
6	-3.995871052	-0.852138667	-8.292357206

6	-3.172416095	-2.011646802	-7.738535427
6	-3.054128352	-1.910824006	-6.219139917
1	-5.012096490	-0.880600742	-7.847262069
1	-4.127927953	-0.953580923	-9.389890575
1	-3.631415824	-2.983745713	-8.007259437
1	-2.152759062	-2.017419413	-8.179226398
1	-4.029548992	-2.157356505	-5.737757011
1	-2.315772075	-2.634560502	-5.822219380
6	-2.943317661	0.633613810	-6.525079842
6	-2.712821215	1.862828696	-5.908692472
1	-2.721024738	1.955562223	-4.814128920
1	-2.872340408	2.797305625	-6.464471849
6	-2.731876530	-0.536669118	-5.737225344
8	-2.316513609	-0.368311792	-4.501533433
6	-2.137704199	0.744192320	-8.917832686
1	-2.457746389	0.771815527	-9.979746305
1	-1.631912397	1.704111190	-8.693204978
1	-1.390083714	-0.061091131	-8.796389935
6	-4.414239861	1.611510407	-8.348570090
1	-5.289231779	1.550498184	-7.676479247
1	-3.997017070	2.636422266	-8.286175615
1	-4.765858563	1.467703493	-9.390532845
1	0.395665947	2.845739997	-7.217806831
6	1.383811530	0.690412642	-8.379313610
1	1.627513620	-0.334907069	-8.714392668
1	0.792494814	1.186884295	-9.176393698
1	2.327511302	1.264593189	-8.282397357
6	1.069906853	-4.391400006	-3.543610001
6	2.228016532	-3.976964776	-4.229710488
6	1.140979840	-5.534081460	-2.723265808
6	3.421930752	-4.704597511	-4.139838154
6	2.335334988	-6.270574754	-2.633751399
6	3.462602135	-5.879189476	-3.371695756
9	0.111723724	-5.957999698	-1.992981521
9	2.404293564	-7.342431838	-1.841178383
9	4.583754475	-6.591829131	-3.299816596
9	4.521484960	-4.277013952	-4.768720567
9	2.228582184	-2.870057694	-4.976290654
6	-1.281827349	4.249907897	-1.093511642
6	-0.075068337	4.575637794	-1.736591507
6	-1.450546167	4.644730527	0.248331102
6	0.937316484	5.287195389	-1.076813262
6	-0.444280258	5.366327377	0.918518631
6	0.753124301	5.685089947	0.256952777
9	2.101201129	5.534371151	-1.690414901
9	1.724038583	6.333416093	0.896965083
9	-0.615809397	5.730045536	2.191174666
9	-2.537325455	4.342619643	0.953949484
9	0.170737803	4.193433936	-2.993895213

TS-D3: Imaginary frequency = - 130.21

15	-0.333372368	-1.705312921	-1.869669258
8	1.144326934	-1.094598752	-1.432523632
8	-0.306321339	-3.142629270	-1.063689314
6	2.303570514	-1.847944211	-1.468683911
6	3.325451080	-1.424040068	-2.380143594
6	4.521776682	-2.139683290	-2.368176533

6	4.698667276	-3.306877106	-1.579770388
6	5.892172573	-4.083960203	-1.668025188
6	6.022957604	-5.266530157	-0.958562242
6	4.951243271	-5.730063364	-0.150556206
6	3.790280185	-4.981522713	-0.020708042
6	3.640639494	-3.737895475	-0.702267297
6	2.463792696	-2.915254151	-0.570817248
6	1.480678945	-3.157994309	0.523860168
6	1.902712873	-3.263095923	1.904446135
6	3.254832006	-3.089869032	2.331677183
6	3.608929570	-3.200430718	3.669247300
6	2.633338006	-3.505044352	4.653875326
6	1.307318539	-3.650667160	4.279814399
6	0.905771471	-3.506244480	2.918270712
6	-0.467797975	-3.567052370	2.562179556
6	-0.897786197	-3.411280828	1.248215023
6	0.111660976	-3.227283703	0.251615377
6	3.193998596	-0.233395491	-3.258279608
1	5.360540982	-1.795738055	-2.989679223
1	6.696285107	-3.736613728	-2.334218851
1	6.940763890	-5.866106237	-1.047381427
1	5.035544462	-6.696836931	0.367279596
1	2.961328022	-5.356389041	0.595062894
1	4.029289621	-2.856150309	1.590321931
1	4.658459777	-3.053606762	3.965036180
1	2.929729176	-3.612919519	5.707939265
1	0.531221738	-3.863579793	5.030148274
1	-1.208612837	-3.742018247	3.356319755
6	-2.345223376	-3.428379444	0.916988762
6	2.518724642	0.938681468	-2.850610974
6	2.593450012	2.114853419	-3.602850394
6	3.343497571	2.120326846	-4.781124610
6	3.954326094	0.956811630	-5.264822717
6	3.867968238	-0.211850623	-4.501899901
6	-3.252878281	-2.787313122	1.789511096
6	-4.632462295	-2.831390859	1.565228198
6	-5.107441116	-3.529783815	0.448337239
6	-4.235303344	-4.151744667	-0.453988643
6	-2.856050079	-4.095458606	-0.220133983
1	1.964481066	0.957269356	-1.906518444
1	4.333071059	-1.132823956	-4.881030196
1	-2.875092702	-2.221085798	2.652356188
1	-2.184159957	-4.583867945	-0.937963774
7	-1.362431272	-0.659617833	-1.351894167
15	-1.943055701	0.739200557	-0.911902996
8	-1.103795263	1.522762791	0.304405064
8	-3.295866207	0.239858499	-0.109072496
6	-0.981797446	0.981505979	1.573626670
6	0.338373372	0.641796847	2.020260142
6	0.458252028	0.014583519	3.258102282
6	-0.673340060	-0.283377775	4.063826823
6	-0.535879900	-0.972497996	5.305176703
6	-1.647263123	-1.293941961	6.068581795
6	-2.943789932	-0.930389980	5.616591542
6	-3.107064330	-0.225797259	4.431995834
6	-1.982972288	0.129136341	3.626993394
6	-2.117582406	0.837837575	2.378149424

6	-3.431304174	1.407908407	1.969287138
6	-4.130636529	2.346051071	2.812209306
6	-3.556900877	2.903300742	3.991662811
6	-4.264472402	3.806948817	4.772041852
6	-5.581601733	4.195270938	4.413392508
6	-6.157565492	3.692296206	3.257315921
6	-5.449873252	2.778426371	2.422011973
6	-6.011573585	2.311904882	1.201402373
6	-5.309692842	1.481037910	0.336910588
6	-4.005538198	1.062872188	0.747547580
6	1.560272632	1.016378122	1.262105620
1	1.457692079	-0.243437047	3.639279458
1	0.474907490	-1.253800280	5.635647046
1	-1.529341301	-1.832728854	7.020794476
1	-3.827246321	-1.204650295	6.212413738
1	-4.114197906	0.053941043	4.093942885
1	-2.533092595	2.622729949	4.273953570
1	-3.795813046	4.231808933	5.672471238
1	-6.135332931	4.906876721	5.043922354
1	-7.168675537	4.004716876	2.953590022
1	-7.025954184	2.639692505	0.928167149
6	-5.846213040	1.097974020	-0.990248549
6	1.699499801	2.285746476	0.656595608
6	2.908871720	2.684449105	0.073338601
6	3.985008744	1.787148817	0.062089053
6	3.872619502	0.510041600	0.622856124
6	2.666794957	0.143626420	1.229126474
6	-6.503332134	2.063852515	-1.783054594
6	-6.978697668	1.750073894	-3.059798709
6	-6.786106268	0.450142602	-3.544263687
6	-6.159108228	-0.539456902	-2.780229372
6	-5.694050291	-0.207174408	-1.504740935
1	0.854427332	2.983811762	0.664701411
1	2.579307069	-0.851842133	1.679200604
1	-6.586756451	3.096435998	-1.417814021
1	-5.200947333	-0.980326461	-0.905743807
7	-0.494913514	-2.126730140	-3.427393415
7	-2.052694399	1.797672495	-2.103897520
16	-0.511402383	-3.689921507	-3.975433354
8	-1.598239013	-4.473113514	-3.356139328
8	-0.458750486	-3.611911698	-5.454282218
16	-2.617730114	3.326766637	-2.019348416
8	-2.592557805	3.881167196	-3.388338361
8	-3.856096586	3.456929329	-1.222692818
1	4.506101537	0.961989960	-6.212966245
1	2.108019461	3.029133435	-3.246210276
1	4.713473951	-0.194249186	0.592766752
1	3.011031385	3.683456942	-0.368133945
1	-7.462170286	2.516738870	-3.677328611
1	-6.041763238	-1.561954407	-3.157063507
1	-4.618851367	-4.688970113	-1.330221980
1	-5.322962322	-2.323607166	2.250295763
1	-1.817153596	-1.254741189	-4.121040418
16	-6.909466542	-3.647273470	0.173034247
16	-7.308903728	0.052357597	-5.241285783
16	3.583704366	3.698826709	-5.666931772
16	5.570439248	2.286871957	-0.696429565

9	2.016015216	4.151432971	-5.452890936
9	3.182280193	3.016571084	-7.120618251
9	3.803739822	5.104997313	-6.471807685
9	3.994345112	4.482645619	-4.290986822
9	5.162663226	3.363226782	-5.957245713
9	5.627241542	0.920182456	-1.615775073
9	4.824392115	3.083073842	-1.920957302
9	5.612341048	3.674344520	0.175524651
9	6.436258862	1.525503573	0.474785451
9	6.993750591	2.724214743	-1.367478215
9	-7.850997375	-1.434992286	-4.822777347
9	-8.807236783	0.671434095	-5.022685139
9	-7.736634233	-0.298743950	-6.787007359
9	-6.791706160	1.516561865	-5.804987358
9	-5.822203044	-0.599345252	-5.594856810
9	-6.811348896	-5.241132778	-0.199058900
9	-6.750160409	-3.270609849	-1.424408976
9	-7.187221036	-4.030408025	1.743965746
9	-7.134381958	-2.055962452	0.528501671
9	-8.519272102	-3.749312400	-0.078690460
6	0.164000610	1.934209542	-6.525382135
6	0.600415188	0.662124176	-7.082609889
6	0.365394521	-0.524693604	-6.447431752
6	-0.511305836	2.118935504	-5.343727074
1	-0.066892088	-0.579523383	-5.441233594
1	-0.604729395	1.312820674	-4.600054756
1	-0.729018444	3.130370533	-4.976768237
1	0.677502754	-1.484283949	-6.886023109
6	-3.398817109	0.524277966	-7.985457865
6	-3.979000485	-0.871616220	-8.320877826
6	-3.111818218	-2.018576636	-7.809601686
6	-3.005926354	-1.971543433	-6.286693048
1	-4.992194550	-0.955330535	-7.875288098
1	-4.111043044	-0.937058384	-9.421122236
1	-3.530041277	-2.997062813	-8.118654582
1	-2.091100757	-1.965080279	-8.245085297
1	-3.975482187	-2.270179148	-5.823117064
1	-2.245671215	-2.684451955	-5.909891271
6	-2.960034737	0.582218121	-6.506218685
6	-2.732632118	1.794249867	-5.853929078
1	-2.736791012	1.849556364	-4.756897093
1	-2.898328543	2.745990439	-6.377792990
6	-2.733372695	-0.606438084	-5.751107185
8	-2.347403325	-0.468431159	-4.501724733
6	-2.198337058	0.825788321	-8.905663215
1	-2.542052969	0.904983862	-9.957454321
1	-1.716642996	1.786749778	-8.635181108
1	-1.426677385	0.036662293	-8.847999626
6	-4.495204258	1.574368623	-8.260398108
1	-5.353189622	1.450826221	-7.575513731
1	-4.115065220	2.610499883	-8.159655877
1	-4.862177583	1.462156641	-9.301020599
1	0.371181697	2.825762116	-7.141109340
6	1.315546593	0.714710485	-8.410416050
1	1.556959576	-0.297133247	-8.787092313
1	0.706183640	1.237041657	-9.176799700
1	2.257171452	1.291286678	-8.311214831

6	1.075840178	-4.454208717	-3.522295782
6	2.232743476	-4.038542729	-4.209817808
6	1.150986141	-5.590270060	-2.693389736
6	3.429409125	-4.760785226	-4.114337214
6	2.348016590	-6.322038359	-2.599380657
6	3.473968085	-5.930918240	-3.339428148
9	0.123254917	-6.011481635	-1.959552126
9	2.420934700	-7.388627372	-1.800004514
9	4.597791775	-6.639129744	-3.263335492
9	4.528311952	-4.332267034	-4.743872318
9	2.228609204	-2.936325595	-4.962958981
6	-1.326921563	4.246159185	-1.092814880
6	-0.114075492	4.566511838	-1.727704711
6	-1.503956379	4.646284927	0.246101232
6	0.893331813	5.281437251	-1.063969828
6	-0.499704649	5.364664498	0.922792352
6	0.701434017	5.682758138	0.267786351
9	2.060001468	5.529922213	-1.672402152
9	1.666900544	6.335543325	0.911323228
9	-0.680051427	5.732391323	2.193111058
9	-2.599219328	4.353668148	0.942771737
9	0.143148634	4.177130315	-2.980868688

TS-D3: Imaginary frequency =   - 173.17

15	-0.145844830	-1.883452082	-1.746727436
8	1.271765142	-1.191643431	-1.229783904
8	-0.209358172	-3.207298068	-0.753264268
6	2.409044161	-1.983776774	-1.241877834
6	3.381722395	-1.728082869	-2.261209643
6	4.549416817	-2.488880421	-2.227010295
6	4.739099107	-3.544271610	-1.296289009
6	5.912316148	-4.356535898	-1.334385352
6	6.053125443	-5.441067800	-0.485357257
6	5.007395675	-5.773908804	0.416010567
6	3.864497694	-4.991828350	0.491639033
6	3.711084110	-3.834569500	-0.328857866
6	2.559737608	-2.969018774	-0.254348262
6	1.563171164	-3.112818295	0.847335764
6	1.970816128	-3.130491904	2.234035236
6	3.314075182	-2.897504155	2.660479651
6	3.658934741	-2.928283909	4.004549290
6	2.682761976	-3.210895447	4.995614176
6	1.364829147	-3.415240501	4.619615621
6	0.971514186	-3.351015314	3.249752586
6	-0.394442862	-3.476597241	2.882145435
6	-0.817428884	-3.390601350	1.558904131
6	0.196073807	-3.213399213	0.560624535
6	3.196662584	-0.700070323	-3.318604588
1	5.352009183	-2.277931896	-2.948598590
1	6.692383917	-4.116491187	-2.072502971
1	6.955536336	-6.068005000	-0.533118184
1	5.094312959	-6.670996412	1.046534602
1	3.053976059	-5.273977616	1.176560871
1	4.087331382	-2.682763054	1.911293213
1	4.701057665	-2.736892116	4.301165745
1	2.972455003	-3.256702536	6.056109557
1	0.588688784	-3.613295477	5.373886157
1	-1.135597319	-3.646876116	3.677016569

6	-2.261152206	-3.471174213	1.216674460
6	2.588554122	0.553219868	-3.078681000
6	2.538231690	1.535353682	-4.074584023
6	3.096711319	1.262608140	-5.328475667
6	3.672899554	0.019574918	-5.617345291
6	3.709386158	-0.952451831	-4.612557466
6	-3.203932058	-2.870583491	2.080904281
6	-4.576519578	-2.938991500	1.826416904
6	-5.013144416	-3.629482252	0.688786925
6	-4.108717525	-4.230689972	-0.195017586
6	-2.736113095	-4.148191462	0.070317135
1	2.175862606	0.784552081	-2.089693275
1	4.128615955	-1.941422590	-4.843610091
1	-2.859443294	-2.308770201	2.959218431
1	-2.045353754	-4.618507018	-0.639930999
7	-1.231738915	-0.793109651	-1.434285454
15	-1.958031212	0.438228091	-0.801577846
8	-1.180131897	1.310286187	0.379545586
8	-3.209571019	-0.230835108	0.029121687
6	-1.052684314	0.855380030	1.687319525
6	0.281408939	0.665728319	2.176787426
6	0.439494463	0.134104338	3.452543582
6	-0.674866504	-0.205066031	4.265889443
6	-0.497427527	-0.782154448	5.558204728
6	-1.588169845	-1.146907571	6.331480637
6	-2.903860511	-0.943464203	5.836524473
6	-3.108819723	-0.351649521	4.598275604
6	-2.007764990	0.047985559	3.781372313
6	-2.188521583	0.645764036	2.480911799
6	-3.541973384	1.055445748	2.011224877
6	-4.349005647	1.984462889	2.762481090
6	-3.896605786	2.624877580	3.953316493
6	-4.716636302	3.501099486	4.650003645
6	-6.030580565	3.781985787	4.192294049
6	-6.488318324	3.204173870	3.019226931
6	-5.662978254	2.316063246	2.267494035
6	-6.095064185	1.802404896	1.015391581
6	-5.286175677	0.984411053	0.234051198
6	-4.025747507	0.603024390	0.785897503
6	1.472415325	1.088212799	1.394698636
1	1.453888849	-0.014899509	3.852575389
1	0.528662470	-0.942993031	5.920349154
1	-1.438912150	-1.597637500	7.324150859
1	-3.769528119	-1.253525556	6.440802690
1	-4.131282482	-0.197424156	4.227363295
1	-2.878737925	2.428636579	4.315146294
1	-4.340611041	3.987588145	5.562623192
1	-6.674108984	4.471683829	4.758609528
1	-7.493662437	3.436270378	2.635178140
1	-7.088012541	2.092844800	0.640418565
6	-5.667335182	0.637837970	-1.157227567
6	1.554295490	2.382973389	0.839020004
6	2.705913971	2.805825911	0.165459561
6	3.778123104	1.915107238	0.028785027
6	3.737134930	0.630374891	0.580897771
6	2.586131517	0.235135171	1.271842698
6	-6.228071459	1.640977807	-1.979416447

6	-6.622014867	1.367441048	-3.293863855
6	-6.449344983	0.068852949	-3.787261074
6	-5.876647455	-0.946234845	-3.010094282
6	-5.479767273	-0.650851920	-1.701605963
1	0.719432011	3.080851237	0.975615188
1	2.541062305	-0.768385291	1.708726443
1	-6.311897704	2.668676090	-1.599519941
1	-5.034985583	-1.444846562	-1.092449522
7	-0.045464283	-2.460415684	-3.236294524
7	-2.367066016	1.520365728	-1.941760870
16	-0.398599679	-4.020282697	-3.603557882
8	-1.589064894	-4.533178842	-2.891973526
8	-0.346238362	-4.167221607	-5.070419306
16	-2.851526125	3.080595256	-1.668205796
8	-3.632906429	3.524981587	-2.839828355
8	-3.402398067	3.291128714	-0.317341654
1	4.085746443	-0.192521672	-6.611362464
1	2.089977012	2.513036537	-3.867853507
1	4.582977957	-0.059805505	0.469262155
1	2.782219073	3.826617052	-0.225895854
1	-7.040713716	2.161857665	-3.923600147
1	-5.758670672	-1.964994218	-3.399310260
1	-4.455446537	-4.765061840	-1.088344323
1	-5.291617944	-2.453313718	2.501976963
1	-1.835456841	0.968461994	-3.362874503
16	-6.805879103	-3.749900126	0.360188321
16	-6.992875552	-0.308432374	-5.490777338
16	3.093905771	2.562119872	-6.613233276
16	5.257856031	2.449292021	-0.896603087
9	1.495626813	2.893150470	-6.364392364
9	2.680727013	1.481302974	-7.801536871
9	3.077692126	3.715410070	-7.771462232
9	3.506757307	3.724347361	-5.540057754
9	4.674776324	2.311850058	-6.961249713
9	5.332751033	1.025083271	-1.718882152
9	4.341039006	3.100110767	-2.100522719
9	5.282706495	3.903656955	-0.137825096
9	6.268658535	1.836738042	0.241728654
9	6.577365746	2.926163918	-1.730539399
9	-7.607759644	-1.759639468	-5.051861352
9	-8.449778856	0.398020293	-5.267175110
9	-7.448978568	-0.650201643	-7.023130691
9	-6.393127273	1.117788937	-6.074384909
9	-5.541061720	-1.044323368	-5.851017723
9	-6.717717984	-5.368691213	0.121332710
9	-6.595840939	-3.507376731	-1.257872728
9	-7.148128869	-4.000393140	1.946101820
9	-7.013866166	-2.128886203	0.577586033
9	-8.409276754	-3.849221921	0.063866645
6	-1.014815191	-2.241745057	-7.617974232
6	-2.316455323	-2.350759945	-7.009283820
6	-2.510953126	-1.934471269	-5.716613051
6	0.144422114	-1.869193866	-6.954619065
1	-1.682392414	-1.644265333	-5.059094679
1	0.228006933	-2.071492019	-5.877991359
1	1.091540625	-1.858659361	-7.514354124
1	-3.513192512	-1.969337359	-5.265761399

6	-1.906282630	1.456252800	-7.791432924
6	-2.411945426	2.841063940	-7.301571419
6	-3.500663584	2.750731013	-6.224263655
6	-3.306986603	1.552479383	-5.263971750
1	-1.531383846	3.389563235	-6.903607037
1	-2.783771238	3.423195721	-8.171008872
1	-3.508218889	3.679213100	-5.620449863
1	-4.502485285	2.664101899	-6.687238745
1	-3.574405875	1.830651129	-4.230015849
1	-3.995941736	0.735618756	-5.554580709
6	-1.262730476	0.797490362	-6.557290879
6	0.022391633	0.241069037	-6.566986373
1	0.507818539	0.083568032	-5.594300270
1	0.686646934	0.442526137	-7.411556349
6	-1.936337577	0.958748763	-5.311662355
8	-1.322922740	0.641777787	-4.201085854
6	-3.071448830	0.635124868	-8.387490220
1	-3.552074694	1.213027135	-9.203865698
1	-2.703011894	-0.311841170	-8.824204063
1	-3.853875074	0.378279874	-7.649519380
6	-0.862870846	1.681577793	-8.899183352
1	-0.002891331	2.279673247	-8.538437046
1	-0.474807691	0.722622167	-9.298748270
1	-1.328295239	2.226373761	-9.744803698
1	-0.973304683	-2.371606607	-8.714862203
6	-3.456736686	-2.884884847	-7.842585458
1	-4.438923298	-2.630155557	-7.403015032
1	-3.427353725	-2.495320621	-8.880665309
1	-3.387901822	-3.991216139	-7.911707313
6	1.064348121	-4.954781308	-3.020627006
6	2.276566745	-4.765484575	-3.710548669
6	1.013510521	-5.930999832	-2.010589583
6	3.404670411	-5.548703218	-3.433029434
6	2.143261293	-6.717178035	-1.719278352
6	3.325214403	-6.554362568	-2.456960248
9	-0.077692866	-6.144757184	-1.272911821
9	2.097963212	-7.623484464	-0.738142706
9	4.382282122	-7.323334761	-2.197252355
9	4.552402888	-5.333577567	-4.086412428
9	2.399257966	-3.816114850	-4.644945541
6	-1.270912761	3.985375286	-1.765626314
6	-0.616470217	4.101460006	-3.007694279
6	-0.670335927	4.569294790	-0.636227489
6	0.611759072	4.767614031	-3.127427476
6	0.558759855	5.242373712	-0.748968699
6	1.211959379	5.325180697	-1.986783282
9	1.227484594	4.842449073	-4.307303527
9	2.405440320	5.906051549	-2.070326788
9	1.151722242	5.733752517	0.343970743
9	-1.192274857	4.476413503	0.586715786
9	-1.132611128	3.565914296	-4.117286390

TS-D4: Imaginary frequency = - 110.19

15	-0.390349211	-1.727830931	-1.754014300
8	1.058940341	-1.104246610	-1.247446148
8	-0.321597348	-3.207244426	-1.030933503
6	2.243989823	-1.808568900	-1.322576969
6	3.251865186	-1.279480543	-2.192001015

6	4.478309786	-1.941521809	-2.216857547
6	4.701247550	-3.145999809	-1.498576705
6	5.931986539	-3.858786037	-1.618078910
6	6.115608272	-5.071806440	-0.975275764
6	5.061469737	-5.632820811	-0.206615686
6	3.863117324	-4.951789819	-0.049707971
6	3.657554229	-3.679132468	-0.659890031
6	2.440770157	-2.923788067	-0.493327669
6	1.455486806	-3.274840519	0.570007997
6	1.864490928	-3.456757132	1.947301470
6	3.202192555	-3.257763314	2.407048999
6	3.544370404	-3.446792663	3.738958828
6	2.571315335	-3.861519044	4.684799053
6	1.257406837	-4.035030495	4.281065928
6	0.866214272	-3.809949314	2.927491884
6	-0.500771232	-3.892719811	2.550708146
6	-0.921034889	-3.653504740	1.246902160
6	0.091511757	-3.368183910	0.277787535
6	3.058940860	-0.039221931	-2.985690701
1	5.306565679	-1.522311435	-2.805406305
1	6.723943337	-3.434524508	-2.253569142
1	7.063077536	-5.619109656	-1.087066995
1	5.189306398	-6.622135441	0.256855155
1	3.048339498	-5.402163092	0.533390337
1	3.976905609	-2.943125017	1.696684340
1	4.582908577	-3.276662694	4.059932945
1	2.859082926	-4.031399781	5.733037079
1	0.482032552	-4.332970628	5.002864369
1	-1.244113771	-4.145566876	3.321128619
6	-2.362056329	-3.661096982	0.890274825
6	2.298679964	1.055568558	-2.513416870
6	2.277096146	2.271955761	-3.201050909
6	3.029902926	2.403363485	-4.371519255
6	3.742536087	1.323902379	-4.906911875
6	3.740979700	0.109089788	-4.214942205
6	-3.290273184	-3.082618258	1.784121303
6	-4.661120602	-3.079549710	1.506560709
6	-5.103777371	-3.663365357	0.313436814
6	-4.211364615	-4.233899568	-0.602308496
6	-2.842892392	-4.232109474	-0.310338172
1	1.744699293	0.983101817	-1.571942277
1	4.279850414	-0.748375235	-4.642188319
1	-2.936232815	-2.595373720	2.703568586
1	-2.154887912	-4.677946431	-1.040051826
7	-1.464725883	-0.747765978	-1.191338028
15	-1.932090759	0.713836528	-0.787865011
8	-1.074407429	1.391119053	0.468396262
8	-3.369294149	0.339270321	-0.074126397
6	-1.007228067	0.800884236	1.716107796
6	0.294292164	0.429703521	2.191891892
6	0.368337335	-0.293993970	3.378179287
6	-0.798115673	-0.641836993	4.114379122
6	-0.718670794	-1.433708786	5.297848079
6	-1.861608945	-1.785794150	5.999276468
6	-3.133851377	-1.348000856	5.543788807
6	-3.241391413	-0.544271511	4.417245487
6	-2.084422043	-0.161337613	3.674442902

6	-2.167778632	0.644678044	2.482036802
6	-3.434992893	1.333282675	2.104593114
6	-4.079931352	2.250568436	3.015370722
6	-3.513528187	2.624767828	4.269671331
6	-4.165138098	3.512041299	5.115067540
6	-5.417500311	4.070647424	4.751000722
6	-5.983518961	3.748965707	3.527379977
6	-5.333342059	2.853283339	2.628484609
6	-5.891007569	2.565203778	1.350970809
6	-5.246171579	1.735095521	0.446717501
6	-4.004828347	1.152118789	0.846439816
6	1.528863516	0.897000623	1.507710209
1	1.352810699	-0.587317244	3.773564338
1	0.273705092	-1.767849985	5.634873019
1	-1.786941953	-2.404155268	6.906503702
1	-4.041821574	-1.643323005	6.090774942
1	-4.228759790	-0.206123138	4.073274215
1	-2.538428527	2.213354524	4.562401521
1	-3.700744489	3.789407473	6.073437321
1	-5.927786448	4.768068427	5.432049058
1	-6.943727379	4.191991179	3.220781216
1	-6.851061620	3.025433142	1.071630048
6	-5.765366195	1.478682563	-0.918870459
6	1.660759850	2.242640988	1.095101947
6	2.847162510	2.711269515	0.519339911
6	3.910466109	1.818178021	0.335220182
6	3.820742355	0.483067947	0.744885950
6	2.633312553	0.039096240	1.339600452
6	-6.058719217	2.553638937	-1.781540699
6	-6.467408909	2.322808754	-3.100238139
6	-6.596442210	0.999941686	-3.537905654
6	-6.346078324	-0.092223213	-2.698489457
6	-5.922498549	0.158920514	-1.390932609
1	0.832597548	2.943965145	1.249874636
1	2.554928307	-1.004421984	1.664736022
1	-5.890013633	3.582982245	-1.437811051
1	-5.716641934	-0.688353828	-0.727473256
7	-0.512707329	-2.081143120	-3.331927843
7	-1.888157399	1.778372497	-1.982285519
16	-0.455234398	-3.613790569	-3.956951549
8	-1.532129177	-4.462661195	-3.407148411
8	-0.374779682	-3.464363385	-5.428131412
16	-2.557627822	3.268424159	-2.004632296
8	-3.173580964	3.481254878	-3.331411057
8	-3.350032982	3.585818527	-0.791013978
1	4.302124107	1.427829582	-5.844650289
1	1.707682879	3.115146347	-2.792119148
1	4.659518849	-0.209090417	0.597871974
1	2.936480744	3.764438785	0.226841122
1	-6.645434207	3.164195611	-3.780583884
1	-6.476867453	-1.124833159	-3.042807436
1	-4.568040423	-4.680851559	-1.538392581
1	-5.367398923	-2.616763727	2.207103660
1	-1.856679524	-1.230589564	-4.016306271
16	-6.891401898	-3.684061258	-0.057716368
16	-7.051322995	0.685530642	-5.271052840
16	3.134878455	4.033154719	-5.185812374

16	5.444022225	2.412182809	-0.458944192
9	1.501245141	4.250126416	-5.113433088
9	2.969252869	3.374716944	-6.691908286
9	3.224204552	5.493116510	-5.911677549
9	3.297729897	4.810801080	-3.745369431
9	4.762512520	3.929429558	-5.321916481
9	5.479164814	1.128813510	-1.493461590
9	4.612707454	3.288970717	-1.573528606
9	5.512206612	3.726118254	0.519248226
9	6.386419111	1.575934047	0.595729444
9	6.818540880	2.937359936	-1.168841804
9	-8.115366058	-0.493248702	-4.871940884
9	-8.256289243	1.792780264	-5.252278361
9	-7.426492611	0.396705338	-6.848169590
9	-6.003106109	1.840701515	-5.814930914
9	-5.862235429	-0.461123623	-5.437054454
9	-6.820681191	-5.207386443	-0.657597913
9	-6.637487396	-3.080447997	-1.575294071
9	-7.259782162	-4.278447207	1.424748474
9	-7.096392368	-2.150597593	0.515280806
9	-8.487743035	-3.690653794	-0.399306587
6	0.127167118	1.892111229	-6.532219987
6	0.694560157	0.626321670	-6.978098961
6	0.499860043	-0.535248672	-6.290512758
6	-0.614573779	2.102215552	-5.398491351
1	-0.026978436	-0.569232216	-5.328807071
1	-0.711975184	1.337129619	-4.614489738
1	-0.900305670	3.122707789	-5.121144904
1	0.913689520	-1.490681892	-6.642980715
6	-3.342148685	0.206448345	-8.031860945
6	-4.042568199	-1.162255975	-8.212785302
6	-3.288956117	-2.317048237	-7.558145752
6	-3.160930655	-2.094894170	-6.052278553
1	-5.062295593	-1.094292326	-7.777950296
1	-4.171827111	-1.346968275	-9.299771532
1	-3.807171149	-3.278827079	-7.743664093
1	-2.273120288	-2.423503443	-7.993137055
1	-4.146875279	-2.253452481	-5.554121181
1	-2.457903044	-2.815535599	-5.594471664
6	-2.999801777	0.420075897	-6.541167356
6	-2.831829314	1.686094268	-5.988207392
1	-2.892770916	1.842038409	-4.901497457
1	-2.992876728	2.581295656	-6.604795117
6	-2.790578485	-0.698346801	-5.678933438
8	-2.342945002	-0.454648968	-4.469842442
6	-2.049199517	0.264232418	-8.872963363
1	-2.294528839	0.156508750	-9.949751168
1	-1.540981771	1.239863272	-8.737621292
1	-1.328956584	-0.525940636	-8.591144047
6	-4.295760008	1.299590644	-8.553694801
1	-5.223442035	1.351493666	-7.956533266
1	-3.821490520	2.301253773	-8.551821845
1	-4.570689430	1.075083299	-9.604223226
1	0.308050063	2.758932534	-7.190903568
6	1.505026595	0.659694826	-8.250490760
1	1.880420175	-0.345039740	-8.522815426
1	0.907430369	1.051125725	-9.100525100

1	2.372987754	1.341597965	-8.132660923
6	1.142035917	-4.355178722	-3.498732154
6	2.303112156	-3.885674342	-4.143073299
6	1.227191383	-5.525792012	-2.720062416
6	3.512455021	-4.588214235	-4.061288716
6	2.437492714	-6.236724955	-2.637532916
6	3.567509452	-5.791393185	-3.339817868
9	0.196298852	-6.001163650	-2.024893930
9	2.519779954	-7.335035364	-1.883336943
9	4.703862192	-6.480280856	-3.274064340
9	4.611040084	-4.110143147	-4.653604156
9	2.291714083	-2.749330916	-4.844789722
6	-1.111763164	4.402538621	-1.925227696
6	-0.643744507	5.136405185	-3.034618905
6	-0.478715177	4.622140602	-0.685295518
6	0.478116183	5.981341093	-2.933276877
6	0.661874792	5.431453129	-0.581705275
6	1.151354633	6.108061738	-1.709636919
9	0.899443787	6.669302327	-3.991503731
9	2.232059703	6.875121712	-1.610319892
9	1.285058701	5.569603878	0.597070330
9	-0.931646031	4.066199527	0.440170986
9	-1.222133172	5.068611164	-4.235794342

TS-D5: Imaginary frequency = - 189.98

15	0.191682572	-1.029426603	-1.707252816
8	1.842246010	-0.985133881	-1.619102325
8	-0.179647739	-2.462302297	-0.938863886
6	2.650732091	-2.108154678	-1.556661335
6	3.609990062	-2.288095813	-2.601845669
6	4.400899736	-3.431527027	-2.557995236
6	4.271685243	-4.408335181	-1.535244219
6	5.061115312	-5.595791678	-1.541880844
6	4.912092633	-6.555751126	-0.553470525
6	3.954907902	-6.362528089	0.476414121
6	3.182213318	-5.210188228	0.518484834
6	3.326424433	-4.191886778	-0.470042975
6	2.542793675	-2.979716002	-0.471876976
6	1.593344287	-2.717098449	0.643852382
6	2.038383132	-2.637767316	2.009806400
6	3.419331835	-2.574591133	2.362824725
6	3.810447183	-2.460990313	3.689826559
6	2.840920453	-2.432201212	4.728056359
6	1.491167534	-2.492654019	4.416473613
6	1.054355304	-2.573106690	3.061086168
6	-0.327484903	-2.618107802	2.728877617
6	-0.770216499	-2.638932437	1.407705692
6	0.228295194	-2.600557661	0.379705960
6	3.870899820	-1.280302477	-3.658024904
1	5.167896049	-3.579370838	-3.332980070
1	5.786254567	-5.738573431	-2.358007388
1	5.522781558	-7.470798519	-0.572017200
1	3.817854010	-7.136316495	1.246818179
1	2.437524951	-5.084941008	1.315704758
1	4.173408336	-2.602070869	1.563600073
1	4.880284777	-2.398152747	3.939435965
1	3.164503793	-2.361913224	5.777537411
1	0.728798579	-2.469183561	5.209276653

1	-1.062903061	-2.660013969	3.546406571
6	-2.213259138	-2.801360989	1.100646100
6	4.040793853	0.083400032	-3.339489361
6	4.596174986	0.976060542	-4.265039264
6	4.949682376	0.496943292	-5.531406418
6	4.682053524	-0.822444607	-5.921053392
6	4.135203941	-1.700696708	-4.980363989
6	-3.190820583	-2.163413750	1.892917712
6	-4.556497687	-2.368695948	1.670929956
6	-4.951541768	-3.236286565	0.645296511
6	-4.011435500	-3.892218921	-0.160071577
6	-2.648878108	-3.667698929	0.071782142
1	3.808682153	0.444602646	-2.330578319
1	3.918224181	-2.739485396	-5.266632785
1	-2.878421812	-1.473745643	2.687485972
1	-1.914495901	-4.182503336	-0.559249662
7	-0.205914724	0.216531478	-0.798048268
15	-1.399677729	1.222713836	-0.611681236
8	-1.088377045	2.224915729	0.666284279
8	-2.659198084	0.345408224	0.013963462
6	-0.963298083	1.670218858	1.933234007
6	0.356151017	1.508528658	2.461320759
6	0.472835753	0.974876128	3.740228694
6	-0.661876462	0.563767862	4.489833832
6	-0.509745513	-0.008747304	5.787792433
6	-1.602528401	-0.497131447	6.486106631
6	-2.894547122	-0.432875805	5.901895832
6	-3.080302226	0.152449565	4.657062192
6	-1.981313368	0.691128954	3.921156969
6	-2.123384927	1.303457096	2.618919739
6	-3.461230672	1.455427809	1.982773741
6	-4.547531520	2.104079919	2.670400493
6	-4.357862931	2.883936125	3.849390152
6	-5.437097456	3.465183941	4.499031862
6	-6.757960371	3.284077856	4.009828029
6	-6.971197246	2.561295526	2.847499871
6	-5.879924293	1.979378777	2.135698352
6	-6.078818356	1.292707691	0.908516915
6	-5.022021081	0.791151976	0.150951865
6	-3.710141896	0.904470530	0.717889855
6	1.562365140	1.872202352	1.677993017
1	1.471725841	0.856606768	4.185851107
1	0.502569339	-0.067485494	6.215502455
1	-1.471296706	-0.942847663	7.483428125
1	-3.759963295	-0.846732352	6.440728423
1	-4.089154542	0.195636086	4.226829245
1	-3.339233933	3.030078945	4.235226668
1	-5.266130186	4.077140009	5.397194615
1	-7.606437441	3.739407753	4.541700795
1	-7.986551387	2.442313808	2.439280295
1	-7.108218698	1.162632711	0.545203479
6	-5.273580049	0.147462078	-1.161198234
6	1.655810529	3.095797085	0.980621833
6	2.815617237	3.434284761	0.276392366
6	3.893276767	2.539471278	0.274408001
6	3.831611454	1.313353052	0.947808172
6	2.662055224	0.988518287	1.644535517

6	-6.360425829	0.580595176	-1.953830580
6	-6.675890261	-0.040154853	-3.164715316
6	-5.882107620	-1.110557628	-3.595288561
6	-4.782353528	-1.549619174	-2.854599842
6	-4.486910548	-0.920002753	-1.643842755
1	0.820746958	3.805784703	0.993869333
1	2.596656317	0.014163597	2.148964147
1	-6.950896621	1.451134483	-1.637470542
1	-3.637977515	-1.293557691	-1.065147910
7	-0.459185890	-1.047148982	-3.168546678
7	-1.708763961	2.179142775	-1.867929374
16	0.136665256	-1.883033704	-4.443427847
8	0.499166397	-0.932203182	-5.515166426
8	1.116162671	-2.926631543	-4.047318141
16	-2.888689245	3.304086862	-2.161534691
8	-2.335305176	4.240856261	-3.159514639
8	-4.181837473	2.645352885	-2.441156108
1	4.918742376	-1.167443831	-6.935174090
1	4.798263661	2.012315496	-3.969462559
1	4.674558348	0.611412794	0.924834898
1	2.876897033	4.390612075	-0.256960524
1	-7.520268017	0.313995222	-3.768697141
1	-4.173648954	-2.398317650	-3.181513010
1	-4.326689139	-4.558964194	-0.972925062
1	-5.301937750	-1.848132417	2.285023211
1	-0.856005628	1.972027724	-3.254161197
16	-6.733970476	-3.498882010	0.345764922
16	-6.289602748	-1.954899299	-5.155779897
16	5.875817517	1.584128929	-6.667433903
16	5.413451853	2.990542963	-0.632236565
9	4.845422565	2.845732724	-6.425351214
9	4.988998768	1.091465736	-7.970297791
9	6.701323530	2.555003791	-7.698120501
9	6.834435583	2.147284388	-5.464017362
9	6.976554087	0.411384132	-6.992273707
9	5.663244199	1.439275980	-1.126874122
9	4.552130540	3.290986666	-2.013457559
9	5.280565614	4.570137831	-0.213120539
9	6.357674796	2.717643029	0.677994433
9	6.760307562	3.392251001	-1.462684509
9	-6.016460565	-3.440728747	-4.501980293
9	-7.875494886	-2.068178957	-4.775599008
9	-6.625575604	-2.715140299	-6.563717752
9	-6.576601337	-0.531057544	-5.935882997
9	-4.710541744	-1.897131654	-5.657805651
9	-6.542287657	-5.125529533	0.272884347
9	-6.551675036	-3.406265813	-1.282881434
9	-7.046079718	-3.607635143	1.955782784
9	-7.046900505	-1.881311305	0.404521025
9	-8.331107285	-3.731237840	0.088306268
6	2.095407883	3.730850865	-7.145657130
6	2.144888214	2.391870674	-7.691553540
6	1.848815700	1.303357418	-6.922706216
6	1.682919767	4.068226393	-5.872752323
1	1.614310353	1.367371459	-5.851531913
1	1.622413299	3.311490975	-5.077009630
1	1.774583491	5.112739943	-5.540071509

1	1.850658150	0.286208042	-7.338388407
6	-1.815299132	2.461761907	-7.620820197
6	-3.230427399	1.974521816	-7.203145140
6	-3.181745335	0.760353020	-6.279270032
6	-2.390864675	1.056853345	-5.001986917
1	-3.761794394	2.811328669	-6.698207453
1	-3.809173360	1.733305804	-8.119583102
1	-4.205085748	0.444341575	-6.010839386
1	-2.726738978	-0.099255925	-6.809868328
1	-3.063700851	1.542269538	-4.255501894
1	-2.020156095	0.147184203	-4.485845884
6	-1.083221763	2.834167029	-6.319860814
6	-0.472593102	4.078384048	-6.081477659
1	-0.459536254	4.429035352	-5.039214804
1	-0.533112033	4.858415349	-6.851599667
6	-1.254867082	2.011423179	-5.167942250
8	-0.439821227	2.218659522	-4.165405406
6	-1.097074540	1.330784207	-8.391550104
1	-1.747816204	0.962622050	-9.212241768
1	-0.155308053	1.697466886	-8.837678727
1	-0.839135789	0.476461309	-7.739448033
6	-1.964541326	3.670961362	-8.556540680
1	-2.520469754	4.501012583	-8.074532131
1	-0.980062934	4.056725561	-8.891667082
1	-2.525649720	3.372123126	-9.464554237
1	2.324547061	4.553585810	-7.846529476
6	2.514592405	2.267889299	-9.149414904
1	2.488372445	1.216194666	-9.489726284
1	1.829517412	2.862010940	-9.791020534
1	3.541550117	2.651819730	-9.316716193
6	-1.323169238	-2.801834261	-5.046949449
6	-1.677515381	-2.838072313	-6.409470344
6	-2.013706718	-3.655526850	-4.168445321
6	-2.714133844	-3.673196956	-6.863822467
6	-3.057265651	-4.481236382	-4.605585827
6	-3.396159773	-4.508907842	-5.967523071
9	-1.725594792	-3.668813353	-2.862595379
9	-3.737414157	-5.224987284	-3.726160997
9	-4.390815970	-5.278560081	-6.394962191
9	-3.056658613	-3.663651339	-8.152866437
9	-1.086364911	-2.072660317	-7.329922193
6	-3.075645555	4.226876751	-0.612611964
6	-1.932878735	4.807438863	-0.033186241
6	-4.322407418	4.407727185	0.016449177
6	-1.997360446	5.430663779	1.219537916
6	-4.401532595	5.075652409	1.250932585
6	-3.238634550	5.565672099	1.864345379
9	-0.884757595	5.892425190	1.799794332
9	-3.312578127	6.150448092	3.057690040
9	-5.583823834	5.248111018	1.841665542
9	-5.462385853	3.955754939	-0.500205359
9	-0.742283929	4.715248820	-0.628457180

TS-D6: Imaginary frequency = - 188.47

15	0.187024000	-1.028612000	-1.710405000
8	1.837769000	-0.983888000	-1.625463000
8	-0.182789000	-2.461763000	-0.941794000
6	2.646829000	-2.106528000	-1.563881000

6	3.605851000	-2.285426000	-2.609487000
6	4.396649000	-3.429009000	-2.567099000
6	4.267977000	-4.406700000	-1.545127000
6	5.057213000	-5.594271000	-1.553231000
6	4.909103000	-6.554703000	-0.565136000
6	3.953127000	-6.361822000	0.465938000
6	3.180637000	-5.209395000	0.509418000
6	3.323839000	-4.190712000	-0.478860000
6	2.540211000	-2.978556000	-0.479324000
6	1.592596000	-2.716214000	0.638022000
6	2.040009000	-2.636522000	2.003167000
6	3.421557000	-2.573245000	2.353803000
6	3.814910000	-2.458713000	3.680065000
6	2.847130000	-2.428979000	4.719901000
6	1.496850000	-2.489698000	4.410655000
6	1.057745000	-2.571309000	3.056076000
6	-0.324653000	-2.616848000	2.726244000
6	-0.769653000	-2.638392000	1.405848000
6	0.227126000	-2.599972000	0.376176000
6	3.868087000	-1.275852000	-3.663654000
1	5.163458000	-3.576049000	-3.342418000
1	5.781498000	-5.736729000	-2.370172000
1	5.519634000	-7.469831000	-0.584801000
1	3.816868000	-7.135939000	1.236152000
1	2.436876000	-5.084366000	1.307545000
1	4.174282000	-2.601248000	1.553326000
1	4.885169000	-2.395768000	3.927832000
1	3.172488000	-2.357778000	5.768770000
1	0.735823000	-2.465579000	5.204739000
1	-1.058652000	-2.658598000	3.545053000
6	-2.213146000	-2.801350000	1.101263000
6	4.039081000	0.087031000	-3.342116000
6	4.597607000	0.980755000	-4.264774000
6	4.953224000	0.503650000	-5.531300000
6	4.684286000	-0.814531000	-5.924191000
6	4.134160000	-1.693868000	-4.986413000
6	-3.189552000	-2.163293000	1.894850000
6	-4.555547000	-2.368478000	1.674804000
6	-4.952071000	-3.236127000	0.649791000
6	-4.013136000	-3.892479000	-0.156592000
6	-2.650239000	-3.668069000	0.073369000
1	3.805572000	0.446529000	-2.332896000
1	3.916435000	-2.731823000	-5.275150000
1	-2.875985000	-1.473459000	2.688798000
1	-1.916778000	-4.183130000	-0.558522000
7	-0.209248000	0.216770000	-0.799786000
15	-1.403069000	1.222512000	-0.610966000
8	-1.090425000	2.224219000	0.667091000
8	-2.661430000	0.344537000	0.015960000
6	-0.965088000	1.669765000	1.934101000
6	0.354490000	1.508523000	2.462023000
6	0.471585000	0.974963000	3.740930000
6	-0.662838000	0.563493000	4.490761000
6	-0.510262000	-0.009329000	5.788540000
6	-1.602791000	-0.497968000	6.487069000
6	-2.895005000	-0.433597000	5.903296000
6	-3.081183000	0.151968000	4.658643000

6	-1.982443000	0.690787000	3.922458000
6	-2.124953000	1.303054000	2.620235000
6	-3.463029000	1.455011000	1.984567000
6	-4.549110000	2.103804000	2.672398000
6	-4.359190000	2.883386000	3.851532000
6	-5.438257000	3.464663000	4.501421000
6	-6.759192000	3.283948000	4.012270000
6	-6.972660000	2.561530000	2.849760000
6	-5.881574000	1.979508000	2.137756000
6	-6.080769000	1.293107000	0.910464000
6	-5.024194000	0.791142000	0.152872000
6	-3.712277000	0.903970000	0.719796000
6	1.560502000	1.872593000	1.678577000
1	1.470608000	0.857026000	4.186335000
1	0.502177000	-0.068012000	6.215981000
1	-1.471217000	-0.943944000	7.484227000
1	-3.760230000	-0.847614000	6.442310000
1	-4.090173000	0.195155000	4.228723000
1	-3.340502000	3.029265000	4.237310000
1	-5.267096000	4.076362000	5.399722000
1	-7.607537000	3.739316000	4.544320000
1	-7.988067000	2.442872000	2.441581000
1	-7.110225000	1.163507000	0.547136000
6	-5.275912000	0.147524000	-1.159285000
6	1.653408000	3.096188000	0.981142000
6	2.813033000	3.435125000	0.276841000
6	3.891044000	2.540744000	0.274840000
6	3.829968000	1.314663000	0.948359000
6	2.660591000	0.989402000	1.645193000
6	-6.362119000	0.581424000	-1.952390000
6	-6.677309000	-0.038902000	-3.163589000
6	-5.883992000	-1.109772000	-3.593864000
6	-4.785088000	-1.549791000	-2.852505000
6	-4.489820000	-0.920515000	-1.641545000
1	0.818086000	3.805851000	0.994461000
1	2.595702000	0.015097000	2.149772000
1	-6.952097000	1.452379000	-1.636251000
1	-3.641415000	-1.294708000	-1.062496000
7	-0.466595000	-1.046048000	-3.170464000
7	-1.714054000	2.179343000	-1.866277000
16	0.126808000	-1.878447000	-4.448555000
8	0.480456000	-0.924917000	-5.520994000
8	1.112650000	-2.918087000	-4.057870000
16	-2.892969000	3.305442000	-2.159250000
8	-2.338760000	4.241955000	-3.157003000
8	-4.186800000	2.648112000	-2.438818000
1	4.922655000	-1.157863000	-6.938488000
1	4.800557000	2.016128000	-3.966733000
1	4.673219000	0.613084000	0.925437000
1	2.873893000	4.391450000	-0.256571000
1	-7.521021000	0.316008000	-3.768057000
1	-4.176778000	-2.398833000	-3.179167000
1	-4.329577000	-4.559376000	-0.968850000
1	-5.300082000	-1.847693000	2.289809000
1	-0.861481000	1.973171000	-3.251217000
16	-6.734917000	-3.497938000	0.352143000
16	-6.290285000	-1.953119000	-5.155208000

16	5.884205000	1.591452000	-6.662827000
16	5.410862000	2.992306000	-0.632098000
9	4.854260000	2.853774000	-6.422621000
9	5.001253000	1.101411000	-7.969304000
9	6.714220000	2.562728000	-7.689422000
9	6.839320000	2.152059000	-5.455460000
9	6.984745000	0.417906000	-6.985426000
9	5.660297000	1.441306000	-1.127827000
9	4.548965000	3.293691000	-2.012798000
9	5.278224000	4.571623000	-0.211925000
9	6.355657000	2.718515000	0.677527000
9	6.757370000	3.394430000	-1.462916000
9	-6.019326000	-3.439501000	-4.501621000
9	-7.876756000	-2.065220000	-4.777042000
9	-6.625151000	-2.712573000	-6.563863000
9	-6.575230000	-0.528757000	-5.935092000
9	-4.710582000	-1.896530000	-5.655236000
9	-6.543984000	-5.124644000	0.278552000
9	-6.554317000	-3.404859000	-1.276656000
9	-7.045298000	-3.607039000	1.962471000
9	-7.047126000	-1.880255000	0.411715000
9	-8.332425000	-3.729572000	0.096350000
6	2.100886000	3.729208000	-7.137292000
6	2.152532000	2.391254000	-7.684991000
6	1.857239000	1.301406000	-6.917699000
6	1.682363000	4.064350000	-5.865441000
1	1.621580000	1.363835000	-5.846696000
1	1.620482000	3.306498000	-5.070846000
1	1.773568000	5.108240000	-5.530615000
1	1.860063000	0.284872000	-7.334829000
6	-1.804641000	2.452301000	-7.622900000
6	-3.223129000	1.970860000	-7.209667000
6	-3.181747000	0.760429000	-6.280472000
6	-2.395406000	1.059931000	-5.001074000
1	-3.754544000	2.811290000	-6.710845000
1	-3.798268000	1.727245000	-8.127753000
1	-4.207033000	0.448149000	-6.014988000
1	-2.726695000	-0.102457000	-6.805620000
1	-3.069831000	1.551534000	-4.260098000
1	-2.030633000	0.151188000	-4.479156000
6	-1.077949000	2.828720000	-6.320123000
6	-0.467798000	4.073736000	-6.082586000
1	-0.461472000	4.427789000	-5.041330000
1	-0.526704000	4.851723000	-6.854943000
6	-1.255339000	2.009896000	-5.166398000
8	-0.442899000	2.217985000	-4.161997000
6	-1.086158000	1.315137000	-8.384412000
1	-1.735340000	0.942676000	-9.204409000
1	-0.142735000	1.677607000	-8.830517000
1	-0.831206000	0.464731000	-7.726065000
6	-1.945622000	3.657225000	-8.565318000
1	-2.502559000	4.490950000	-8.090851000
1	-0.958181000	4.039030000	-8.896317000
1	-2.501927000	3.355226000	-9.475242000
1	2.331994000	4.553189000	-7.836034000
6	2.522143000	2.269787000	-9.143073000
1	2.500441000	1.218182000	-9.483983000

1	1.833689000	2.861065000	-9.783733000
1	3.547142000	2.658594000	-9.310941000
6	-1.331533000	-2.802836000	-5.047632000
6	-1.685910000	-2.844807000	-6.410033000
6	-2.020392000	-3.654926000	-4.166294000
6	-2.721474000	-3.682908000	-6.861312000
6	-3.063015000	-4.483466000	-4.600312000
6	-3.402320000	-4.516314000	-5.961993000
9	-1.732015000	-3.663882000	-2.860460000
9	-3.741744000	-5.225183000	-3.718045000
9	-4.395885000	-5.288971000	-6.386544000
9	-3.063760000	-3.678852000	-8.150441000
9	-1.095665000	-2.082301000	-7.333407000
6	-3.078607000	4.227750000	-0.609892000
6	-1.935205000	4.807523000	-0.030947000
6	-4.324893000	4.408922000	0.020020000
6	-1.998619000	5.430386000	1.222011000
6	-4.402944000	5.076541000	1.254740000
6	-3.239437000	5.565780000	1.867618000
9	-0.885446000	5.891454000	1.801737000
9	-3.312373000	6.150218000	3.061192000
9	-5.584797000	5.249388000	1.846239000
9	-5.465395000	3.957551000	-0.495994000
9	-0.745047000	4.714999000	-0.627038000

Reference:

(1) Paul, K.; Tanasari, B.; Andrei, G. US2005/288523, **2005**, A1

(2) Hirata, T.; Shimoda, K.; Gondai, T. Asymmetric Hydrogenation of the C-C Double Bond of Enones with the Reductases from *Nicotiana tabacum. Chemistry Letters* **2000**, 850–851.

(3) Amecke, R.; Groth, U.; Köhler, T. *α*-Alkylidenecyclopentanones by α-Alkylidation of Methyl 2-Oxocyclopentanecarboxylate. *Liebigs Ann. Chem.* **1994**, 891–894.

(4) Börner, C.; Dennis, M. R.; Sinn, E.; Woodward, S. Copper-Catalysed Asymmetric 1,4-Addition of Organozinc Compounds to Linear Aliphatic Enones Using 2,2'-Dihydroxy 3,3'-Dithioether Derivatives of 1,1'-Binaphthalene. *Eur. J. Org. Chem.* **2001**, 2435–2446.

(5) Li, J.; Zhu, Y.; Lu, Y.; Wang, Y.; Liu, Y.; Liu, D.; Zhang, W. RuPHOX-Ru-Catalyzed Selective Asymmetric Hydrogenation of Exocyclic *α*,*β*-Unsaturated Pentanones. *Organometallics* **2019**, *38*, 3970−3978.

(6) L. K. Wong, H.-T. Chen, Z.-Z- Ji, US5250735, **1993**, A.

(7) (a) Sano, S.; Matsumoto, T.; Nanataki, H.; Tempaku, S.; Nakao, M. Z-Selective Horner–Wadsworth–Emmons reaction of 2-TOM-cyclopentanone for the synthesis of rac-N-Cbz-Gly-Ψ[(Z)-CF=C]-Pro-OH dipeptide isostere. *Tetrahedron Letters* **2014**, *55*, 6248–6251. (b) Bugarin, A.; Jones K. D.; Connell. B. T. Efficient, direct *α*-methylenation of carbonyls mediated by diisopropylammonium trifluoroacetate. *Chem. Commun*., **2010**, *46*, 1715–1717.

(8) (a) Reuvers, J. T. A.; Groot, A. de. Synthesis of *δ*-substituted *δ*-lactone. *Synthesis* **1982**, 1105. (b) For the synthesis and characterization of **5j**, **5l**, **5m**: Li, Y. P.; Li, Z. Q.; Zhou, B.; Li, M. L.; Xue, X. S.; Zhu, S. F.; Zhou, Q. L. Chiral Spiro Phosphoric Acid Catalyzed Friedel–Crafts Conjugate Addition/Enantioselective Protonation Reactions. *ACS Catal.* **2019**, *9*, 6522−6529. (c) For the characterization of compound 5n: Janssen, J.; Lüttke, W. Synthese von 4,4,4',4'-Tetramethyl[bi-2,5-cyclohexadien-l-yliden] und einiger Derivate *Chem. Ber.* **1982**, *115*, 1234–1243.

(9) (a) Moritz, B. J.; Mack, D. J.; Tong, L.; Thomson, R. J. Total Synthesis of the Isodon Diterpene Sculponeatin N. *Angew. Chem. Int. Ed.* **2014**, *53*, 2988 –2991. (b) Chen, R.; Qiu, D.; Lei, X.; Niu, Y.; Hua, Y.; Peng, H.; Zeng, T.; Zhang, Y. Total Synthesis and Assignment of the Absolute Configuration of (+)-Omphalic Acid. *Org. Lett*., **2021**, *23*, 6972–6976. (c) Sawada, T.; Nakada, M. Enantioselective Total Synthesis of (+)-Colletoic Acid via Catalytic Asymmetric Intramolecular Cyclopropanation of an *α*-Diazo-*β*-keto Diphenylphosphine Oxide. *Org. Lett.*, **2013**, *15*, 1004–1007.

(10) (a) Properzi, R.; Kaib, P. S. J.; Leutzsch, M.; Pupo, G.; Mitra, R.; De, C. K.; Song, L.; Schreiner, P. R.; List, B. Catalytic enantiocontrol over a non-classical carbocation. *Nature Chemistry* **2020**, *12*, 1174–1179. (b) Wipf, P.; Jung, J.-K. Formal Total Synthesis of (+)-Diepoxin σ. *J. Org. Chem.* **2000**, *65*, 6319–6337. (c) He, H.; Chen, L-Y.; Wong, W-Y.; Chan, W-H.; Lee, Al. W. M. Practical Synthetic Approach to Chiral Sulfonimides (CSIs) – Chiral Brønsted Acids for Organocatalysis. *Eur. J. Org. Chem.* **2010**, 4181–4184. (d) Wu, K.; Jiang, Y-J.; Fan, Y-S.; Sha, D.; Zhang, S. Double Axially Chiral Bisphosphorylimides Catalyzed Highly Enantioselective and Efficient Friedel–Crafts Reaction of Indoles with Imines. *Chem. Eur. J.* **2013**, *19*, 474–478.

(11) (a) Gatzenmeier, T.; Turberg, M.; Yepes, D.; Xie, Y.; Neese, F.; Bistoni, G.; List, B., Scalable and Highly Diastereo- and Enantioselective Catalytic Diels–Alder Reaction of *α*, *β*-Unsaturated Methyl Esters. *J. Am. Chem. Soc.* **2018**, *140*, 12671−12676. (b) Lee, S.; Bae, H. Y.; List, B. Can a Ketone Be More Reactive than an Aldehyde? Catalytic Asymmetric Synthesis of Substituted Tetrahydrofurans. *Angew.Chem. Int. Ed.* **2018**, *57*, 12162–12166. (c) Kaib, P. S.; Schreyer, L.; Lee, S.; Properzi, R.; List, B. Extremely Active Organocatalysts Enable a Highly Enantioselective Addition of Allyltrimethylsilane to Aldehydes. *Angew. Chem. Int. Ed.* **2016**, *55*, 13200–13203. (d) Lee, S.; Kaib, P. S. J.; List, B. Asymmetric Catalysis via Cyclic, Aliphatic Oxocarbenium Ions. *J. Am. Chem. Soc.* **2017**, *139*, 2156−2159. (e) For the synthesis of Boronic Ester for the BINOL-**22b** please see the ref. Díaz-Oviedo, C. D.; Maji, R.; List, B. *J. Am. Chem. Soc.* **2021**, *143*, 20598–20604. (f) Schwengers, S. A.; De, C. K.; Grossmann, O.; Grimm, J. A. A.; Sadlowski, N. R.; Gerosa, G. G.; List, B. *J. Am. Chem. Soc.* **2021**, *143*, 14835–14844.

(12) Matsuo, A.; Nakayama, N.; Nakayama, M. Enantiomeric Type Sesquiterpenoids of the Liverwort *Marchantia Polymorpha*. *Phytochemistry,* **1985**, *24*, 777–781.

(13) (a) Ohta, Y.; Hirose, Y. New Sesquiterpenoids From *Schisandra chinensis. Tetrahedron Lett*. **1968**, *9*, 2483–2485. (b) White, D. E.; Stewart. I. C.; Seashore-Ludlow, B. A.; Grubbs, R. H.; Stoltz, B. M. A General Enantioselective Route to the Chamigrene Natural Product Family. *Tetrahedron* **2010**, *66*, 4668–4686.

(14) Asakawa, Y.; Tori, M.; Masuya, T.; Frahm, J.-P. Ent-sesquiterpenoids and cyclic bis(bibenzyls) from the German liverwort marchantia Polymorpha. *Phytochemistry* **1990**, *29*, 1577–1584.

(15) Neese, F. Wiley Interdiscip. *Rev.: Comput. Mol. Sci.* **2011**, *2*, 73–78.

(16) Zhang, Y.; Yang, W. Comment on "Generalized gradient approximation made simple". *Phys. Rev. Lett.* **1998**, *80*, 890–890.

(17) (a) Grimme, S.; Antony, J.; Ehrlich, S.; Krieg, H. A consistent and accurate *ab initio* parametrization of density functional dispersion correction (DFT-D) for the 94 elements H-Pu. *J. Chem. Phys.* **2010**, *132*, 154104. (b) Grimme, S.; Ehrlich, S.; Goerigk, L. Effect of the damping function in dispersion corrected density functional theory. *J. Comput. Chem.* **2011**, *32*, 1456–1465.

(18) Weigend, F.; Ahlrichs, R. Balanced basis sets of split valence, triple zeta valence and quadruple zeta valence quality for H to Rn: Design and assessment of accuracy. *Phys. Chem. Chem. Phys.* **2005**, *7*, 3297–3305.

(19) Barone, V.; Cossi, M. Quantum Calculation of Molecular Energies and Energy Gradients in Solution by a Conductor Solvent Model. *J. Phys. Chem. A* **1998**, *102*, 1995-2001.

(20) Becke, A. D. Density-functional thermochemistry. III. The role of exact exchange. *J. Chem. Phys.* **1993**, *98*, 5648–5652.

(21) Zhao, Y.; Truhlar, D. G. The M06 suite of density functionals for main group thermochemistry, thermochemical kinetics, noncovalent interactions, excited states, and transition elements: two new functionals and systematic testing of four M06-class functionals and 12 other functionals. *Theor. Chem. Acc.* **2008**, *120*, 215–241.

(22) Bader, R. F. W. *Chem. Rev.* **1991**, *91*, 893–928.

(23) Bickelhaupt, F. M.; Houk, K. N., Analyzing Reaction Rates with the Distortion/Interaction-Activation Strain Model. *Angew.Chem. Int. Ed.* **2017,** *56*, 10070–10086

(24) Legault, C. Y.: CYLview, 1.0b; Université de Sherbrooke, 2009 (http://www.cylview.org).

(25) Ghosh, S.; Das, S.; De, C. K.; Yepes, D.; Neese, F.; Bistoni, G.; Leutzsch, M.; List, B., Strong and Confined Acids Control Five Stereogenic Centers in Catalytic Asymmetric Diels–Alder Reactions of Cyclohexadienones with Cyclopentadiene. *Angew.Chem. Int. Ed.* **2020,** *59*, 12347−12351

NMR Traces

^{1}H NMR ($CDCl_3$, 501 MHz) of (5a)

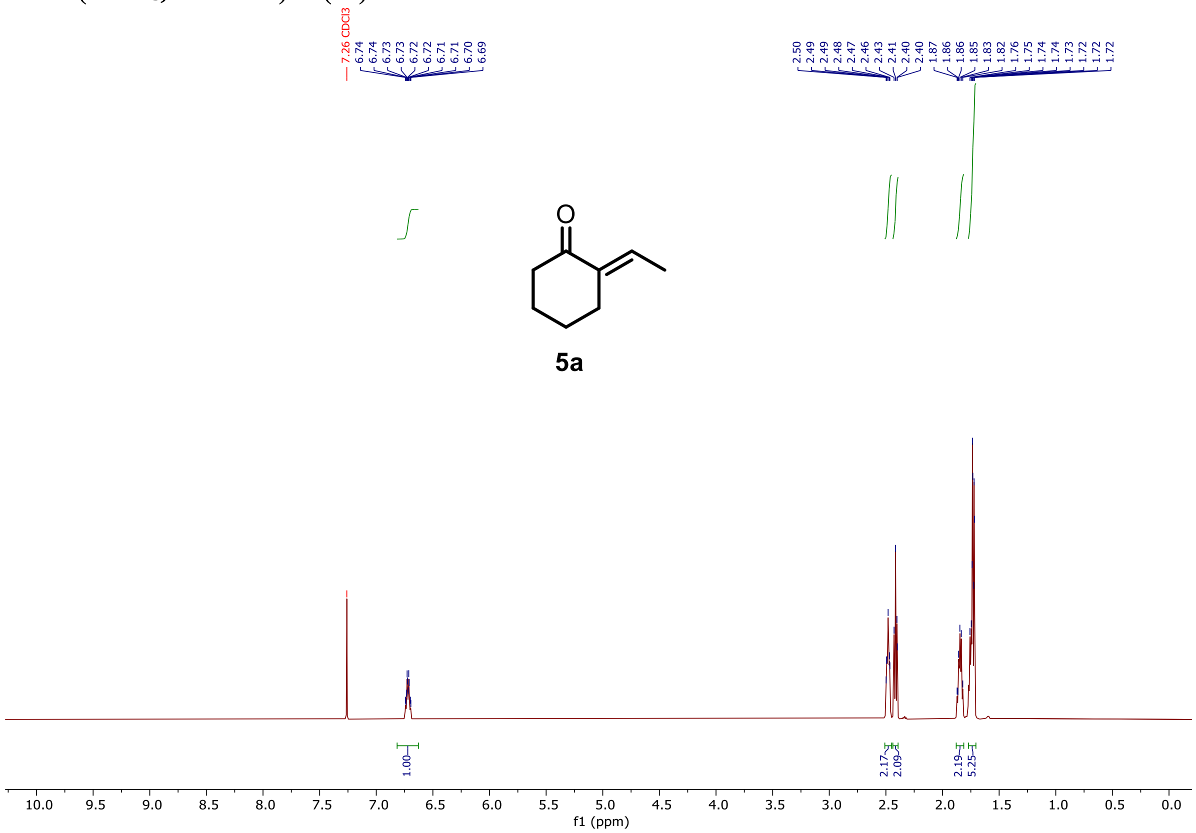

^{13}C NMR ($CDCl_3$, 126 MHz)

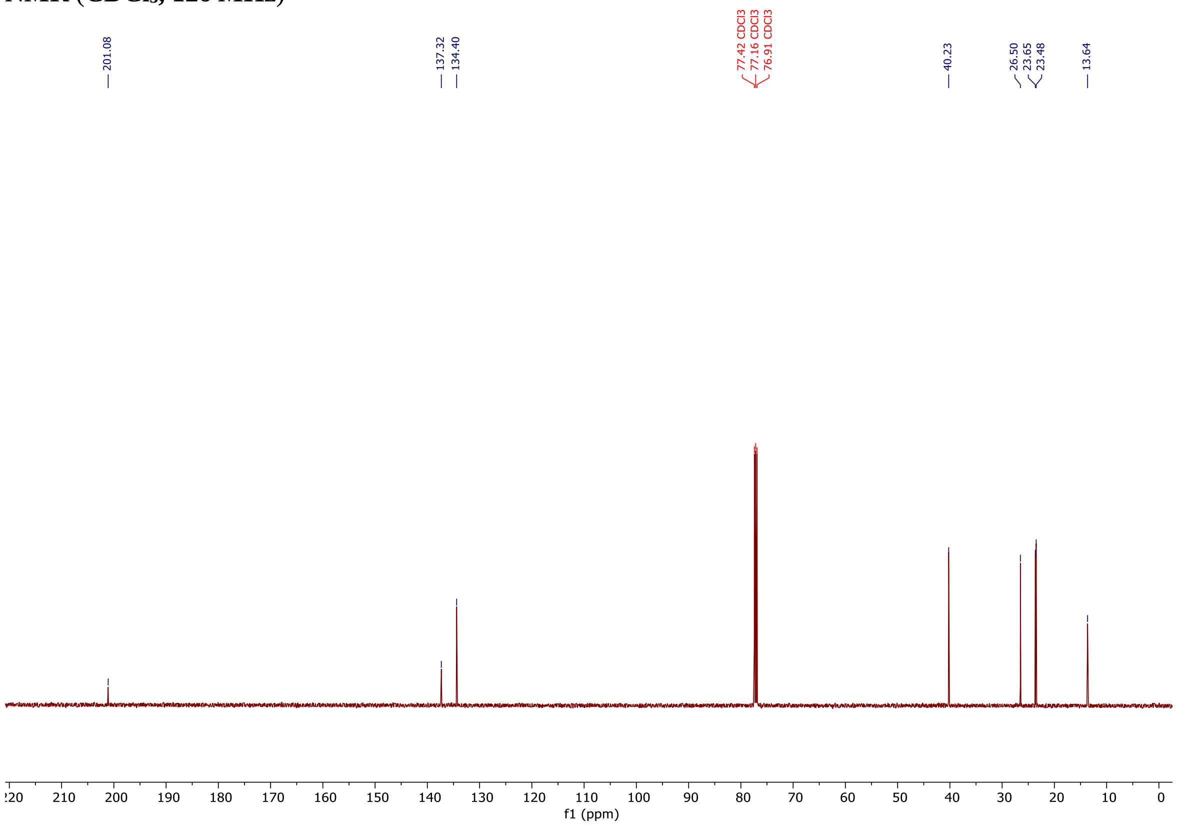

^{1}H NMR ($CDCl_3$, 501 MHz) of (5b)

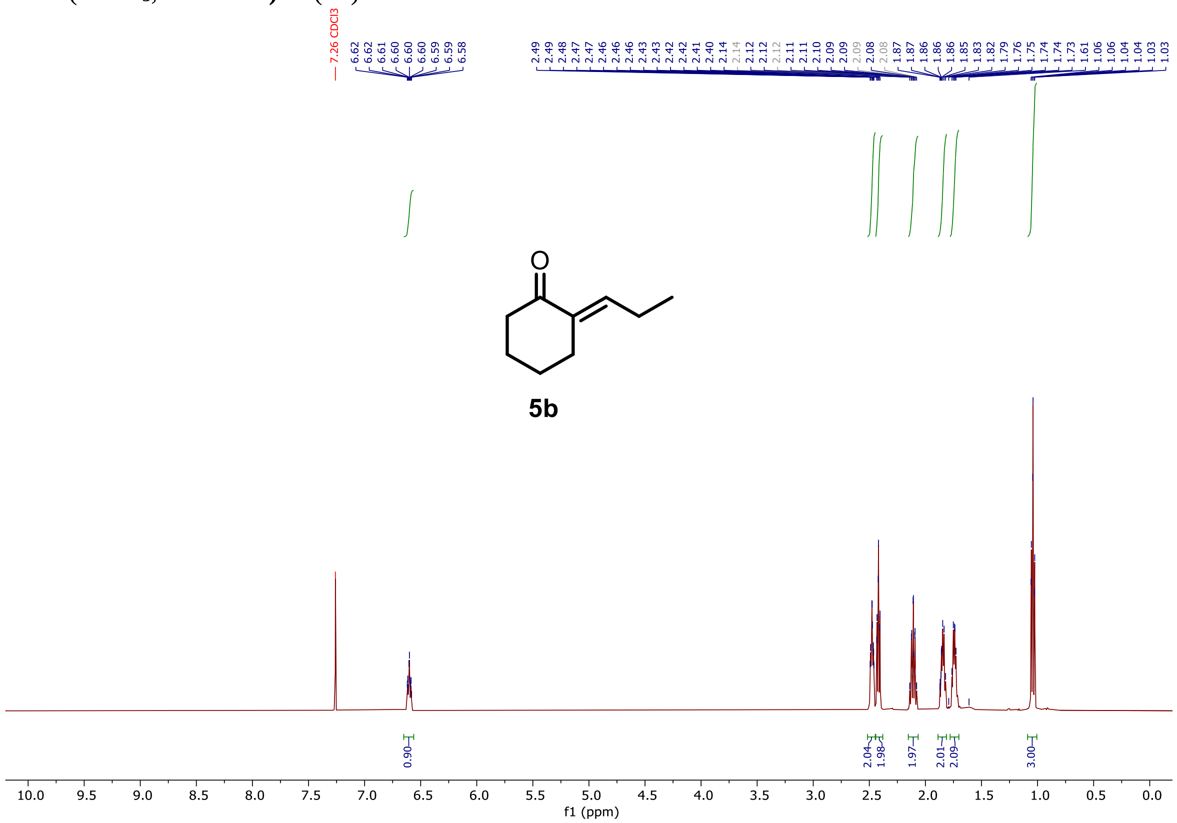

^{13}C NMR ($CDCl_3$, 126MHz)

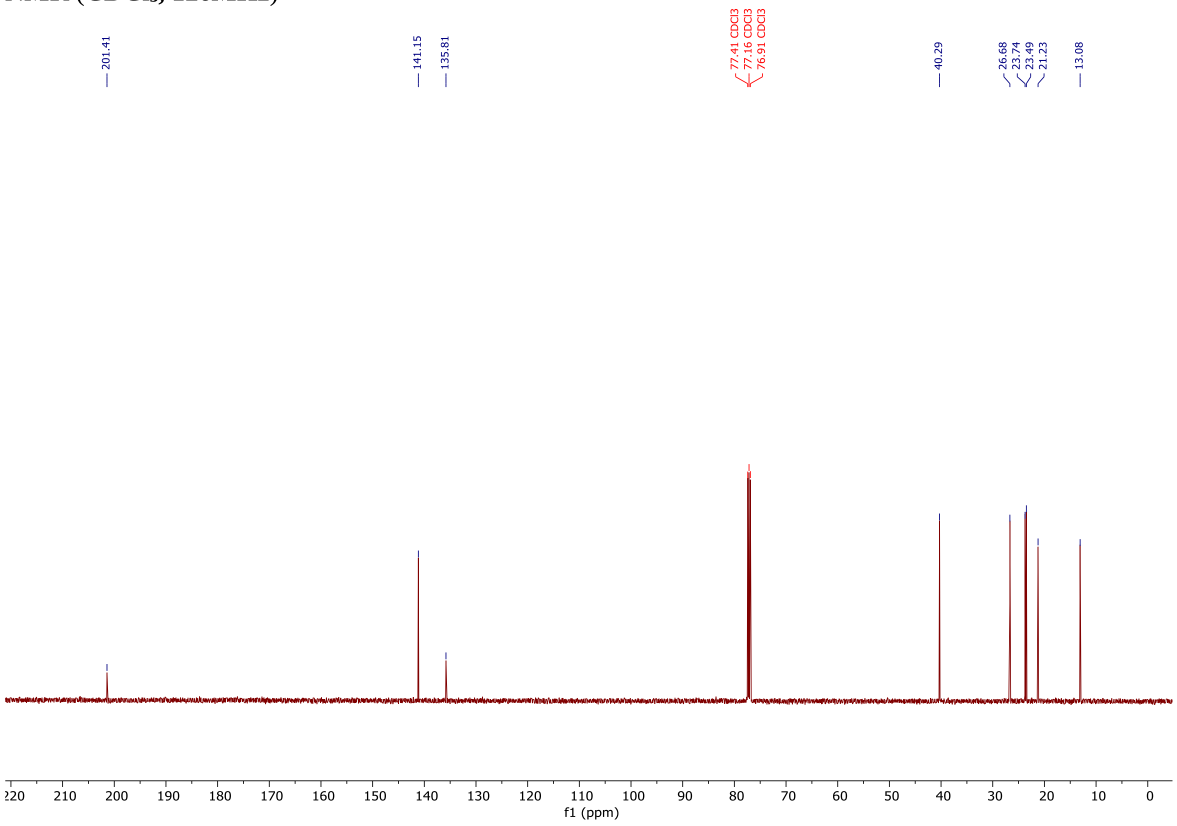

^{1}H NMR ($CDCl_3$, 501 MHz) of (5e)

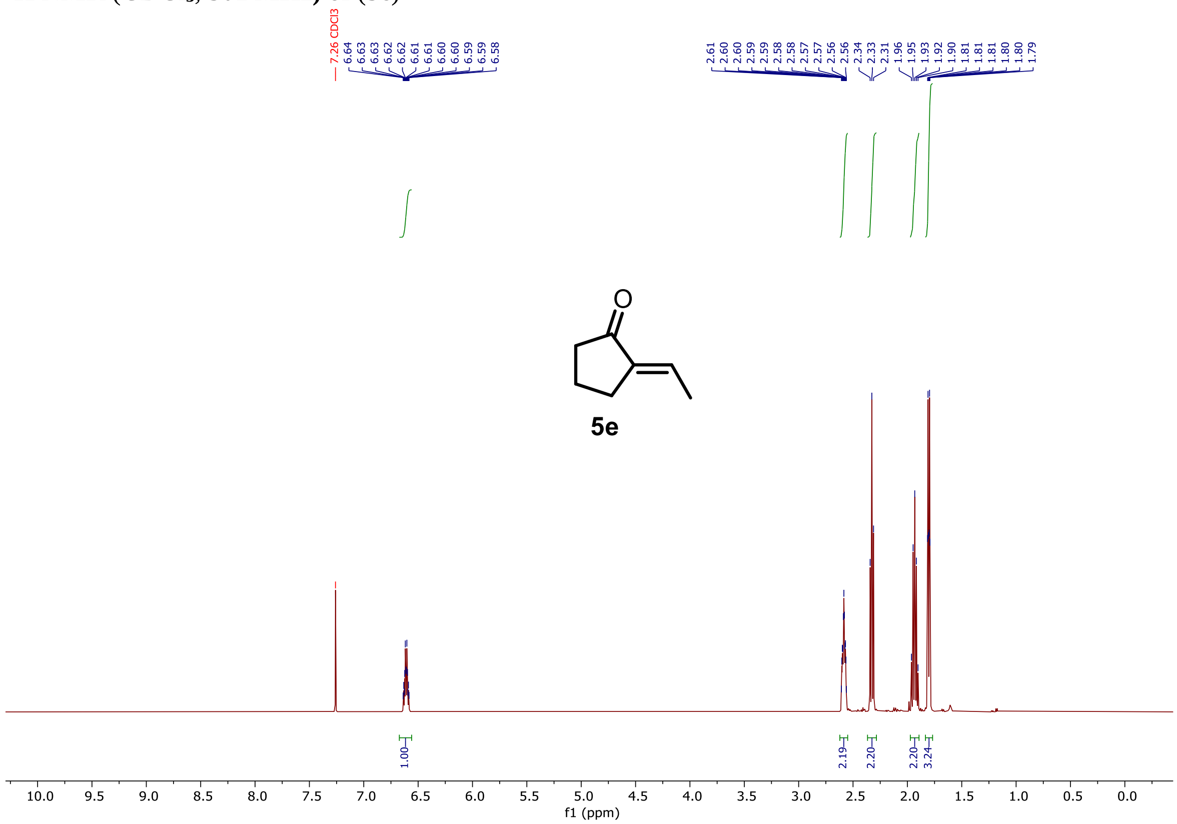

^{13}C NMR ($CDCl_3$, 126 MHz)

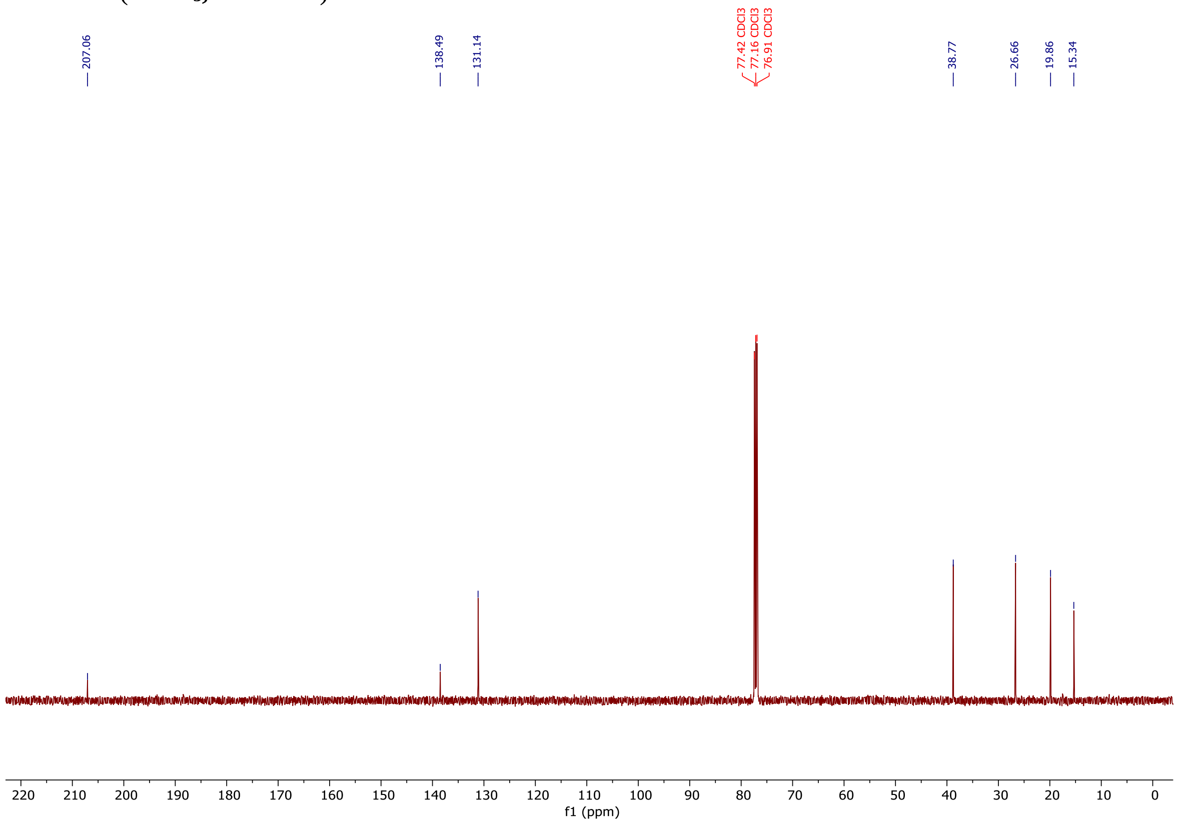

^{1}H NMR ($CDCl_3$, 501 MHz) of (5f)

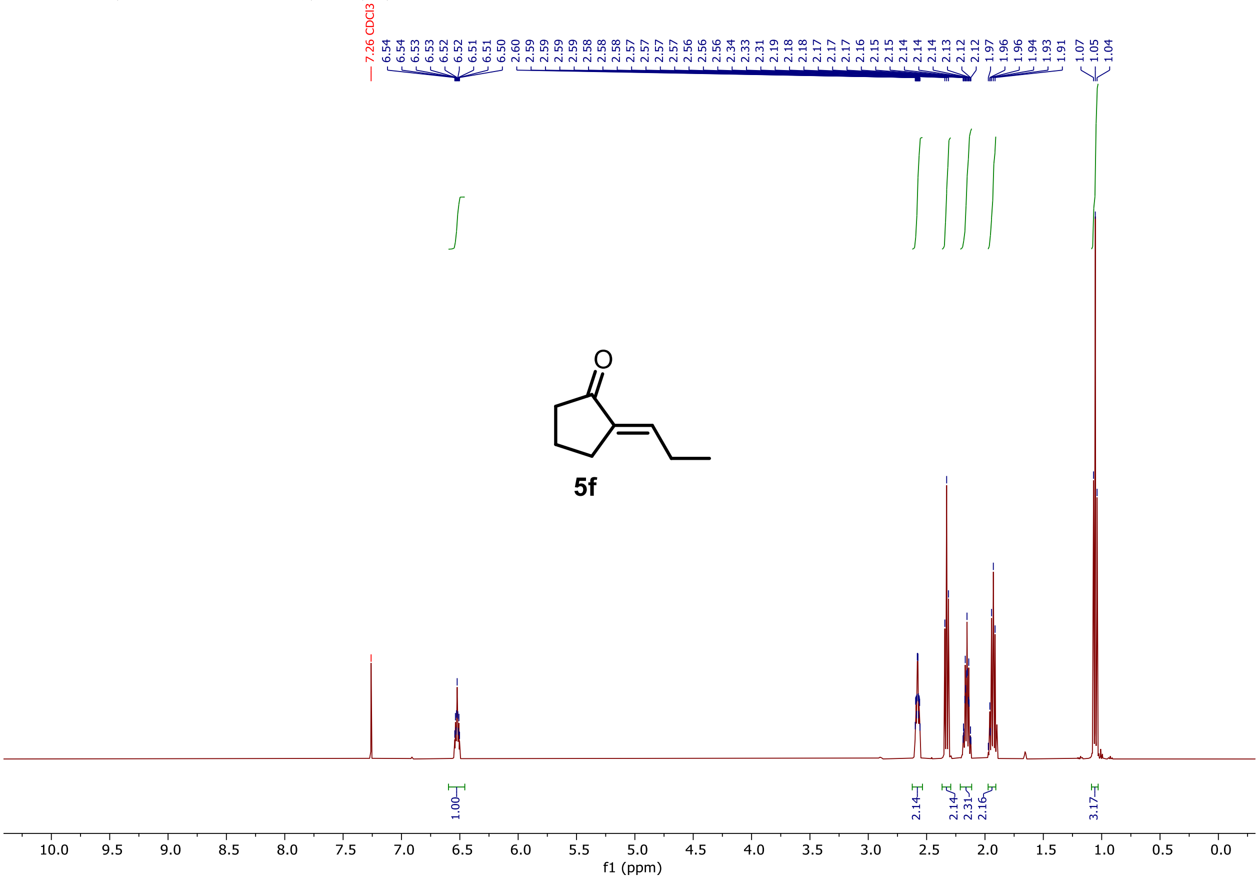

^{13}C NMR ($CDCl_3$, 126 MHz)

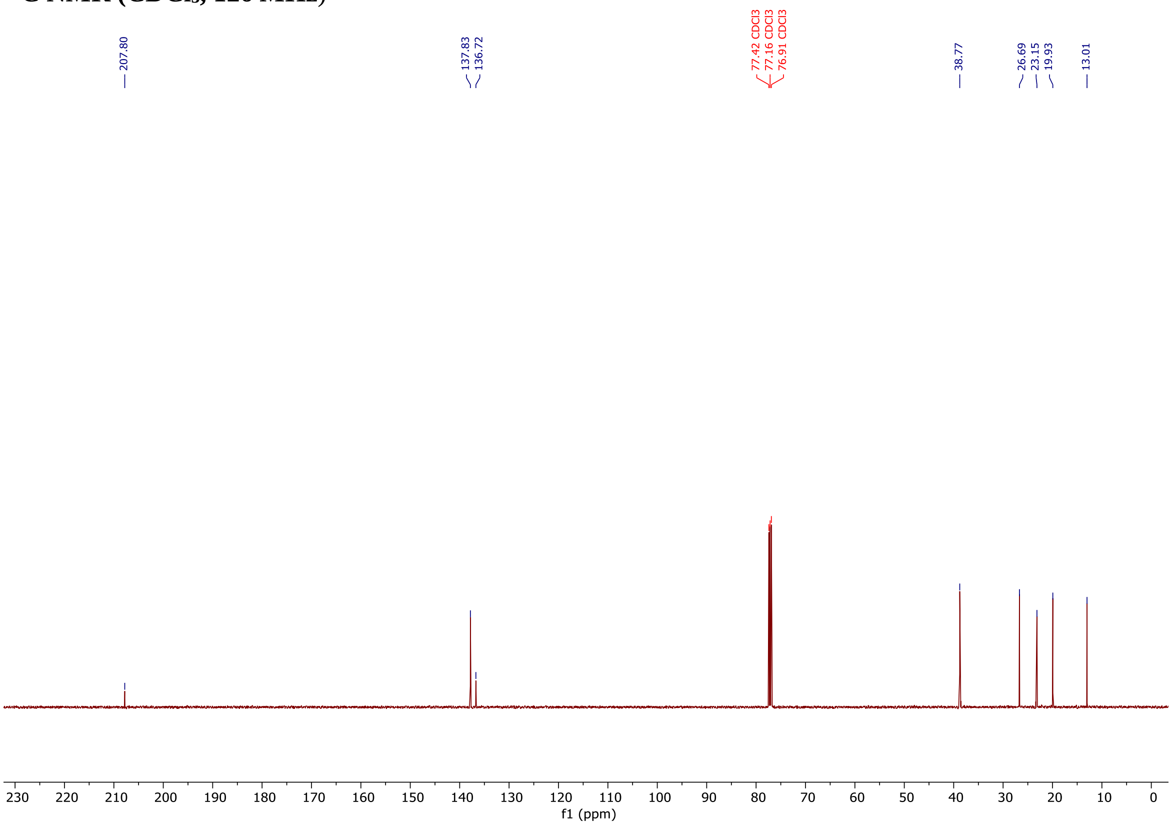

¹H NMR, (CD_2Cl_2, 501 MHz) of (5d)

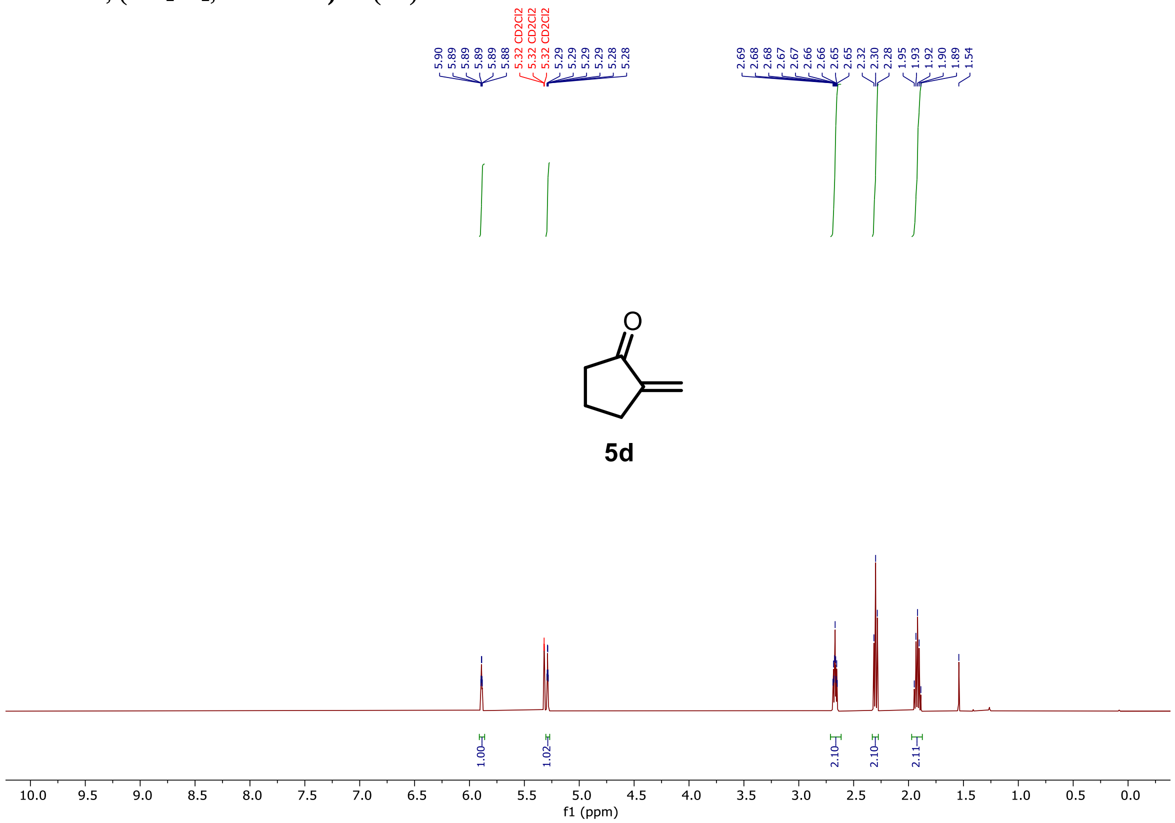

¹³C NMR (CD_2Cl_2, 126 MHz)

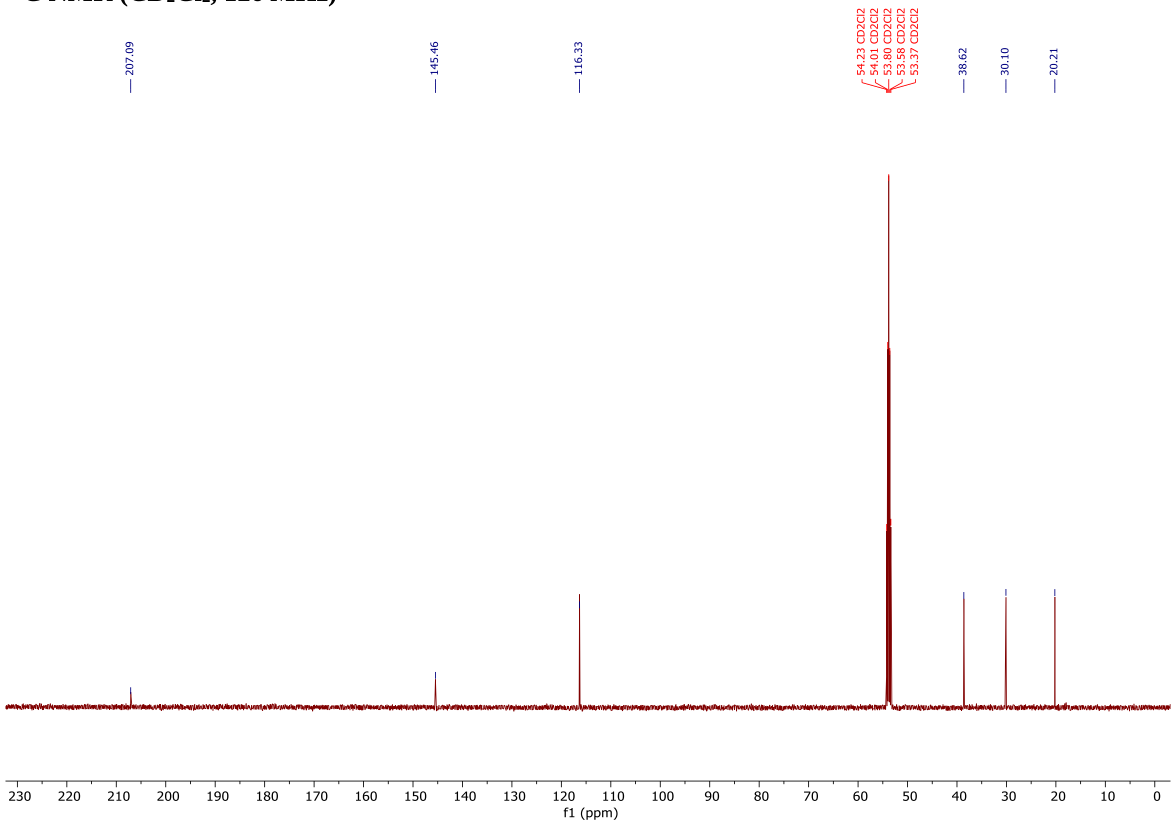

^{1}H NMR ($CDCl_3$, 501 MHz) of (5n)

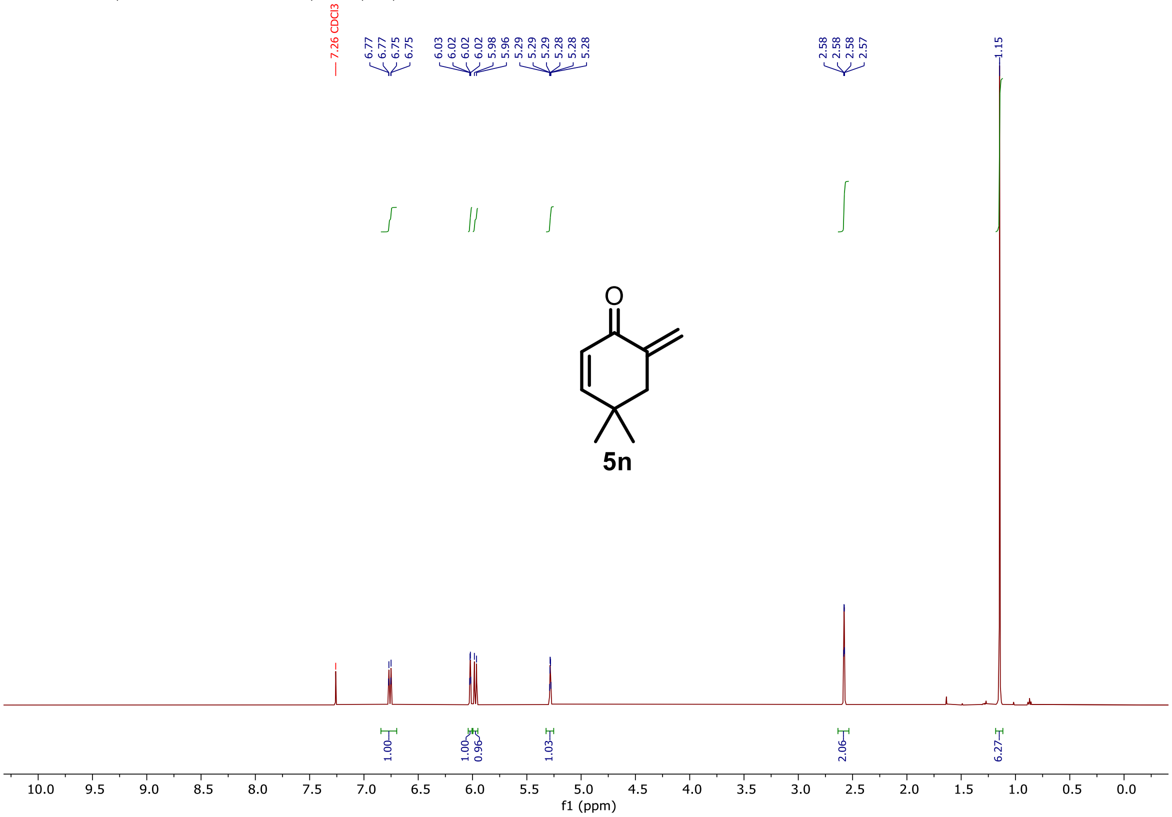

^{13}C NMR ($CDCl_3$, 126 MHz)

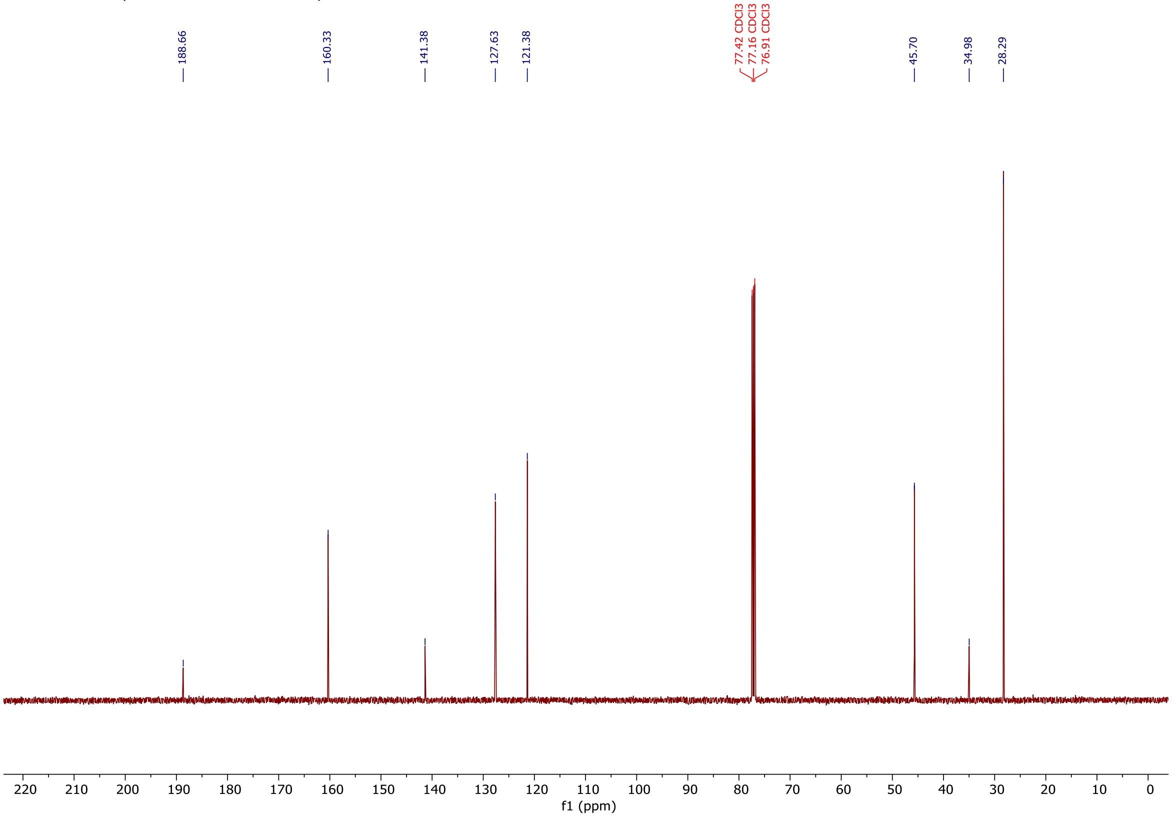

^{1}H NMR ($CDCl_3$, 501 MHz) of (5o)

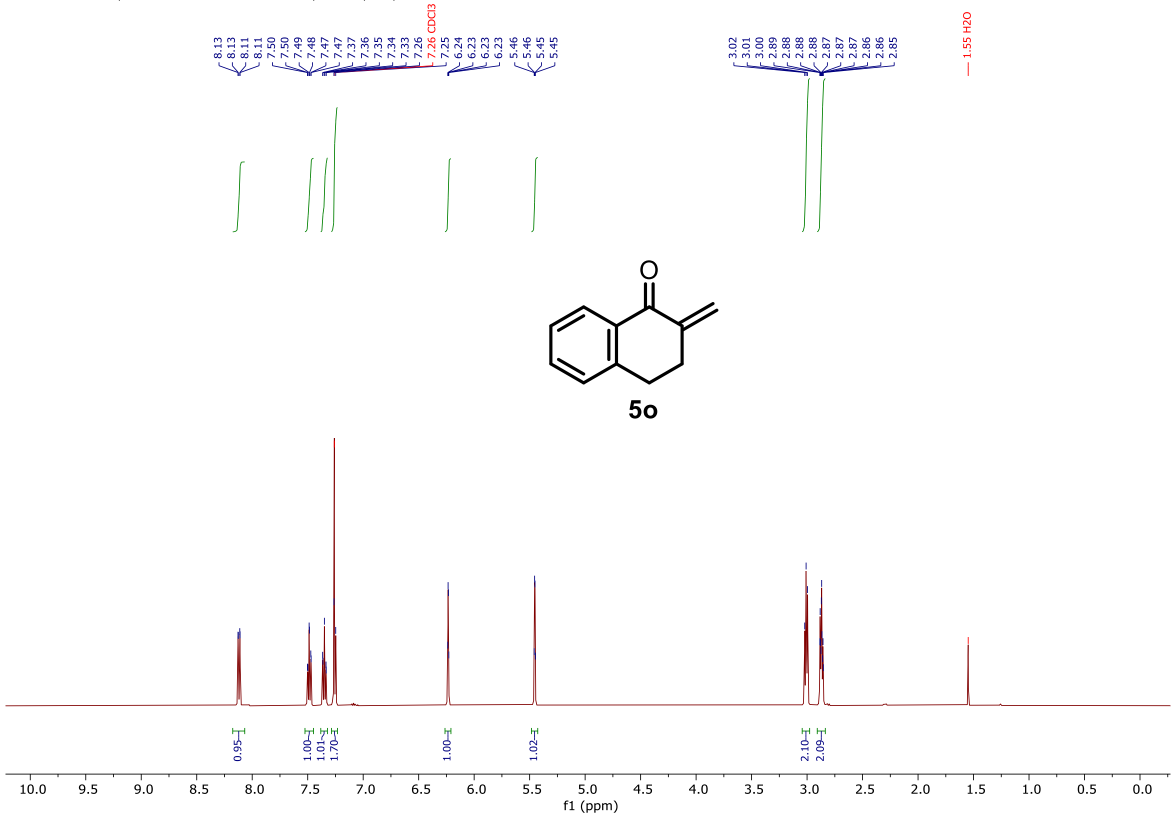

^{13}C NMR ($CDCl_3$, 126 MHz)

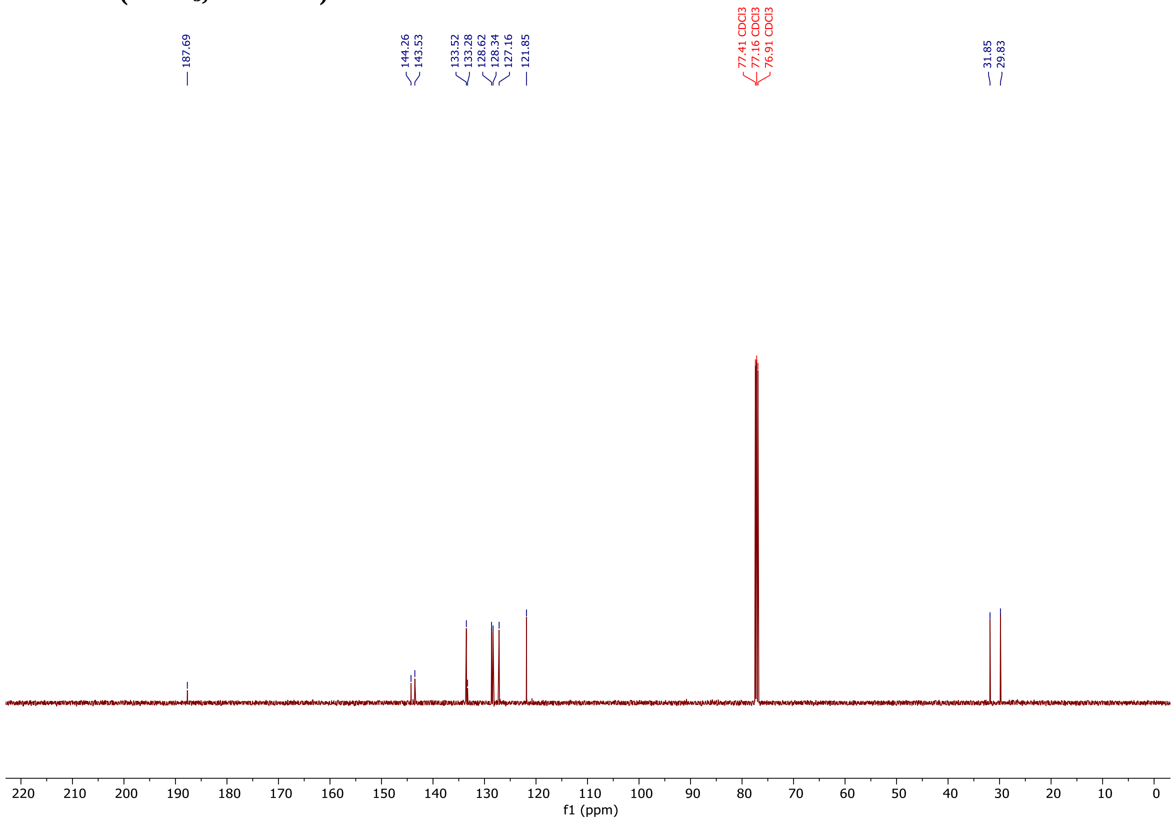

^{1}H NMR ($CDCl_3$, 501 MHz) of (5k)

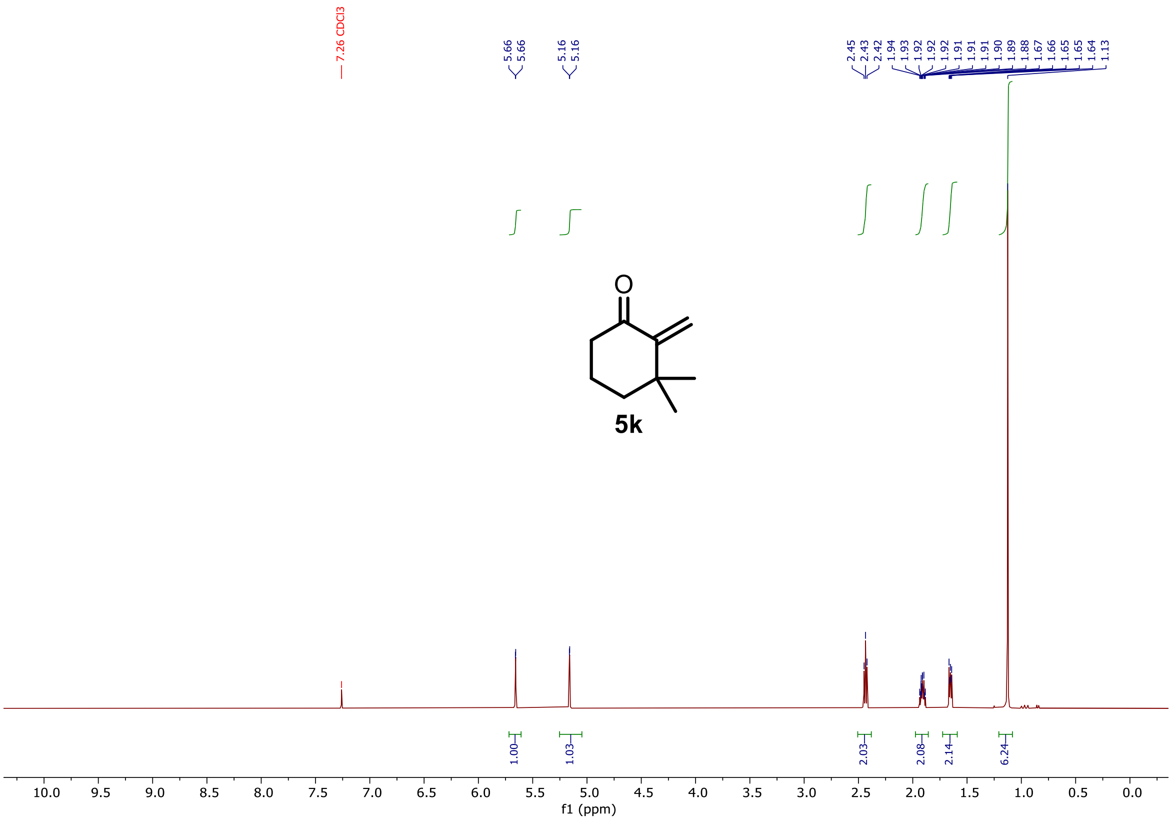

^{13}C NMR ($CDCl_3$, 126 MHz)

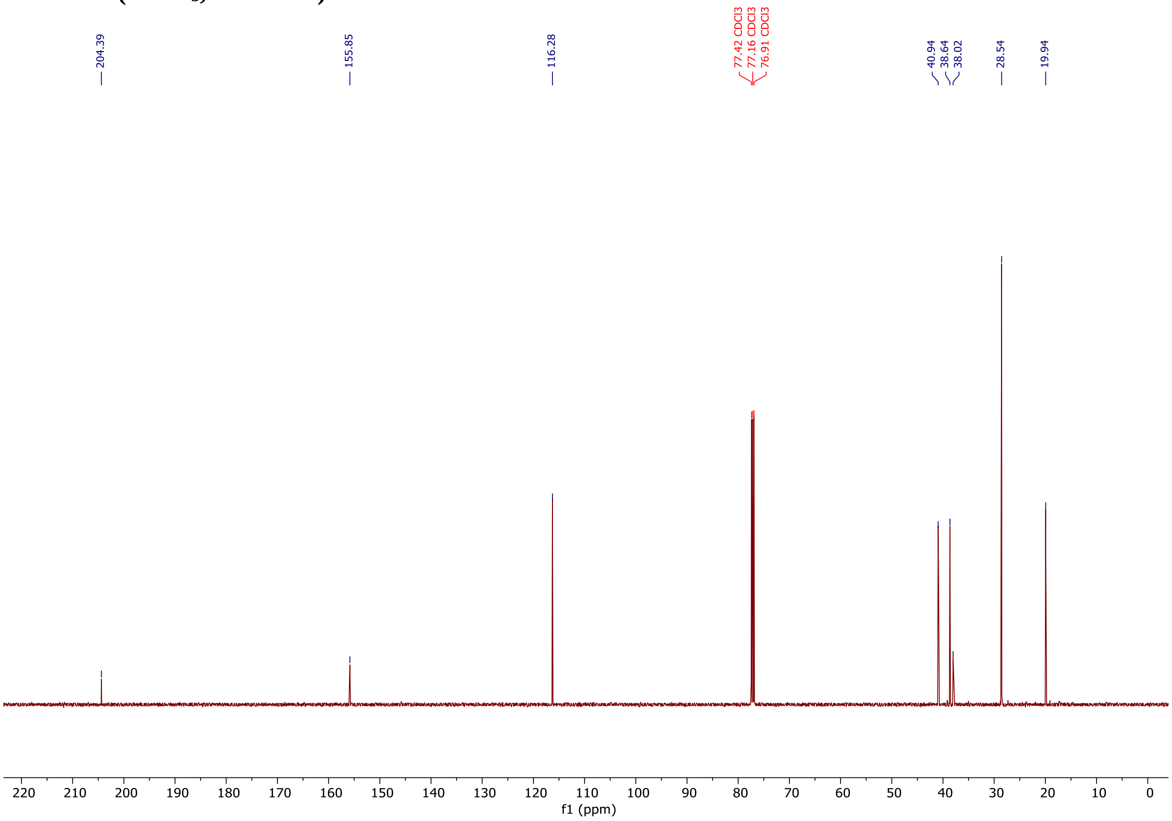

^{1}H NMR ($CDCl_3$, 501 MHz) of (12)

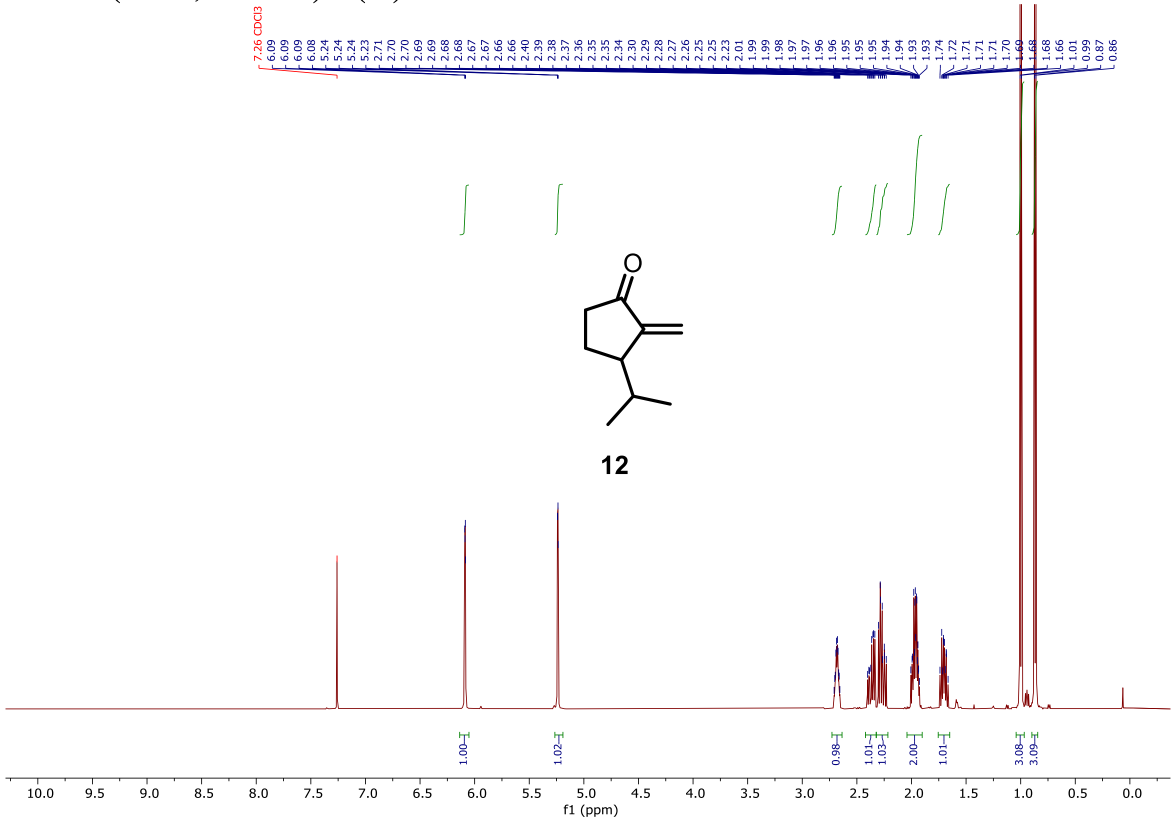

^{13}C NMR ($CDCl_3$, 126 MHz)

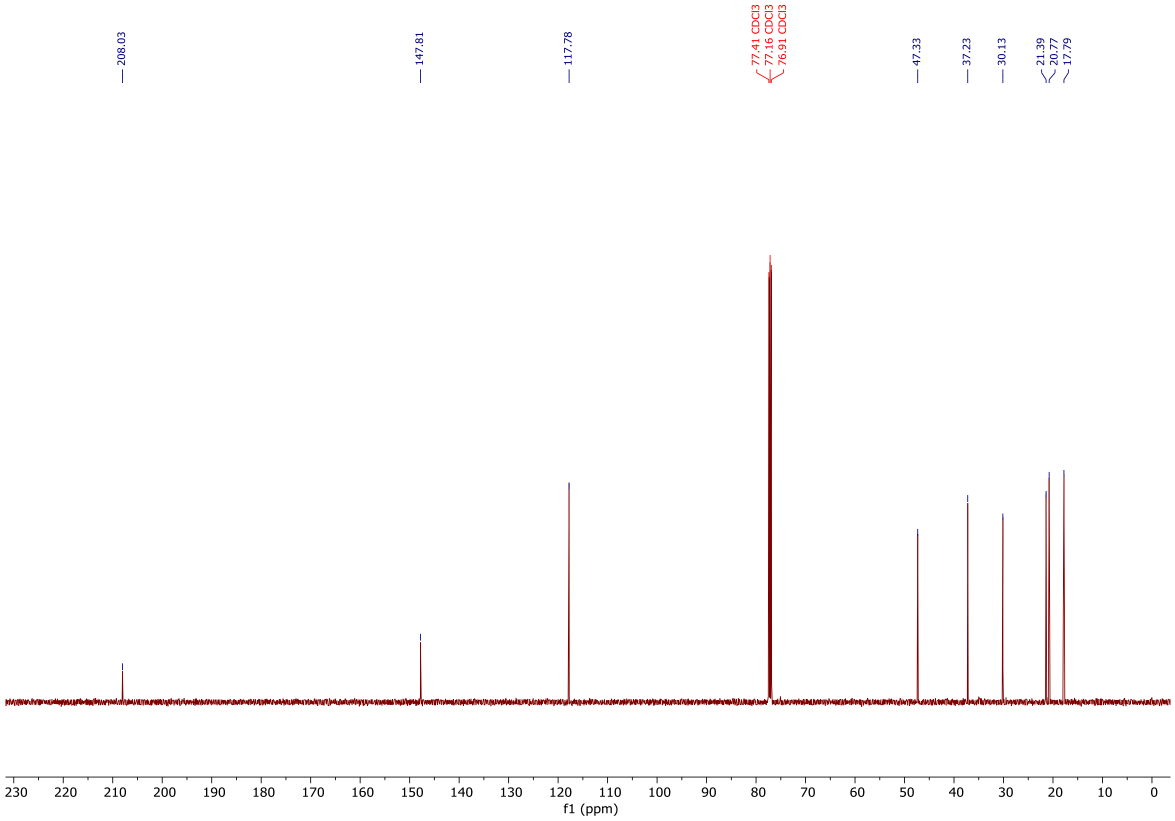

^{1}H NMR ($CDCl_3$, 500 MHz) of (23b)

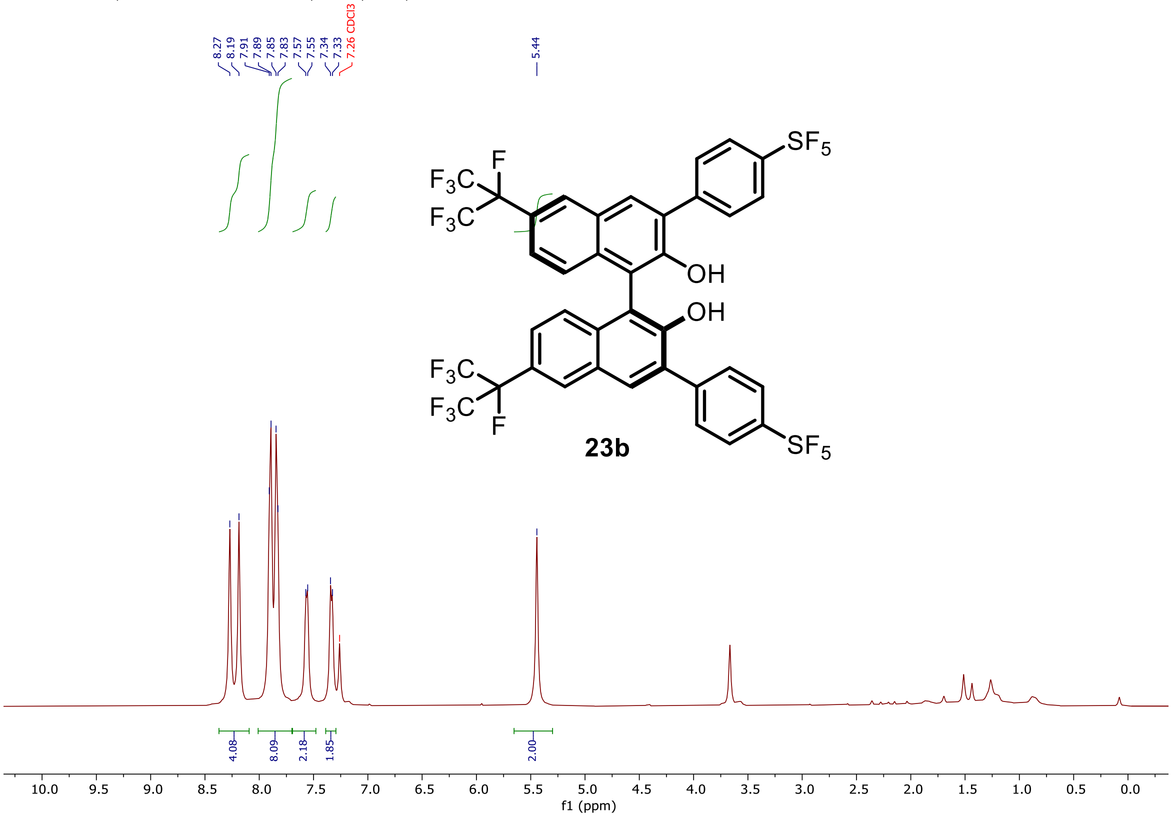

^{13}C NMR ($CDCl_3$, 126 MHz)

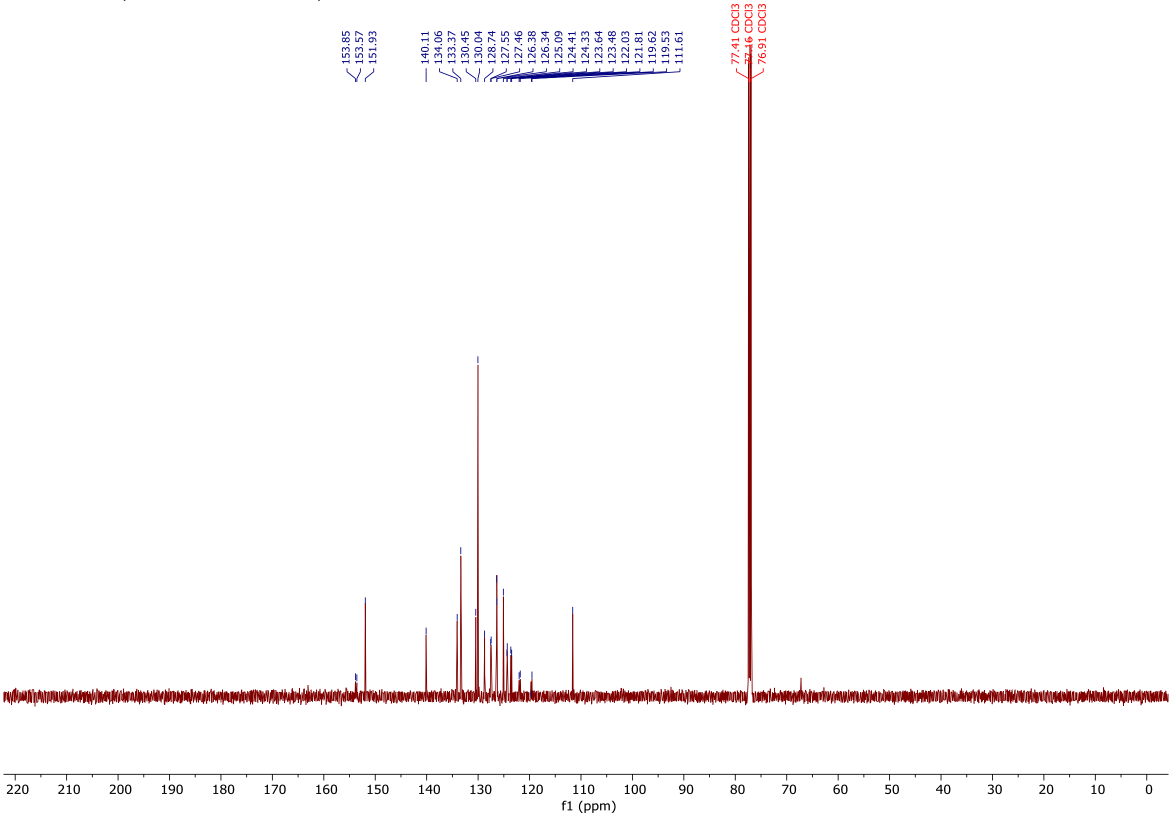

^{19}F NMR ($CDCl_3$, 471 MHz)

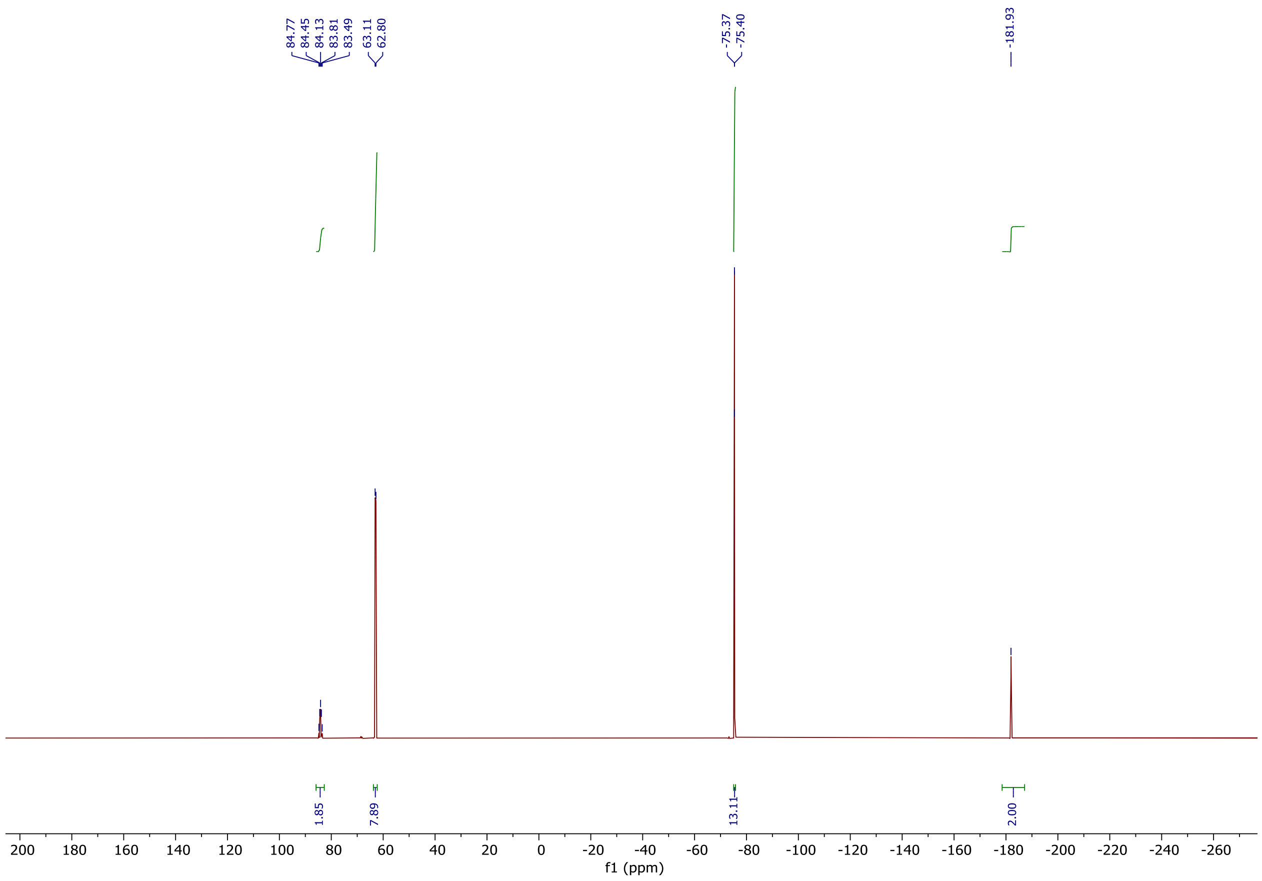

^{1}H NMR ($CDCl_3$, 501 MHz) of (11b)

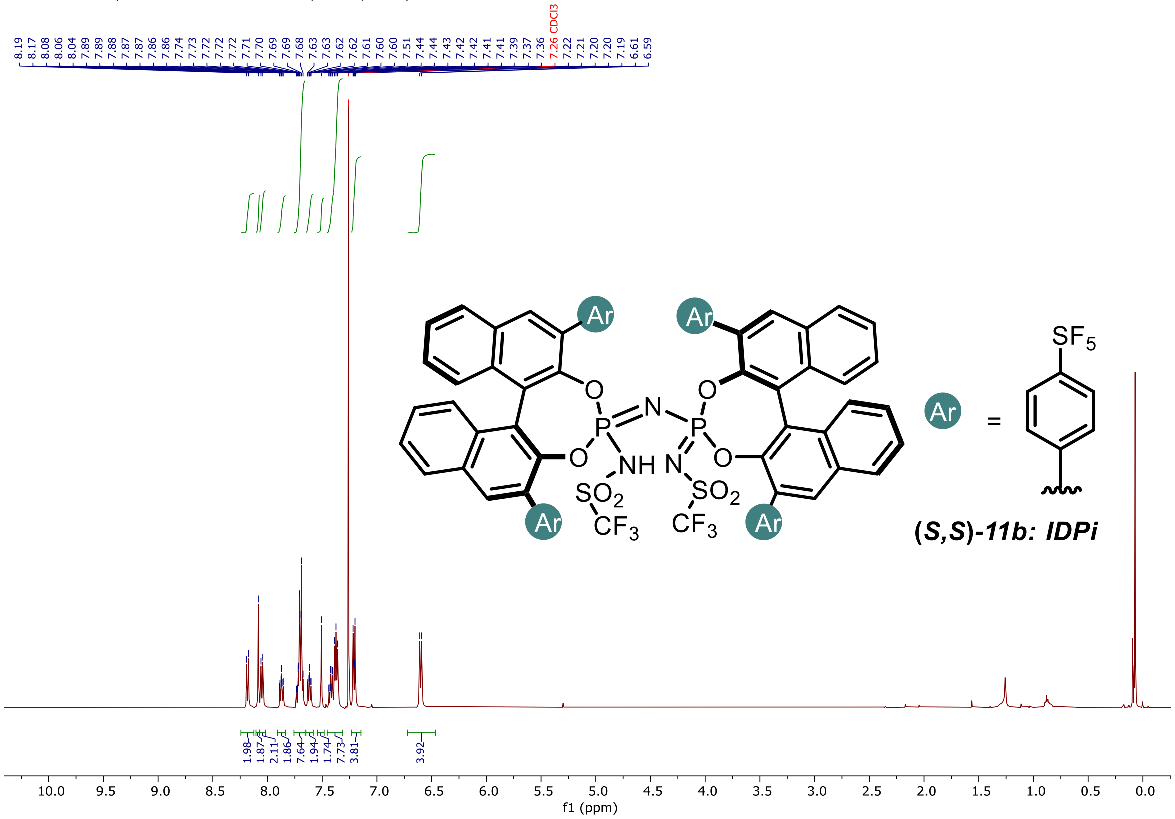

^{13}C NMR ($CDCl_3$, 151 MHz)

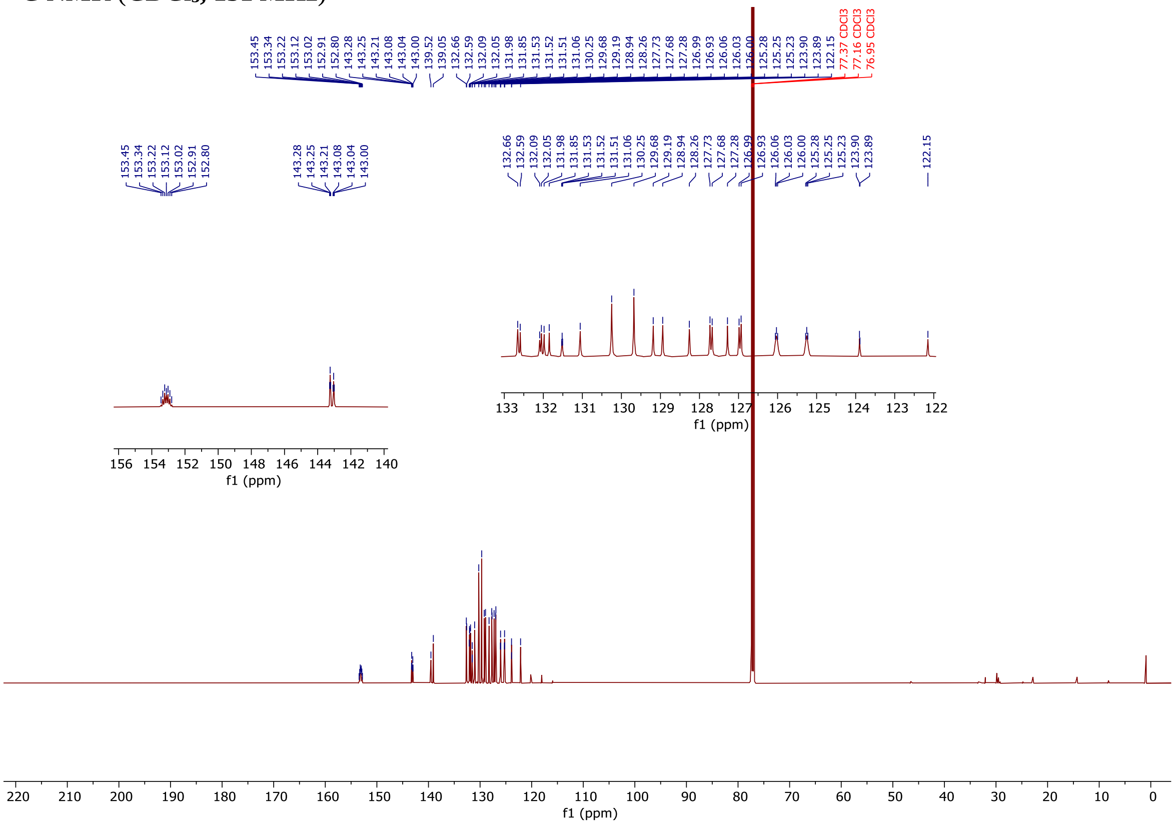

^{19}F NMR ($CDCl_3$, 471 MHz)

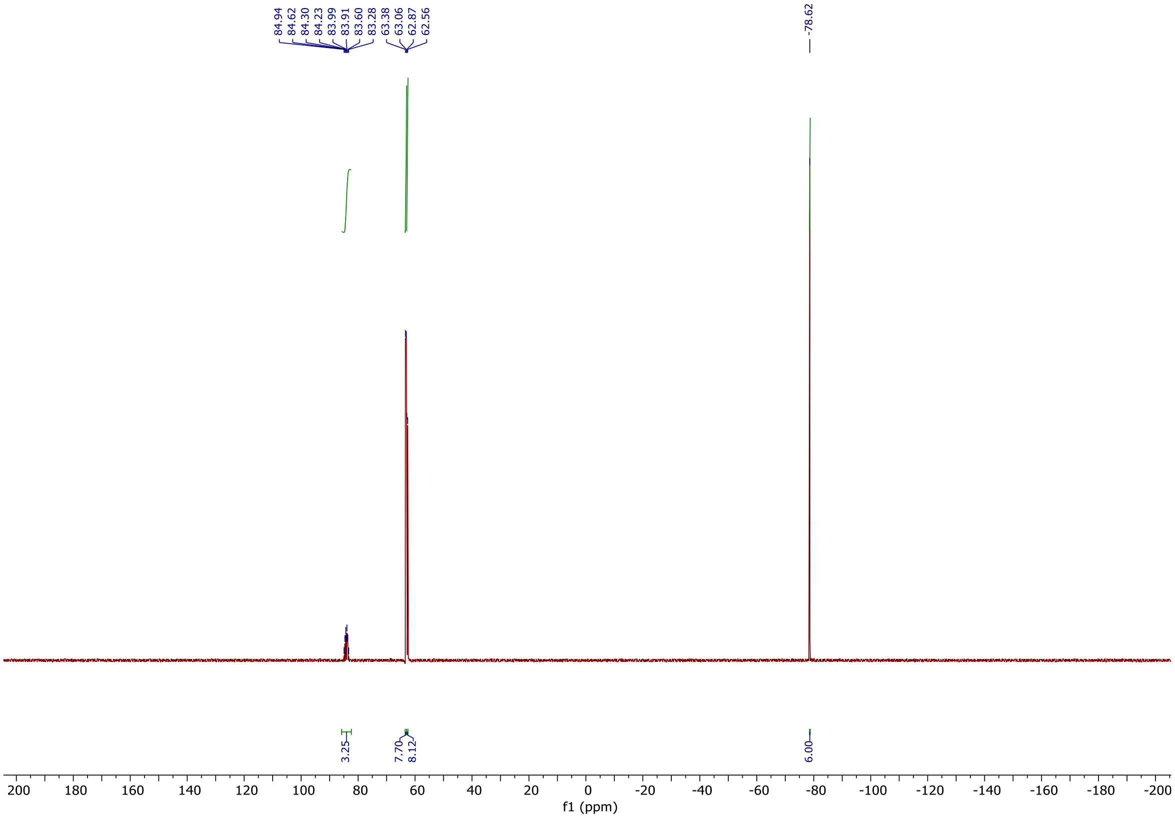

^{31}P NMR ($CDCl_3$, 203 MHz)

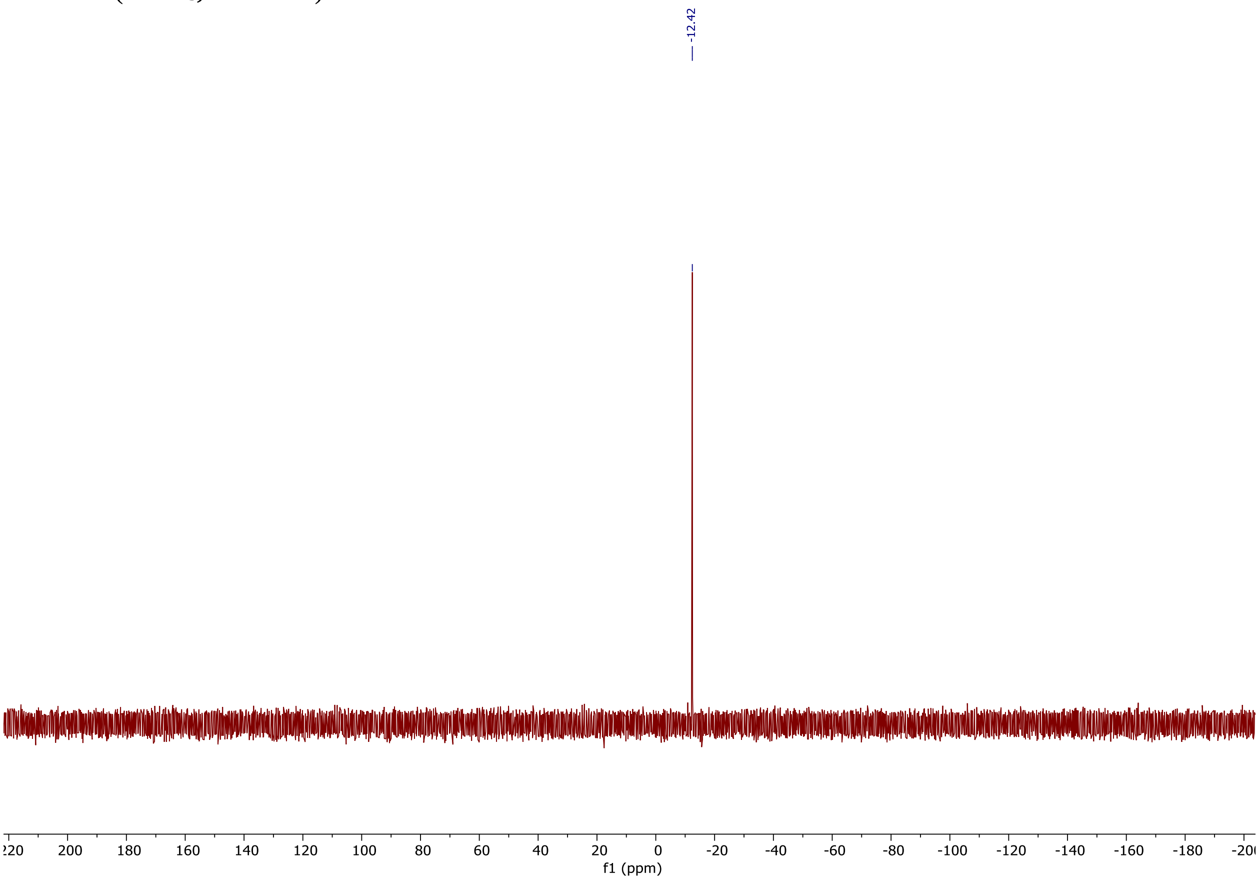

^{1}H NMR ($CDCl_3$, 501 MHz) of (11e)

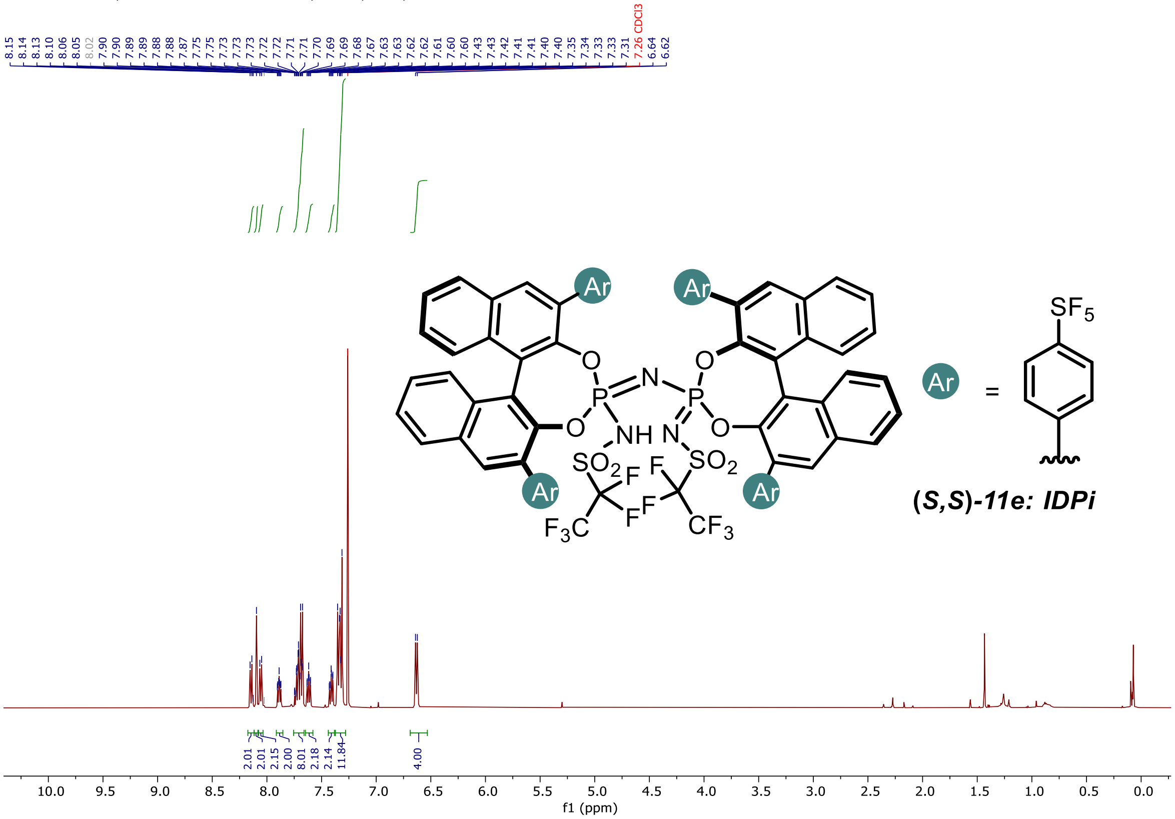

^{13}C NMR ($CDCl_3$, 151 MHz)

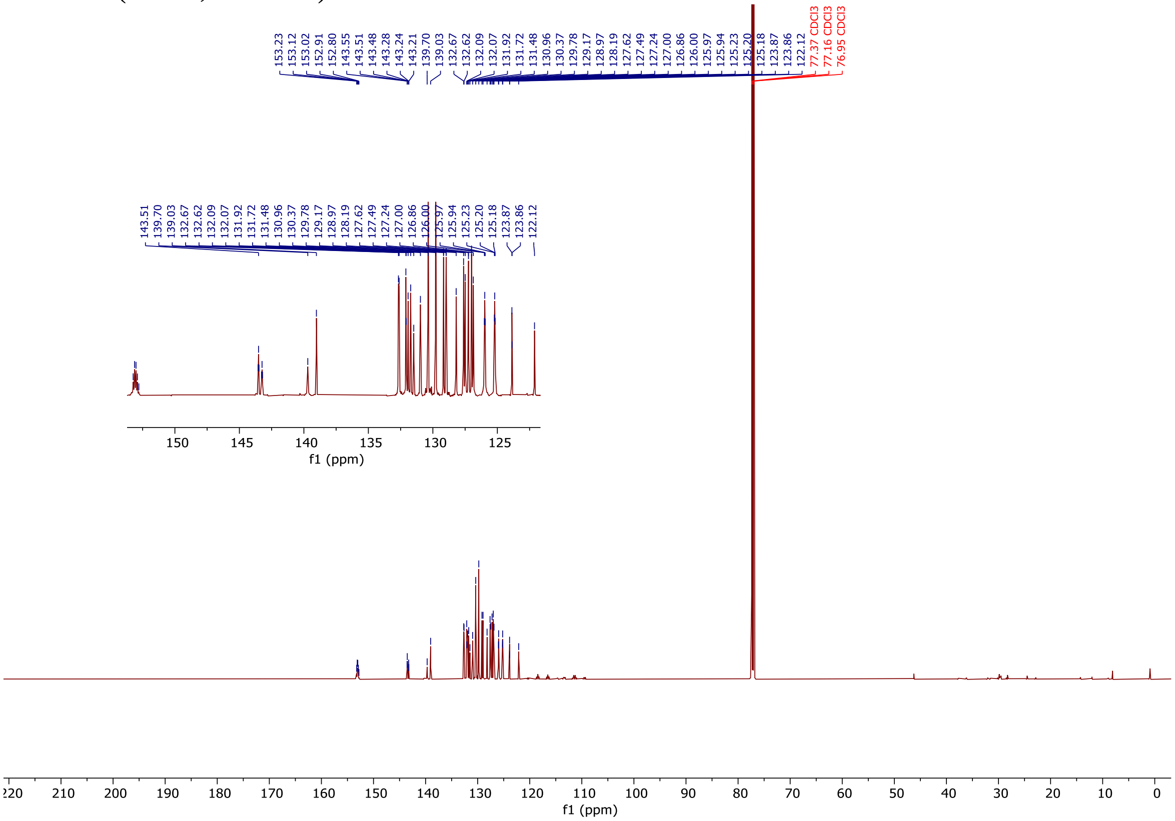

^{19}F NMR ($CDCl_3$, 471 MHz)

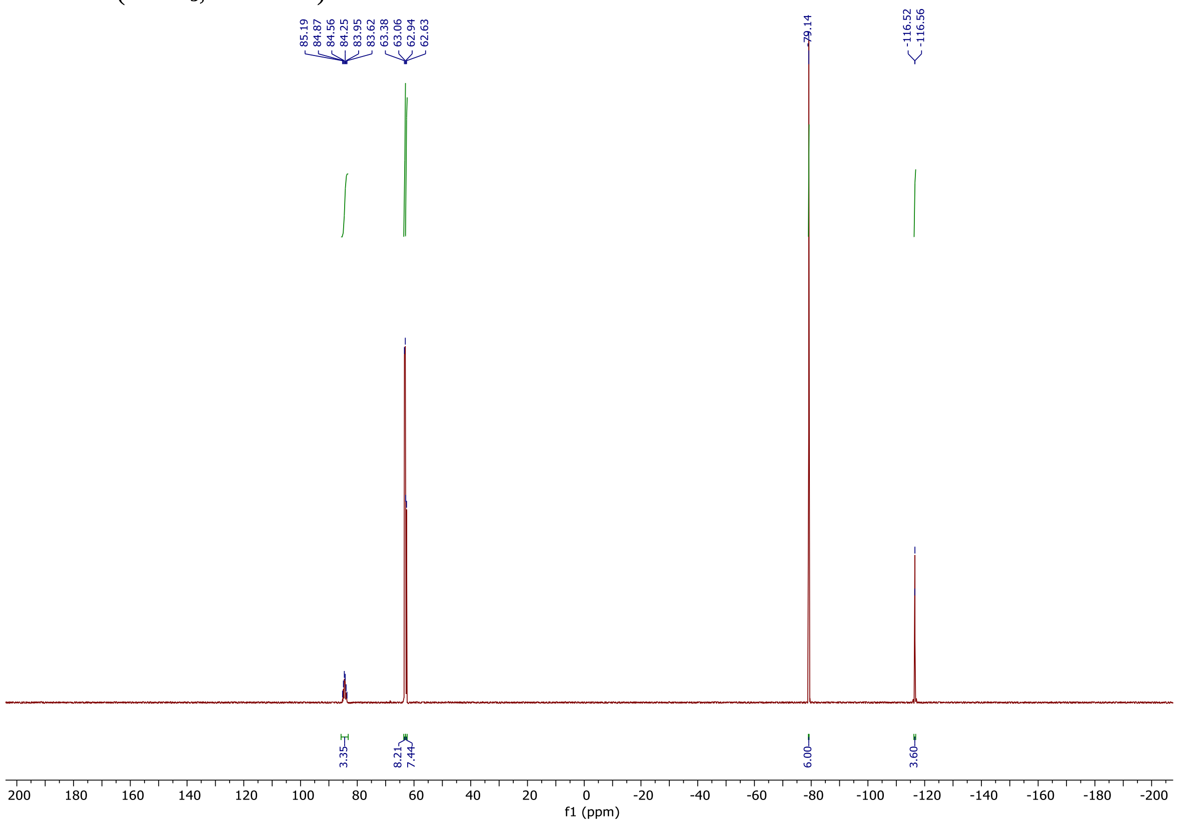

^{31}P NMR ($CDCl_3$, 203 MHz)

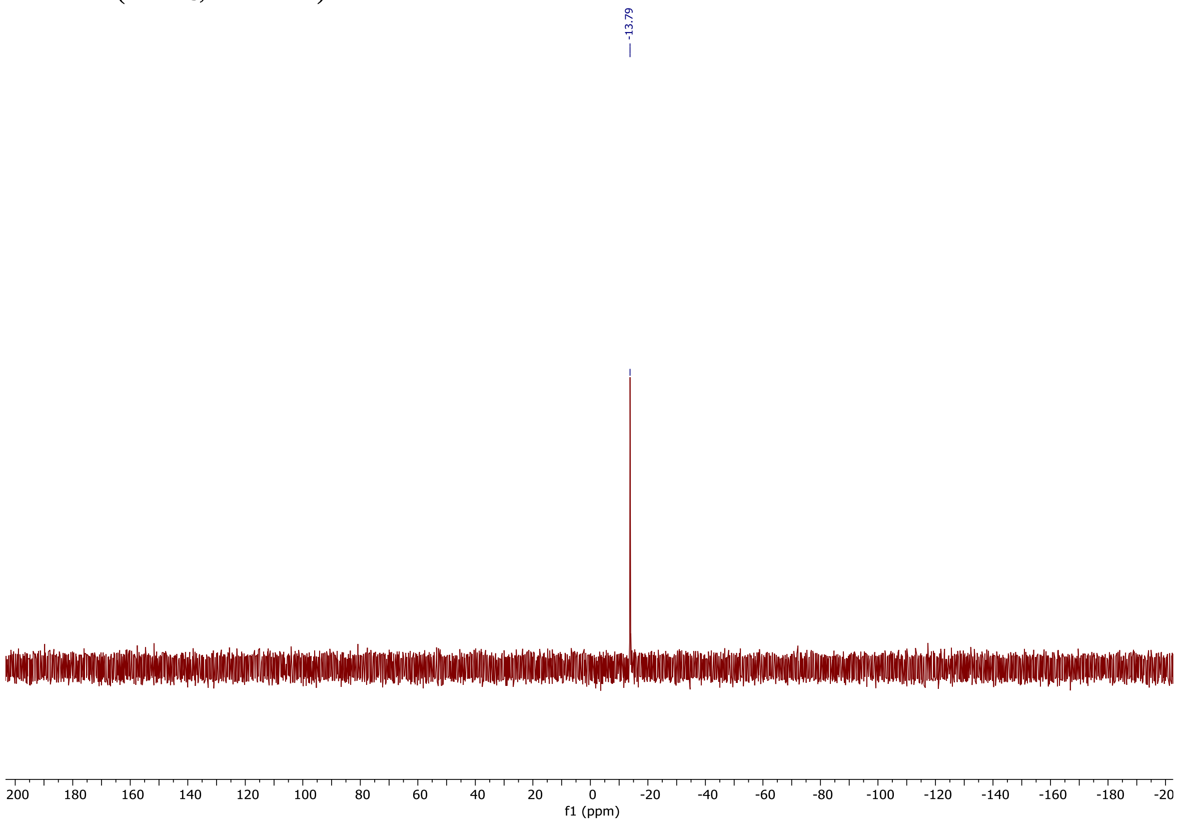

^{1}H NMR ($CDCl_3$, 501 MHz) of (11f)

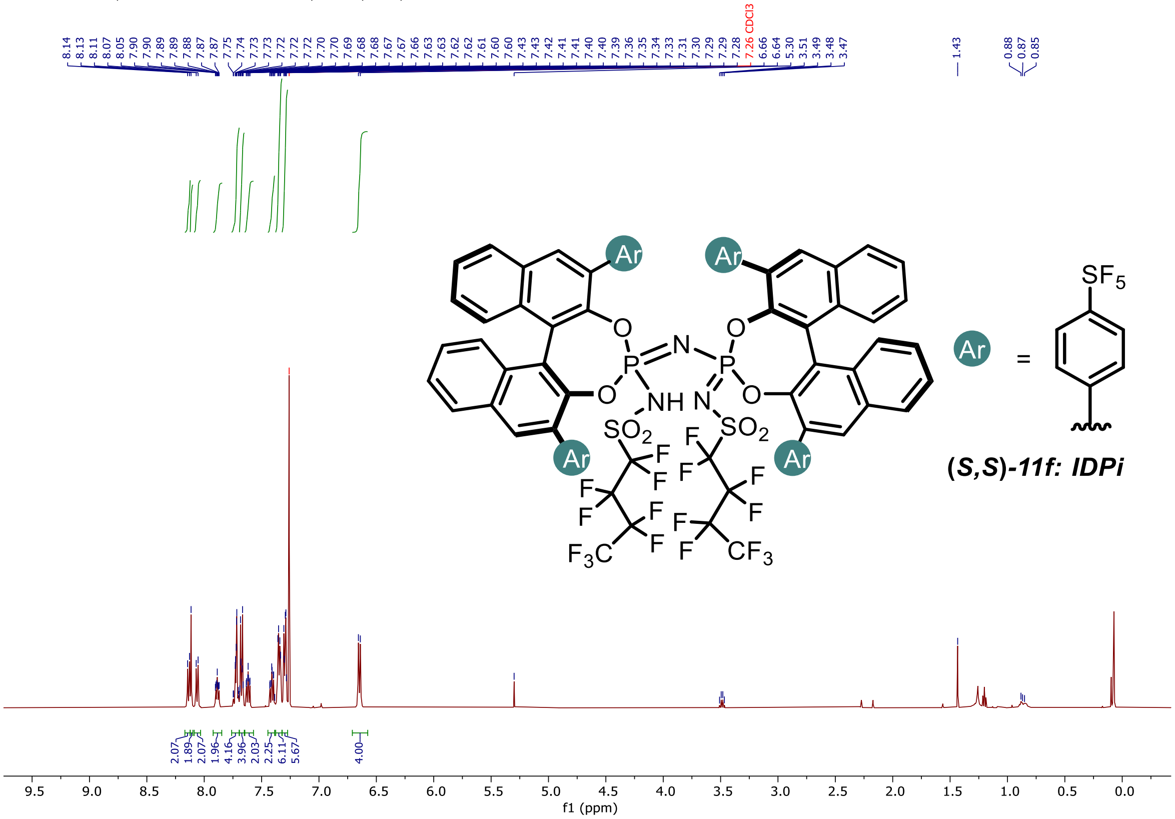

^{13}C NMR ($CDCl_3$, 151 MHz)

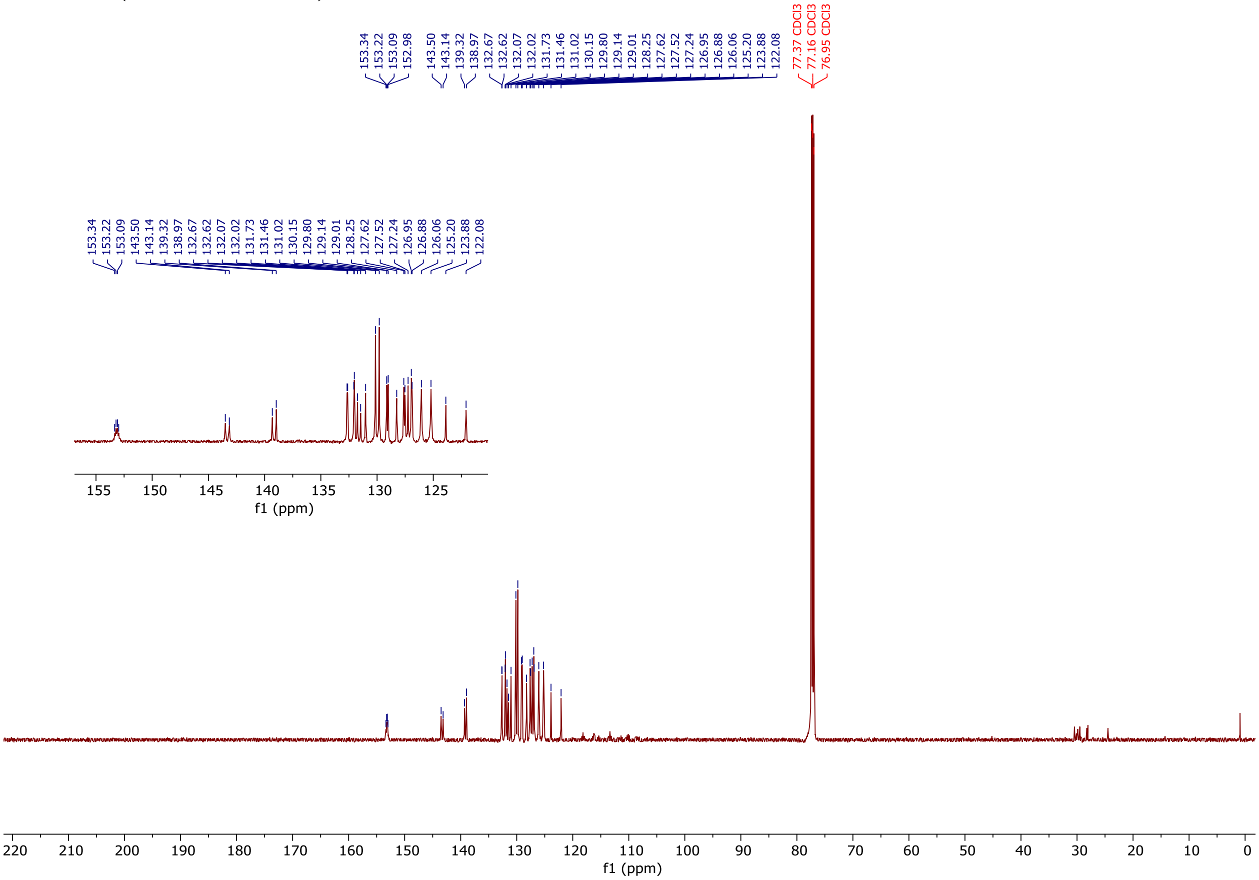

^{19}F NMR ($CDCl_3$, 471 MHz)

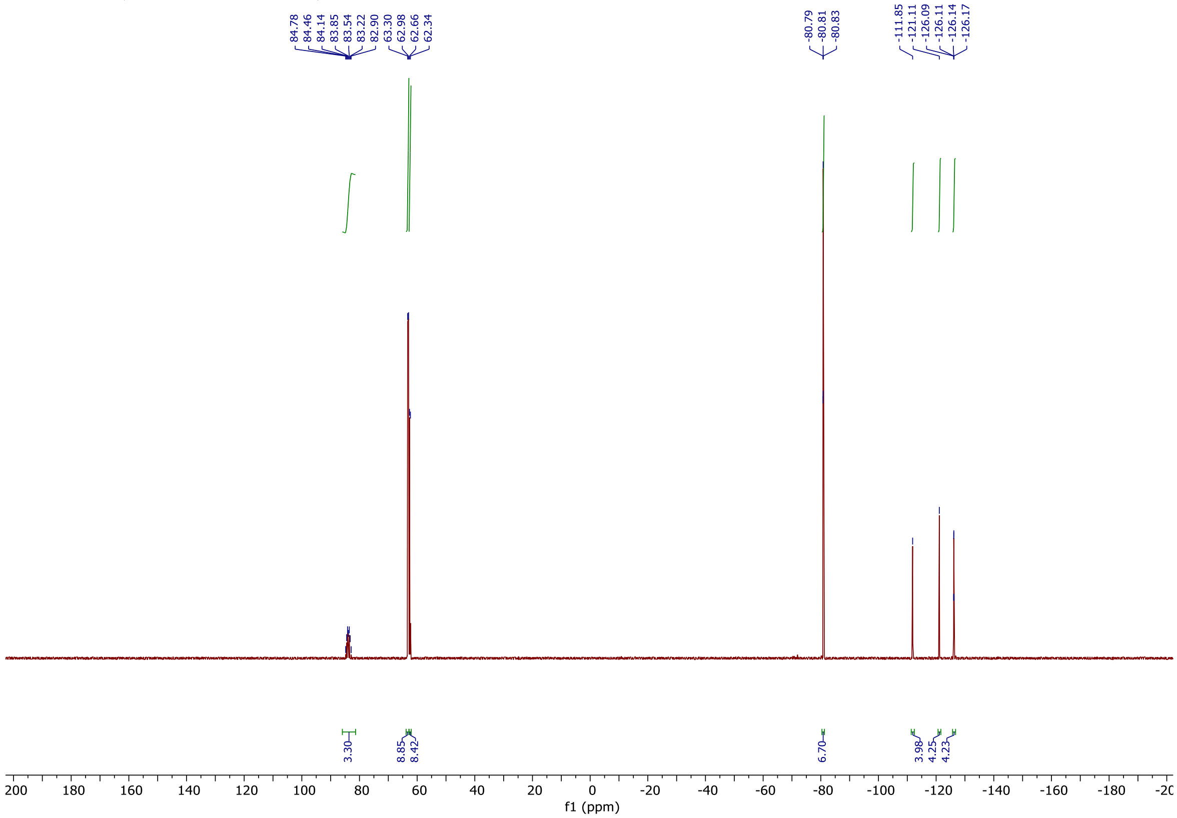

^{31}P NMR ($CDCl_3$, 203 MHz)

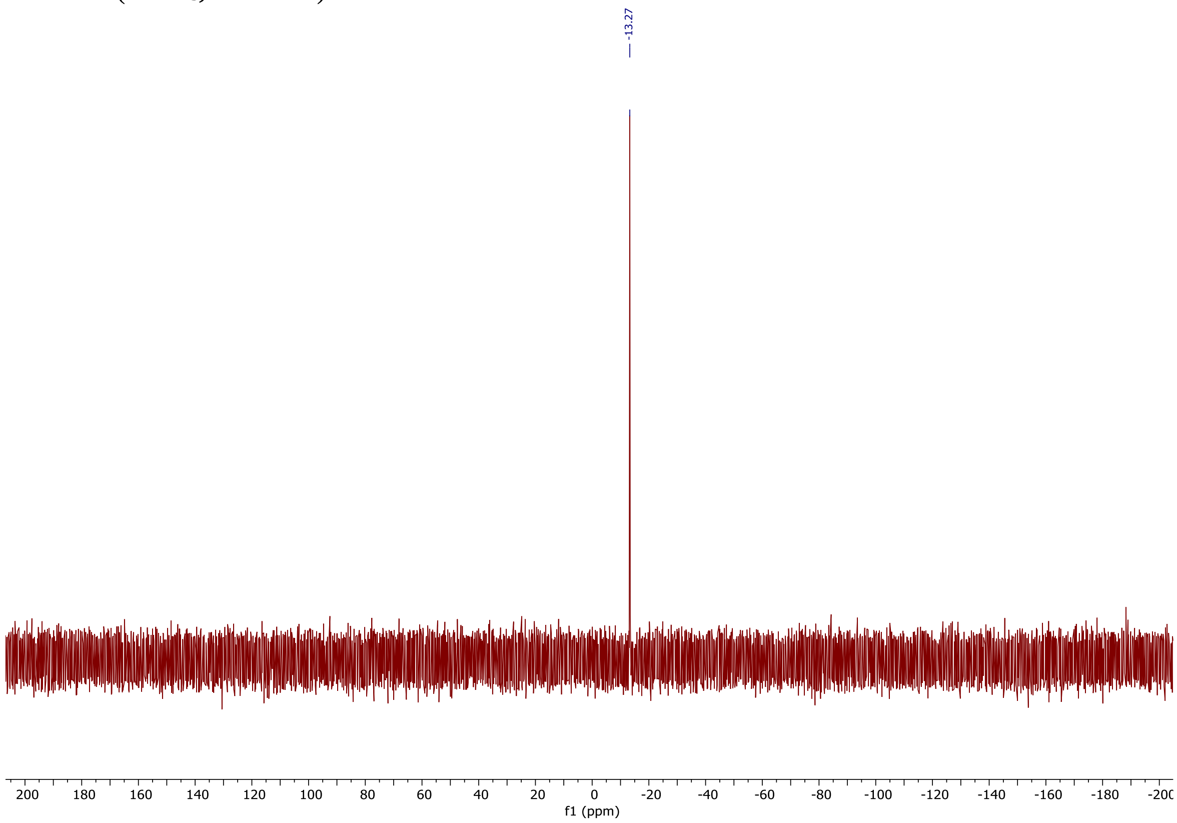

^{1}H NMR ($CDCl_3$, 600 MHz) of (11g)

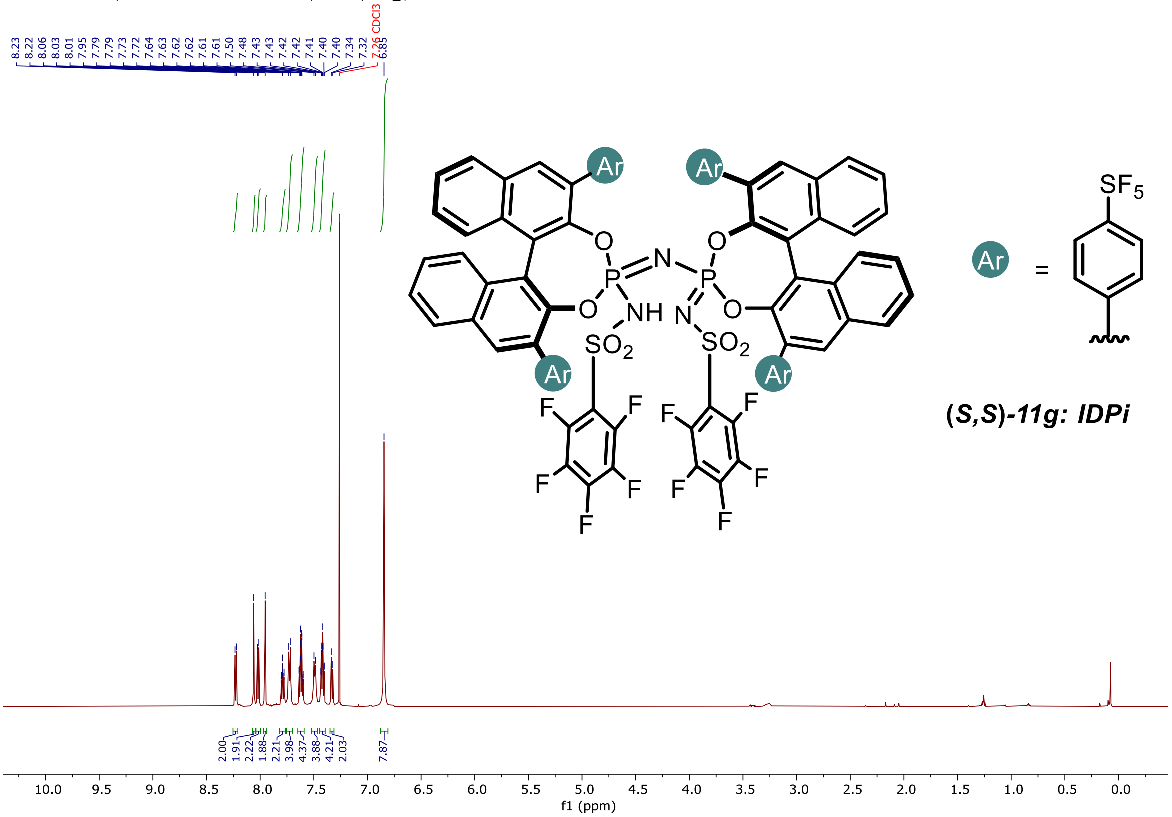

^{13}C NMR ($CDCl_3$, 151 MHz)

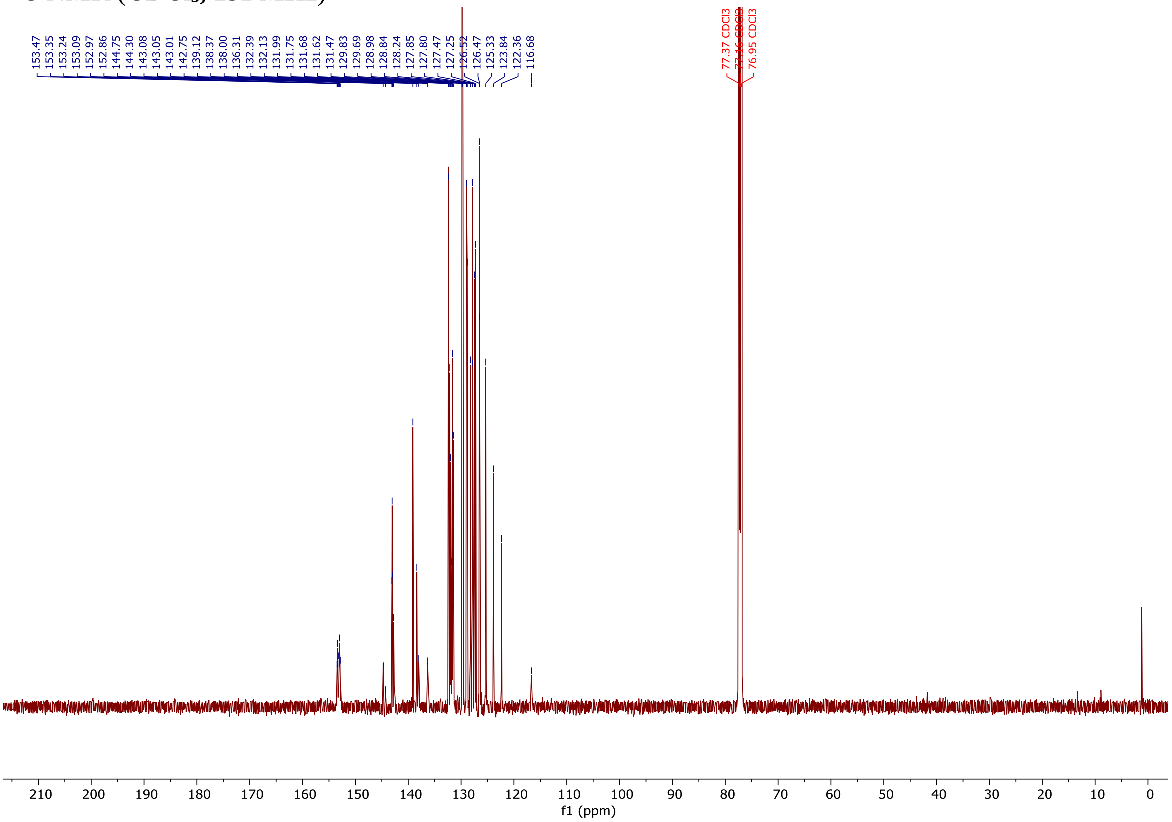

^{19}F NMR CDCl$_3$, 565 MHz

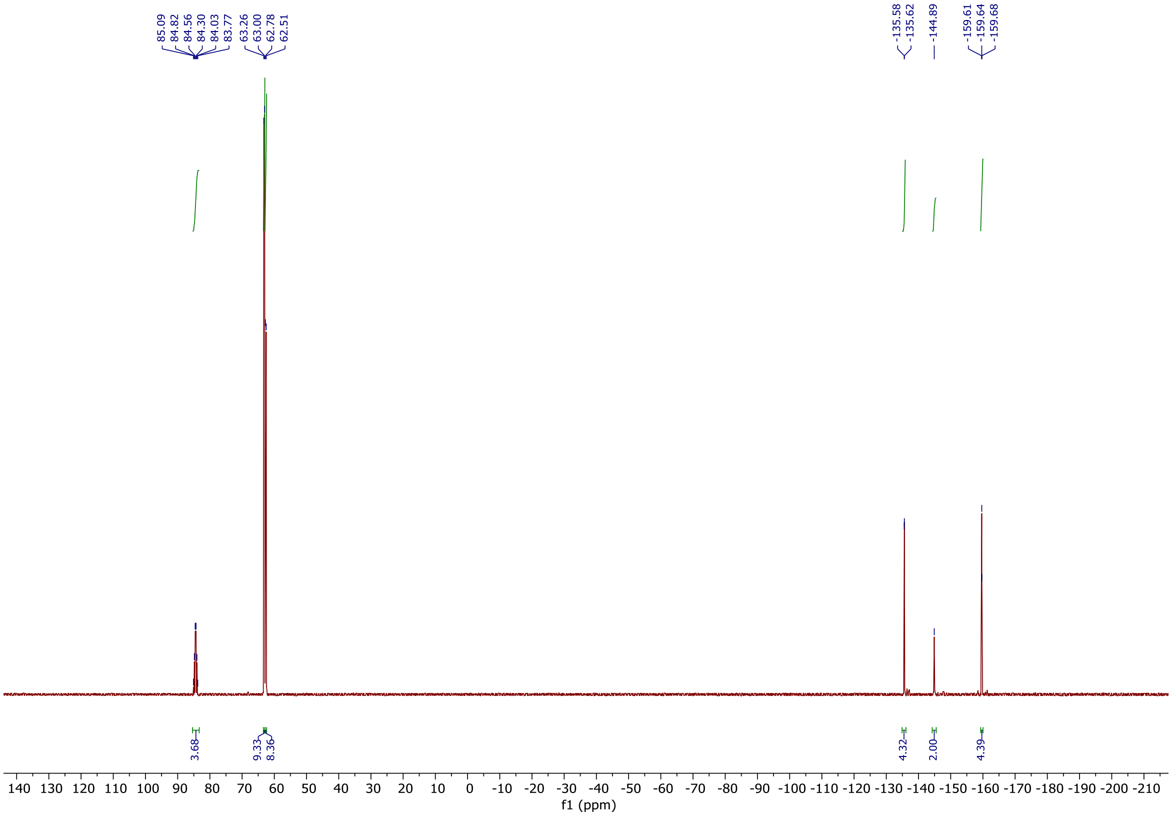

^{31}P NMR CDCl$_3$, 203 MHz

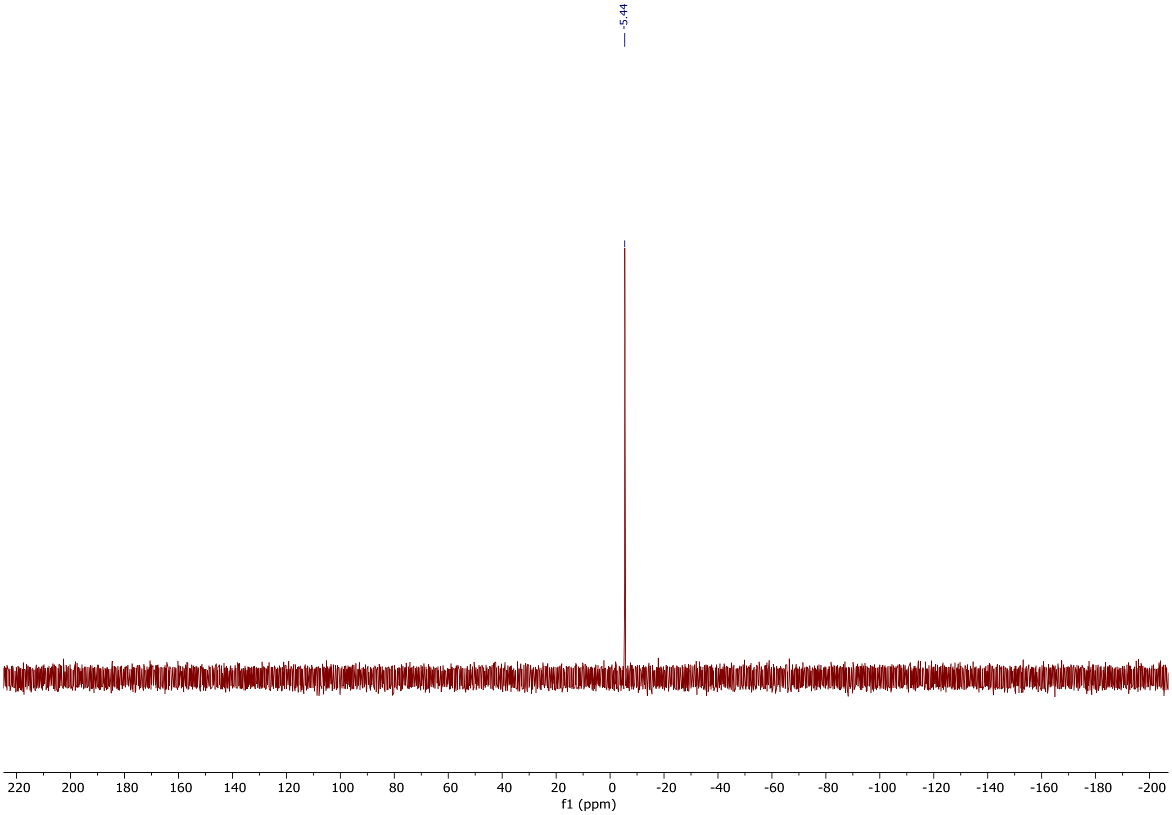

^{1}H NMR ($CDCl_3$, 500 MHz) of (11h)

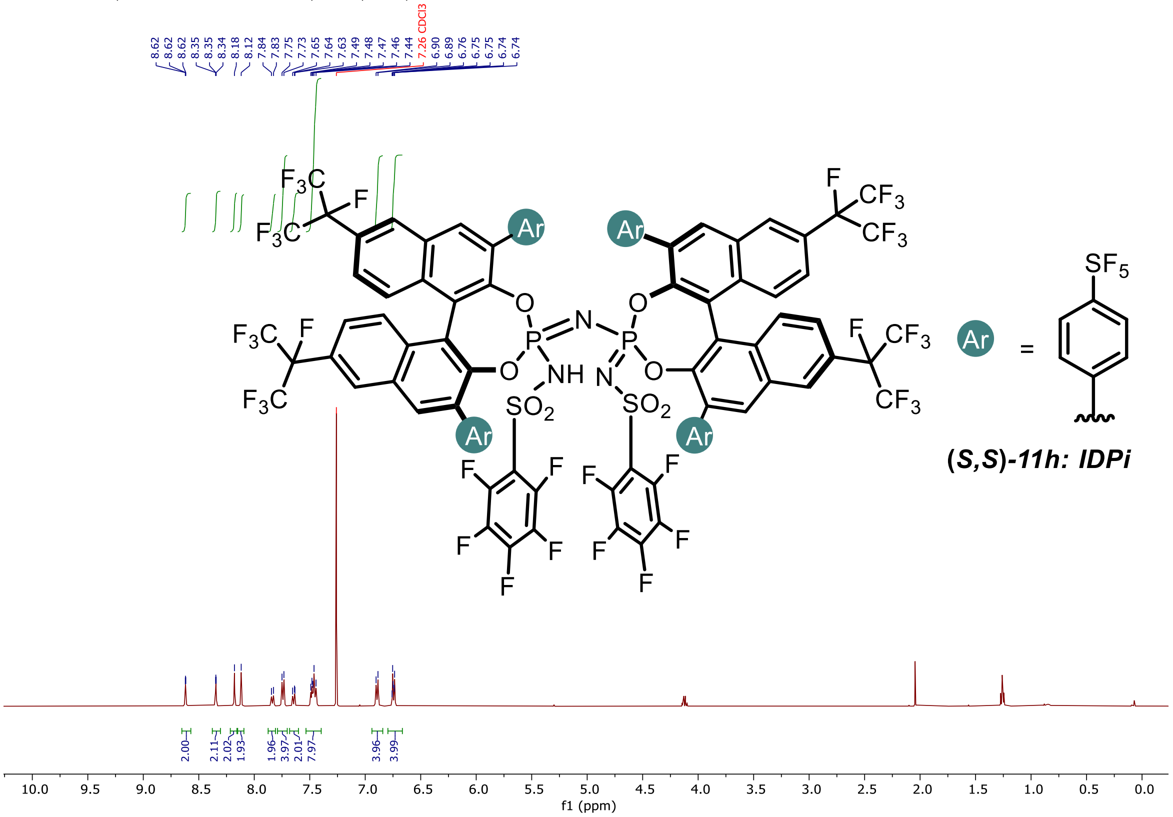

^{13}C NMR ($CDCl_3$, 126 MHz)

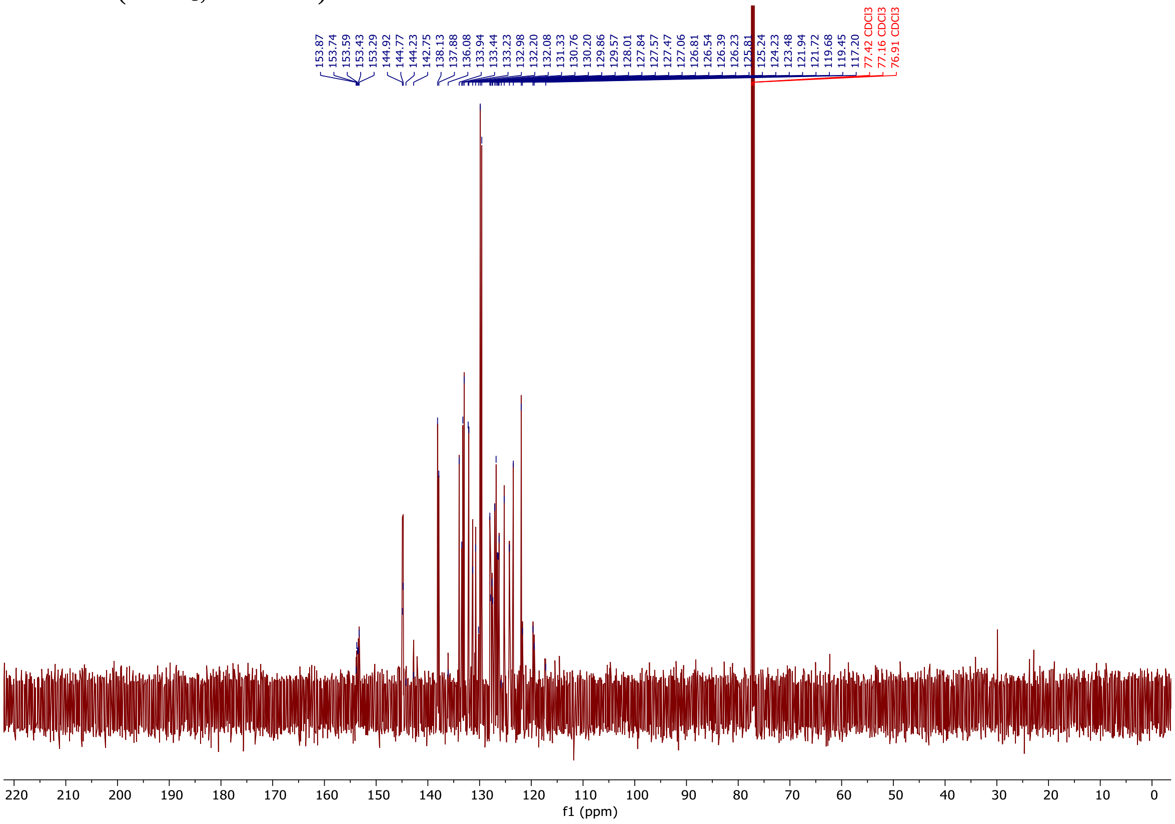

^{19}F NMR ($CDCl_3$, 471 MHz)

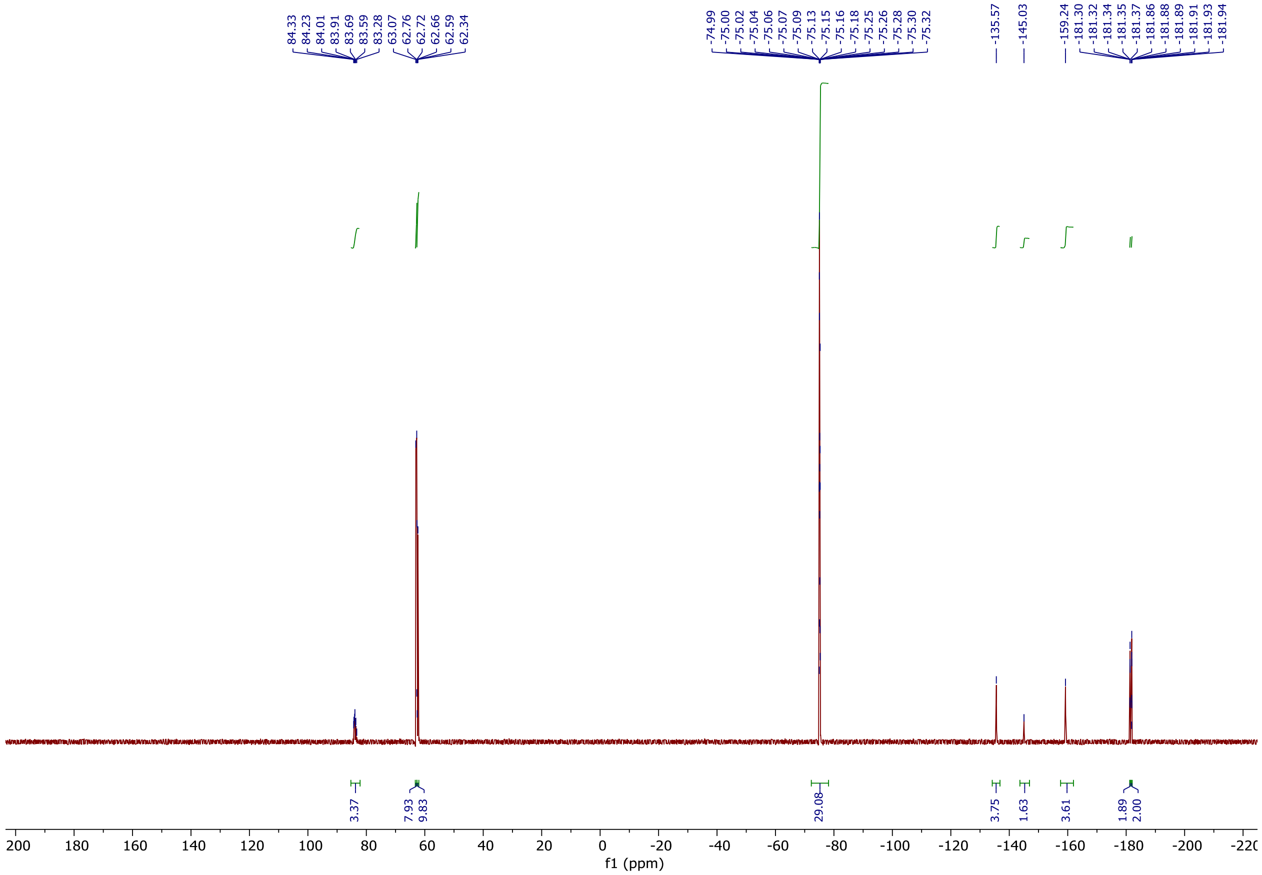

^{31}P NMR ($CDCl_3$, 203 MHz)

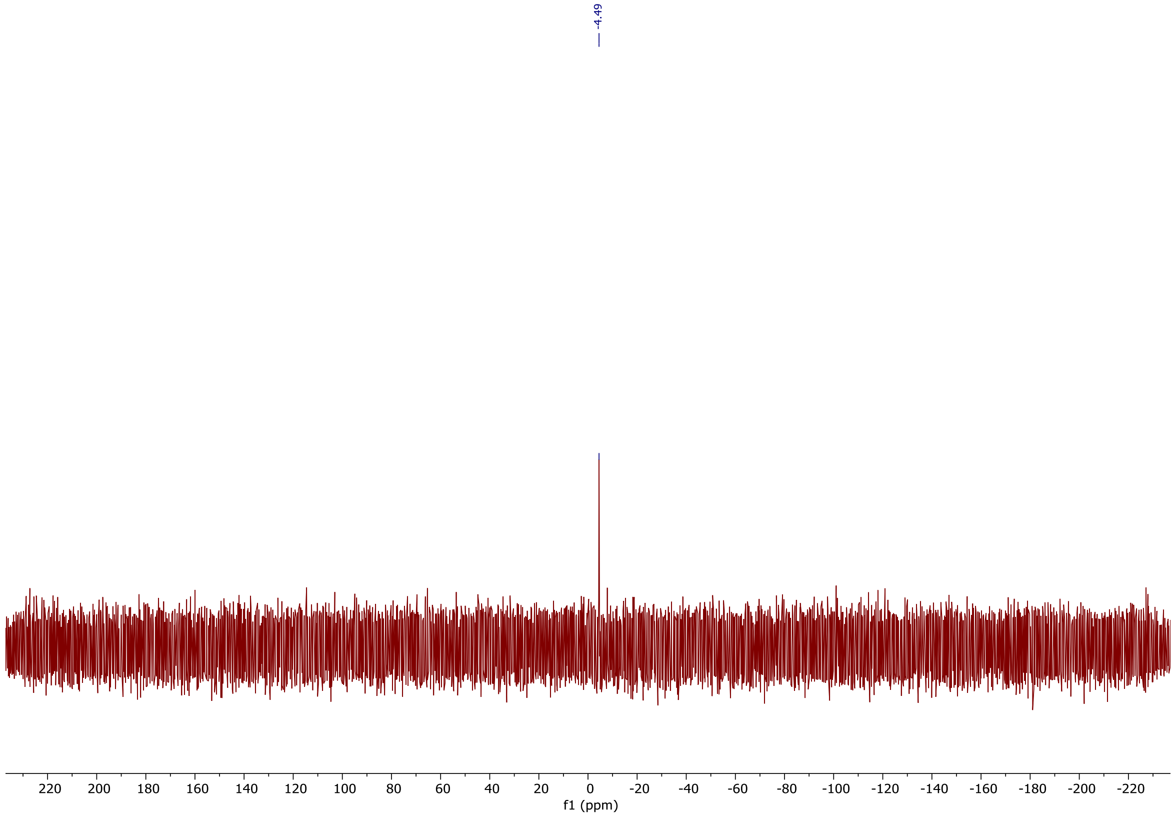

^{1}H NMR ($CDCl_3$, 501 MHz) of (7a)

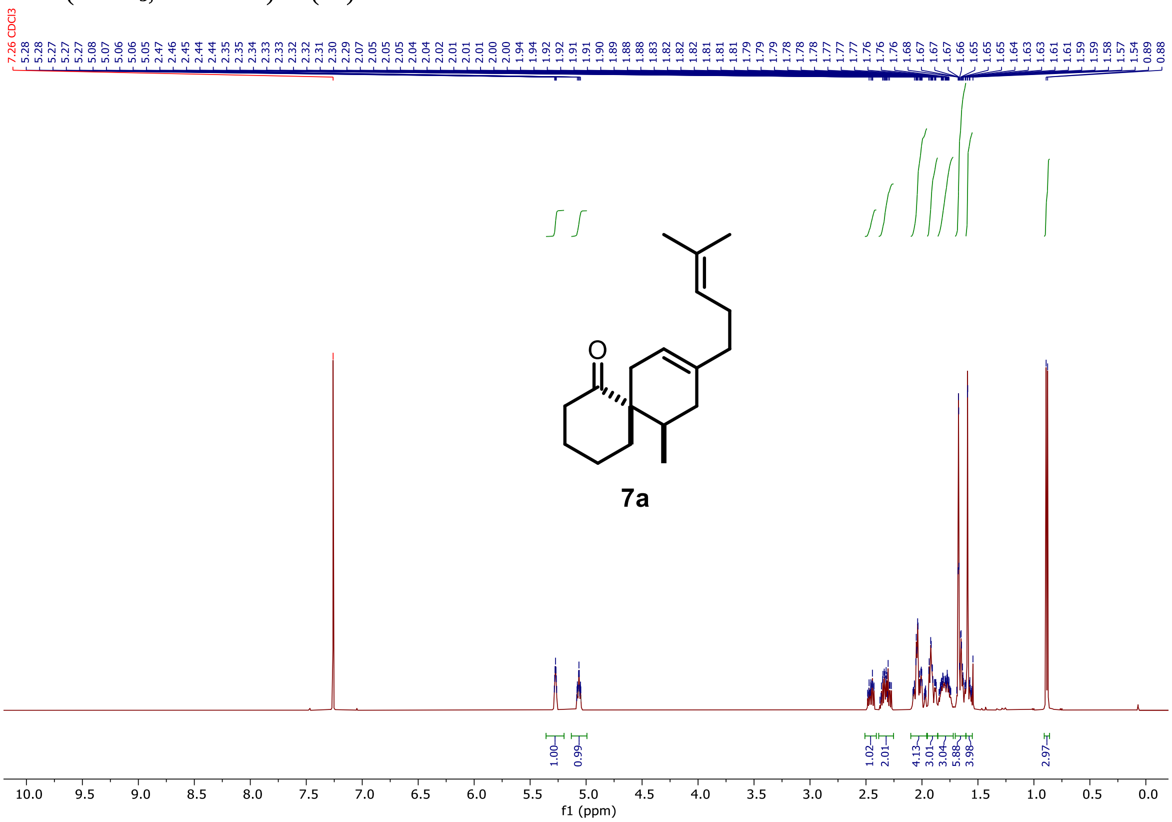

^{13}C NMR ($CDCl_3$, 126 MHz)

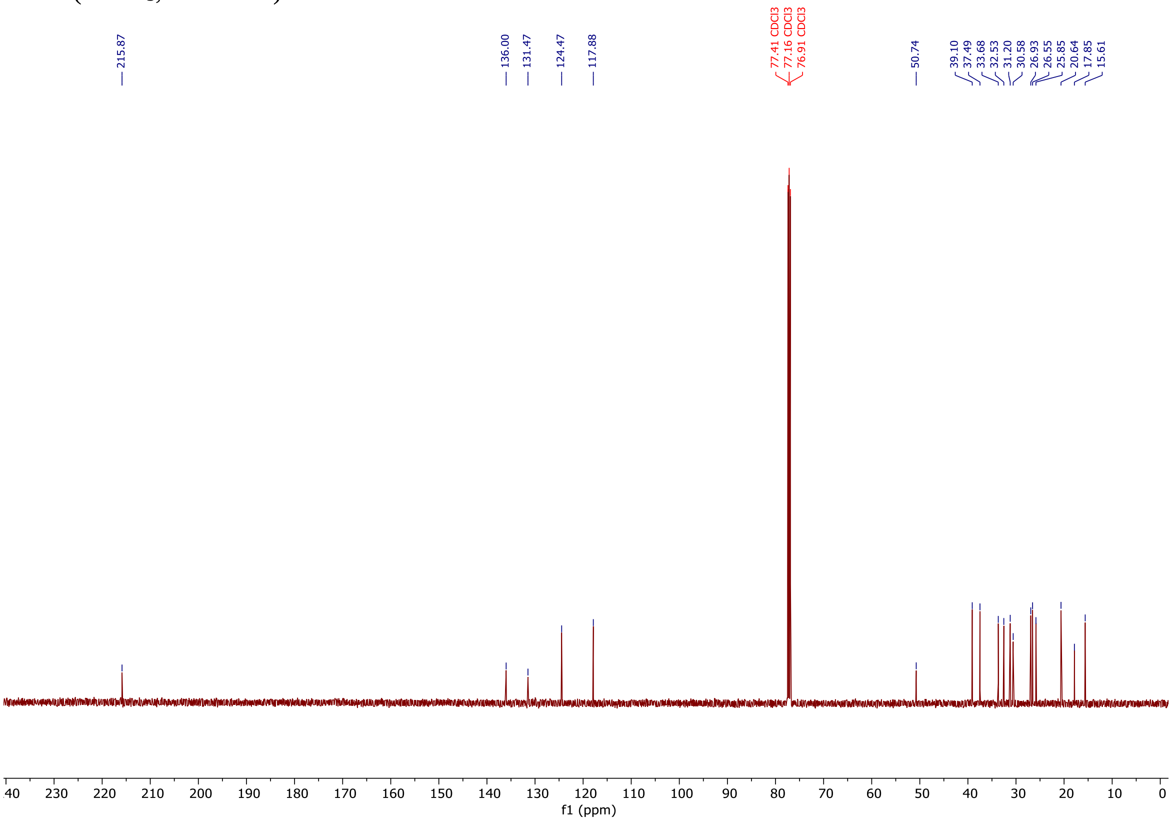

^{1}H NMR (CDCl$_3$, 501 MHz) of (7b)

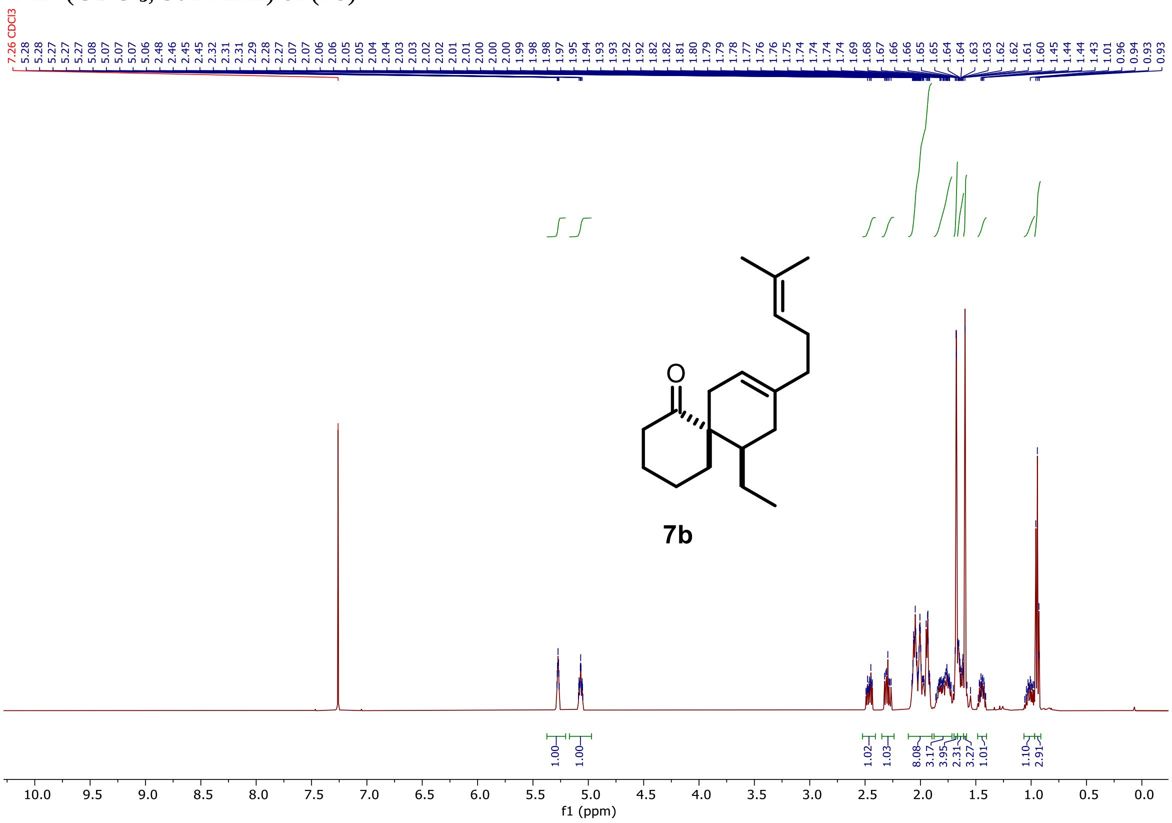

^{13}C NMR (CDCl$_3$, 126 MHz)

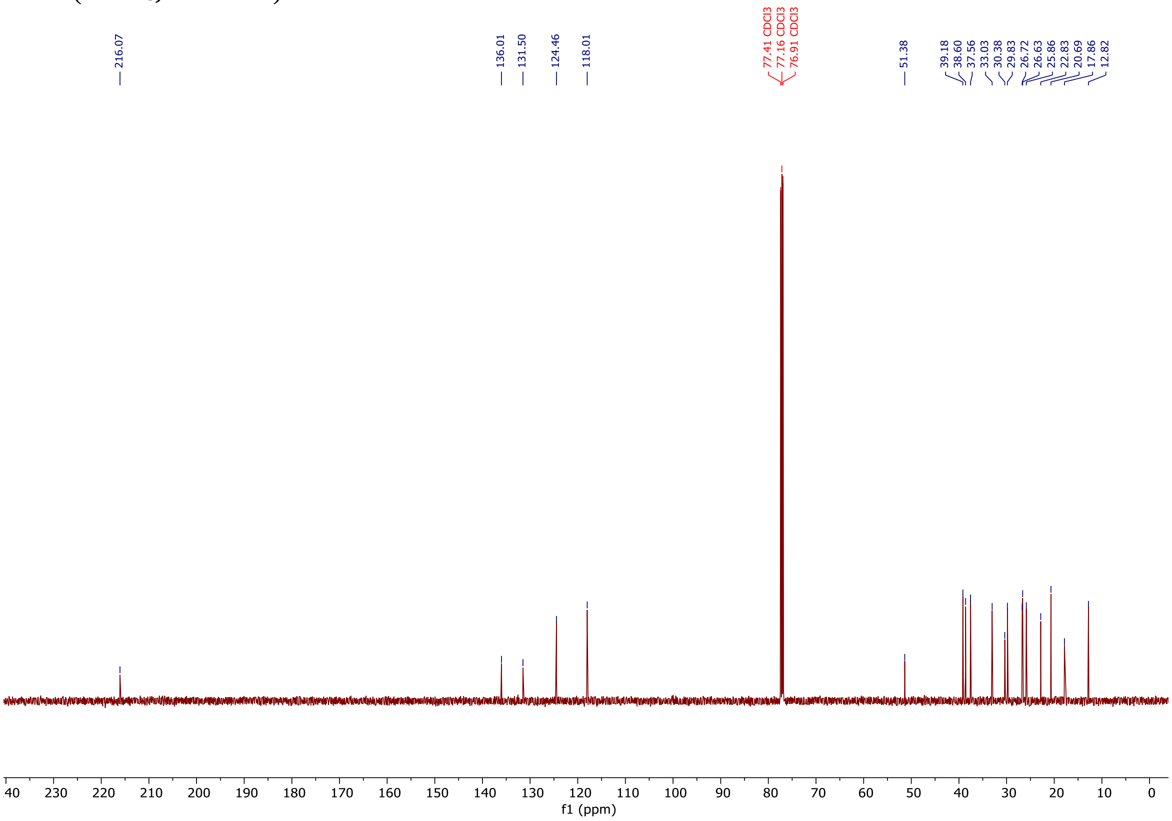

^{1}H NMR ($CDCl_3$, 501 MHz) of (7c)

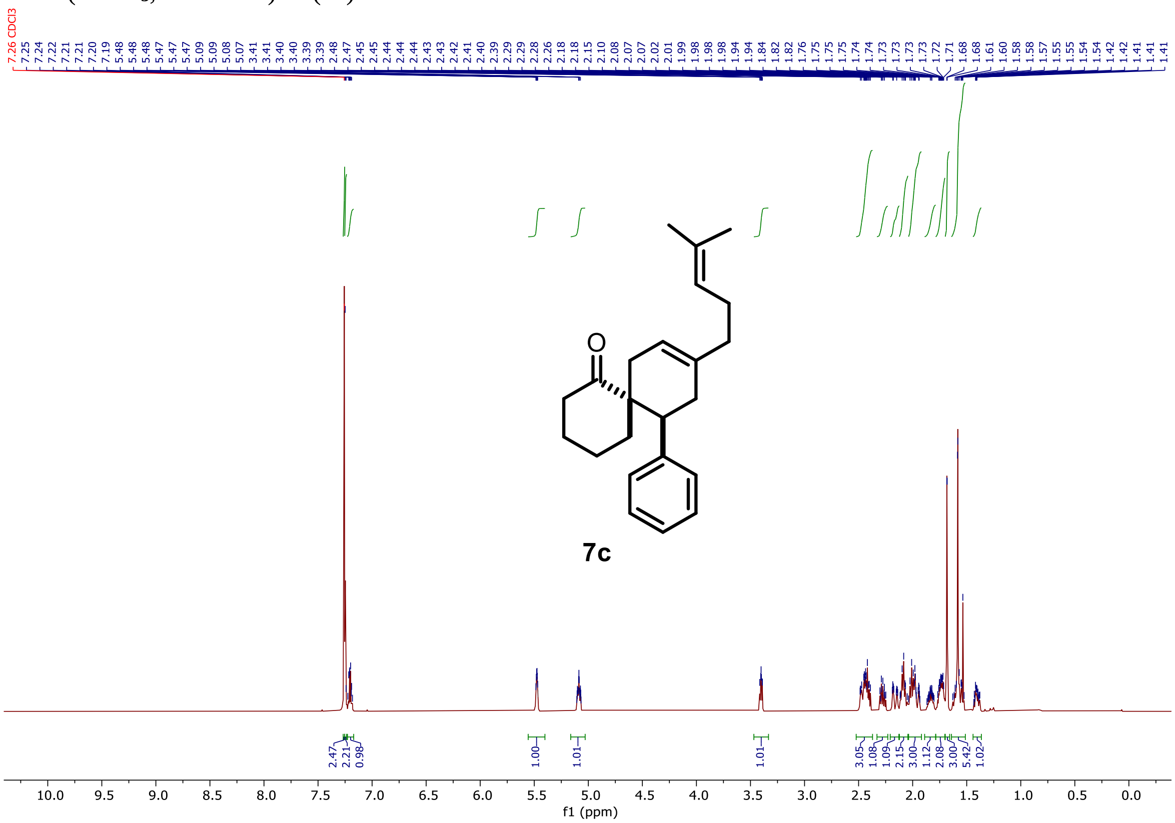

^{13}C NMR ($CDCl_3$, 126 MHz)

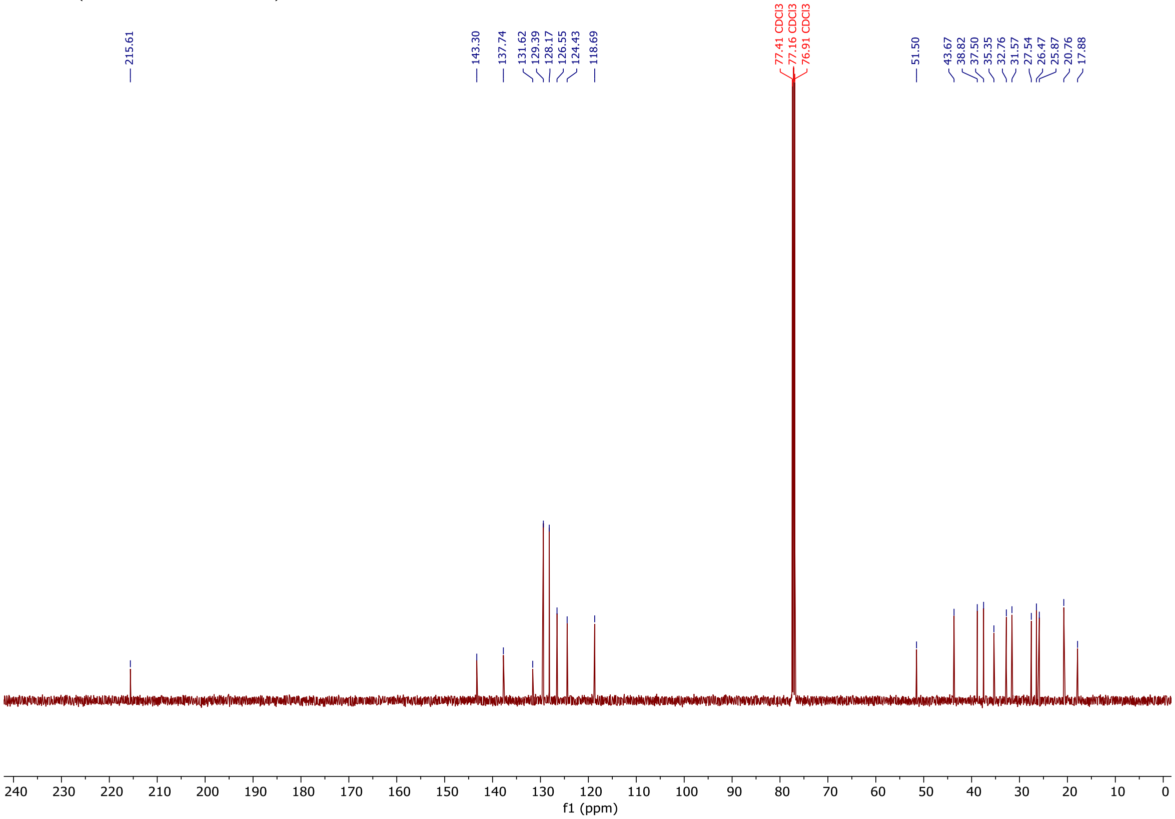

^{1}H NMR ($CDCl_3$, 501 MHz) of (7d)

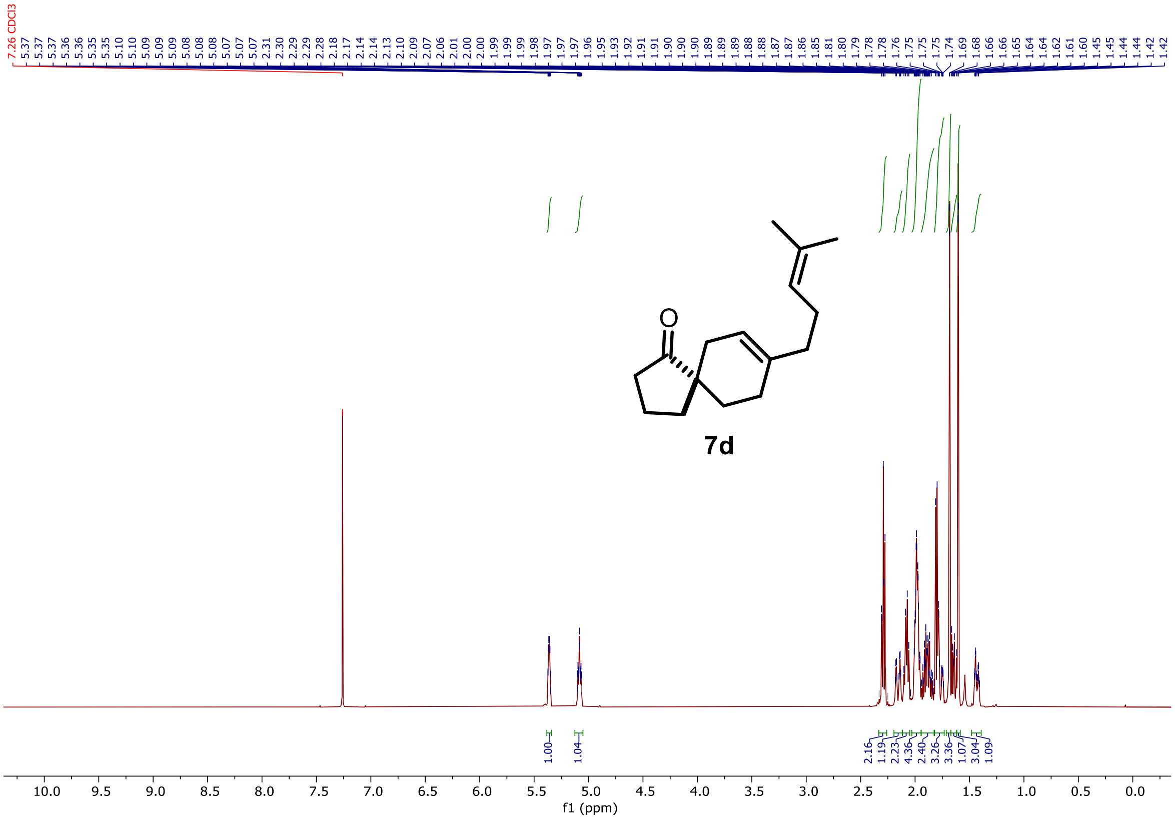

^{13}C NMR ($CDCl_3$, 126 MHz)

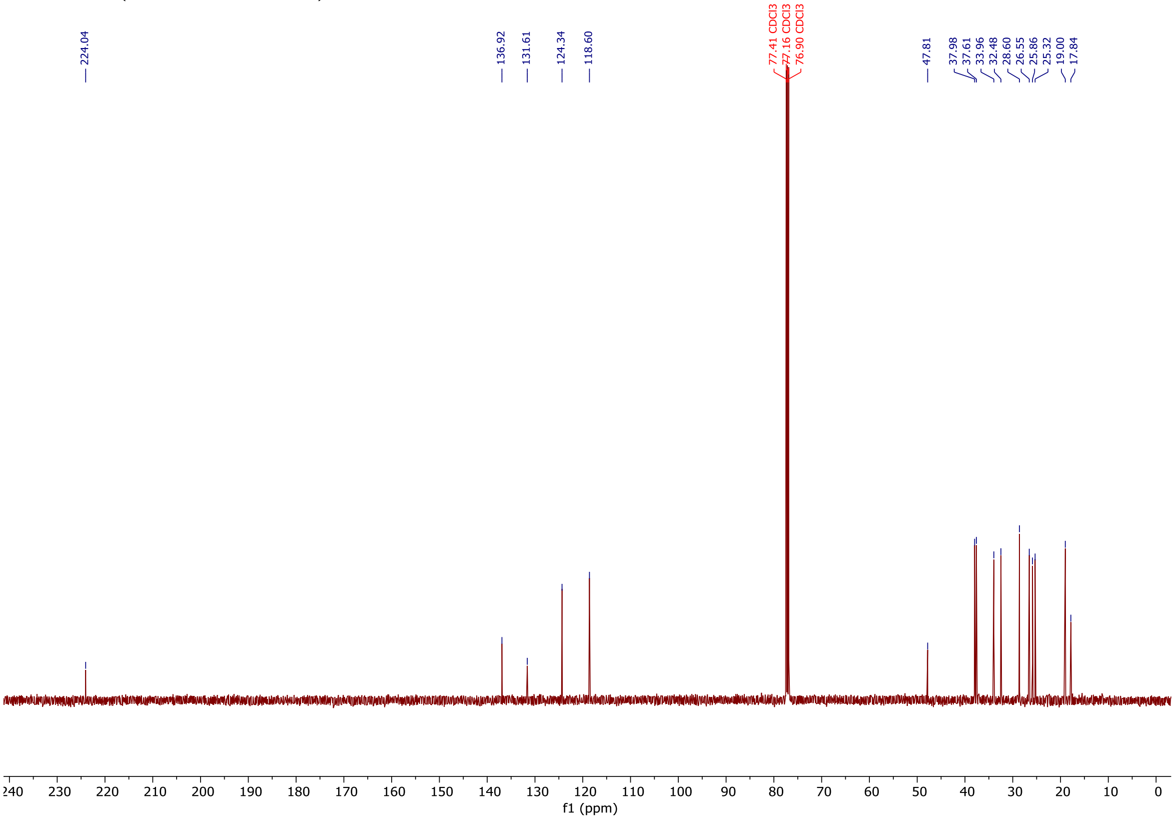

^{1}H NMR (CDCl$_3$, 501 MHz) of (7e)

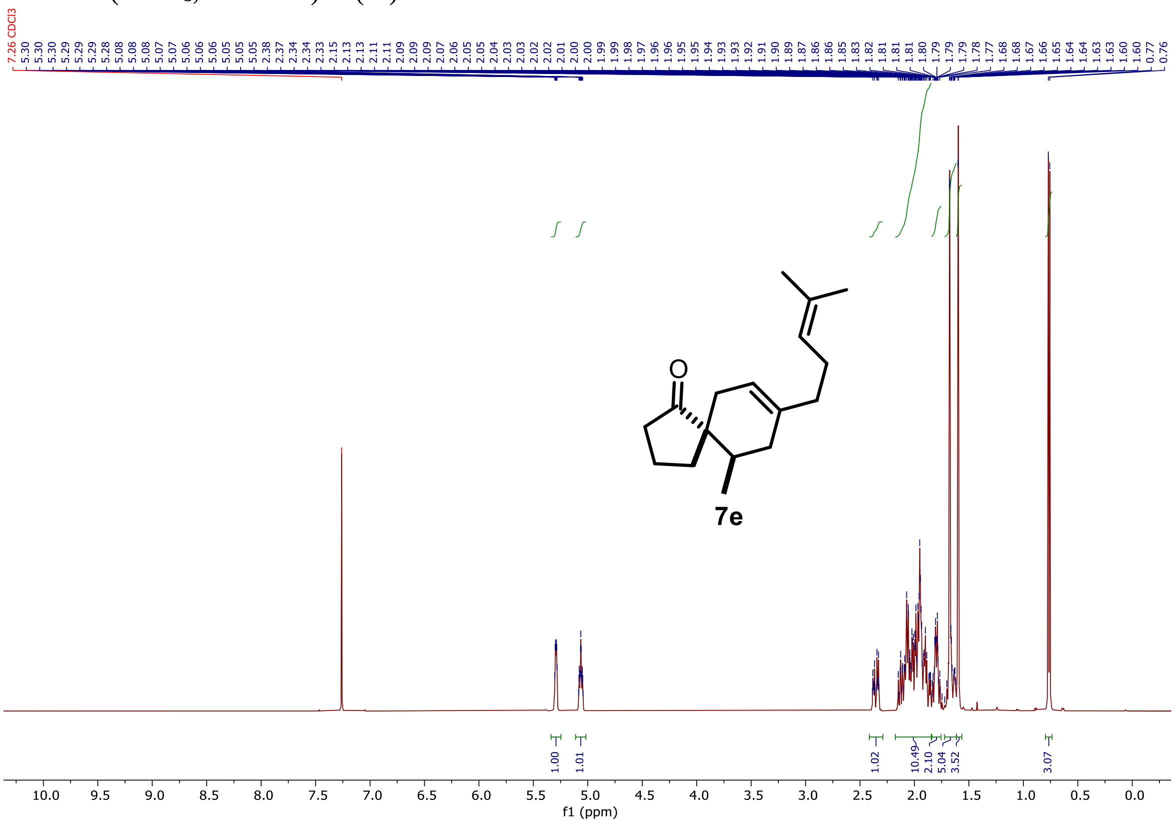

^{13}C NMR (CDCl$_3$, 126 MHz)

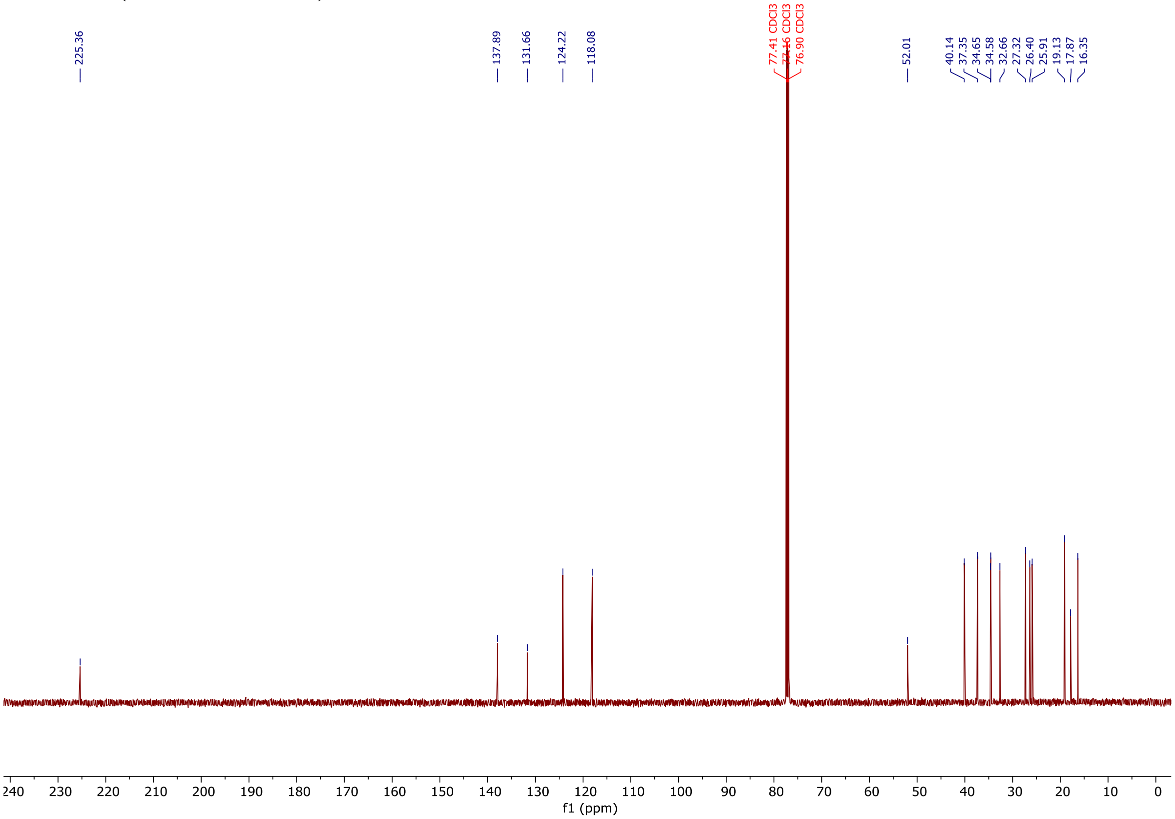

^{1}H NMR ($CDCl_3$, 501MHz) of (7f)

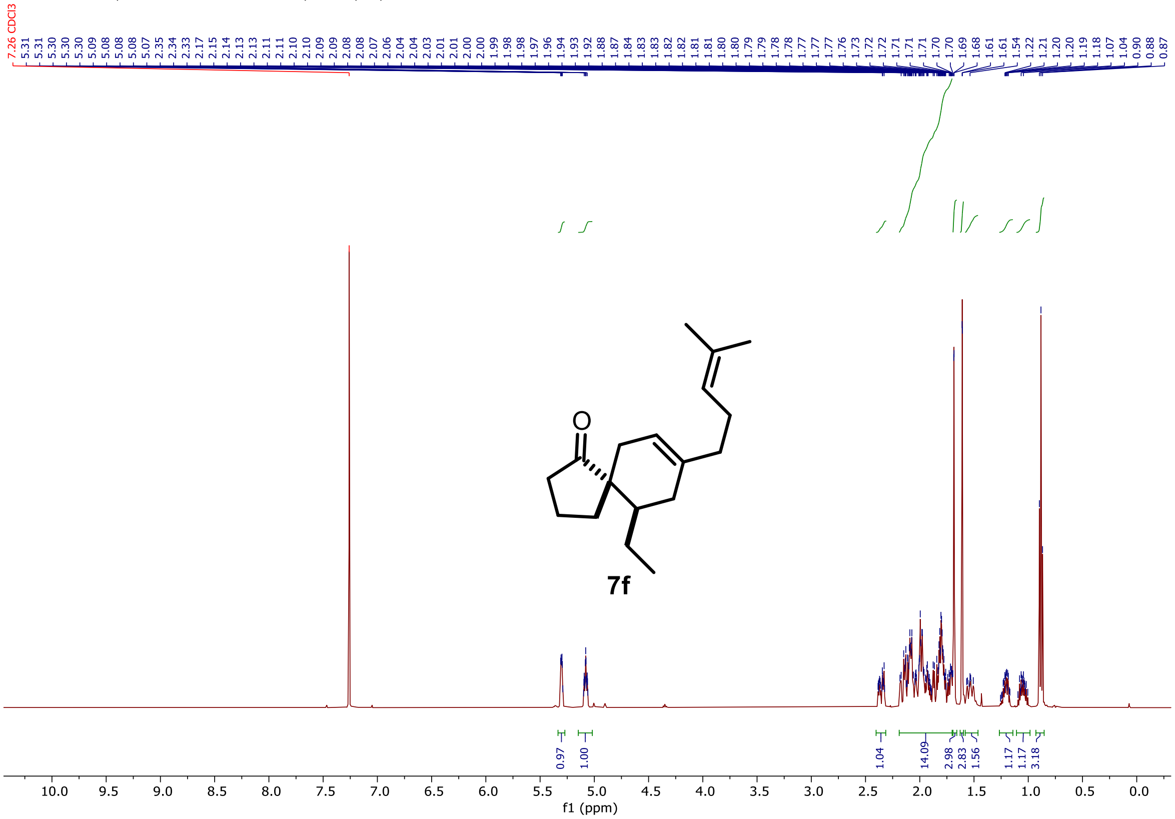

^{13}C NMR ($CDCl_3$, 126 MHz)

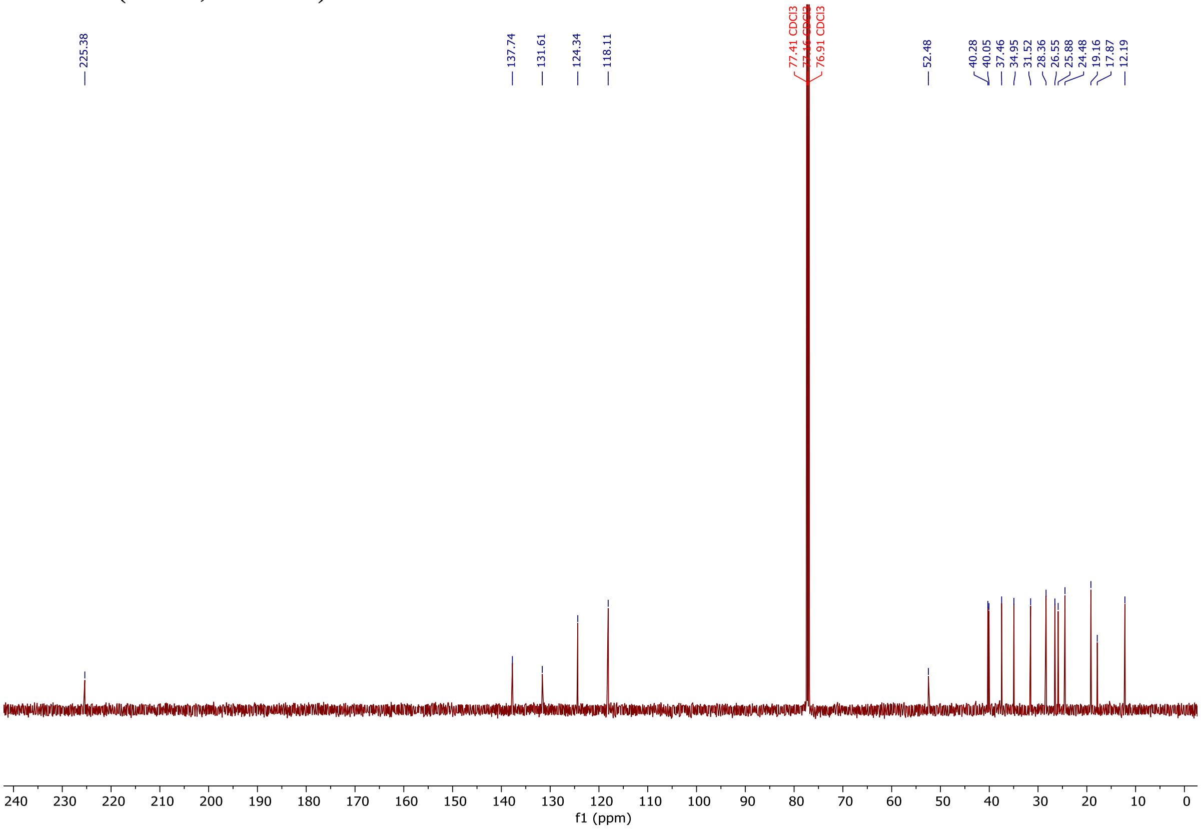

^{1}H NMR ($CDCl_3$, 501 MHz) of (7g)

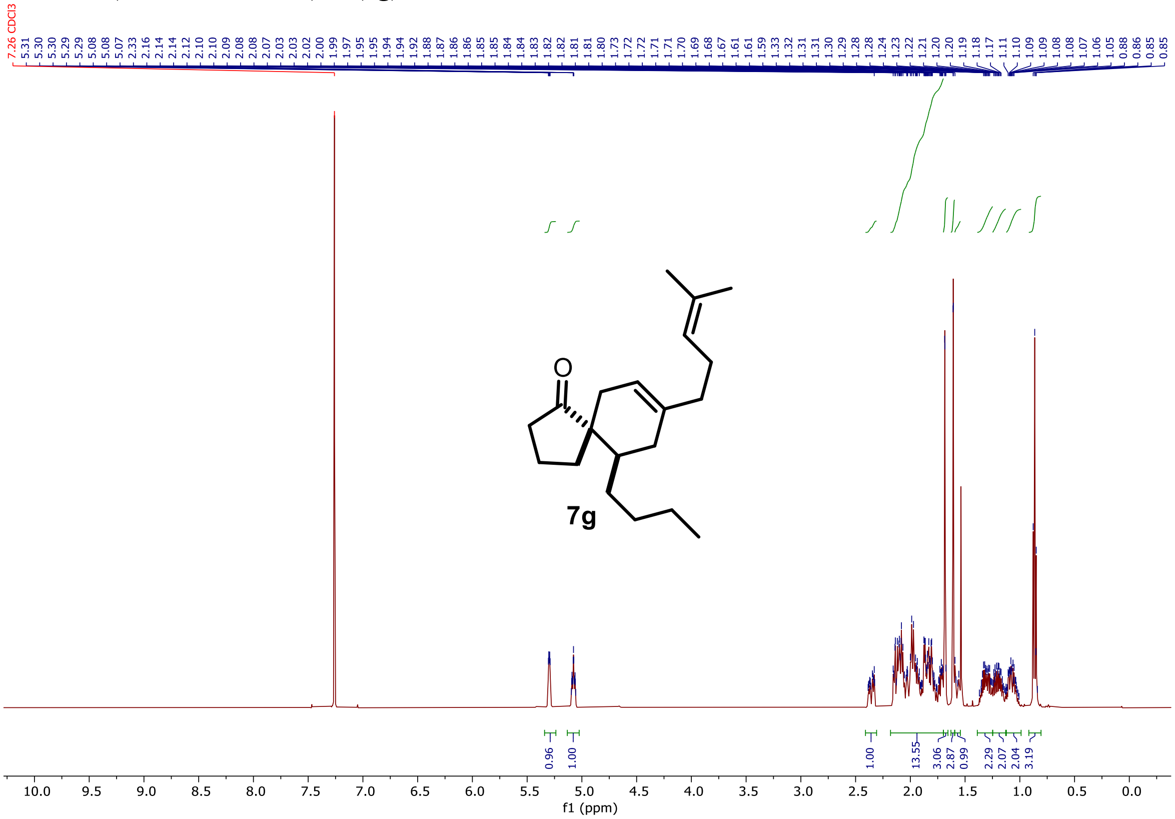

^{13}C NMR ($CDCl_3$, 126 MHz)

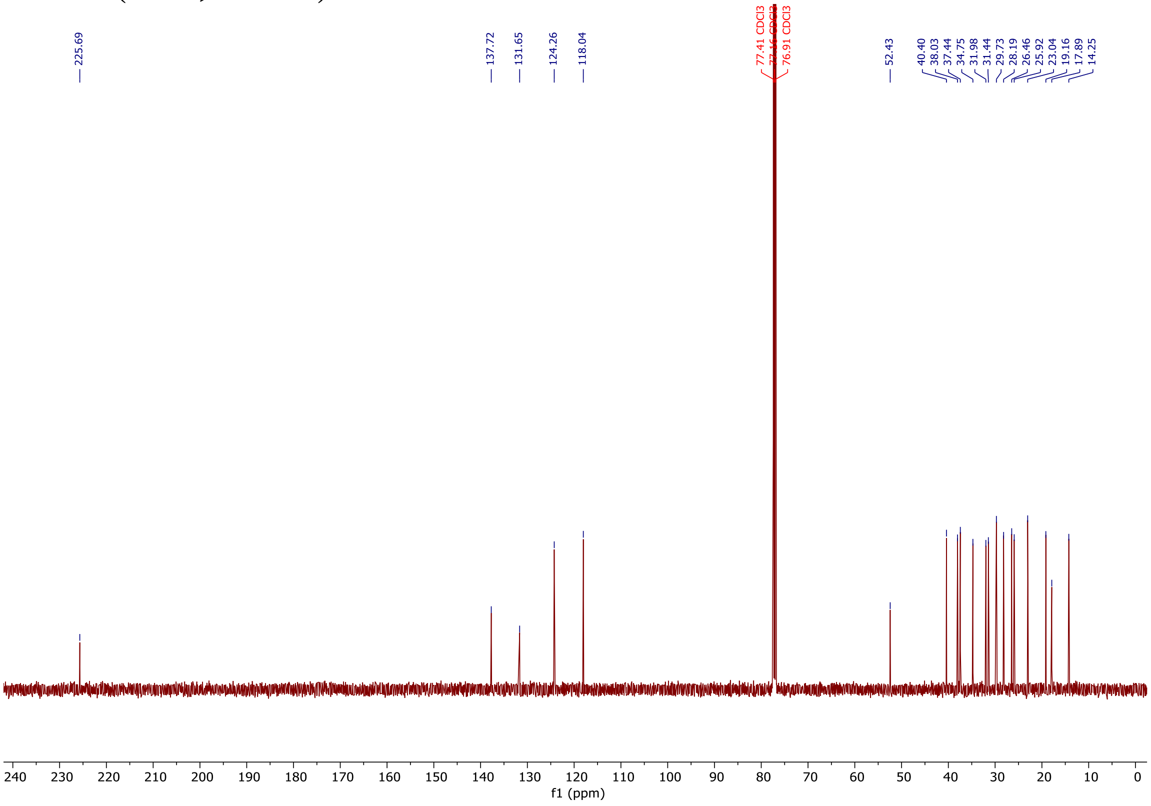

^{1}H NMR (CDCl$_3$, 501 MHz) of (7h)

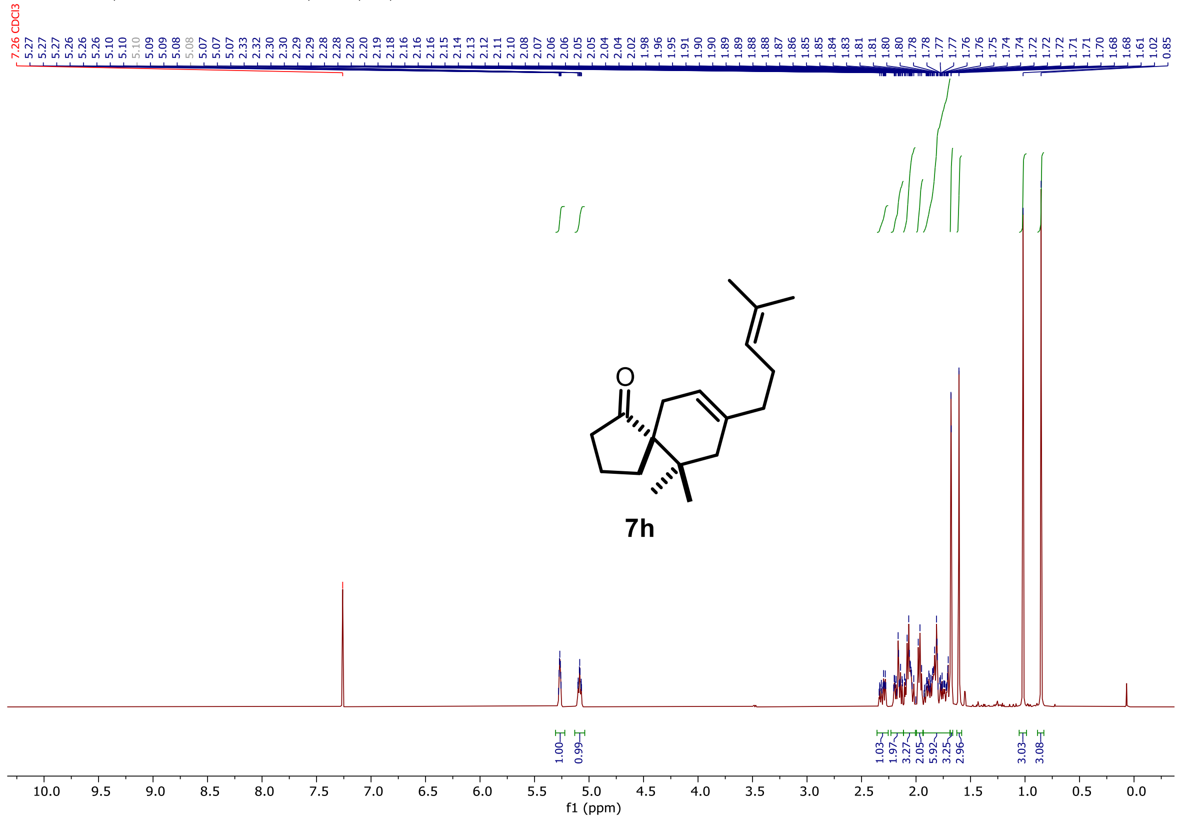

^{13}C NMR (CDCl$_3$, 126 MHz)

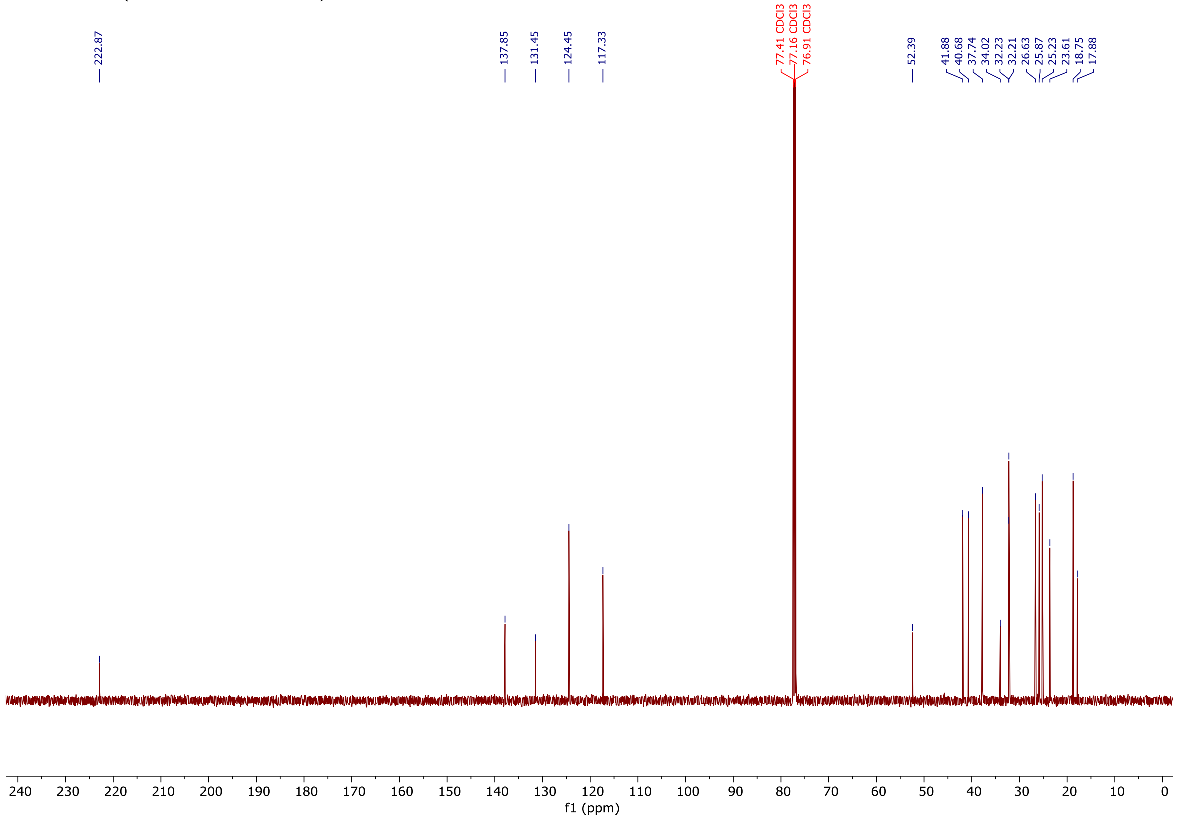

^{1}H NMR ($CDCl_3$, 600 MHz) of (7i and 7i')

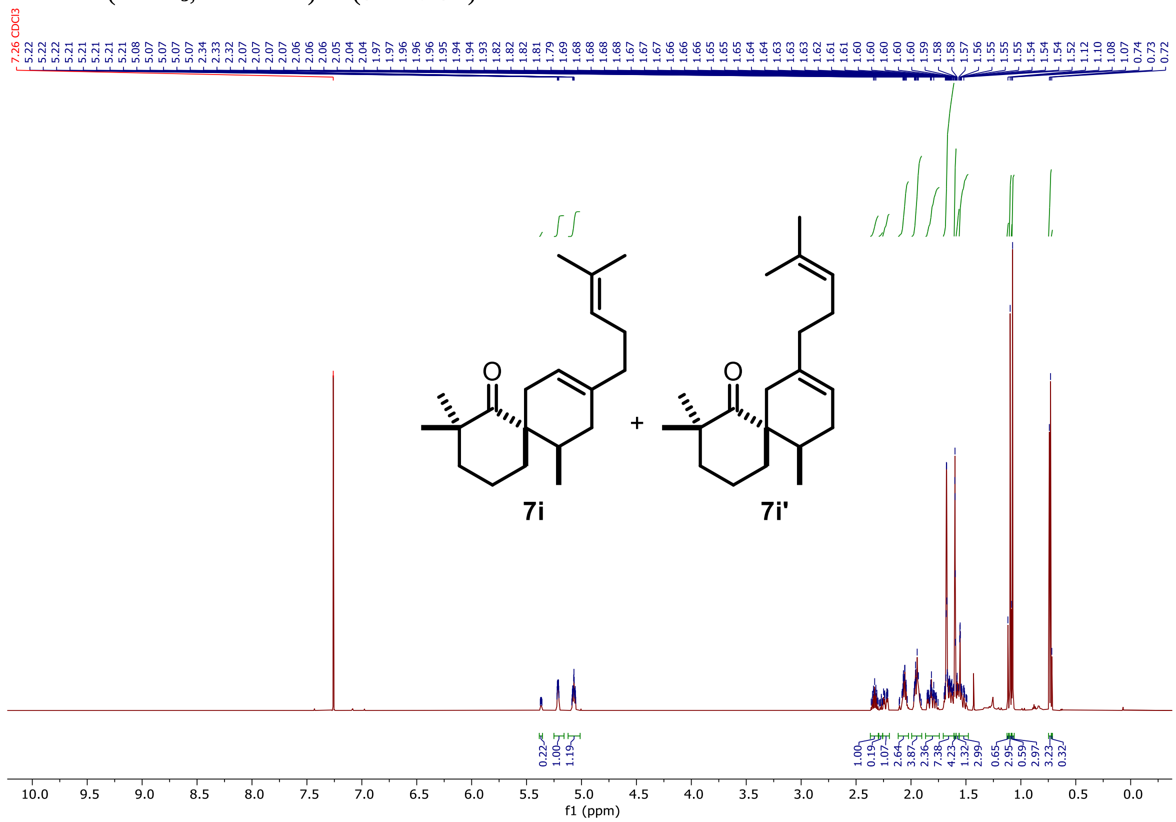

^{13}C NMR ($CDCl_3$, 151 MHz)

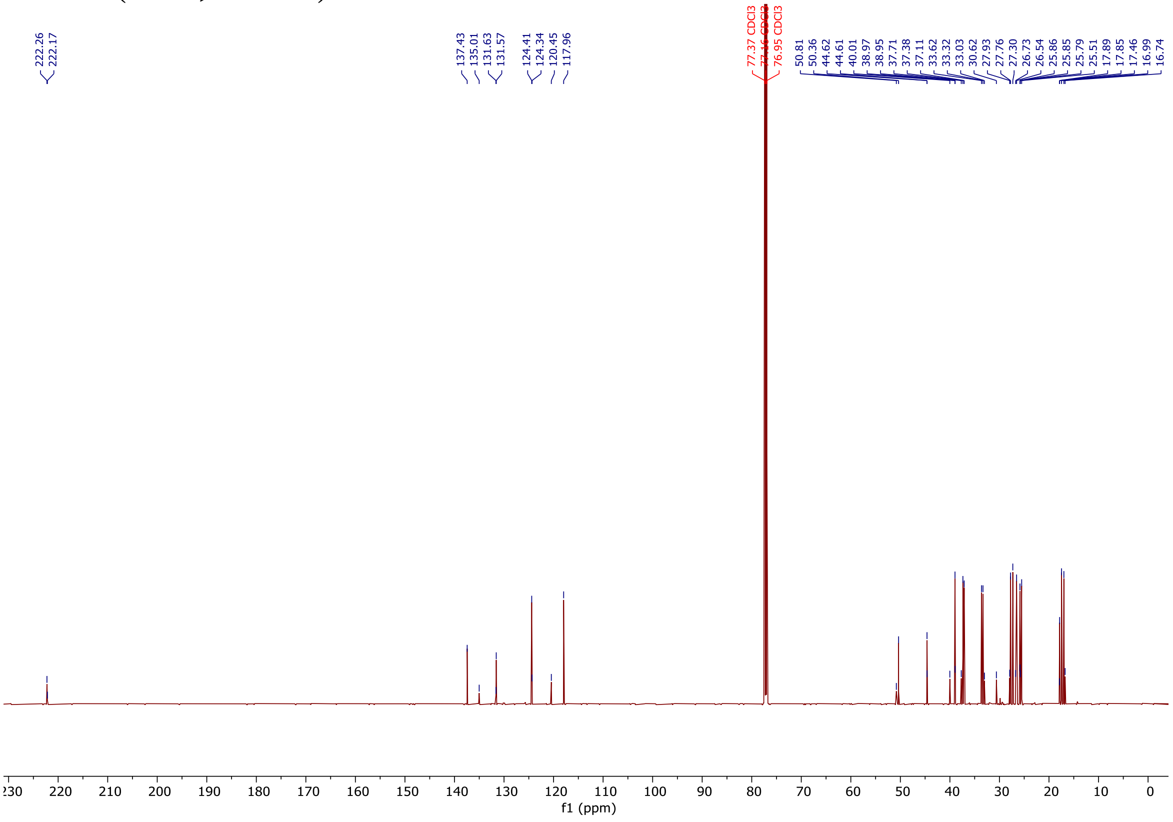

NOESY spectra

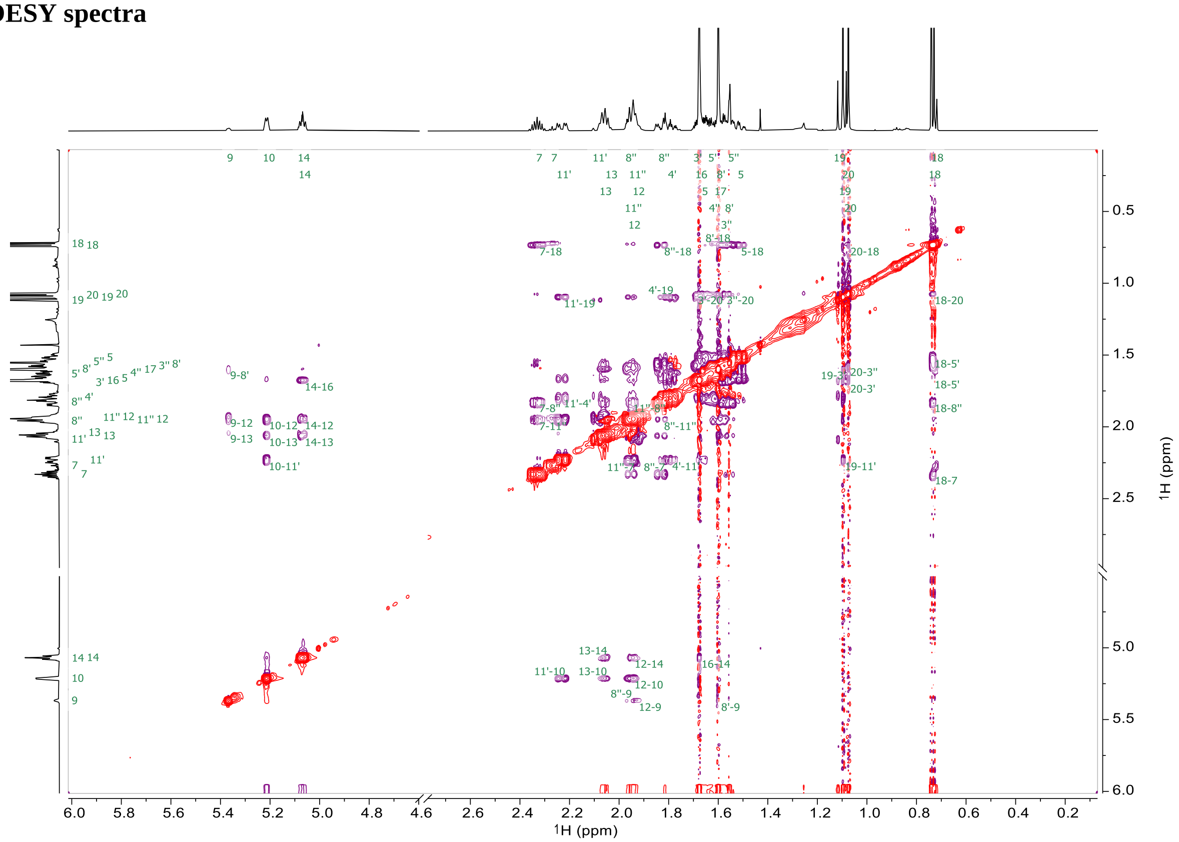

COSY spectra

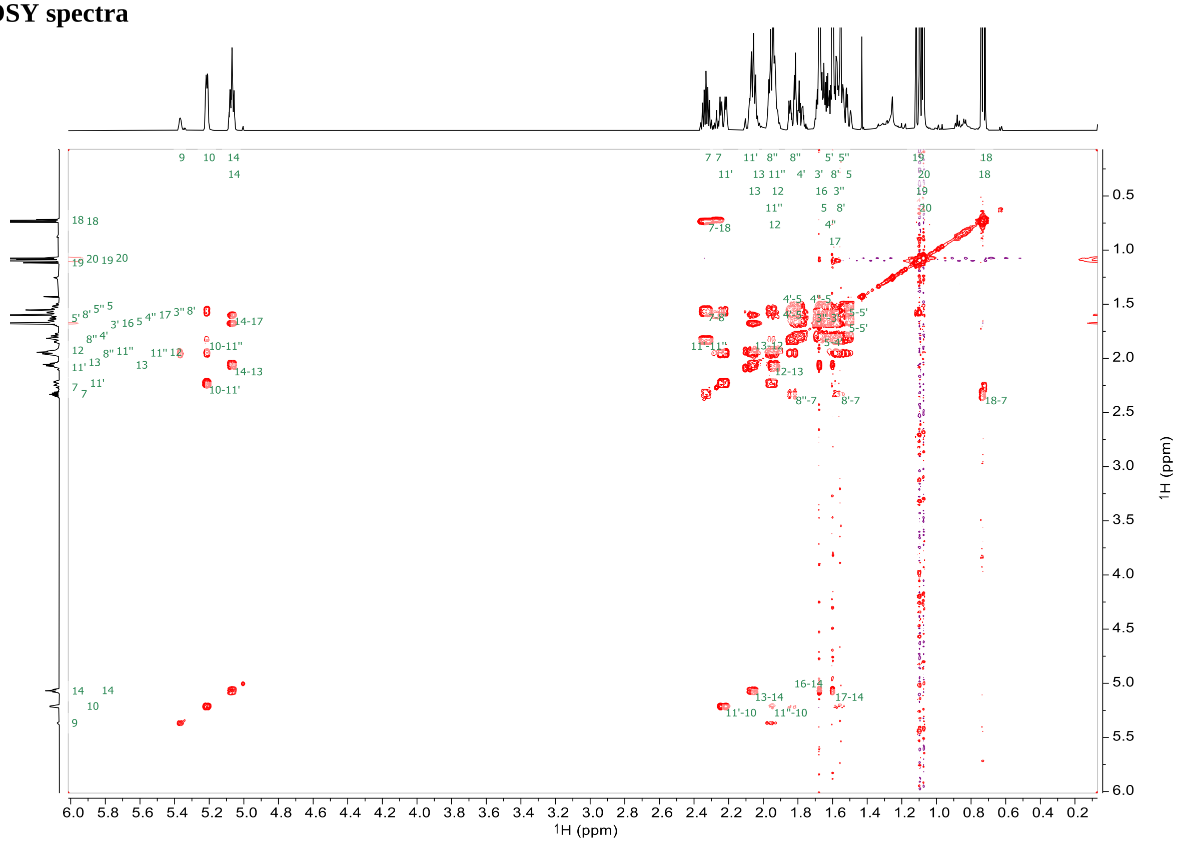

HMBC spectra

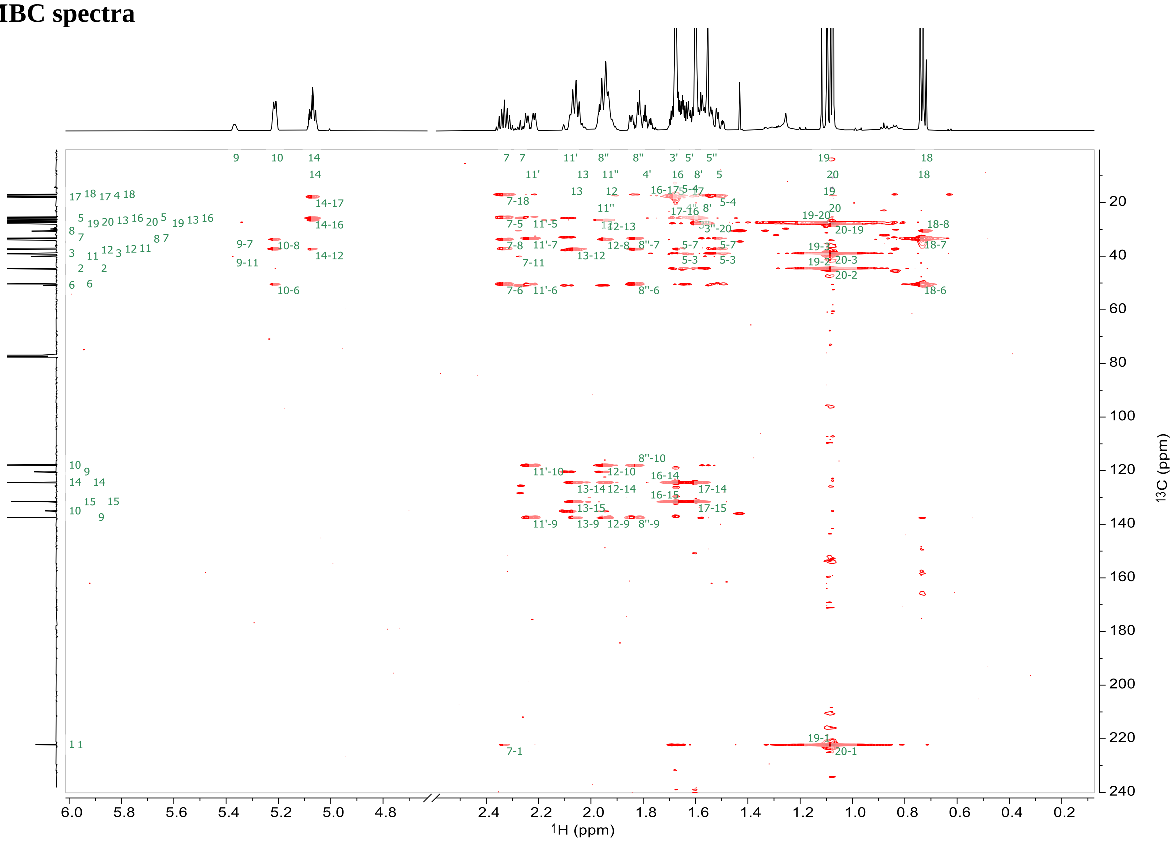

HSQC spectra

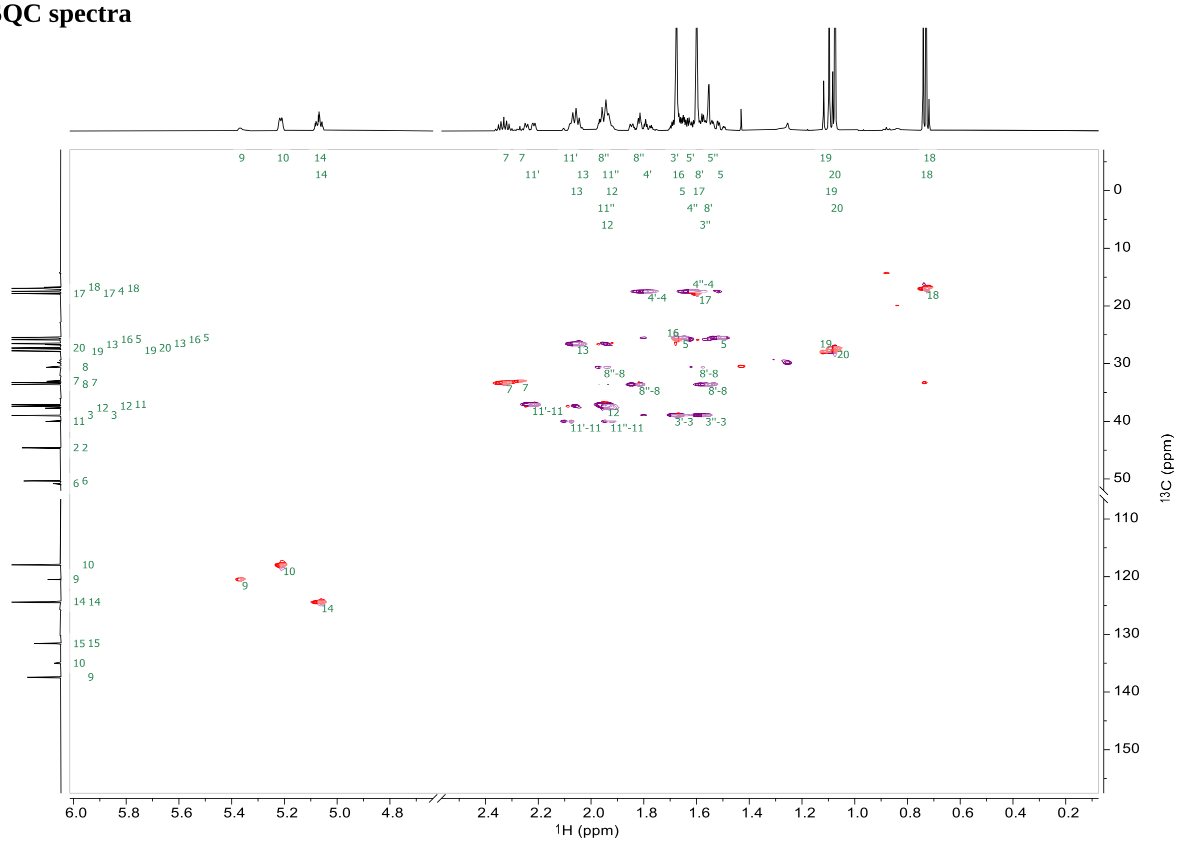

^{1}H NMR ($CDCl_3$, 501 MHz) of (7j and 7j')

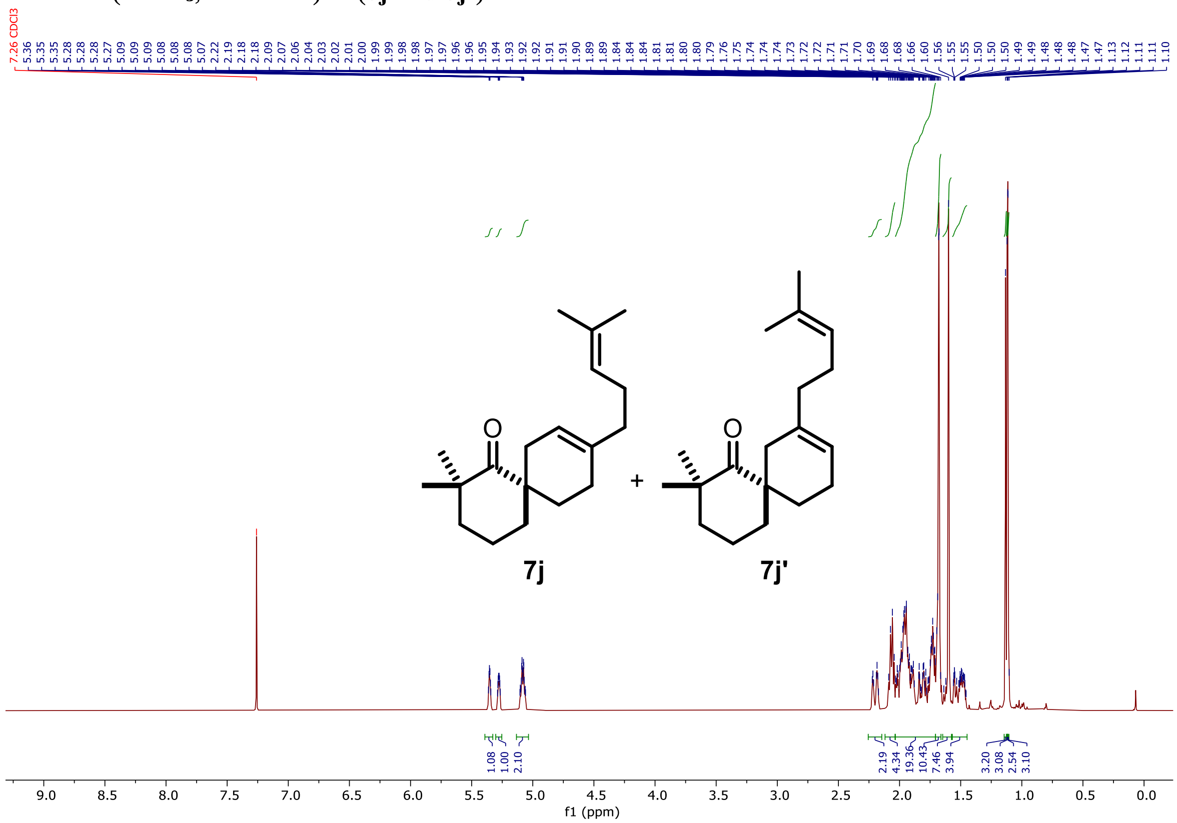

^{13}C NMR ($CDCl_3$, 126 MHz)

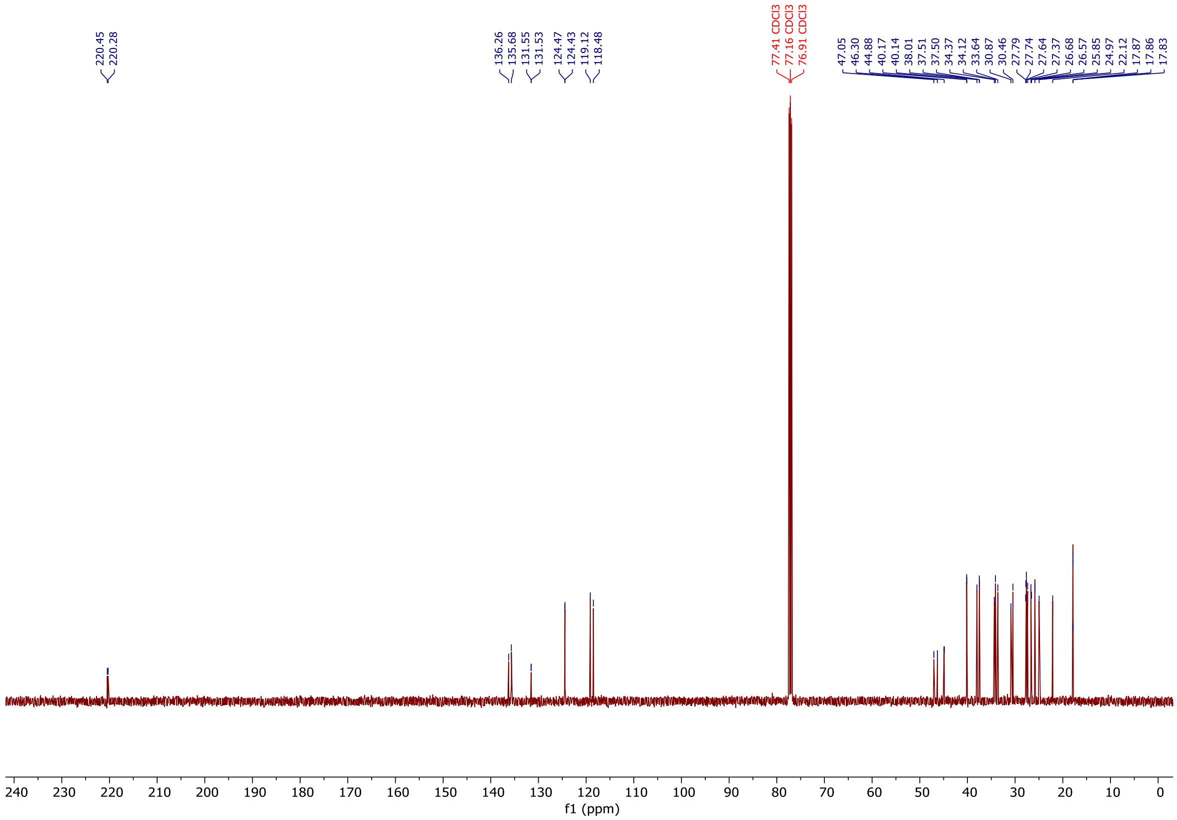

^{1}H NMR (CDCl$_3$, 501 MHz) of (7k)

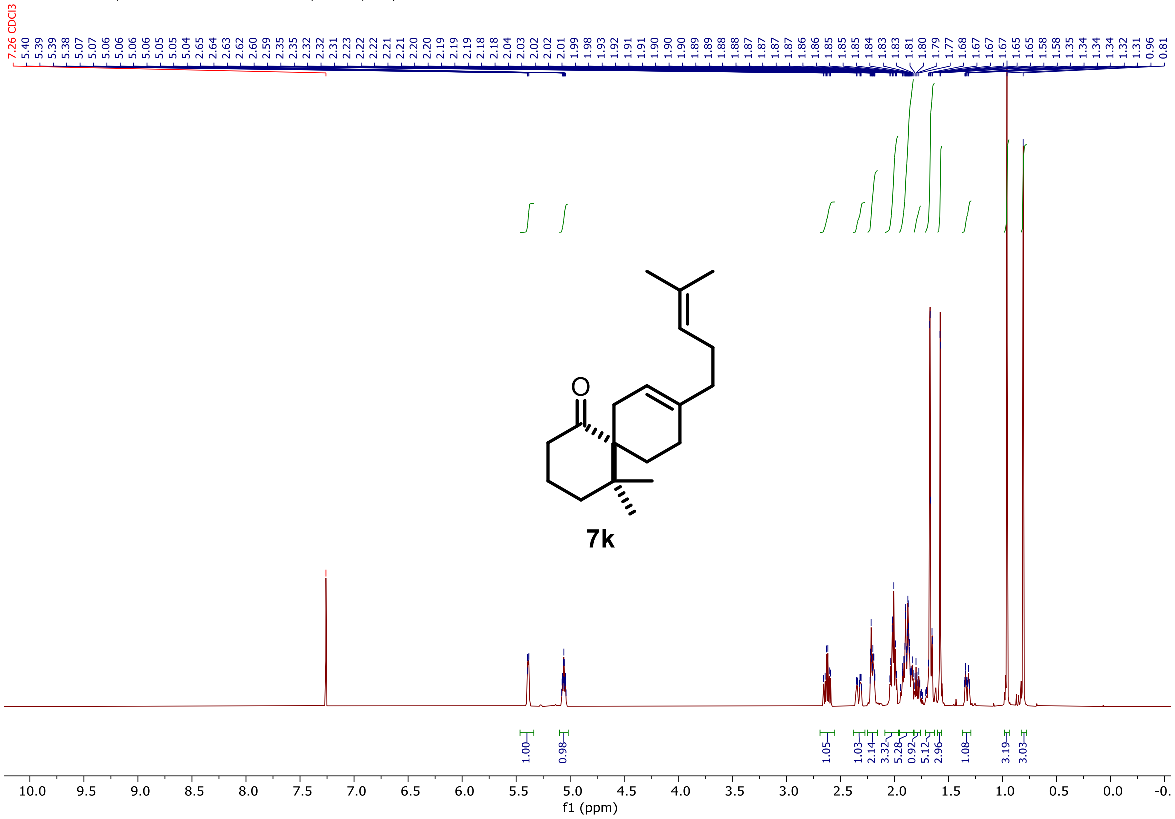

^{13}C NMR (CDCl$_3$, 126 MHz)

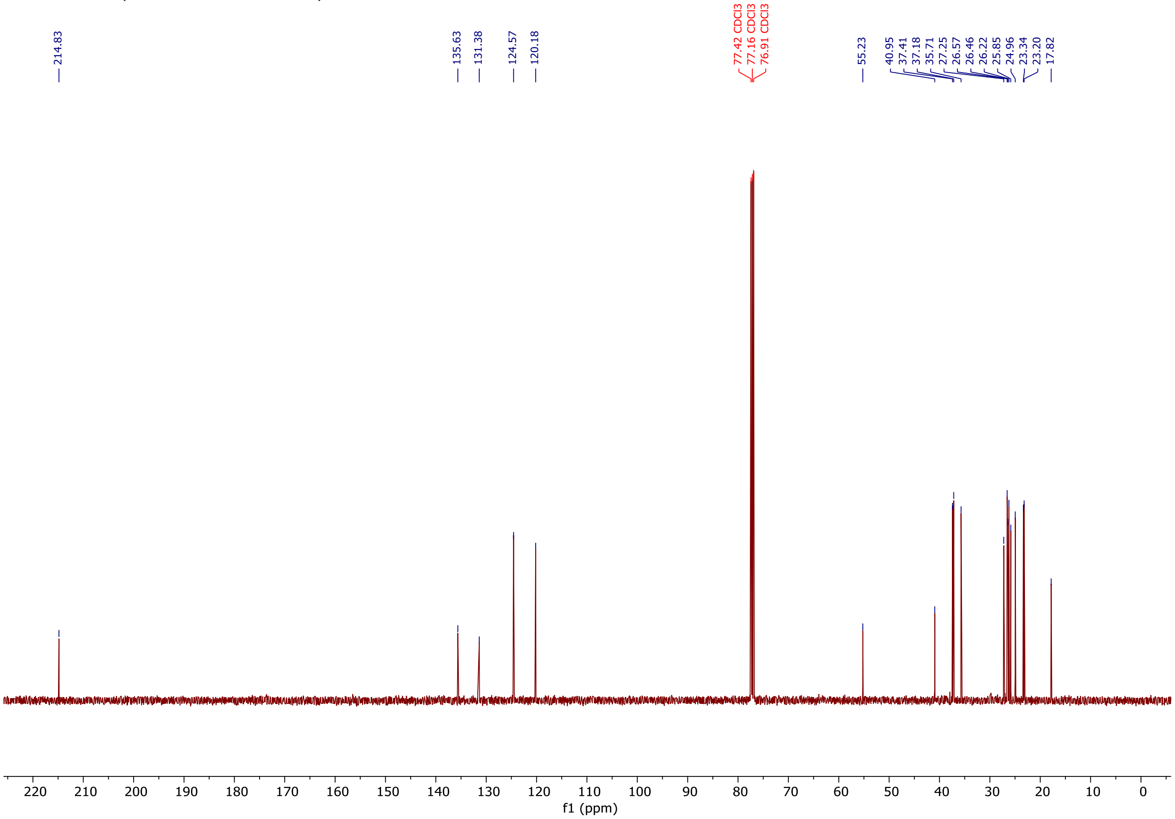

^{1}H NMR (CDCl$_3$, 501 MHz) of (7l')

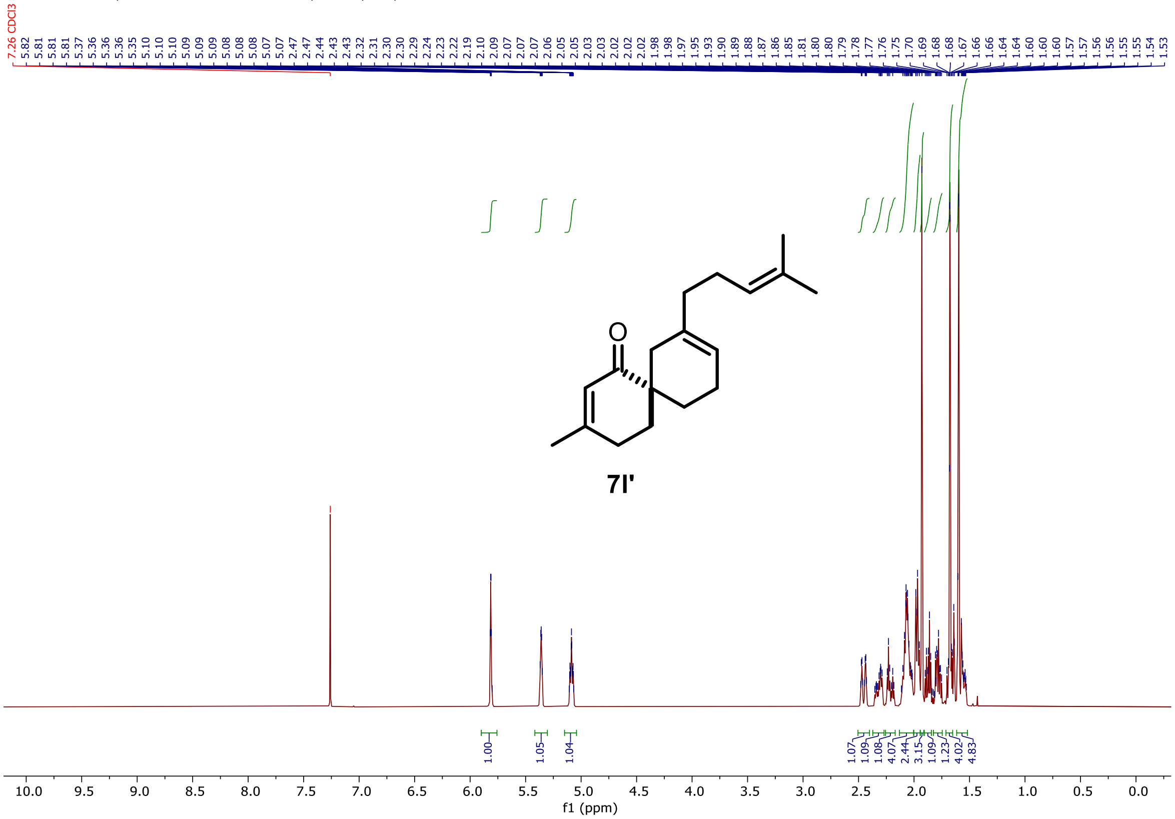

^{13}C NMR (CDCl$_3$, 126 MHz)

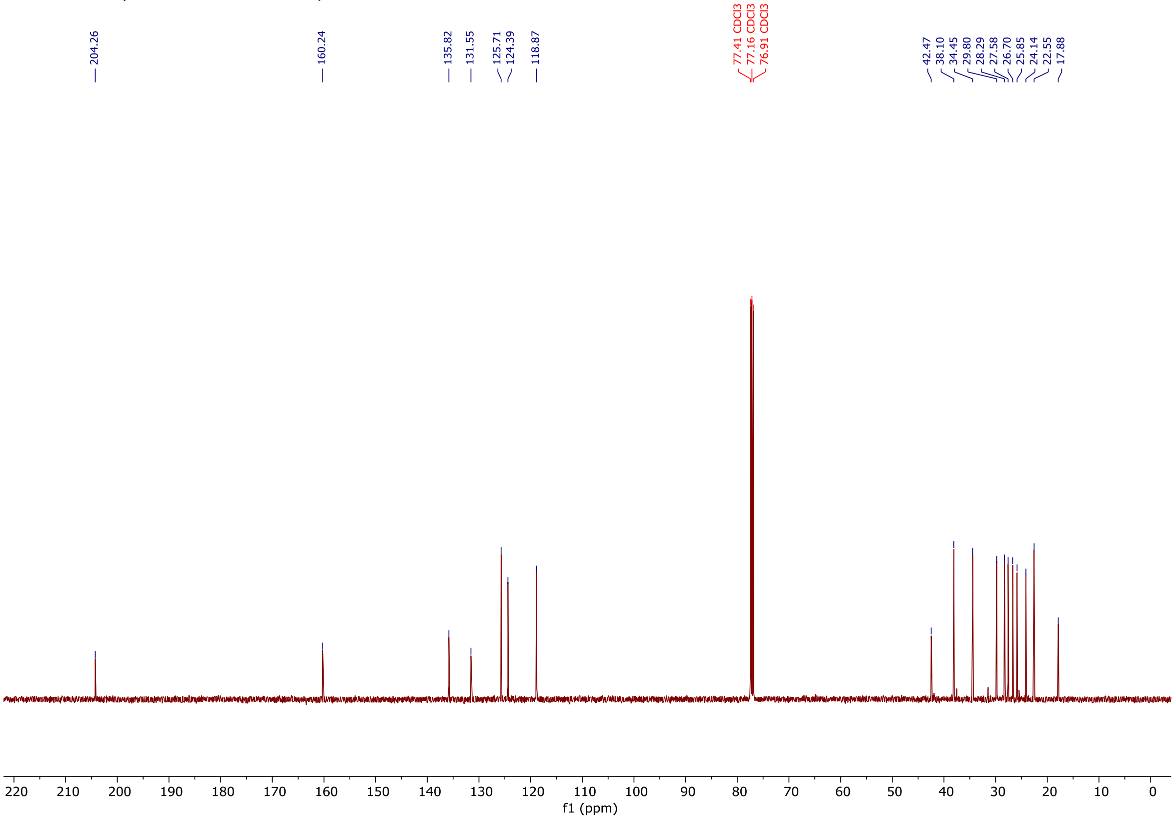

HSQC Spectra of 7l’

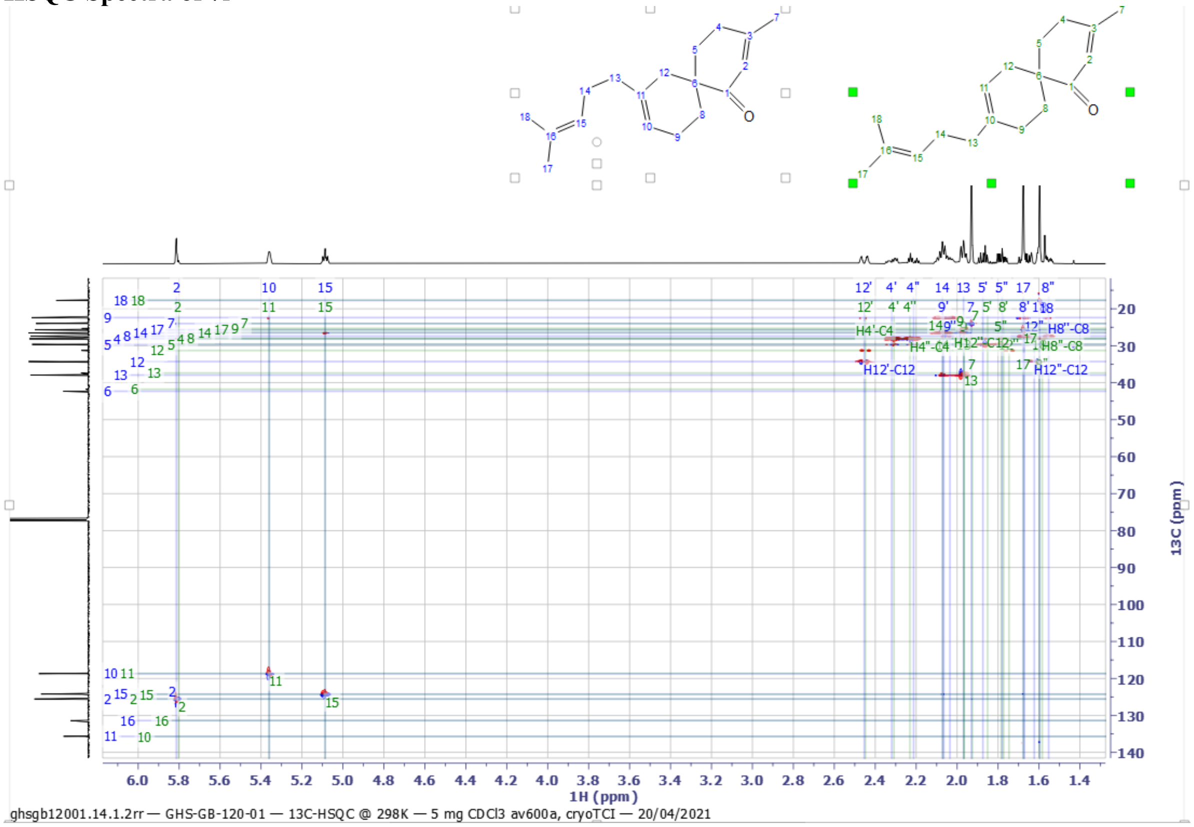

COSY Spectra of 7l’

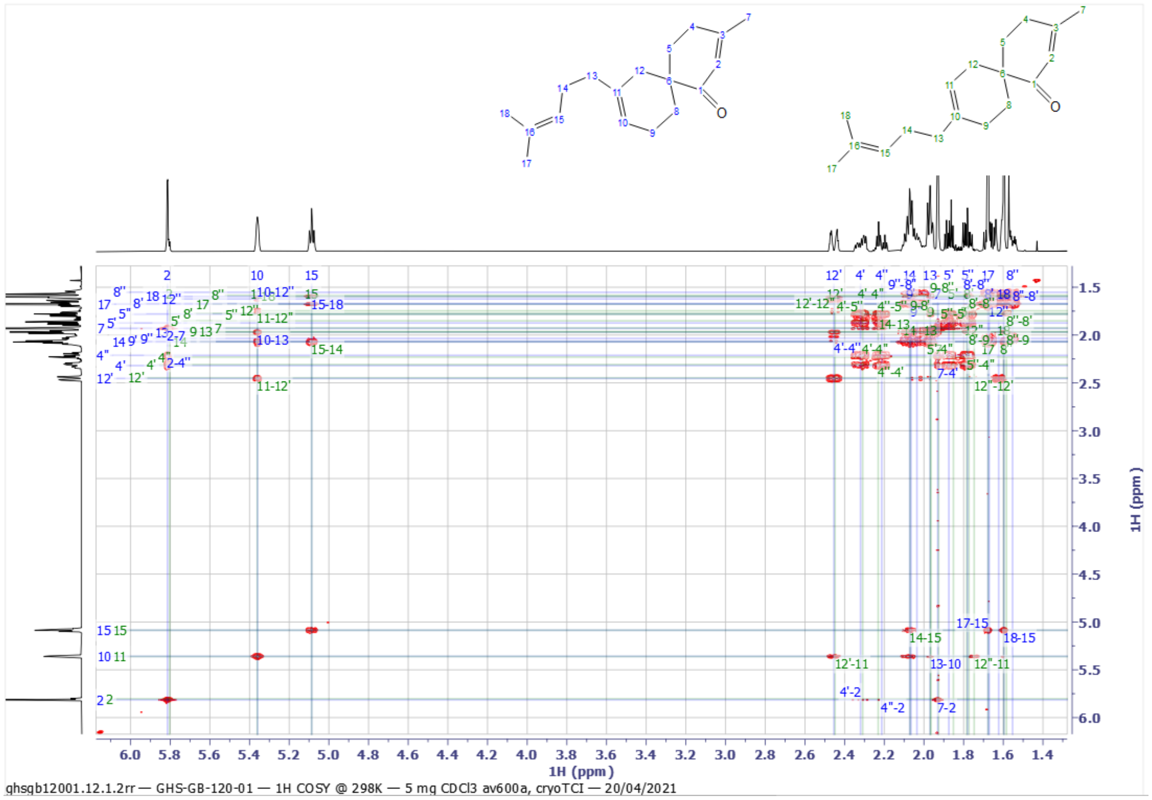

HMBC Spectra of 7l’

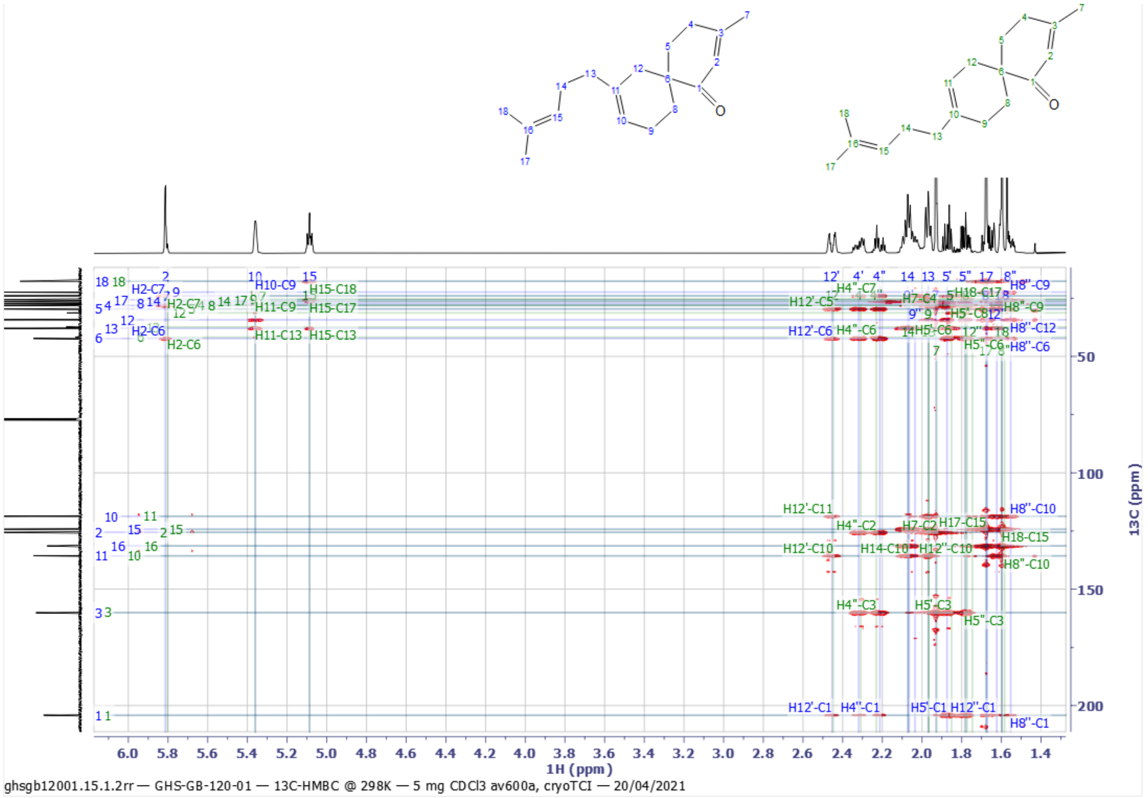

NOESY Spectra of 7l’

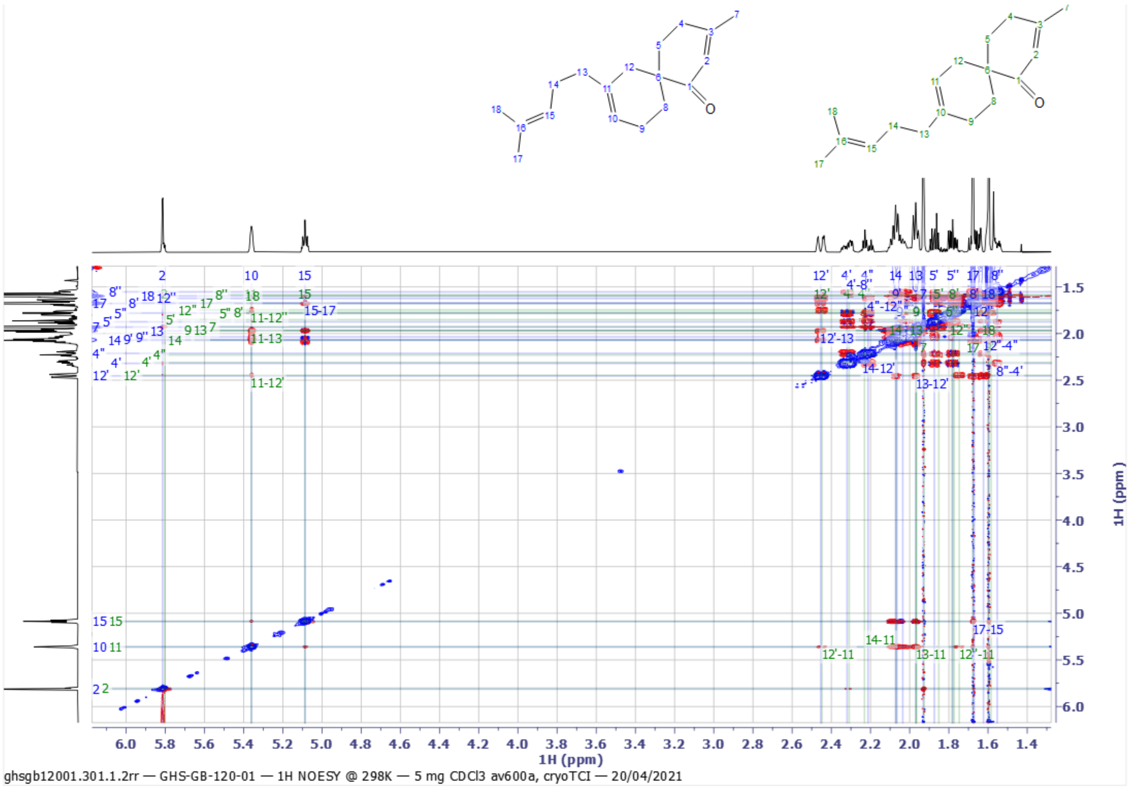

^{1}H NMR (CDCl$_3$, 501 MHz) of (7m')

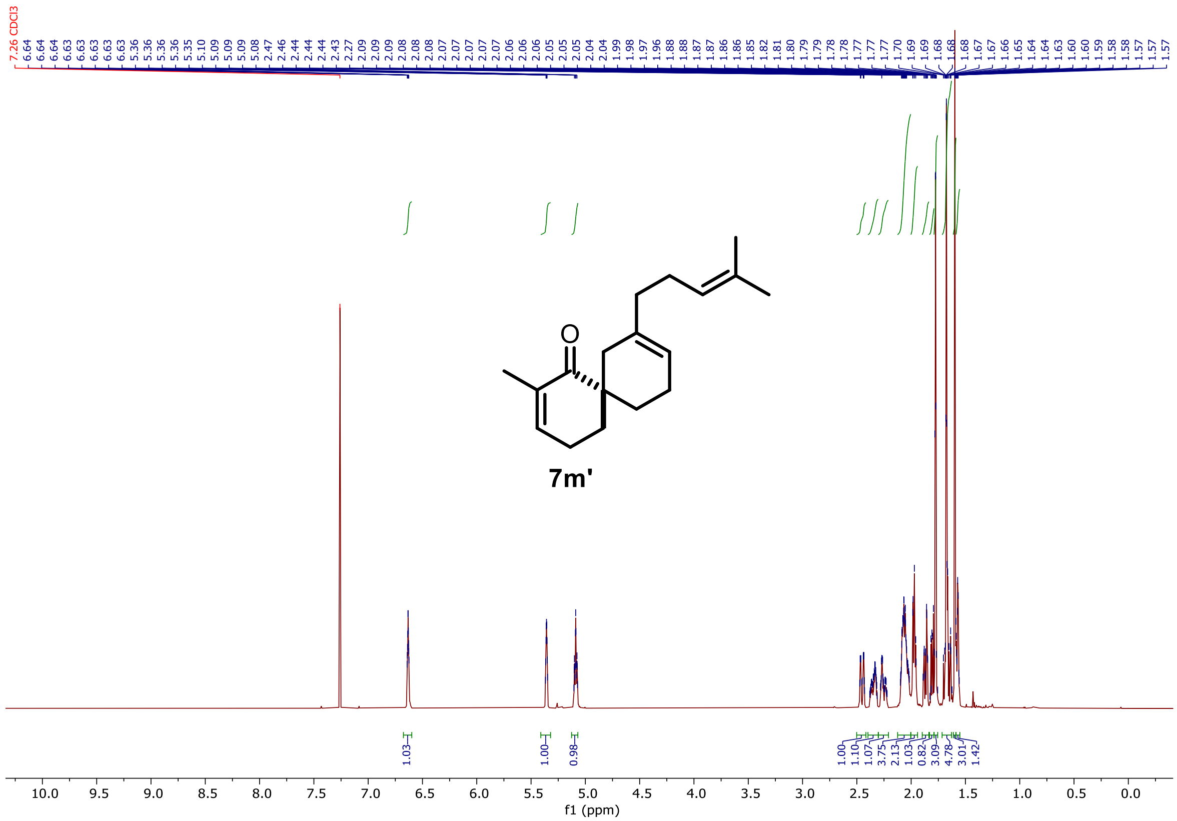

^{13}C NMR (CDCl$_3$, 126 MHz)

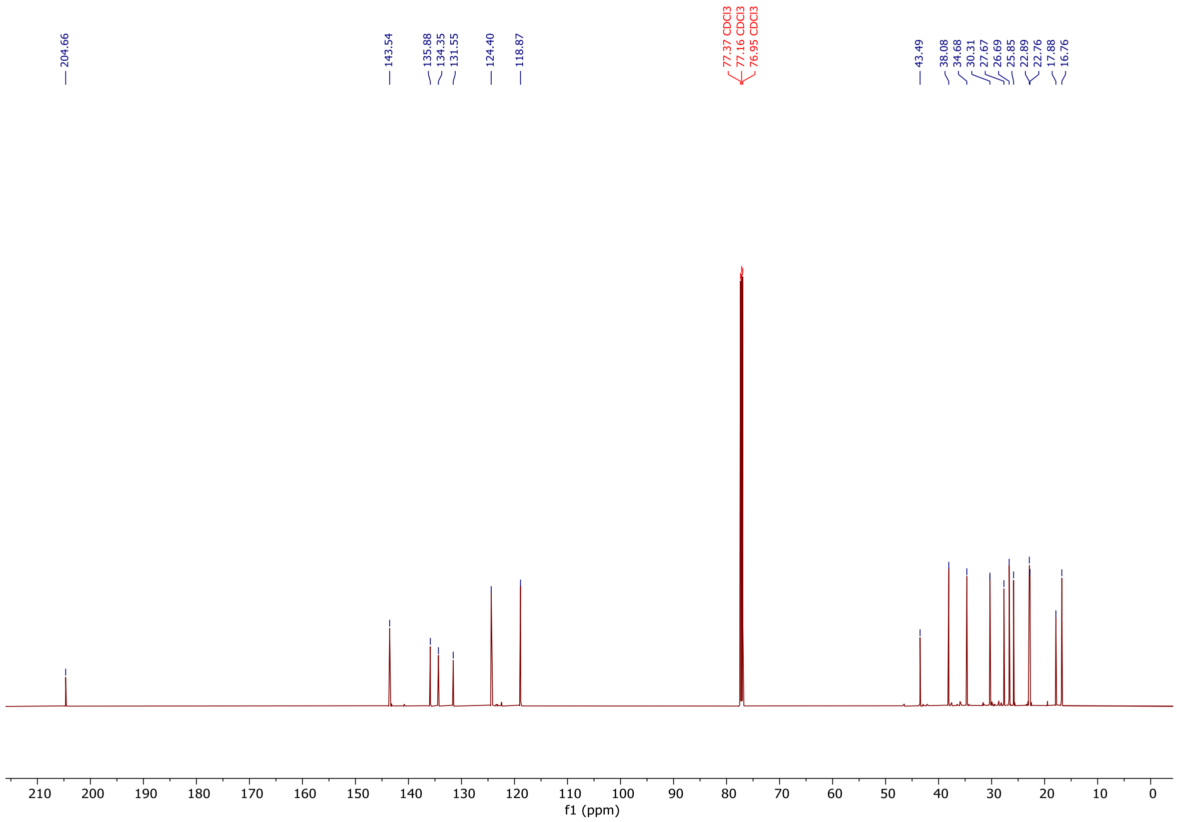

^{1}H NMR (CDCl$_3$, 501 MHz) of (7n')

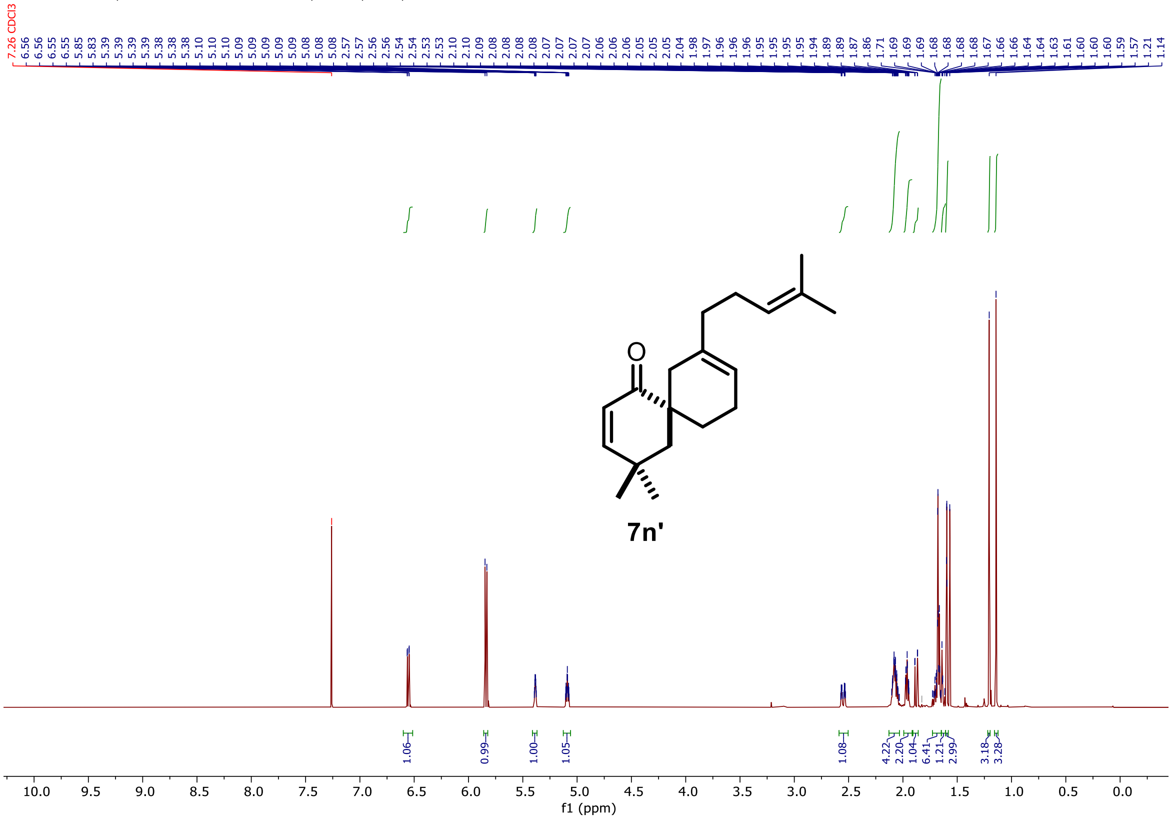

^{13}C NMR (CDCl$_3$, 126 MHz)

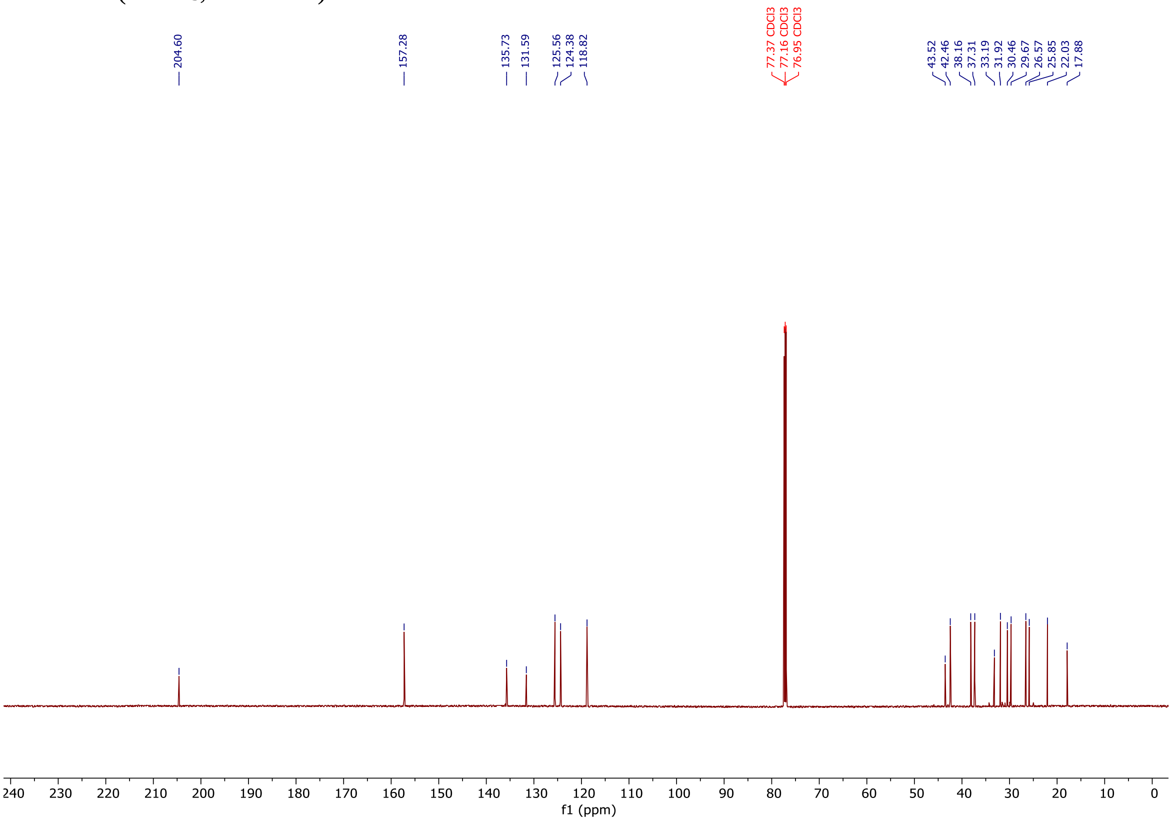

^{1}H NMR ($CDCl_3$, 600 MHz) of (7o’)

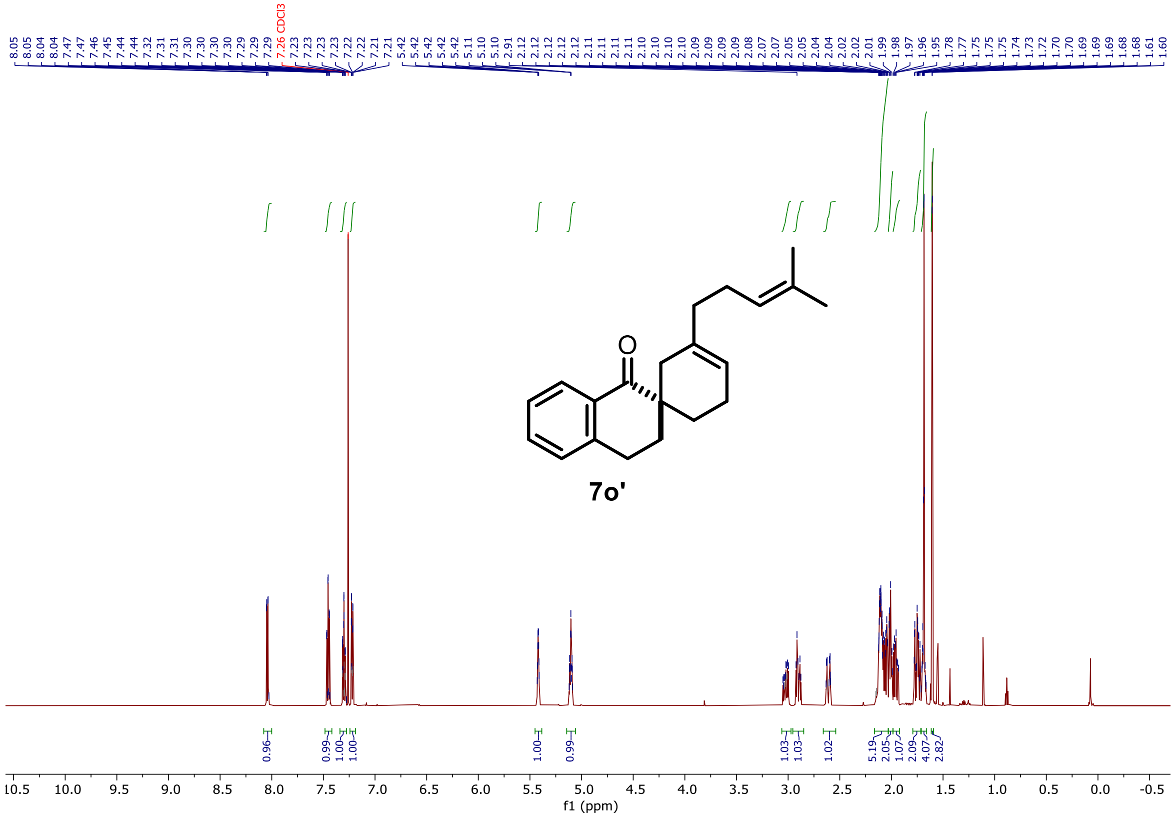

^{13}C NMR ($CDCl_3$, 151 MHz)

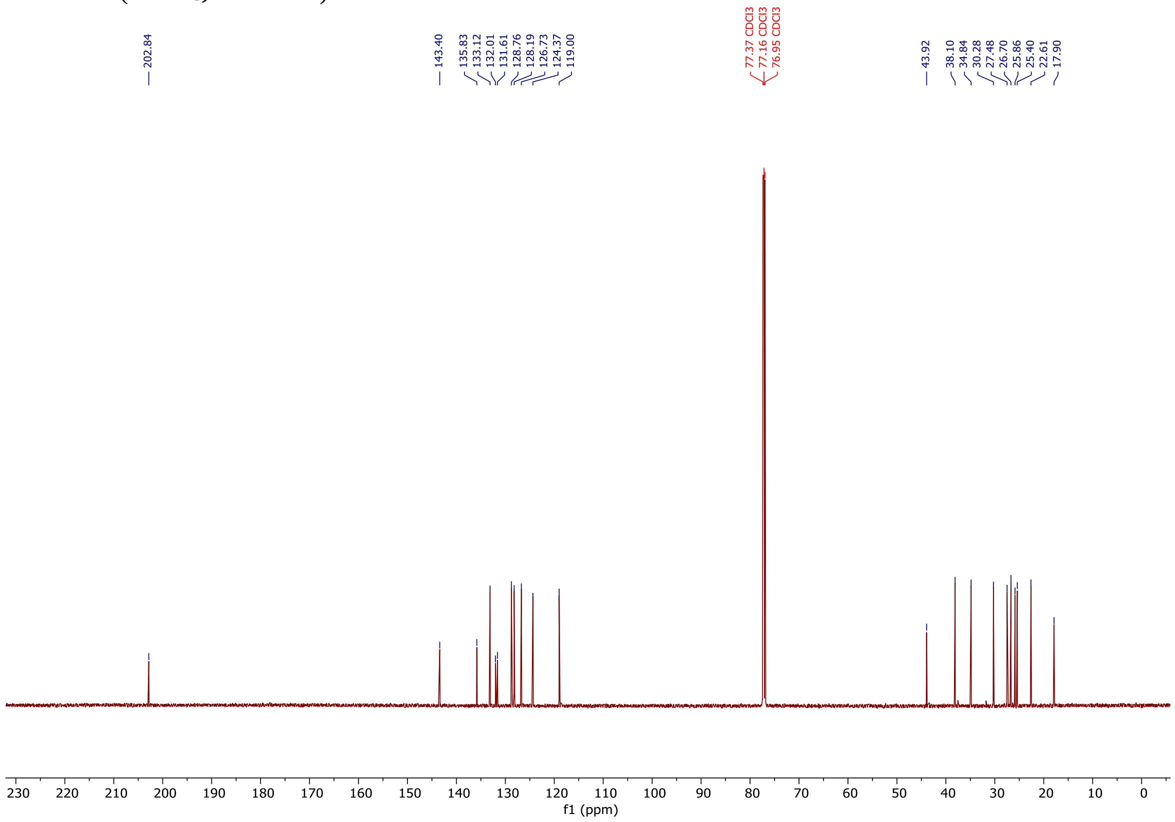

^{1}H NMR ($CDCl_3$, 501 MHz) of (7p)

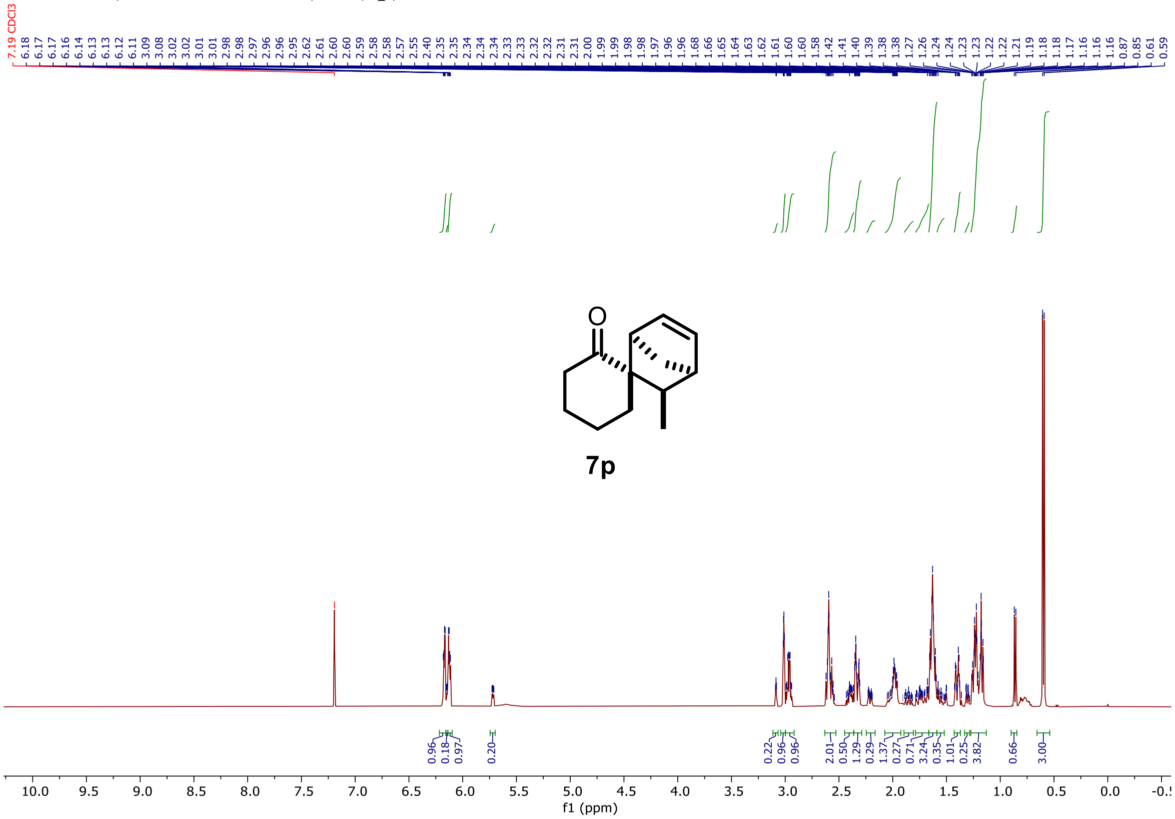

^{13}C NMR ($CDCl_3$, 126 MHz)

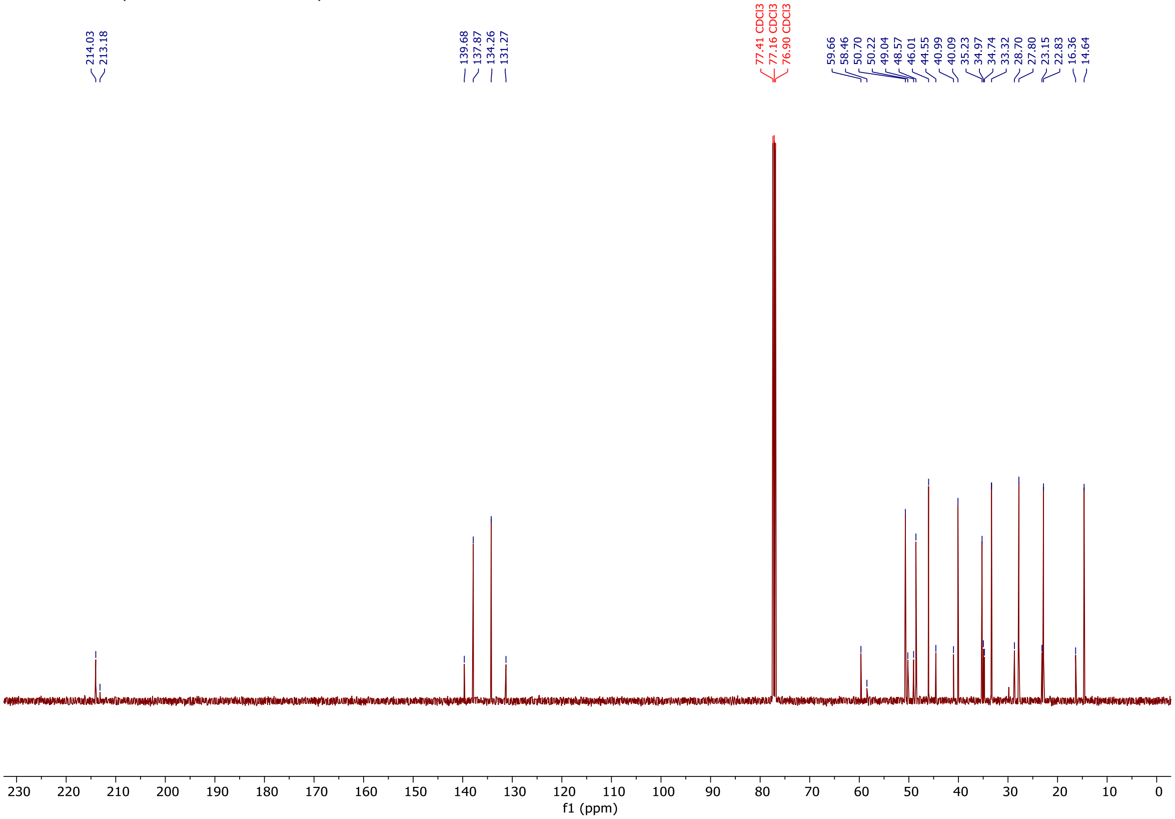

^{1}H NMR ($CDCl_3$, 501 MHz) of (7q)

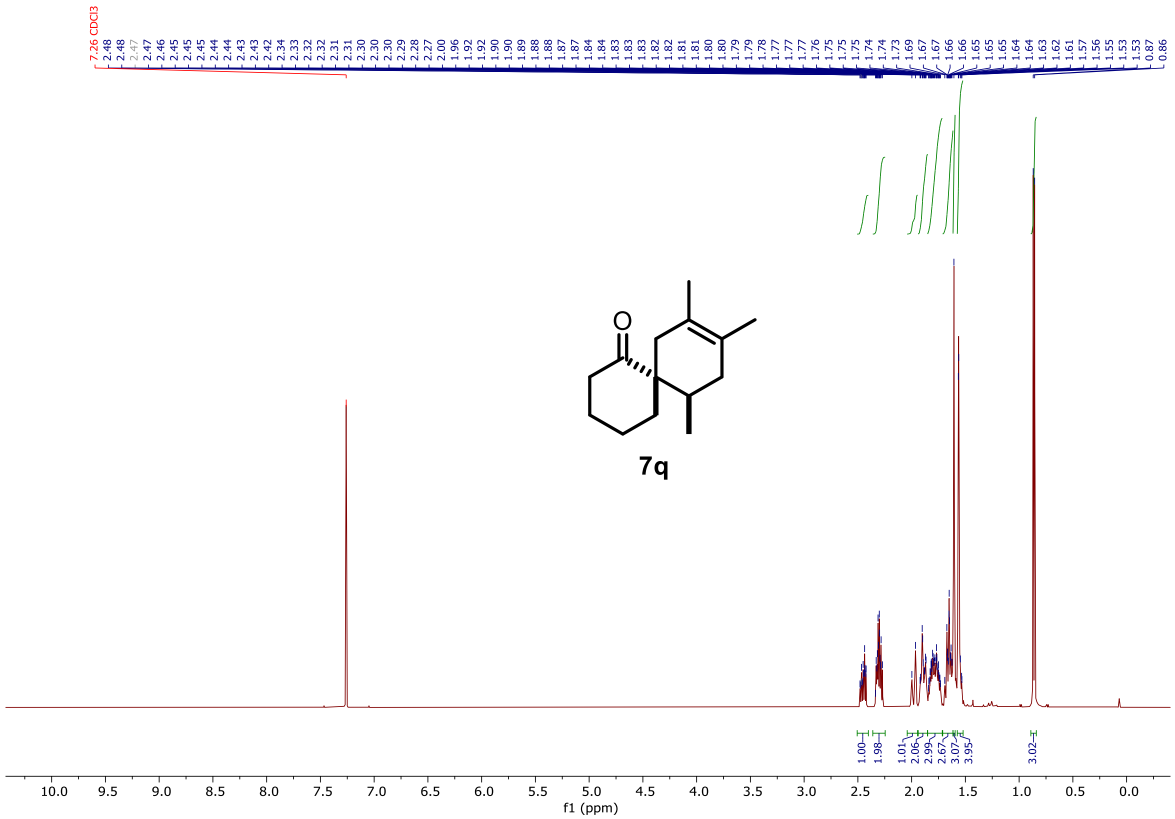

^{13}C NMR ($CDCl_3$, 126 MHz)

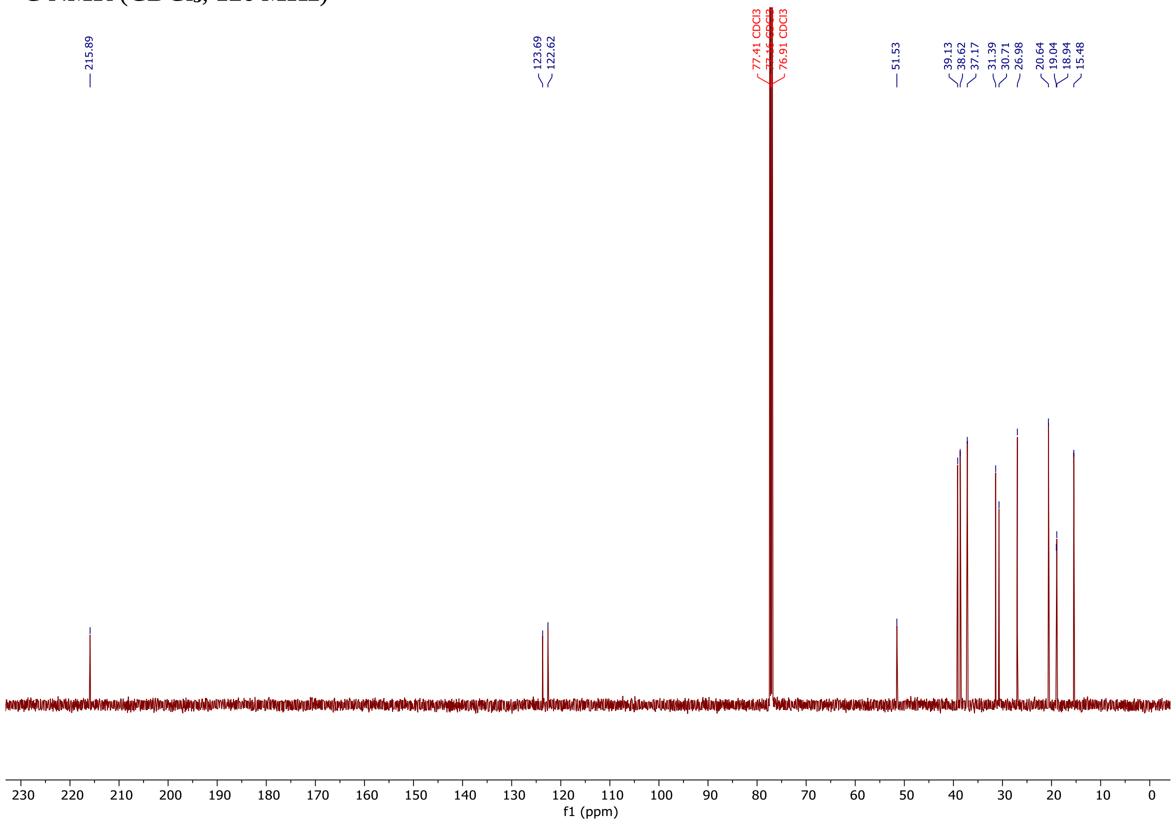

^{1}H NMR ($CDCl_3$, 501 MHz) of (7r)

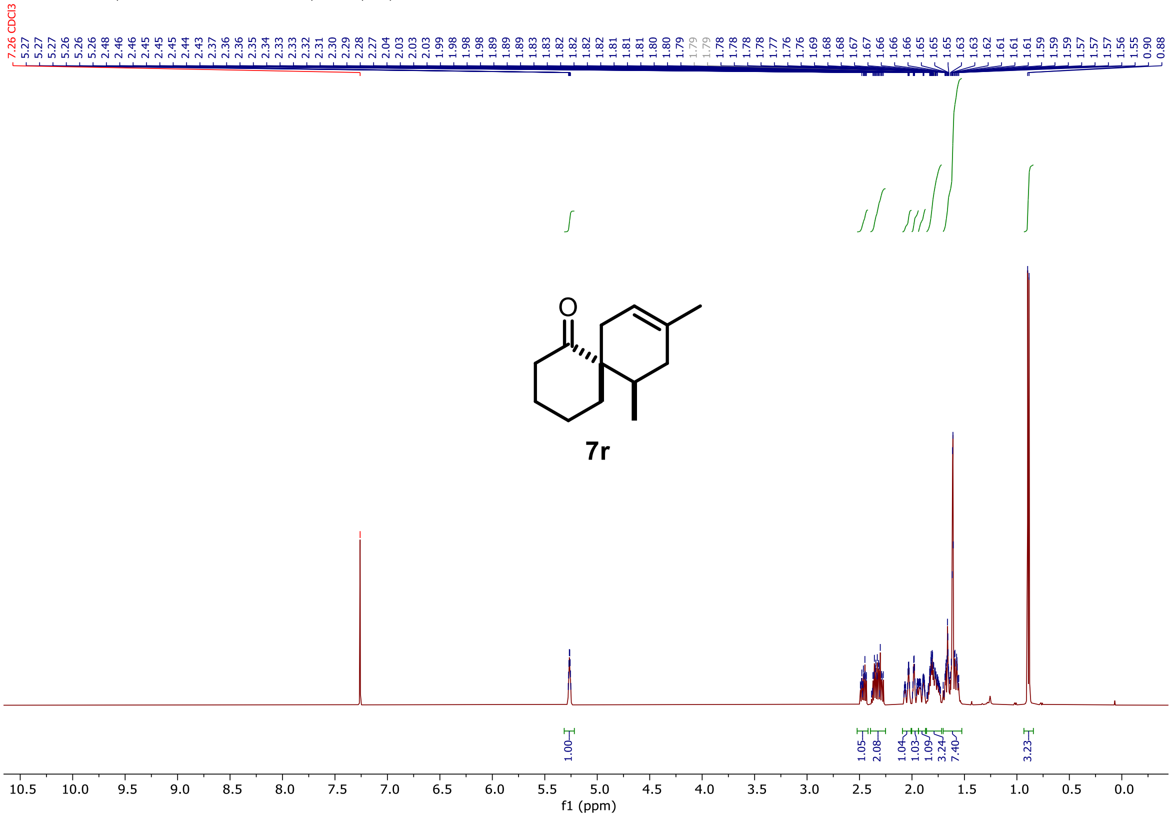

^{13}C NMR ($CDCl_3$, 126 MHz)

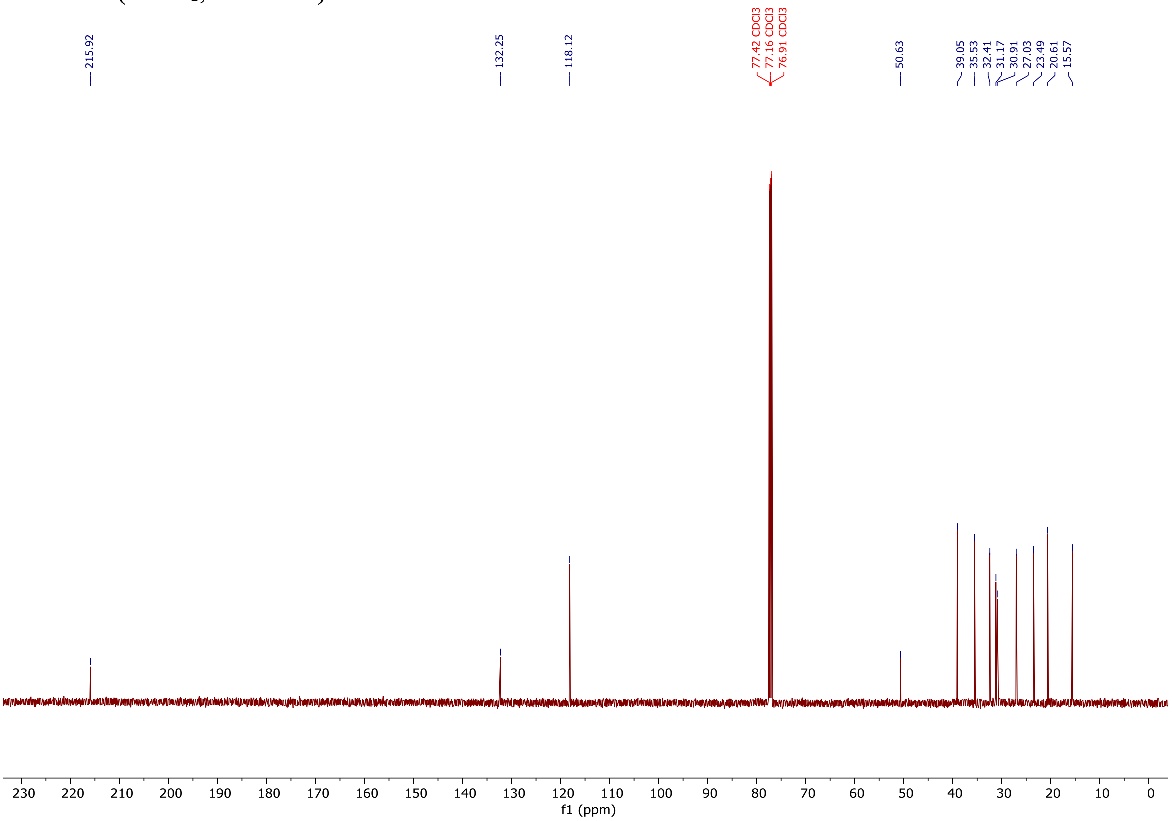

^{1}H NMR ($CDCl_3$, 501MHz) of (13a)

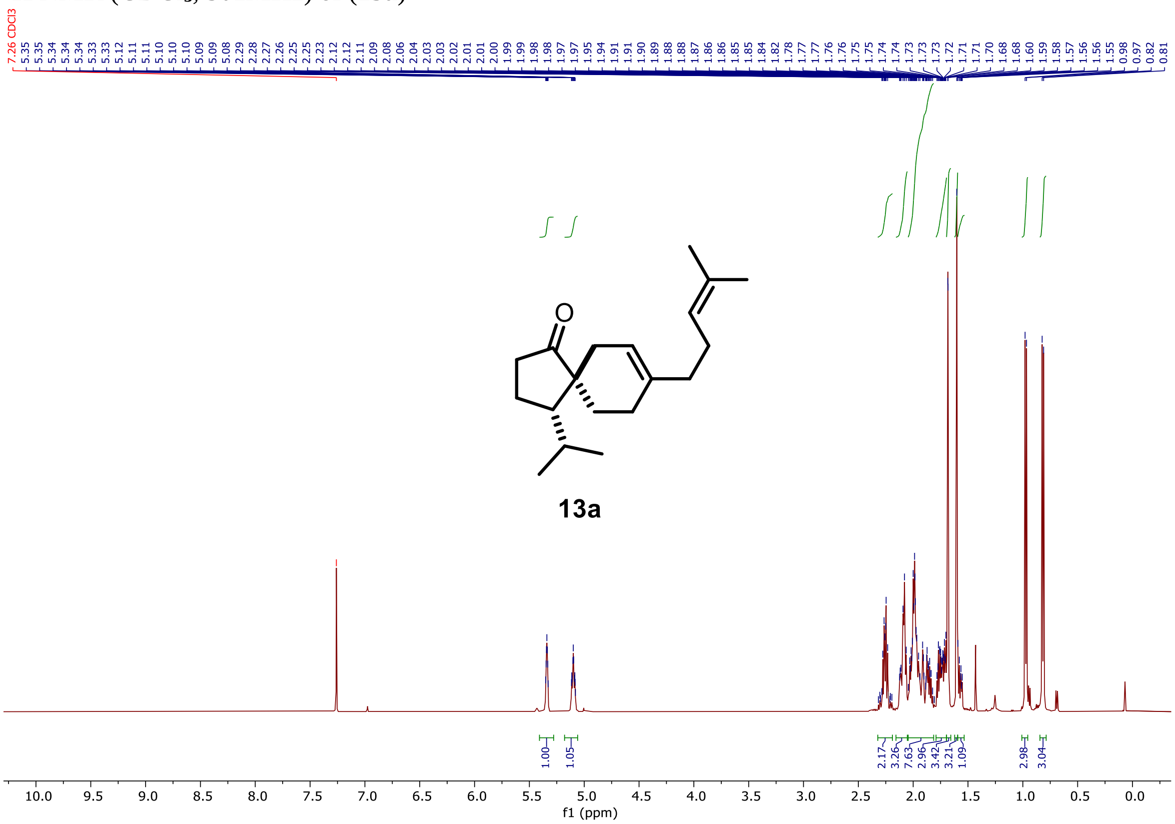

13a

^{13}C NMR ($CDCl_3$, 126 MHz)

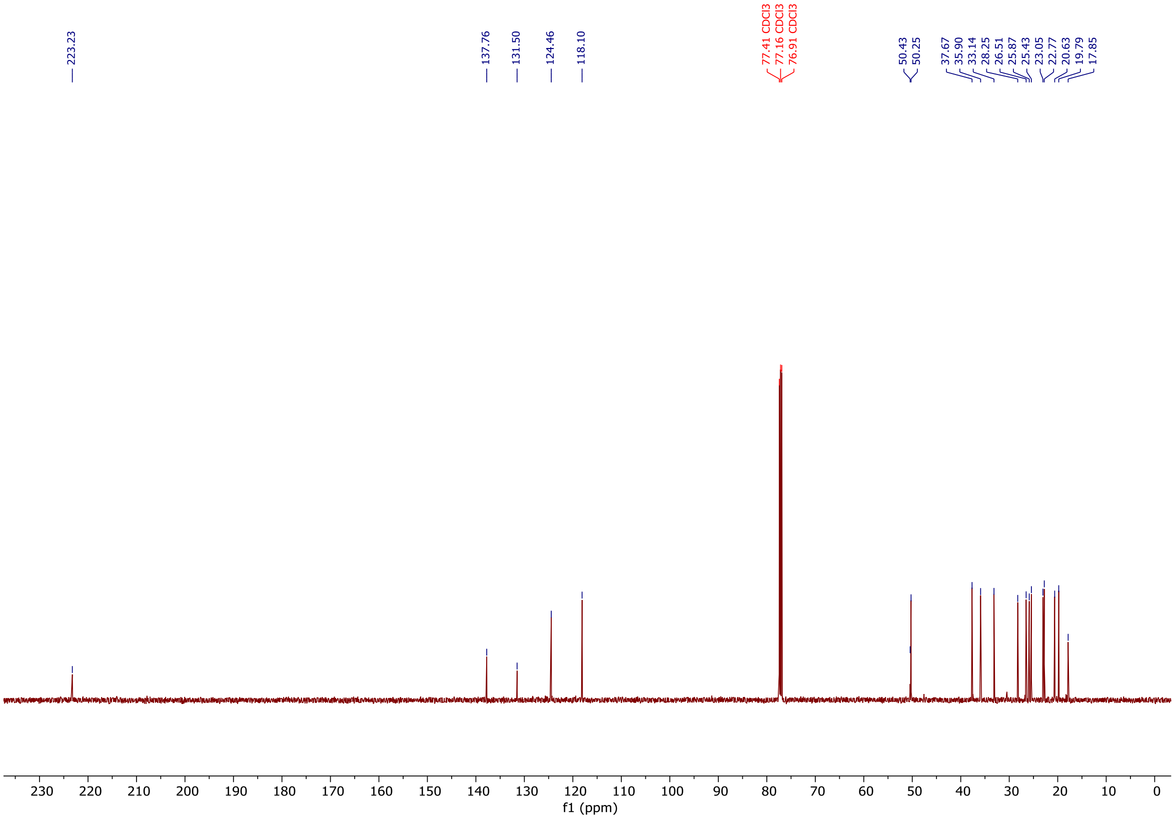

^{1}H NMR (CDCl$_3$, 501 MHz) of (+)-12

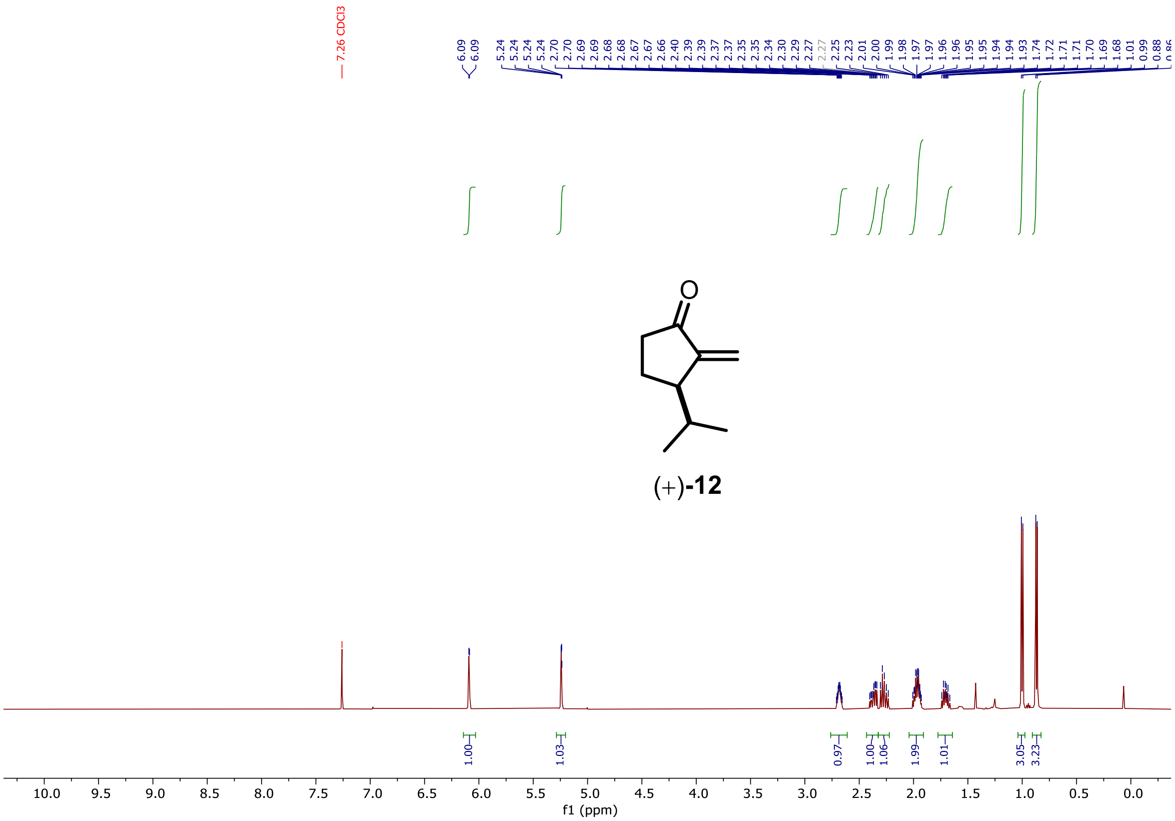

^{13}C NMR (CDCl$_3$, 126 MHz)

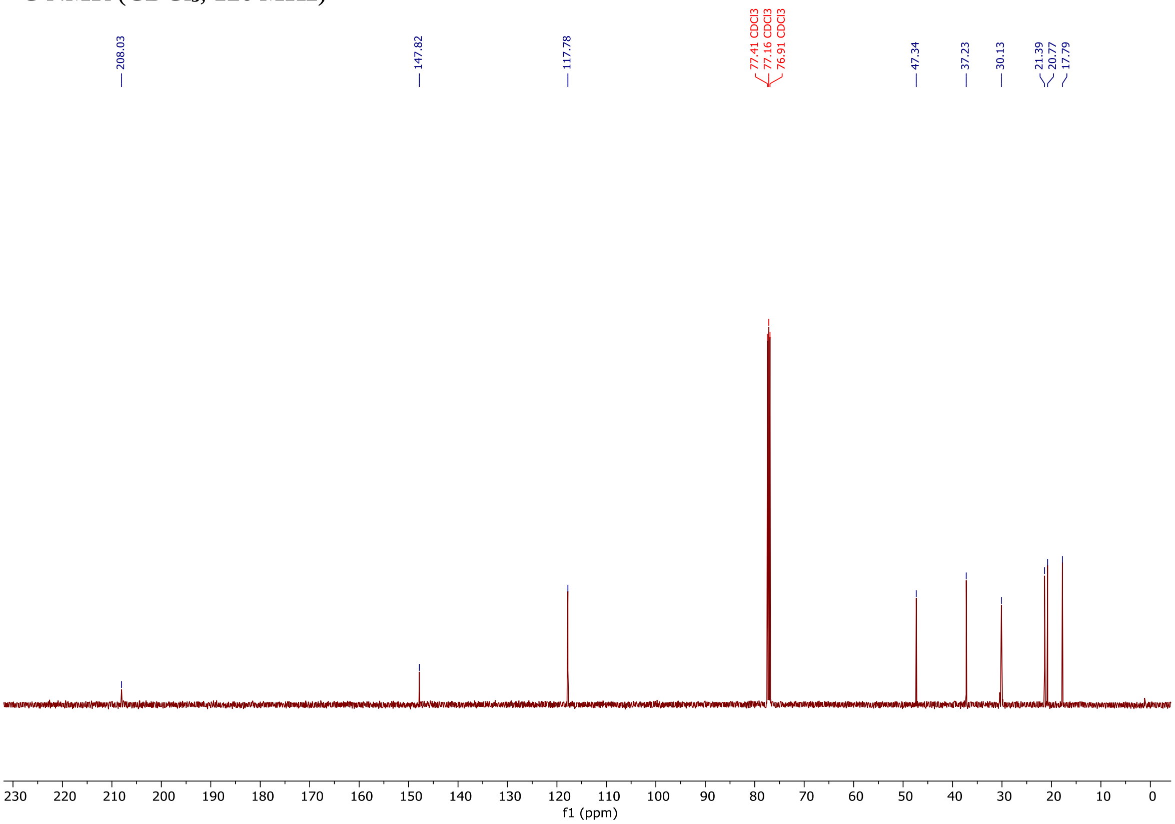

^{1}H NMR ($CDCl_3$, 501MHz) of (13b)

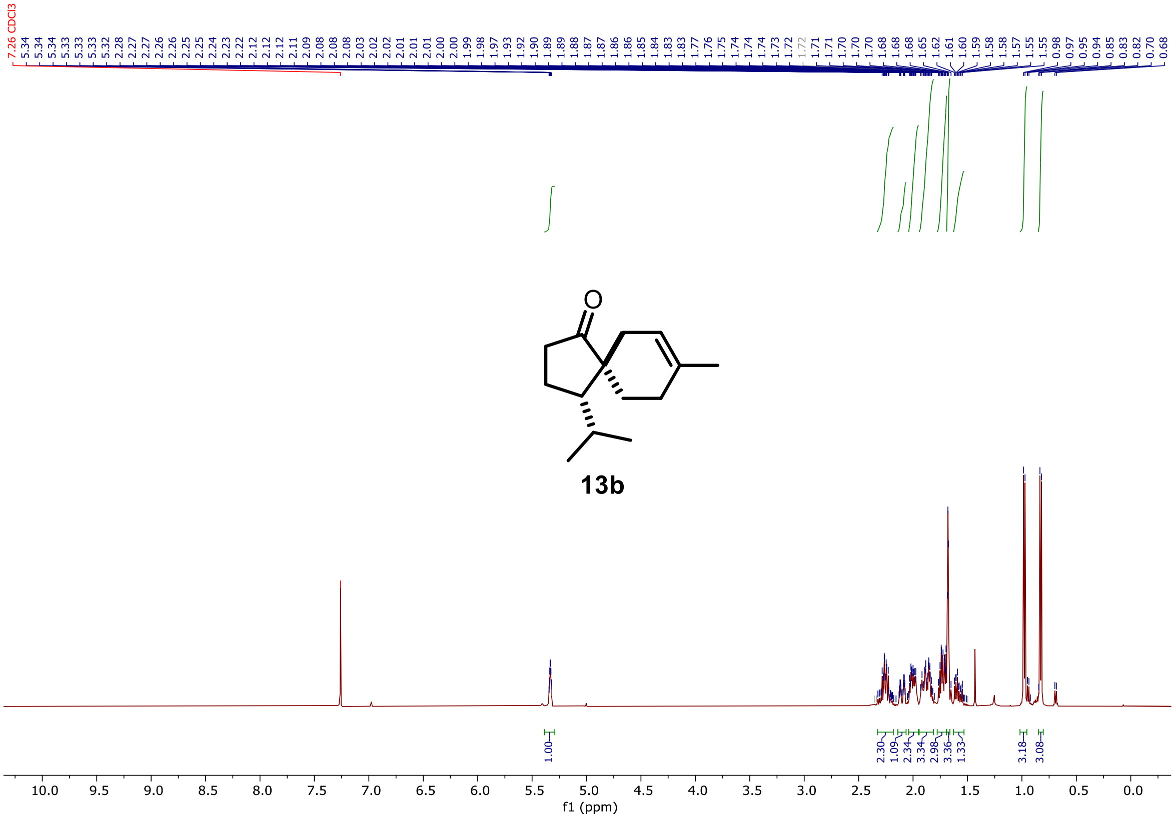

^{13}C NMR ($CDCl_3$, 126 MHz)

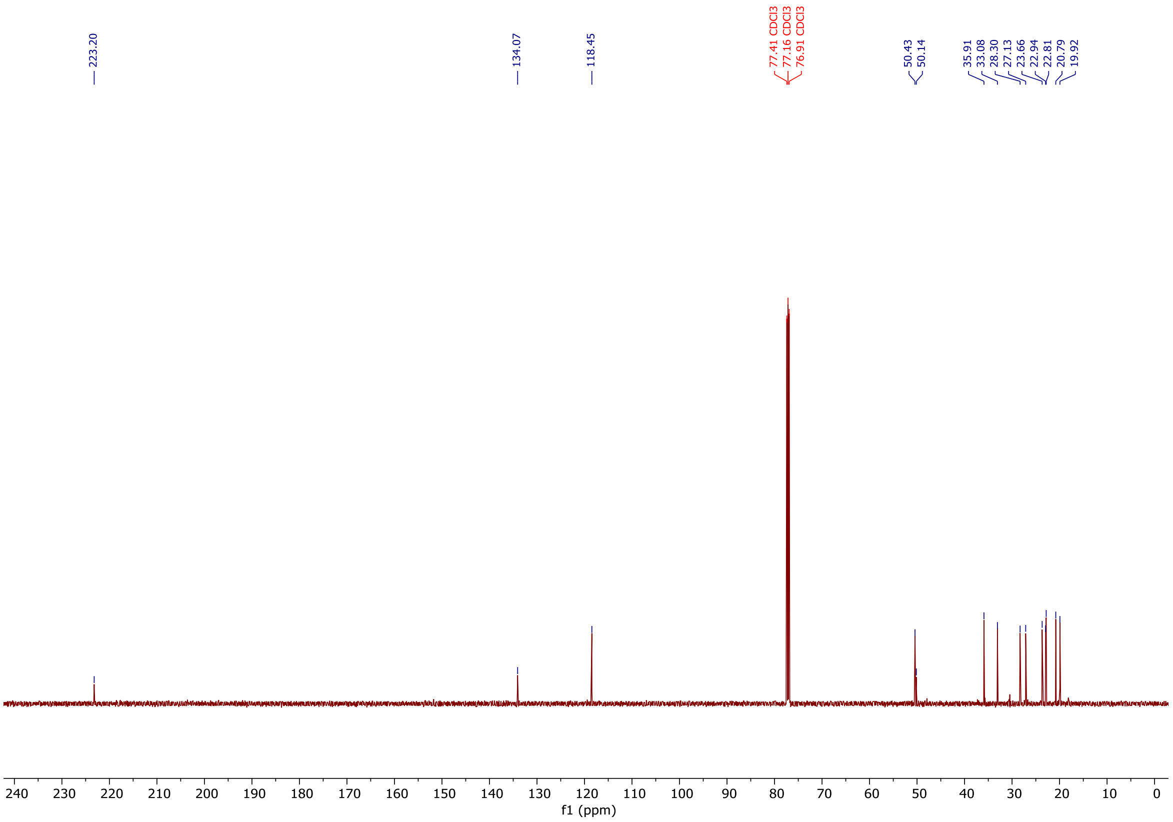

^{1}H NMR ($CDCl_3$, 501 MHz) of (14)

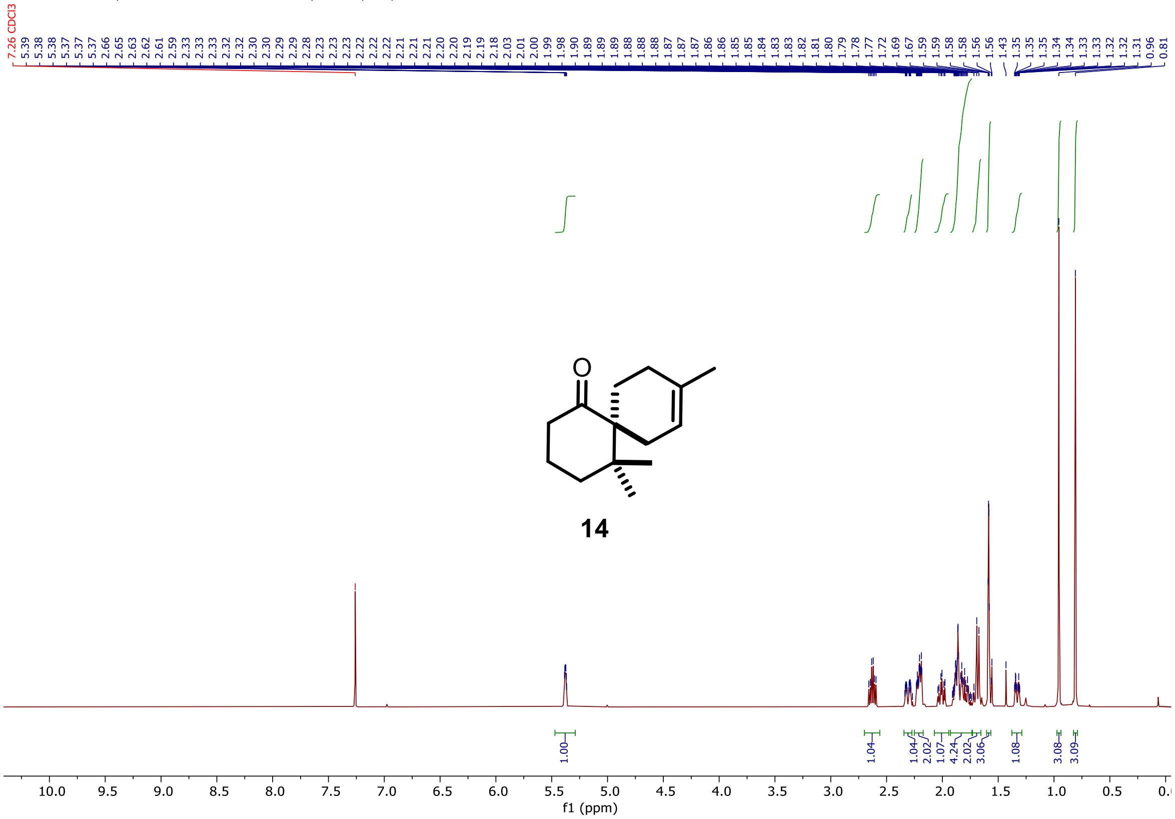

^{13}C NMR ($CDCl_3$, 126 MHz)

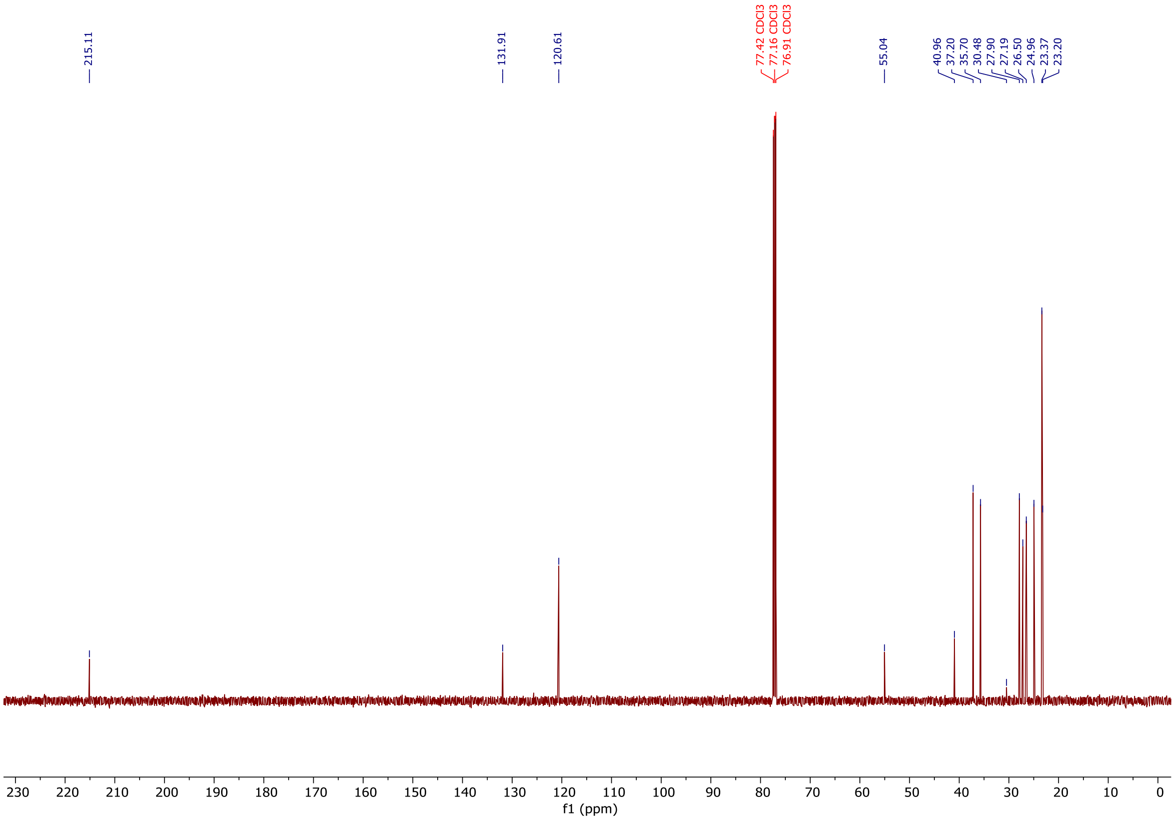

^{1}H NMR (CDCl3, 501 MHz) of (4b)

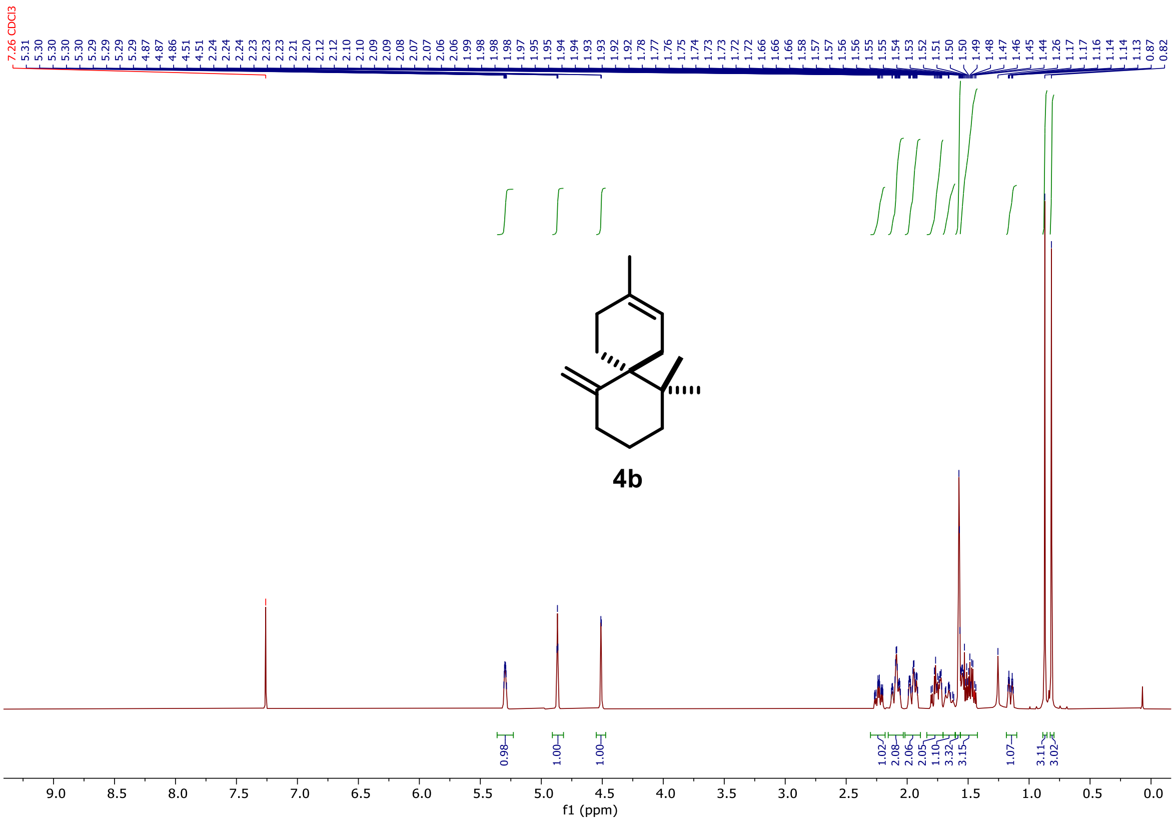

^{13}C NMR (CDCl3, 126MHz)

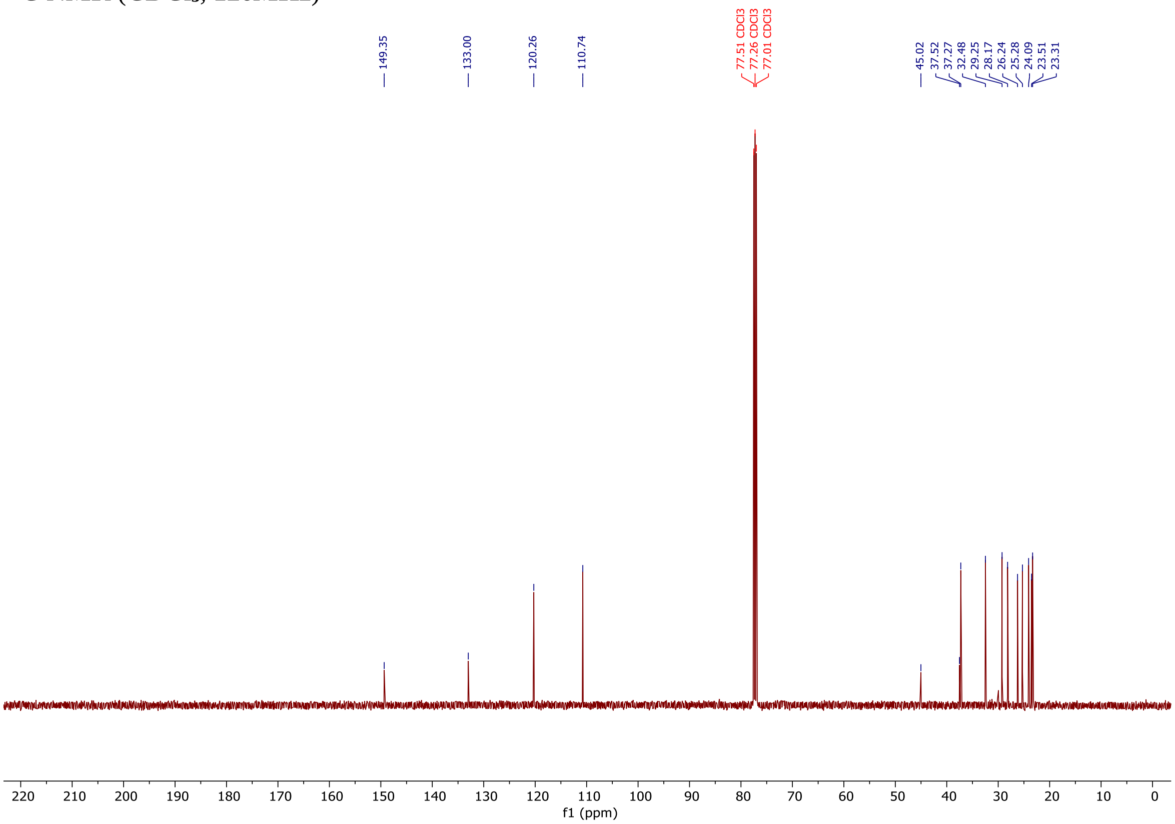

^{1}H NMR ($CDCl_3$, 501 MHz) of (15)

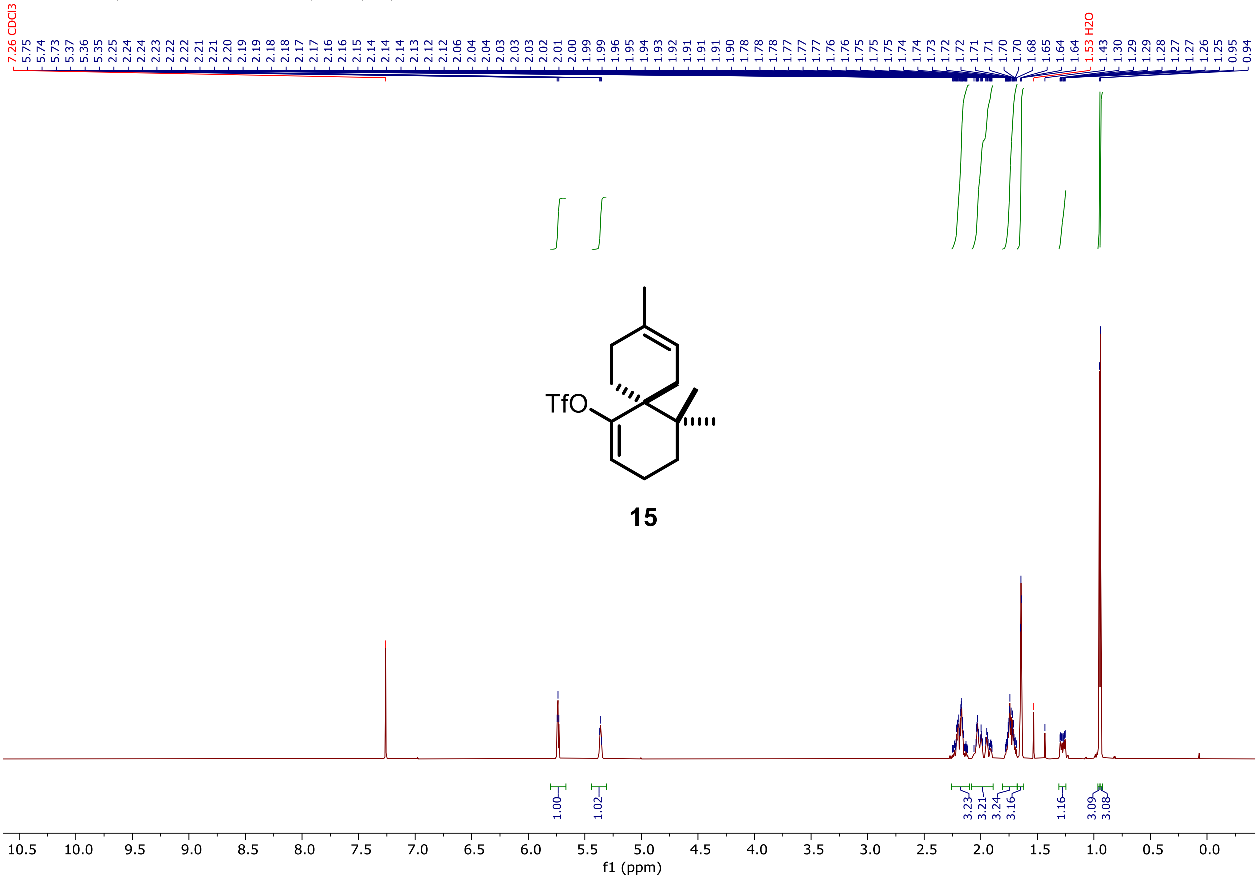

^{13}C NMR ($CDCl_3$, 126 MHz)

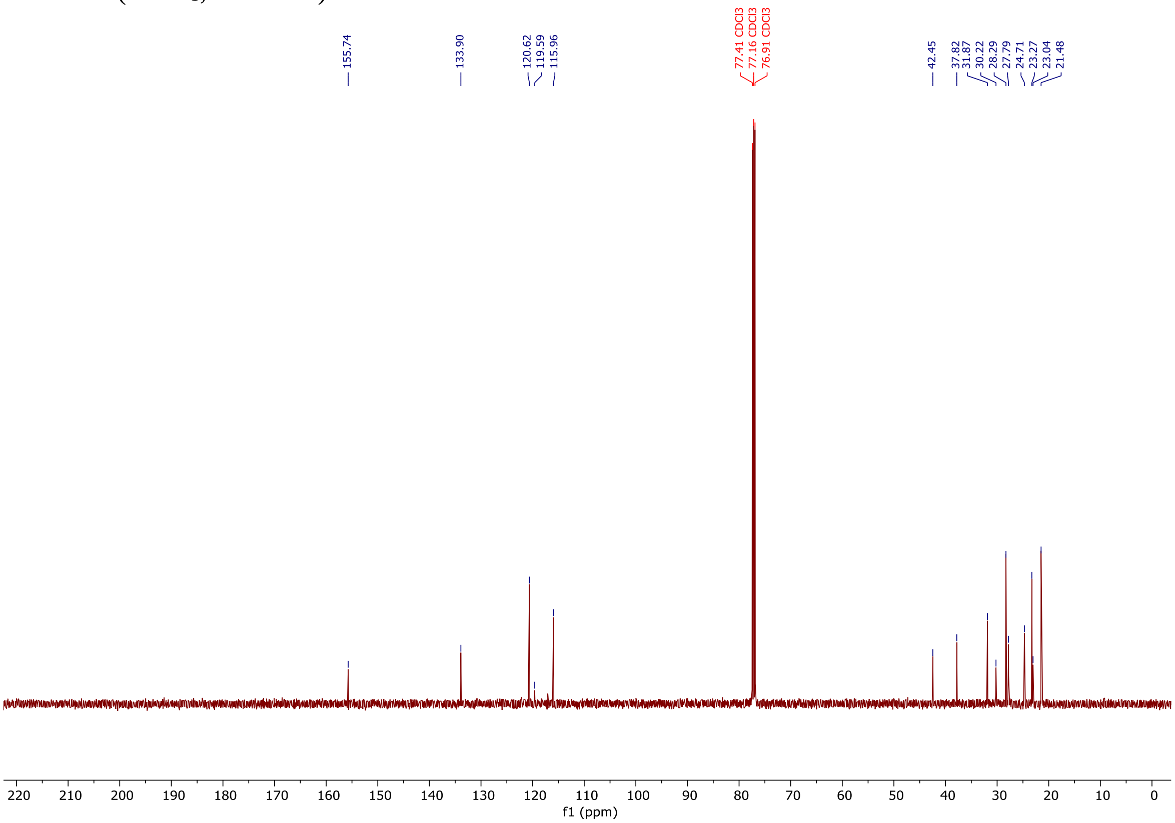

^{19}F NMR ($CDCl_3$, 471 MHz)

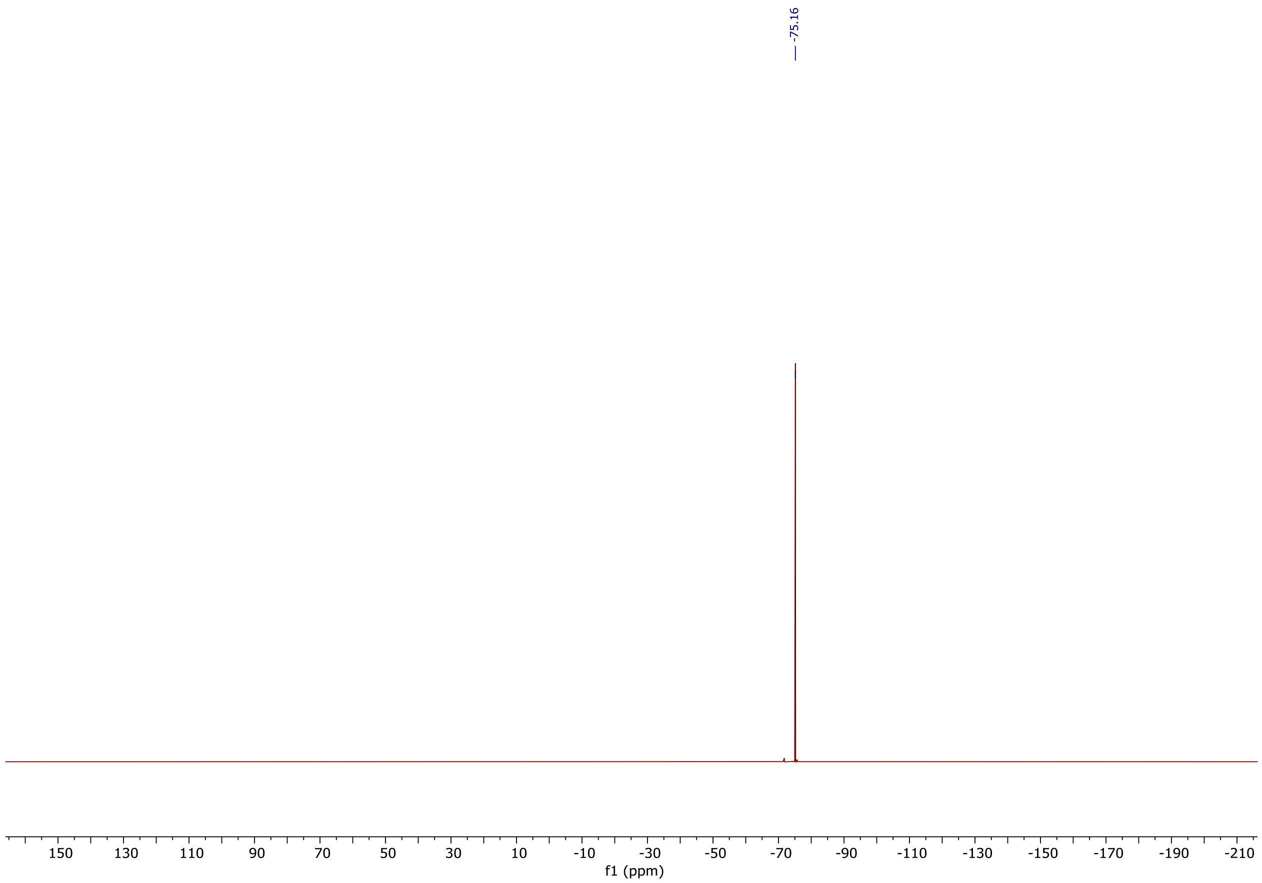

^{1}H NMR ($CDCl_3$, 501 MHz) of (4a)

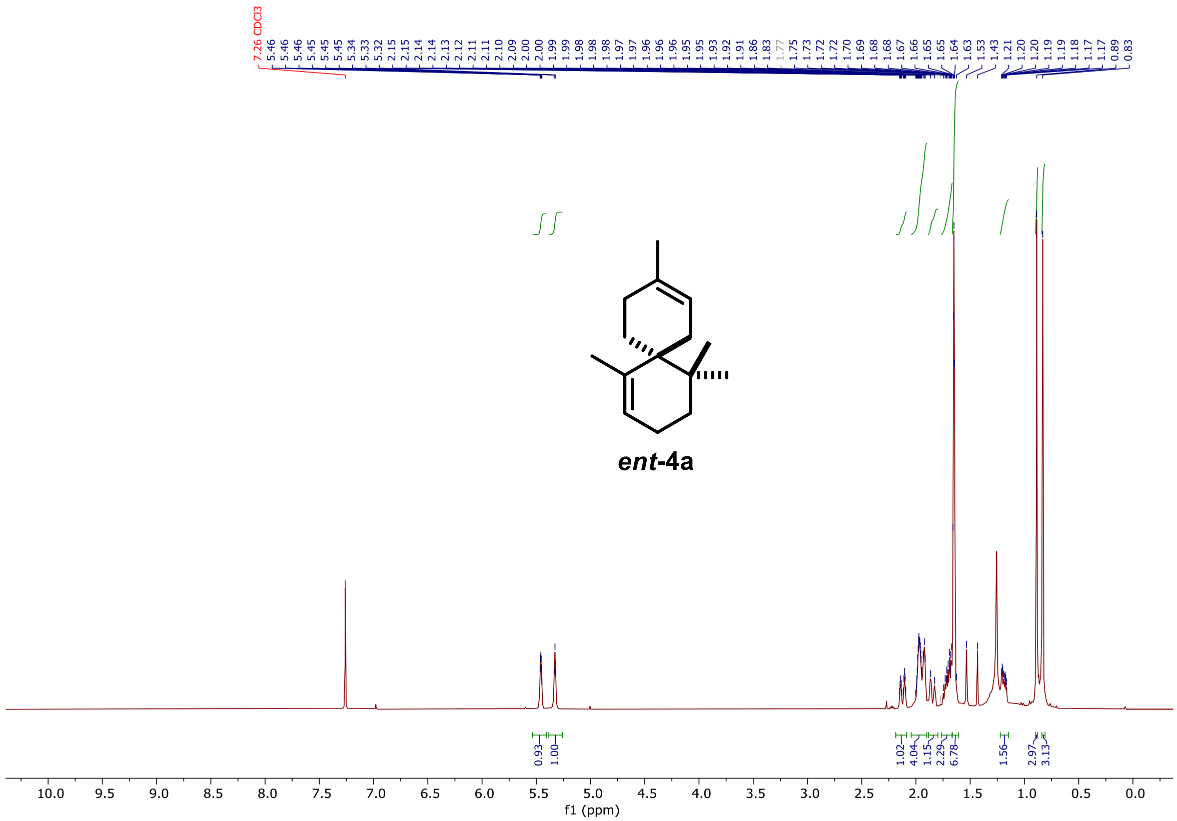

^{13}C NMR ($CDCl_3$, 126 MHz)

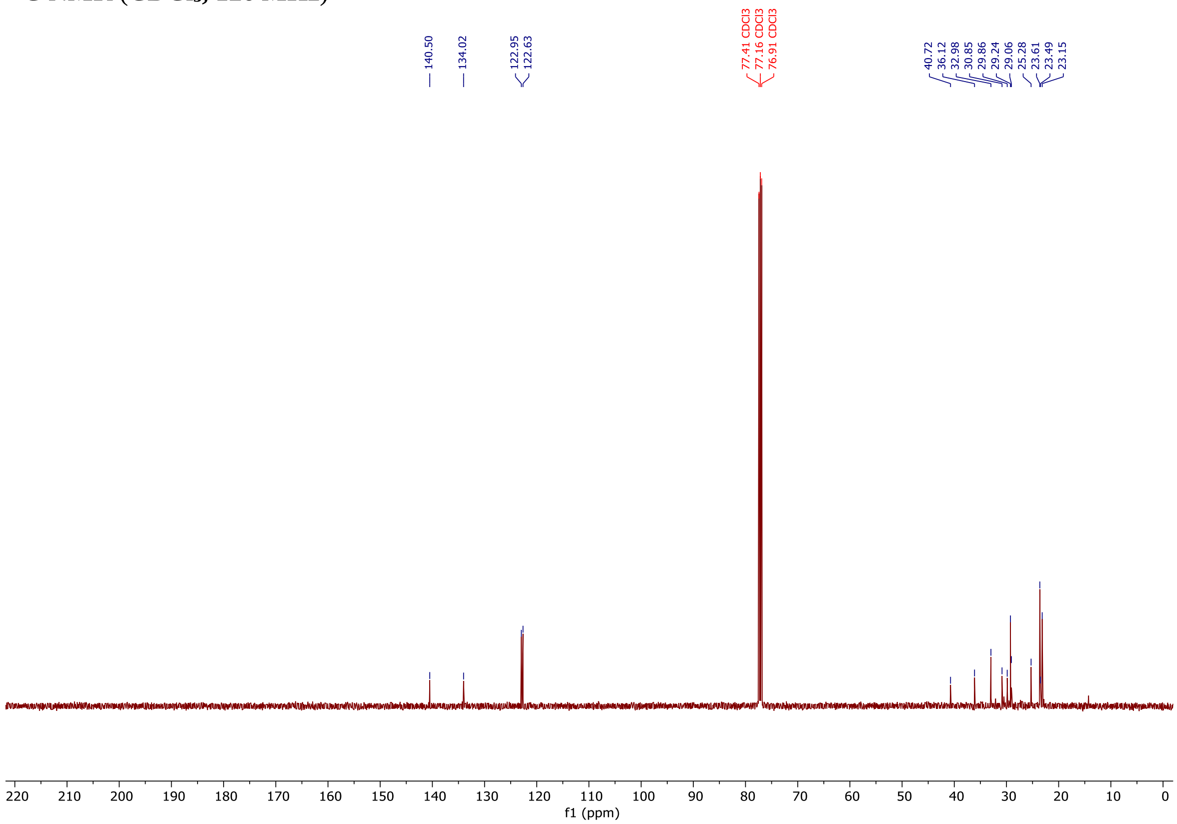

^{1}H NMR ($CDCl_3$, 501 MHz) of (16)

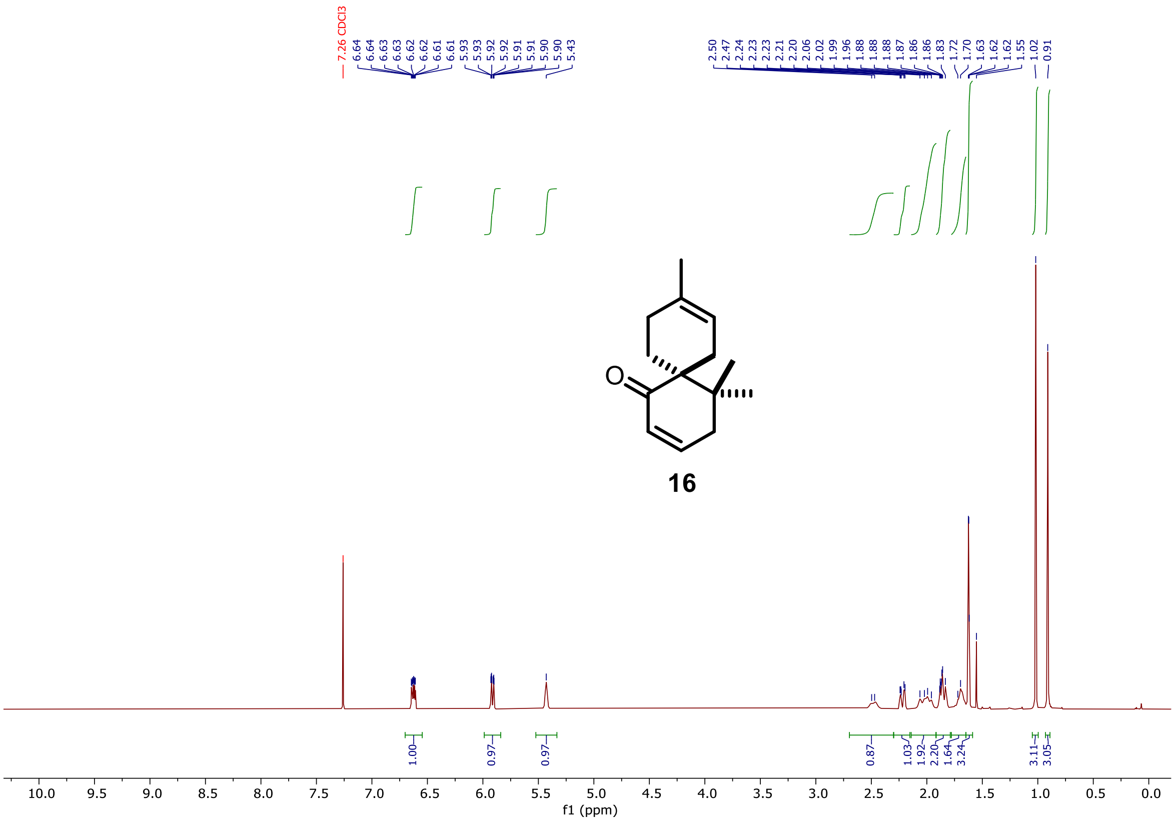

^{13}C NMR ($CDCl_3$, 151 MHz)

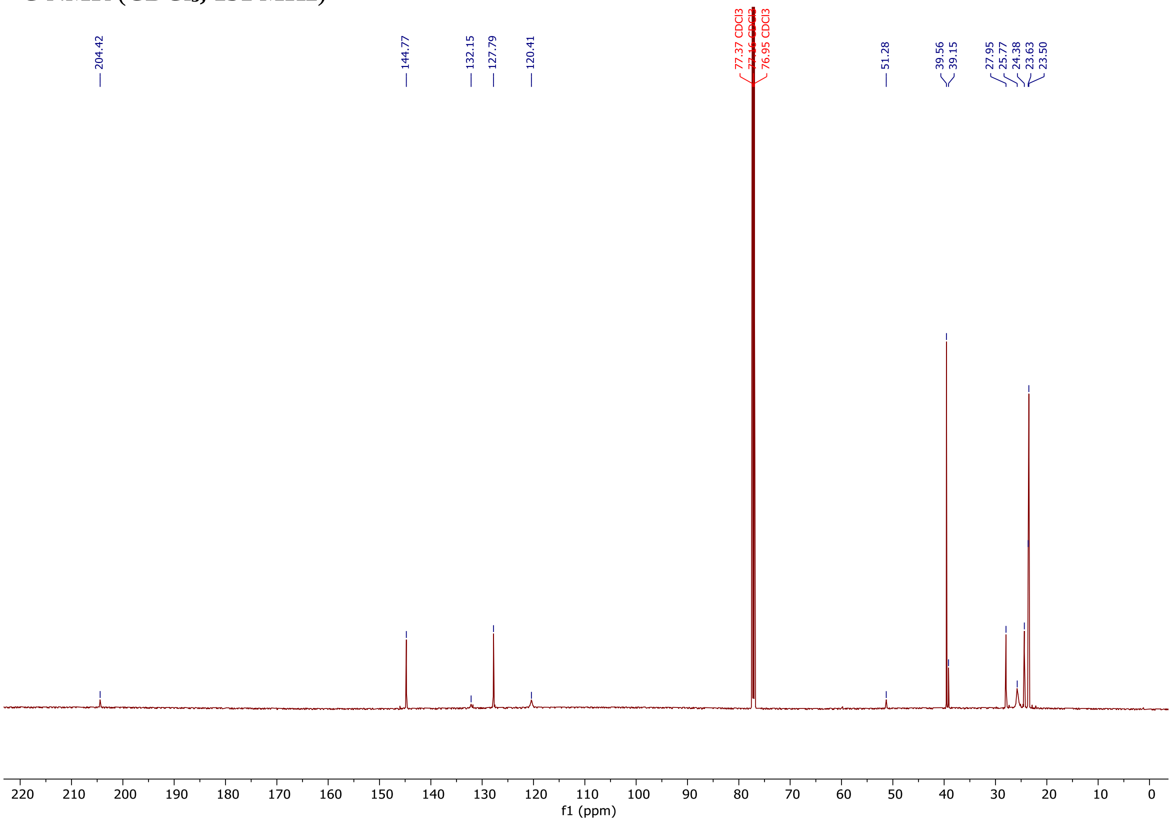

^{1}H NMR ($CDCl_3$, 501 MHz) of (*ent*-4c)

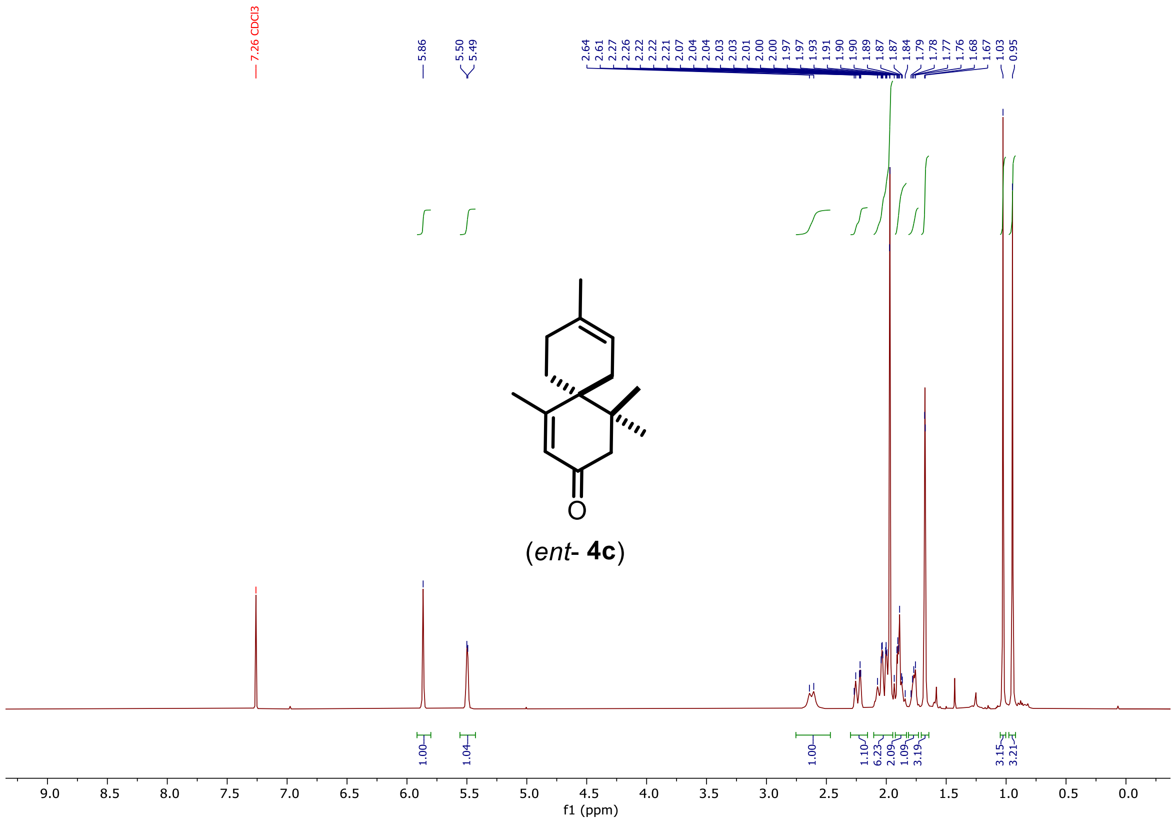

^{13}C NMR ($CDCl_3$, 126 MHz)

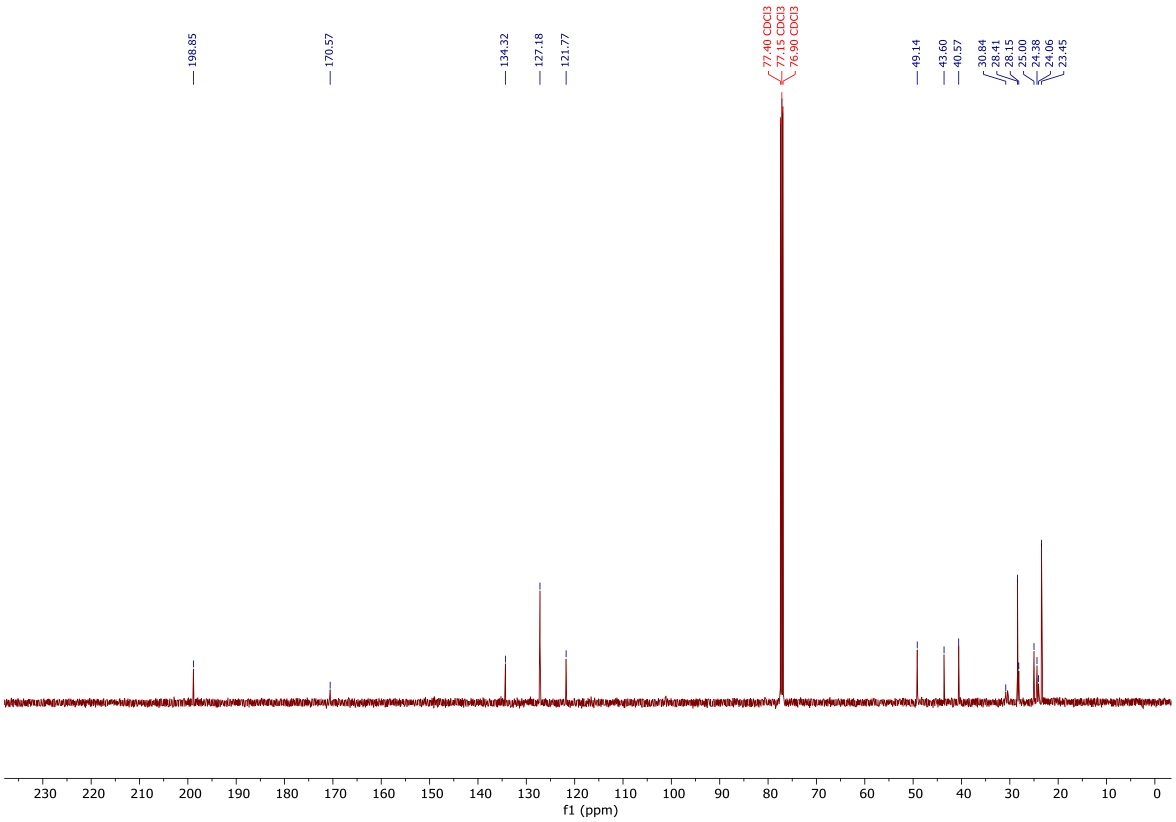

^{1}H NMR (CDCl$_3$, 600 MHz) of (27a)

(spectra contain in separable mixture of diastereomers and regioisomers)

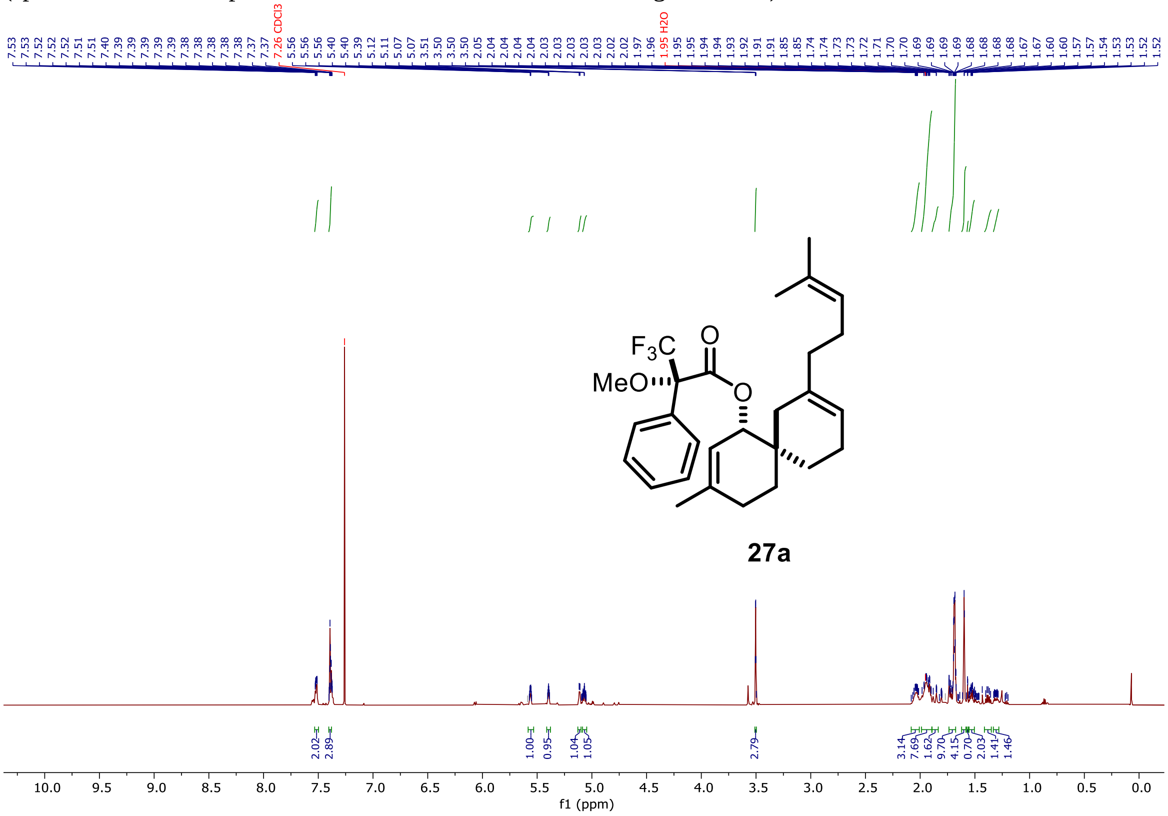

^{13}C NMR (CDCl$_3$, 151 MHz) of (27a)

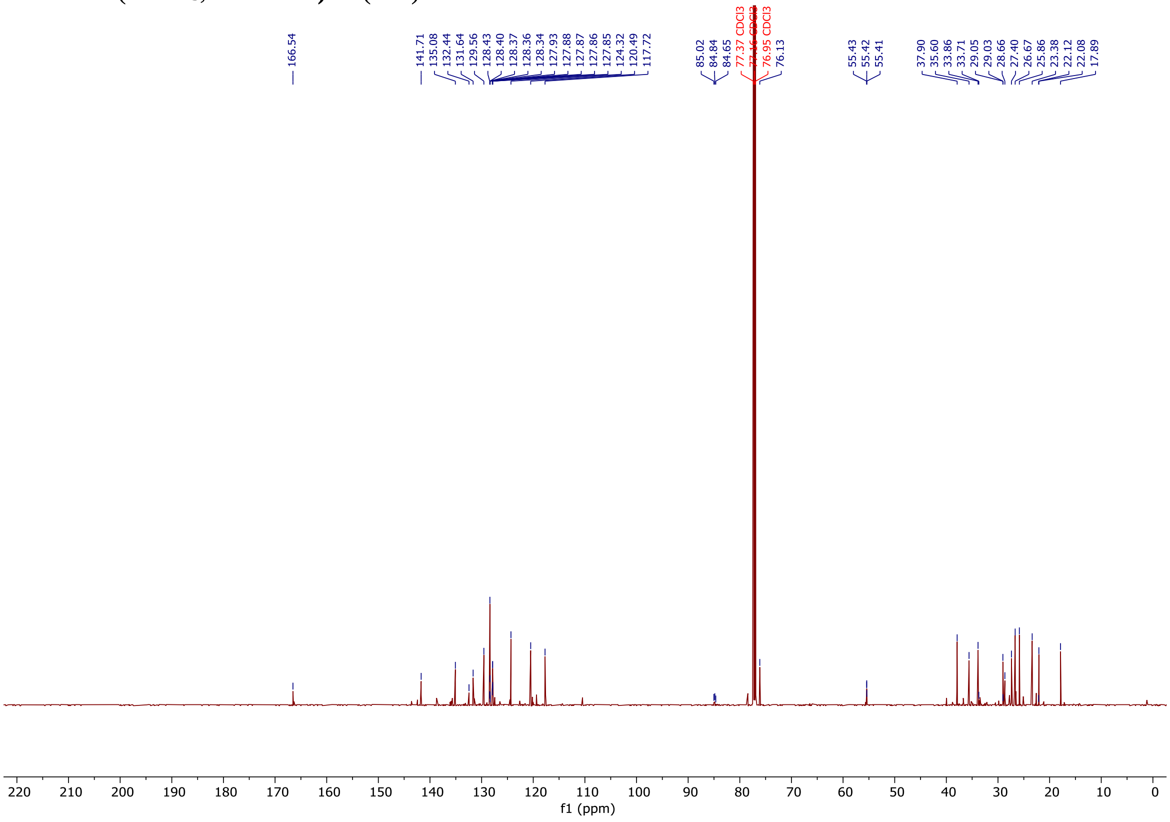

^{19}F NMR (565 MHz, CDCl$_3$) of (27a)

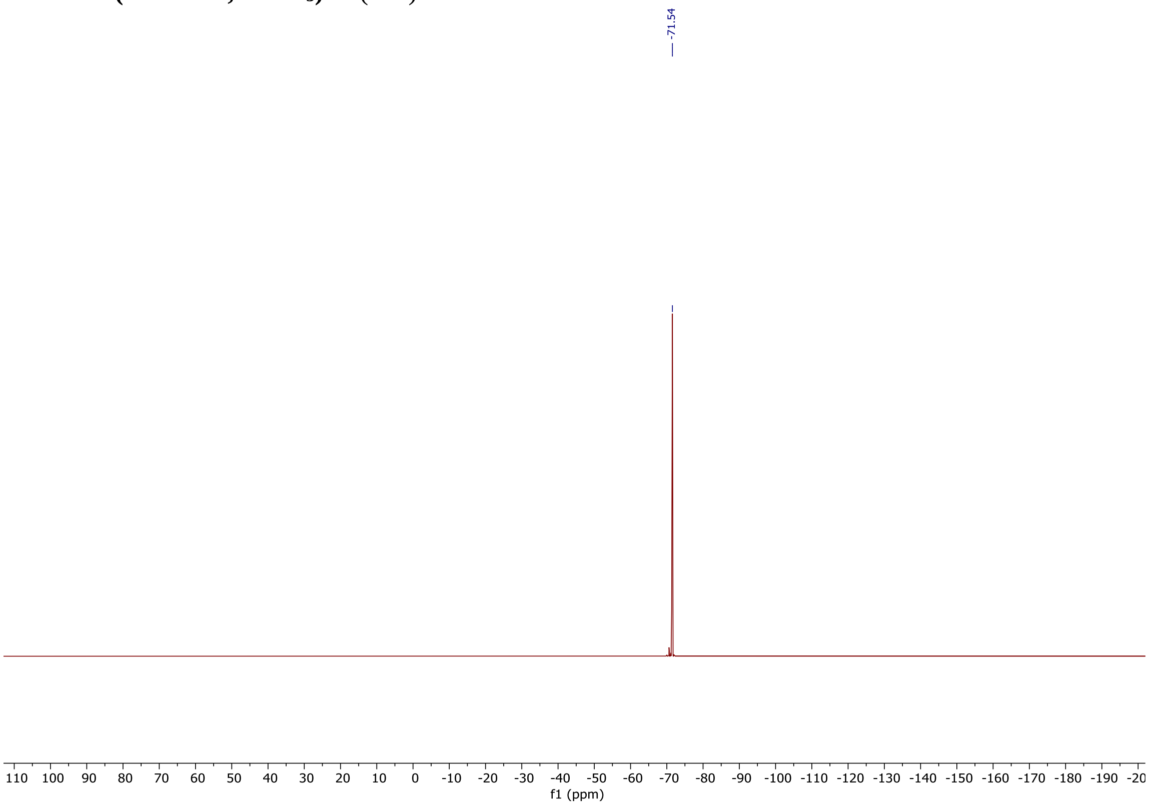

HSQC spectra of 27a

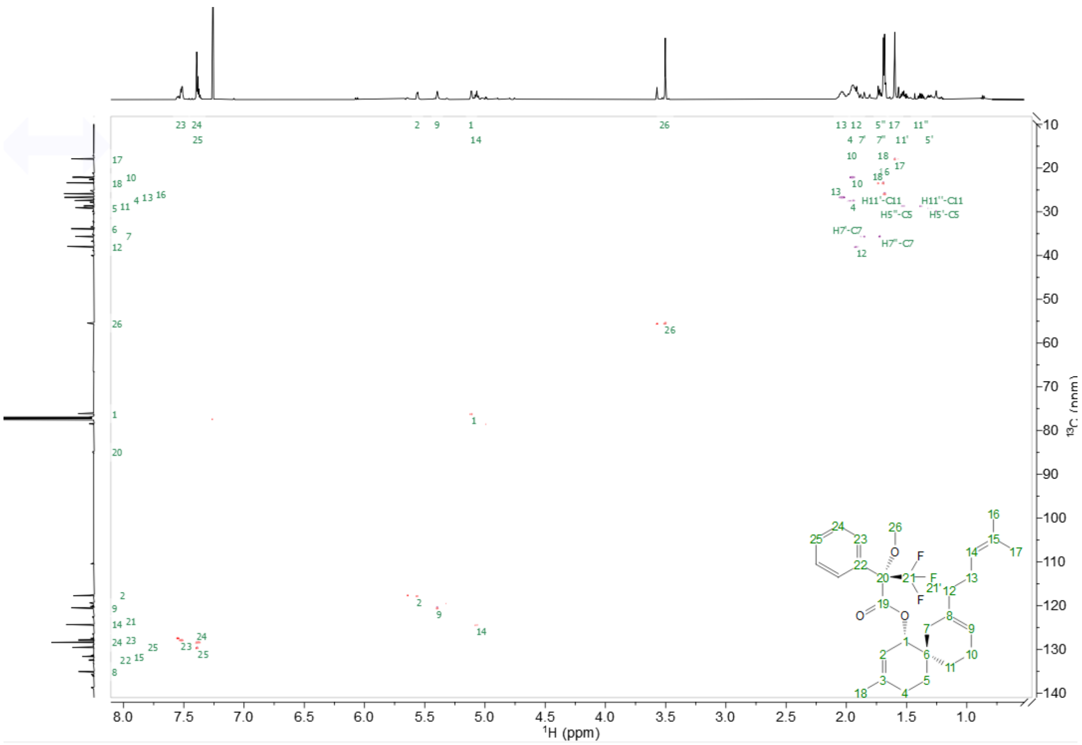

HMBC spectra of 27a

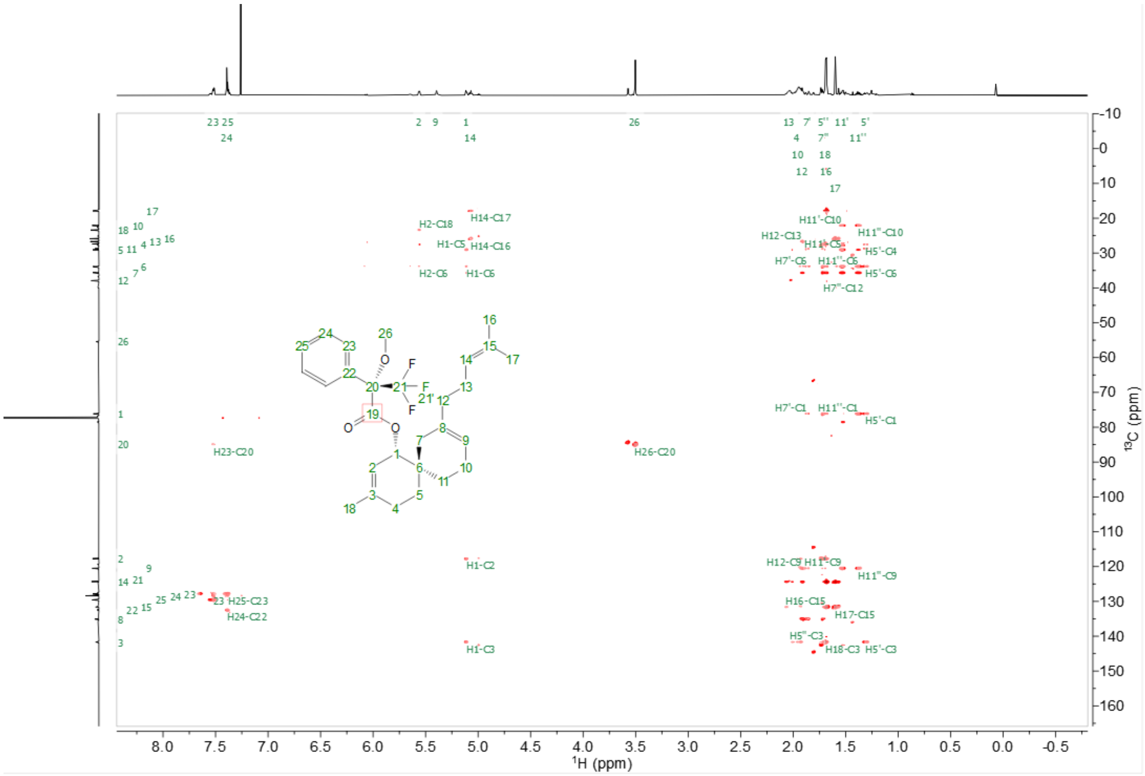

COSY spectra of 27a

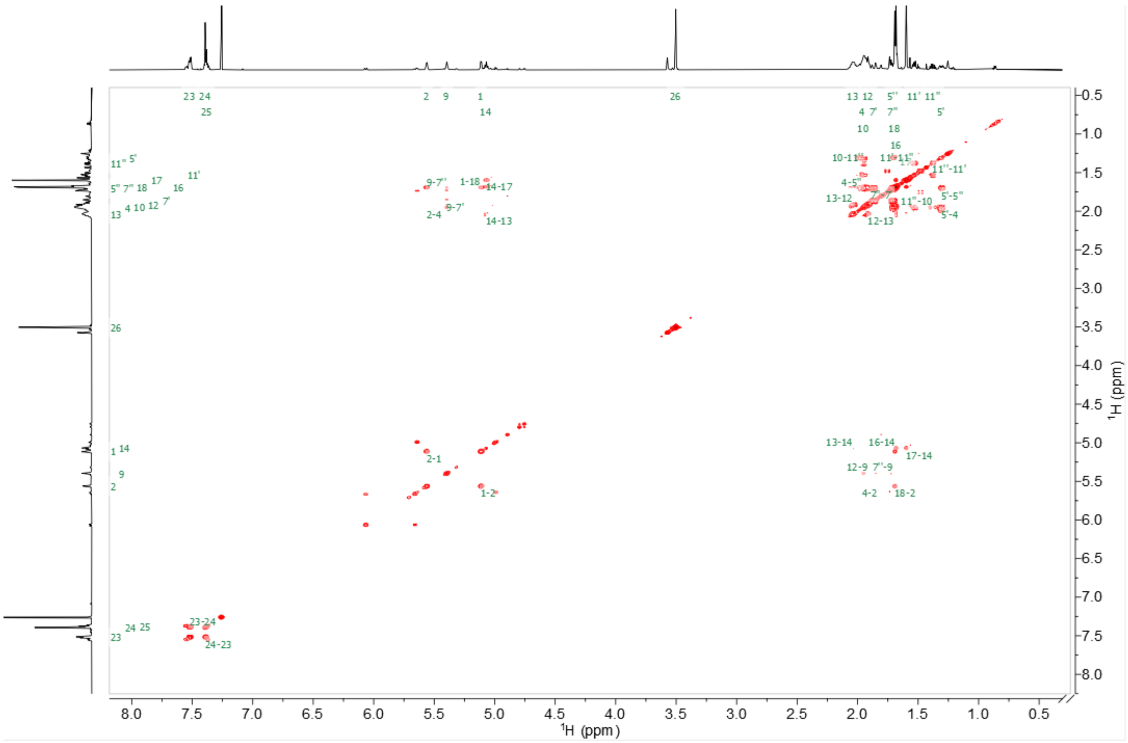

NOESY spectra of 27a

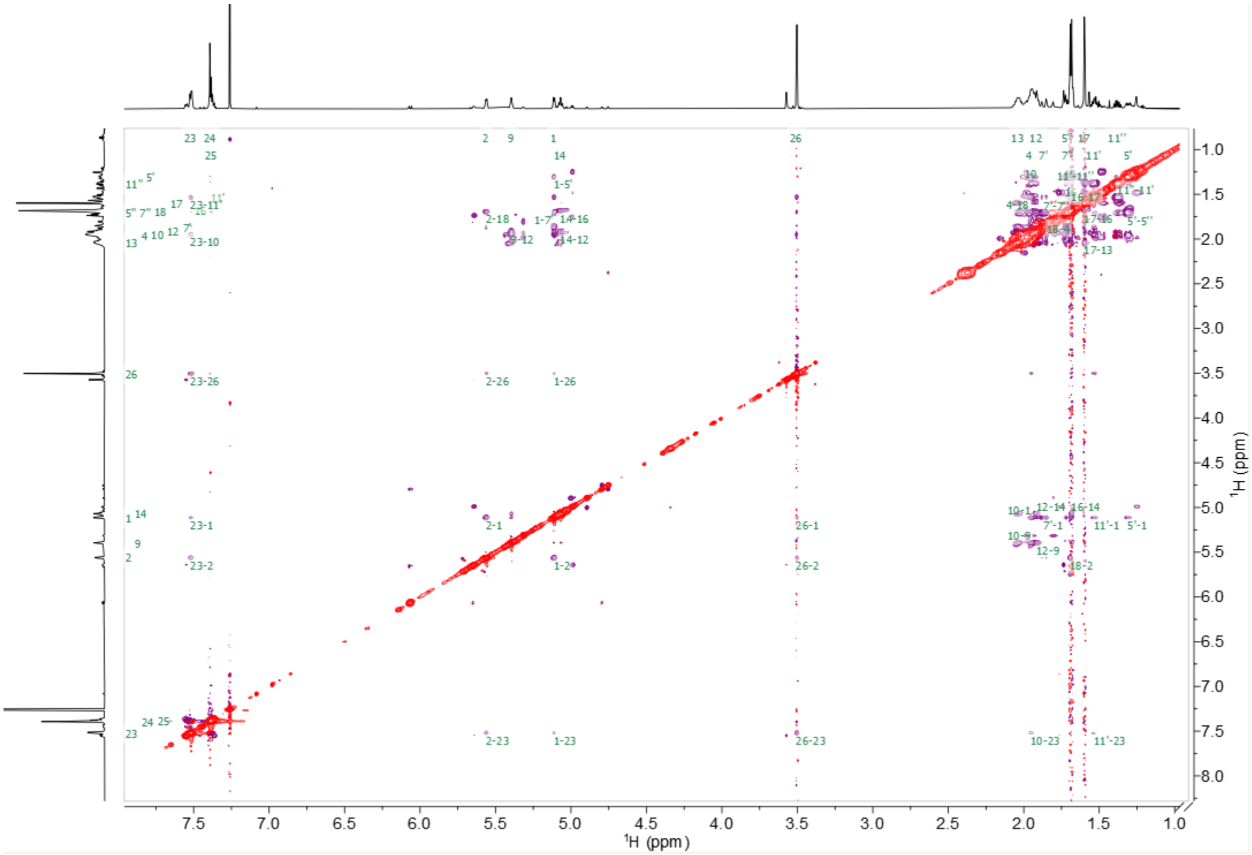

^{1}H NMR (CDCl$_3$, 600 MHz) of (27b)

(spectra contain in separable mixture of diastereomers and regioisomers)

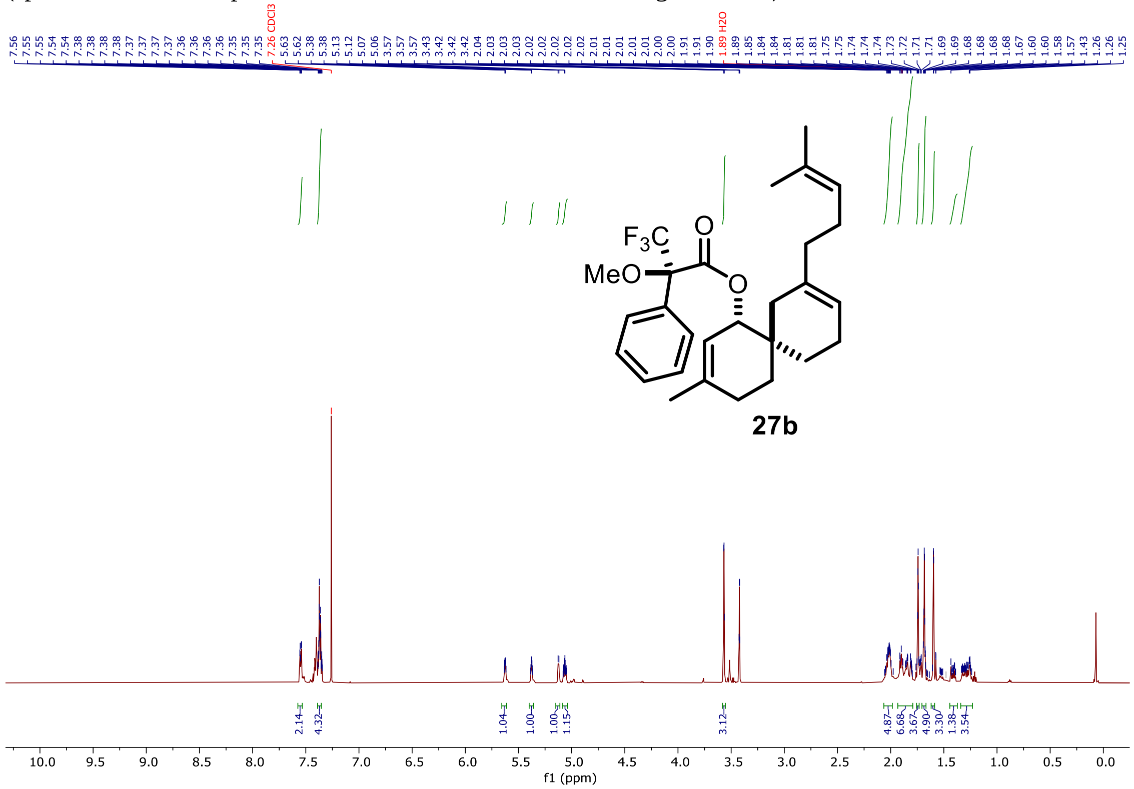

^{13}C NMR (CDCl$_3$, 151 MHz) of (27b)

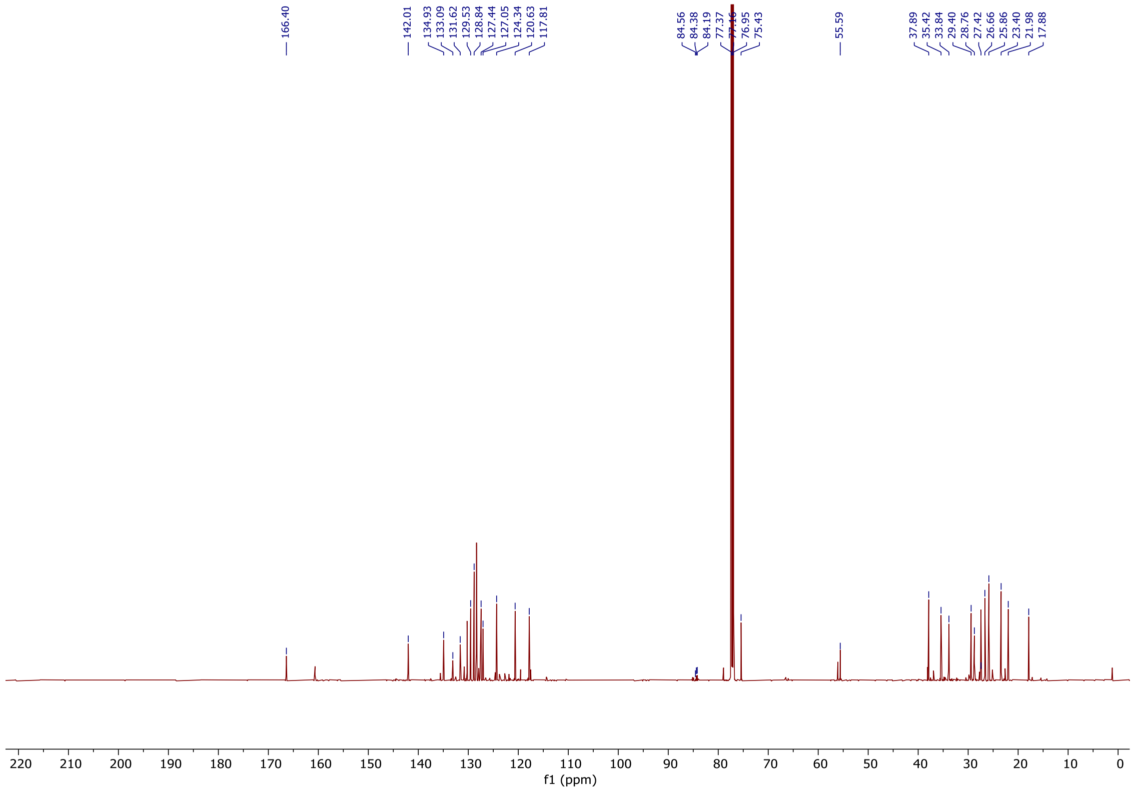

HSQC spectra of 27b

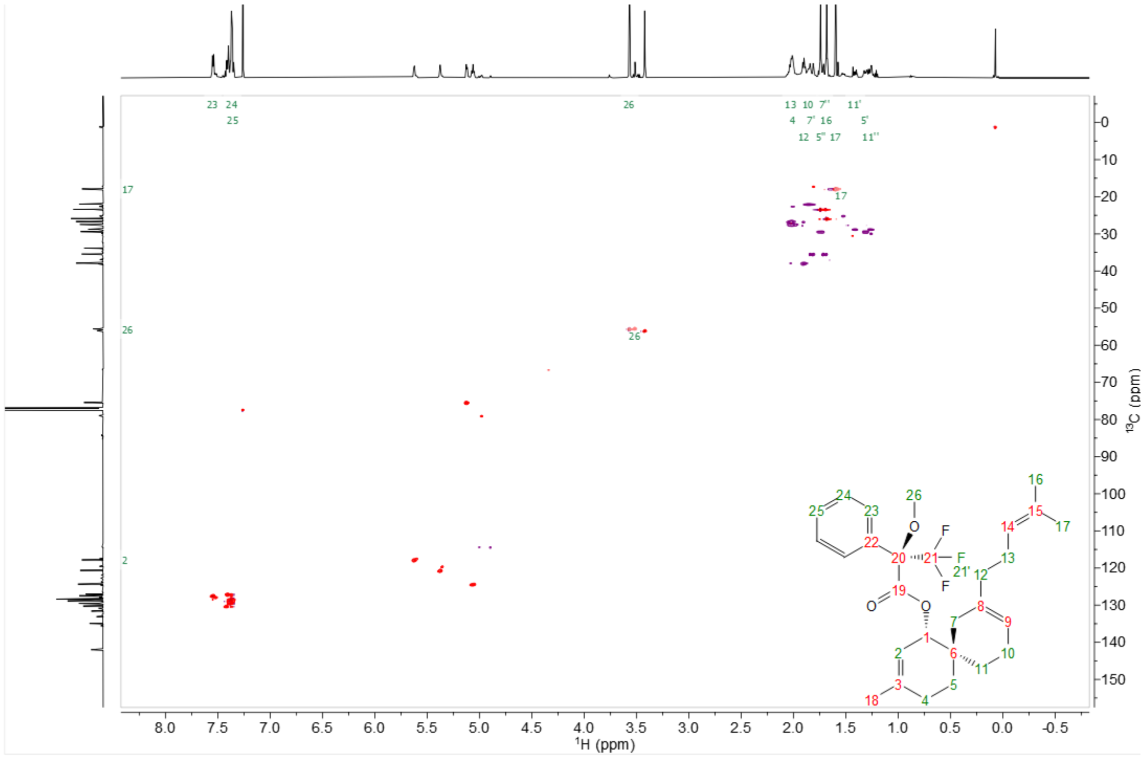

HMBC spectra of 27b

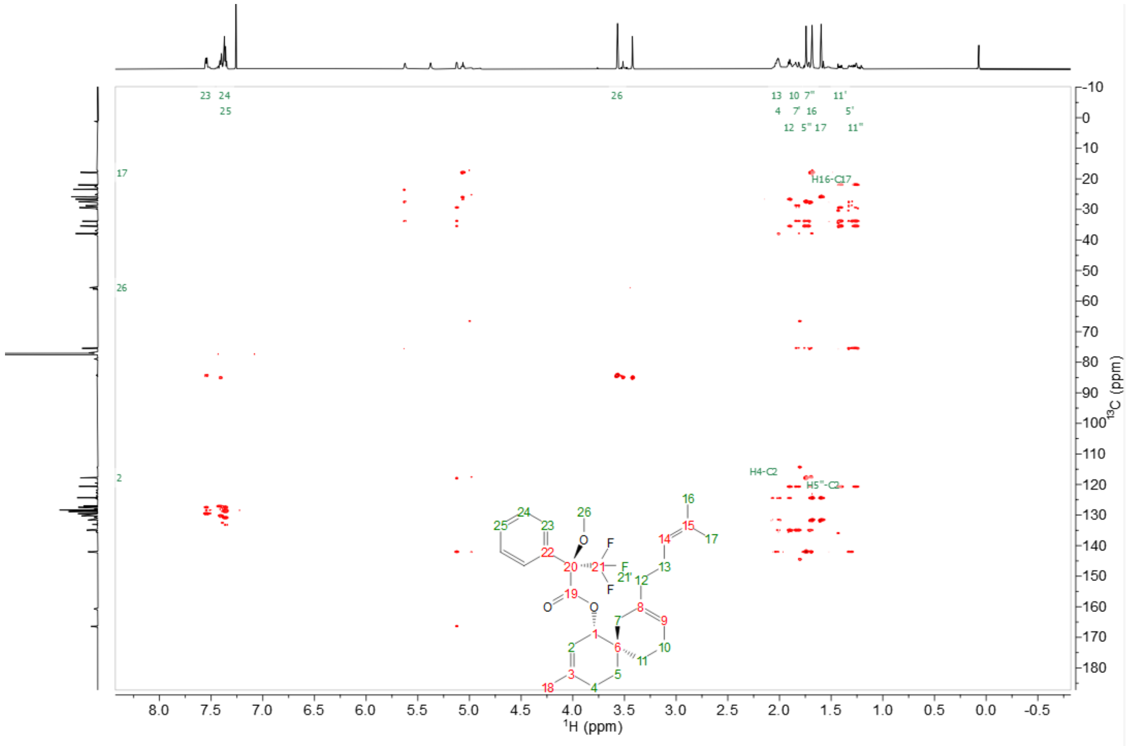

COSY spectra of 27b

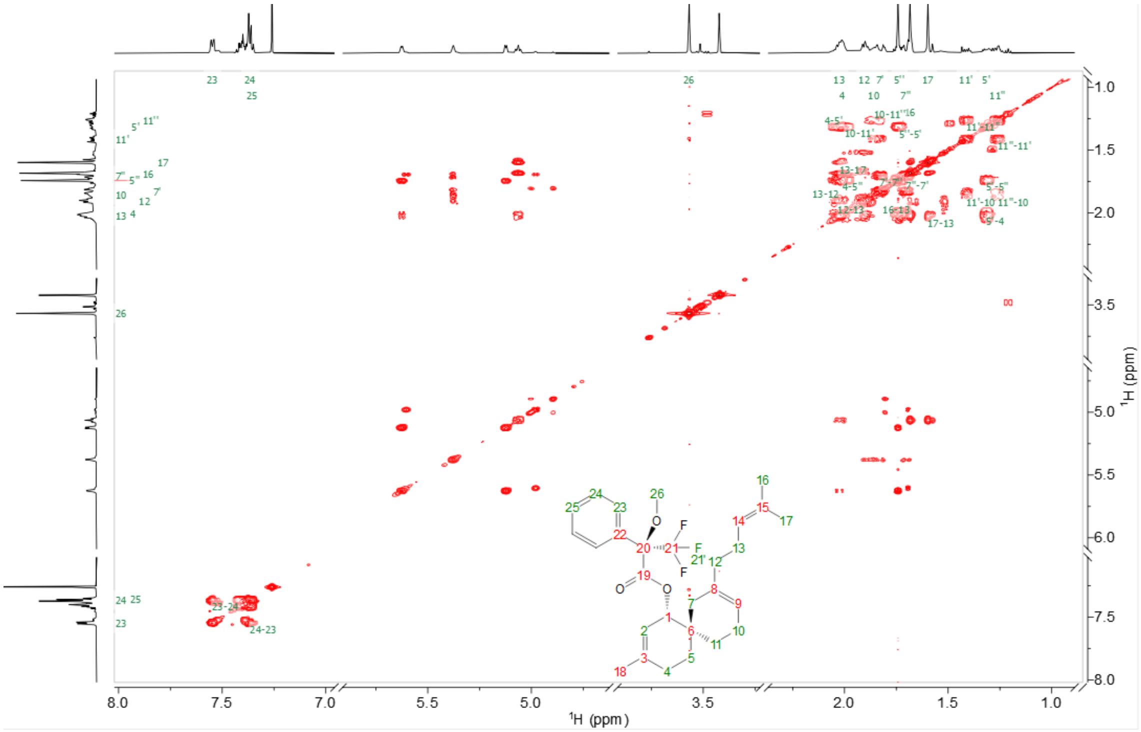

NOESY spectra of 27b

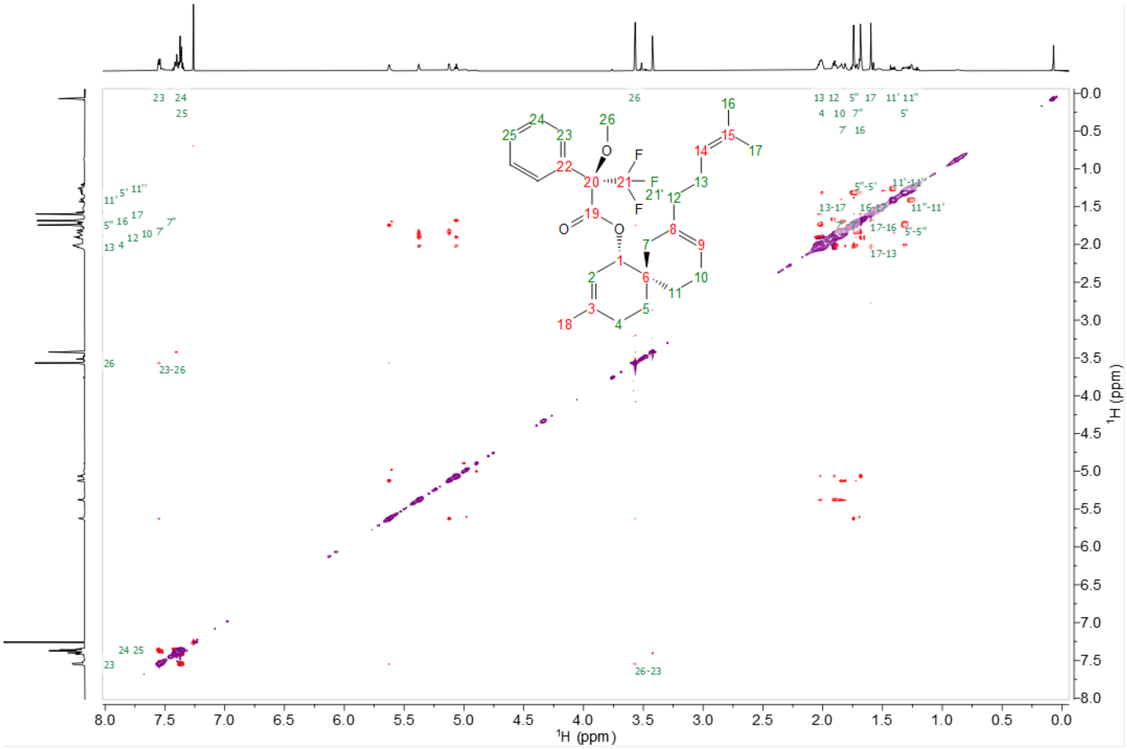

GC and HPLC Traces

GC Trace of Racemic (7a)

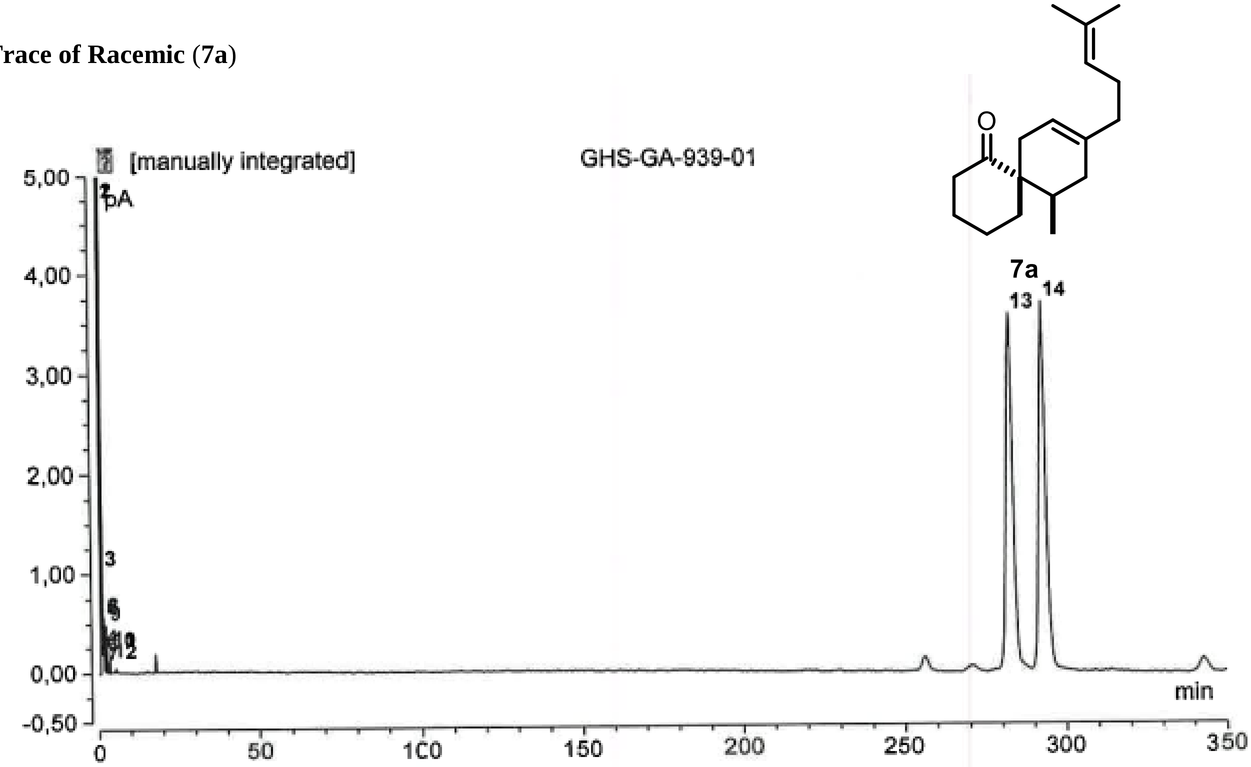

Sample:	GHS-GA-939-01	Instrument:	GC_112
Sequenz:	8075 GHS-GA PH	Measured:	03.03.21 10:40
Sequenz date:	01.03.21	Processing M.:	MPI
		Report-File:	Verhältnis

Razemat
Zuordnung nach achiral Messung

No.	Ret.Time min	Rel Area %	Peak Name
13	283,11	50,09	Component 1
14	293,30	49,91	Component 2

Instrument parameters:

Column:	25,0 m	Hydrodex-gamma-TBDAc-CD 0,25/?df G/624
Temperature:	220 / 115 iso / 350	
Gas:	0,60 bar	H2
Sample size:	0,2 µL	

GC Trace of Enantiopure (**7a**)

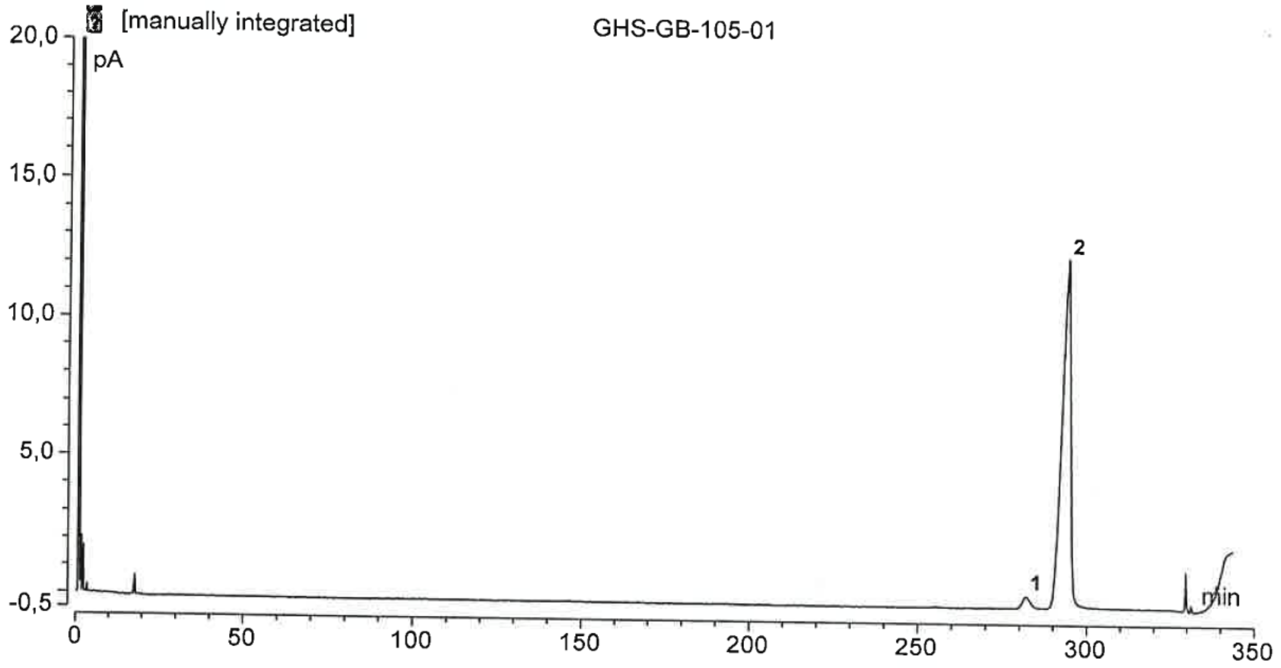

Sample:	**GHS-GB-105-01**	Instrument:	**GC_112**
Sequenz:	**8186 GHS-GB MM**	Measured:	**13.04.21 12:21**
Sequenz date:	**13.04.21**	Processing M.:	GHS 939
		Report-File:	Verhältnis 105-01

Enantiomerenverhältnis
Zuordnung nach achiral Messung
Rel.Area aufgrund der geringen Intensität der Peaks ungenau, Angaben unter Vorbehalt

No.	Ret.Time min	Rel.Area %	Peak Name
1	281,97	2,89	.
2	294,15	97,11	.

Instrument parameters:

Column:	25,0 m	Hydrodey-gamma-TBDAc-CD 0,25/?df G/624
Temperature:	220 / 115, 325 min iso 8/min 240, 3 min iso / 350	
Gas:	0,60 bar	H2
Sample size:	0,2 µL	

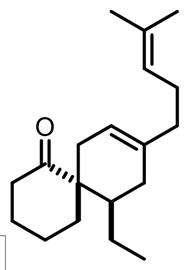

7b

HPLC Trace of Racemic (7b)

Column: IA-3R **Solvent**: 70:30 $CH_3CN:H_2O$, **Flow rate**: 1 mL/min

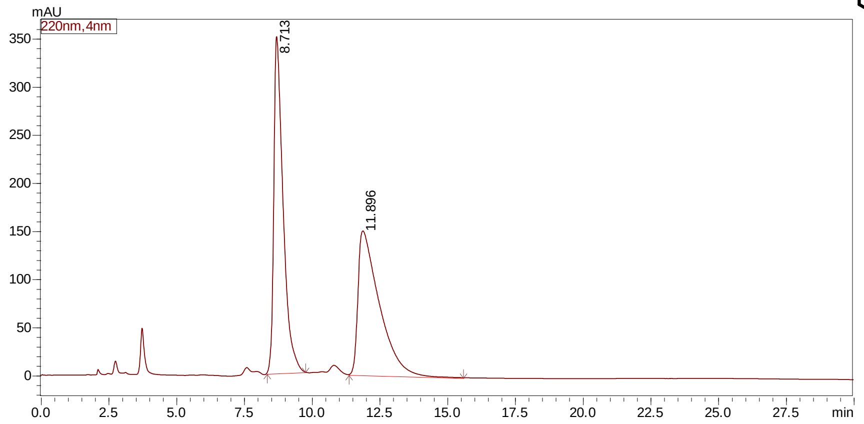

Peak#	Ret. Time	Area%
1	8.71	50.594
2	11.9	49.406
Total		100

HPLC Trace of Enantiopure (7b)

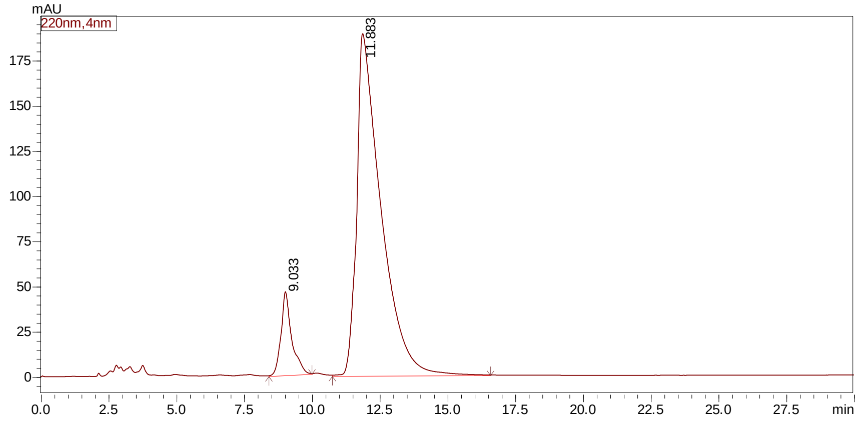

Peak#	Ret. Time	Area%
1	9.033	9.575
2	11.883	90.425
Total		100

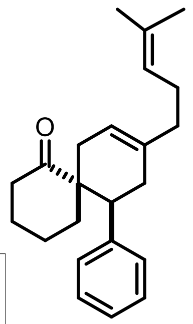

7c

HPLC Trace of Racemic (7c)

Column: AD-3R; **Solvent**: MeCN/H2O = 60/40; **Flow Rate**: 1.0 mL/min

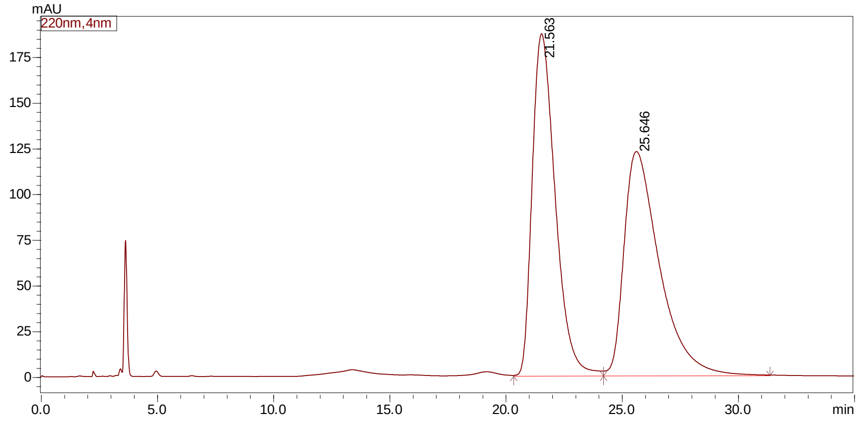

Peak#	Ret. Time	Area%
1	21.56	49.882
2	25.65	50.118
Total		100

HPLC Trace of Enantiopure (7c)

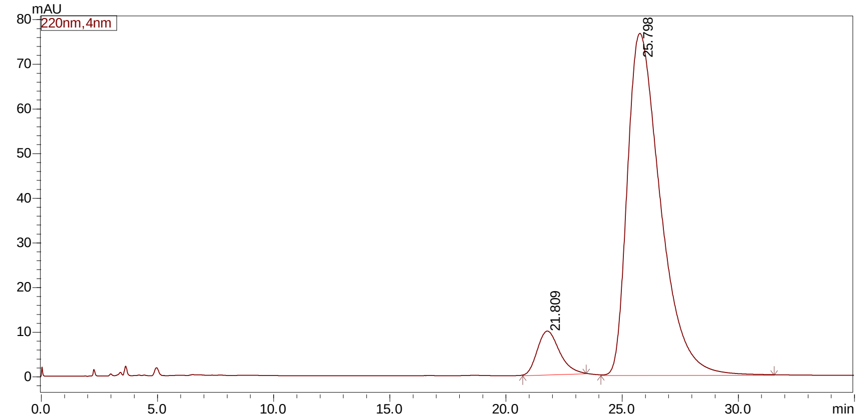

Peak#	Ret. Time	Area%
1	21.81	7.772
2	25.80	92.228
Total		100

HPLC Trace of Racemic (7d)

Column: AD-3R **Solvent**: 60:40 CH_3CN:H_2O, **Flow rate**: 1 mL/min

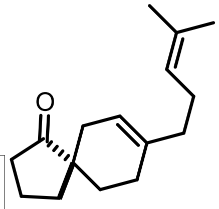

7d

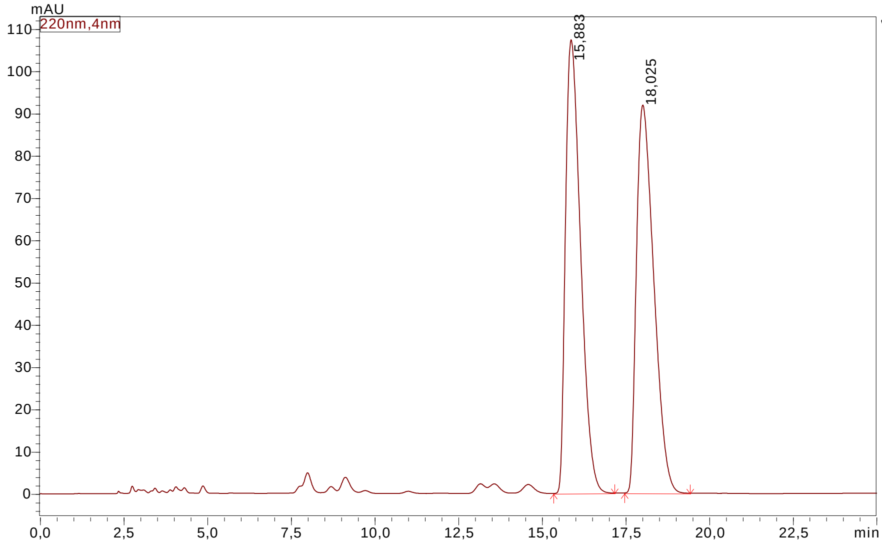

Peak#	Ret. Time	Area%
1	15.883	50.398
2	18.025	49.602
Total		100

HPLC Trace of Enantiopure (7d)

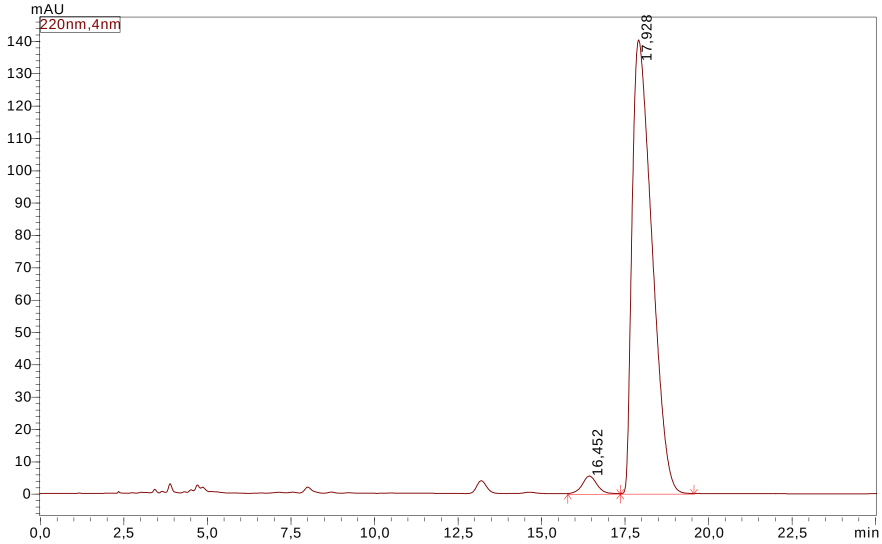

Peak#	Ret. Time	Area%
1	16.452	2.726
2	17.928	97.274
Total		100

HPLC Trace of Racemic (7e)

Column: AD-3R; **Solvent**: MeCN/H2O = 60/40; **Flow Rate**: 1.2 mL/min

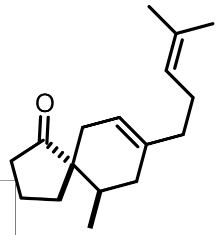

7e

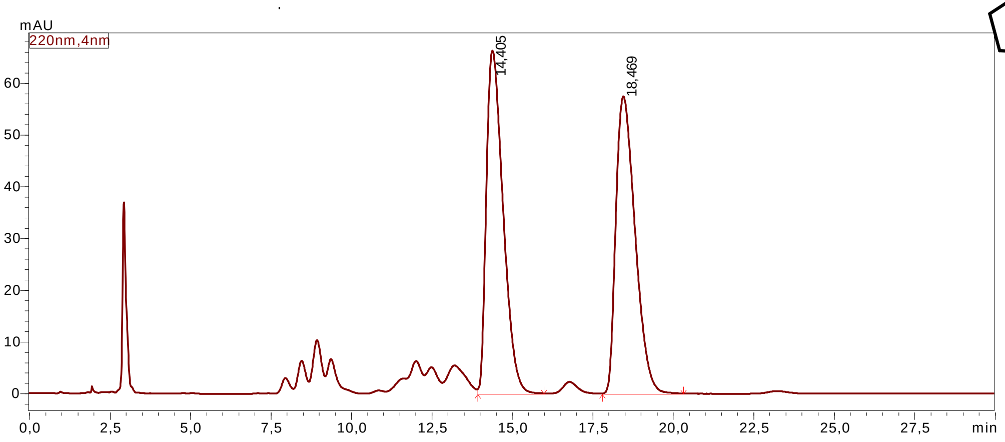

Peak#	Ret. Time	Area%
1	14.41	50.06
2	18.47	49.94
Total		100

HPLC Trace of Enantiopure (7e)

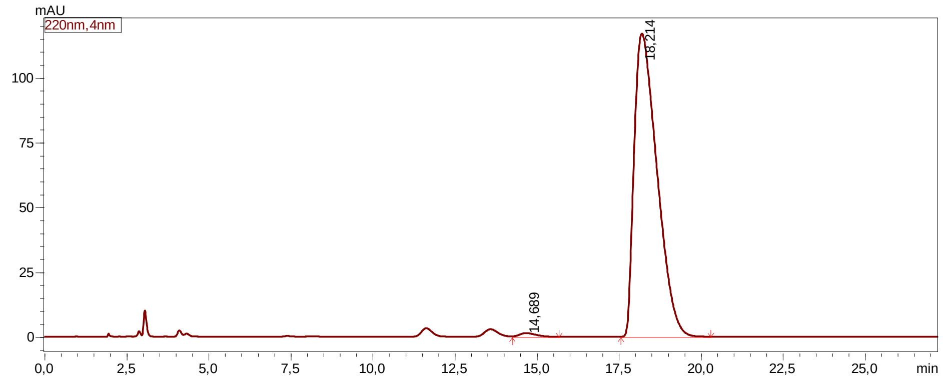

Peak#	Ret. Time	Area%
1	14.69	0.880
2	18.21	99.120
Total		100

HPLC Trace of Racemic (7f)

Column: IA-3R; **Solvent**: MeCN/H_2O = 70/30; **Flow Rate**: 1.2 mL/min

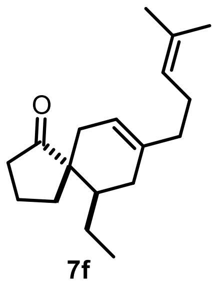

7f

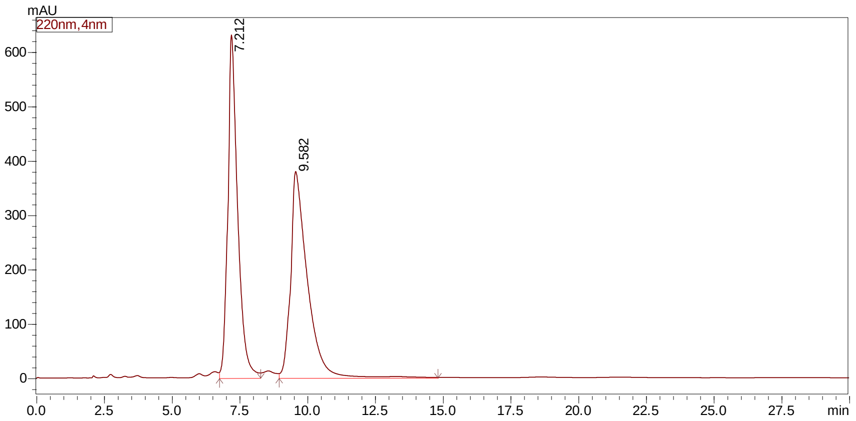

Peak#	Ret. Time	Area%
1	7.212	49.641
2	9.582	50.359
Total		100

HPLC Trace of Enantiopure (7f)

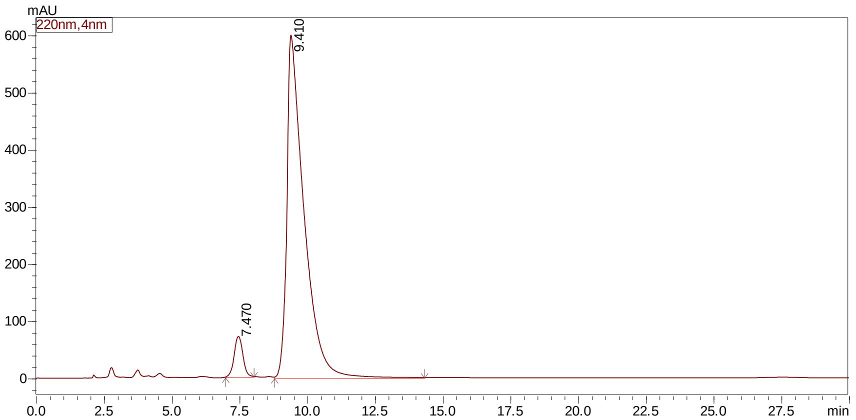

Peak#	Ret. Time	Area%
1	7.47	5.659
2	9.41	94.341
Total		100

HPLC Trace of Racemic (7g)

Column: AD-3R; **Solvent**: MeCN/H_2O = 70/30; **Flow Rate**: 1.0 mL/min

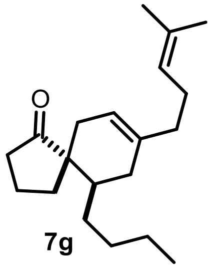

7g

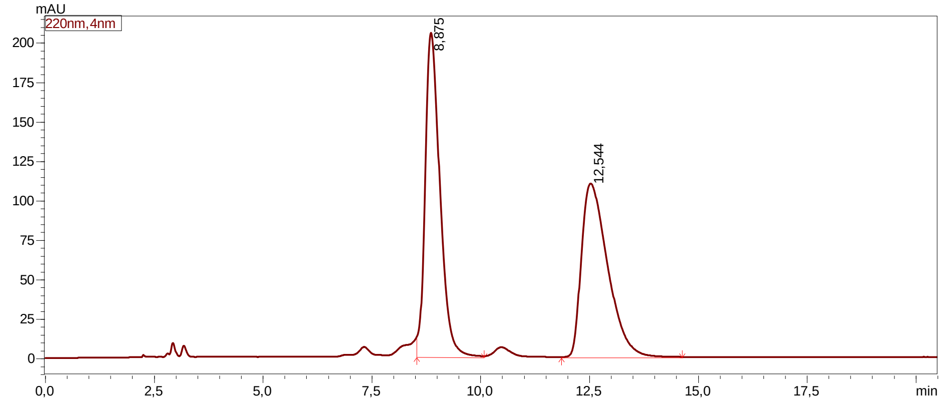

Peak#	Ret. Time	Area%
1	8.88	51.427
2	12.54	48.573
Total		100

HPLC Trace of Enantiopure (7g)

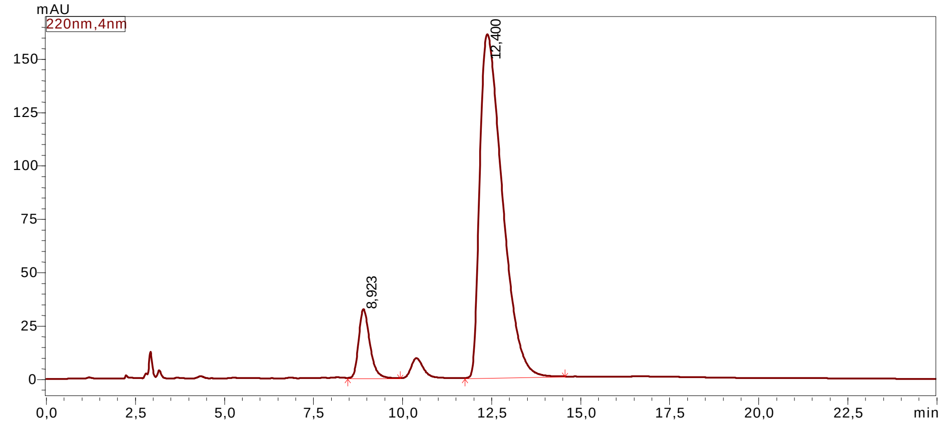

Peak#	Ret. Time	Area%
1	8.92	9.072
2	12.40	90.928
Total		100

HPLC Trace of Racemic (7h)

Column: AD-3R; **Solvent**: MeCN/H_2O = 60/40; **Flow Rate**: 1.0 mL/min

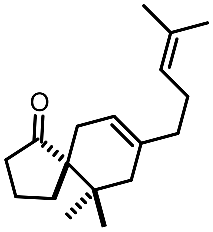

7h

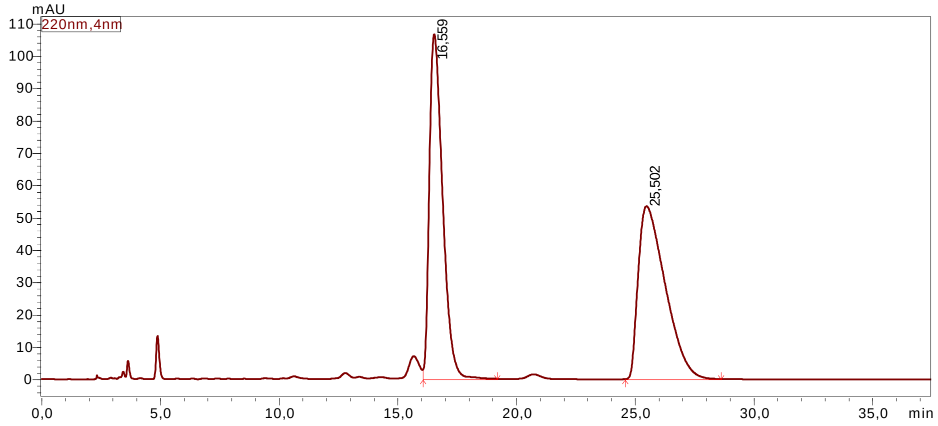

Peak#	Ret. Time	Area%
1	16.56	49.440
2	25.50	50.560
Total		100

HPLC Trace of Enantiopure (7h)

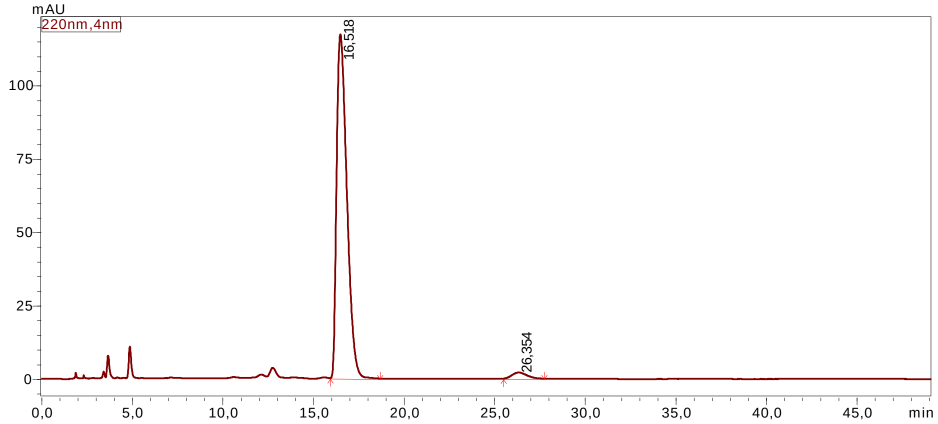

Peak#	Ret. Time	Area%
1	16.52	97.481
2	26.35	2.519
Total		100

HPLC Trace of Racemic (7i) and (7i')

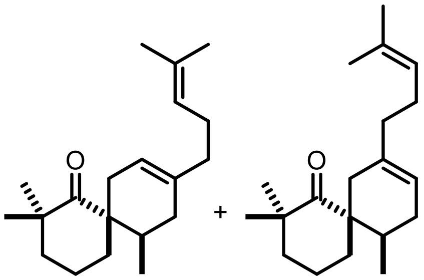

7i + 7i'

Column: AD-3R; **Solvent**: MeCN/H_2O = 70/30; **Flow Rate**: 1.0 mL/min

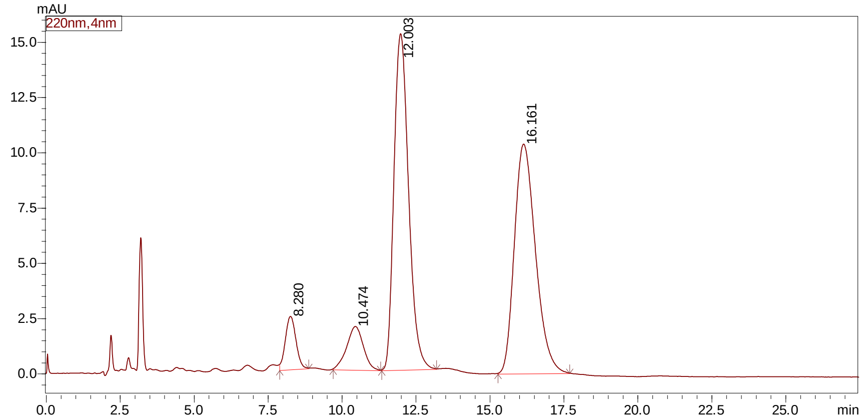

Peak#	Ret. Time	Area%	
1	8.28	5.026	E1/R1
2	10.47	6.458	E2/R1
3	12.00	44.682	E1/R2
4	16.16	43.833	E2/R2
Total		100	

HPLC Trace of Enantiopure (7i)

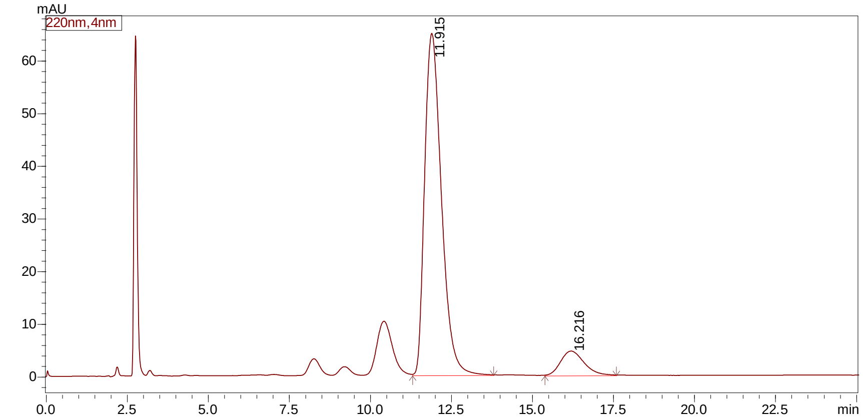

Peak#	Ret. Time	Area%	
3	11.92	91.193	E1/R2
4	16.22	8.807	E2/R2
Total		100	

HPLC Trace of Racemic (7j) and (7j')

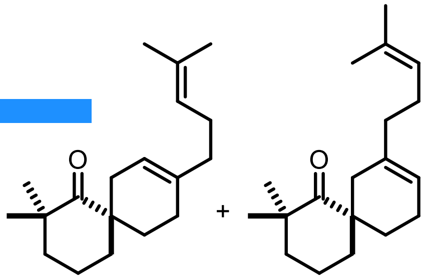

7j 7j'

2D-LC Results Report

Data File:	W:\Workgroup_Geräte\NP-4 2D\Kundendaten\Ghosh\GHS-GB-068-01-HC001.D
Sample Name:	GHS-GB-068-01-HC
Description:	1.0 µL GHS-GB-068-01 (1 µL in 200 µL n-Heptan)<br>100 mm Zorbax RX SIL, 4.6 mm i.D.,<br>n-Heptan / 2-Propanol = 99.9:0.1 (v/v)<br>1.0 mL / min, 13.9 MPa, 308 K<br>UV, 220 nm<br><br>HC peak based 30 mAu<br><br>150 mm Chiralcel OD-3, 4.6 mm i.D.<br>n-Heptan/2-Propanol = 99.8:0.2 (v/v)<br>1.0 mL/min, 8.3 MPa, 298K<br>UV, 220 nm

Instrument:	NP-4 2D	Location:	D1F-A1
Injection date:	27-Aug-21, 14:33:25	Injection volume:	1.00
Acq. method:	Ghosh-HC.M	Acq. operator:	SYSTEM

¹D chromatogram(s)

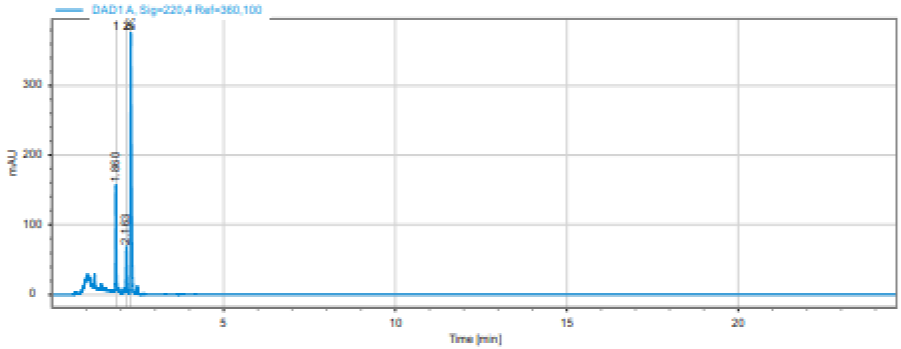

Sampling table (¹D)

Cut group	Cut #	¹D Cut start [min]	¹D Ret. time [min]	¹D Duration [min]	Trigger	²D Run start [min]	
	1	1.84	1.860	0.04	Peak	1.89	impurity
	2	2.15	2.163	0.04	Peak	14.00	1. diastereomer
	3	2.27	2.294	0.04	Peak	8.90	2. diastereomer

Component table

Signal: DAD2 A, Sig=220,4 Ref=360,100

Component	¹D Sampling range [min]	Ret.Time ²D [min]	Area	Area%	
1	1.84 - 1.88	2.480	659.998	12.111	impurity
2	1.84 - 1.88	2.581	688.578	12.635	impurity
3	1.84 - 1.88	3.566	32.167	0.590	impurity
4	2.15 - 2.19	2.887	242.613	4.452	1. diastereomer 1. enantiomer
5	2.15 - 2.19	3.270	313.592	5.754	1. diastereomer 2.enantiomer+impurity
6	2.15 - 2.19	3.417	73.788	1.354	impurity
7	2.27 - 2.31	3.432	1719.318	31.549	2. diastereomer 1. enantiomer
8	2.27 - 2.31	3.706	1719.592	31.554	2. diastereomer 2. enantiomer

no better separation possible

Printed 27.08.2021 15:01 Page 1

2D-LC Results Report

Cut# : 1

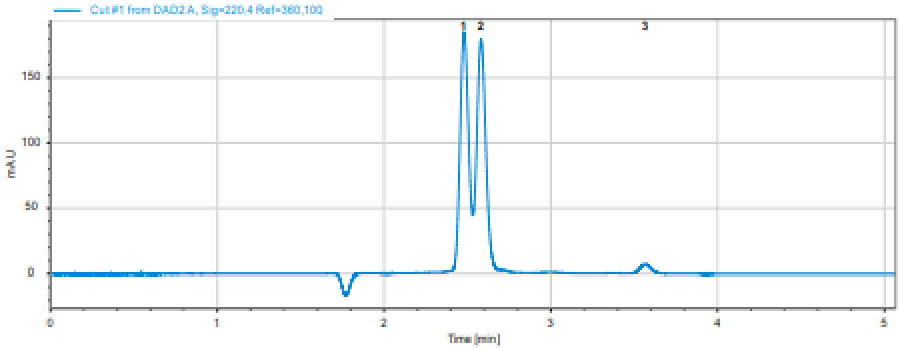

Signal: DAD2 A, Sig=220,4 Ref=360,100

Compound	Cut	Ret.Time	Area	Width	Height	Symmetry
1	1	2.480	659.998<br>**659.998**	0.056	184.083	0.883
2	1	2.581	688.578<br>**688.578**	0.059	178.483	0.859
3	1	3.566	32.167<br>**32.167**	0.061	7.090	0.878

2D-LC Results Report

Cut# : 2

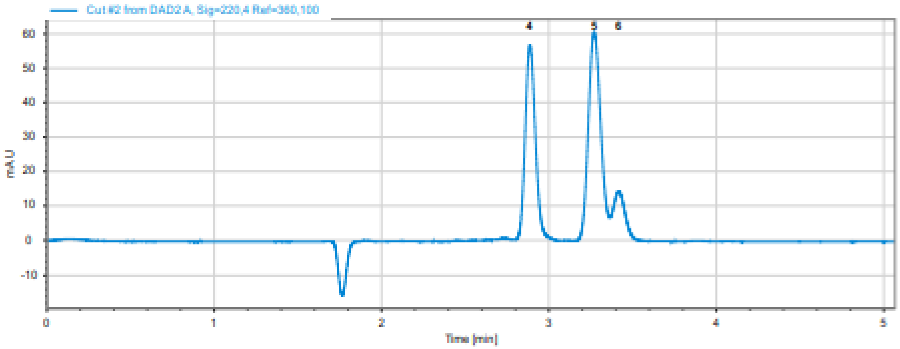

Signal: DAD2 A, Sig=220,4 Ref=360,100

Compound	Cut	Ret.Time	Area	Width	Height	Symmetry
4	2	2.887	242.613<br>**242.613**	0.066	56.765	0.824
5	2	3.270	313.592<br>**313.592**	0.079	60.639	0.833
6	2	3.417	73.788<br>**73.788**	0.071	14.381	0.856

2D-LC Results Report

Cut# : 3

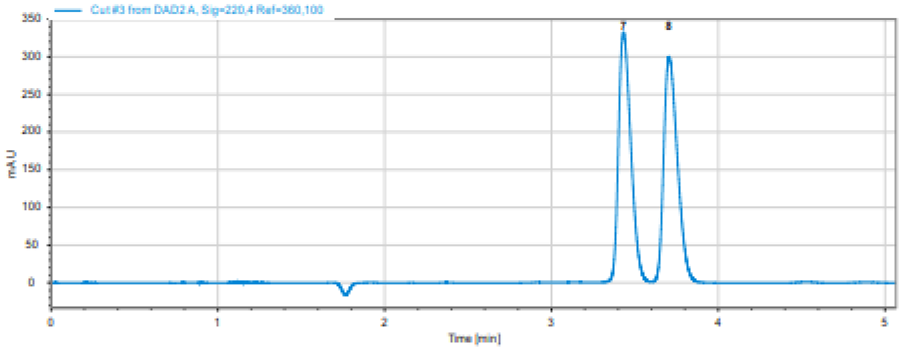

Signal: DAD2 A, Sig=220,4 Ref=360,100

Compound	Cut	Ret.Time	Area	Width	Height	Symmetry
7	3	3.432	1719.318<br>**1719.318**	0.081	332.306	0.638
8	3	3.706	1719.592<br>**1719.592**	0.089	300.016	0.620

```
======================================================================
Acq. Operator   : SYSTEM
Sample Operator : SYSTEM
Acq. Instrument : NP-4 2D                          Location :   D1F-A1
Injection Date  : 27.08.2021 14:33:25                   Inj :   1
                                                 Inj Volume : 1.000 µl
Acq. Method     : W:\WORKGROUP\_GERÄTE\NP-4 2D\KUNDENDATEN\GHOSH\Ghosh-HC.M
Last changed    : 27.08.2021 14:07:14 by SYSTEM
                  (modified after loading)
Analysis Method : W:\WORKGROUP\_GERÄTE\NP-4 2D\KUNDENDATEN\GHOSH\Ghosh.M
Last changed    : 27.08.2021 09:30:14 by SYSTEM
                  (modified after loading)
Sample Info     : 1.0 µL GHS-GB-068-01 (1 µL in 200 µL n-Heptan)
                  100 mm Zorbax RX SIL, 4.6 mm i.D.,
                  n-Heptan / 2-Propanol = 99.9:0.1 (v/v)
                  1.0 mL / min, 13.9 MPa, 308 K
                  UV, 220 nm
                  HC peak based 30 mAu
                  150 mm Chiralcel OD-3, 4.6 mm i.D.
                  n-Heptan/2-Propanol = 99.8:0.2 (v/v)
                  1.0 mL/min, 8.3 MPa, 298K
                  UV, 220 nm
```

Additional Info : Peak(s) manually integrated

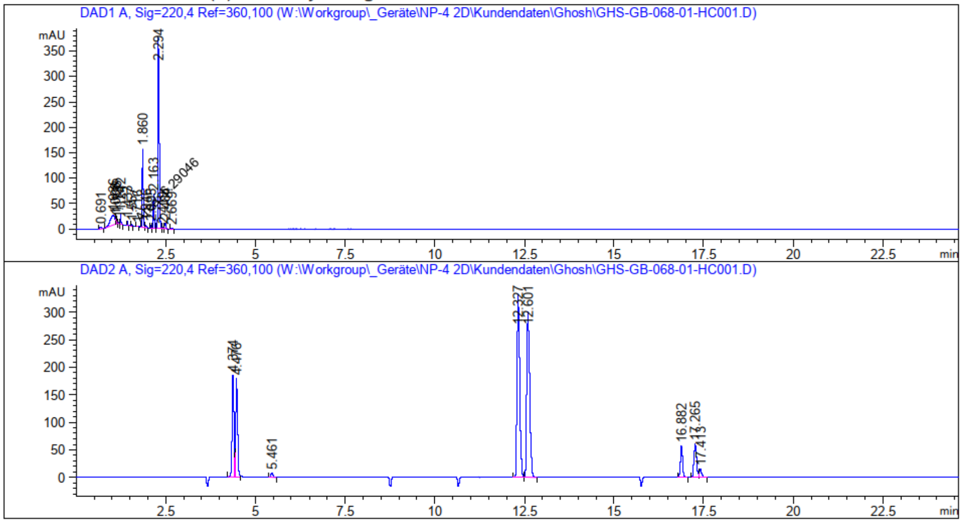

===

Area Percent Report

===

Sorted By : Signal
Multiplier : 1.0000
Dilution : 1.0000
Do not use Multiplier & Dilution Factor with ISTDs

Signal 1: DAD1 A, Sig=220,4 Ref=360,100

Peak #	RetTime [min]	Type	Width [min]	Area [mAU*s]	Height [mAU]	Area %	
1	0.691	BB	0.0584	10.18335	2.64763	0.5917	
2	1.036	BV	0.1104	161.64648	19.97437	9.3920	
3	1.098	VV	0.0214	14.40798	10.25995	0.8371	
4	1.128	VB	0.0166	14.50107	13.44717	0.8425	
5	1.173	BV	0.0328	6.43816	3.08980	0.3741	
6	1.242	VB	0.0182	23.44633	19.90106	1.3623	
7	1.433	BB	0.0226	12.10783	8.26194	0.7035	
8	1.557	MF	0.0258	6.29046	4.05707	0.3655	
9	1.716	BV	0.0305	2.22678	1.05977	0.1294	
10	1.860	VV	0.0276	277.76654	153.53761	16.1389	
11	1.946	VB	0.0330	12.77919	5.73752	0.7425	
12	2.095	BV	0.0307	12.80354	6.59049	0.7439	
13	2.163	VB	0.0332	141.65829	66.95653	8.2307	1. diastereomer
14	2.294	BV	0.0402	986.07782	374.82327	57.2935	2. diastereomer
15	2.419	VV	0.0355	6.31512	2.68270	0.3669	
16	2.484	VB	0.0395	28.06780	10.90471	1.6308	
17	2.669	VB	0.0456	4.38343	1.54348	0.2547	

Totals : 1721.10020 705.47507

Signal 2: DAD2 A, Sig=220,4 Ref=360,100

Peak #	RetTime [min]	Type	Width [min]	Area [mAU*s]	Height [mAU]	Area %	
1	4.374	BV	0.0556	659.99835	184.08284	12.1108	
2	4.476	VV	0.0593	688.57806	178.48268	12.6353	
3	5.461	VV	0.0609	32.16674	7.08994	0.5903	
4	12.327	BV	0.0809	1719.31836	332.30591	31.5492	2. diastereomer 1. enantiomer
5	12.601	VV	0.0894	1719.59241	300.01636	31.5542	2. diastereomer 2. enantiomer
6	16.882	VB	0.0659	242.61320	56.76513	4.4519	1. diastereomer 1. enantiomer
7	17.265	BV	0.0791	313.59216	60.63866	5.7544	2. diastereomer 2. enantiomer + impurity
8	17.413	VV	0.0714	73.78756	14.38132	1.3540	impurity

Totals : 5449.64685 1133.76284

HPLC Trace of Enantiopure (7j) and (7j')

2D-LC Results Report

Data File:	W:\Workgroup_Geräte\NP-4 2D\Kundendaten\Ghosh\GHS-GB-138-01-HC002.D
Sample Name:	GHS-GB-138-01-HC
Description:	1.0 µL GHS-GB-138-01 (1 µL in 200 µL n-Heptan)<br>100 mm Zorbax RX SIL, 4.6 mm i.D.,<br>n-Heptan / 2-Propanol = 99.9:0.1 (v/v)<br>1.0 mL / min, 13.9 MPa, 308 K<br>UV, 220 nm<br><br>HC peak based 30 mAu<br><br>150 mm Chiralcel OD-3, 4.6 mm i.D.<br>n-Heptan/2-Propanol = 99.8:0.2 (v/v)<br>1.0 mL/min, 8.3 MPa, 298K<br>UV, 220 nm

Instrument:	NP-4 2D	Location:	D1F-A2
Injection date:	27-Aug-21, 14:08:27	Injection volume:	1.00
Acq. method:	Ghosh-HC.M	Acq. operator:	SYSTEM

¹D chromatogram(s)

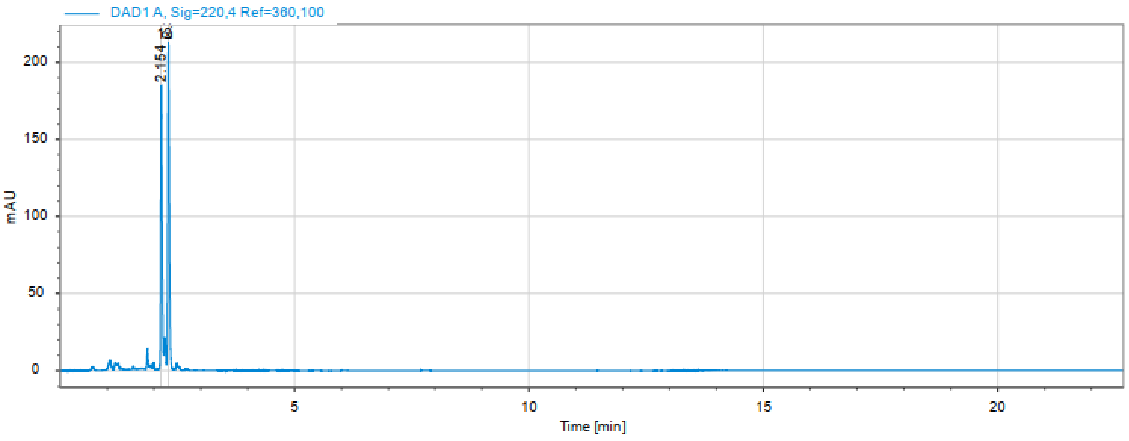

Sampling table (¹D)

Cut group	Cut #	¹D Cut start [min]	¹D Ret. time [min]	¹D Duration [min]	Trigger	²D Run start [min]	
	1	2.13	2.154	0.04	Peak	2.19	1. diastereomer
	2	2.28	2.306	0.04	Peak	9.19	2. diastereomer

Component table

Signal: DAD2 A, Sig=220,4 Ref=360,100

ee 1 = 86.6%

Component	¹D Sampling range [min]	Ret.Time ²D [min]	Area	Area%	
1	2.13 - 2.17	2.885	117.072	3.188	1. diastereomer 1. enantiomer
2	2.13 - 2.17	3.254	1627.548	44.324	1. diastereomer 2. enantiomer
3	2.28 - 2.32	3.454	78.290	2.132	2. diastereomer 1. enantiomer
4	2.28 - 2.32	3.706	1793.875	48.853	2. diastereomer 2. enantiomer
5	2.28 - 2.32	4.535	55.165	1.502	

ee 2 = 91.6%

2D-LC Results Report

Cut# : 1

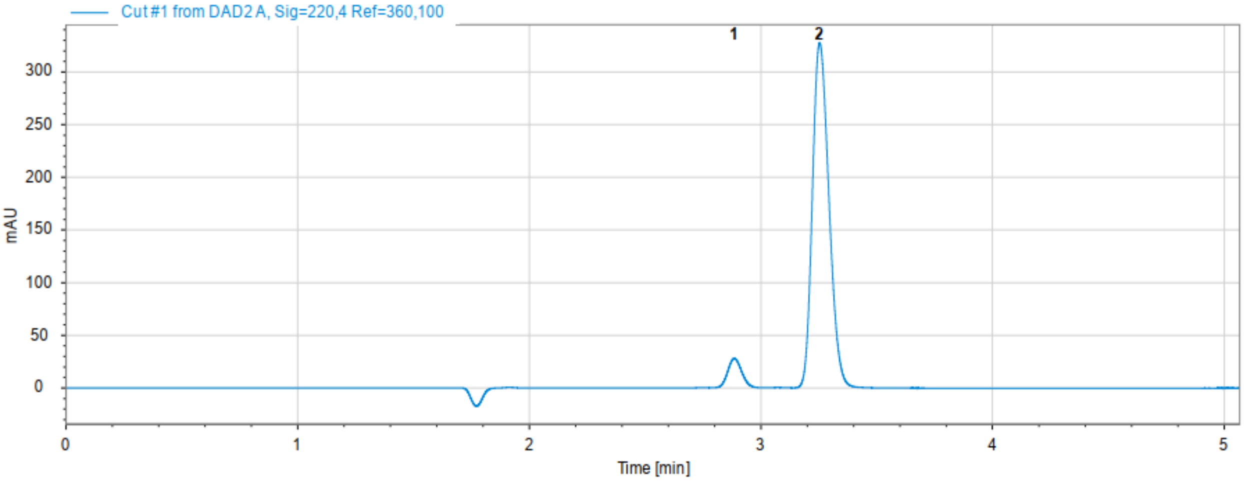

Signal: DAD2 A, Sig=220,4 Ref=360,100

Compound	Cut	Ret.Time	Area	Width	Height	Symmetry
1	1	2.885	117.072<br>**117.072**	0.063	27.905	0.872
2	1	3.254	1627.548<br>**1627.548**	0.078	327.630	0.741

2D-LC Results Report

Cut# : 2

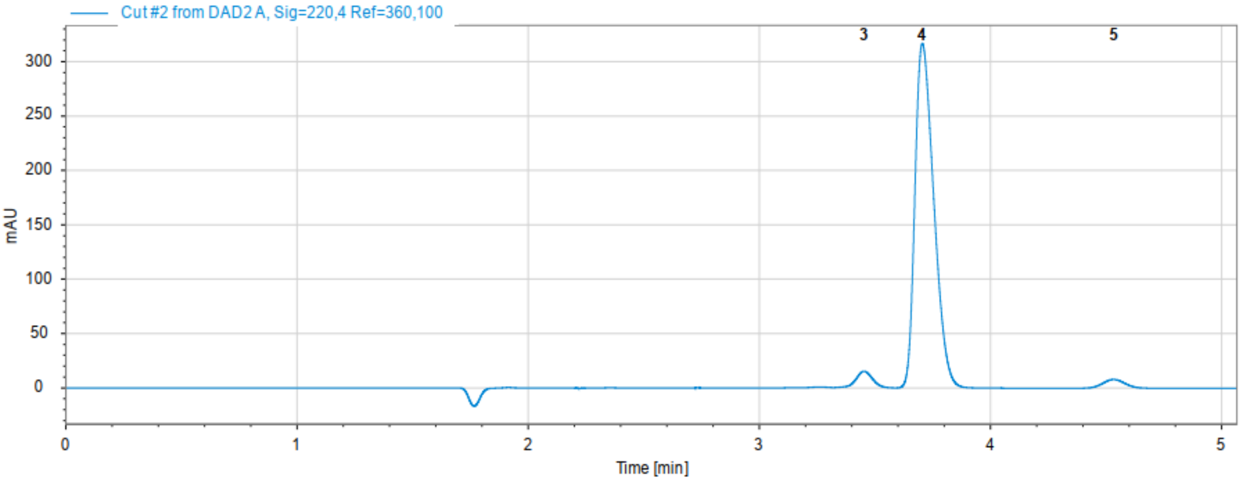

Signal: DAD2 A, Sig=220,4 Ref=360,100

Compound	Cut	Ret.Time	Area	Width	Height	Symmetry
3	2	3.454	78.290<br>**78.290**	0.073	15.376	1.004
4	2	3.706	1793.875<br>**1793.875**	0.087	316.877	0.610
5	2	4.535	55.165<br>**55.165**	0.083	8.164	0.990

```
=====================================================================
Acq. Operator   : SYSTEM
Sample Operator : SYSTEM
Acq. Instrument : NP-4 2D                          Location :   D1F-A2
Injection Date  : 27.08.2021 14:08:27                   Inj :   1
                                                Inj Volume : 1.000 µl
Acq. Method     : W:\WORKGROUP\_GERÄTE\NP-4 2D\KUNDENDATEN\GHOSH\Ghosh-HC.M
Last changed    : 27.08.2021 14:07:14 by SYSTEM
                  (modified after loading)
Analysis Method : W:\WORKGROUP\_GERÄTE\NP-4 2D\KUNDENDATEN\GHOSH\Ghosh.M
Last changed    : 27.08.2021 09:30:14 by SYSTEM
                  (modified after loading)
Sample Info     : 1.0 µL GHS-GB-138-01 (1 µL in 200 µL n-Heptan)
                  100 mm Zorbax RX SIL, 4.6 mm i.D.,
                  n-Heptan / 2-Propanol = 99.9:0.1 (v/v)
                  1.0 mL / min, 13.9 MPa, 308 K
                  UV, 220 nm
                  HC peak based 30 mAu
                  150 mm Chiralcel OD-3, 4.6 mm i.D.
                  n-Heptan/2-Propanol = 99.8:0.2 (v/v)
                  1.0 mL/min, 8.3 MPa, 298K
                  UV, 220 nm
```

Additional Info : Peak(s) manually integrated

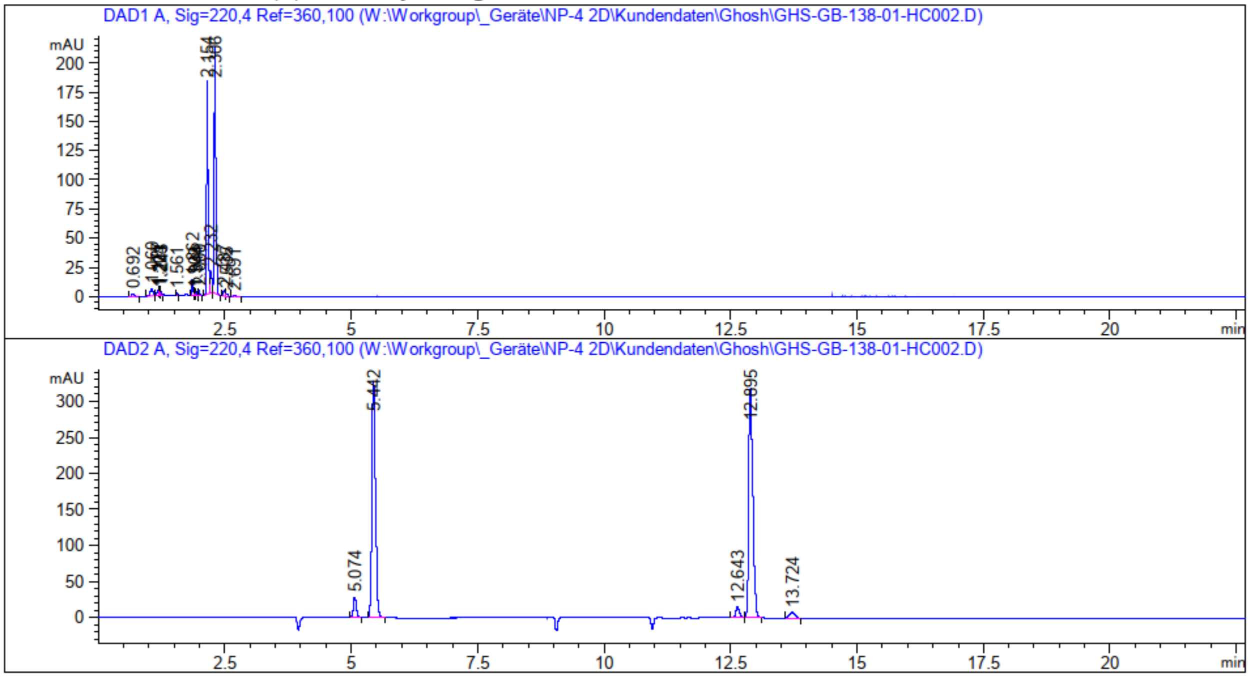

Area Percent Report

Sorted By : Signal
Multiplier : 1.0000
Dilution : 1.0000
Do not use Multiplier & Dilution Factor with ISTDs

Signal 1: DAD1 A, Sig=220,4 Ref=360,100

Peak #	RetTime [min]	Type	Width [min]	Area [mAU*s]	Height [mAU]	Area %	
1	0.692	BB	0.0564	9.16277	2.40580	0.8667	
2	1.060	BB	0.0534	23.46656	6.23052	2.2198	
3	1.177	BV	0.0329	9.72698	4.65103	0.9201	
4	1.204	VV	0.0166	4.08005	3.51145	0.3859	
5	1.223	VV	0.0103	2.01658	2.82141	0.1908	
6	1.243	VB	0.0193	5.36064	4.09854	0.5071	
7	1.561	VV	0.0240	3.07797	1.99699	0.2912	
8	1.862	BV	0.0269	23.53314	13.77001	2.2261	
9	1.903	VV	0.0214	2.93986	2.02507	0.2781	
10	1.944	VV	0.0298	6.09142	3.12343	0.5762	
11	2.000	VB	0.0287	8.86743	4.76463	0.8388	
12	2.154	BV	0.0332	395.46152	182.76651	37.4078	1. diastereomer
13	2.232	VB	0.0319	36.65110	18.24439	3.4669	
14	2.306	BB	0.0371	507.00122	210.28258	47.9587	2. diastereomer
15	2.487	BV	0.0372	11.00550	4.47433	1.0410	
16	2.535	VB	0.0320	4.28675	2.04013	0.4055	
17	2.691	BB	0.0568	4.43353	1.04952	0.4194	
Totals :				1057.16303	468.25633		

Signal 2: DAD2 A, Sig=220,4 Ref=360,100

Peak #	RetTime [min]	Type	Width [min]	Area [mAU*s]	Height [mAU]	Area %	
1	5.074	VB	0.0629	117.07150	27.90473	3.1883	1. diastereomer 1. enantiomer
2	5.442	BV	0.0777	1627.54785	327.63040	44.3238	1. diastereomer 2. enantiomer
3	12.643	VB	0.0734	78.28954	15.37569	2.1321	2. diastereomer 1. enantiomer
4	12.895	BV	0.0870	1793.87512	316.87723	48.8535	2. diastereomer 2. enantiomer
5	13.724	VV	0.0831	55.16455	8.16438	1.5023	
Totals :				3671.94857	695.95242		

HPLC Trace of Racemic (7k)

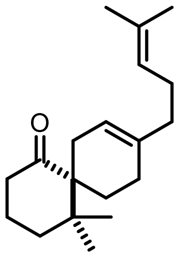

7k

Column: Amycoat R; **Solvent**: MeCN/H_2O = 70/30; **Flow Rate**: 1.0 mL/min

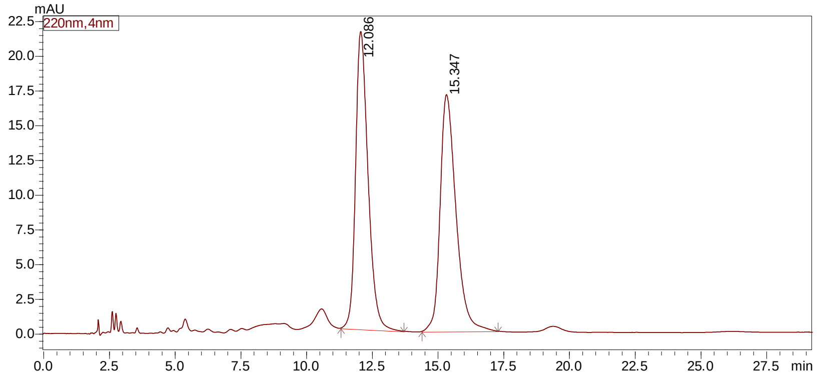

Peak#	Ret. Time	Area%
1	12.09	49.736
2	15.35	50.264
Total		100

HPLC Trace of Enantiopure (7k)

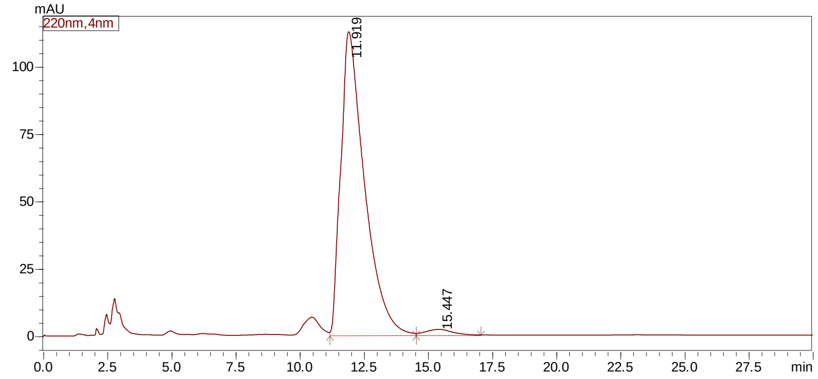

Peak#	Ret. Time	Area%
1	11.92	97.982
2	15.45	2.018
Total		100

HPLC Trace of Racemic (7l) and (7l')

Column: AD-3R **Solvent**: 40:60 CH_3CN:H_2O, **Flow rate**: 1 mL/min

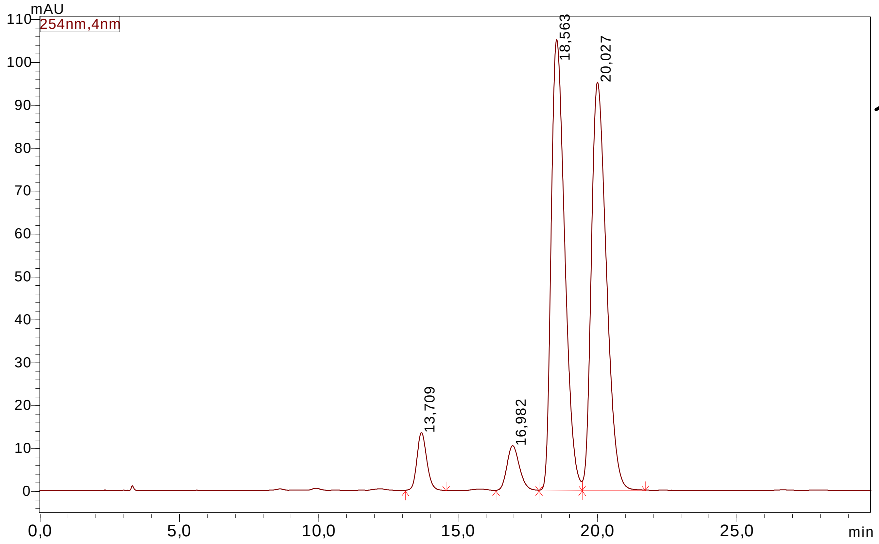

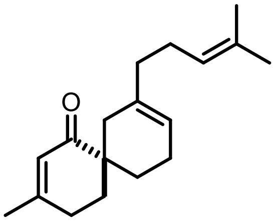

7l'

Peak#	Ret. Time	Area%	
1	13.709	4.234	E1/R1
2	16.982	4.148	E2/R1
3	18.563	45.655	E1/R2
4	20.027	45.963	E2/R2
Total		100	

HPLC Trace of Enantiopure (7l')

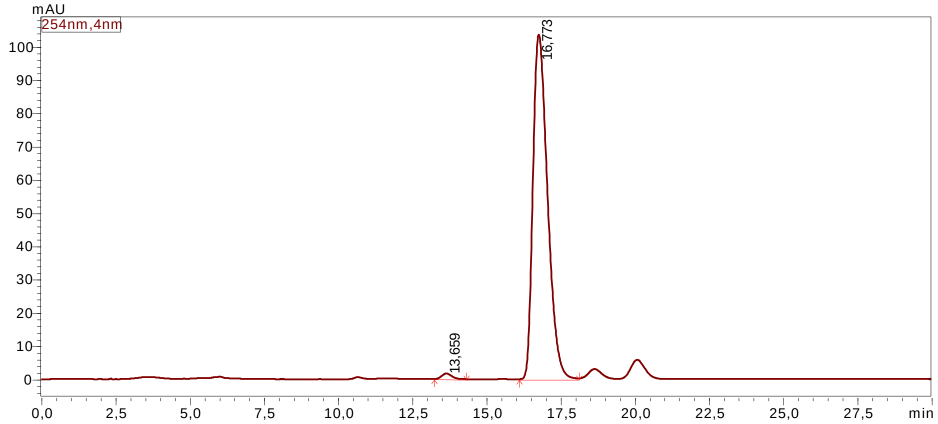

Peak#	Ret. Time	Area%	
1	13.66	1.106	E1/R1
2	16.77	98.894	E2/R1
Total		100	

HPLC Trace of Racemic (**7m**) **and** (**7m'**)

Column: OJ-3R; **Solvent**: MeCN/H_2O = 45/55; **Flow Rate**: 1.0 mL/min

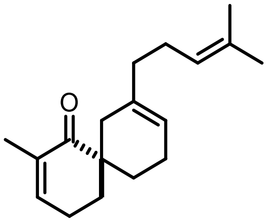

7m'

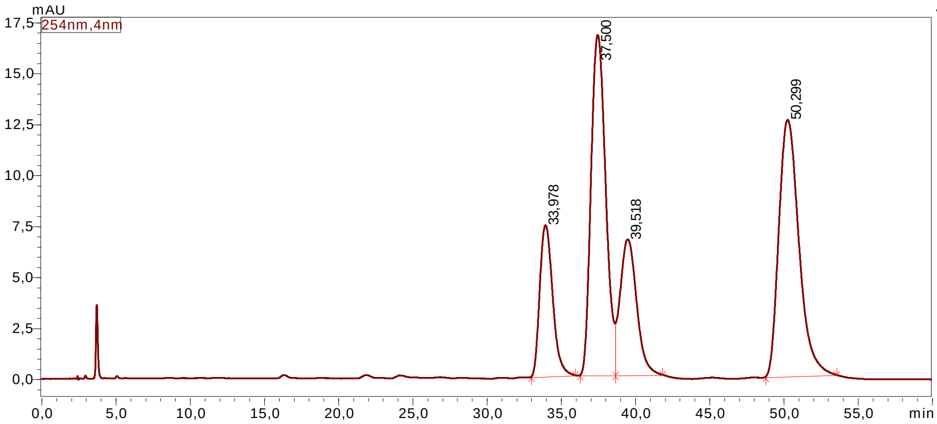

Peak#	Ret. Time	Area%	
1	33.98	13.615	E1/R1
2	37.50	34.710	E1/R2
3	39.52	15.774	E2/R1
4	50.30	35.900	E2/R2
Total		100	

HPLC Trace of Enantiopure (**7m'**)

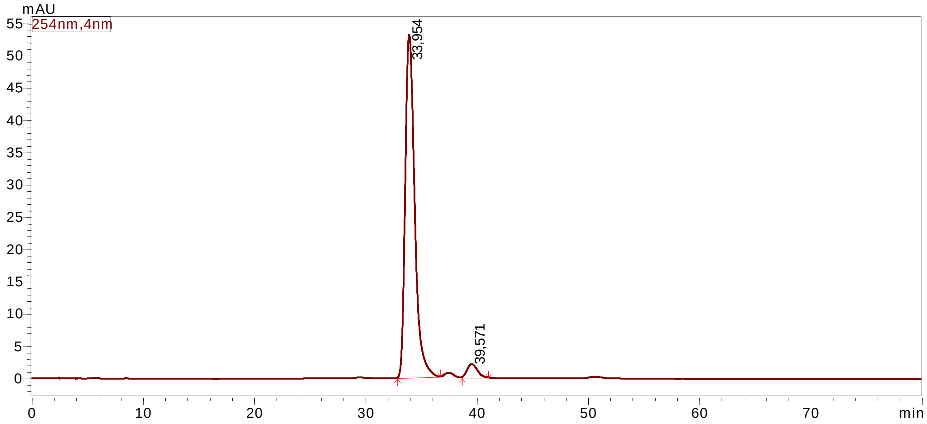

Peak#	Ret. Time	Area%	
1	33.95	96.112	E1/R1
3	39.57	3.888	E2/R1
Total		100	

GC Trace of Racemic (7n) and (7n')

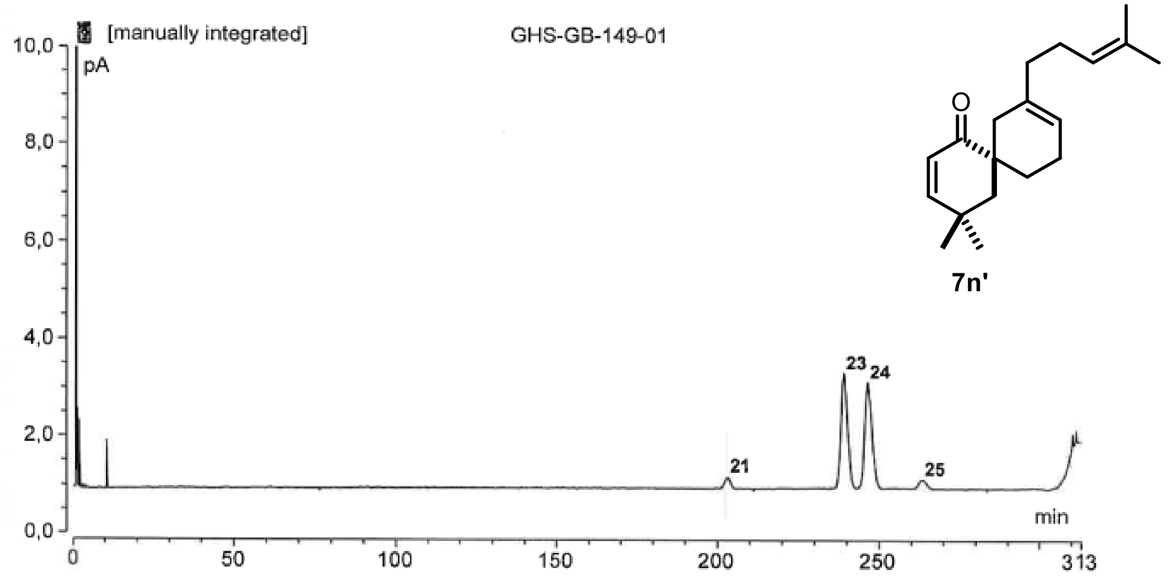

Sample:	**GHS-GB-149-01**	Instrument:	**GC_412**
Sequenz:	**8249 GHS-GB PH**	Measured:	**27.08.21 08:42**
Sequenz date:	**26.08.21**	Processing M.:	GHS-GB-149
		Report-File:	Verhältnis

Razemat
Zuordnung nach achiral Messung

No.	Ret.Time min	Rel.Area %	Peak Name
21	203,13	3,76	.
23	239,10	46,31	..
24	246,46	46,07	..
25	263,51	3,86	.

Instrument parameters:

Column:	24,0 m	Cycloextrin-H 0.25/0.125df;G/632
Temperature:	220 / 120, 300 min iso 6/min 180, 3 min iso / 350	
Gas:	0,50 bar	Hydrogen
Sample size:	0,2 µL	

GC Trace of Enantiopure (7n')

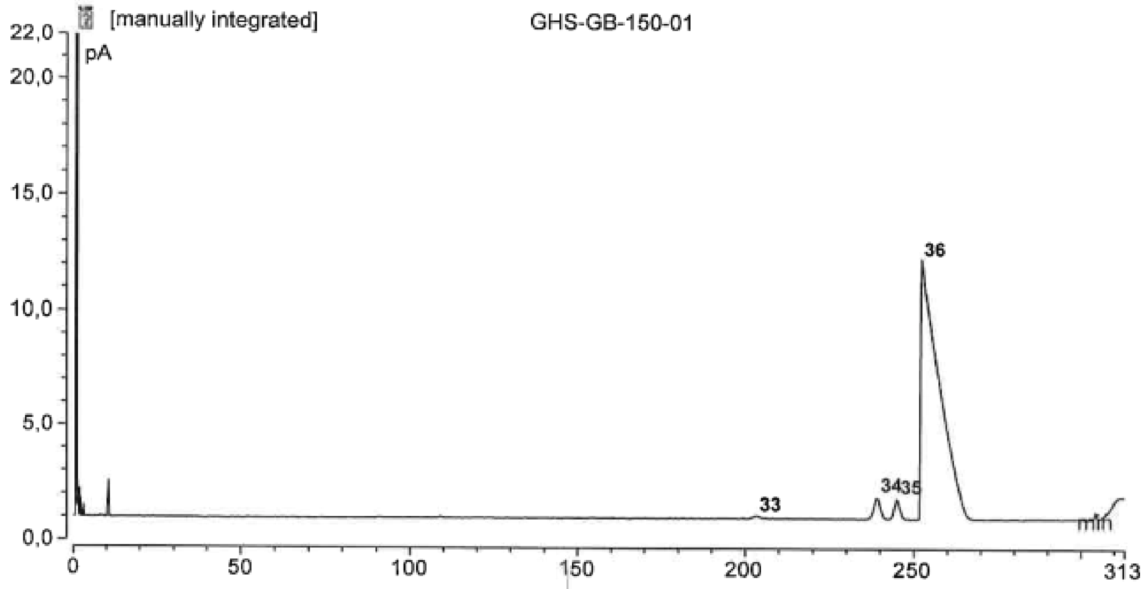

Sample:	**GHS-GB-150-01**	Instrument:	**GC_412**
Sequenz:	**8249 GHS-GB PH**	Measured:	**30.08.21 08:08**
Sequenz date:	**26.08.21**	Processing M.:	GHS-GB-149
		Report-File:	Verhältnis

Enantiomerenverhältnis
Zuordnung nach achiral Messung

No.	Ret.Time min	Rel.Area %	Peak Name
33	203,55	0,36	.
34	239,14	2,58	..
35	244,97	1,98	..
36	252,44	95,08	.

Instrument parameters:

Column:	24,0 m	Cycloextrin-H 0.25/0.125df;G/632
Temperature:	220 / 120, 300 min iso 6/min 180, 3 min iso / 350	
Gas:	0,50 bar	Hydrogen
Sample size:	0,2 µL	

Peak#	Ret. Time	Area%	
1	203.55	0.36	E1/R1
3	252.44	95.08	E2/R1
Total		95.44	

e.r. > 99.5:0.5

HPLC Trace of Racemic (7o')

Column: AD-3R; **Solvent**: MeCN/H_2O = 70/30; **Flow Rate**: 1.0 mL/min

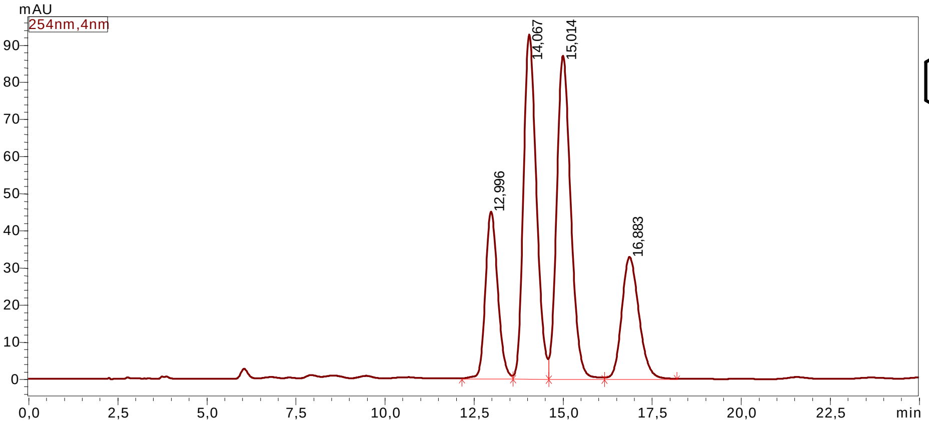

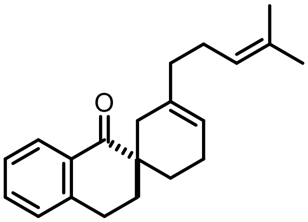

7o'

Peak#	Ret. Time	Area%	
1	13.0	15.622	E1/R1
2	14.07	33.960	E1/R2
3	15.01	34.531	E2/R2
4	16.88	15.886	E2/R1
Total		100	

HPLC Trace of Enantiopure (7o')

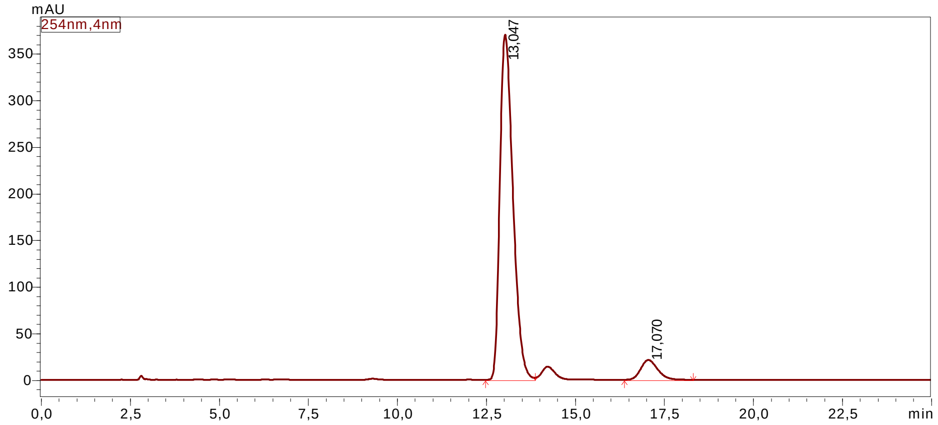

Peak#	Ret. Time	Area%	
1	13.05	93.135	E1/R1
4	17.07	6.865	E2/R1
Total		100	

HPLC Trace of Racemic (7p) (*endo*- and *exo*-)

Column: AD-3R **Solvent**: 40:60 $CH_3CN:H_2O$, **Flow rate**: 1 mL/min

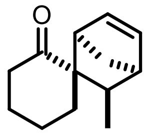

7p

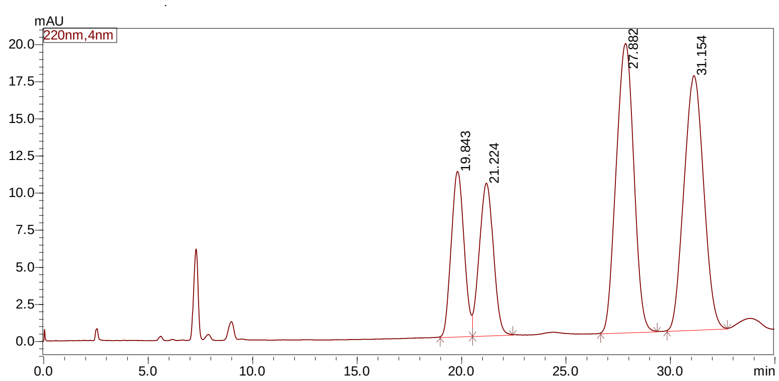

Peak#	Ret. Time	Area%	
1	19.843	14.678	E1/*endo*
2	21.224	14.960	E2/ *endo*
3	27.882	35.427	E1/*exo*
4	31.154	34.936	E2/ *exo*
Total		100	

HPLC Trace of Enantiopure (7p) (*exo* isomer)

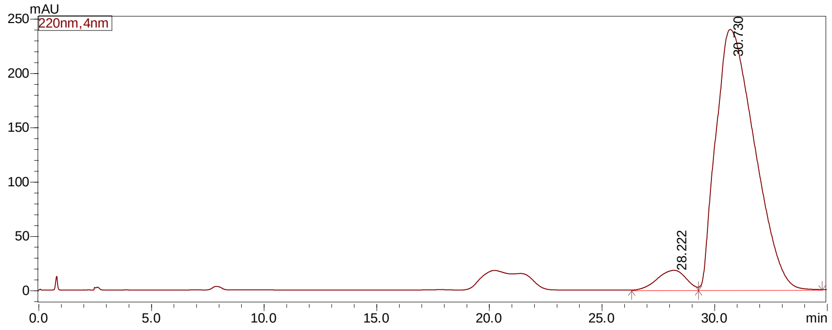

Peak#	Ret. Time	Area%	
3	28.222	5.267	E1/*exo*
4	30.730	94.733	E2/ *exo*
Total		100	

GC Trace of Racemic (7q)

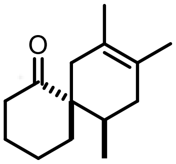

7q

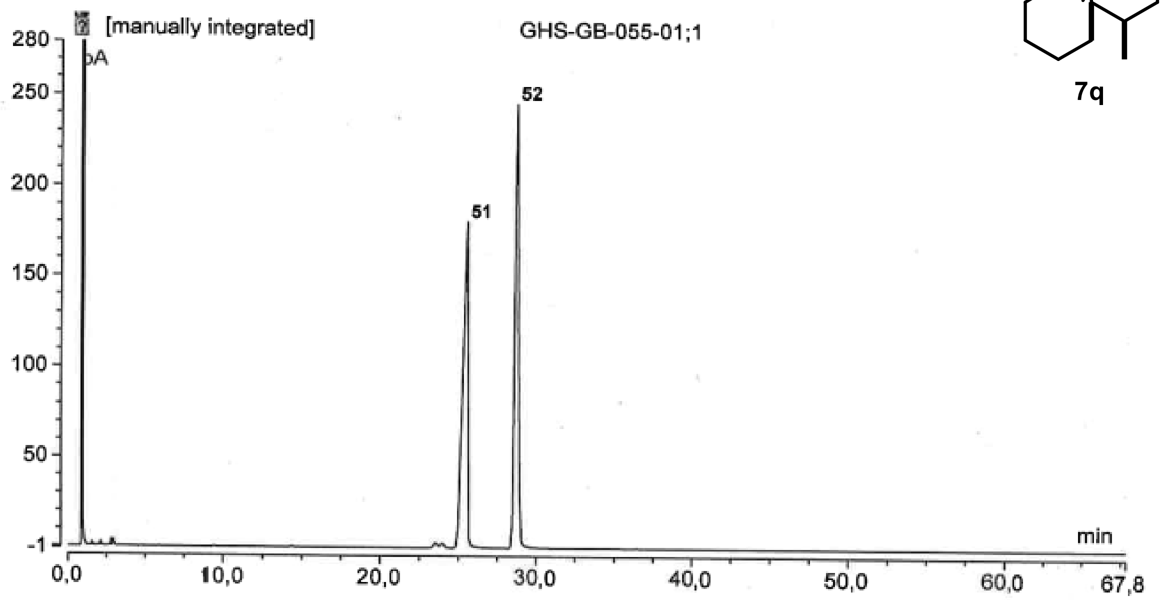

Sample:	**GHS-GB-055-01;1**	Instrument:	**GC_112**
Sequenz:	**8015 GHS-GB SE**	Measured:	**10.02.21 08:53**
Sequenz date:	**10.02.21**	Processing M.:	GHS
		Report-File:	Verhältnis 055

Racemat
Verhältnis der Enantiomere
Zuordnung achiral nach GCMS 29567 GHS-GB-055-01 21/8015

No.	Ret.Time min	Rel.Area %	Peak Name
51	25,57	49,99	.
52	28,75	50,01	.

Instrument parameters:

Column:	25,0 m	Hydrodex-gamma-TBDAc-CD 0,25/?df G/624
Temperature:	220/120 iso/350	
Gas:	0,60 bar	H2
Sample size:	0,2 µL	

GC Trace of Enantiopure (7q)

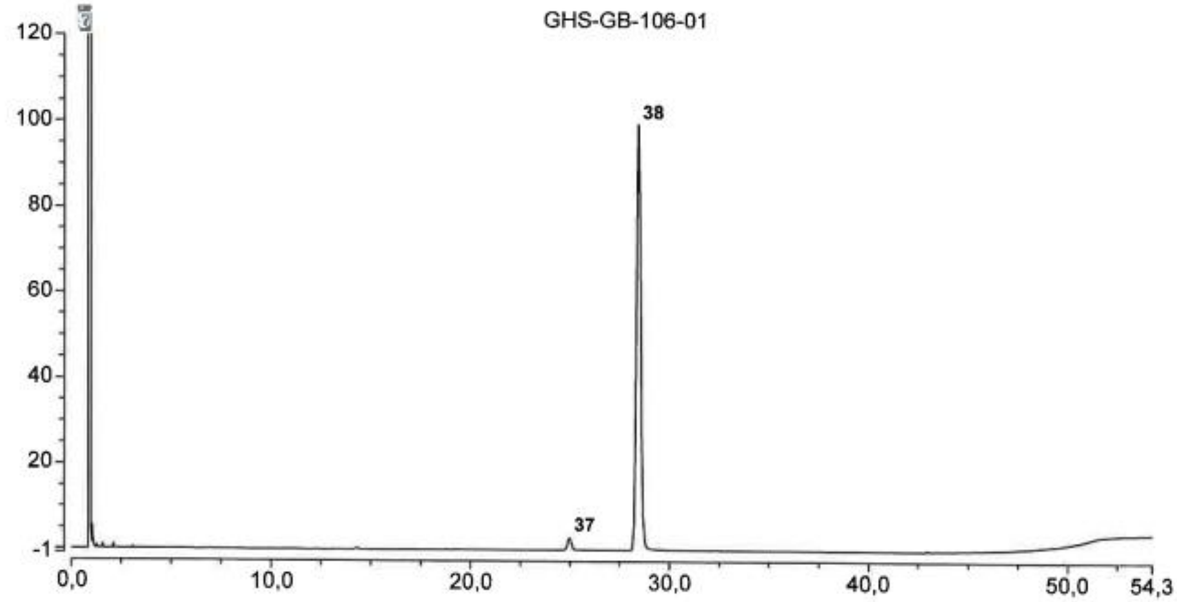

Sample:	**GHS-GB-106-01**	Instrument:	**GC_112**
Sequenz:	**8185 GHS-GB SE**	Measured:	**13.04.21 08:29**
Sequenz date:	**13.04.21**	Processing M.:	GHS
		Report-File:	Verhältnis 106

Verhältnis der Enantiomere
Zuordnung achiral nach GCMS 29567 GHS-GB-055-01 21/8015

No.	Ret.Time min	Rel.Area %	Peak Name
37	24,97	2,52	.
38	28,42	97,48	.

Instrument parameters:

Column:	25,0 m	Hydrodex-gamma-TBDAc-CD 0,25/?df G/624
Temperature:	220/120, 35 min iso 8/min 250, 3 min iso/350	
Gas:	0,60 bar	H2
Sample size:	0,2 µL	

GC Trace of Racemic (7r)

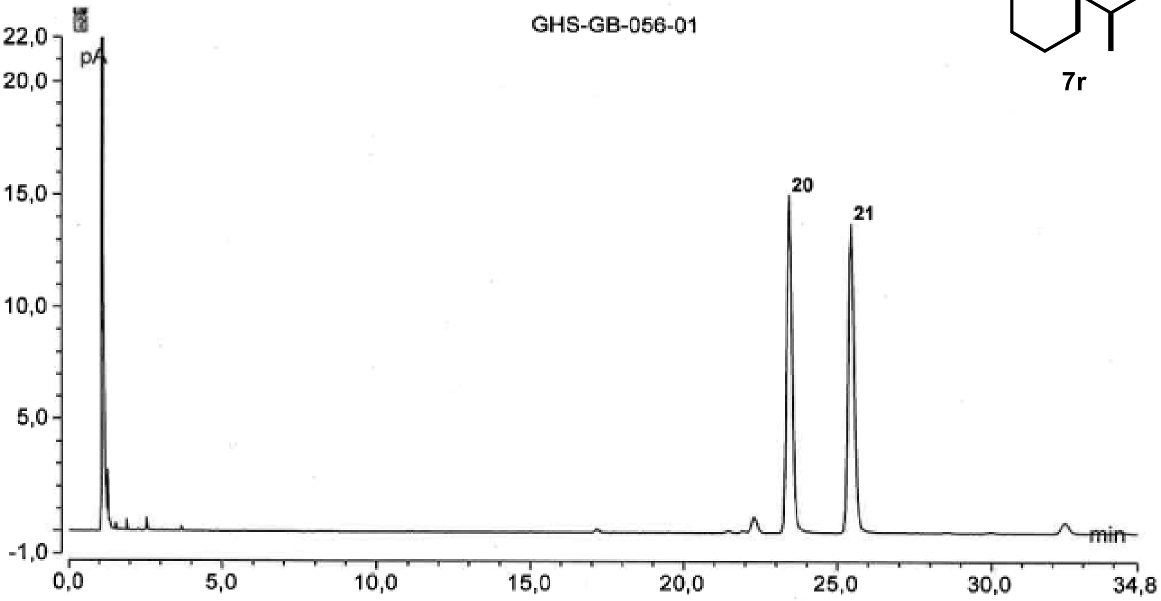

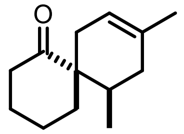

7r

Sample: **GHS-GB-056-01**
Sequenz: **8016 GHS-GB PH**
Sequenz date: **15.02.21**

Instrument: **GC_112**
Measured: **16.02.21 08:52**
Processing M.: MPI
Report-File: Verhältnis

Razemat
Zuordnung nach achiral Messung

No.	Ret.Time min	Rel.Area %	Peak Name
20	23,44	50,01	.
21	25,45	49,99	.

Instrument parameters:

Column:	25,0 m	Hydrodex-gamma-TBDAc-CD 0,25/?df G/624
Temperature:	220 / 120 iso / 350	
Gas:	0,50 bar	H2
Sample size:	0,2 µL	

HPLC Trace of Enantiopure (7r)

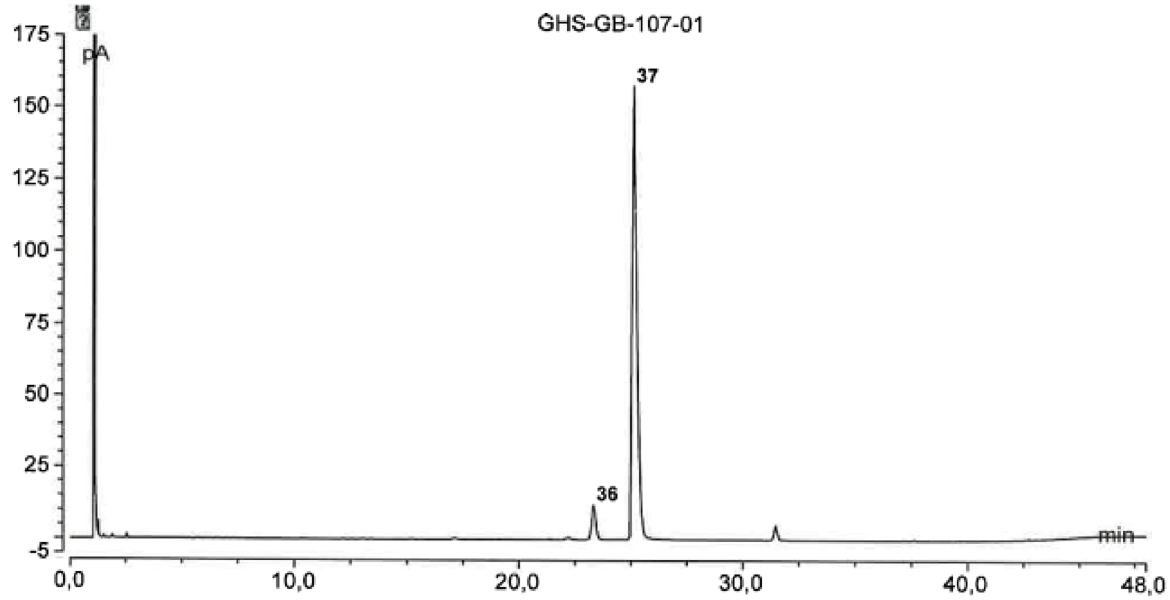

Sample:	**GHS-GB-107-01**	Instrument:	**GC_112**
Sequenz:	**8195 GHS-GB PH**	Measured:	**14.04.21 11:45**
Sequenz date:	**14.04.21**	Processing M.:	GHS 56
		Report-File:	Verhältnis

Enantiomerenverhältnis
Zuordnung nach achiral Messung

No.	Ret.Time min	Rel.Area %	Peak Name
36	23,35	6,06	.
37	25,13	93,94	.

Instrument parameters:

Column:	25,0 m	Hydrodex-gamma-TBDAc-CD 0,25/?df G/624
Temperature:	220 / 120, 30 min iso 8/min 240, 3 min iso / 350	
Gas:	0,50 bar	H2
Sample size:	0,2 µL	

GC Trace of Racemic (13a)

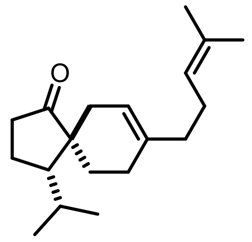

13a

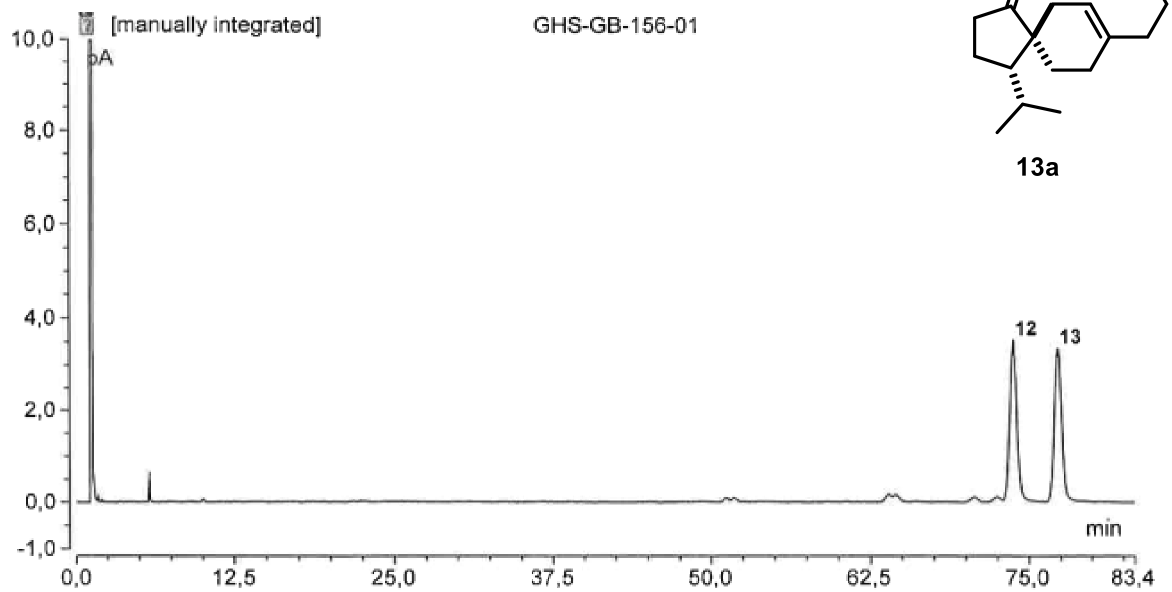

Sample:	**GHS-GB-156-01**	Instrument:	**GC_112**
Sequenz:	**8389 GHS-GB PH**	Measured:	**17.08.21 08:48**
Sequenz date:	**16.08.21**	Processing M.:	GHS
		Report-File:	Verhältnis

Razemat

Zuordnung nach achiral Messung

No.	Ret.Time min	Rel.Area %	Peak Name
12	73,77	50,24	.
13	77,30	49,76	.

Instrument parameters:

Column:	25,0 m	Hydrodex-gamma-TBDAc-CD 0,25/?df G/624
Temperature:	220 / 150 iso / 350	
Gas:	0,50 bar	H2
Sample size:	0,2 µL	

GC Trace of Enantiopure (13a)

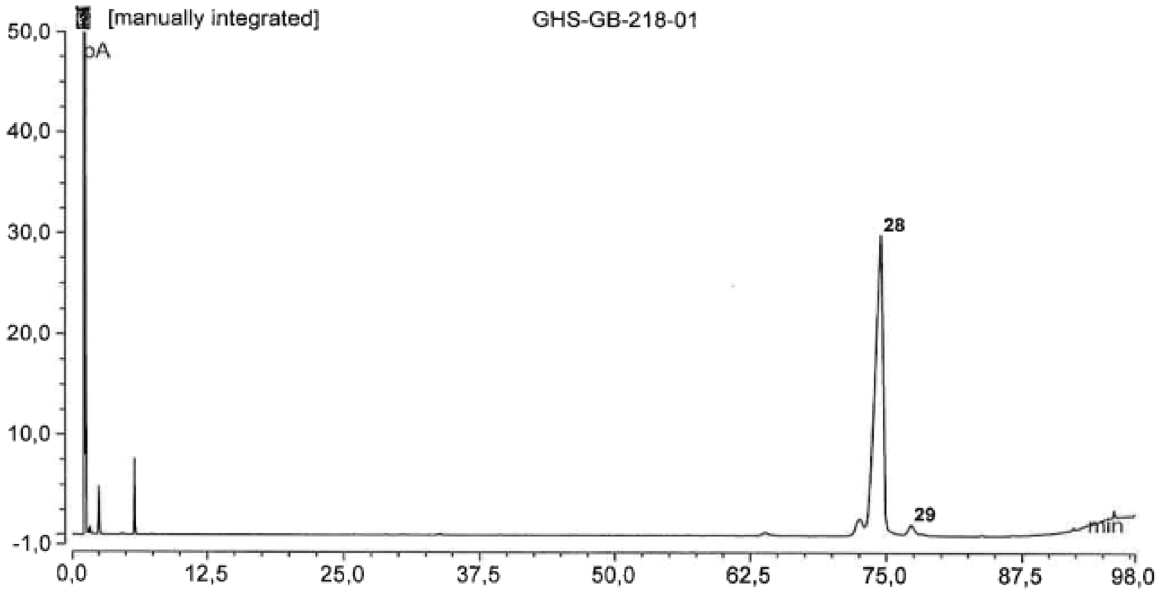

Sample:	**GHS-GB-218-01**	Instrument:	**GC_112**
Sequenz:	**8421 GHS-GB PH**	Measured:	**02.09.21 14:24**
Sequenz date:	**02.09.21**	Processing M.:	GHS
		Report-File:	Verhältnis

Enantiomerenverhältnis
Zuordnung nach achiral Messung

No.	Ret.Time min	Rel.Area %	Peak Name
28	74,54	97,66	.
29	77,30	2,34	.

Instrument parameters:

Column:	25,0 m	Hydrodex-gamma-TBDAc-CD 0,25/?df G/624
Temperature:	220 8 150, 80 min iso 6/min 240, 3 min iso / 350	
Gas:	0,50 bar	H2
Sample size:	0,2 µL	

GC Trace of Racemic (13b)

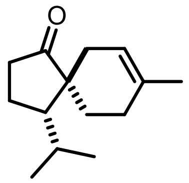

13b

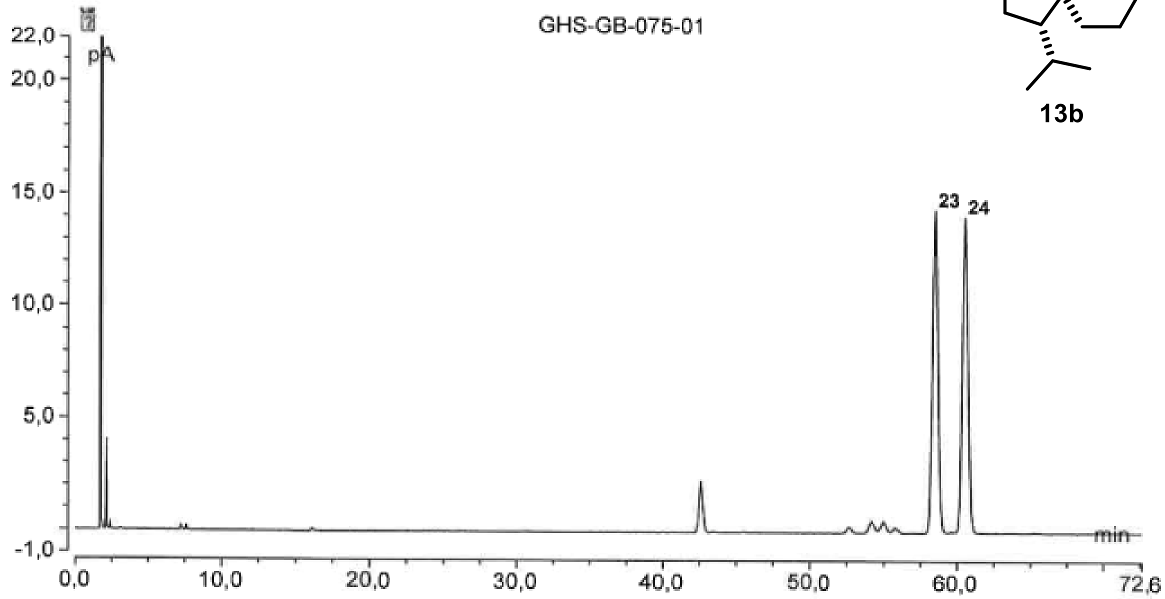

Sample:	**GHS-GB-075-01**	Instrument:	**GC_313**
Sequenz:	**8397 GHS-GB PH**	Measured:	**01.09.21 10:41**
Sequenz date:	**26.08.21**	Processing M.:	MPI
		Report-File:	Verhältnis

Razemat
Zuordnung nach achiral Messung

No.	Ret.Time min	Rel.Area %	Peak Name
23	58,52	50,05	.
24	60,56	49,95	.

Instrument parameters:

Column:	30,0 m	BGB-176/BGB-15 0,25/0,25df G/618
Temperature:	220 / 120 iso / 350	
Gas:	0,60 bar	N2
Sample size:	1,0 µL	

GC Trace of Enantiopure (13b)

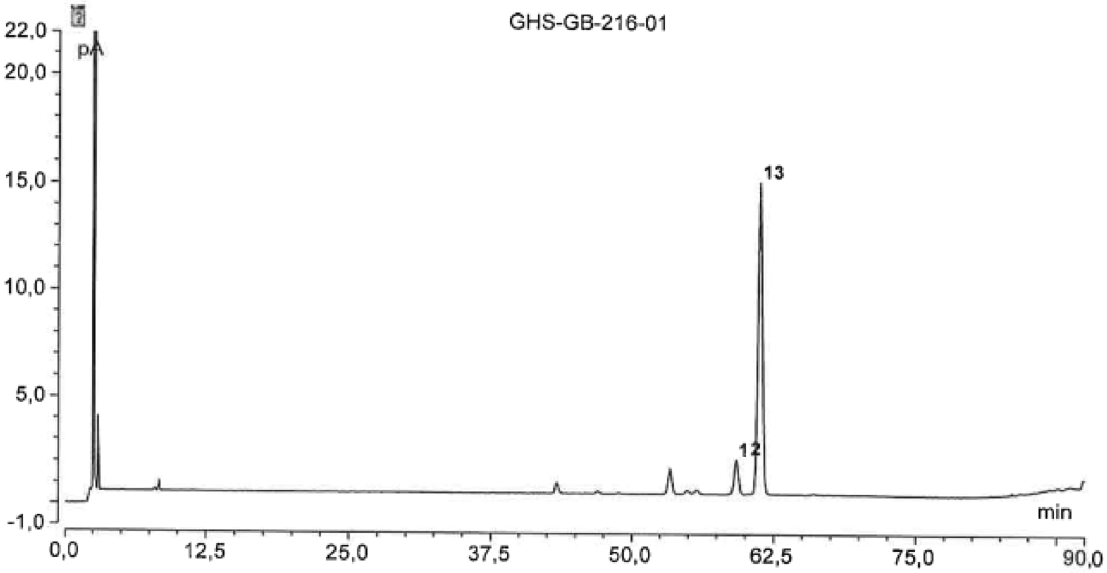

Sample:	**GHS-GB-216-01**	Instrument:	**GC_313**
Sequenz:	**8422 GHS-GB PH**	Measured:	**02.09.21 16:03**
Sequenz date:	**02.09.21**	Processing M.:	MPI
		Report-File:	Verhältnis

Enantiomerenverhältnis
Zuordnung nach achiral Messung

No.	Ret.Time min	Rel.Area %	Peak Name
12	59,23	9,26	.
13	61,29	90,74	.

Instrument parameters:

Column:	30,0 m	BGB-176/BGB-15 0,25/0,25df G/618
Temperature:	220 / 120, 70 min iso 6/min 220, 3 / 350	
Gas:	0,60 bar	H2
Sample size:	0,2 µL	

HPLC Trace of Racemic (12)

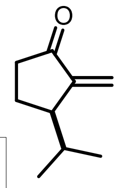

12

Column: AD-3R; **Solvent**: MeCN/H_2O = 50/50; **Flow Rate**: 1.0 mL/min

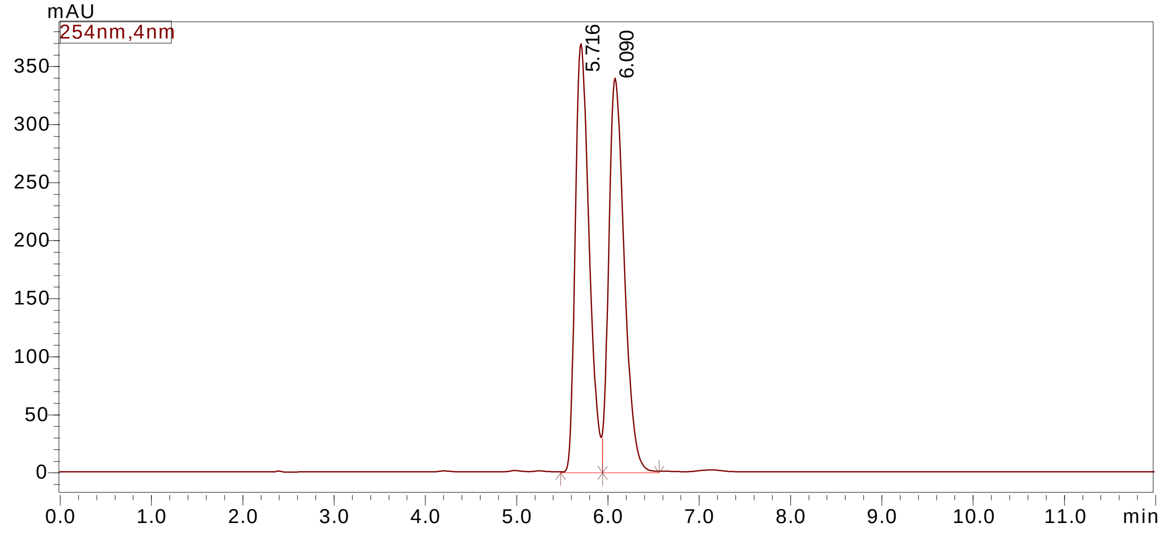

Peak#	Ret. Time	Area%
1	5.72	49.370
2	6.09	50.630
Total		100

HPLC Trace of Enantiopure (12) obtained after the reaction with diene (6a)

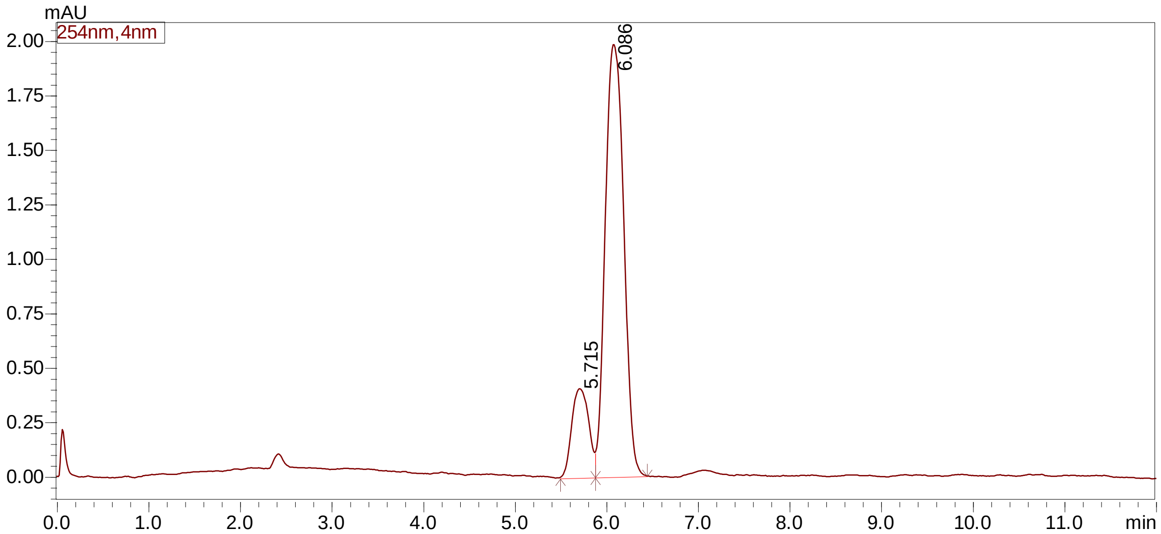

Peak#	Ret. Time	Area%
1	5.72	15.808
2	6.09	84.192
Total		100

HPLC Trace of Enantiopure 12 obtained after the reaction with diene (6d)

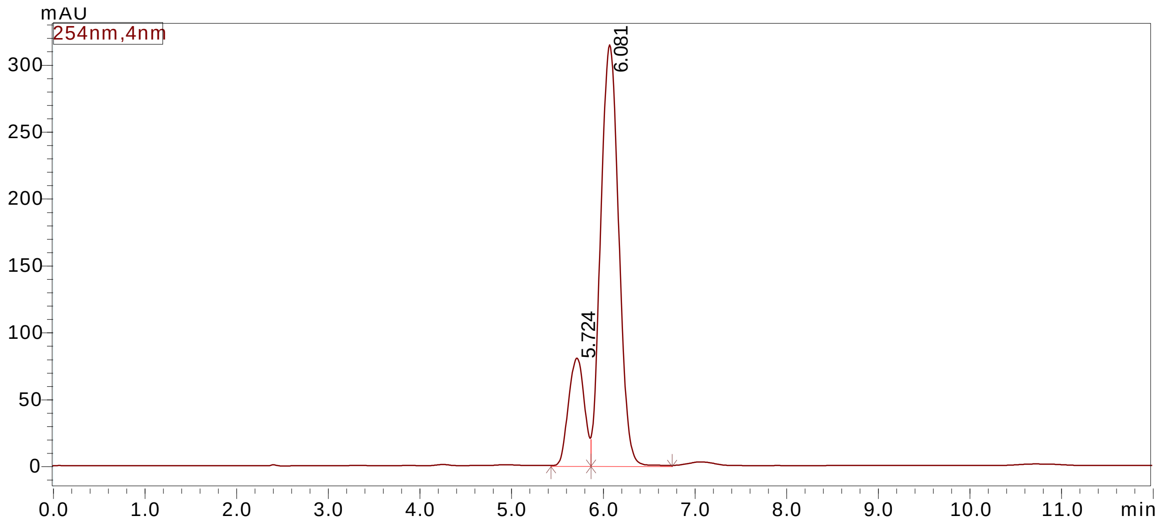

Peak#	Ret. Time	Area%
1	5.72	18.289
2	6.08	81.711
Total		100

GC Trace of Racemic (14)

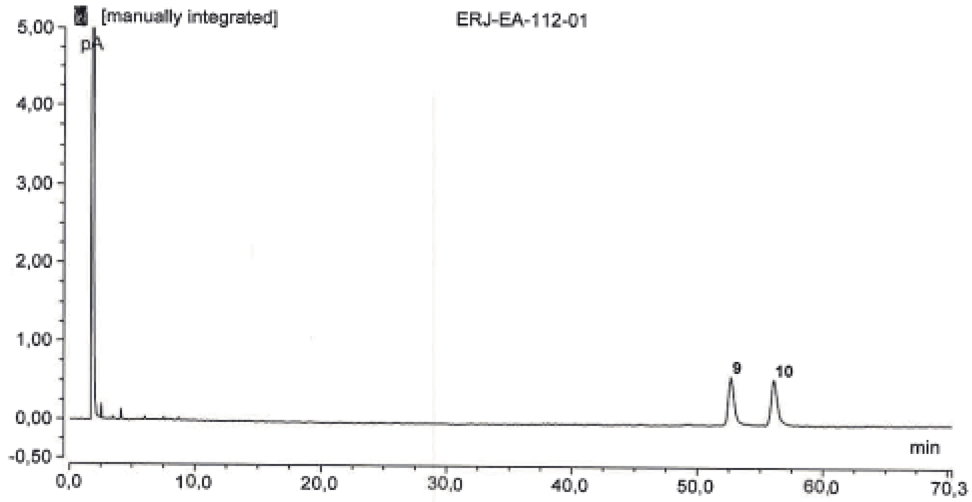

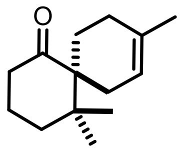

14

Sample:	**ERJ-EA-112-01**	Instrument:	**GC_213**
Sequenz:	**7872 ERJ-EA PH**	Measured:	**09.12.20 13:16**
Sequenz date:	**09.12.20**	Processing M.:	ERJ 112
		Report-File:	Verhältnis

Razemat
Zuordnung achiral nach GCMS:28823

No.	Ret.Time min	Rel.Area %	Peak Name
9	52,68	50,46	.
10	56,06	49,54	.

Instrument parameters:

Column:	30,0 m	G-TA 0,25/?df ; G/448
Temperature:	220 / 110 iso / 350	
Gas:	0,60 bar	H2
Sample size:	1,0 µL	

GC Trace of Enantiopure (14)

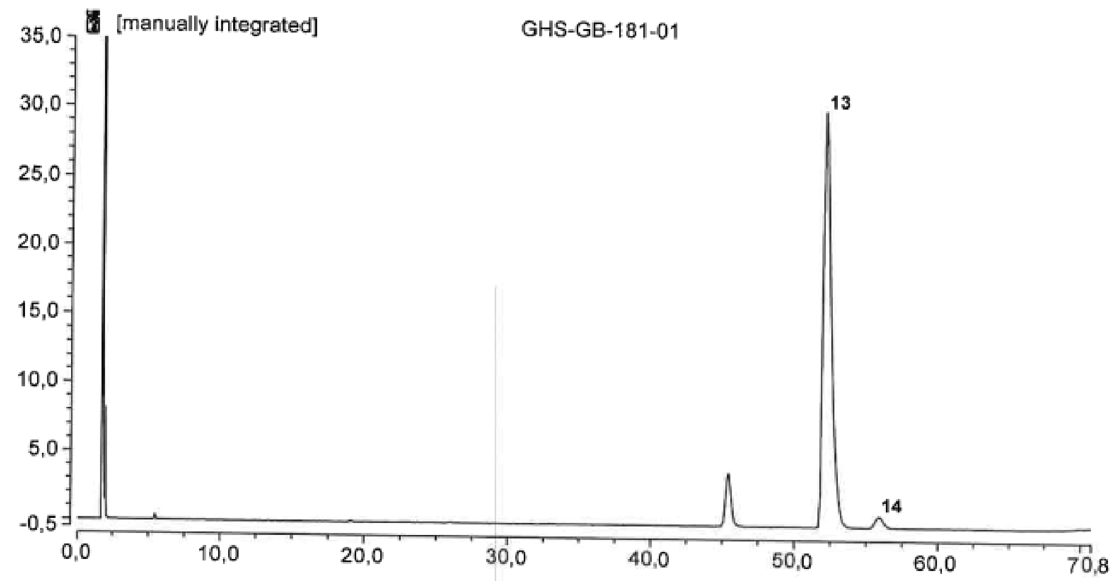

Sample:	**GHS-GB-181-01**	Instrument:	**GC_213**
Sequenz:	**8324 GHS-GB MM**	Measured:	**13.07.21 12:18**
Sequenz date:	**13.07.21**	Processing M.:	181-01
		Report-File:	Verhältnis 181-01

chirale Messung der Probe, Verhältnis der Enantiomere
Zuordnung nach achiraler Messung, Vergleich mit Racemat ERJ-EA-112-01 20/7872

No.	Ret.Time min	Rel.Area %	Peak Name
13	52,07	97,40	.
14	55,90	2,60	.

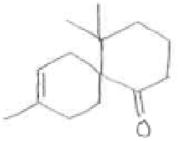

Instrument parameters:

Column:	30,0 m	G-TA 0,25/?df ; G/448
Temperature:	220/ 110, 62min iso 8/min 180/ 350	
Gas:	0,60 bar	H2
Sample size:	0,2 µL	

GC Trace of Enantiopure (14) (After Preparative HPLC Separation)

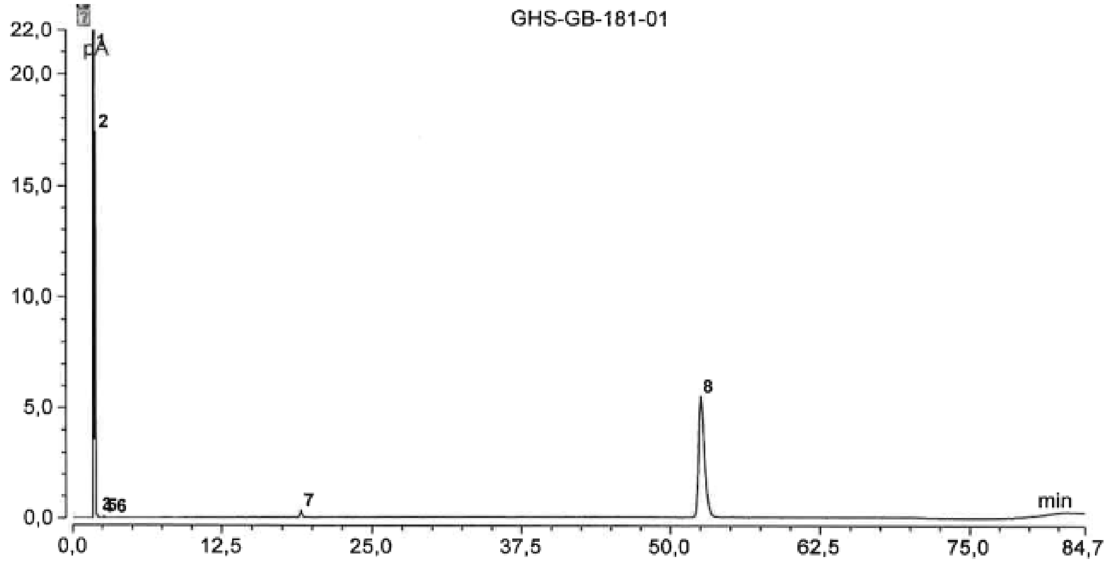

Sample:	**GHS-GB-181-01**	Instrument:	**GC_213**
Sequenz:	**8409 GHS-GB PH**	Measured:	**26.08.21 11:23**
Sequenz date:	**26.08.21**	Processing M.:	MPI
		Report-File:	Verhältnis

Enantiomerenverhältnis

Zuordnung nach Racematvergleich

Keine Spuren des zweiten Enantiomers gefunden, EE 100% ist anzunehmen

**No.**	**Ret.Time min**	**Rel.Area %**	**Peak Name**
8	52,55	100,00	.

Instrument parameters:

Column:	30,0 m	G-TA 0,25/?df ; G/448
Temperature:	220 / 110, 70 min iso 6/min 180, 3 min / 350	
Gas:	0,60 bar	H2
Sample size:	1,0 µL	

GC Trace of Racemic (4b)

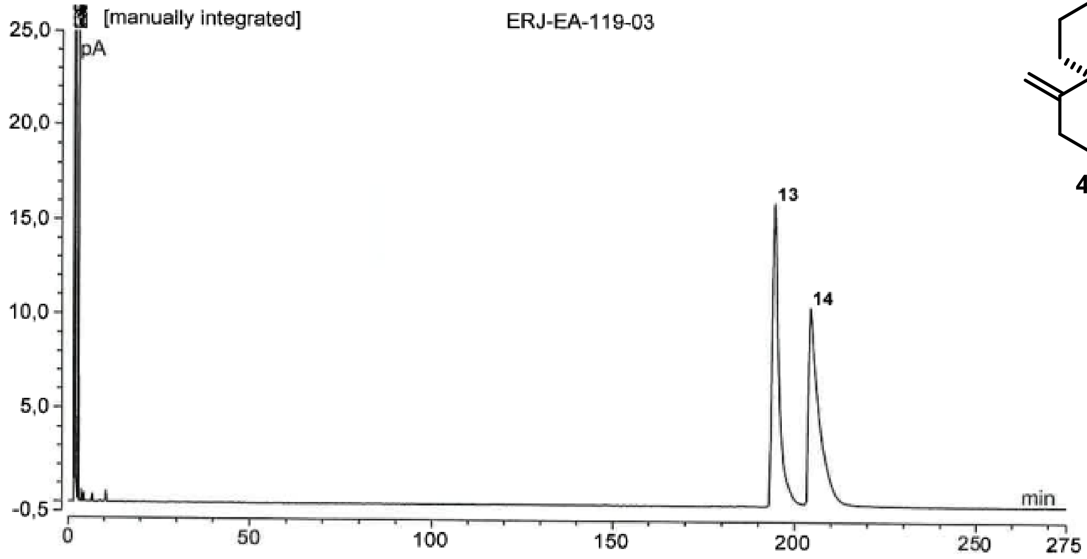

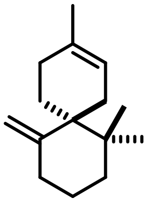

4b

Sample:	**ERJ-EA-119-03**	Instrument:	**GC_213**
Sequenz:	**7932 ERJ-EA PH**	Measured:	**19.01.21 08:34**
Sequenz date:	**18.01.21**	Processing M.:	ERJ 119
		Report-File:	Verhältnis

Enantiomerenverhältnis
Zuordnung achiral nach GCMS:29147

No.	Ret.Time min	Rel.Area %	Peak Name
13	194,63	49,63	.
14	204,32	50,37	.

Instrument parameters:

Column:	30,0 m	G-TA 0,25/?df ; G/448
Temperature:	220 / 60 iso / 350	
Gas:	0,60 bar	H2
Sample size:	1,0 µL	

GC Trace of Enantiopure (4b)

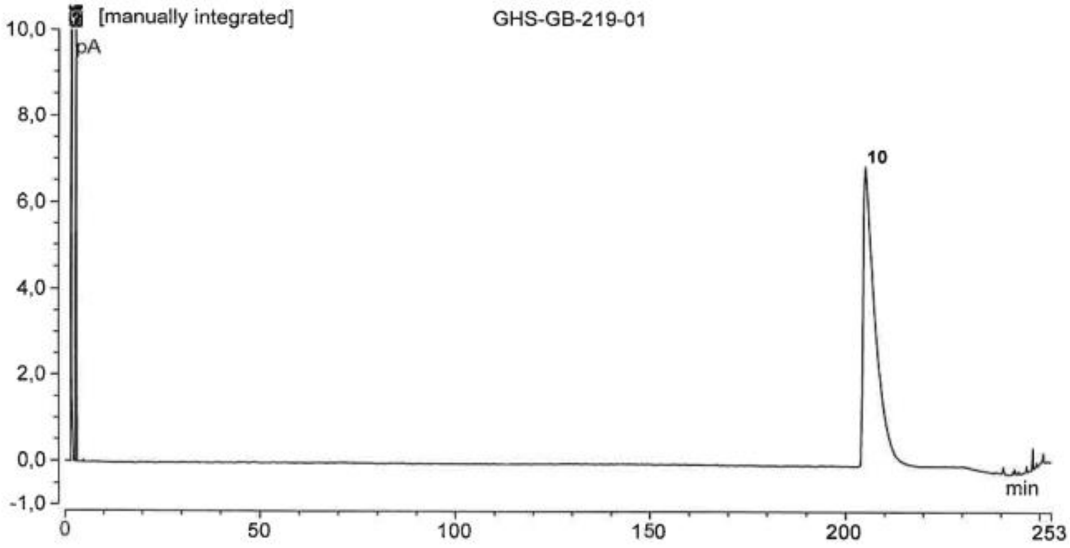

Sample:	**GHS-GB-219-01**	Instrument:	**GC_213**
Sequenz:	**8424 GHS-GB PH**	Measured:	**02.09.21 14:26**
Sequenz date:	**02.09.21**	Processing M.:	MPI
		Report-File:	Verhältnis

Enantiomerenverhältnis
Zuordnung nach ERJ-EA-119-03 (rac.)

No.	Ret.Time min	Rel.Area %	Peak Name
10	205,13	100,00	.

Instrument parameters:

Column:	30,0 m	G-TA 0,25/?df ; G/448
Temperature:	220 / 60, 230 min iso 6/min 180, 3 min iso / 350	
Gas:	0,60 bar	H2
Sample size:	1,0 µL	

GC Trace of Racemic (4a)

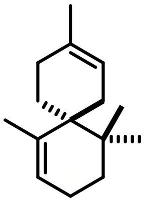

4a

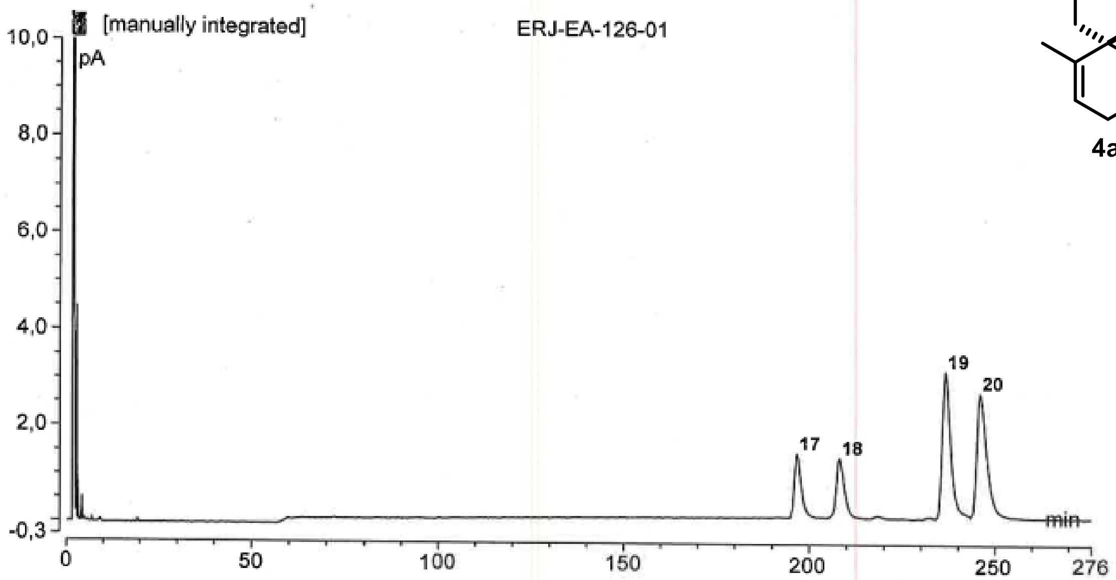

Sample:	**ERJ-EA-126-01**	Instrument:	**GC_213**
Sequenz:	**7957 ERJ-EA PH**	Measured:	**27.01.21 09:16**
Sequenz date:	**27.01.21**	Processing M.:	ERJ 126
		Report-File:	Verhältnis

Razemat
Zuordnung nach achiral GCMS:29147 und GCMS:29336

No.	Ret.Time min	Rel.Area %	Peak Name
17	196,64	13,11 .	
18	208,00	13,50 .	
19	236,55	36,60 ..	
20	245,83	36,80 ..	

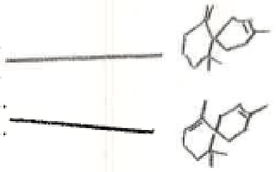

Instrument parameters:

Column:	30,0 m	G-TA 0,25/?df ; G/448
Temperature:	220 / 60 iso / 350	
Gas:	0,60 bar	H2
Sample size:	1,0 µL	

GC Trace of Enantiopure (4a)

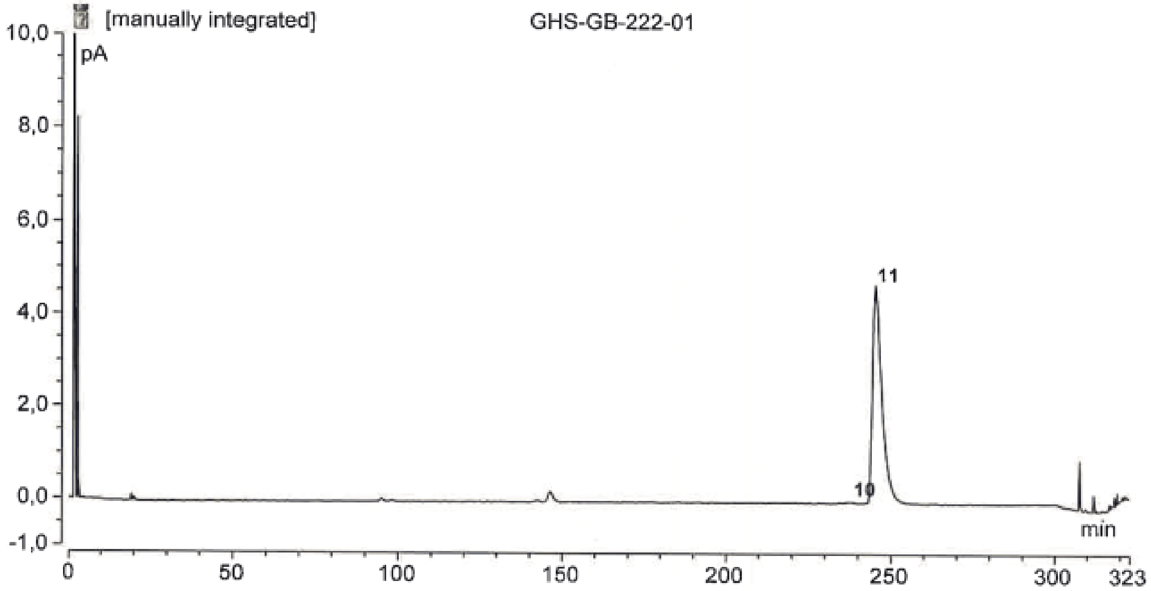

Sample:	**GHS-GB-222-01**	Instrument:	**GC_213**
Sequenz:	**8440 GHS-GB PH**	Measured:	**20.09.21 10:09**
Sequenz date:	**20.09.21**	Processing M.:	MPI
		Report-File:	Verhältnis

Enantiomerenverhältnis
Zurdnung nach Razemat ERJ-EA-126-01

No.	Ret.Time min	Rel.Area %	Peak Name
10	237,68	0,44	.
11	245,31	99,56	.

Instrument parameters:

Column:	30,0 m	G-TA 0,25/?df ; G/448
Temperature:	220 / 60, 300 min iso 6/min 180, 3 min iso / 350	
Gas:	0,60 bar	H2
Sample size:	1,0 µL	

GC Trace of Racemic (4c)

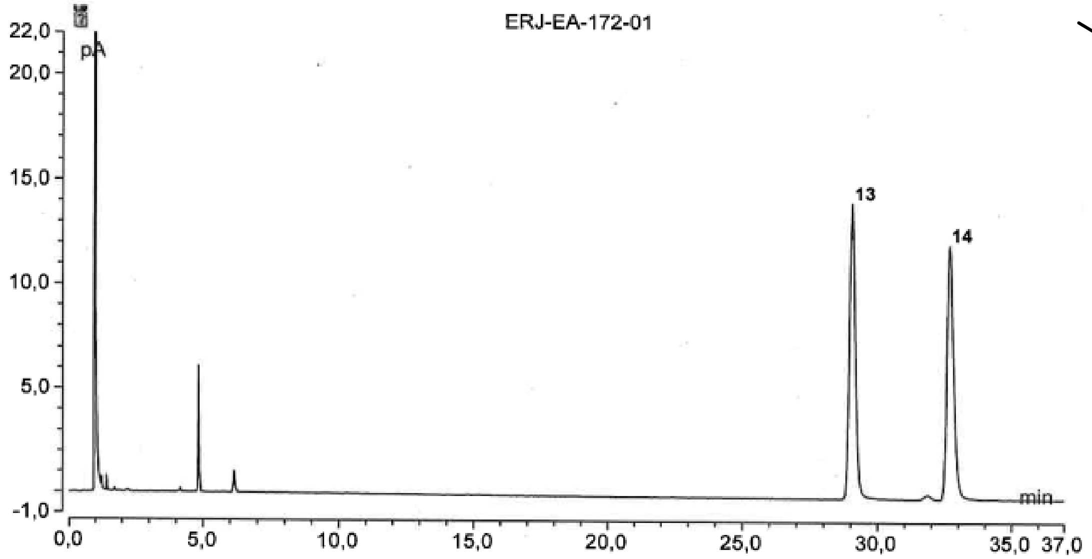

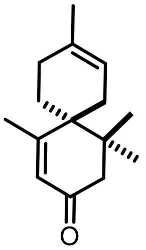

(*ent*- **4c**)

Sample:	**ERJ-EA-172-01**	Instrument:	**GC_112**
Sequenz:	**8009 ERJ-EA PH**	Measured:	**15.02.21 13:45**
Sequenz date:	**12.02.21**	Processing M.:	MPI
		Report-File:	Verhältnis

Razemat

Zuordnung nach achiral Massung

No.	Ret.Time min	Rel.Area %	Peak Name
13	29,07	50,51	.
14	32,69	49,49	.

Instrument parameters:

Column:	25,0 m	Hydrodex-gamma-TBDAc-CD 0,25/?df G/624
Temperature:	220 / 150 iso / 350	
Gas:	0,60 bar	H2
Sample size:	0,2 µL	

GC Trace of Enantiopure (*ent*-4c)

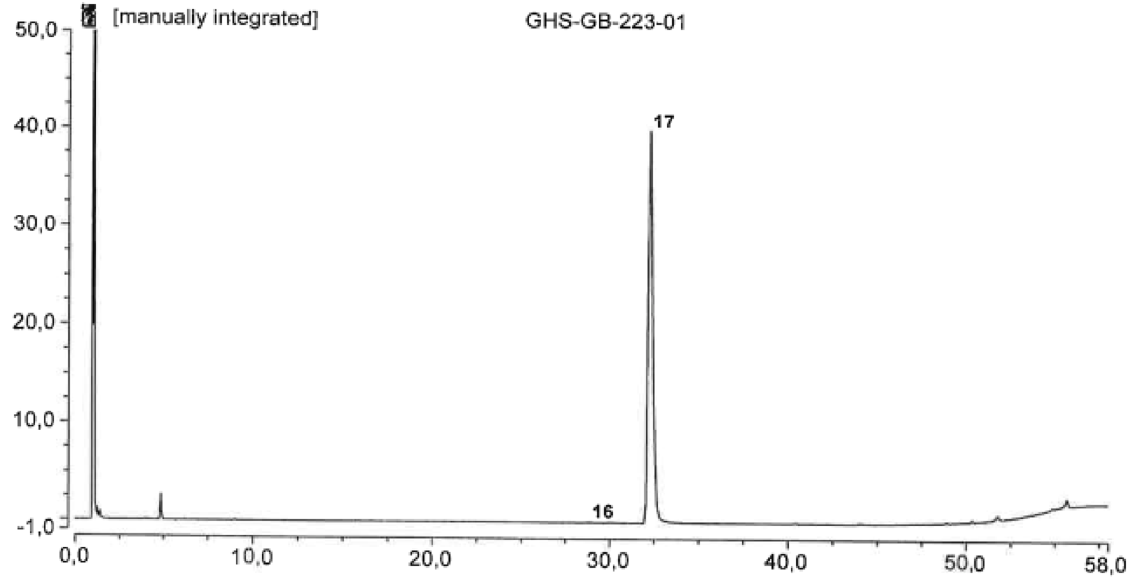

Sample:	**GHS-GB-223-01**	Instrument:	**GC_112**
Sequenz:	**8423 GHS-GB PH**	Measured:	**03.09.21 08:46**
Sequenz date:	**03.09.21**	Processing M.:	MPI
		Report-File:	Verhältnis

Enantiomerenverhältnis
Zuordnung nach achiral Messung

No.	Ret.Time min	Rel.Area %	Peak Name
16	28,88	0,09	.
17	32,22	99,91	.

Instrument parameters:

Column:	25,0 m	Hydrodex-gamma-TBDAc-CD 0,25/?df G/624
Temperature:	220 / 150, 40 min iso 6/min 240, 3 min iso / 350	
Gas:	0,60 bar	H2
Sample size:	0,2 µL	
